# Antioxidant and Antimutagenic Activities of Different Fractions from the Leaves of *Rhododendron arboreum* Sm. and Their GC-MS Profiling

**DOI:** 10.3390/molecules23092239

**Published:** 2018-09-03

**Authors:** Vandana Gautam, Sukhmeen Kaur Kohli, Saroj Arora, Renu Bhardwaj, Mohsin Kazi, Ajaz Ahmad, Mohammad Raish, Majid Ahmad Ganaie, Parvaiz Ahmad

**Affiliations:** 1 Department of Botanical and Environmental Sciences, Guru Nanak Dev University, Amritsar 143005, Punjab, India; vndu.gndu@gmail.com (V.G.); sukhmeenkohli@gmail.com (S.K.K.); sarojarora@gmail.com (S.A.); 2 Department of Pharmaceutics, College of Pharmacy, King Saud University, Riyadh 11451, Saudi Arabia; mkazi@ksu.edu.sa (M.K.); mraish@ksu.edu.sa (M.R.); 3 Department of Clinical Pharmacy, College of Pharmacy, King Saud University, Riyadh 11451, Saudi Arabia; aajaz@ksu.edu.sa; 4 Department of Pharmacology, College of Pharmacy, Prince Sattam bin Abdulaziz University, Alkharaj 11942, Saudi Arabia; majidsays@gmail.com; 5 Department of Botany and Microbiology, Faculty of Science, King Saud University, Riyadh 11451, Saudi Arabia; 6 Department of Botany, S.P. College, Srinagar 190001, Jammu and Kashmir, India

**Keywords:** antimutagenic activity, Ames assay, GC-MS, *Rhododendron arboreum* Sm., chemical composition, antioxidant activity

## Abstract

In this era of urbanization and environmental pollution, antioxidants and antimutagens derived from plants are promising safeguards for human health. In the current investigation, we analyzed the antioxidant and antimutagenic effects of the hexane, chloroform, and ethyl acetate fractions of *Rhododendron arboreum* Sm. leaves and determined their chemical composition. The different fractions inhibited lipid peroxidation, repressed the production of nitric oxide radicals, and prevented deoxyribose degradation. The antimutagenic activity of the leaf fractions was analyzed against 4-nitro-*O*-phenylenediamine, sodium azide and 2-aminofluorene mutagens in two test strains (TA-98 and TA-100) of *Salmonella typhimurium.* The experiment was conducted using pre- and co-incubation modes. The best results were obtained in the pre-incubation mode, and against indirect acting mutagen. The presence of a number of bioactive constituents was confirmed in the different fractions by GC-MS analysis. The study reveals the strong antioxidant and antimutagenic activity of *R. arboreum* leaves. We propose that those activities of *R. arboreum* might correspond to the combined effect of the phytochemicals identified by GC-MS analysis. To the best of our knowledge, this is the first report on the antimutagenic activity of *R. arboreum* leaves.

## 1. Introduction

In living systems, free radicals and other oxygen-derived species are produced constantly through the mitochondrial respiratory chain and other biological processes [1]. The oxygen-derived moieties include hydroxyl free radicals, singlet oxygen, lipid hydroperoxides, nitric oxide and other reactive oxygen species. All these species are partially reduced forms of atmospheric oxygen (O_2_). Under normal conditions, a balance is maintained between the production and scavenging of reactive oxygen species (ROS). An imbalance between the two results in an oxidative burst, which leads to oxidative stress [2]. The major causative agents of oxidative stress are pollutants, chemicals, radiations, toxins, physical stress, and spicy food. The over production of ROS results in increased lipid peroxidation in cell wall, causes damage to DNA, RNA, proteins and alters the calcium and sulfhydryl homeostasis [3]. Other consequences of ROS include an increase in cell proliferation and cell death, pathological neuron deterioration leading to Alzheimer’s disease, oxidation of low-density lipoprotein (LDL) resulting in atherosclerosis, gastritis, cancer, central nervous system injury, cardiovascular diseases, diabetes, ageing, and neurodegenerative diseases [4,5,6].

Antioxidants protect us from various diseases caused by oxidative stress including the DNA mutations [7]. They are also used as food supplements, flavor enhancers, and preservatives for increasing the shelf life of food. However, synthetic antioxidants have several harmful side effects; there is, therefore, a need to identify, isolate, characterize, and utilize natural herbal sources of antioxidants and antimutagens [8]. Plants are rich in secondary metabolites with antioxidant and antimutagenic potential.

*Rhododendron arboreum* Sm. is an evergreen tree belonging to the Ericaceae family. The young leaves of *R. arboreum* are said to be poisonous if consumed in large quantities [9]. It is not listed among potential harmful plants on the Horticultural Trades Association list [10]; therefore, the possibility of human poisoning is extremely low. *R. arboreum* is used in traditional medicinal systems but there are not many scientific reports to confirm its medicinal properties. Keeping this in mind, the present study has been undertaken to investigate the chemical composition of chloroform, hexane, and ethyl acetate fractions of *Rhododendron arboreum* Sm. leaves and to investigate their antioxidant potential in reference to lipid peroxidation, nitric oxide scavenging, and deoxyribose degradation. We also determined the antimutagenic activity of these fractions. We compared its efficacy with that of recognized mutagenic chemicals by using AMES assay to decipher the scientific basis behind its traditional use. This is the first report on the antimutagenic potential of this plant.

## 2. Results

The present study investigated the antioxidant and antimutagenic potential as well as the bioactive phytoconstituents of hexane, chloroform, and ethyl acetate fractions of *R. arboreum* leaves. The following results were observed:

### 2.1. Antioxidant Activity

#### 2.1.1. Nitric Oxide Quenching Assay

**Figure 1:**
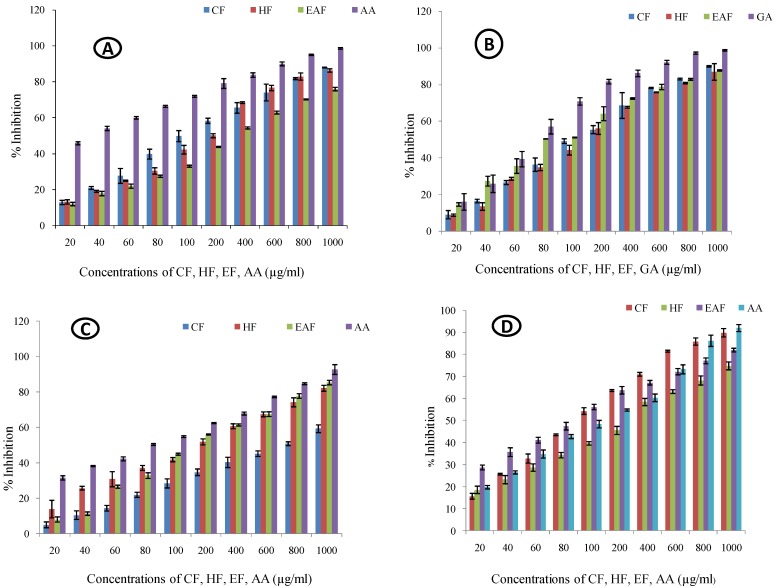
Antioxidant activity of *Rhododendron arboreum* fractions: (**A**) Nitric oxide scavenging assay; (**B**) Lipid peroxidation assay; (**C**) Site specific deoxyribose deprivation assay and; (**D**) Non- site specific deoxyribose deprivation assay. (CF- chloroform fraction; HF- hexane fraction; EAF- ethyl acetate fraction; AA- ascorbic acid; GA- gallic acid).

**Figure 2:**
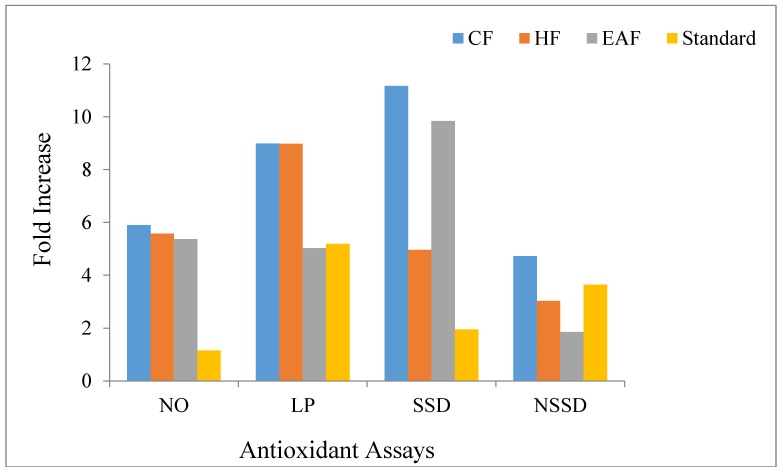
Assay wise fold increase in antioxidant activity of different fractions. [CF: chloroform fraction; HF: hexane fraction; EAF: ethyl acetate fraction; NO: Nitric oxide scavenging assay; LP: Lipid peroxidation assay; SSD: Site specific deoxyribose deprivation assay and; NSSD: Non- site specific deoxyribose deprivation assay].

In the nitric oxide quenching assay, an increase in percentage quenching was noted with the rise in concentration (Figure 1A). The chloroform fraction showed the maximum fold-increase (5.894) with the minimum IC_50_ value (150.133 µg/mL) compared to those of hexane (fold increase = 5.581, IC_50_ = 170.226 µg/mL) and ethyl acetate (fold-increase = 5.372, IC_50_ = 267.067 µg/mL) fractions (Table 1 and Figure 2).

#### 2.1.2. Lipid Peroxidation Assay

In the lipid peroxidation assay, all the fractions showed an increase in the percentage inhibition in a dose dependent manner (Figure 1B). The chloroform fraction showed the maximum fold-increase (8.98) with an IC_50_ value of 155.73 µg/mL. The hexane fraction showed a fold-increase of 8.97 with an IC_50_ value of 167.57 µg/mL. However, the ethyl acetate fraction showed the minimum fold-increase of 5.02 but the IC_50_ value of ethyl acetate fraction was minimum (116.02 µg/mL) compared to those of the chloroform and hexane fractions (Table 1 and Figure 2).

#### 2.1.3. Site-Specific Deoxyribose Deprivation Assay

The inhibitory activity of all the fractions against site-specific deprivation of deoxyribose increased with the increase in concentration (Figure 1C). The chloroform fraction showed the maximum fold-increase (11.163) in the site-specific deoxyribose deprivation assay but it had the maximum IC_50_ value (698.372 µg/mL) compared to those of the hexane (fold-increase = 4.959, IC_50_ = 181.585 µg/mL) and ethyl acetate (fold-increase = 9.84, IC_50_ = 191.907 µg/mL) fractions (Table 1 and Figure 2).

#### 2.1.4. Non-Site Specific Deoxyribose Deprivation Assay

All the fractions showed significant inhibition in the non-site-specific deoxyribose deprivation assay and the activity increased with the increase in the concentration (Figure 1D). The ethyl acetate fraction demonstrated the maximum protective activity (fold-increase = 1.85) and minimum IC_50_ value (96.817 µg/mL). The chloroform fraction showed a fold-increase of 4.726 and an IC_50_ value of 118.353 µg/mL. Hexane fraction caused a fold-increase of 3.026 and the IC_50_ was 226.412 µg/mL (Table 1 and Figure 2).

### 2.2. Antimutagenic Activity

**Figure 3:**
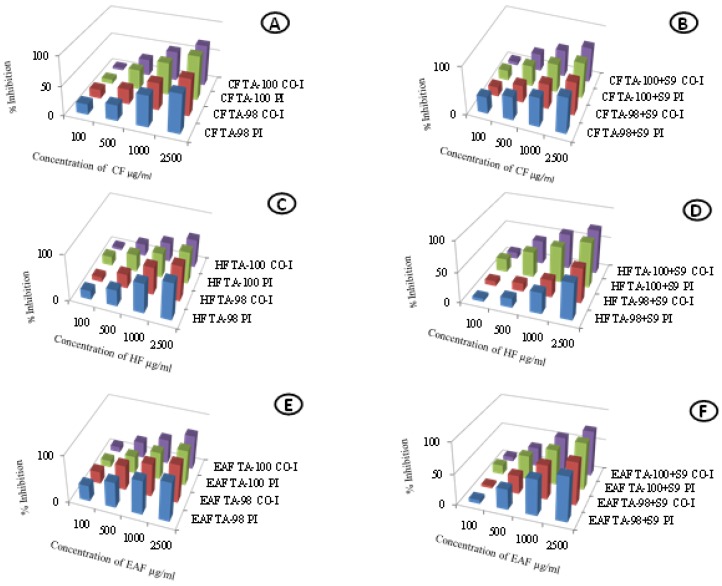
Antimutagenic activity of *Rhododendron arboreum* fractions in TA-98 and TA-100 strains of *Salmonella typhimurium*. Pre-incubation (PI) and co-incubation (Co-I) modes of experimentation; without metabolic activation against direct acting mutagens, 4-nitro-*O*-phenylenediamine for TA-98 & sodium azide for TA-100 and with metabolic activation against 2-aminofluorene using rat liver homogenate (S9). (**A**) Chloroform fraction (CF) without metabolic activation; (**B**) Chloroform fraction (CF) with metabolic activation; (**C**) Hexane fraction (HF) without metabolic activation; (**D**) Hexane fraction (HF) with metabolic activation; (**E**) Ethyl acetate fraction (EAF) without metabolic activation; (**F**) Ethyl acetate fraction (EAF) with metabolic activation.

The antimutagenic activity of *R. arboreum* leaf fractions as deduced from Ames assay is shown in Figure 3. The percentage inhibition of mutagenicity ranged from low to high, depending upon the per-plate concentration of the *Rhododendron* fractions. The fractions significantly reduced the number of induced histidine revertant colonies in the pre-incubation mode whereas, comparatively, lower efficiency of reducing the mutagenicity was observed in the co-incubation mode in all the fractions. Moreover, better results were obtained against the S9 dependent mutagen (2-aminofluorene) through the metabolic activation than against the direct mutagens (NPD and sodium azide) without metabolic activation in both the tester strains.

In the chloroform fraction, maximum activity of 75.679% was observed in the TA-100 strain against the directly acting mutagen in the pre-incubation mode (Figure 3A.) whereas in the case of S9 dependent mutagen, maximum activity of 75.741% was observed with TA-98 strain in the pre-incubation mode (Figure 3B). In the hexane fraction, maximum activity of 81% was observed in the TA-98 strain in the pre-incubation mode against the directly acting mutagen (Figure 3C) whereas, maximum activity of 75.012% was observed with the TA-100 strain in the pre-incubation mode against the S9 dependent mutagen (Figure 3D). The activity of the ethyl acetate fraction against the directly acting mutagen was maximum (83.48%) for the TA-98 strain in the pre-incubation mode (Figure 3E) whereas against the indirectly acting mutagen, maximum activity of 76.869% was observed with the TA-100 strain in the pre-incubation mode (Figure 3F). The honestly significant difference (HSD) values and the F ratio for treatment, dose and, interaction between the treatment and dose obtained from the two-way analysis of variance are given in Table 2.

### 2.3. Analysis Using GC-MS

The GC-MS study reveals that in the *R. arboreum* leaves, the chloroform fraction contained 24 compounds, the hexane fraction contained 17 compounds, and the ethyl acetate fraction contained 26 compounds (Table 3). Of these, 3,7,11,15-tetramethyl-2-hexadecen-1-ol, linoleic acid, 9-octadecenoic acid, 3,10-epoxy-(3β,10β)-D:B-friedo-18,19-secolup-19-ene, methyl commate C, andmethyl commate D were present in all the three fractions. 2-Hexyl-1-decanol, Octadecyl Chloroacetate, 3-bromo-3β-cholest-5-ene, methyl commate B andolean-12-en-28-al were present in the chloroform and hexane fractions. 1-Tetradecene, neophytadiene, 3(*E*)-eicosene and 9(*E*)-eicosene were present in the chloroform and ethyl acetate fractions. 2,6,10,15,19,23-Hexamethyl- 2,6,10,14,18,22-tetracosahexaene, and vitamin E were present in the ethyl acetate and hexane fractions (Table 3). The existence of these constituents in the leaves of *R. arboreum* confirms its potential for use in the therapeutics.

## 3. Discussion

All the fractions of *Rhododendron arboreum* leaves showed promising effect in all the antioxidant assays. The results established that the radical quenching effects of the different fractions were dose dependent (Figure 1) The Pearson’s correlation coefficient data indicate that the nitric oxide value positively correlated with lipid peroxidation and site and non-site specific deoxyribose deprivation values at *p* < 0.05 (Table 4). The lipid peroxidation data positively correlate with the site-specific and non-site-specific deoxyribose deprivation data at *p* ≤ 0.05. A significant positive correlation was observed among the site-specific and non-site-specific deoxyribose deprivation values. The results suggest that *R. arboreum* leaves are proficient in protecting against oxidative stress. The possible reason for this could be the fact that the phytoconstituents of *R. arboreum* like vitamin E, 3,7,11,15-tetramethyl-2-hexadecen-1-ol, efficiently scavenge the free radicals [11,12].

The results also indicate that the leaf fractions have the potential to protect the DNA of the tester strains from frame shift or base pair mutations. The analysis of variance showed significant differences between the TA-100 and TA-98 strains (Table 2). The possible mechanism of action for this could be that some secondary metabolites like vitamin E [13,14] might act as direct anti-mutagens by reverting the toxic effect of mutagens or by inactivating them or they might hinder the direct contact of mutagens with the cells by making changes in their absorption rates. This could be achieved either by reacting with the mutagens and making nontoxic byproducts or by binding at the site of action of the mutagens, thereby, not allowing the mutagen to come in direct contact with the cell. It is evident from the results that the pre-incubation mode of experiment is more efficient in protecting the cells from mutation. This proves that during the incubation period, the phytoconstituents of the *R. arboreum* leaf extracts might react with the mutagen to make it less toxic or nontoxic. The antimutagenicity of several plant extracts has been reported against several mutagens, such as sodium azide, 2-aminofluorene, 2-nitrofluoranthene, and heterocyclic amines [15,16,17,18]. However, to the best of our knowledge, this is the first report regarding the antimutagenic activity of *R. arboreum* leaf fractions against sodium azide, 4-nitro-*O*-phenylenediamine, and 2-aminofluorene in the two tester strains (TA-98 and TA-100) of *S. typhimurium*. To find out the causative agents responsible for these antioxidant and antimutagenic activities, the phytoconstituents of the fractions were identified using the GC-MS technique. We could identify 24 compounds in the chloroform fraction, 17 in the hexane fraction, and 26 in the ethyl acetate fraction. These compounds are reported to possess different activities. For example, 9-octadecenoic acid has antimicrobial and antibacterial potential [19]. A literature review shows that vitamin E exhibits curative potential against leukemia, tumors, cancer, dermatitis, ulcers and inflammation along with antiaging, analgesic, antidiabetic, vasodilatory, antispasmodic, antibronchitic, antiplasmodial, antimicrobial, and anti-inflammatory properties [20,21]. 1,2-Benzenedicarboxylic acid has antimicrobial properties [14]. 9,12-Octadecadien-1-ol possesses anti- arthritic and anti-inflammatory properties [21]. Octadecene is reported to exhibit anticancer, antioxidant, and antimicrobial activities [22,23]. *Candida albicans* uses farnesol as a sensing molecule to stop filamentation [24]. (3β)-Stigmast-5-en-3-ol helps in cholesterol lowering [25] and curing proliferation [26]. (3β)-Stigmast-5-en-3-ol is also used as anti-diabetic agent that controls glucose transport. It restores the uptake of glucose without the stimulus of insulin, therefore, indicating insulin-like property [27]. The presence of steroids, terpenoids, flavonoids, anthraquinones, tannins, phlobatannins in leaves of *R. arboreum* have been reported [28]. In *Rhododendron arboreum* Sm. ssp. nilagiricum (Zenker) Tagg. leaves, phenols, flavonoids, quinones, steroids, tannins, xanthoproteins and coumarins are reported [29]. The presence of these compounds in the leaves of *R. arboreum* indicates that they are promising candidates for therapeutic use. 9,12-Octadecadienoic acid is helpful in curing inflammation, microbial infection and arthritis [30]. Linoleic acid, heptadecanoic acid, and oleic acid possess antimicrobial activities [31].

## 4. Materials and Methods

### 4.1. Sample Preparation

The leaves were procured from Kataula village in the Kullu district of Himachal Pradesh and were authenticated at the herbarium of Guru Nanak Dev University, Amritsar. The leaves were washed with double distilled water, dried in shade, and ground to a fine powder in a mixer–grinder. The 1 kg powder was then extracted with 80% methanol to get 68.38 g (6.838%) crude extract, 50 g of which was further fractionated using different solvents (polarity wise) to obtain 13.53 g (27.06%) hexane fraction, 15.03 g (30.06%) chloroform fraction and 17.32 g (34.64%) ethyl acetate fraction. The fractions were dried out with the help of rotary vacuum evaporator at a temperature of 30 degree Celsius. For antioxidant assays, 1000 μg/mL stock solutions of different fractions were prepared, which were further used to make different concentrations (20, 40, 60, 80, 100, 200, 400, 600, 800, 1000 μg/mL) by serial dilution. To assess the antimutagenic activity, 100, 500, 1000 and 2500 μg/mL concentrations of different fractions were prepared.

### 4.2. Antioxidant Activity

#### 4.2.1. Lipid Peroxidation Assay

Lipid peroxidation assay was performed using the method described by Halliwell and Gutteridge [32] after slight modifications. The leaf fractions (1 mL) at different concentrations were mixed with 0.5 mL KCl and 0.5 mL rat liver homogenate. To this mixture, 100 µL FeCl_3_ was added to induce lipid peroxidation. After incubating for 30 min at 37 °C, 2 mL of ice cold 0.25 N HCl with 15% trichloroacetic acid (TCA) and 0.5% thiobarbituric acid (TBA) was added to the mixture. Thereafter, 50 µL butylated hydroxytoluene (BHT) was added, followed by incubation of 60 min at 100 °C. After centrifugation of 3 min at 900 g optical density was noted at 532 nm. Calculation was done as follows:

$$\%\;\mathrm{Inhibition}=\frac{Ac-As}{Ac}\times 100$$

*Ac* = Control absorbance

*As* = Sample absorbance

#### 4.2.2. Nitric Oxide Quenching Assay

The nitric oxide quenching activity was estimated according to the technique given by Marcocci et al. [33], with minor alterations. 1 mL of each fraction was mixed with 0.5 mL sodium nitroprusside (5 mM) and incubated at 25 °C for 30 min. Subsequently, 1.5 mL Griess reagent was mixed with the above solution and the absorbance was read at 546 nm. The percentage quenching of nitric oxide was estimated as:

$$\%\;\mathrm{quenching}=\frac{Ac-As}{Ac}\times 100$$

*Ac* = Control absorbance

*As* = Sample absorbance

#### 4.2.3. Deoxyribose Deprivation Assay

Deoxyribose deprivation assay was conducted by the technique given by Halliwell et al. [34] and Arouma et al. [35] after slight modifications. This assay was divided into site and non-site-specific mode. In the site-specific mode, 100 µL of phosphate buffer was combined with 20 µL of FeCl_3_ (10 mM), 100 µL H_2_O_2_ (10 mM), 360 µL of 10 mM deoxyribose, 1mL of the fraction, 320 µL of phosphate buffer, and 100 µL of 1 mM of ascorbic acid. In the non-site-specific mode, 100 µL EDTA (1 mM) was mixed with 20 µL FeCl_3_ (10 mM), 100 µL H_2_O_2_ (10 mM), 360 µL of 10 mM deoxyribose, 1mL of the fraction, 320 µL of phosphate buffer and 100 µL of 1 mM ascorbic acid. After incubating at 37 °C for 1 h, 1 mL of the above solution was mixed with 1 mL TCA (10%) and 1 mL TBA (0.5%). The mixture was heated for 90 min on a water bath at 80 °C until pink color was formed which was read at 532 nm with spectrophotometer. The calculation was done as follows:

$$\%\;\mathrm{Inhibition}=\frac{Ac-As}{Ac}\times 100$$

*Ac* = Control absorbance

*As* = Sample absorbance

### 4.3. Antimutagenic Activity

The tester strains TA-98 and TA-100 of *S. typhimurium* were used to examine the antimutagenic potential of the hexane, chloroform, and ethyl acetate fractions of *R. arboreum* leaves. The method suggested by Maron and Ames [36] was employed for the assay incorporating slight alterations given by Bala and Grover [37]. For TA-98, 4-nitro-*O*-phenylenediamine (NPD) and for TA-100, sodium azide (NaN_3_) was used as the direct acting mutagen. The different concentrations of plant fractions (100, 500, 1000, 2500 µg/0.1 mL) were prepared in dimethyl sulfoxide (DMSO) and were analyzed using pre-incubation and co-incubation modes of the experiment so that the bio-antimutagenicity and desmutagenicity could be differentiated. We used 2-aminofluorene (2-AF) as a promutagen in the presence of liver homogenate of rat (S9 mix) [38]. The liver homogenate activates the promutagen through the cytochrome P450 metabolic activation system. The antimutagenic activity was calculated as:

$$\%\;\mathrm{Inhibition}=\frac{X-Y\;}{X-Z}\times 100$$

X = number of histidine revertants induced by mutagen alone (+ve control). Y = number of histidine revertants induced by mutagen in the presence of extract/fraction. Z = number of histidine revertants induced by extract/fraction alone (− ve control).

### 4.4. Analysis Using GC-MS

The hexane, chloroform, and ethyl acetate fractions of *R. arboreum* leaves were analyzed using a GCMS-QP2010 Plus system (Shimadzu, Kyoto, Japan). The instrument conditions and parameters used for analysis were as follows: Column oven temperature: 70 °C for first 5 min; increased to 250 °C at a rate of 10 °C/min and held for next 10 minutes; further increased to 300°C at a rate of 10 °C/min and held at this temperature for 10 min. Carrier gas: Helium. Injection temperature: 280 °C. Injection mode: Splitless. Sampling time: 1 min. Pressure: 110.8 kPa. Total flow: 38.9 mL/min. Column flow: 1.71 mL/min. Linear velocity: 47.9 cm/sec. Purge flow: 3 mL/min. All the discovered compounds were recognized by evaluating their mass spectra with those listed in the National Institute of Standard and Technology (NIST08s) and Wiley 7 libraries.

### 4.5. Statistical Analysis

Results are given as mean of three replications ± standard error. For the interactions at 5% level of significance, 1-way and 2-way analysis of variance (ANOVA) were performed using MS Excel. Pearson’s correlation coefficients were also calculated.

## 5. Conclusions

The results reveal that the different leaf fractions of *R. arboreum* inhibit lipid peroxidation, repress the nitric oxide radicals, and prevent the deprivation of deoxyribose. *Rhododendron arboreum* leaves also possess considerable antimutagenic activity in against base-pair and frame-shift mutations. These activities can be credited to the synergistic effect of different phytoconstituents of leaves. Based on the results of present study, it is suggested that the selected components of *R. arboreum* leaves may be used as food additives or leaves can serve as raw material for development of medicines.

## Figures and Tables

**Table 1:** Percent Inhibition, IC_50_ fold increase activity, one-way ANOVA F-ratio and HSD value of different fractions in antioxidant assays.

Fractions		NO	LP	SS DDA	NSS DDA
Chloroform Fraction	%I (20 µg/mL)	12.749 ± 1.19	9.0 ± 2.266	4.862 ± 1.62	15.682 ± 1.318
	%I (1000 µg/mL)	87.905 ± 0.224	89.91 ± 0.395	59.151 ± 2.122	89.803 ± 1.893
	IC_50_	150.133	155.732	698.372	118.353
	Fold Increase	5.894	8.989	11.163	4.726
	F-Ratio (9,20)	295.564 *	319.124 *	247.581 *	1298.927 *
	HSD	7.61	8.03	5.773	3.647
Hexane Fraction	%I (20 µg/mL)	13.106 ± 1.2	8.704 ± 0.617	13.758 ± 4.943	18.565 ± 1.674
	%I (1000 µg/mL)	86.267 ± 0.895	86.844 ± 4.54	81.991 ± 1.689	74.753 ± 1.794
	IC_50_	170.226	167.57	181.558	226.412
	Fold Increase	5.581	8.977	4.959	3.026
	F-Ratio (9,20)	1171.209 *	505.111 *	237.822 *	460.541 *
	HSD	4.033	6.251	7.27	4.608
Ethylacetate Fraction	%I (20 µg/mL)	11.915 ± 0.917	14.54 ± 0.943	7.861 ± 1.441	28.745 ± 1.087
	%I (1000 µg/mL)	75.930 ± 0.988	87.668 ± 0.317	85.22 ± 1.256	81.939 ± 0.823
	IC_50_	267.067	116.029	191.907	96.817
	Fold Increase	5.372	5.029	9.84	1.85
	F-Ratio (9,20)	2410.284 *	452.876 *	1787.76 *	506.233 *
	HSD	2.337	5.865	3.194	4.063
Standard	%I (20 µg/mL)	45.755 ± 0.852	15.924 ± 4.532	31.376 ± 1.214	19.789 ± 0.792
	%I (1000 µg/mL)	98.599 ± 0.449	98.615 ± 0.395	92.577 ± 2.763	91.948 ± 1.585
	IC_50_	25.648	95.185	83.86	132.597
	Fold Increase	1.154	5.192	1.95	3.646
	F-Ratio (9,20)	698.461 *	317.475 *	1028.868 *	721.773 *
	HSD	3.388	8.537	3.192	4.54
NO = Nitric oxide scavenging assay			LP = Lipid peroxidation assay		
SS DDA = Site specific deoxyribose deprivation assay			NSS DDA = Non site specific deoxyribose deprivation assay		
%I = Percent Inhibition			IC_50_ = 50% Inhibition concentration (µg/mL)		
Values are given as mean±standard error			HSD = Honestly significant difference		
* significant at *p* ≤ 0.001 level of significance			ANOVA = Analysis of variance		

**Table 2:** The honestly significant difference (HSD) values and F ratio for treatment, dose and, interaction between treatment and dose obtained from 2-way analysis of variance.

	Degree of Freedom	F-Ratio			
		(TA-98)	(TA-98+S9)	(TA-100)	(TA-100+S9)
**Chloroform Fraction**					
F ratio Treatment	1, 40	23.19 ***	482.9 ***	97.69 ***	160.8 ***
F ratio Dose	3, 40	1017 ***	1384 ***	3484 ***	2267 ***
F ratio Treatment × Dose	3, 40	3.171 **	13.14 ***	0.748	25.08 ***
HSD		154.2	131.8	115.7	116.2
**Hexane Fraction**					
F ratio Treatment	1, 40	24.84 ***	2.475	373.4 ***	88.68 ***
F ratio Dose	3, 40	611.1 ***	398.5 ***	1367 ***	707.1 ***
F ratio Treatment × Dose	3, 40	0.67	2.606 *	3.294 **	18.11 ***
HSD		297.4	159	142.2	187.3
**Ethyl Acetate Fraction**					
F ratio Treatment	1, 40	42.24 ***	2.399 *	37.12 ***	22.94 ***
F ratio Dose	3, 40	1407 ***	319.1 ***	1607 ***	791 ***
F ratio Treatment × Dose	3, 40	4.286 **	0.018	1.502	3.663 **
HSD		153.2	370.1	164.5	240.4

* significant at *p* ≤ 0.100; ** significant at *p* ≤ 0.05; *** significant at *p* ≤ 0.001 level of significance.

**Table 3:** Phytochemicals present in chloroform, hexane and ethyl acetate fractions of *Rhododendron arboreum* leaves.

No.	Chloroform Fraction	Hexane Fraction	Ethyl Acetate Fraction
1	1-Dodecene	3,7,11,15-Tetramethyl-2-hexa-decen-1-ol	1-Dodecene
2	1-Tetradecene	14-Methylpentadecanoic acid, methyl ester	1-Tetradecene
3	Docosanoic acid	9-Octadecenoic acid	*n*-Tetradecane
4	1-Nonadecene	3,7,11,15-tetramethyl-2-hexadecen-1-ol,	2,4-DI-Tert-butylphenol,
5	Neophytadiene	Linoleic acid	9-(*E*)-Eicosene,
6	3,7,11,15-Tetramethyl-2-hexa-decen-1-ol	Docosanoic acid	9-Octadecenoic acid
7	Pentadecanoic acid	1,2-Benzenedicarboxylic acid, ditridecyl ester	3-(*E*)-Eicosene
8	3-(*E*)-Eicosene,	2-Hexyl-1-decanol	Neophytadiene
9	3,7,11,15-Tetramethyl-, 2-hexadecen-1-ol, [*R*-[R*,R*-(*E*)]] phytol	2,6,10,14,18,22-Tetracosa-hexaene	3,7,11,15-Tetramethyl-2-hexadecen-1-ol
10	Linoleic acid	Octadecyl Chloroacetate	2-Methyl-7-octadecyne
11	9-Octadecenoic acid	Vitamin E	Butyl-2-methylpropylphthalate
12	9-(*E*)-Eicosene	3,10-Epoxy-D:B-friedo-18,19-secolup-19-ene	Pentadecanoic acid
13	Heptadecyl trifluoroacetate	3-Bromo-(3β)-cholest-5-ene	Pentadecyl trifluoroacetate
14	Hexatriacontane	Methyl commate C	3,7,11,15-Tetramethyl-2-hexadecen-1-ol
15	2-Hexyl-1-decanol,	Methyl commate B	Linoleic acid
16	Docosyl pentafluoropropionate	Methyl commate D	Eicosanoic acid
17	Farnesol isomer a	Olean-12-en-28-al	9-Tricosene
18	Octadecyl Chloroacetate		1-Docosanol behenic alcohol
19	3,10-Epoxy-D:B-friedo-18,19-secolup-19-ene		1,2-Benzenedicarboxylic acid,
20	Cholest-5-ene		Nonadecyl pentafluoropropionate
21	Methyl commate C		Vitamin E
22	Methyl commate B		3,10-Epoxy-(3β,10β)-D:B-friedo-18,19-secolup-19-ene
23	Methyl commate D		3β-Stigmast-5-en-3-ol
24	Olean-12-en-28-al		Methyl commate C
25			Methyl commate D
26			Urs-12-en-28-ol

**Table 4:** Pearson’s correlation coefficients (R) among antioxidant activity (NO = Nitric oxide scavenging, LP = Lipid peroxidation, SSDD = Site specific deoxyribose deprivation, NSSDD = Non-site specific deoxyribose deprivation).

	NO	LP	SSDD	NSSDD
NO	1			
LP	0.9979 *	1		
SSDDA	0.9971 *	0.9946 *	1	
NSSDDA	0.9983 *	0.9980 *	0.9923 *	1

* Correlations significant at *p* ≤ 0.01.

## Supplementary Materials

The following are available online: Supplementary file S-1-Tables: GC-MS tables of *Rhododendron arboreum* leaf fractions with retention times of different compounds; Supplementary file S-1-Histograms: Histograms for the antimutagenic activity of different fractions of *Rhododendron arboreum* leaves; Supplementary files S-3: GCMS report Chloroform leaves; Supplementary files S-4: GCMS report Hexane leaves; Supplementary files S-5: GCMS report Ethyl Acetate leaves.
