# Supplementary material for: Antioxidant and Antimutagenic Activities of Different Fractions from the Leaves of Rhododendron arboreum Sm. and Their GC-MS Profiling
Source: Molecules. 2018 Sep 3;23(9):2239. doi: 10.3390/molecules23092239 (PMC6225473; doi:10.3390/molecules23092239)
Supplement: Supplementary file 1 [file molecules-23-02239-s001.pdf]

molecules

MDPI

Article

Antioxidant and Antimutagenic Activities of Different Fractions from the Leaves of *Rhododendron arboreum* Sm. and their GC-MS Profiling

Vandana Gautam[1], Sukhmeen Kaur Kohli[1], Saroj Arora[1], Renu Bhardwaj[1], Mohsin Kazi[2], Ajaz Ahmad[3], Mohammad Raish[2], Majid Ahmad Ganaie[3], Parvaiz Ahmad[4,5,*]

1 Department of Botanical and Environmental Sciences, Guru Nanak Dev University, Amritsar-143005, Punjab, India
2 Department of Pharmaceutics, College of Pharmacy, King Saud University, Riyadh 11451, Saudi Arabia
3 Department of Pharmacology, College of Pharmacy, Prince Sattam bin Abdulaziz University, Alkharaj, Saudi Arabia
4 Department of Botany and Microbiology, Faculty of Science, King Saud University, Riyadh 11451, Saudi Arabia
5 Department of Botany, S.P. College, Srinagar, 190001, Jammu and Kashmir, India
* Corresponding author E-mail: renubhardwaj82@gmail.com; parvaizbot@yahoo.com

Supplementary Materials:

Supplementary file S-1-Tables

Table S1. Retention time and % area of the phytochemicals detected in chloroform fraction of *R. arboreum* leaves by GC-MS.

**Chloroform Fraction**			
Sr. No.	Compound	% Area	Retention Time (Minutes)
1	1-Dodecene	0.08	8.049
2	1-Tetradecene	0.22	12.029
3	Docosanoic acid	0.37	14.815
4	1-Nonadecene	0.47	17.144
5	Neophytadiene	0.93	17.640
6	3,7,11,15-Tetramethyl-2-hexadecen-1-ol	0.24	18.070
7	Pentadecanoic acid	1.44	18.845
8	3-Eicosene, (E)	0.47	19.211
9	2-Hexadecen-1-ol, 3,7,11,15-tetramethyl-, [R-[R*,R*-(E)]] phytol	0.55	20.252
10	Linoleic acid	0.23	20.459
11	9-Octadecenoic acid	0.41	20.708
12	9-Eicosene, (E)	0.35	21.088
13	Heptadecyl trifluoroacetate	0.30	22.809
14	Hexatriacontane	0.27	23.682
15	1-Decanol, 2-hexyl	0.21	24.417
16	Tetratetracontane	2.64	25.404
17	Docosyl pentafluoropropionate	0.14	26.372
18	Farnesol isomer a	0.72	26.574
19	Acetic acid, chloro-, octadecyl ester	0.37	27.518
20	Pentatriacontane	1.34	27.765
21	Stearyl alcohol P1298	2.26	31.128
22	D:B-Friedo-18,19-secolup-19-ene, 3,10-epoxy	8.30	33.685
23	Cholest-5-ene	2.22	35.174

24	Methyl commate C	7.58	35.429
25	Methyl commate B	0.50	35.593
26	Methyl commate D	35.62	36.302
27	Flavone 4'-OH,5-OH,7-di-O-glucoside	2.02	37.189
28	Globulol	27.81	37.966
29	Olean-12-en-28-al	1.93	39.895

Table S2. Retention time and % area of the phytochemicals detected in hexane fraction of *R. arboreum* leaves by GC-MS.

**Hexane Fraction**			
Sr. No.	Compound	% Area	Retention Time
1	3,7,11,15-Tetramethyl-2-hexadecen-1-ol	0.14	17.643
2	Pentadecanoic acid, 14-methyl-, methyl ester	0.10	18.415
3	9-Octadecenoic acid	0.62	18.827
4	2-Hexadecen-1-ol, 3,7,11,15-tetramethyl	0.46	20.257
5	Linoleic acid	0.17	20.451
6	Docosanoic acid	0.10	20.699
7	Octadecane	0.08	23.684
8	1,2-Benzenedicarboxylic acid, ditridecyl ester	0.09	23.745
9	2-hexyl-1-Decanol	0.03	24.421
10	Tetratetracontane	0.39	25.393
11	2,6,10,14,18,22-Tetracosahexaene	0.95	26.579
12	Acetic acid	0.17	27.529
13	Octadecane	0.38	27.760
14	Vitamin E	1.34	31.152
15	D:B-Friedo-18,19-secolup-19-ene, 3,10-epoxy	9.82	33.717
16	Cholest-5-ene, 3-bromo-, (3.beta.)	1.81	35.186
17	Methyl commate C	8.65	35.445
18	Methyl commate B	0.60	35.613
19	Methyl commate D	37.46	36.328
20	Flavone 4'-OH,5-OH,7-di-O-glucoside	1.86	37.206
21	Globulol	33.20	37.999
22	Olean-12-en-28-al	1.59	38.952

Table S3. Retention time and % area of the phytochemicals detected in ethyl acetate fraction of *R. arboreum* leaves by GC-MS.

**Ethyl acetate Fraction**			
Sr. No.	Compound	% Area	Retention Time
1	1-Dodecene	0.54	8.074
2	1-Tetradecene	0.91	12.040
3	n-Tetradecane	0.14	12.210
4	Phenol, 2,4-bis(1,1-dimethylethyl)-2,4-di-tert-butylphenol	0.86	13.586
5	9-Eicosene, (E)	1.12	14.826
6	9-Octadecenoic acid	0.17	16.732
7	3-Eicosene, (E)	1.16	17.154
8	Neophytadiene	1.13	17.644
9	3,7,11,15-Tetramethyl-2-hexadecen-1-ol	0.18	17.892
10	7-Octadecyne, 2-methyl	0.30	18.076
11	Butyl-2-methylpropylphthalate	0.41	18.494
12	Pentadecanoic acid	1.29	18.845
13	Pentadecyl trifluoroacetate	0.77	19.220
14	2-Hexadecen-1-ol, 3,7,11,15-tetramethyl	0.65	20.262
15	Linoleic acid	0.19	20.463
16	Eicosanoic acid	0.34	20.716
17	9-Tricosene	0.49	21.096
18	1-Docosanol behenic alcohol	0.28	22.814
19	1,2-Benzenedicarboxylic acid,	0.30	23.751
20	Nonadecyl pentafluoropropionate	0.19	24.423
21	Octadecane	0.38	25.395
22	2,6,10,14,18,22-Tetracosahexaene, 2,6,10,15,19,23-hexamethyl	0.75	26.584
23	17-Pentatriacontene	0.26	27.536
24	Nonadecane	0.38	27.766

25	Vitamin E	1.63	31.156
26	D:B-Friedo-18,19-secolup-19-ene, 3,10-epoxy-, (3.beta.,10.beta.)	9.07	33.729
27	Stigmast-5-en-3-ol	2.38	35.200
28	Methyl commate C	8.30	35.469
29	Methyl commate D	33.72	36.329
30	Flavone 4'-OH,5-OH,7-di-O-glucoside	2.08	37.215
31	Globulol	28.02	37.993
32	Urs-12-en-28-ol	1.62	39.919

Supplementary file S-2-Histograms

Chloroform fraction:

Conc	100	500	1000	2500
CF TA-98 PI	18.01845	26.77254	53.46721	66.5745
CF TA-98 CO-I	15.43121	25.79002	46.66907	63.60904
CF TA-100 PI	7.804655	33.9637	55.61623	75.67915
CF TA-100 CO-I	3.966986	28.19549	51.07319	70.39728
CF TA-98+S9 PI	34.87856	49.20484	61.37259	75.7416
CF TA-98+S9 CO-I	19.33826	35.85603	51.69903	68.66653
CF TA-100+S9 PI	20.96522	42.65198	58.6364	73.20182
CF TA-100+S9 CO-I	6.882624	35.40188	54.61313	71.50917

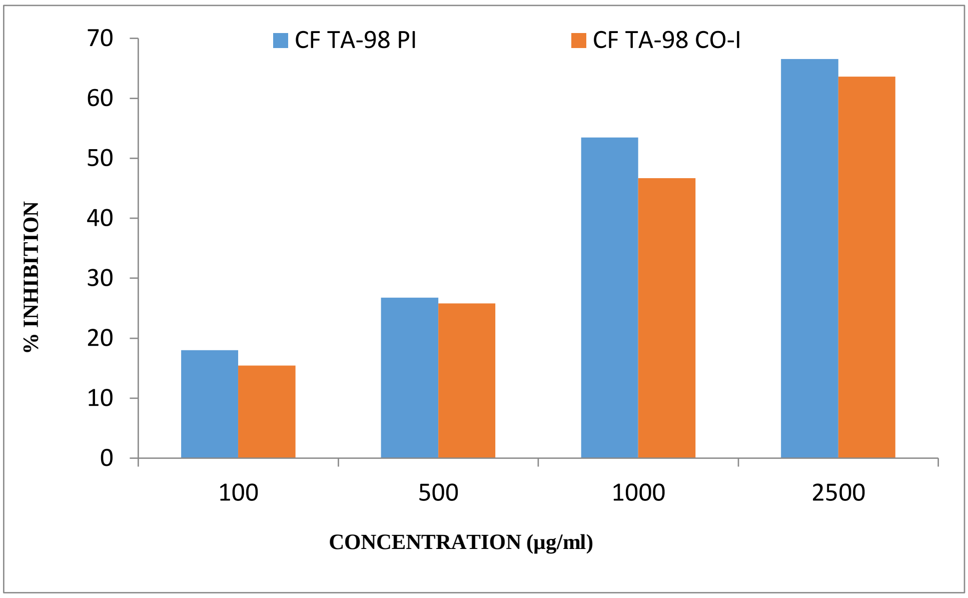

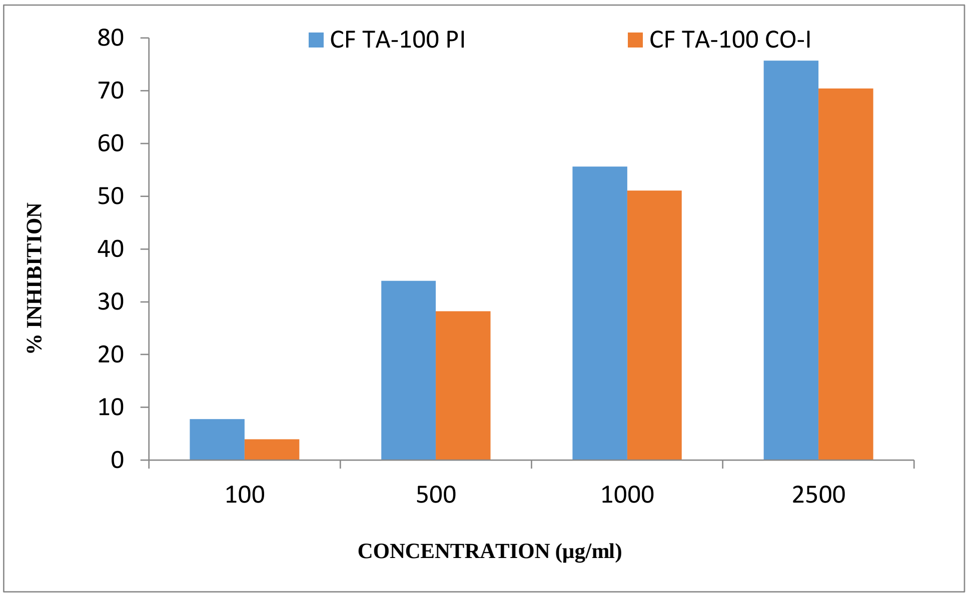

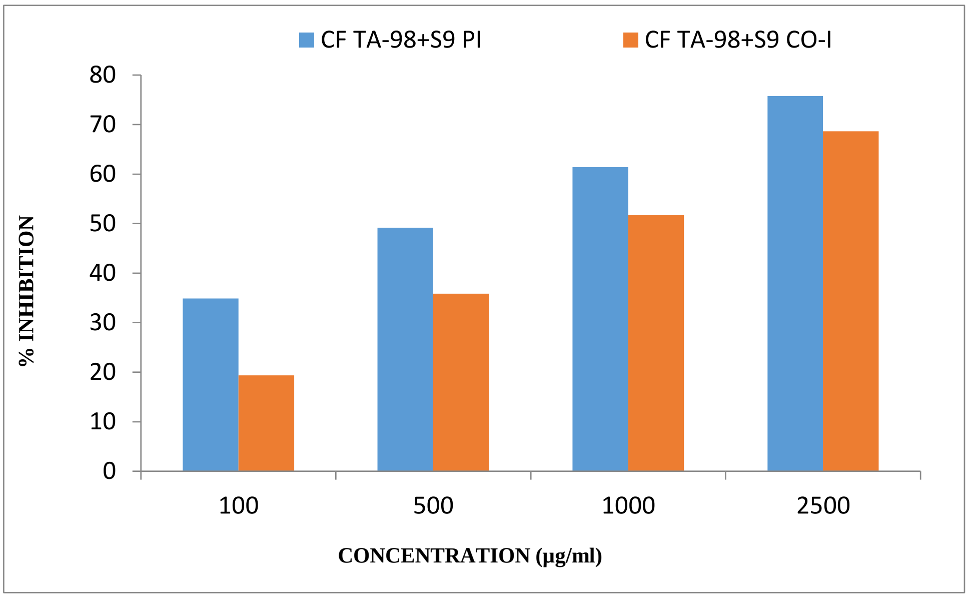
CF TA-98+S9 PI
CF TA-98+S9 CO-I
% INHIBITION
80
70
60
50
40
30
20
10
0
100
500
1000
2500
CONCENTRATION (µg/ml)

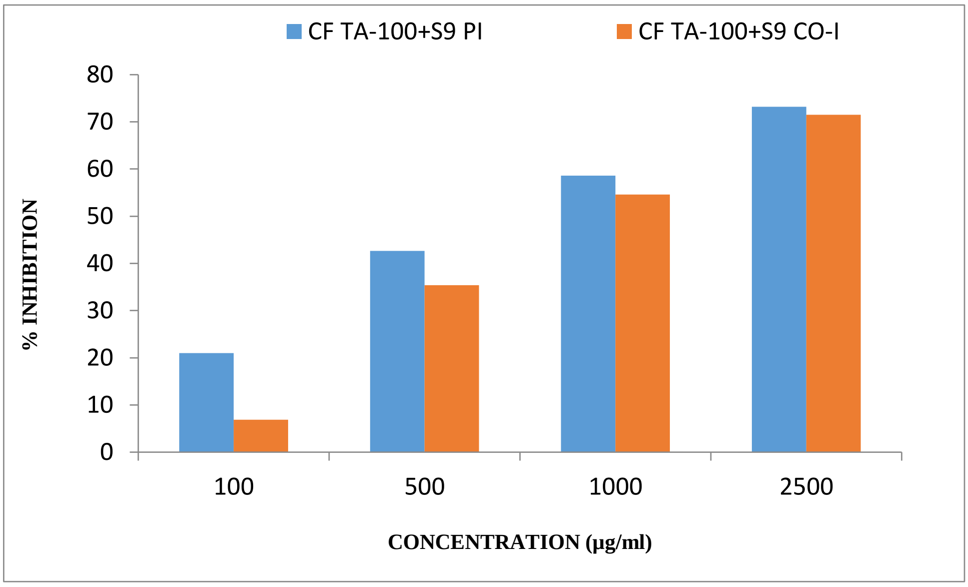
CF TA-100+S9 PI
CF TA-100+S9 CO-I
% INHIBITION
80
70
60
50
40
30
20
10
0
100
500
1000
2500
CONCENTRATION (µg/ml)

Hexane fraction:

Conc	100	500	1000	2500
HF TA-98 PI	19.14856	35.62746	65.0827	81.00343
HF TA-98 CO-I	10.51114	30.21797	60.06448	76.69399
HF TA-100 PI	19.65675	36.91592	52.67331	68.74717
HF TA-100 CO-I	5.335622	25.25726	40.78311	59.91256
HF TA-98+S9 PI	5.316654	15.00586	34.60336	60.19997
HF TA-98+S9 CO-I	8.183477	13.84655	28.48118	57.33022
HF TA-100+S9 PI	22.1939	42.50586	59.78708	75.01208
HF TA-100+S9 CO-I	10.75908	39.48475	58.74667	73.78824

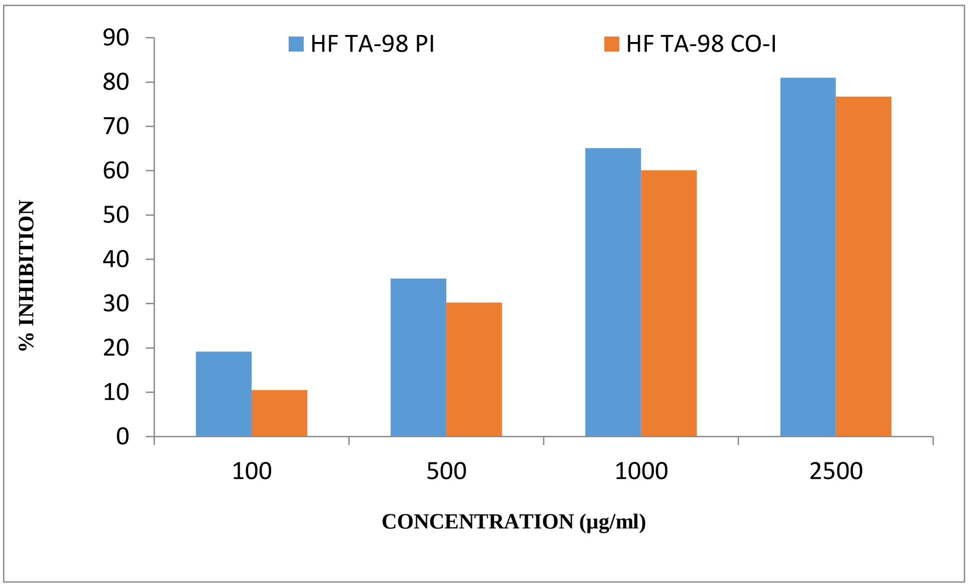

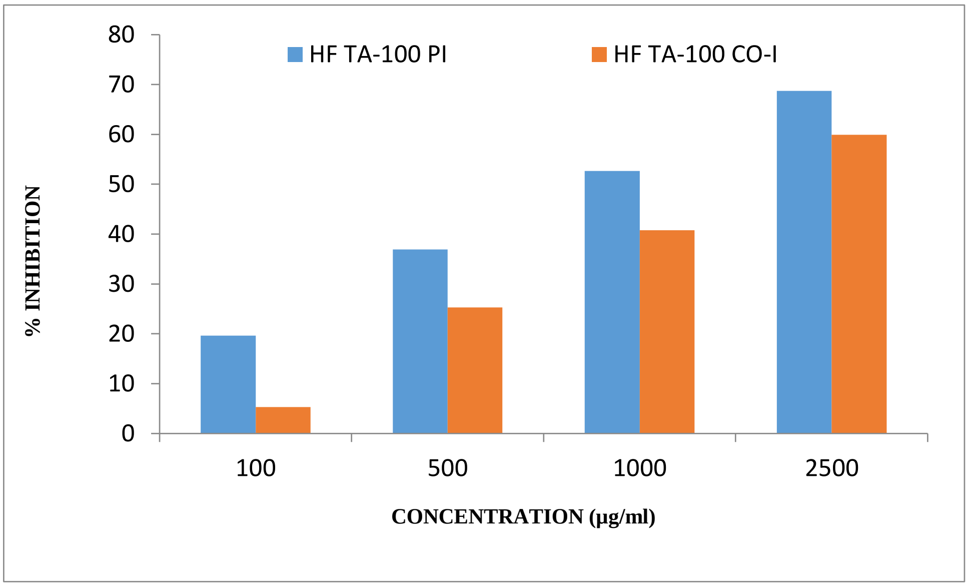

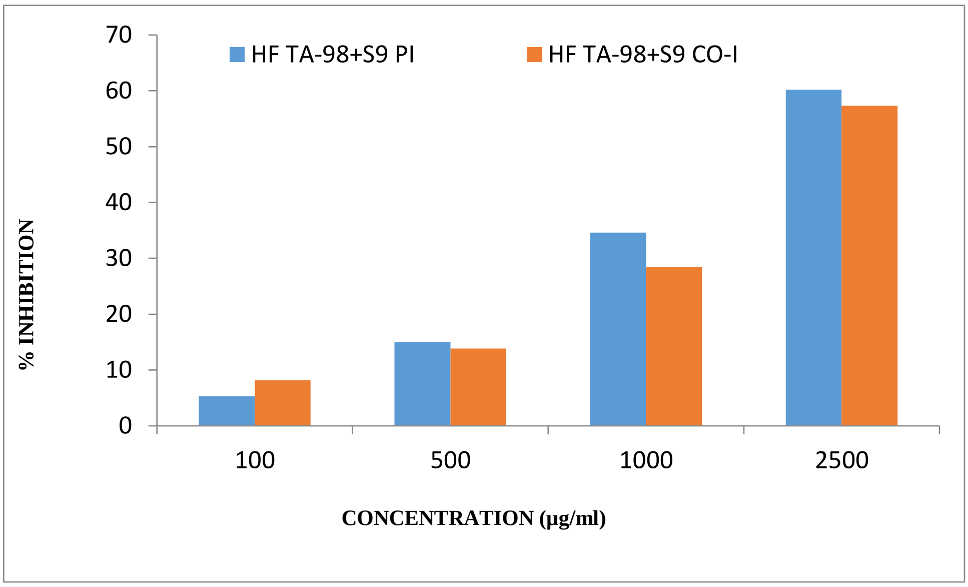
HF TA-98+S9 PI
HF TA-98+S9 CO-I
% INHIBITION
0
10
20
30
40
50
60
70
100
500
1000
2500
CONCENTRATION (µg/ml)

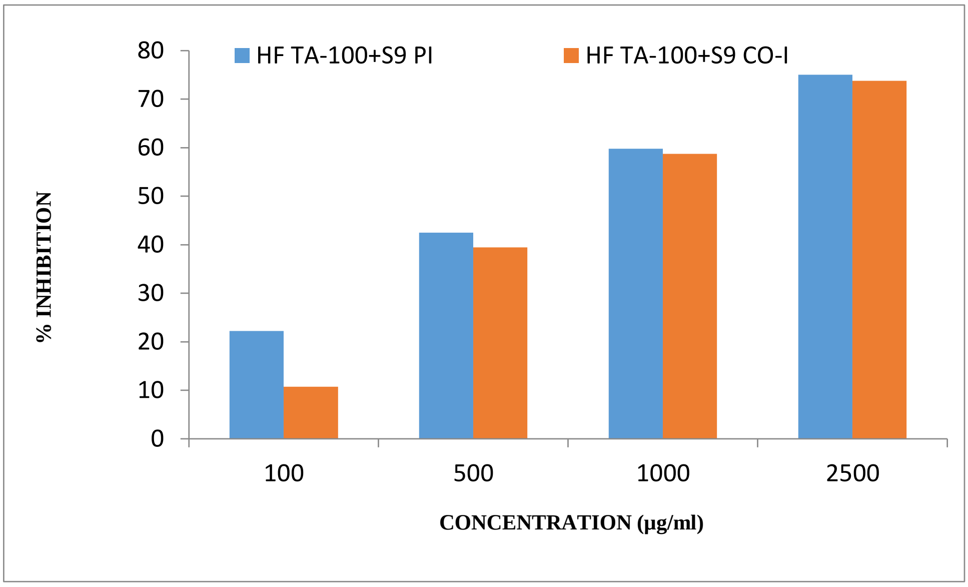
HF TA-100+S9 PI
HF TA-100+S9 CO-I
% INHIBITION
0
10
20
30
40
50
60
70
80
100
500
1000
2500
CONCENTRATION (µg/ml)

Ehyl acetate fraction:

Conc	100	500	1000	2500
EAF TA-98 PI	33.2328	54.50596	72.73315	83.48038
EAF TA-98 CO-I	25.50984	52.14347	69.13633	81.1563
EAF TA-100 PI	14.49321	36.41427	57.57896	74.46174
EAF TA-100 CO-I	11.0581	33.29808	51.25494	71.52325
EAF TA-98+S9 PI	6.286653	33.12231	58.22207	71.84231
EAF TA-98+S9 CO-I	4.171554	30.00391	55.94067	69.31694
EAF TA-100+S9 PI	15.32107	39.38817	57.58252	76.86947
EAF TA-100+S9 CO-I	7.273679	31.1893	56.65329	74.81563

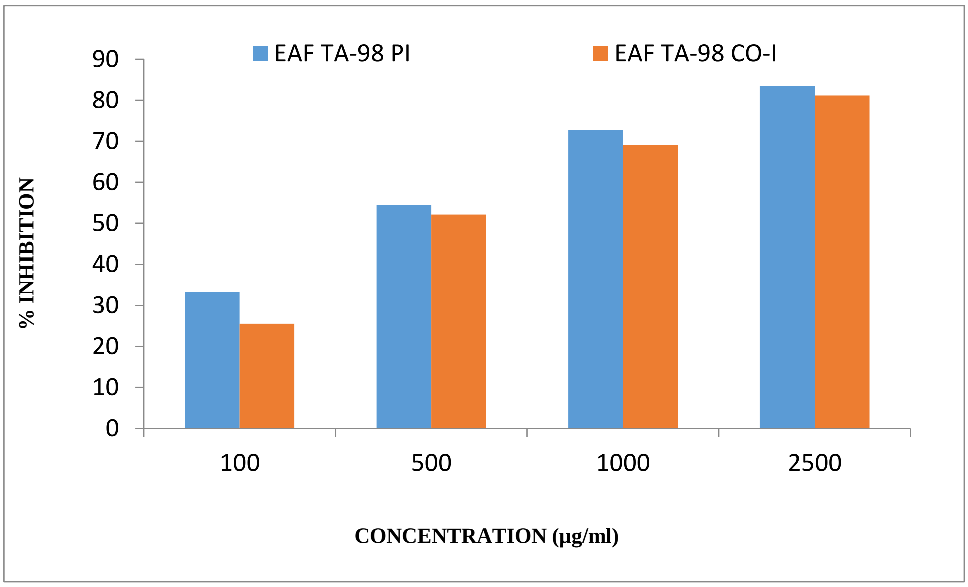

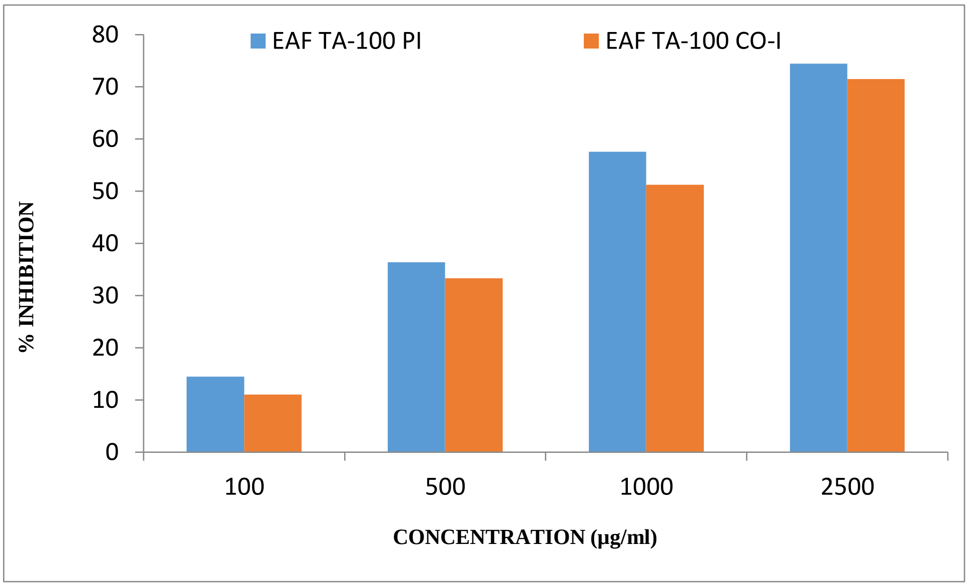

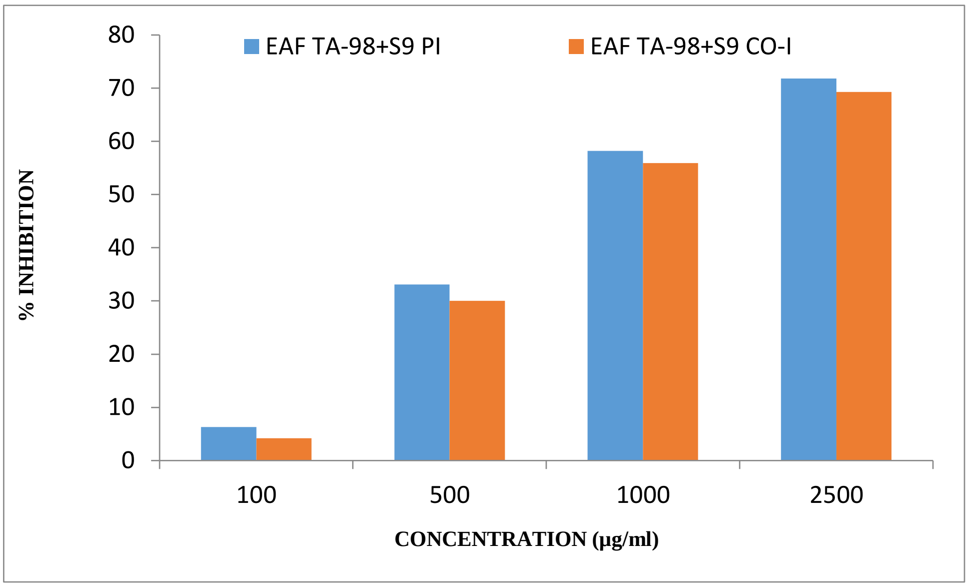

EAF TA-98+S9 PI
EAF TA-98+S9 CO-I
% INHIBITION
0
10
20
30
40
50
60
70
80
100
500
1000
2500
CONCENTRATION (µg/ml)

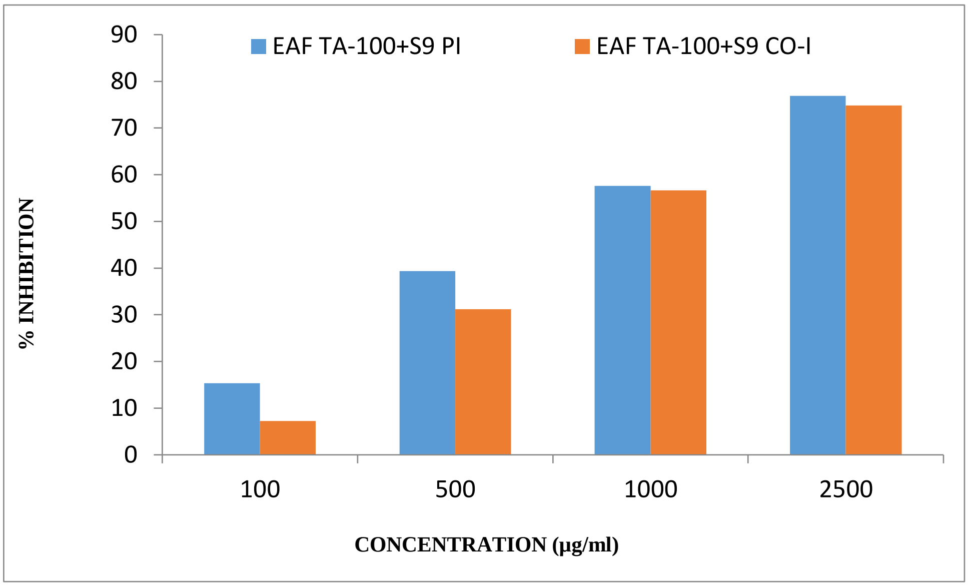

EAF TA-100+S9 PI
EAF TA-100+S9 CO-I
% INHIBITION
0
10
20
30
40
50
60
70
80
90
100
500
1000
2500
CONCENTRATION (µg/ml)

Supplementary file S-3
GCMS Report Chloroform Leaves

DEPTT. OF BOTANICAL & ENVIRONMENTAL SCIENCES, G.N.D.U. AMRITSAR

Sample Information

Analyzed by	: Admin
Analyzed	: 8/22/2015 12:13:17 PM
Sample Type	: Unknown
Sample Name	: ACIDS
Sample ID	: ANKET
Injection Volume	: 2
Data File	: E:\GCMS\GCMS DATA\Vandana\2.qgd
Method File	: E:\GCMS\GCMS DATA\anket\ANKET. B. JUNCEA PROFILING.qgm
Tuning File	: C:\GCMSsolution\System\Tune1\05-06-2014.qgt

Chromatogram ACIDS E:\GCMS\GCMS DATA\Vandana\2.qgd

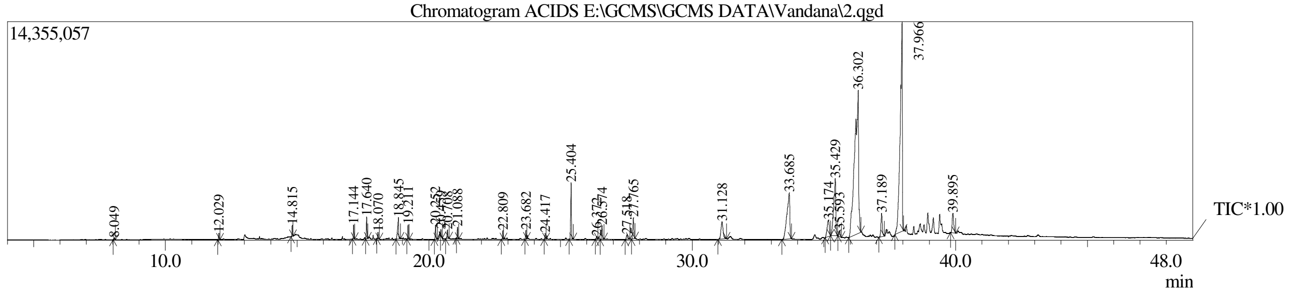

Spectrum

Line#:1 R.Time:8.1(Scan#:1216)
MassPeaks:116
RawMode:Averaged 8.0-8.1(1197-1233) BasePeak:55(7340)
BG Mode:None Group 1 - Event 1

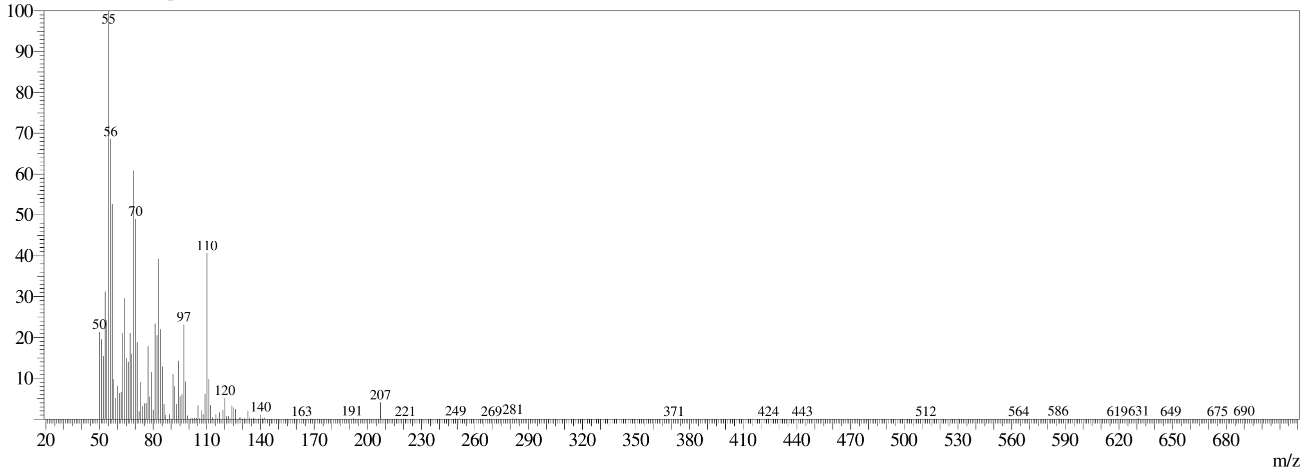

DEPTT. OF BOTANICAL & ENVIRONMENTAL SCIENCES, G.N.D.U. AMRITSAR

Line#:2 R.Time:12.0(Scan#:2410)
MassPeaks:144
RawMode:Averaged 12.0-12.1(2396-2425) BasePeak:55(18542)
BG Mode:None Group 1 - Event 1

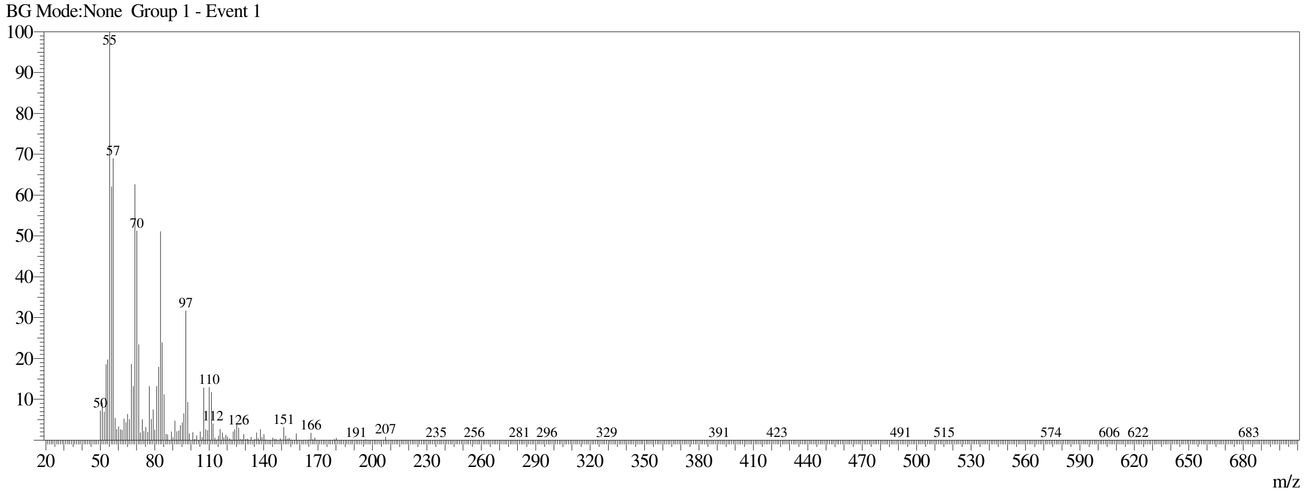

Line#:3 R.Time:14.8(Scan#:3246)
MassPeaks:158
RawMode:Averaged 14.8-14.9(3232-3264) BasePeak:57(41543)
BG Mode:None Group 1 - Event 1

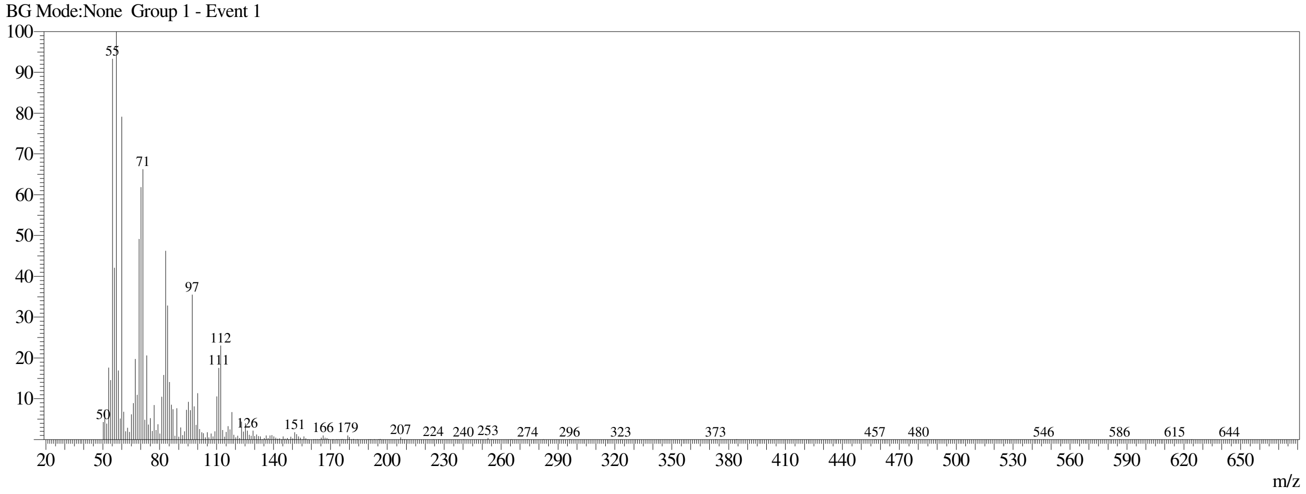

Line#:4 R.Time:17.1(Scan#:3944)
MassPeaks:178
RawMode:Averaged 17.1-17.2(3929-3963) BasePeak:55(28432)
BG Mode:None Group 1 - Event 1

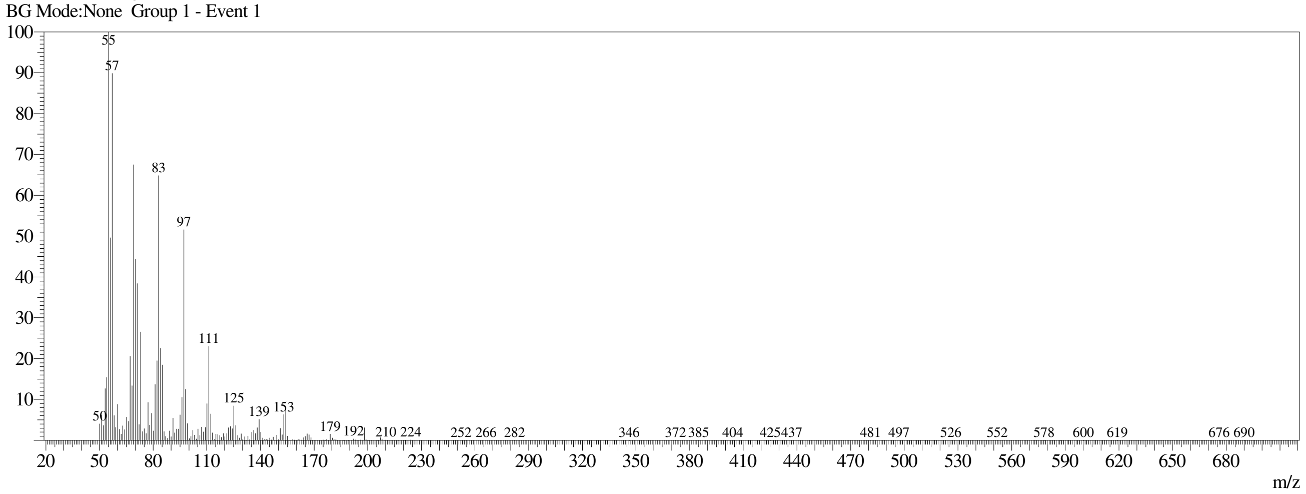

DEPTT. OF BOTANICAL & ENVIRONMENTAL SCIENCES, G.N.D.U. AMRITSAR

Line#:5 R.Time:17.6(Scan#:4093)
MassPeaks:163
RawMode:Averaged 17.6-17.7(4077-4108) BasePeak:68(45931)
BG Mode:None Group 1 - Event 1

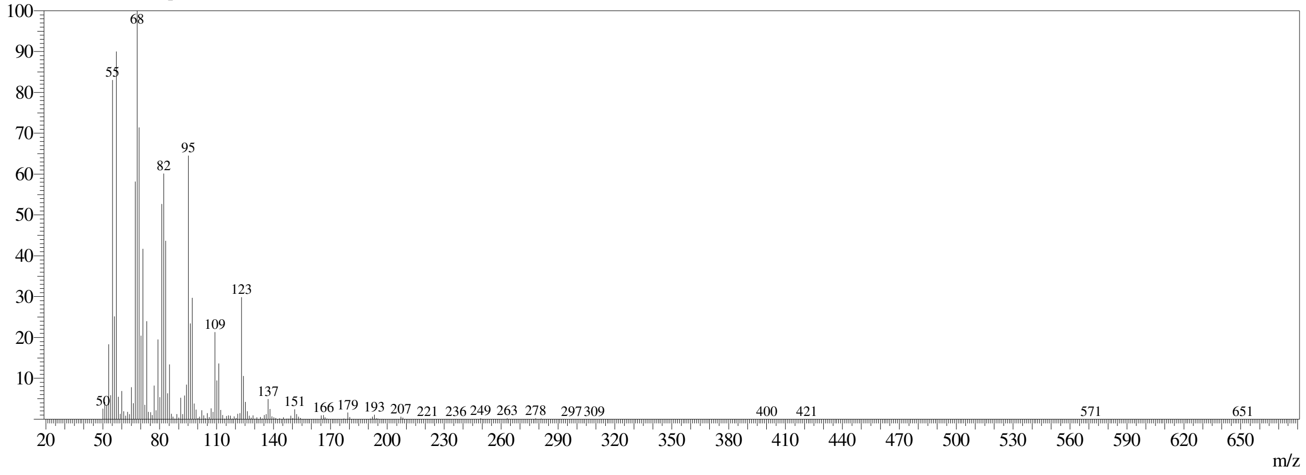

Line#:6 R.Time:18.1(Scan#:4222)
MassPeaks:189
RawMode:Averaged 18.0-18.1(4209-4245) BasePeak:57(14159)
BG Mode:None Group 1 - Event 1

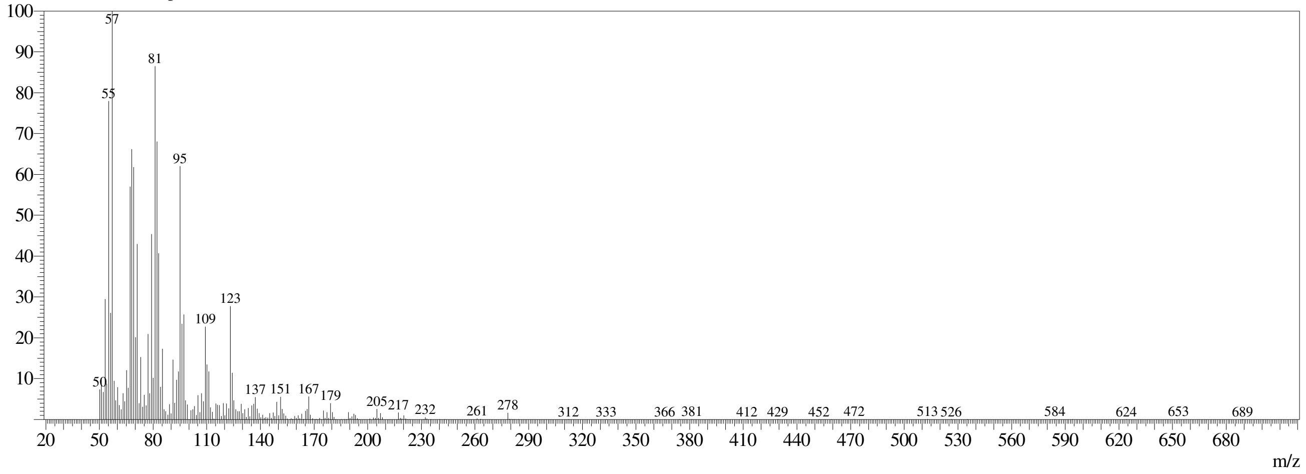

Line#:7 R.Time:18.8(Scan#:4455)
MassPeaks:199
RawMode:Averaged 18.7-18.9(4422-4474) BasePeak:73(43531)
BG Mode:None Group 1 - Event 1

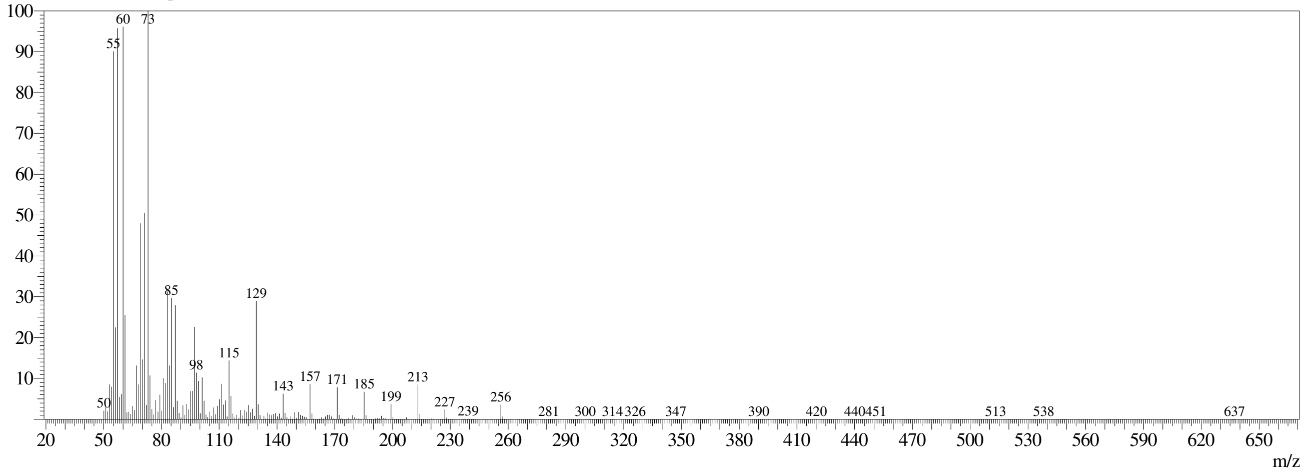

DEPTT. OF BOTANICAL & ENVIRONMENTAL SCIENCES, G.N.D.U. AMRITSAR

Line#:8 R.Time:19.2(Scan#:4564)
MassPeaks:165
RawMode:Averaged 19.2-19.3(4550-4577) BasePeak:57(34351)
BG Mode:None Group 1 - Event 1

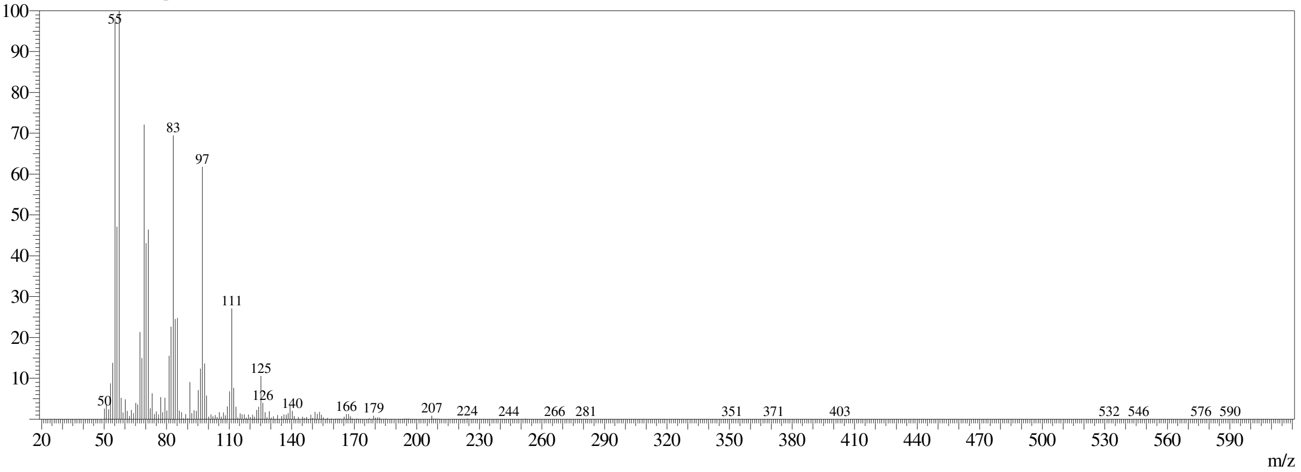

Line#:9 R.Time:20.3(Scan#:4877)
MassPeaks:173
RawMode:Averaged 20.2-20.3(4860-4898) BasePeak:71(37806)
BG Mode:None Group 1 - Event 1

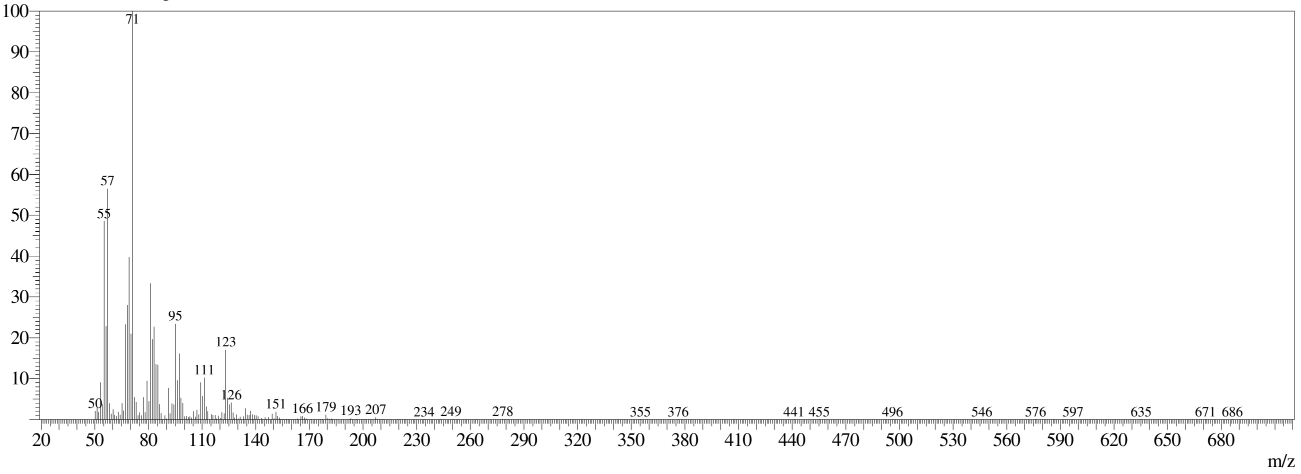

Line#:10 R.Time:20.5(Scan#:4939)
MassPeaks:195
RawMode:Averaged 20.4-20.5(4930-4959) BasePeak:55(32494)
BG Mode:None Group 1 - Event 1

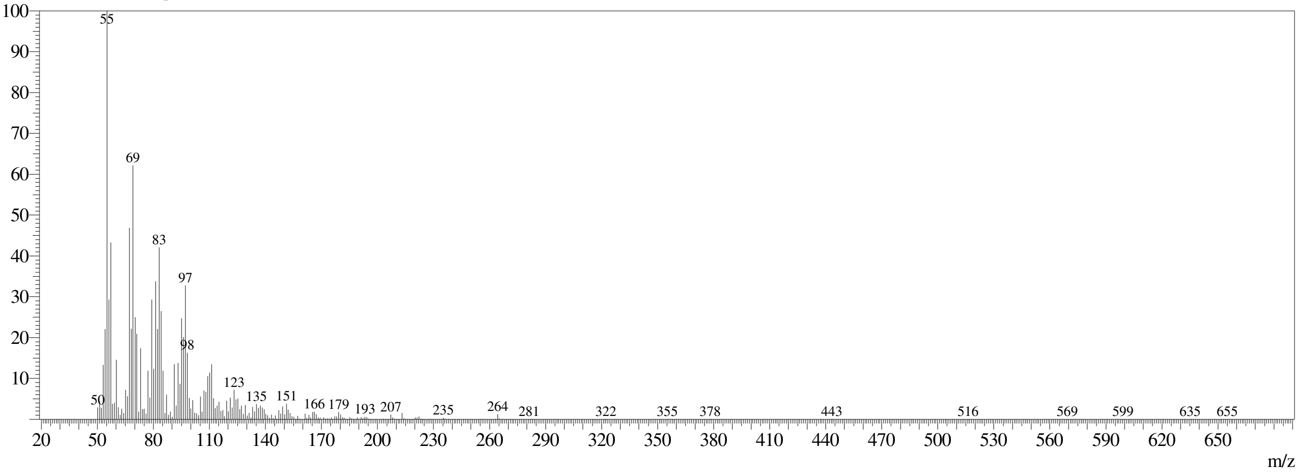

DEPTT. OF BOTANICAL & ENVIRONMENTAL SCIENCES, G.N.D.U. AMRITSAR

Line#:11 R.Time:20.7(Scan#:5013)
MassPeaks:189
RawMode:Averaged 20.6-20.8(4993-5035) BasePeak:57(21088)
BG Mode:None Group 1 - Event 1

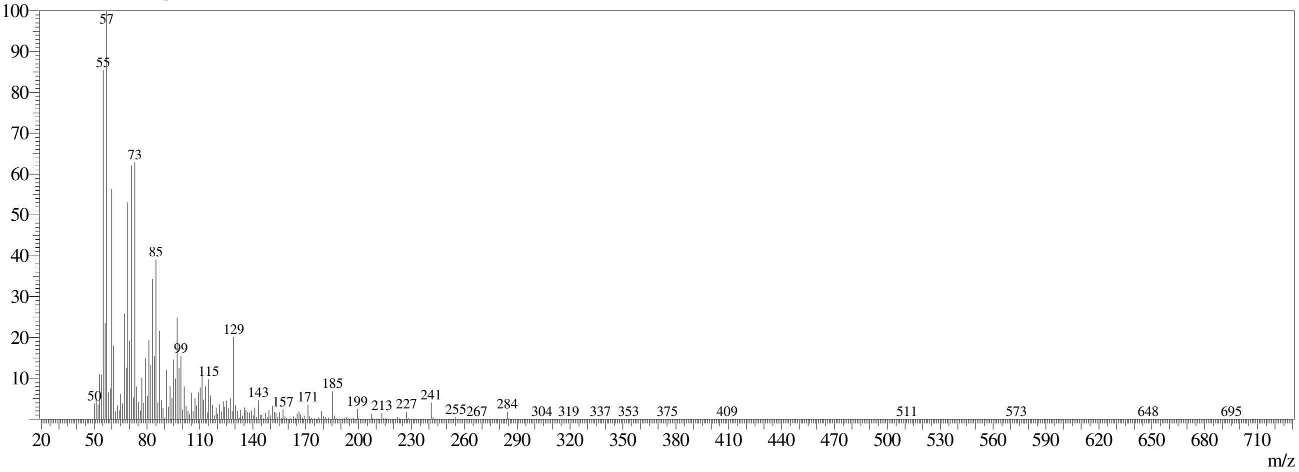

Line#:12 R.Time:21.1(Scan#:5127)
MassPeaks:179
RawMode:Averaged 21.1-21.2(5116-5147) BasePeak:57(28150)
BG Mode:None Group 1 - Event 1

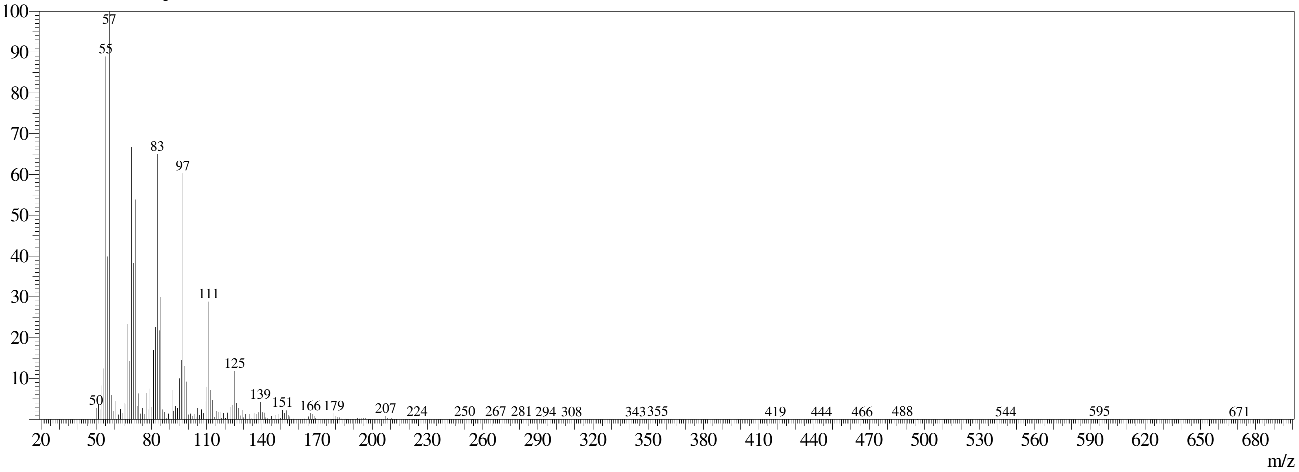

Line#:13 R.Time:22.8(Scan#:5644)
MassPeaks:166
RawMode:Averaged 22.8-22.8(5631-5653) BasePeak:57(26856)
BG Mode:None Group 1 - Event 1

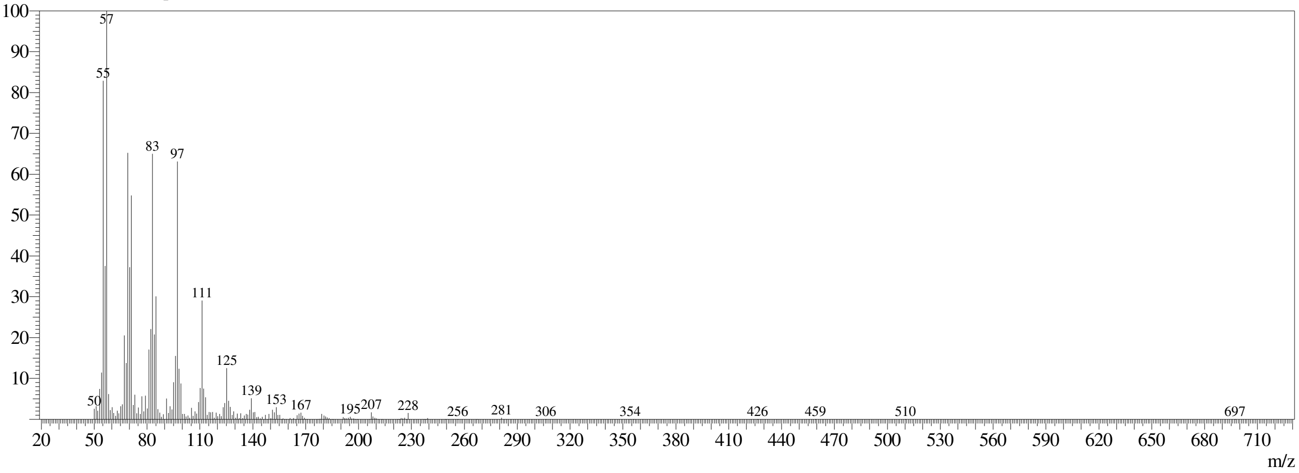

DEPTT. OF BOTANICAL & ENVIRONMENTAL SCIENCES, G.N.D.U. AMRITSAR

Line#:14 R.Time:23.7(Scan#:5906)
MassPeaks:171
RawMode:Averaged 23.6-23.7(5893-5915) BasePeak:57(50761)
BG Mode:None Group 1 - Event 1

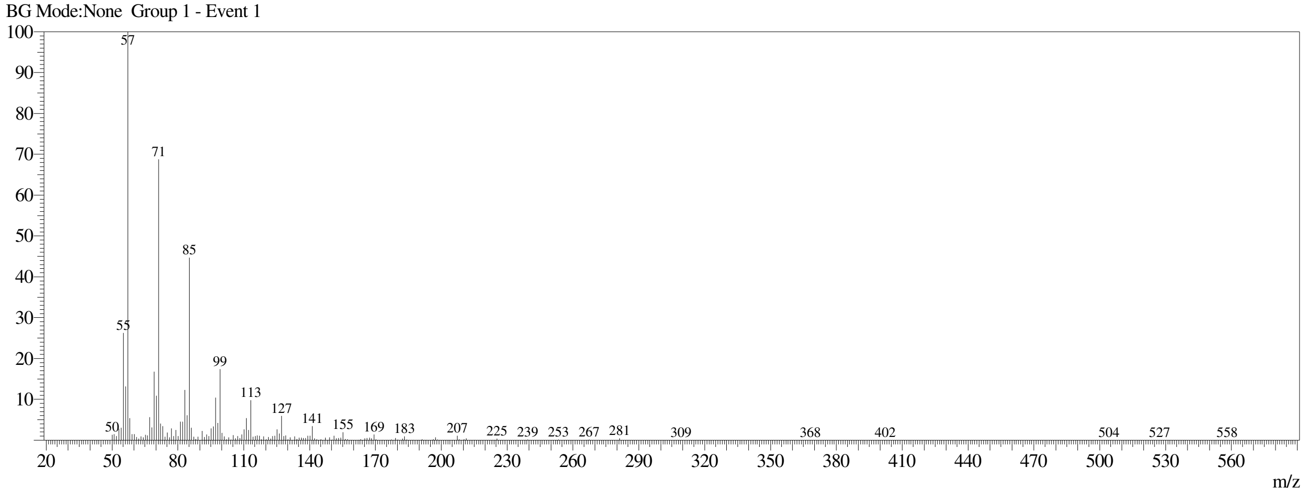

Line#:15 R.Time:24.4(Scan#:6126)
MassPeaks:202
RawMode:Averaged 24.4-24.5(6113-6146) BasePeak:57(24940)
BG Mode:None Group 1 - Event 1

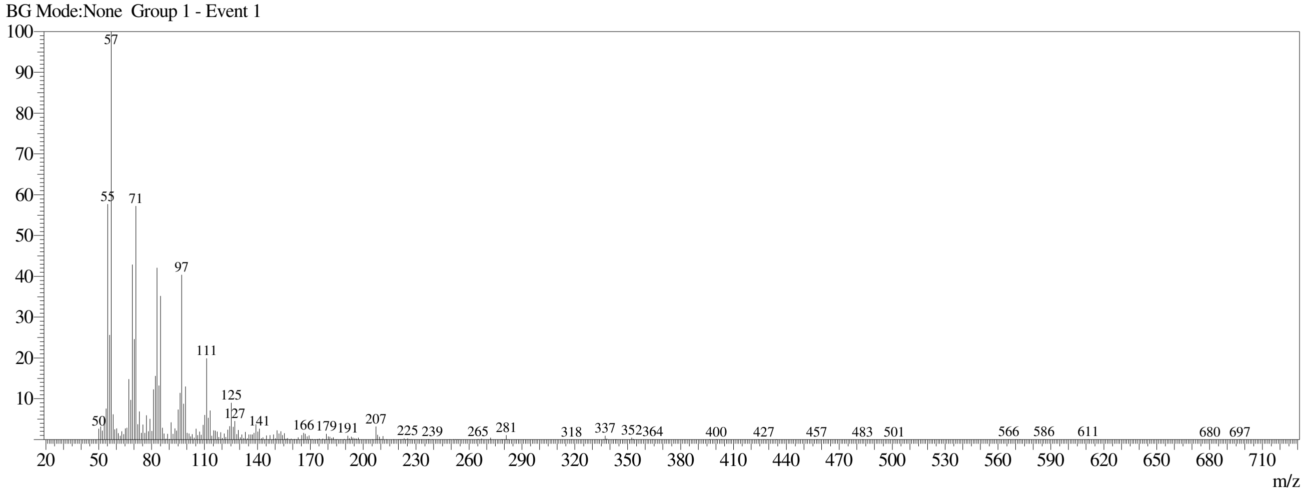

Line#:16 R.Time:25.4(Scan#:6422)
MassPeaks:247
RawMode:Averaged 25.3-25.5(6402-6449) BasePeak:57(195962)
BG Mode:None Group 1 - Event 1

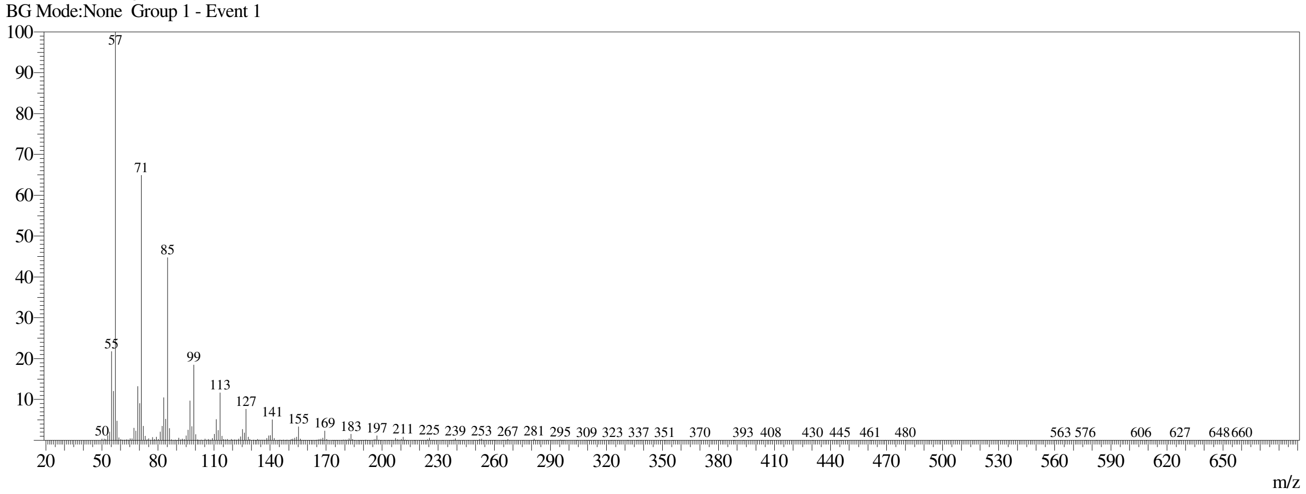

DEPTT. OF BOTANICAL & ENVIRONMENTAL SCIENCES, G.N.D.U. AMRITSAR

Line#:17 R.Time:26.4(Scan#:6713)
MassPeaks:187
RawMode:Averaged 26.3-26.4(6694-6728) BasePeak:57(12448)
BG Mode:None Group 1 - Event 1

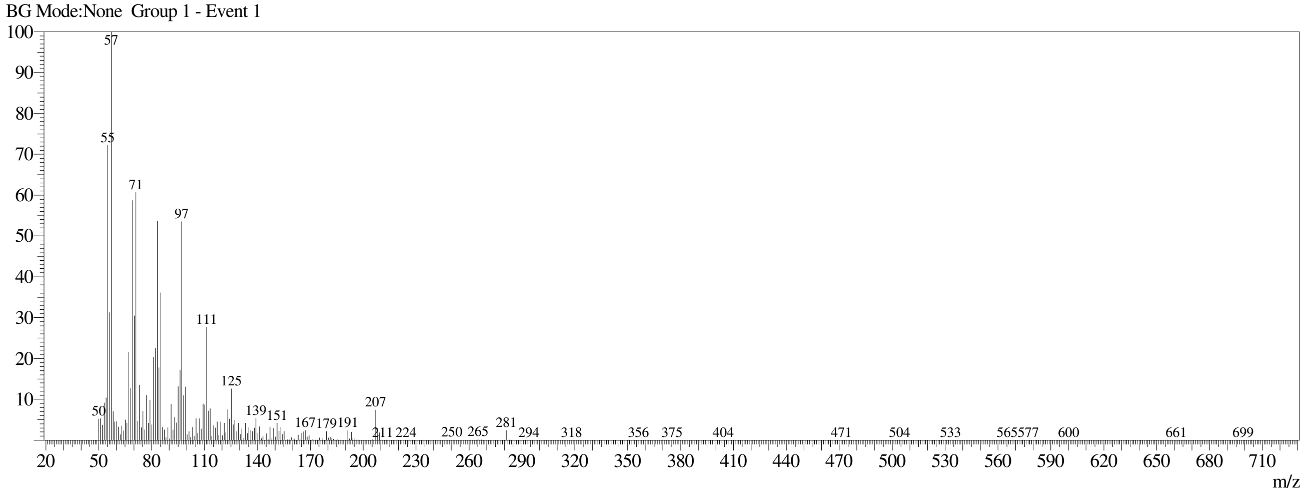

Line#:18 R.Time:26.6(Scan#:6773)
MassPeaks:211
RawMode:Averaged 26.5-26.6(6752-6793) BasePeak:69(74549)
BG Mode:None Group 1 - Event 1

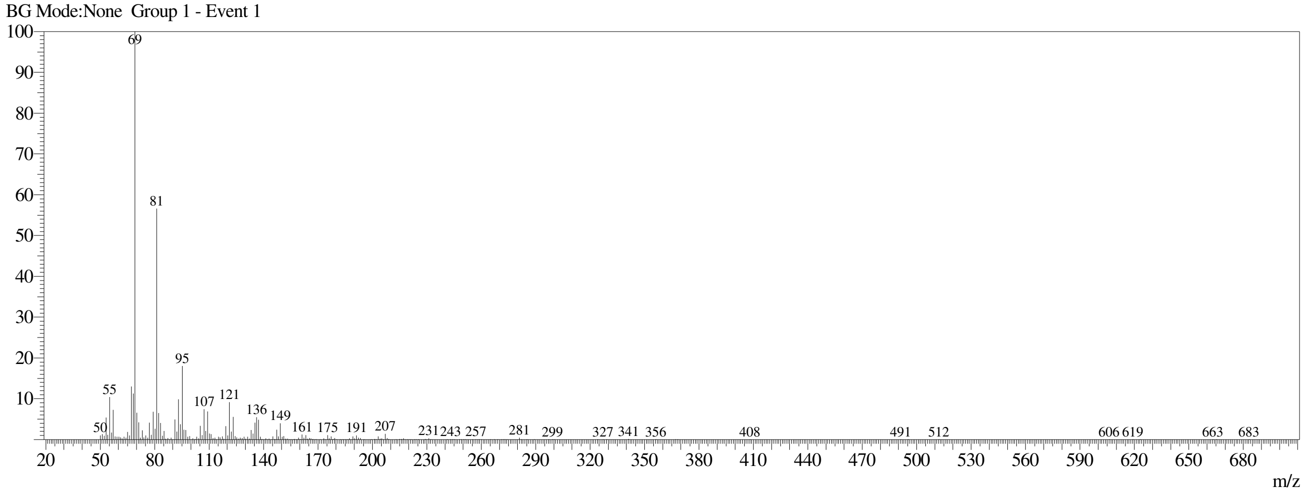

Line#:19 R.Time:27.5(Scan#:7056)
MassPeaks:194
RawMode:Averaged 27.4-27.6(7035-7086) BasePeak:57(15378)
BG Mode:None Group 1 - Event 1

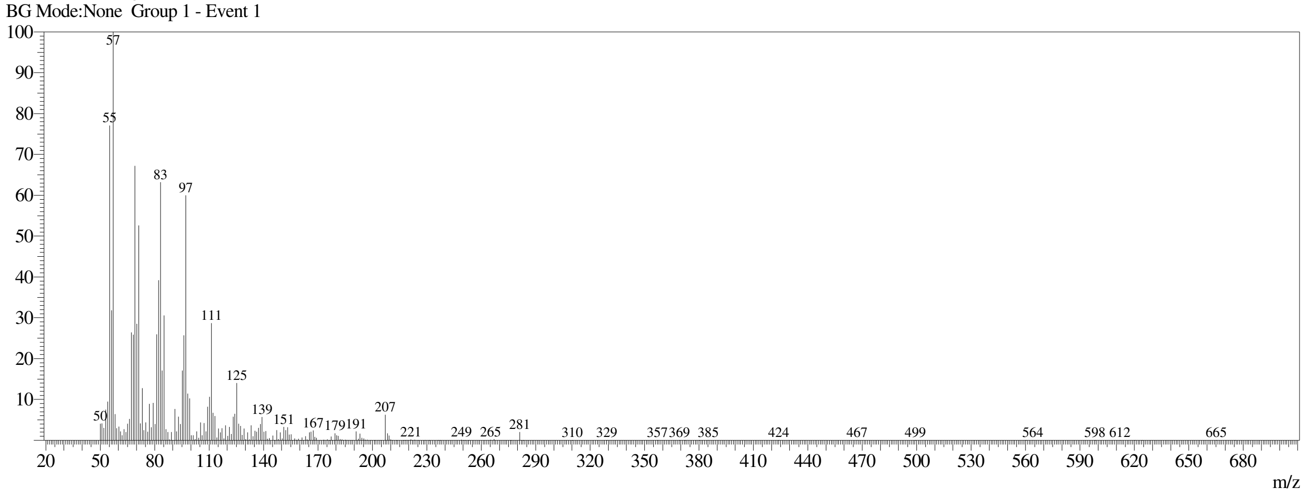

DEPTT. OF BOTANICAL & ENVIRONMENTAL SCIENCES, G.N.D.U. AMRITSAR

Line#:20 R.Time:27.8(Scan#:7131)
MassPeaks:216
RawMode:Averaged 27.7-27.8(7106-7149) BasePeak:57(108072)
BG Mode:None Group 1 - Event 1

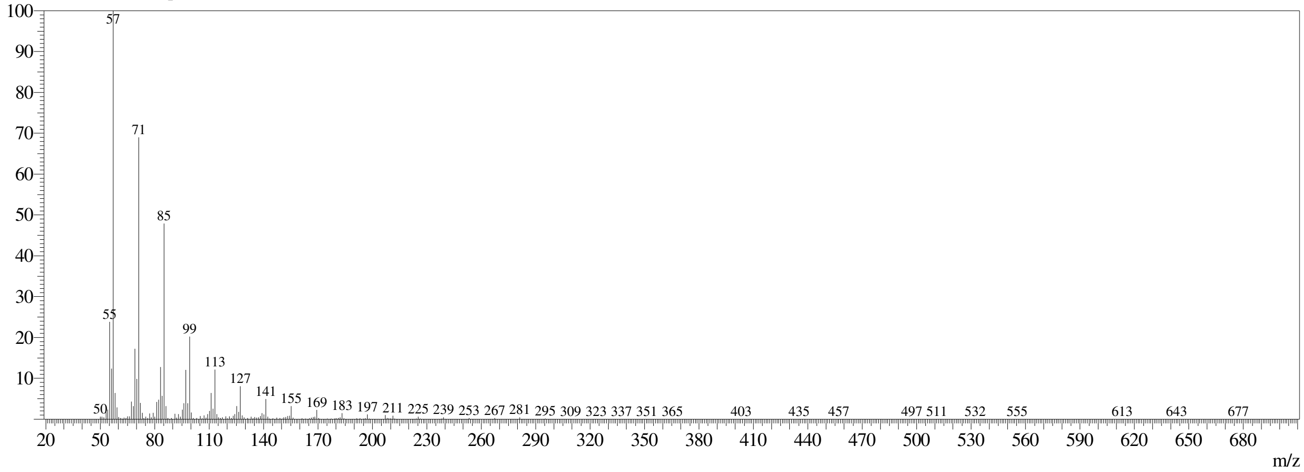

Line#:21 R.Time:31.1(Scan#:8139)
MassPeaks:279
RawMode:Averaged 31.0-31.3(8099-8188) BasePeak:57(39615)
BG Mode:None Group 1 - Event 1

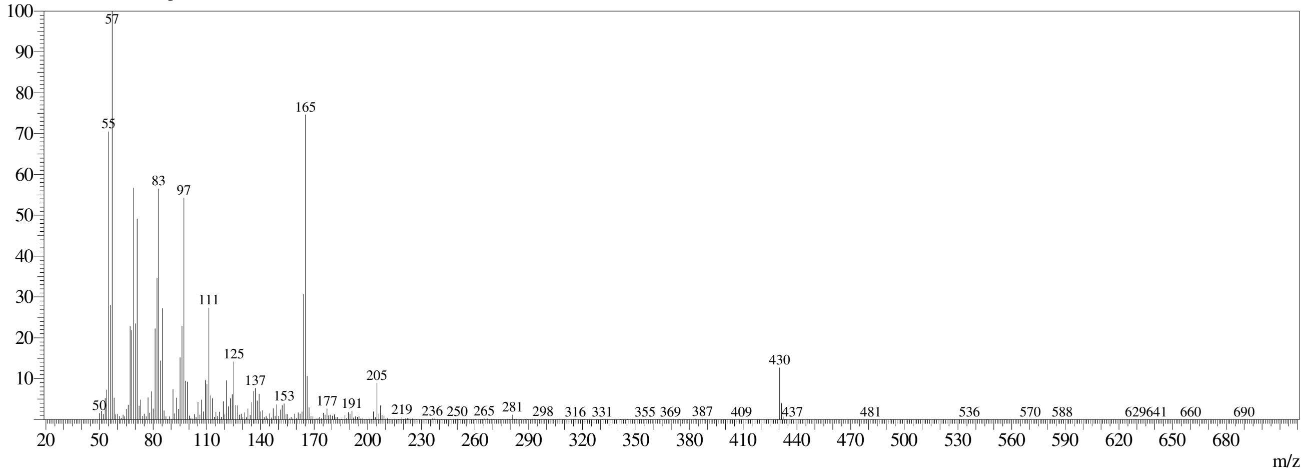

Line#:22 R.Time:33.7(Scan#:8907)
MassPeaks:367
RawMode:Averaged 33.4-33.8(8818-8946) BasePeak:137(79578)
BG Mode:None Group 1 - Event 1

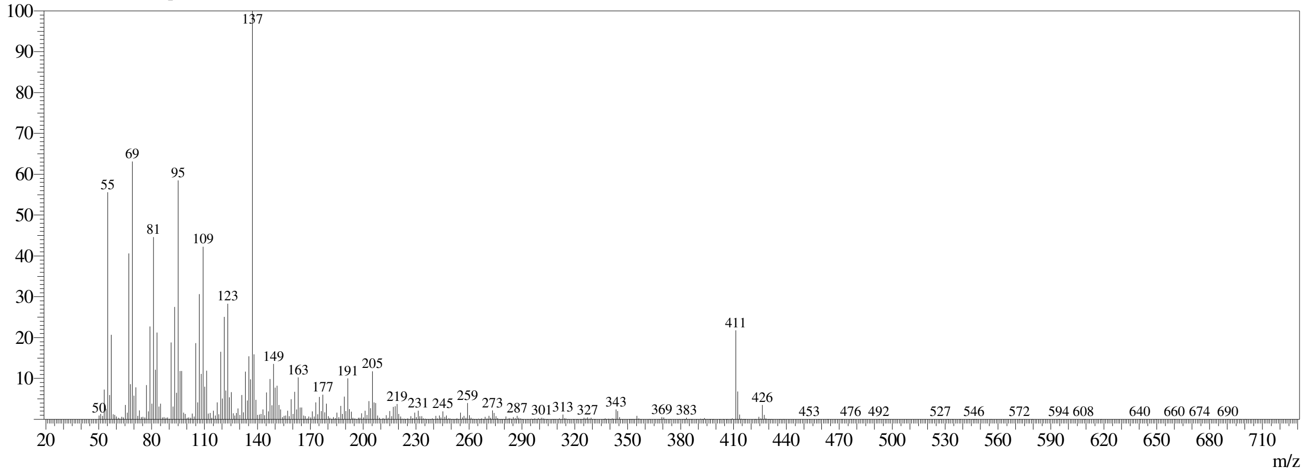

DEPTT. OF BOTANICAL & ENVIRONMENTAL SCIENCES, G.N.D.U. AMRITSAR

Line#:23 R.Time:35.2(Scan#:9353)
MassPeaks:343
RawMode:Averaged 35.0-35.3(9301-9379) BasePeak:55(23290)
BG Mode:None Group 1 - Event 1

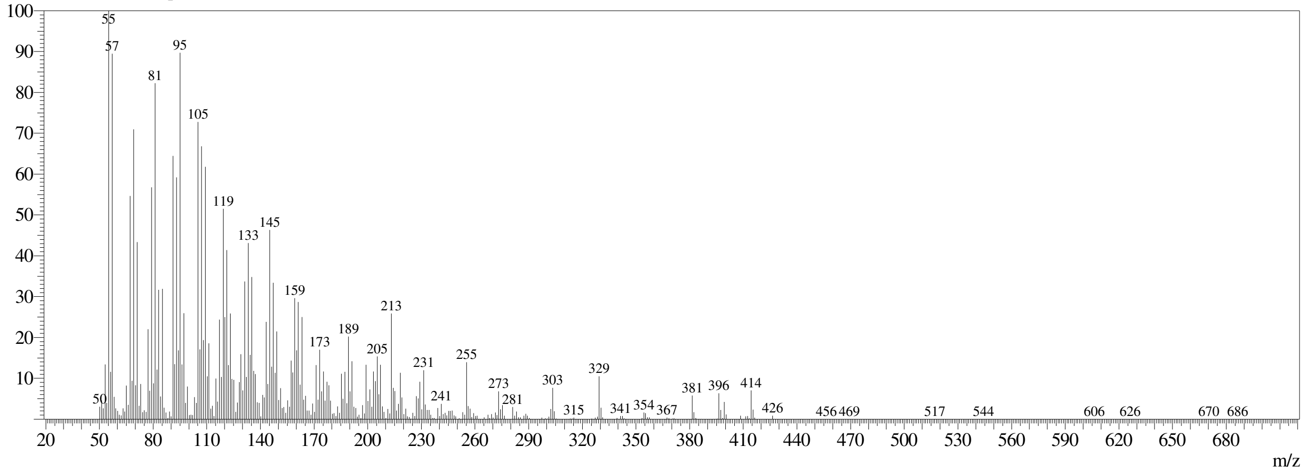

Line#:24 R.Time:35.4(Scan#:9430)
MassPeaks:354
RawMode:Averaged 35.3-35.5(9379-9458) BasePeak:218(135255)
BG Mode:None Group 1 - Event 1

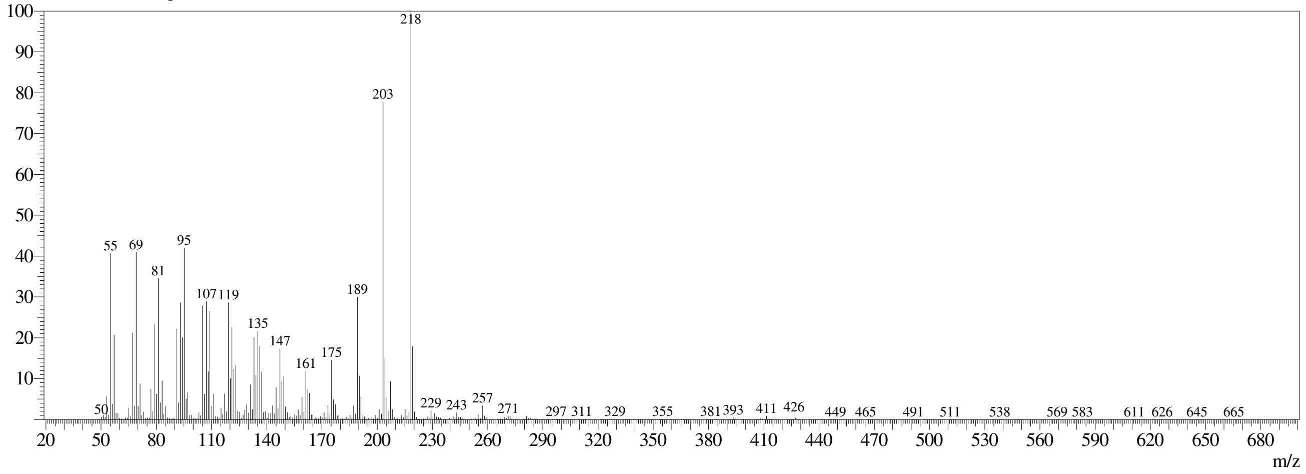

Line#:25 R.Time:35.6(Scan#:9479)
MassPeaks:273
RawMode:Averaged 35.5-35.7(9458-9507) BasePeak:55(14134)
BG Mode:None Group 1 - Event 1

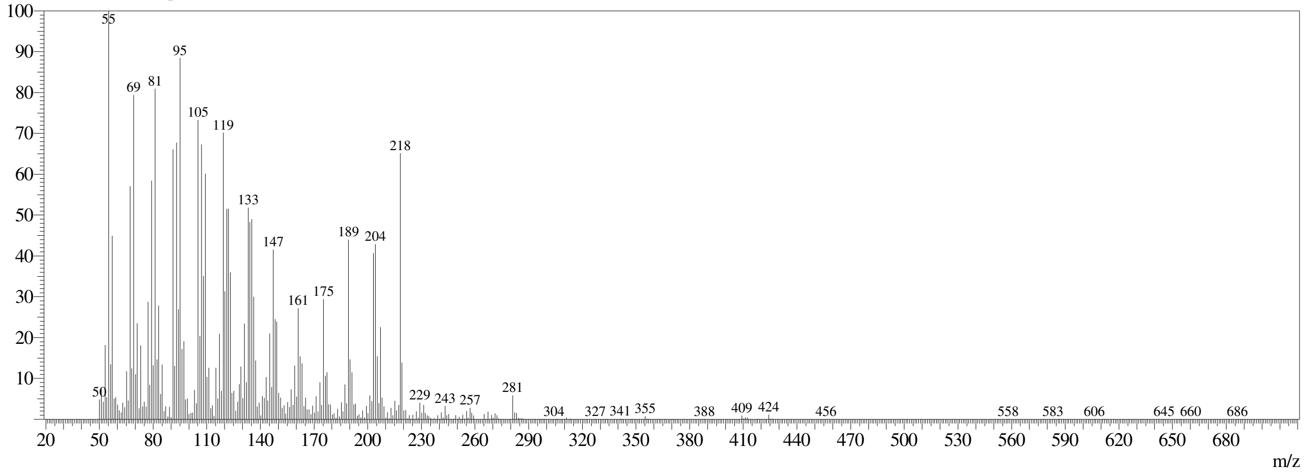

DEPTT. OF BOTANICAL & ENVIRONMENTAL SCIENCES, G.N.D.U. AMRITSAR

Line#:26 R.Time:36.3(Scan#:9692)
MassPeaks:423
RawMode:Averaged 35.9-36.4(9585-9726) BasePeak:218(184365)
BG Mode:None Group 1 - Event 1

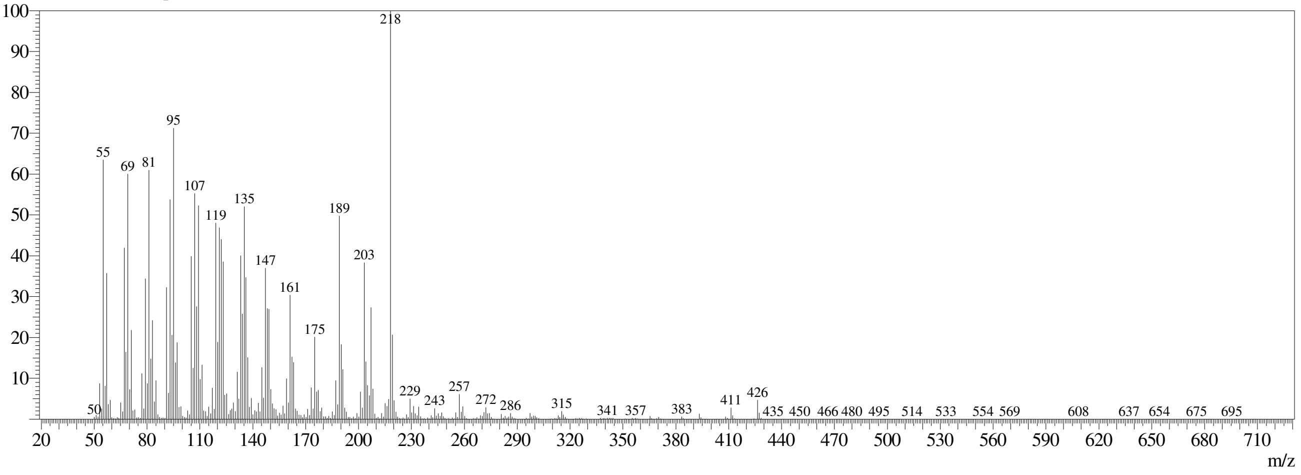

Line#:27 R.Time:37.2(Scan#:9958)
MassPeaks:347
RawMode:Averaged 37.1-37.3(9928-9989) BasePeak:69(31129)
BG Mode:None Group 1 - Event 1

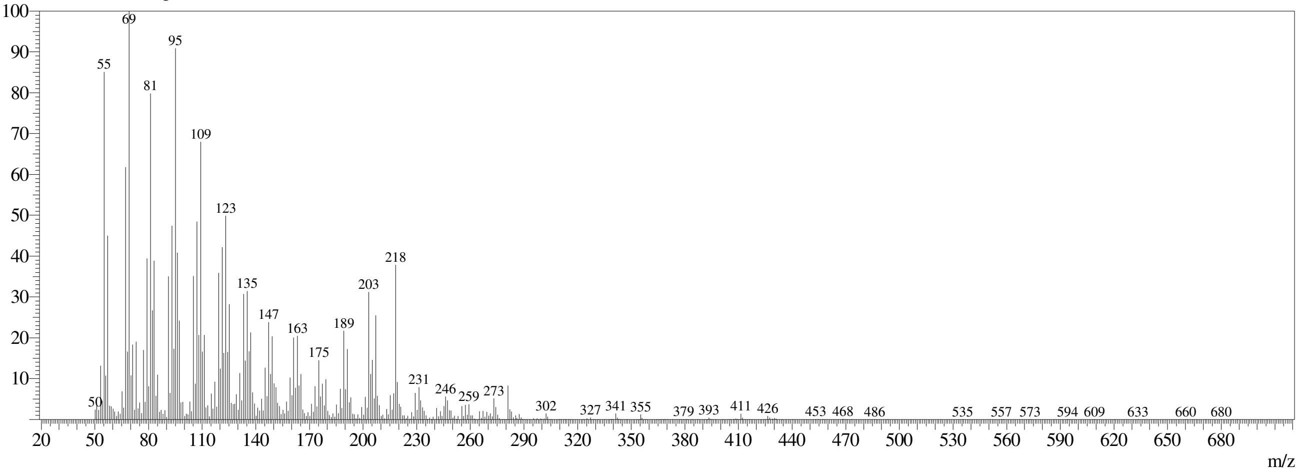

Line#:28 R.Time:38.0(Scan#:10191)
MassPeaks:419
RawMode:Averaged 37.7-38.0(10099-10207) BasePeak:69(202219)
BG Mode:None Group 1 - Event 1

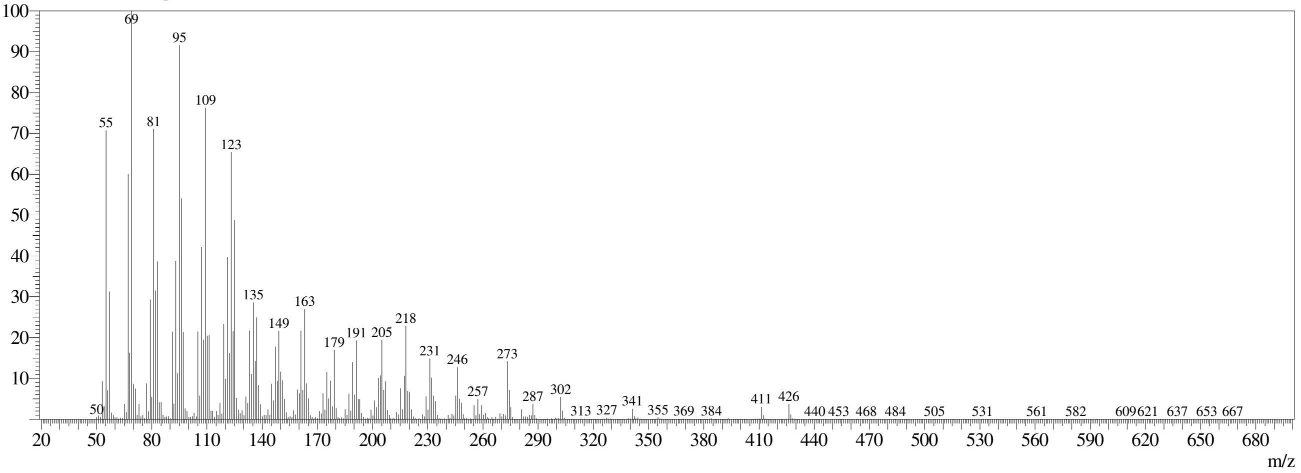

DEPTT. OF BOTANICAL & ENVIRONMENTAL SCIENCES,
G.N.D.U.
AMRITSAR

Line#:29 R.Time:39.9(Scan#:10769)
MassPeaks:399
RawMode:Averaged 39.8-40.0(10735-10805) BasePeak:203(53655)
BG Mode:None Group 1 - Event 1

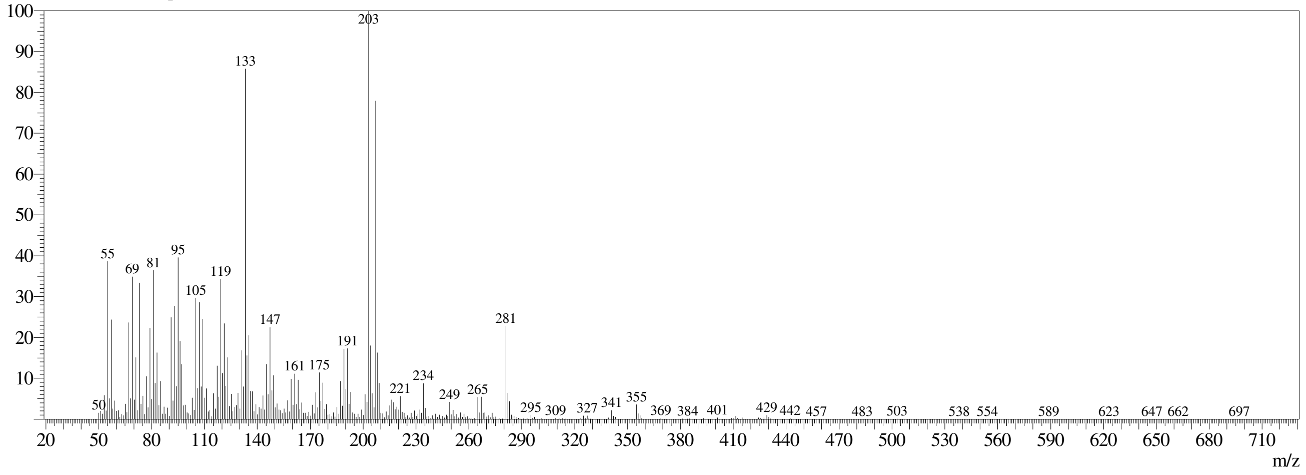

Mass Table
Line#:1 R.Time:8.1(Scan#:1216)
MassPeaks:116
RawMode:Averaged 8.0-8.1(1197-1233) BasePeak:55(7340)
BG Mode:None Group 1 - Event 1

#	m/z	Abs. Int.	Rel. Int.	#	m/z	Abs. Int.	Rel. Int.	#	m/z	Abs. Int.	Rel. Int.
1	50.05	1565	21.32	40	90.10	5	0.07	79	134.10	28	0.38
2	51.10	1436	19.56	41	91.10	812	11.06	80	135.20	12	0.16
3	52.10	1133	15.44	42	92.10	591	8.05	81	136.10	17	0.23
4	53.15	2293	31.24	43	93.10	272	3.71	82	137.10	5	0.07
5	54.15	1778	24.22	44	94.15	1046	14.25	83	139.10	10	0.14
6	55.15	7340	100.00	45	95.10	411	5.60	84	140.20	81	1.10
7	56.15	5030	68.53	46	96.15	444	6.05	85	141.10	12	0.16
8	57.15	3865	52.66	47	97.15	1697	23.12	86	142.10	24	0.33
9	58.10	722	9.84	48	98.20	672	9.16	87	163.00	6	0.08
10	59.10	378	5.15	49	99.20	65	0.89	88	166.10	6	0.08
11	60.10	597	8.13	50	101.10	19	0.26	89	168.20	13	0.18
12	61.10	466	6.35	51	102.10	12	0.16	90	172.10	12	0.16
13	62.10	482	6.57	52	103.10	25	0.34	91	176.10	5	0.07
14	63.10	1550	21.12	53	104.20	17	0.23	92	191.10	12	0.16
15	64.10	2176	29.65	54	105.10	249	3.39	93	192.10	11	0.15
16	65.10	1094	14.90	55	106.20	25	0.34	94	207.10	297	4.05
17	66.05	1033	14.07	56	107.20	155	2.11	95	209.10	6	0.08
18	67.15	1549	21.10	57	108.00	85	1.16	96	221.10	5	0.07
19	68.10	1173	15.98	58	109.10	456	6.21	97	249.10	11	0.15
20	69.15	4467	60.86	59	110.10	2981	40.61	98	251.10	11	0.15
21	70.15	3598	49.02	60	111.15	722	9.84	99	269.20	5	0.07
22	71.15	1382	18.83	61	112.10	255	3.47	100	281.20	42	0.57
23	72.10	136	1.85	62	113.10	26	0.35	101	283.10	12	0.16
24	73.10	662	9.02	63	115.10	87	1.19	102	371.20	5	0.07
25	74.10	235	3.20	64	116.10	11	0.15	103	424.10	5	0.07
26	75.20	280	3.81	65	117.10	123	1.68	104	443.10	6	0.08
27	76.10	287	3.91	66	119.10	168	2.29	105	454.10	5	0.07
28	77.10	1308	17.82	67	120.10	379	5.16	106	512.10	5	0.07
29	78.10	405	5.52	68	121.10	54	0.74	107	564.10	6	0.08
30	79.10	845	11.51	69	122.10	54	0.74	108	586.10	13	0.18
31	80.10	166	2.26	70	123.10	5	0.07	109	619.10	6	0.08
32	81.10	1714	23.35	71	124.10	240	3.27	110	621.10	6	0.08
33	82.15	1508	20.54	72	125.10	206	2.81	111	630.20	5	0.07
34	83.10	2887	39.33	73	126.10	181	2.47	112	631.10	12	0.16
35	84.10	1611	21.95	74	128.10	23	0.31	113	649.00	6	0.08
36	85.15	944	12.86	75	129.10	30	0.41	114	675.10	5	0.07
37	86.10	272	3.71	76	130.10	5	0.07	115	688.10	5	0.07
38	87.10	76	1.04	77	131.00	12	0.16	116	690.10	11	0.15
39	89.10	85	1.16	78	133.10	150	2.04				

Line#:2 R.Time:12.0(Scan#:2410)
MassPeaks:144
RawMode:Averaged 12.0-12.1(2396-2425) BasePeak:55(18542)
BG Mode:None Group 1 - Event 1

#	m/z	Abs. Int.	Rel. Int.	#	m/z	Abs. Int.	Rel. Int.	#	m/z	Abs. Int.	Rel. Int.
1	50.10	1331	7.18	3	52.10	1298	7.00	5	54.15	3664	19.76
2	51.15	1698	9.16	4	53.15	3452	18.62	6	55.15	18542	100.00

DEPTT. OF BOTANICAL & ENVIRONMENTAL SCIENCES, G.N.D.U. AMRITSAR

#	m/z	Abs. Int.	Rel. Int.
7	56.15	11515	62.10
8	57.15	12796	69.01
9	58.15	1025	5.53
10	59.10	503	2.71
11	60.10	632	3.41
12	61.10	497	2.68
13	62.10	471	2.54
14	63.05	981	5.29
15	64.10	815	4.40
16	65.10	1187	6.40
17	66.10	955	5.15
18	67.10	3459	18.65
19	68.15	2452	13.22
20	69.15	11626	62.70
21	70.15	9511	51.29
22	71.15	4344	23.43
23	72.10	356	1.92
24	73.15	949	5.12
25	74.10	416	2.24
26	75.10	595	3.21
27	76.10	383	2.07
28	77.10	2461	13.27
29	78.10	960	5.18
30	79.10	1395	7.52
31	80.10	482	2.60
32	81.10	2453	13.23
33	82.15	3333	17.98
34	83.15	9479	51.12
35	84.15	4435	23.92
36	85.15	2082	11.23
37	86.20	291	1.57
38	87.10	257	1.39
39	89.20	388	2.09
40	90.10	133	0.72
41	91.10	887	4.78
42	92.20	410	2.21
43	93.20	438	2.36
44	94.10	684	3.69
45	95.15	814	4.39
46	96.15	1221	6.59
47	97.15	5897	31.80
48	98.20	1730	9.33
49	99.15	309	1.67
50	100.10	13	0.07
51	101.10	358	1.93
52	102.10	58	0.31

#	m/z	Abs. Int.	Rel. Int.
53	103.10	212	1.14
54	105.10	393	2.12
55	106.10	143	0.77
56	107.10	2383	12.85
57	108.10	490	2.64
58	109.10	456	2.46
59	110.10	2414	13.02
60	111.20	2180	11.76
61	112.20	761	4.10
62	113.20	120	0.65
63	114.20	29	0.16
64	115.20	209	1.13
65	116.10	513	2.77
66	117.20	349	1.88
67	118.10	131	0.71
68	119.20	251	1.35
69	120.10	170	0.92
70	121.10	91	0.49
71	122.10	43	0.23
72	123.20	392	2.11
73	124.20	495	2.67
74	125.20	740	3.99
75	126.20	560	3.02
76	127.20	56	0.30
77	128.20	47	0.25
78	129.10	280	1.51
79	130.20	61	0.33
80	131.20	67	0.36
81	132.10	14	0.08
82	133.10	140	0.76
83	135.10	55	0.30
84	136.10	356	1.92
85	137.10	83	0.45
86	138.20	494	2.66
87	139.20	146	0.79
88	140.20	272	1.47
89	141.20	24	0.13
90	142.10	17	0.09
91	143.10	14	0.08
92	145.20	120	0.65
93	146.20	62	0.33
94	147.10	37	0.20
95	148.20	7	0.04
96	149.20	82	0.44
97	150.20	35	0.19
98	151.10	599	3.23

#	m/z	Abs. Int.	Rel. Int.
99	152.20	197	1.06
100	153.20	59	0.32
101	154.20	98	0.53
102	155.20	22	0.12
103	156.20	13	0.07
104	157.10	13	0.07
105	158.10	302	1.63
106	159.10	13	0.07
107	160.10	6	0.03
108	165.10	32	0.17
109	166.20	341	1.84
110	167.10	44	0.24
111	168.20	116	0.63
112	169.20	16	0.09
113	170.20	7	0.04
114	172.10	7	0.04
115	177.10	6	0.03
116	179.10	46	0.25
117	180.10	106	0.57
118	183.20	9	0.05
119	191.10	14	0.08
120	196.20	22	0.12
121	197.20	15	0.08
122	201.10	6	0.03
123	207.20	164	0.88
124	217.10	7	0.04
125	229.10	6	0.03
126	235.10	7	0.04
127	237.20	7	0.04
128	256.20	7	0.04
129	262.10	6	0.03
130	264.10	7	0.04
131	267.10	6	0.03
132	277.10	6	0.03
133	281.10	7	0.04
134	296.10	6	0.03
135	322.10	6	0.03
136	329.10	8	0.04
137	391.10	14	0.08
138	423.10	6	0.03
139	491.10	7	0.04
140	515.10	7	0.04
141	574.10	7	0.04
142	606.20	6	0.03
143	622.10	7	0.04
144	683.20	7	0.04

Line#:3 R.Time:14.8(Scan#:3246)
MassPeaks:158
RawMode:Averaged 14.8-14.9(3232-3264) BasePeak:57(41543)
BG Mode:None Group 1 - Event 1

#	m/z	Abs. Int.	Rel. Int.
1	50.15	1779	4.28
2	51.10	3021	7.27
3	52.10	1627	3.92
4	53.15	7317	17.61
5	54.15	6070	14.61
6	55.15	38766	93.32
7	56.15	17485	42.09
8	57.15	41543	100.00
9	58.10	7022	16.90
10	59.15	2149	5.17
11	60.10	32883	79.15
12	61.10	2849	6.86
13	62.10	839	2.02
14	63.10	1217	2.93
15	64.10	796	1.92
16	65.10	2579	6.21
17	66.10	3712	8.94
18	67.10	8212	19.77
19	68.10	4576	11.02
20	69.15	20434	49.19
21	70.15	25717	61.90
22	71.15	27527	66.26
23	72.10	2007	4.83
24	73.10	8571	20.63
25	74.10	1542	3.71
26	75.10	2172	5.23
27	76.10	878	2.11
28	77.10	3513	8.46

#	m/z	Abs. Int.	Rel. Int.
29	78.10	985	2.37
30	79.10	1570	3.78
31	80.10	624	1.50
32	81.15	4364	10.50
33	82.10	6585	15.85
34	83.15	19213	46.25
35	84.15	13642	32.84
36	85.15	5859	14.10
37	86.10	3561	8.57
38	87.10	3104	7.47
39	88.10	406	0.98
40	89.10	3189	7.68
41	90.10	291	0.70
42	91.10	1243	2.99
43	92.10	443	1.07
44	93.10	856	2.06
45	94.10	3039	7.32
46	95.10	3852	9.27
47	96.15	2992	7.20
48	97.15	14771	35.56
49	98.15	3403	8.19
50	99.15	1508	3.63
51	100.10	4713	11.34
52	101.05	1090	2.62
53	102.15	750	1.81
54	103.10	653	1.57
55	104.00	233	0.56
56	105.10	765	1.84

#	m/z	Abs. Int.	Rel. Int.
57	106.00	236	0.57
58	107.10	627	1.51
59	108.10	317	0.76
60	109.15	846	2.04
61	110.15	4414	10.63
62	111.15	7308	17.59
63	112.15	9592	23.09
64	113.20	983	2.37
65	114.20	291	0.70
66	115.10	777	1.87
67	116.10	1358	3.27
68	117.10	1053	2.53
69	118.10	2809	6.76
70	119.10	505	1.22
71	120.10	229	0.55
72	121.10	382	0.92
73	122.10	172	0.41
74	123.10	1822	4.39
75	124.20	817	1.97
76	125.20	1646	3.96
77	126.20	917	2.21
78	127.20	502	1.21
79	128.20	406	0.98
80	129.15	901	2.17
81	130.10	359	0.86
82	131.10	512	1.23
83	132.10	354	0.85
84	133.10	330	0.79

DEPTT. OF BOTANICAL & ENVIRONMENTAL SCIENCES, G.N.D.U. AMRITSAR

#	m/z	Abs. Int.	Rel. Int.
85	134.10	48	0.12
86	135.20	166	0.40
87	136.20	436	1.05
88	137.20	150	0.36
89	138.20	439	1.06
90	139.20	470	1.13
91	140.20	339	0.82
92	141.20	200	0.48
93	142.20	86	0.21
94	143.20	133	0.32
95	144.20	35	0.08
96	145.10	312	0.75
97	146.10	113	0.27
98	147.20	159	0.38
99	148.10	41	0.10
100	149.20	302	0.73
101	150.20	143	0.34
102	151.20	751	1.81
103	152.20	560	1.35
104	153.20	338	0.81
105	154.20	209	0.50
106	155.20	46	0.11
107	156.10	310	0.75
108	157.10	119	0.29
109	158.10	12	0.03

#	m/z	Abs. Int.	Rel. Int.
110	161.10	6	0.01
111	162.20	25	0.06
112	163.20	20	0.05
113	164.10	6	0.01
114	165.20	200	0.48
115	166.20	433	1.04
116	167.20	180	0.43
117	168.20	169	0.41
118	169.10	61	0.15
119	171.20	12	0.03
120	173.10	14	0.03
121	174.20	40	0.10
122	175.20	36	0.09
123	176.10	62	0.15
124	177.20	21	0.05
125	179.20	416	1.00
126	180.10	256	0.62
127	181.20	45	0.11
128	182.20	66	0.16
129	184.20	19	0.05
130	190.20	14	0.03
131	191.10	7	0.02
132	193.20	41	0.10
133	195.20	6	0.01
134	196.20	76	0.18

#	m/z	Abs. Int.	Rel. Int.
135	207.10	220	0.53
136	208.10	32	0.08
137	209.10	6	0.01
138	224.20	41	0.10
139	227.20	12	0.03
140	240.10	6	0.01
141	253.10	177	0.43
142	255.10	6	0.01
143	265.20	6	0.01
144	274.10	6	0.01
145	282.10	7	0.02
146	296.20	6	0.01
147	323.10	7	0.02
148	373.20	6	0.01
149	384.10	6	0.01
150	457.10	6	0.01
151	480.10	6	0.01
152	546.10	7	0.02
153	586.20	6	0.01
154	615.10	7	0.02
155	619.10	6	0.01
156	624.10	6	0.01
157	644.10	6	0.01
158	645.10	6	0.01

Line#:4 R.Time:17.1(Scan#:3944)
MassPeaks:178
RawMode:Averaged 17.1-17.2(3929-3963) BasePeak:55(28432)
BG Mode:None Group 1 - Event 1

#	m/z	Abs. Int.	Rel. Int.
1	50.10	1159	4.08
2	51.15	1763	6.20
3	52.15	1040	3.66
4	53.15	3620	12.73
5	54.15	4391	15.44
6	55.15	28432	100.00
7	56.15	14112	49.63
8	57.15	25553	89.87
9	58.15	1746	6.14
10	59.15	915	3.22
11	60.10	2531	8.90
12	61.10	775	2.73
13	62.10	440	1.55
14	63.10	1021	3.59
15	64.05	763	2.68
16	65.10	1631	5.74
17	66.15	1346	4.73
18	67.10	5872	20.65
19	68.15	3821	13.44
20	69.15	19199	67.53
21	70.15	12614	44.37
22	71.15	10934	38.46
23	72.15	1108	3.90
24	73.10	7558	26.58
25	74.10	628	2.21
26	75.10	836	2.94
27	76.10	485	1.71
28	77.10	2664	9.37
29	78.10	1067	3.75
30	79.10	1893	6.66
31	80.15	676	2.38
32	81.15	3910	13.75
33	82.15	5552	19.53
34	83.15	18450	64.89
35	84.15	6433	22.63
36	85.15	5253	18.48
37	86.15	635	2.23
38	87.10	300	1.06
39	88.10	162	0.57
40	89.10	675	2.37
41	90.10	257	0.90
42	91.15	1552	5.46
43	92.10	532	1.87
44	93.15	794	2.79
45	94.10	809	2.85
46	95.15	1788	6.29
47	96.15	3020	10.62
48	97.15	14673	51.61
49	98.20	3570	12.56

#	m/z	Abs. Int.	Rel. Int.
50	99.20	1177	4.14
51	100.20	132	0.46
52	101.20	295	1.04
53	102.20	706	2.48
54	103.20	388	1.36
55	104.20	116	0.41
56	105.15	800	2.81
57	106.20	356	1.25
58	107.15	945	3.32
59	108.20	596	2.10
60	109.15	916	3.22
61	110.15	2559	9.00
62	111.15	6559	23.07
63	112.20	1849	6.50
64	113.20	538	1.89
65	114.20	73	0.26
66	115.10	455	1.60
67	116.20	429	1.51
68	117.20	359	1.26
69	118.10	212	0.75
70	119.20	489	1.72
71	120.10	270	0.95
72	121.20	484	1.70
73	122.15	887	3.12
74	123.20	978	3.44
75	124.20	834	2.93
76	125.20	2403	8.45
77	126.20	1053	3.70
78	127.20	356	1.25
79	128.10	176	0.62
80	129.20	459	1.61
81	130.10	86	0.30
82	131.20	261	0.92
83	132.10	25	0.09
84	133.10	323	1.14
85	134.10	96	0.34
86	135.10	584	2.05
87	136.20	717	2.52
88	137.15	497	1.75
89	138.15	896	3.15
90	139.20	1473	5.18
91	140.20	580	2.04
92	141.20	183	0.64
93	142.10	64	0.23
94	143.20	85	0.30
95	144.10	56	0.20
96	145.20	168	0.59
97	146.10	35	0.12
98	147.20	236	0.83

#	m/z	Abs. Int.	Rel. Int.
99	148.20	27	0.09
100	149.20	380	1.34
101	150.20	99	0.35
102	151.15	851	2.99
103	152.25	404	1.42
104	153.15	1808	6.36
105	154.20	2350	8.27
106	155.10	308	1.08
107	156.20	30	0.11
108	157.10	21	0.07
109	158.10	90	0.32
110	159.10	40	0.14
111	161.10	62	0.22
112	162.10	63	0.22
113	163.10	41	0.14
114	164.10	192	0.68
115	165.10	282	0.99
116	166.20	464	1.63
117	167.20	391	1.38
118	168.20	193	0.68
119	169.20	14	0.05
120	171.10	12	0.04
121	172.10	12	0.04
122	175.10	35	0.12
123	176.10	5	0.02
124	177.10	84	0.30
125	178.20	18	0.06
126	179.10	437	1.54
127	180.20	186	0.65
128	181.20	86	0.30
129	182.20	97	0.34
130	183.10	26	0.09
131	185.10	12	0.04
132	186.20	11	0.04
133	187.10	13	0.05
134	189.10	6	0.02
135	191.20	27	0.09
136	192.10	104	0.37
137	193.20	49	0.17
138	194.10	31	0.11
139	195.20	16	0.06
140	196.10	52	0.18
141	198.10	899	3.16
142	199.10	70	0.25
143	200.10	6	0.02
144	201.10	5	0.02
145	202.10	25	0.09
146	205.10	6	0.02
147	206.10	15	0.05

#	m/z	Abs. Int.	Rel. Int.
148	207.10	199	0.70
149	208.10	88	0.31
150	209.20	25	0.09
151	210.10	40	0.14
152	211.10	13	0.05
153	213.10	12	0.04
154	217.10	6	0.02
155	220.10	6	0.02
156	221.10	5	0.02
157	224.20	59	0.21
158	252.20	12	0.04
159	255.20	6	0.02
160	266.10	13	0.05
161	281.10	5	0.02
162	282.10	13	0.05
163	346.20	6	0.02
164	372.10	17	0.06
165	385.10	5	0.02
166	404.20	6	0.02
167	425.10	5	0.02
168	437.10	6	0.02
169	481.10	5	0.02
170	497.10	19	0.07
171	526.10	5	0.02
172	552.10	12	0.04
173	578.10	6	0.02
174	587.10	6	0.02
175	600.10	6	0.02
176	619.10	11	0.04
177	676.10	5	0.02
178	690.10	5	0.02

Line#:5 R.Time:17.6(Scan#:4093)
MassPeaks:163
RawMode:Averaged 17.6-17.7(4077-4108) BasePeak:68(45931)
BG Mode:None Group 1 - Event 1

#	m/z	Abs. Int.	Rel. Int.
1	50.10	1187	2.58
2	51.15	1928	4.20
3	52.10	1571	3.42
4	53.15	8424	18.34
5	54.15	2718	5.92
6	55.15	38142	83.04
7	56.15	11569	25.19
8	57.15	41355	90.04
9	58.15	2538	5.53
10	59.15	625	1.36
11	60.10	3175	6.91
12	61.10	893	1.94
13	62.10	381	0.83
14	63.10	836	1.82
15	64.10	587	1.28
16	65.10	3596	7.83
17	66.15	1809	3.94
18	67.10	26717	58.17
19	68.15	45931	100.00
20	69.15	32820	71.46
21	70.15	9394	20.45
22	71.15	19180	41.76
23	72.15	1608	3.50
24	73.10	11006	23.96
25	74.10	831	1.81
26	75.15	797	1.74
27	76.10	476	1.04
28	77.10	3770	8.21
29	78.10	1000	2.18
30	79.10	8980	19.55
31	80.10	2503	5.45
32	81.15	24203	52.69
33	82.15	27632	60.16
34	83.15	20076	43.71
35	84.15	2934	6.39
36	85.15	6147	13.38
37	86.15	629	1.37
38	87.10	273	0.59
39	88.10	72	0.16
40	89.10	572	1.25
41	90.10	178	0.39
42	91.10	2410	5.25
43	92.10	569	1.24
44	93.15	2669	5.81
45	94.15	3898	8.49
46	95.15	29640	64.53
47	96.15	10768	23.44
48	97.15	13649	29.72
49	98.15	1775	3.86
50	99.15	1081	2.35
51	100.20	157	0.34
52	101.10	301	0.66
53	102.15	1000	2.18
54	103.20	419	0.91
55	104.10	124	0.27
56	105.15	697	1.52
57	106.10	241	0.52
58	107.15	1239	2.70
59	108.15	814	1.77
60	109.15	9810	21.36
61	110.15	4341	9.45
62	111.20	6263	13.64
63	112.15	1040	2.26
64	113.20	476	1.04
65	114.20	56	0.12
66	115.20	371	0.81
67	116.20	449	0.98
68	117.20	408	0.89
69	118.20	67	0.15
70	119.20	314	0.68
71	120.20	129	0.28
72	121.10	626	1.36
73	122.25	680	1.48
74	123.20	13725	29.88
75	124.20	4874	10.61
76	125.20	1958	4.26
77	126.15	899	1.96
78	127.20	379	0.83
79	128.20	185	0.40
80	129.20	419	0.91
81	130.20	67	0.15
82	131.20	219	0.48
83	132.20	28	0.06
84	133.10	235	0.51
85	134.20	68	0.15
86	135.20	473	1.03
87	136.20	582	1.27
88	137.20	2279	4.96
89	138.20	1141	2.48
90	139.20	329	0.72
91	140.20	227	0.49
92	141.20	158	0.34
93	142.20	6	0.01
94	143.20	91	0.20
95	144.20	20	0.04
96	145.20	209	0.46
97	146.20	21	0.05
98	147.10	69	0.15
99	148.10	84	0.18
100	149.20	383	0.83
101	150.20	159	0.35
102	151.20	1110	2.42
103	152.20	563	1.23
104	153.20	301	0.66
105	154.20	161	0.35
106	155.20	14	0.03
107	156.20	12	0.03
108	157.20	6	0.01
109	158.20	6	0.01
110	160.20	13	0.03
111	161.20	19	0.04
112	162.20	27	0.06
113	163.20	27	0.06
114	165.25	434	0.94
115	166.30	460	1.00
116	167.20	203	0.44
117	168.20	56	0.12
118	169.20	28	0.06
119	171.20	27	0.06
120	172.10	6	0.01
121	174.10	36	0.08
122	176.20	6	0.01
123	177.20	60	0.13
124	178.20	30	0.07
125	179.20	759	1.65
126	180.20	250	0.54
127	181.20	16	0.03
128	182.20	22	0.05
129	185.20	6	0.01
130	189.20	13	0.03
131	191.10	63	0.14
132	192.10	293	0.64
133	193.15	488	1.06
134	194.20	101	0.22
135	195.10	73	0.16
136	197.20	20	0.04
137	203.20	6	0.01
138	204.10	6	0.01
139	207.20	276	0.60
140	208.20	163	0.35
141	209.20	6	0.01
142	221.20	31	0.07
143	235.20	7	0.02
144	236.20	46	0.10
145	239.20	6	0.01
146	244.20	6	0.01
147	249.20	101	0.22
148	250.20	29	0.06
149	263.20	109	0.24
150	264.20	7	0.02
151	272.20	6	0.01
152	278.20	98	0.21
153	279.20	7	0.02
154	280.20	13	0.03
155	281.20	57	0.12
156	297.20	7	0.02
157	300.20	7	0.02
158	309.10	12	0.03
159	400.20	6	0.01
160	421.20	7	0.02
161	571.20	13	0.03
162	580.20	6	0.01
163	651.20	7	0.02

Line#:6 R.Time:18.1(Scan#:4222)
MassPeaks:189
RawMode:Averaged 18.0-18.1(4209-4245) BasePeak:57(14159)
BG Mode:None Group 1 - Event 1

#	m/z	Abs. Int.	Rel. Int.
1	50.15	1043	7.37
2	51.10	1441	10.18
3	52.15	952	6.72
4	53.15	4181	29.53
5	54.15	1242	8.77
6	55.15	11043	77.99

DEPTT. OF BOTANICAL & ENVIRONMENTAL SCIENCES, G.N.D.U. AMRITSAR

#	m/z	Abs. Int.	Rel. Int.	#	m/z	Abs. Int.	Rel. Int.	#	m/z	Abs. Int.	Rel. Int.
7	56.15	3696	26.10	68	117.10	495	3.50	129	178.10	55	0.39
8	57.15	14159	100.00	69	118.10	126	0.89	130	179.25	567	4.00
9	58.15	1343	9.49	70	119.15	561	3.96	131	180.20	257	1.82
10	59.15	664	4.69	71	120.10	148	1.05	132	181.10	89	0.63
11	60.10	1126	7.95	72	121.10	554	3.91	133	182.10	5	0.04
12	61.10	504	3.56	73	122.20	392	2.77	134	185.20	12	0.08
13	62.10	353	2.49	74	123.20	3936	27.80	135	187.10	14	0.10
14	63.15	913	6.45	75	124.20	1619	11.43	136	188.20	11	0.08
15	64.05	638	4.51	76	125.20	670	4.73	137	189.20	251	1.77
16	65.10	1712	12.09	77	126.20	356	2.51	138	190.20	51	0.36
17	66.10	1096	7.74	78	127.20	284	2.01	139	191.20	108	0.76
18	67.10	8072	57.01	79	128.20	290	2.05	140	192.20	197	1.39
19	68.10	9376	66.22	80	129.20	542	3.83	141	193.20	151	1.07
20	69.15	8750	61.80	81	130.20	227	1.60	142	194.20	47	0.33
21	70.15	2855	20.16	82	131.20	352	2.49	143	195.20	5	0.04
22	71.15	6084	42.97	83	132.20	91	0.64	144	201.20	36	0.25
23	72.15	565	3.99	84	133.15	395	2.79	145	203.20	71	0.50
24	73.10	2169	15.32	85	134.10	114	0.81	146	204.20	55	0.39
25	74.10	442	3.12	86	135.15	487	3.44	147	205.10	364	2.57
26	75.10	857	6.05	87	136.20	539	3.81	148	206.10	53	0.37
27	76.10	485	3.43	88	137.20	775	5.47	149	207.20	225	1.59
28	77.10	2962	20.92	89	138.20	380	2.68	150	208.20	79	0.56
29	78.15	906	6.40	90	139.20	217	1.53	151	217.15	247	1.74
30	79.10	6434	45.44	91	140.20	78	0.55	152	218.20	54	0.38
31	80.10	1439	10.16	92	141.20	167	1.18	153	219.20	34	0.24
32	81.15	12249	86.51	93	142.10	55	0.39	154	220.10	148	1.05
33	82.15	9644	68.11	94	143.10	78	0.55	155	221.20	36	0.25
34	83.15	5761	40.69	95	144.10	65	0.46	156	222.20	12	0.08
35	84.15	1136	8.02	96	145.10	226	1.60	157	223.00	5	0.04
36	85.15	2455	17.34	97	146.10	57	0.40	158	231.10	5	0.04
37	86.10	354	2.50	98	147.10	247	1.74	159	232.20	82	0.58
38	87.10	292	2.06	99	148.10	118	0.83	160	233.10	35	0.25
39	88.20	164	1.16	100	149.10	609	4.30	161	235.20	5	0.04
40	89.10	535	3.78	101	150.20	148	1.05	162	240.10	5	0.04
41	90.10	214	1.51	102	151.20	784	5.54	163	243.00	5	0.04
42	91.10	2077	14.67	103	152.20	365	2.58	164	261.20	35	0.25
43	92.10	579	4.09	104	153.10	209	1.48	165	263.20	27	0.19
44	93.15	1376	9.72	105	154.20	128	0.90	166	278.30	231	1.63
45	94.15	1661	11.73	106	155.10	36	0.25	167	279.20	17	0.12
46	95.15	8789	62.07	107	156.20	5	0.04	168	280.10	6	0.04
47	96.15	3318	23.43	108	157.10	39	0.28	169	281.20	11	0.08
48	97.20	3641	25.72	109	158.10	17	0.12	170	282.10	6	0.04
49	98.15	663	4.68	110	159.10	118	0.83	171	284.10	5	0.04
50	99.15	524	3.70	111	160.10	53	0.37	172	312.20	5	0.04
51	100.10	12	0.08	112	161.10	145	1.02	173	333.30	5	0.04
52	101.20	318	2.25	113	162.10	40	0.28	174	366.10	5	0.04
53	102.20	359	2.54	114	163.10	205	1.45	175	374.20	5	0.04
54	103.10	465	3.28	115	164.10	21	0.15	176	381.20	6	0.04
55	104.20	160	1.13	116	165.20	297	2.10	177	412.10	5	0.04
56	105.10	844	5.96	117	166.20	370	2.61	178	429.20	5	0.04
57	106.10	250	1.77	118	167.10	798	5.64	179	452.30	5	0.04
58	107.15	916	6.47	119	168.00	167	1.18	180	459.20	5	0.04
59	108.05	636	4.49	120	169.10	43	0.30	181	472.10	6	0.04
60	109.15	3215	22.71	121	170.10	11	0.08	182	513.10	12	0.08
61	110.15	1905	13.45	122	171.20	23	0.16	183	526.20	5	0.04
62	111.20	1663	11.75	123	172.10	5	0.04	184	535.20	5	0.04
63	112.15	424	2.99	124	173.10	54	0.38	185	584.20	11	0.08
64	113.20	262	1.85	125	174.20	11	0.08	186	594.10	6	0.04
65	114.10	36	0.25	126	175.20	312	2.20	187	624.20	5	0.04
66	115.10	556	3.93	127	176.20	51	0.36	188	653.30	6	0.04
67	116.10	506	3.57	128	177.20	251	1.77	189	689.20	5	0.04

Line#:7 R.Time:18.8(Scan#:4455)
MassPeaks:199
RawMode:Averaged 18.7-18.9(4422-4474) BasePeak:73(43531)
BG Mode:None Group 1 - Event 1

#	m/z	Abs. Int.	Rel. Int.	#	m/z	Abs. Int.	Rel. Int.	#	m/z	Abs. Int.	Rel. Int.
1	50.15	936	2.15	14	63.10	827	1.90	27	76.10	507	1.16
2	51.15	1310	3.01	15	64.05	589	1.35	28	77.10	2038	4.68
3	52.10	859	1.97	16	65.10	1420	3.26	29	78.10	831	1.91
4	53.15	3732	8.57	17	66.10	993	2.28	30	79.10	2617	6.01
5	54.15	3497	8.03	18	67.10	5746	13.20	31	80.15	909	2.09
6	55.15	39239	90.14	19	68.15	3715	8.53	32	81.15	4416	10.14
7	56.15	9809	22.53	20	69.15	20882	47.97	33	82.15	3858	8.86
8	57.15	41685	95.76	21	70.15	6399	14.70	34	83.15	13615	31.28
9	58.15	2384	5.48	22	71.15	22037	50.62	35	84.15	5744	13.20
10	59.15	2702	6.21	23	72.15	1542	3.54	36	85.15	12951	29.75
11	60.10	41848	96.13	24	73.10	43531	100.00	37	86.15	1311	3.01
12	61.10	11132	25.57	25	74.10	4667	10.72	38	87.10	12138	27.88
13	62.10	738	1.70	26	75.10	1083	2.49	39	88.10	1973	4.53

DEPTT. OF BOTANICAL & ENVIRONMENTAL SCIENCES, G.N.D.U. AMRITSAR

#	m/z	Abs. Int.	Rel. Int.
40	89.10	670	1.54
41	90.10	193	0.44
42	91.10	1500	3.45
43	92.10	467	1.07
44	93.10	1643	3.77
45	94.10	1068	2.45
46	95.15	2994	6.88
47	96.15	3032	6.97
48	97.15	9884	22.71
49	98.15	4968	11.41
50	99.15	4089	9.39
51	100.15	645	1.48
52	101.15	4488	10.31
53	102.10	1970	4.53
54	103.10	510	1.17
55	104.00	247	0.57
56	105.10	820	1.88
57	106.10	345	0.79
58	107.15	1252	2.88
59	108.10	559	1.28
60	109.15	1419	3.26
61	110.15	2154	4.95
62	111.20	3799	8.73
63	112.15	1581	3.63
64	113.20	2004	4.60
65	114.15	310	0.71
66	115.10	6283	14.43
67	116.15	2479	5.69
68	117.10	609	1.40
69	118.10	192	0.44
70	119.10	473	1.09
71	120.20	184	0.42
72	121.15	1004	2.31
73	122.20	417	0.96
74	123.20	1002	2.30
75	124.15	830	1.91
76	125.20	1521	3.49
77	126.15	761	1.75
78	127.20	1139	2.62
79	128.25	370	0.85
80	129.15	12619	28.99
81	130.20	1612	3.70
82	131.20	459	1.05
83	132.20	41	0.09
84	133.20	361	0.83
85	134.20	93	0.21
86	135.20	711	1.63
87	136.20	513	1.18
88	137.20	445	1.02
89	138.20	594	1.36
90	139.20	656	1.51
91	140.15	305	0.70
92	141.25	623	1.43
93	142.20	158	0.36

#	m/z	Abs. Int.	Rel. Int.
94	143.15	2730	6.27
95	144.15	667	1.53
96	145.20	230	0.53
97	146.20	25	0.06
98	147.20	302	0.69
99	148.20	55	0.13
100	149.10	750	1.72
101	150.10	187	0.43
102	151.10	788	1.81
103	152.15	492	1.13
104	153.20	386	0.89
105	154.20	287	0.66
106	155.20	244	0.56
107	156.20	32	0.07
108	157.20	3751	8.62
109	158.20	611	1.40
110	159.20	105	0.24
111	161.20	47	0.11
112	162.20	71	0.16
113	163.20	219	0.50
114	164.20	68	0.16
115	165.20	307	0.71
116	166.20	478	1.10
117	167.20	480	1.10
118	168.25	319	0.73
119	169.20	60	0.14
120	170.20	3	0.01
121	171.25	3400	7.81
122	172.25	470	1.08
123	173.20	104	0.24
124	176.20	8	0.02
125	177.20	180	0.41
126	178.20	50	0.11
127	179.20	436	1.00
128	180.20	214	0.49
129	181.20	82	0.19
130	182.20	83	0.19
131	183.20	12	0.03
132	185.20	2934	6.74
133	186.20	439	1.01
134	187.20	47	0.11
135	188.10	3	0.01
136	189.20	24	0.06
137	191.20	100	0.23
138	192.20	153	0.35
139	193.10	92	0.21
140	194.25	392	0.90
141	195.20	110	0.25
142	196.20	101	0.23
143	197.20	33	0.08
144	199.20	1642	3.77
145	200.20	241	0.55
146	201.20	17	0.04
147	202.20	3	0.01

#	m/z	Abs. Int.	Rel. Int.
148	203.10	8	0.02
149	205.10	33	0.08
150	207.20	188	0.43
151	208.20	46	0.11
152	210.20	36	0.08
153	213.20	3710	8.52
154	214.20	569	1.31
155	215.20	50	0.11
156	219.20	9	0.02
157	220.20	73	0.17
158	221.20	17	0.04
159	223.10	21	0.05
160	225.10	4	0.01
161	227.20	1069	2.46
162	228.20	194	0.45
163	229.20	3	0.01
164	236.20	36	0.08
165	237.20	14	0.03
166	239.20	67	0.15
167	241.20	3	0.01
168	247.20	3	0.01
169	251.20	3	0.01
170	254.10	3	0.01
171	256.25	1560	3.58
172	257.20	292	0.67
173	258.20	23	0.05
174	265.10	4	0.01
175	267.10	8	0.02
176	281.20	25	0.06
177	282.20	3	0.01
178	300.10	4	0.01
179	301.30	4	0.01
180	302.10	3	0.01
181	313.30	3	0.01
182	314.20	7	0.02
183	321.10	3	0.01
184	326.10	3	0.01
185	347.20	4	0.01
186	390.20	4	0.01
187	395.20	3	0.01
188	398.10	4	0.01
189	420.10	4	0.01
190	440.10	4	0.01
191	451.10	8	0.02
192	513.20	3	0.01
193	520.30	4	0.01
194	538.10	3	0.01
195	545.10	3	0.01
196	548.10	3	0.01
197	549.30	3	0.01
198	636.30	3	0.01
199	637.30	4	0.01

Line#:8 R.Time:19.2(Scan#:4564)
MassPeaks:165
RawMode:Averaged 19.2-19.3(4550-4577) BasePeak:57(34351)
BG Mode:None Group 1 - Event 1

#	m/z	Abs. Int.	Rel. Int.
1	50.10	887	2.58
2	51.10	1242	3.62
3	52.20	848	2.47
4	53.15	3023	8.80
5	54.15	4755	13.84
6	55.15	33715	98.15
7	56.15	16196	47.15
8	57.15	34351	100.00
9	58.15	1794	5.22
10	59.10	557	1.62
11	60.10	1670	4.86
12	61.10	694	2.02
13	62.10	271	0.79
14	63.10	787	2.29
15	64.10	504	1.47
16	65.15	1376	4.01
17	66.15	1233	3.59
18	67.15	7325	21.32
19	68.10	5151	15.00
20	69.15	24779	72.13

#	m/z	Abs. Int.	Rel. Int.
21	70.15	14817	43.13
22	71.15	15963	46.47
23	72.15	905	2.63
24	73.10	2179	6.34
25	74.10	442	1.29
26	75.10	655	1.91
27	76.10	397	1.16
28	77.10	1866	5.43
29	78.10	591	1.72
30	79.10	1802	5.25
31	80.10	716	2.08
32	81.15	5329	15.51
33	82.15	7782	22.65
34	83.15	23865	69.47
35	84.15	8436	24.56
36	85.15	8524	24.81
37	86.15	736	2.14
38	87.10	588	1.71
39	88.10	92	0.27
40	89.20	438	1.28

#	m/z	Abs. Int.	Rel. Int.
41	90.10	77	0.22
42	91.10	3113	9.06
43	92.10	514	1.50
44	93.10	760	2.21
45	94.15	702	2.04
46	95.15	2445	7.12
47	96.15	4251	12.38
48	97.15	21219	61.77
49	98.20	4679	13.62
50	99.15	1994	5.80
51	100.20	225	0.66
52	101.20	392	1.14
53	102.20	252	0.73
54	103.20	353	1.03
55	104.20	182	0.53
56	105.20	596	1.74
57	106.20	204	0.59
58	107.20	570	1.66
59	108.20	324	0.94
60	109.15	1072	3.12

DEPTT. OF BOTANICAL & ENVIRONMENTAL SCIENCES, G.N.D.U. AMRITSAR

#	m/z	Abs. Int.	Rel. Int.
61	110.15	2340	6.81
62	111.20	9321	27.13
63	112.20	2651	7.72
64	113.20	1039	3.02
65	114.20	127	0.37
66	115.20	496	1.44
67	116.20	417	1.21
68	117.20	402	1.17
69	118.10	141	0.41
70	119.20	410	1.19
71	120.10	187	0.54
72	121.20	372	1.08
73	122.20	211	0.61
74	123.20	791	2.30
75	124.20	1057	3.08
76	125.20	3624	10.55
77	126.20	1387	4.04
78	127.25	601	1.75
79	128.20	162	0.47
80	129.20	664	1.93
81	130.10	126	0.37
82	131.20	233	0.68
83	132.20	7	0.02
84	133.20	338	0.98
85	134.20	31	0.09
86	135.20	259	0.75
87	136.20	400	1.16
88	137.30	365	1.06
89	138.25	517	1.51
90	139.20	1225	3.57
91	140.25	698	2.03
92	141.20	279	0.81
93	142.20	25	0.07
94	143.20	202	0.59
95	144.20	33	0.10

#	m/z	Abs. Int.	Rel. Int.
96	145.20	222	0.65
97	146.10	97	0.28
98	147.20	201	0.59
99	148.20	17	0.05
100	149.10	383	1.11
101	150.10	113	0.33
102	151.20	616	1.79
103	152.20	449	1.31
104	153.25	620	1.80
105	154.20	384	1.12
106	155.20	151	0.44
107	156.20	14	0.04
108	157.20	99	0.29
109	159.20	66	0.19
110	161.20	44	0.13
111	162.20	47	0.14
112	163.20	54	0.16
113	164.20	15	0.04
114	165.20	203	0.59
115	166.20	428	1.25
116	167.25	428	1.25
117	168.20	244	0.71
118	169.20	44	0.13
119	171.20	60	0.17
120	172.20	7	0.02
121	177.20	69	0.20
122	179.20	297	0.86
123	180.20	148	0.43
124	181.20	161	0.47
125	182.20	145	0.42
126	183.20	17	0.05
127	185.20	60	0.17
128	187.20	16	0.05
129	191.20	40	0.12
130	192.20	39	0.11

#	m/z	Abs. Int.	Rel. Int.
131	193.20	40	0.12
132	194.20	47	0.14
133	195.20	57	0.17
134	196.20	60	0.17
135	199.20	7	0.02
136	201.20	7	0.02
137	206.20	15	0.04
138	207.20	292	0.85
139	208.10	47	0.14
140	209.20	52	0.15
141	210.30	43	0.13
142	218.20	8	0.02
143	221.20	7	0.02
144	222.30	14	0.04
145	223.20	26	0.08
146	224.20	58	0.17
147	227.20	15	0.04
148	229.20	8	0.02
149	236.20	7	0.02
150	242.20	7	0.02
151	244.20	14	0.04
152	252.20	45	0.13
153	266.20	16	0.05
154	272.20	7	0.02
155	280.30	8	0.02
156	281.20	16	0.05
157	290.20	7	0.02
158	351.20	7	0.02
159	355.20	7	0.02
160	371.10	7	0.02
161	403.10	7	0.02
162	532.10	7	0.02
163	546.20	7	0.02
164	576.20	9	0.03
165	590.20	8	0.02

Line#:9   R.Time:20.3(Scan#:4877)
MassPeaks:173
RawMode:Averaged 20.2-20.3(4860-4898) BasePeak:71(37806)
BG Mode:None Group 1 - Event 1

#	m/z	Abs. Int.	Rel. Int.
1	50.10	809	2.14
2	51.15	1074	2.84
3	52.15	713	1.89
4	53.10	3436	9.09
5	54.15	1416	3.75
6	55.15	18344	48.52
7	56.15	8624	22.81
8	57.15	21365	56.51
9	58.15	1502	3.97
10	59.10	539	1.43
11	60.15	949	2.51
12	61.10	457	1.21
13	62.10	320	0.85
14	63.10	700	1.85
15	64.10	448	1.18
16	65.10	1508	3.99
17	66.10	831	2.20
18	67.10	8812	23.31
19	68.15	10606	28.05
20	69.15	15064	39.85
21	70.15	7961	21.06
22	71.15	37806	100.00
23	72.15	2084	5.51
24	73.10	1637	4.33
25	74.10	393	1.04
26	75.10	663	1.75
27	76.10	378	1.00
28	77.10	2074	5.49
29	78.15	668	1.77
30	79.15	3591	9.50
31	80.15	1692	4.48
32	81.15	12596	33.32
33	82.15	7437	19.67
34	83.15	8606	22.76
35	84.15	5129	13.57
36	85.15	5061	13.39
37	86.15	1422	3.76
38	87.10	592	1.57
39	88.20	40	0.11

#	m/z	Abs. Int.	Rel. Int.
40	89.10	397	1.05
41	90.10	78	0.21
42	91.10	2938	7.77
43	92.10	571	1.51
44	93.10	1469	3.89
45	94.15	1401	3.71
46	95.15	8879	23.49
47	96.15	3605	9.54
48	97.15	6122	16.19
49	98.15	2025	5.36
50	99.15	1539	4.07
51	100.20	301	0.80
52	101.20	334	0.88
53	102.20	242	0.64
54	103.20	306	0.81
55	104.10	182	0.48
56	105.20	770	2.04
57	106.20	254	0.67
58	107.15	888	2.35
59	108.15	478	1.26
60	109.20	3431	9.08
61	110.20	2206	5.84
62	111.20	3892	10.29
63	112.20	1203	3.18
64	113.15	763	2.02
65	114.20	33	0.09
66	115.20	511	1.35
67	116.10	427	1.13
68	117.20	419	1.11
69	118.20	92	0.24
70	119.20	363	0.96
71	120.20	142	0.38
72	121.10	671	1.77
73	122.20	565	1.49
74	123.20	6452	17.07
75	124.20	2028	5.36
76	125.20	1391	3.68
77	126.20	1575	4.17
78	127.20	660	1.75

#	m/z	Abs. Int.	Rel. Int.
79	128.20	186	0.49
80	129.20	482	1.27
81	130.20	90	0.24
82	131.20	277	0.73
83	132.20	36	0.10
84	133.20	298	0.79
85	134.15	1026	2.71
86	135.10	433	1.15
87	136.20	409	1.08
88	137.20	787	2.08
89	138.20	462	1.22
90	139.20	404	1.07
91	140.20	393	1.04
92	141.20	309	0.82
93	142.20	18	0.05
94	143.20	153	0.40
95	145.20	194	0.51
96	146.20	40	0.11
97	147.20	232	0.61
98	148.20	18	0.05
99	149.20	546	1.44
100	150.20	131	0.35
101	151.25	708	1.87
102	152.20	336	0.89
103	153.20	192	0.51
104	154.20	55	0.15
105	155.20	45	0.12
106	156.20	23	0.06
107	157.20	46	0.12
108	158.20	5	0.01
109	159.20	39	0.10
110	160.10	39	0.10
111	161.20	33	0.09
112	162.20	11	0.03
113	163.20	86	0.23
114	164.20	22	0.06
115	165.20	306	0.81
116	166.20	318	0.84
117	167.20	179	0.47

DEPTT. OF BOTANICAL & ENVIRONMENTAL SCIENCES, G.N.D.U. AMRITSAR

#	m/z	Abs. Int.	Rel. Int.
118	168.20	84	0.22
119	169.20	23	0.06
120	171.20	10	0.03
121	173.20	17	0.04
122	175.20	10	0.03
123	176.20	5	0.01
124	177.20	56	0.15
125	179.25	442	1.17
126	180.20	120	0.32
127	181.20	54	0.14
128	182.30	79	0.21
129	183.20	10	0.03
130	185.20	36	0.10
131	189.20	40	0.11
132	191.20	25	0.07
133	192.20	6	0.02
134	193.20	114	0.30
135	194.20	35	0.09
136	195.20	16	0.04
137	196.30	65	0.17
138	197.30	18	0.05
139	199.20	17	0.04
140	200.30	5	0.01
141	201.20	6	0.02
142	203.20	5	0.01
143	205.10	6	0.02
144	207.20	220	0.58
145	208.20	46	0.12
146	209.20	33	0.09
147	210.20	21	0.06
148	211.20	5	0.01
149	215.20	10	0.03
150	219.10	5	0.01
151	220.20	10	0.03
152	223.20	5	0.01
153	234.20	10	0.03
154	243.20	5	0.01
155	249.20	16	0.04
156	250.20	5	0.01
157	253.20	11	0.03
158	263.20	11	0.03
159	278.20	59	0.16
160	279.20	5	0.01
161	281.20	39	0.10
162	355.20	5	0.01
163	376.20	5	0.01
164	441.10	5	0.01
165	455.20	5	0.01
166	496.20	5	0.01
167	546.20	5	0.01
168	576.20	5	0.01
169	586.20	5	0.01
170	597.20	5	0.01
171	635.20	5	0.01
172	671.20	11	0.03
173	686.20	5	0.01

Line#:10 R.Time:20.5(Scan#:4939)
MassPeaks:195
RawMode:Averaged 20.4-20.5(4930-4959) BasePeak:55(32494)
BG Mode:None Group 1 - Event 1

#	m/z	Abs. Int.	Rel. Int.
1	50.10	951	2.93
2	51.15	1442	4.44
3	52.15	923	2.84
4	53.15	4325	13.31
5	54.15	7198	22.15
6	55.15	32494	100.00
7	56.15	9535	29.34
8	57.15	14073	43.31
9	58.10	1214	3.74
10	59.10	1331	4.10
11	60.10	4733	14.57
12	61.10	981	3.02
13	62.10	359	1.10
14	63.10	844	2.60
15	64.10	507	1.56
16	65.10	2333	7.18
17	66.15	1833	5.64
18	67.10	15237	46.89
19	68.15	7211	22.19
20	69.10	20216	62.21
21	70.15	8134	25.03
22	71.15	6803	20.94
23	72.10	603	1.86
24	73.10	5656	17.41
25	74.10	808	2.49
26	75.10	851	2.62
27	76.10	456	1.40
28	77.10	3877	11.93
29	78.10	1726	5.31
30	79.10	9541	29.36
31	80.15	4034	12.41
32	81.15	10977	33.78
33	82.15	7189	22.12
34	83.15	13681	42.10
35	84.10	8617	26.52
36	85.15	3872	11.92
37	86.15	513	1.58
38	87.10	1964	6.04
39	88.10	363	1.12
40	89.10	613	1.89
41	90.10	196	0.60
42	91.10	4384	13.49
43	92.15	1105	3.40
44	93.15	4480	13.79
45	94.15	2827	8.70
46	95.15	8057	24.80
47	96.15	6541	20.13
48	97.15	10656	32.79
49	98.15	5296	16.30
50	99.15	1695	5.22
51	100.10	877	2.70
52	101.10	1539	4.74
53	102.10	524	1.61
54	103.10	490	1.51
55	104.10	337	1.04
56	105.10	1808	5.56
57	106.10	603	1.86
58	107.15	2286	7.04
59	108.15	2187	6.73
60	109.15	3447	10.61
61	110.15	3733	11.49
62	111.15	4378	13.47
63	112.15	1673	5.15
64	113.15	900	2.77
65	114.15	1100	3.39
66	115.10	1392	4.28
67	116.10	673	2.07
68	117.15	739	2.27
69	118.10	264	0.81
70	119.15	1490	4.59
71	120.15	631	1.94
72	121.15	1741	5.36
73	122.20	944	2.91
74	123.15	2344	7.21
75	124.20	1582	4.87
76	125.15	1649	5.07
77	126.20	816	2.51
78	127.15	1086	3.34
79	128.20	445	1.37
80	129.20	1144	3.52
81	130.20	318	0.98
82	131.20	512	1.58
83	132.20	152	0.47
84	133.15	1006	3.10
85	134.15	642	1.98
86	135.15	1216	3.74
87	136.20	906	2.79
88	137.20	1086	3.34
89	138.20	951	2.93
90	139.20	819	2.52
91	140.15	423	1.30
92	141.20	342	1.05
93	142.20	135	0.42
94	143.20	375	1.15
95	144.10	103	0.32
96	145.20	299	0.92
97	146.20	54	0.17
98	147.20	739	2.27
99	148.20	448	1.38
100	149.20	1012	3.11
101	150.20	412	1.27
102	151.20	1240	3.82
103	152.20	757	2.33
104	153.20	511	1.57
105	154.20	248	0.76
106	155.20	192	0.59
107	156.20	7	0.02
108	157.20	286	0.88
109	158.20	47	0.14
110	159.20	22	0.07
111	161.20	462	1.42
112	162.20	125	0.38
113	163.20	360	1.11
114	164.20	153	0.47
115	165.25	587	1.81
116	166.20	623	1.92
117	167.20	398	1.22
118	168.20	153	0.47
119	169.20	131	0.40
120	171.20	153	0.47
121	172.20	42	0.13
122	173.20	81	0.25
123	174.10	57	0.18
124	175.20	164	0.50
125	176.20	14	0.04
126	177.20	245	0.75
127	178.20	236	0.73
128	179.20	590	1.82
129	180.20	373	1.15
130	181.20	154	0.47
131	182.20	130	0.40
132	183.20	61	0.19
133	184.20	7	0.02
134	185.20	171	0.53
135	186.20	67	0.21
136	187.20	23	0.07
137	189.20	161	0.50
138	190.20	52	0.16
139	191.20	173	0.53
140	192.20	113	0.35
141	193.20	205	0.63
142	194.20	180	0.55
143	195.20	39	0.12
144	196.20	31	0.10
145	197.20	30	0.09
146	199.20	28	0.09
147	203.20	49	0.15
148	205.20	34	0.10
149	206.10	15	0.05
150	207.20	392	1.21
151	208.20	157	0.48
152	209.10	61	0.19
153	210.10	22	0.07
154	211.20	13	0.04
155	213.10	519	1.60
156	214.20	83	0.26
157	215.20	6	0.02
158	217.20	23	0.07
159	218.20	15	0.05
160	219.20	6	0.02
161	220.20	150	0.46
162	221.20	163	0.50
163	222.20	239	0.74
164	223.20	54	0.17
165	224.20	6	0.02

DEPTT. OF BOTANICAL & ENVIRONMENTAL SCIENCES, G.N.D.U. AMRITSAR

#	m/z	Abs. Int.	Rel. Int.	#	m/z	Abs. Int.	Rel. Int.	#	m/z	Abs. Int.	Rel. Int.
166	227.20	6	0.02	176	256.20	14	0.04	186	322.20	7	0.02
167	228.20	37	0.11	177	263.20	6	0.02	187	355.10	6	0.02
168	234.20	15	0.05	178	264.30	413	1.27	188	378.10	6	0.02
169	235.20	122	0.38	179	265.30	68	0.21	189	443.20	7	0.02
170	236.20	54	0.17	180	267.30	15	0.05	190	504.20	6	0.02
171	238.10	7	0.02	181	269.20	14	0.04	191	516.20	9	0.03
172	243.20	6	0.02	182	271.10	7	0.02	192	569.30	7	0.02
173	246.20	17	0.05	183	281.10	33	0.10	193	599.10	6	0.02
174	250.20	14	0.04	184	282.30	16	0.05	194	635.20	6	0.02
175	255.10	14	0.04	185	283.20	14	0.04	195	655.10	7	0.02

Line#:11 R.Time:20.7(Scan#:5013)
MassPeaks:189
RawMode:Averaged 20.6-20.8(4993-5035) BasePeak:57(21088)
BG Mode:None Group 1 - Event 1

#	m/z	Abs. Int.	Rel. Int.	#	m/z	Abs. Int.	Rel. Int.	#	m/z	Abs. Int.	Rel. Int.
1	50.15	809	3.84	64	113.20	1699	8.06	127	179.20	413	1.96
2	51.10	1087	5.15	65	114.10	353	1.67	128	180.20	173	0.82
3	52.15	758	3.59	66	115.10	2065	9.79	129	181.20	116	0.55
4	53.15	2333	11.06	67	116.15	1232	5.84	130	182.20	16	0.08
5	54.15	2295	10.88	68	117.10	748	3.55	131	183.20	86	0.41
6	55.15	18030	85.50	69	118.10	201	0.95	132	185.20	1434	6.80
7	56.15	4960	23.52	70	119.15	600	2.85	133	186.20	184	0.87
8	57.15	21088	100.00	71	120.10	286	1.36	134	187.20	59	0.28
9	58.15	1390	6.59	72	121.15	766	3.63	135	188.20	10	0.05
10	59.15	1582	7.50	73	122.20	393	1.86	136	189.20	31	0.15
11	60.10	11900	56.43	74	123.20	922	4.37	137	191.10	56	0.27
12	61.10	3802	18.03	75	124.20	648	3.07	138	192.20	55	0.26
13	62.10	420	1.99	76	125.15	967	4.59	139	193.20	95	0.45
14	63.10	716	3.40	77	126.20	565	2.68	140	194.20	49	0.23
15	64.20	452	2.14	78	127.25	1067	5.06	141	195.20	34	0.16
16	65.10	1310	6.21	79	128.20	420	1.99	142	196.20	5	0.02
17	66.10	820	3.89	80	129.15	4243	20.12	143	197.20	47	0.22
18	67.10	5456	25.87	81	130.15	730	3.46	144	199.20	535	2.54
19	68.15	2646	12.55	82	131.20	424	2.01	145	200.20	59	0.28
20	69.15	11191	53.07	83	132.30	62	0.29	146	201.10	10	0.05
21	70.15	4050	19.21	84	133.20	474	2.25	147	205.20	19	0.09
22	71.15	13096	62.10	85	134.20	184	0.87	148	207.20	272	1.29
23	72.15	1126	5.34	86	135.20	610	2.89	149	208.20	74	0.35
24	73.10	13271	62.93	87	136.10	481	2.28	150	209.20	5	0.02
25	74.10	1683	7.98	88	137.10	384	1.82	151	210.20	25	0.12
26	75.10	900	4.27	89	138.10	349	1.65	152	211.20	15	0.07
27	76.10	448	2.12	90	139.20	443	2.10	153	213.20	322	1.53
28	77.10	2131	10.11	91	140.20	197	0.93	154	214.20	51	0.24
29	78.15	836	3.96	92	141.25	594	2.82	155	215.20	49	0.23
30	79.10	3166	15.01	93	142.20	122	0.58	156	216.20	35	0.17
31	80.15	1202	5.70	94	143.20	974	4.62	157	219.10	5	0.02
32	81.15	4084	19.37	95	144.20	211	1.00	158	220.20	10	0.05
33	82.15	2794	13.25	96	145.20	226	1.07	159	221.20	16	0.08
34	83.15	7244	34.35	97	146.10	48	0.23	160	222.20	118	0.56
35	84.15	3236	15.35	98	147.20	329	1.56	161	223.20	42	0.20
36	85.15	8240	39.07	99	148.20	116	0.55	162	224.20	10	0.05
37	86.15	856	4.06	100	149.20	460	2.18	163	225.10	15	0.07
38	87.10	4571	21.68	101	150.20	196	0.93	164	227.25	382	1.81
39	88.10	971	4.60	102	151.20	687	3.26	165	228.30	72	0.34
40	89.10	571	2.71	103	152.20	379	1.80	166	237.30	10	0.05
41	90.20	92	0.44	104	153.20	321	1.52	167	241.25	856	4.06
42	91.10	2545	12.07	105	154.20	138	0.65	168	242.30	123	0.58
43	92.10	653	3.10	106	155.20	357	1.69	169	255.20	104	0.49
44	93.15	1701	8.07	107	156.20	58	0.28	170	256.20	17	0.08
45	94.15	1104	5.24	108	157.20	497	2.36	171	264.20	18	0.09
46	95.15	3074	14.58	109	158.20	147	0.70	172	265.20	23	0.11
47	96.15	2108	10.00	110	159.20	59	0.28	173	267.20	9	0.04
48	97.15	5235	24.82	111	160.20	4	0.02	174	272.30	9	0.04
49	98.15	2623	12.44	112	161.20	70	0.33	175	273.30	4	0.02
50	99.20	3263	15.47	113	162.20	23	0.11	176	284.35	379	1.80
51	100.15	492	2.33	114	163.10	155	0.74	177	285.30	42	0.20
52	101.10	1690	8.01	115	164.20	65	0.31	178	304.10	4	0.02
53	102.15	677	3.21	116	165.20	284	1.35	179	319.20	5	0.02
54	103.20	435	2.06	117	166.20	394	1.87	180	337.30	5	0.02
55	104.20	270	1.28	118	167.20	246	1.17	181	350.10	4	0.02
56	105.15	1354	6.42	119	168.20	66	0.31	182	353.20	5	0.02
57	106.10	406	1.93	120	169.20	179	0.85	183	375.20	4	0.02
58	107.15	1073	5.09	121	171.25	751	3.56	184	409.20	4	0.02
59	108.15	713	3.38	122	172.30	156	0.74	185	511.10	11	0.05
60	109.15	1380	6.54	123	173.20	59	0.28	186	573.20	4	0.02
61	110.15	1643	7.79	124	175.20	42	0.20	187	648.10	5	0.02
62	111.15	2494	11.83	125	177.20	113	0.54	188	656.20	5	0.02
63	112.15	1005	4.77	126	178.10	5	0.02	189	695.30	4	0.02

DEPTT. OF BOTANICAL & ENVIRONMENTAL SCIENCES, G.N.D.U. AMRITSAR

Line#:12 R.Time:21.1(Scan#:5127)
MassPeaks:179
RawMode:Averaged 21.1-21.2(5116-5147) BasePeak:57(28150)
BG Mode:None Group 1 - Event 1

#	m/z	Abs. Int.	Rel. Int.
1	50.00	790	2.81
2	51.15	1057	3.75
3	52.10	690	2.45
4	53.15	2347	8.34
5	54.15	3512	12.48
6	55.15	25037	88.94
7	56.15	11229	39.89
8	57.15	28150	100.00
9	58.15	1668	5.93
10	59.20	563	2.00
11	60.15	1260	4.48
12	61.20	572	2.03
13	62.10	323	1.15
14	63.10	709	2.52
15	64.10	448	1.59
16	65.15	1157	4.11
17	66.10	1044	3.71
18	67.10	6584	23.39
19	68.15	4025	14.30
20	69.15	18786	66.74
21	70.15	10783	38.31
22	71.15	15173	53.90
23	72.20	935	3.32
24	73.10	1799	6.39
25	74.10	393	1.40
26	75.10	790	2.81
27	76.10	367	1.30
28	77.05	1837	6.53
29	78.10	676	2.40
30	79.15	2112	7.50
31	80.15	840	2.98
32	81.15	4785	17.00
33	82.15	6356	22.58
34	83.15	18309	65.04
35	84.15	6136	21.80
36	85.15	8467	30.08
37	86.20	675	2.40
38	87.10	509	1.81
39	88.10	67	0.24
40	89.20	400	1.42
41	90.10	55	0.20
42	91.10	2041	7.25
43	92.10	603	2.14
44	93.10	922	3.28
45	94.15	768	2.73
46	95.15	2822	10.02
47	96.15	4079	14.49
48	97.15	16969	60.28
49	98.20	3691	13.11
50	99.20	2612	9.28
51	100.20	321	1.14
52	101.20	394	1.40
53	102.20	250	0.89
54	103.20	350	1.24
55	104.20	162	0.58
56	105.10	768	2.73
57	106.10	295	1.05
58	107.20	680	2.42
59	108.20	414	1.47
60	109.15	1245	4.42
61	110.15	2258	8.02
62	111.20	8130	28.88
63	112.20	2031	7.21
64	113.20	1339	4.76
65	114.20	149	0.53
66	115.20	577	2.05
67	116.20	510	1.81
68	117.20	533	1.89
69	118.20	94	0.33
70	119.20	452	1.61
71	120.20	90	0.32
72	121.20	470	1.67
73	122.20	258	0.92
74	123.20	838	2.98
75	124.20	1000	3.55
76	125.20	3334	11.84
77	126.20	1122	3.99
78	127.20	788	2.80
79	128.20	274	0.97
80	129.20	665	2.36
81	130.20	123	0.44
82	131.20	346	1.23
83	132.20	32	0.11
84	133.10	363	1.29
85	134.20	77	0.27
86	135.20	374	1.33
87	136.20	436	1.55
88	137.20	370	1.31
89	138.25	482	1.71
90	139.20	1223	4.34
91	140.20	476	1.69
92	141.20	453	1.61
93	142.20	130	0.46
94	143.10	63	0.22
95	145.20	214	0.76
96	146.10	6	0.02
97	147.20	282	1.00
98	148.20	44	0.16
99	149.20	355	1.26
100	150.20	63	0.22
101	151.20	633	2.25
102	152.25	435	1.55
103	153.25	623	2.21
104	154.25	312	1.11
105	155.20	202	0.72
106	156.10	19	0.07
107	157.20	39	0.14
108	161.20	47	0.17
109	162.10	14	0.05
110	163.20	47	0.17
111	165.20	203	0.72
112	166.20	420	1.49
113	167.25	380	1.35
114	168.30	230	0.82
115	169.30	79	0.28
116	171.20	22	0.08
117	173.20	14	0.05
118	174.10	15	0.05
119	175.20	26	0.09
120	176.20	28	0.10
121	177.10	40	0.14
122	178.20	6	0.02
123	179.20	426	1.51
124	180.20	229	0.81
125	181.30	176	0.63
126	182.20	122	0.43
127	183.20	52	0.18
128	185.30	37	0.13
129	186.20	6	0.02
130	187.10	7	0.02
131	189.20	20	0.07
132	191.20	43	0.15
133	192.10	86	0.31
134	193.20	62	0.22
135	194.30	73	0.26
136	195.30	103	0.37
137	196.30	62	0.22
138	198.20	7	0.02
139	207.30	234	0.83
140	208.20	58	0.21
141	209.30	49	0.17
142	210.20	80	0.28
143	211.20	20	0.07
144	213.10	6	0.02
145	216.20	13	0.05
146	218.20	6	0.02
147	220.20	6	0.02
148	221.20	12	0.04
149	222.30	6	0.02
150	224.30	29	0.10
151	225.30	15	0.05
152	227.30	27	0.10
153	236.30	13	0.05
154	238.30	13	0.05
155	250.30	26	0.09
156	252.30	14	0.05
157	254.20	6	0.02
158	267.20	12	0.04
159	280.30	6	0.02
160	281.20	26	0.09
161	293.20	7	0.02
162	294.20	7	0.02
163	302.20	6	0.02
164	308.30	6	0.02
165	343.20	6	0.02
166	355.20	13	0.05
167	419.20	7	0.02
168	426.10	6	0.02
169	444.20	6	0.02
170	447.20	6	0.02
171	466.20	6	0.02
172	480.20	12	0.04
173	488.10	20	0.07
174	496.20	7	0.02
175	544.30	6	0.02
176	595.20	14	0.05
177	602.20	6	0.02
178	669.30	6	0.02
179	671.20	7	0.02

Line#:13 R.Time:22.8(Scan#:5644)
MassPeaks:166
RawMode:Averaged 22.8-22.8(5631-5653) BasePeak:57(26856)
BG Mode:None Group 1 - Event 1

#	m/z	Abs. Int.	Rel. Int.
1	50.00	705	2.63
2	51.15	857	3.19
3	52.00	575	2.14
4	53.15	1997	7.44
5	54.15	3065	11.41
6	55.15	22270	82.92
7	56.15	10099	37.60
8	57.15	26856	100.00
9	58.15	1670	6.22
10	59.20	605	2.25
11	60.20	799	2.98
12	61.20	417	1.55
13	62.20	228	0.85
14	63.20	574	2.14
15	64.10	394	1.47
16	65.15	868	3.23
17	66.10	988	3.68
18	67.15	5513	20.53
19	68.15	3707	13.80
20	69.15	17528	65.27
21	70.15	10004	37.25
22	71.15	14726	54.83
23	72.15	938	3.49
24	73.10	1623	6.04
25	74.10	392	1.46
26	75.15	781	2.91
27	76.10	362	1.35
28	77.10	1512	5.63
29	78.10	526	1.96
30	79.15	1562	5.82

#	m/z	Abs. Int.	Rel. Int.
31	80.10	714	2.66
32	81.15	4597	17.12
33	82.15	5937	22.11
34	83.15	17472	65.06
35	84.15	5576	20.76
36	85.15	8089	30.12
37	86.20	675	2.51
38	87.20	449	1.67
39	88.20	176	0.66
40	89.20	343	1.28
41	90.20	22	0.08
42	91.15	1372	5.11
43	92.10	425	1.58
44	93.15	859	3.20
45	94.10	653	2.43
46	95.15	2452	9.13
47	96.15	4170	15.53
48	97.20	16965	63.17
49	98.20	3338	12.43
50	99.20	2356	8.77
51	100.20	360	1.34
52	101.20	360	1.34
53	102.20	185	0.69
54	103.20	259	0.96
55	104.20	114	0.42
56	105.20	755	2.81
57	106.20	232	0.86
58	107.20	530	1.97
59	108.20	385	1.43
60	109.15	1132	4.22
61	110.15	2059	7.67
62	111.15	7809	29.08
63	112.20	2029	7.56
64	113.20	1450	5.40
65	114.20	292	1.09
66	115.20	482	1.79
67	116.10	461	1.72
68	117.20	484	1.80
69	118.10	114	0.42
70	119.20	439	1.63
71	120.20	207	0.77
72	121.20	354	1.32
73	122.20	204	0.76
74	123.20	794	2.96
75	124.20	1072	3.99
76	125.20	3375	12.57
77	126.20	1216	4.53
78	127.20	831	3.09
79	128.20	293	1.09
80	129.20	520	1.94
81	130.20	97	0.36
82	131.20	375	1.40
83	132.20	88	0.33
84	133.20	396	1.47
85	134.20	74	0.28
86	135.20	262	0.98
87	136.20	348	1.30
88	137.20	321	1.20
89	138.20	638	2.38
90	139.20	1381	5.14
91	140.20	473	1.76
92	141.20	486	1.81
93	142.20	154	0.57
94	143.20	165	0.61
95	144.20	69	0.26
96	145.30	165	0.61
97	147.20	304	1.13
98	148.20	19	0.07
99	149.20	368	1.37
100	150.20	80	0.30
101	151.20	639	2.38
102	152.20	454	1.69
103	153.25	795	2.96
104	154.30	290	1.08
105	155.30	296	1.10
106	156.20	18	0.07
107	157.20	73	0.27
108	158.20	21	0.08
109	159.30	31	0.12
110	161.20	88	0.33
111	163.20	74	0.28
112	165.20	269	1.00
113	166.20	374	1.39
114	167.30	449	1.67
115	168.30	237	0.88
116	169.30	109	0.41
117	170.20	20	0.07
118	171.20	20	0.07
119	177.20	29	0.11
120	178.20	39	0.15
121	179.20	352	1.31
122	180.30	263	0.98
123	181.30	190	0.71
124	182.20	133	0.50
125	183.30	83	0.31
126	185.20	9	0.03
127	191.20	141	0.53
128	192.20	85	0.32
129	193.10	62	0.23
130	194.20	110	0.41
131	195.30	165	0.61
132	196.20	73	0.27
133	197.20	73	0.27
134	198.20	9	0.03
135	199.20	10	0.04
136	206.30	20	0.07
137	207.20	469	1.75
138	208.20	181	0.67
139	209.20	105	0.39
140	210.20	67	0.25
141	211.20	22	0.08
142	213.20	9	0.03
143	222.30	9	0.03
144	223.10	8	0.03
145	224.20	92	0.34
146	225.10	63	0.23
147	226.20	102	0.38
148	227.10	10	0.04
149	228.20	422	1.57
150	229.10	31	0.12
151	236.30	22	0.08
152	237.20	8	0.03
153	238.30	9	0.03
154	239.20	83	0.31
155	249.20	9	0.03
156	250.30	20	0.07
157	256.20	9	0.03
158	267.20	9	0.03
159	281.20	110	0.41
160	306.20	8	0.03
161	354.20	8	0.03
162	356.20	8	0.03
163	426.30	8	0.03
164	459.20	8	0.03
165	510.20	9	0.03
166	697.20	9	0.03

Line#:14 R.Time:23.7(Scan#:5906)
MassPeaks:171
RawMode:Averaged 23.6-23.7(5893-5915) BasePeak:57(50761)
BG Mode:None Group 1 - Event 1

#	m/z	Abs. Int.	Rel. Int.
1	50.00	660	1.30
2	51.00	750	1.48
3	52.00	506	1.00
4	53.15	1450	2.86
5	54.15	1538	3.03
6	55.15	13335	26.27
7	56.15	6679	13.16
8	57.15	50761	100.00
9	58.15	2756	5.43
10	59.10	745	1.47
11	60.20	753	1.48
12	61.20	412	0.81
13	62.20	210	0.41
14	63.20	472	0.93
15	64.20	360	0.71
16	65.20	693	1.37
17	66.10	604	1.19
18	67.10	2879	5.67
19	68.15	1584	3.12
20	69.15	8523	16.79
21	70.15	5530	10.89
22	71.15	34902	68.76
23	72.15	2072	4.08
24	73.10	1740	3.43
25	74.10	452	0.89
26	75.15	949	1.87
27	76.10	361	0.71
28	77.10	1487	2.93
29	78.10	527	1.04
30	79.15	1264	2.49
31	80.10	500	0.99
32	81.15	2309	4.55
33	82.15	2299	4.53
34	83.15	6242	12.30
35	84.15	3114	6.13
36	85.15	22678	44.68
37	86.15	1543	3.04
38	87.20	426	0.84
39	88.20	140	0.28
40	89.20	450	0.89
41	90.20	8	0.02
42	91.10	1164	2.29
43	92.10	385	0.76
44	93.10	707	1.39
45	94.10	537	1.06
46	95.10	1482	2.92
47	96.15	1693	3.34
48	97.15	5291	10.42
49	98.20	2161	4.26
50	99.20	8853	17.44
51	100.15	900	1.77
52	101.10	469	0.92
53	102.10	113	0.22
54	103.10	352	0.69
55	104.20	98	0.19
56	105.20	640	1.26
57	106.20	230	0.45
58	107.20	536	1.06
59	108.20	287	0.57
60	109.20	723	1.42
61	110.20	1346	2.65
62	111.20	2766	5.45
63	112.20	1287	2.54
64	113.20	4991	9.83
65	114.20	454	0.89
66	115.20	537	1.06
67	116.20	583	1.15
68	117.20	553	1.09
69	118.10	130	0.26
70	119.10	463	0.91
71	120.20	133	0.26
72	121.20	414	0.82
73	122.20	185	0.36
74	123.20	513	1.01
75	124.15	554	1.09
76	125.20	1361	2.68
77	126.20	822	1.62
78	127.20	3023	5.96
79	128.20	534	1.05
80	129.10	617	1.22
81	130.20	129	0.25
82	131.20	341	0.67
83	132.10	31	0.06
84	133.20	466	0.92

DEPTT. OF BOTANICAL & ENVIRONMENTAL SCIENCES, G.N.D.U. AMRITSAR

#	m/z	Abs. Int.	Rel. Int.	#	m/z	Abs. Int.	Rel. Int.	#	m/z	Abs. Int.	Rel. Int.
85	134.20	176	0.35	114	165.20	249	0.49	143	205.20	20	0.04
86	135.20	304	0.60	115	166.20	288	0.57	144	207.30	570	1.12
87	136.20	330	0.65	116	167.30	318	0.63	145	208.20	124	0.24
88	137.20	297	0.59	117	168.20	235	0.46	146	209.10	30	0.06
89	138.20	291	0.57	118	169.30	714	1.41	147	210.30	137	0.27
90	139.20	540	1.06	119	170.30	121	0.24	148	211.30	244	0.48
91	140.25	572	1.13	120	171.20	19	0.04	149	213.20	17	0.03
92	141.25	1750	3.45	121	173.30	42	0.08	150	215.10	29	0.06
93	142.20	258	0.51	122	175.20	29	0.06	151	217.30	18	0.04
94	143.20	132	0.26	123	176.20	19	0.04	152	222.30	19	0.04
95	144.20	50	0.10	124	177.30	64	0.13	153	223.30	37	0.07
96	145.20	134	0.26	125	178.30	9	0.02	154	224.30	52	0.10
97	147.20	313	0.62	126	179.20	289	0.57	155	225.30	180	0.35
98	148.20	59	0.12	127	180.20	95	0.19	156	227.20	8	0.02
99	149.10	378	0.74	128	181.30	58	0.11	157	238.30	55	0.11
100	150.20	89	0.18	129	182.30	198	0.39	158	239.30	92	0.18
101	151.20	562	1.11	130	183.25	469	0.92	159	240.20	9	0.02
102	152.20	232	0.46	131	184.30	70	0.14	160	252.30	19	0.04
103	153.20	274	0.54	132	185.30	18	0.04	161	253.30	94	0.19
104	154.20	323	0.64	133	187.30	27	0.05	162	266.30	29	0.06
105	155.25	998	1.97	134	189.30	9	0.02	163	267.30	69	0.14
106	156.20	145	0.29	135	191.30	114	0.22	164	281.30	219	0.43
107	157.30	112	0.22	136	192.20	53	0.10	165	309.30	8	0.02
108	158.20	19	0.04	137	193.20	38	0.07	166	368.10	9	0.02
109	159.20	27	0.05	138	195.30	9	0.02	167	402.30	8	0.02
110	160.10	9	0.02	139	196.30	130	0.26	168	504.20	9	0.02
111	162.20	39	0.08	140	197.30	347	0.68	169	506.30	9	0.02
112	163.20	132	0.26	141	198.30	65	0.13	170	527.20	9	0.02
113	164.20	8	0.02	142	199.20	9	0.02	171	558.10	8	0.02

Line#:15 R.Time:24.4(Scan#:6126)
MassPeaks:202
RawMode:Averaged 24.4-24.5(6113-6146) BasePeak:57(24940)
BG Mode:None Group 1 - Event 1

#	m/z	Abs. Int.	Rel. Int.	#	m/z	Abs. Int.	Rel. Int.	#	m/z	Abs. Int.	Rel. Int.
1	50.00	668	2.68	46	95.15	1843	7.39	91	140.25	492	1.97
2	51.10	799	3.20	47	96.15	2851	11.43	92	141.20	673	2.70
3	52.10	549	2.20	48	97.15	10075	40.40	93	142.20	84	0.34
4	53.15	1407	5.64	49	98.15	2194	8.80	94	143.20	133	0.53
5	54.15	1905	7.64	50	99.20	3255	13.05	95	144.20	27	0.11
6	55.15	14391	57.70	51	100.20	414	1.66	96	145.20	257	1.03
7	56.15	6389	25.62	52	101.20	366	1.47	97	146.20	13	0.05
8	57.15	24940	100.00	53	102.20	203	0.81	98	147.20	282	1.13
9	58.20	1554	6.23	54	103.10	330	1.32	99	148.20	7	0.03
10	59.15	625	2.51	55	104.20	124	0.50	100	149.20	308	1.23
11	60.20	692	2.77	56	105.20	662	2.65	101	150.20	75	0.30
12	61.20	395	1.58	57	106.20	267	1.07	102	151.20	558	2.24
13	62.20	239	0.96	58	107.20	493	1.98	103	152.20	370	1.48
14	63.10	515	2.06	59	108.20	299	1.20	104	153.25	512	2.05
15	64.20	333	1.34	60	109.15	898	3.60	105	154.20	265	1.06
16	65.20	685	2.75	61	110.15	1505	6.03	106	155.25	391	1.57
17	66.10	726	2.91	62	111.20	4966	19.91	107	156.20	55	0.22
18	67.15	3699	14.83	63	112.20	1323	5.30	108	157.20	96	0.38
19	68.15	2423	9.72	64	113.20	1782	7.15	109	158.20	13	0.05
20	69.15	10702	42.91	65	114.20	241	0.97	110	159.20	59	0.24
21	70.15	6151	24.66	66	115.20	564	2.26	111	160.20	18	0.07
22	71.15	14282	57.27	67	116.20	543	2.18	112	161.20	39	0.16
23	72.15	939	3.77	68	117.20	480	1.92	113	162.20	18	0.07
24	73.10	1712	6.86	69	118.20	157	0.63	114	163.20	134	0.54
25	74.10	419	1.68	70	119.20	447	1.79	115	164.20	18	0.07
26	75.10	914	3.66	71	120.20	99	0.40	116	165.20	283	1.13
27	76.10	406	1.63	72	121.20	374	1.50	117	166.20	402	1.61
28	77.10	1482	5.94	73	122.20	163	0.65	118	167.20	349	1.40
29	78.10	513	2.06	74	123.15	602	2.41	119	168.20	196	0.79
30	79.15	1265	5.07	75	124.25	830	3.33	120	169.25	252	1.01
31	80.15	530	2.13	76	125.20	2258	9.05	121	170.20	36	0.14
32	81.15	3076	12.33	77	126.20	781	3.13	122	171.20	33	0.13
33	82.15	3887	15.59	78	127.20	1126	4.51	123	172.20	6	0.02
34	83.15	10496	42.09	79	128.20	333	1.34	124	173.20	6	0.02
35	84.15	3315	13.29	80	129.20	578	2.32	125	174.20	46	0.18
36	85.15	8783	35.22	81	130.20	135	0.54	126	175.20	54	0.22
37	86.15	732	2.94	82	131.20	306	1.23	127	177.20	62	0.25
38	87.10	377	1.51	83	132.20	98	0.39	128	178.20	6	0.02
39	88.10	68	0.27	84	133.20	470	1.88	129	179.20	355	1.42
40	89.10	358	1.44	85	134.20	75	0.30	130	180.20	197	0.79
41	90.10	44	0.18	86	135.20	314	1.26	131	181.20	174	0.70
42	91.10	1063	4.26	87	136.20	310	1.24	132	182.20	105	0.42
43	92.10	348	1.40	88	137.20	315	1.26	133	183.20	142	0.57
44	93.20	692	2.77	89	138.15	406	1.63	134	184.30	6	0.02
45	94.10	548	2.20	90	139.25	942	3.78	135	185.20	27	0.11

DEPTT. OF BOTANICAL & ENVIRONMENTAL SCIENCES, G.N.D.U. AMRITSAR

#	m/z	Abs. Int.	Rel. Int.	#	m/z	Abs. Int.	Rel. Int.	#	m/z	Abs. Int.	Rel. Int.
136	187.20	13	0.05	159	226.20	6	0.02	182	343.20	6	0.02
137	189.20	37	0.15	160	227.20	19	0.08	183	352.30	119	0.48
138	191.20	242	0.97	161	233.20	12	0.05	184	353.20	33	0.13
139	192.20	87	0.35	162	236.20	6	0.02	185	364.20	12	0.05
140	193.20	171	0.69	163	237.20	32	0.13	186	396.20	5	0.02
141	194.20	109	0.44	164	239.20	21	0.08	187	400.20	6	0.02
142	195.20	73	0.29	165	249.20	11	0.04	188	403.20	6	0.02
143	196.20	63	0.25	166	251.20	16	0.06	189	424.20	5	0.02
144	197.20	120	0.48	167	252.20	18	0.07	190	427.20	6	0.02
145	205.20	27	0.11	168	253.20	6	0.02	191	429.20	5	0.02
146	207.20	803	3.22	169	254.20	7	0.03	192	434.20	5	0.02
147	208.20	299	1.20	170	265.20	42	0.17	193	457.20	5	0.02
148	209.20	171	0.69	171	267.20	61	0.24	194	483.20	5	0.02
149	210.20	31	0.12	172	272.20	119	0.48	195	501.20	6	0.02
150	211.30	213	0.85	173	278.20	6	0.02	196	566.20	13	0.05
151	213.30	12	0.05	174	279.20	6	0.02	197	570.20	6	0.02
152	217.20	20	0.08	175	280.20	6	0.02	198	572.30	6	0.02
153	220.20	5	0.02	176	281.20	278	1.11	199	586.20	12	0.05
154	221.30	12	0.05	177	282.20	29	0.12	200	611.20	12	0.05
155	222.20	13	0.05	178	318.20	6	0.02	201	680.20	6	0.02
156	223.20	81	0.32	179	336.20	7	0.03	202	697.20	6	0.02
157	224.20	6	0.02	180	337.30	239	0.96				
158	225.20	81	0.32	181	338.20	68	0.27				

Line#:16 R.Time:25.4(Scan#:6422)
MassPeaks:247
RawMode:Averaged 25.3-25.5(6402-6449) BasePeak:57(195962)
BG Mode:None Group 1 - Event 1

#	m/z	Abs. Int.	Rel. Int.	#	m/z	Abs. Int.	Rel. Int.	#	m/z	Abs. Int.	Rel. Int.
1	50.00	698	0.36	52	101.20	464	0.24	103	152.20	773	0.39
2	51.15	810	0.41	53	102.10	106	0.05	104	153.20	1252	0.64
3	52.10	554	0.28	54	103.10	374	0.19	105	154.25	1678	0.86
4	53.15	2450	1.25	55	104.20	178	0.09	106	155.25	6548	3.34
5	54.15	4116	2.10	56	105.15	735	0.38	107	156.25	767	0.39
6	55.15	42760	21.82	57	106.20	250	0.13	108	157.30	123	0.06
7	56.15	23650	12.07	58	107.10	647	0.33	109	158.10	4	0.00
8	57.15	195962	100.00	59	108.20	335	0.17	110	159.10	118	0.06
9	58.15	9370	4.78	60	109.15	1064	0.54	111	160.20	10	0.01
10	59.15	1342	0.68	61	110.15	3098	1.58	112	161.10	73	0.04
11	60.10	659	0.34	62	111.15	10215	5.21	113	162.10	9	0.00
12	61.20	350	0.18	63	112.15	4962	2.53	114	163.20	123	0.06
13	62.10	274	0.14	64	113.20	22966	11.72	115	164.30	33	0.02
14	63.20	473	0.24	65	114.20	2125	1.08	116	165.10	286	0.15
15	64.10	347	0.18	66	115.20	588	0.30	117	166.20	605	0.31
16	65.10	894	0.46	67	116.20	511	0.26	118	167.25	740	0.38
17	66.10	903	0.46	68	117.20	571	0.29	119	168.30	1191	0.61
18	67.10	6041	3.08	69	118.20	146	0.07	120	169.30	4538	2.32
19	68.10	4576	2.34	70	119.20	585	0.30	121	170.25	573	0.29
20	69.15	25987	13.26	71	120.10	258	0.13	122	171.30	97	0.05
21	70.15	17869	9.12	72	121.20	479	0.24	123	173.30	60	0.03
22	71.15	127257	64.94	73	122.20	266	0.14	124	174.30	8	0.00
23	72.15	6956	3.55	74	123.15	745	0.38	125	175.20	49	0.03
24	73.15	2084	1.06	75	124.20	1835	0.94	126	176.10	29	0.01
25	74.10	465	0.24	76	125.20	5419	2.77	127	177.20	137	0.07
26	75.10	924	0.47	77	126.20	3648	1.86	128	178.20	52	0.03
27	76.10	338	0.17	78	127.20	15120	7.72	129	179.20	365	0.19
28	77.05	1580	0.81	79	128.25	1680	0.86	130	180.20	345	0.18
29	78.10	556	0.28	80	129.20	671	0.34	131	181.25	375	0.19
30	79.10	1730	0.88	81	130.20	159	0.08	132	182.25	869	0.44
31	80.15	669	0.34	82	131.20	380	0.19	133	183.25	3132	1.60
32	81.15	4100	2.09	83	132.20	75	0.04	134	184.25	557	0.28
33	82.15	6903	3.52	84	133.20	574	0.29	135	185.20	69	0.04
34	83.15	20619	10.52	85	134.20	252	0.13	136	187.30	14	0.01
35	84.15	10297	5.25	86	135.20	327	0.17	137	188.30	4	0.00
36	85.15	87834	44.82	87	136.20	384	0.20	138	189.10	65	0.03
37	86.15	5886	3.00	88	137.20	370	0.19	139	190.10	19	0.01
38	87.20	525	0.27	89	138.20	1071	0.55	140	191.20	332	0.17
39	88.20	127	0.06	90	139.20	2309	1.18	141	192.20	57	0.03
40	89.20	363	0.19	91	140.20	2424	1.24	142	193.20	202	0.10
41	90.20	26	0.01	92	141.20	9956	5.08	143	194.25	188	0.10
42	91.10	1293	0.66	93	142.20	1130	0.58	144	195.25	287	0.15
43	92.10	400	0.20	94	143.20	207	0.11	145	196.25	677	0.35
44	93.10	772	0.39	95	144.20	49	0.03	146	197.25	2298	1.17
45	94.10	626	0.32	96	145.20	266	0.14	147	198.25	327	0.17
46	95.15	2308	1.18	97	146.20	61	0.03	148	199.30	23	0.01
47	96.15	5050	2.58	98	147.20	377	0.19	149	201.20	13	0.01
48	97.15	19012	9.70	99	148.20	55	0.03	150	203.20	18	0.01
49	98.15	6822	3.48	100	149.10	353	0.18	151	204.10	8	0.00
50	99.20	36337	18.54	101	150.20	120	0.06	152	205.10	28	0.01
51	100.20	2932	1.50	102	151.20	657	0.34	153	207.05	1071	0.55

DEPTT. OF BOTANICAL & ENVIRONMENTAL SCIENCES, G.N.D.U. AMRITSAR

#	m/z	Abs. Int.	Rel. Int.	#	m/z	Abs. Int.	Rel. Int.	#	m/z	Abs. Int.	Rel. Int.
154	208.10	411	0.21	186	258.10	4	0.00	218	341.10	5	0.00
155	209.10	299	0.15	187	259.30	4	0.00	219	349.10	4	0.00
156	210.25	580	0.30	188	260.20	4	0.00	220	351.30	63	0.03
157	211.25	1667	0.85	189	264.30	5	0.00	221	355.30	9	0.00
158	212.25	304	0.16	190	265.30	63	0.03	222	356.20	10	0.01
159	213.30	15	0.01	191	266.30	203	0.10	223	370.10	4	0.00
160	217.30	9	0.00	192	267.30	542	0.28	224	380.40	74	0.04
161	220.10	18	0.01	193	268.30	137	0.07	225	388.20	4	0.00
162	221.30	9	0.00	194	275.30	4	0.00	226	393.20	5	0.00
163	222.30	96	0.05	195	280.35	152	0.08	227	403.20	4	0.00
164	223.30	134	0.07	196	281.30	738	0.38	228	408.20	4	0.00
165	224.25	456	0.23	197	282.30	147	0.08	229	430.40	4	0.00
166	225.25	1301	0.66	198	283.30	8	0.00	230	445.20	4	0.00
167	226.25	228	0.12	199	288.10	4	0.00	231	446.10	4	0.00
168	227.30	9	0.00	200	294.30	116	0.06	232	461.20	4	0.00
169	232.30	4	0.00	201	295.35	271	0.14	233	462.30	4	0.00
170	236.30	40	0.02	202	296.30	55	0.03	234	469.20	4	0.00
171	237.30	55	0.03	203	297.30	9	0.00	235	480.10	4	0.00
172	238.30	374	0.19	204	304.20	4	0.00	236	560.30	4	0.00
173	239.25	938	0.48	205	308.30	82	0.04	237	563.10	9	0.00
174	240.30	181	0.09	206	309.40	213	0.11	238	576.30	8	0.00
175	241.10	14	0.01	207	310.30	41	0.02	239	582.20	4	0.00
176	243.20	4	0.00	208	311.30	8	0.00	240	606.20	9	0.00
177	246.10	4	0.00	209	313.30	4	0.00	241	609.10	4	0.00
178	249.20	23	0.01	210	322.40	74	0.04	242	611.30	9	0.00
179	250.30	119	0.06	211	323.30	185	0.09	243	627.10	4	0.00
180	251.20	54	0.03	212	324.30	41	0.02	244	637.20	5	0.00
181	252.25	568	0.29	213	326.20	4	0.00	245	648.10	4	0.00
182	253.30	768	0.39	214	331.40	4	0.00	246	650.40	4	0.00
183	254.30	108	0.06	215	336.40	42	0.02	247	660.10	4	0.00
184	255.30	4	0.00	216	337.40	115	0.06				
185	257.30	4	0.00	217	338.40	19	0.01				

Line#:17 R.Time:26.4(Scan#:6713)
MassPeaks:187
RawMode:Averaged 26.3-26.4(6694-6728) BasePeak:57(12448)
BG Mode:None Group 1 - Event 1

#	m/z	Abs. Int.	Rel. Int.	#	m/z	Abs. Int.	Rel. Int.	#	m/z	Abs. Int.	Rel. Int.
1	50.00	650	5.22	43	92.20	323	2.59	85	134.20	219	1.76
2	51.00	662	5.32	44	93.10	699	5.62	86	135.20	387	3.11
3	52.00	465	3.74	45	94.10	535	4.30	87	136.20	302	2.43
4	53.15	1144	9.19	46	95.15	1636	13.14	88	137.20	271	2.18
5	54.20	1295	10.40	47	96.15	2149	17.26	89	138.20	378	3.04
6	55.15	8992	72.24	48	97.15	6668	53.57	90	139.20	675	5.42
7	56.15	3891	31.26	49	98.20	1370	11.01	91	140.20	227	1.82
8	57.15	12448	100.00	50	99.20	1634	13.13	92	141.20	415	3.33
9	58.20	880	7.07	51	100.20	175	1.41	93	142.20	60	0.48
10	59.15	568	4.56	52	101.20	273	2.19	94	143.20	120	0.96
11	60.20	574	4.61	53	102.10	100	0.80	95	144.20	12	0.10
12	61.10	412	3.31	54	103.20	404	3.25	96	145.20	203	1.63
13	62.20	176	1.41	55	104.20	129	1.04	97	146.20	24	0.19
14	63.20	438	3.52	56	105.20	661	5.31	98	147.20	405	3.25
15	64.20	294	2.36	57	106.10	219	1.76	99	148.20	55	0.44
16	65.20	628	5.04	58	107.20	662	5.32	100	149.10	367	2.95
17	66.10	528	4.24	59	108.20	350	2.81	101	150.20	111	0.89
18	67.10	2685	21.57	60	109.15	1111	8.93	102	151.20	527	4.23
19	68.20	1586	12.74	61	110.15	1086	8.72	103	152.20	283	2.27
20	69.20	7309	58.72	62	111.20	3455	27.76	104	153.25	402	3.23
21	70.15	3793	30.47	63	112.15	894	7.18	105	154.20	186	1.49
22	71.15	7556	60.70	64	113.20	971	7.80	106	155.20	271	2.18
23	72.20	583	4.68	65	114.20	126	1.01	107	157.20	26	0.21
24	73.10	1692	13.59	66	115.20	445	3.57	108	158.20	24	0.19
25	74.10	386	3.10	67	116.10	377	3.03	109	159.30	89	0.71
26	75.10	892	7.17	68	117.20	569	4.57	110	160.20	18	0.14
27	76.10	318	2.55	69	118.10	154	1.24	111	161.20	49	0.39
28	77.10	1375	11.05	70	119.20	571	4.59	112	162.20	5	0.04
29	78.10	528	4.24	71	120.20	151	1.21	113	163.20	163	1.31
30	79.10	1230	9.88	72	121.20	532	4.27	114	164.20	19	0.15
31	80.10	482	3.87	73	122.10	238	1.91	115	165.20	222	1.78
32	81.15	2541	20.41	74	123.15	936	7.52	116	166.20	267	2.14
33	82.15	2807	22.55	75	124.15	654	5.25	117	167.20	303	2.43
34	83.20	6679	53.66	76	125.25	1574	12.64	118	168.30	112	0.90
35	84.20	2218	17.82	77	126.25	481	3.86	119	169.30	151	1.21
36	85.20	4502	36.17	78	127.20	616	4.95	120	170.20	6	0.05
37	86.20	405	3.25	79	128.20	269	2.16	121	171.10	19	0.15
38	87.20	320	2.57	80	129.20	532	4.27	122	173.20	20	0.16
39	88.20	84	0.67	81	130.20	186	1.49	123	174.20	6	0.05
40	89.20	390	3.13	82	131.20	350	2.81	124	175.20	80	0.64
41	90.20	46	0.37	83	132.20	65	0.52	125	176.20	6	0.05
42	91.10	1106	8.88	84	133.20	530	4.26	126	177.20	71	0.57

DEPTT. OF BOTANICAL & ENVIRONMENTAL SCIENCES, G.N.D.U. AMRITSAR

#	m/z	Abs. Int.	Rel. Int.
127	178.10	5	0.04
128	179.20	276	2.22
129	180.20	78	0.63
130	181.30	96	0.77
131	182.20	73	0.59
132	183.20	43	0.35
133	184.20	6	0.05
134	185.20	20	0.16
135	187.20	11	0.09
136	189.20	11	0.09
137	190.20	6	0.05
138	191.20	306	2.46
139	192.20	51	0.41
140	193.20	255	2.05
141	194.20	57	0.46
142	195.20	67	0.54
143	196.30	19	0.15
144	197.10	22	0.18
145	198.20	6	0.05
146	201.20	6	0.05
147	203.30	12	0.10
148	204.20	12	0.10
149	205.30	18	0.14
150	207.10	928	7.46
151	208.10	292	2.35
152	209.20	239	1.92
153	210.10	6	0.05
154	211.10	13	0.10
155	215.20	6	0.05
156	221.10	12	0.10
157	222.20	13	0.10
158	223.10	12	0.10
159	224.30	6	0.05
160	233.30	5	0.04
161	236.20	6	0.05
162	246.20	6	0.05
163	249.20	5	0.04
164	250.20	18	0.14
165	251.20	6	0.05
166	255.20	6	0.05
167	265.10	25	0.20
168	267.10	21	0.17
169	281.20	307	2.47
170	283.10	19	0.15
171	293.20	12	0.10
172	294.10	6	0.05
173	318.20	5	0.04
174	356.20	5	0.04
175	375.20	5	0.04
176	381.20	12	0.10
177	404.20	5	0.04
178	471.20	5	0.04
179	504.20	6	0.05
180	512.10	5	0.04
181	533.20	5	0.04
182	565.20	5	0.04
183	577.20	6	0.05
184	600.10	5	0.04
185	661.10	6	0.05
186	664.30	6	0.05
187	699.10	13	0.10

Line#:18 R.Time:26.6(Scan#:6773)
MassPeaks:211
RawMode:Averaged 26.5-26.6(6752-6793) BasePeak:69(74549)
BG Mode:None Group 1 - Event 1

#	m/z	Abs. Int.	Rel. Int.
1	50.10	753	1.01
2	51.10	1003	1.35
3	52.15	692	0.93
4	53.15	4058	5.44
5	54.15	857	1.15
6	55.15	7749	10.39
7	56.15	1268	1.70
8	57.15	5450	7.31
9	58.10	564	0.76
10	59.20	501	0.67
11	60.20	550	0.74
12	61.20	381	0.51
13	62.10	231	0.31
14	63.10	550	0.74
15	64.10	366	0.49
16	65.10	1419	1.90
17	66.10	760	1.02
18	67.10	9689	13.00
19	68.15	8417	11.29
20	69.15	74549	100.00
21	70.15	4919	6.60
22	71.15	3135	4.21
23	72.10	331	0.44
24	73.15	1719	2.31
25	74.10	427	0.57
26	75.10	785	1.05
27	76.10	329	0.44
28	77.10	3076	4.13
29	78.15	881	1.18
30	79.15	5092	6.83
31	80.15	2000	2.68
32	81.15	42217	56.63
33	82.15	4841	6.49
34	83.15	3026	4.06
35	84.20	677	0.91
36	85.20	1572	2.11
37	86.10	176	0.24
38	87.20	306	0.41
39	88.20	68	0.09
40	89.10	353	0.47
41	90.10	36	0.05
42	91.10	3680	4.94
43	92.15	1476	1.98
44	93.15	7364	9.88
45	94.15	2801	3.76
46	95.15	13463	18.06
47	96.15	1790	2.40
48	97.20	1781	2.39
49	98.20	503	0.67
50	99.20	620	0.83
51	100.20	79	0.11
52	101.10	236	0.32
53	102.20	89	0.12
54	103.20	523	0.70
55	104.20	263	0.35
56	105.15	2518	3.38
57	106.15	792	1.06
58	107.15	5551	7.45
59	108.15	1557	2.09
60	109.20	5120	6.87
61	110.20	1097	1.47
62	111.15	1007	1.35
63	112.20	301	0.40
64	113.20	325	0.44
65	114.10	40	0.05
66	115.20	550	0.74
67	116.10	394	0.53
68	117.20	603	0.81
69	118.20	164	0.22
70	119.15	2468	3.31
71	120.20	770	1.03
72	121.20	6855	9.20
73	122.20	1488	2.00
74	123.20	4124	5.53
75	124.25	640	0.86
76	125.20	401	0.54
77	126.20	202	0.27
78	127.20	352	0.47
79	128.20	266	0.36
80	129.20	530	0.71
81	130.20	152	0.20
82	131.15	504	0.68
83	132.20	166	0.22
84	133.15	1751	2.35
85	134.20	1109	1.49
86	135.20	3127	4.19
87	136.20	4094	5.49
88	137.20	3639	4.88
89	138.20	507	0.68
90	139.20	135	0.18
91	140.20	40	0.05
92	141.20	253	0.34
93	142.20	68	0.09
94	143.10	174	0.23
95	144.20	56	0.08
96	145.20	567	0.76
97	146.20	104	0.14
98	147.20	1825	2.45
99	148.20	603	0.81
100	149.25	2978	3.99
101	150.25	531	0.71
102	151.20	616	0.83
103	152.20	171	0.23
104	153.20	150	0.20
105	154.20	15	0.02
106	155.20	31	0.04
107	156.20	5	0.01
108	157.20	96	0.13
109	158.20	17	0.02
110	159.20	339	0.45
111	160.20	127	0.17
112	161.25	921	1.24
113	162.25	354	0.47
114	163.25	828	1.11
115	164.20	152	0.20
116	165.20	287	0.38
117	166.20	234	0.31
118	167.20	101	0.14
119	168.20	10	0.01
120	169.20	57	0.08
121	171.20	62	0.08
122	173.20	259	0.35
123	174.20	82	0.11
124	175.25	800	1.07
125	176.20	258	0.35
126	177.25	611	0.82
127	178.30	125	0.17
128	179.20	290	0.39
129	180.20	51	0.07
130	182.20	10	0.01
131	183.30	19	0.03
132	184.20	4	0.01
133	185.30	40	0.05
134	186.20	4	0.01
135	187.30	227	0.30
136	188.20	62	0.08
137	189.25	570	0.76
138	190.20	209	0.28
139	191.20	754	1.01
140	192.30	369	0.49
141	193.30	289	0.39
142	195.20	20	0.03
143	199.30	6	0.01
144	201.30	110	0.15
145	202.20	53	0.07
146	203.20	588	0.79
147	204.20	180	0.24
148	205.20	210	0.28
149	206.10	45	0.06
150	207.05	1056	1.42
151	208.10	290	0.39
152	209.10	136	0.18
153	213.10	39	0.05
154	215.10	129	0.17
155	216.10	97	0.13
156	217.10	241	0.32
157	218.10	72	0.10
158	219.10	25	0.03
159	220.10	10	0.01

DEPTT. OF BOTANICAL & ENVIRONMENTAL SCIENCES, G.N.D.U. AMRITSAR

#	m/z	Abs. Int.	Rel. Int.
160	221.20	12	0.02
161	223.20	15	0.02
162	224.10	4	0.01
163	225.20	10	0.01
164	229.10	117	0.16
165	230.10	35	0.05
166	231.10	207	0.28
167	232.10	17	0.02
168	241.10	15	0.02
169	243.10	65	0.09
170	244.10	4	0.01
171	245.10	26	0.03
172	248.10	4	0.01
173	249.10	36	0.05
174	250.10	5	0.01
175	251.20	32	0.04
176	255.10	5	0.01
177	257.10	73	0.10
178	258.10	10	0.01
179	259.10	47	0.06
180	260.10	5	0.01
181	265.10	52	0.07
182	267.20	71	0.10
183	269.10	10	0.01
184	271.10	22	0.03
185	272.20	4	0.01
186	273.10	43	0.06
187	274.10	4	0.01
188	281.10	372	0.50
189	282.10	48	0.06
190	285.10	4	0.01
191	289.10	5	0.01
192	297.10	5	0.01
193	299.10	24	0.03
194	300.10	11	0.01
195	324.10	5	0.01
196	327.10	10	0.01
197	329.20	5	0.01
198	341.10	93	0.12
199	356.20	4	0.01
200	367.10	40	0.05
201	368.10	15	0.02
202	408.10	4	0.01
203	491.10	4	0.01
204	495.20	4	0.01
205	512.10	4	0.01
206	606.10	10	0.01
207	619.00	5	0.01
208	623.10	4	0.01
209	663.10	9	0.01
210	665.10	5	0.01
211	683.10	4	0.01

Line#:19 R.Time:27.5(Scan#:7056)
MassPeaks:194
RawMode:Averaged 27.4-27.6(7035-7086) BasePeak:57(15378)
BG Mode:None Group 1 - Event 1

#	m/z	Abs. Int.	Rel. Int.
1	50.00	628	4.08
2	51.00	637	4.14
3	52.00	475	3.09
4	53.10	1154	7.50
5	54.15	1456	9.47
6	55.15	11852	77.07
7	56.15	4896	31.84
8	57.15	15378	100.00
9	58.15	991	6.44
10	59.10	462	3.00
11	60.20	521	3.39
12	61.10	339	2.20
13	62.10	198	1.29
14	63.10	427	2.78
15	64.10	308	2.00
16	65.10	630	4.10
17	66.10	808	5.25
18	67.15	4064	26.43
19	68.15	3985	25.91
20	69.15	10333	67.19
21	70.15	4395	28.58
22	71.15	8094	52.63
23	72.15	638	4.15
24	73.15	1969	12.80
25	74.10	383	2.49
26	75.10	677	4.40
27	76.10	325	2.11
28	77.10	1380	8.97
29	78.10	496	3.23
30	79.15	1409	9.16
31	80.15	618	4.02
32	81.15	3997	25.99
33	82.15	6029	39.21
34	83.15	9719	63.20
35	84.15	2626	17.08
36	85.15	4705	30.60
37	86.15	425	2.76
38	87.20	313	2.04
39	88.20	36	0.23
40	89.20	312	2.03
41	90.20	16	0.10
42	91.10	1177	7.65
43	92.10	337	2.19
44	93.10	894	5.81
45	94.15	614	3.99
46	95.15	2626	17.08
47	96.15	3953	25.71
48	97.15	9223	59.98
49	98.20	1766	11.48
50	99.20	1578	10.26
51	100.20	190	1.24
52	101.20	198	1.29
53	102.20	50	0.33
54	103.20	333	2.17
55	104.20	101	0.66
56	105.20	673	4.38
57	106.20	188	1.22
58	107.20	650	4.23
59	108.20	332	2.16
60	109.15	1271	8.27
61	110.15	1636	10.64
62	111.20	4416	28.72
63	112.15	1037	6.74
64	113.20	912	5.93
65	114.20	111	0.72
66	115.20	444	2.89
67	116.20	305	1.98
68	117.10	454	2.95
69	118.20	71	0.46
70	119.10	570	3.71
71	120.20	174	1.13
72	121.20	504	3.28
73	122.20	241	1.57
74	123.20	897	5.83
75	124.20	1004	6.53
76	125.20	2153	14.00
77	126.25	626	4.07
78	127.25	543	3.53
79	128.20	206	1.34
80	129.30	441	2.87
81	130.20	48	0.31
82	131.20	301	1.96
83	132.20	31	0.20
84	133.10	567	3.69
85	134.20	151	0.98
86	135.20	359	2.33
87	136.20	329	2.14
88	137.20	469	3.05
89	138.20	614	3.99
90	139.20	883	5.74
91	140.20	322	2.09
92	141.20	347	2.26
93	142.20	60	0.39
94	143.20	84	0.55
95	145.20	186	1.21
96	146.10	4	0.03
97	147.20	386	2.51
98	148.20	46	0.30
99	149.20	301	1.96
100	150.20	70	0.46
101	151.20	507	3.30
102	152.20	384	2.50
103	153.25	491	3.19
104	154.20	214	1.39
105	155.20	228	1.48
106	156.30	8	0.05
107	157.20	75	0.49
108	158.20	20	0.13
109	159.20	66	0.43
110	160.30	4	0.03
111	161.20	106	0.69
112	162.20	17	0.11
113	163.10	159	1.03
114	164.10	35	0.23
115	165.20	299	1.94
116	166.20	330	2.15
117	167.30	370	2.41
118	168.20	133	0.86
119	169.20	96	0.62
120	171.20	17	0.11
121	173.20	28	0.18
122	175.20	37	0.24
123	176.20	3	0.02
124	177.20	150	0.98
125	178.20	8	0.05
126	179.30	271	1.76
127	180.20	189	1.23
128	181.20	168	1.09
129	182.20	43	0.28
130	183.20	33	0.21
131	184.20	9	0.06
132	185.20	19	0.12
133	186.20	8	0.05
134	187.20	8	0.05
135	188.20	8	0.05
136	189.20	18	0.12
137	191.10	337	2.19
138	192.20	58	0.38
139	193.10	248	1.61
140	194.20	93	0.60
141	195.20	76	0.49
142	196.20	25	0.16
143	197.20	28	0.18
144	199.20	18	0.12
145	201.20	4	0.03
146	203.20	12	0.08
147	204.20	12	0.08
148	205.20	16	0.10
149	207.10	968	6.29
150	208.30	268	1.74
151	209.20	186	1.21
152	210.10	34	0.22
153	211.10	11	0.07
154	213.20	3	0.02
155	217.10	4	0.03
156	218.10	4	0.03
157	219.10	8	0.05
158	220.20	8	0.05
159	221.20	35	0.23
160	222.10	9	0.06
161	223.30	29	0.19
162	225.10	4	0.03
163	229.20	13	0.08
164	231.20	4	0.03
165	236.20	4	0.03
166	237.30	4	0.03
167	249.10	26	0.17
168	250.10	4	0.03

DEPTT. OF BOTANICAL & ENVIRONMENTAL SCIENCES, G.N.D.U. AMRITSAR

#	m/z	Abs. Int.	Rel. Int.
169	251.10	3	0.02
170	253.20	3	0.02
171	265.10	20	0.13
172	267.10	45	0.29
173	276.30	4	0.03
174	281.20	319	2.07
175	282.20	13	0.08
176	283.10	4	0.03
177	310.20	4	0.03
178	329.20	3	0.02
179	357.10	3	0.02
180	369.20	4	0.03
181	372.20	3	0.02
182	380.10	4	0.03
183	385.20	8	0.05
184	424.00	4	0.03
185	467.10	7	0.05
186	499.20	4	0.03
187	560.20	3	0.02
188	564.00	8	0.05
189	598.20	3	0.02
190	612.10	3	0.02
191	620.20	4	0.03
192	624.20	4	0.03
193	665.20	3	0.02
194	675.30	4	0.03

Line#:20 R.Time:27.8(Scan#:7131)
MassPeaks:216
RawMode:Averaged 27.7-27.8(7106-7149) BasePeak:57(108072)
BG Mode:None Group 1 - Event 1

#	m/z	Abs. Int.	Rel. Int.
1	50.00	670	0.62
2	51.00	703	0.65
3	52.00	474	0.44
4	53.15	1867	1.73
5	54.15	2539	2.35
6	55.15	25786	23.86
7	56.15	13420	12.42
8	57.15	108072	100.00
9	58.15	6924	6.41
10	59.15	3157	2.92
11	60.10	585	0.54
12	61.10	305	0.28
13	62.10	155	0.14
14	63.10	402	0.37
15	64.10	299	0.28
16	65.10	738	0.68
17	66.10	734	0.68
18	67.10	4680	4.33
19	68.15	3445	3.19
20	69.10	18674	17.28
21	70.15	10657	9.86
22	71.15	74602	69.03
23	72.15	4302	3.98
24	73.10	1707	1.58
25	74.10	344	0.32
26	75.10	638	0.59
27	76.10	307	0.28
28	77.15	1540	1.42
29	78.10	530	0.49
30	79.15	1714	1.59
31	80.10	638	0.59
32	81.15	4594	4.25
33	82.15	5190	4.80
34	83.15	13840	12.81
35	84.15	6188	5.73
36	85.15	51748	47.88
37	86.20	3456	3.20
38	87.20	342	0.32
39	88.20	44	0.04
40	89.20	314	0.29
41	90.20	19	0.02
42	91.10	1425	1.32
43	92.10	419	0.39
44	93.10	1366	1.26
45	94.10	638	0.59
46	95.15	2540	2.35
47	96.15	4219	3.90
48	97.15	13044	12.07
49	98.20	4201	3.89
50	99.20	21829	20.20
51	100.15	1817	1.68
52	101.20	315	0.29
53	102.20	88	0.08
54	103.20	354	0.33
55	104.20	126	0.12
56	105.15	860	0.80
57	106.10	291	0.27
58	107.15	1010	0.93
59	108.20	407	0.38
60	109.15	1352	1.25
61	110.20	2241	2.07
62	111.15	6932	6.41
63	112.20	2831	2.62
64	113.20	13178	12.19
65	114.20	1349	1.25
66	115.20	485	0.45
67	116.20	310	0.29
68	117.20	490	0.45
69	118.20	100	0.09
70	119.15	750	0.69
71	120.10	214	0.20
72	121.20	721	0.67
73	122.20	311	0.29
74	123.20	919	0.85
75	124.20	1346	1.25
76	125.20	3471	3.21
77	126.20	1936	1.79
78	127.20	8742	8.09
79	128.20	1050	0.97
80	129.20	503	0.47
81	130.10	86	0.08
82	131.20	347	0.32
83	132.20	84	0.08
84	133.15	679	0.63
85	134.20	260	0.24
86	135.15	586	0.54
87	136.20	428	0.40
88	137.20	484	0.45
89	138.20	822	0.76
90	139.20	1586	1.47
91	140.25	1278	1.18
92	141.25	5380	4.98
93	142.25	670	0.62
94	143.20	171	0.16
95	144.20	38	0.04
96	145.20	266	0.25
97	146.10	26	0.02
98	147.20	446	0.41
99	148.20	94	0.09
100	149.10	367	0.34
101	150.20	148	0.14
102	151.20	507	0.47
103	152.20	588	0.54
104	153.25	875	0.81
105	154.25	906	0.84
106	155.25	3495	3.23
107	156.25	442	0.41
108	157.30	118	0.11
109	159.20	85	0.08
110	160.30	18	0.02
111	161.20	224	0.21
112	162.20	19	0.02
113	163.20	188	0.17
114	164.20	23	0.02
115	165.20	290	0.27
116	166.25	434	0.40
117	167.30	561	0.52
118	168.25	659	0.61
119	169.30	2419	2.24
120	170.30	341	0.32
121	171.20	59	0.05
122	173.20	21	0.02
123	175.20	163	0.15
124	177.20	183	0.17
125	179.30	274	0.25
126	180.30	253	0.23
127	181.30	311	0.29
128	182.25	501	0.46
129	183.25	1628	1.51
130	184.30	264	0.24
131	185.30	32	0.03
132	187.30	41	0.04
133	189.20	53	0.05
134	190.20	4	0.00
135	191.30	292	0.27
136	192.30	52	0.05
137	193.20	223	0.21
138	194.30	127	0.12
139	195.30	205	0.19
140	196.20	296	0.27
141	197.25	1257	1.16
142	198.30	210	0.19
143	199.20	5	0.00
144	200.20	4	0.00
145	201.20	5	0.00
146	203.20	84	0.08
147	205.20	28	0.03
148	206.10	4	0.00
149	207.05	1072	0.99
150	208.10	334	0.31
151	209.10	241	0.22
152	210.10	244	0.23
153	211.25	946	0.88
154	212.20	190	0.18
155	213.20	4	0.00
156	217.20	5	0.00
157	219.10	4	0.00
158	221.10	33	0.03
159	222.30	69	0.06
160	223.30	86	0.08
161	224.20	209	0.19
162	225.30	677	0.63
163	226.30	140	0.13
164	231.20	9	0.01
165	234.30	4	0.00
166	236.30	54	0.05
167	237.30	48	0.04
168	238.30	158	0.15
169	239.25	527	0.49
170	240.30	91	0.08
171	249.10	15	0.01
172	250.30	10	0.01
173	251.20	4	0.00
174	252.30	80	0.07
175	253.30	351	0.32
176	254.30	38	0.04
177	265.20	63	0.06
178	266.30	70	0.06
179	267.35	320	0.30
180	268.30	21	0.02
181	271.20	10	0.01
182	280.30	47	0.04
183	281.25	520	0.48
184	282.20	95	0.09
185	283.30	24	0.02
186	294.30	27	0.02
187	295.30	135	0.12
188	305.30	11	0.01
189	308.30	9	0.01
190	309.30	71	0.07
191	322.30	15	0.01
192	323.30	57	0.05
193	337.30	49	0.05
194	338.30	4	0.00
195	341.20	11	0.01

DEPTT. OF BOTANICAL & ENVIRONMENTAL SCIENCES, G.N.D.U. AMRITSAR

#	m/z	Abs. Int.	Rel. Int.	#	m/z	Abs. Int.	Rel. Int.	#	m/z	Abs. Int.	Rel. Int.
196	350.30	11	0.01	203	407.20	4	0.00	210	511.10	4	0.00
197	351.30	15	0.01	204	408.30	4	0.00	211	532.20	4	0.00
198	352.30	11	0.01	205	414.30	9	0.01	212	555.30	9	0.01
199	355.20	4	0.00	206	435.30	5	0.00	213	566.10	5	0.00
200	364.30	4	0.00	207	457.10	9	0.01	214	613.30	4	0.00
201	365.30	15	0.01	208	459.30	4	0.00	215	643.30	4	0.00
202	403.30	4	0.00	209	497.30	5	0.00	216	677.30	5	0.00

Line#:21 R.Time:31.1(Scan#:8139)
MassPeaks:279
RawMode:Averaged 31.0-31.3(8099-8188) BasePeak:57(39615)
BG Mode:None Group 1 - Event 1

#	m/z	Abs. Int.	Rel. Int.	#	m/z	Abs. Int.	Rel. Int.	#	m/z	Abs. Int.	Rel. Int.
1	50.00	633	1.60	68	117.10	731	1.85	135	184.20	23	0.06
2	51.10	840	2.12	69	118.10	246	0.62	136	185.20	56	0.14
3	52.10	543	1.37	70	119.15	1777	4.49	137	186.20	25	0.06
4	53.15	2086	5.27	71	120.15	485	1.22	138	187.20	406	1.02
5	54.15	2894	7.31	72	121.10	3786	9.56	139	188.10	122	0.31
6	55.15	27934	70.51	73	122.15	1283	3.24	140	189.15	720	1.82
7	56.15	11122	28.08	74	123.15	2046	5.16	141	190.15	556	1.40
8	57.15	39615	100.00	75	124.20	2460	6.21	142	191.15	827	2.09
9	58.15	2117	5.34	76	125.20	5631	14.21	143	192.10	175	0.44
10	59.10	510	1.29	77	126.20	1383	3.49	144	193.10	308	0.78
11	60.20	523	1.32	78	127.20	1356	3.42	145	194.20	252	0.64
12	61.20	327	0.83	79	128.20	479	1.21	146	195.20	334	0.84
13	62.20	150	0.38	80	129.20	567	1.43	147	196.20	151	0.38
14	63.20	455	1.15	81	130.20	206	0.52	148	197.20	135	0.34
15	64.10	320	0.81	82	131.15	682	1.72	149	198.20	9	0.02
16	65.10	1023	2.58	83	132.10	217	0.55	150	199.20	20	0.05
17	66.10	1417	3.58	84	133.10	1069	2.70	151	200.20	7	0.02
18	67.10	9056	22.86	85	134.20	438	1.11	152	201.20	90	0.23
19	68.10	8667	21.88	86	135.15	1673	4.22	153	202.10	60	0.15
20	69.15	22451	56.67	87	136.15	2773	7.00	154	203.15	776	1.96
21	70.15	9325	23.54	88	137.20	3032	7.65	155	204.20	220	0.56
22	71.15	19472	49.15	89	138.20	1823	4.60	156	205.15	3537	8.93
23	72.15	1347	3.40	90	139.20	2487	6.28	157	206.20	568	1.43
24	73.10	1916	4.84	91	140.20	781	1.97	158	207.10	1378	3.48
25	74.10	337	0.85	92	141.20	907	2.29	159	208.10	445	1.12
26	75.10	548	1.38	93	142.20	230	0.58	160	209.10	380	0.96
27	76.10	324	0.82	94	143.20	355	0.90	161	210.10	138	0.35
28	77.10	2150	5.43	95	144.20	147	0.37	162	211.10	114	0.29
29	78.10	663	1.67	96	145.20	589	1.49	163	212.20	2	0.01
30	79.10	2740	6.92	97	146.20	185	0.47	164	213.10	35	0.09
31	80.10	1068	2.70	98	147.20	1099	2.77	165	215.10	65	0.16
32	81.15	8820	22.26	99	148.20	337	0.85	166	217.10	68	0.17
33	82.15	13723	34.64	100	149.15	1458	3.68	167	218.10	77	0.19
34	83.15	22406	56.56	101	150.20	335	0.85	168	219.10	202	0.51
35	84.15	5712	14.42	102	151.20	960	2.42	169	220.00	65	0.16
36	85.15	10782	27.22	103	152.20	1399	3.53	170	221.10	83	0.21
37	86.20	865	2.18	104	153.25	1538	3.88	171	222.10	137	0.35
38	87.20	323	0.82	105	154.25	499	1.26	172	223.10	159	0.40
39	88.10	56	0.14	106	155.30	528	1.33	173	224.10	90	0.23
40	89.20	312	0.79	107	156.30	95	0.24	174	225.10	81	0.20
41	90.20	30	0.08	108	157.20	256	0.65	175	227.20	6	0.02
42	91.10	2946	7.44	109	158.10	92	0.23	176	229.10	20	0.05
43	92.10	577	1.46	110	159.20	562	1.42	177	230.10	2	0.01
44	93.10	2108	5.32	111	160.20	154	0.39	178	232.10	24	0.06
45	94.15	1024	2.58	112	161.10	678	1.71	179	233.10	4	0.01
46	95.15	6020	15.20	113	162.15	564	1.42	180	234.10	9	0.02
47	96.15	9060	22.87	114	163.20	771	1.95	181	235.10	9	0.02
48	97.20	21506	54.29	115	164.20	12151	30.67	182	236.10	98	0.25
49	98.15	3766	9.51	116	165.15	29594	74.70	183	237.10	86	0.22
50	99.15	3673	9.27	117	166.20	4222	10.66	184	238.10	40	0.10
51	100.20	363	0.92	118	167.20	1190	3.00	185	239.10	24	0.06
52	101.20	146	0.37	119	168.30	355	0.90	186	241.10	9	0.02
53	102.20	83	0.21	120	169.30	319	0.81	187	242.10	2	0.01
54	103.20	522	1.32	121	170.30	24	0.06	188	243.10	4	0.01
55	104.10	241	0.61	122	171.20	107	0.27	189	244.10	2	0.01
56	105.10	1720	4.34	123	172.20	137	0.35	190	246.10	21	0.05
57	106.10	459	1.16	124	173.20	248	0.63	191	249.10	51	0.13
58	107.15	1930	4.87	125	174.20	125	0.32	192	250.10	77	0.19
59	108.10	772	1.95	126	175.20	661	1.67	193	251.10	75	0.19
60	109.15	3824	9.65	127	176.20	477	1.20	194	252.10	27	0.07
61	110.15	3455	8.72	128	177.20	1045	2.64	195	253.20	15	0.04
62	111.20	10849	27.39	129	178.15	393	0.99	196	255.10	17	0.04
63	112.20	2315	5.84	130	179.10	464	1.17	197	257.10	6	0.02
64	113.20	2056	5.19	131	180.25	383	0.97	198	260.10	11	0.03
65	114.20	245	0.62	132	181.30	506	1.28	199	262.10	2	0.01
66	115.10	758	1.91	133	182.20	237	0.60	200	263.10	2	0.01
67	116.10	336	0.85	134	183.20	248	0.63	201	264.10	48	0.12

#	m/z	Abs. Int.	Rel. Int.	#	m/z	Abs. Int.	Rel. Int.	#	m/z	Abs. Int.	Rel. Int.
202	265.10	91	0.23	228	309.10	2	0.01	254	413.10	2	0.01
203	266.10	25	0.06	229	310.20	2	0.01	255	424.10	2	0.01
204	267.00	110	0.28	230	315.10	2	0.01	256	429.10	4	0.01
205	269.10	9	0.02	231	316.10	4	0.01	257	430.40	5018	12.67
206	270.10	2	0.01	232	320.10	2	0.01	258	431.40	1579	3.99
207	271.10	2	0.01	233	321.20	2	0.01	259	432.35	269	0.68
208	273.10	4	0.01	234	328.10	2	0.01	260	433.40	15	0.04
209	274.10	18	0.05	235	330.20	2	0.01	261	437.40	2	0.01
210	275.10	5	0.01	236	331.20	9	0.02	262	447.20	2	0.01
211	277.10	2	0.01	237	332.10	5	0.01	263	481.40	2	0.01
212	278.10	38	0.10	238	341.10	15	0.04	264	492.10	2	0.01
213	279.10	5	0.01	239	342.10	9	0.02	265	536.40	2	0.01
214	280.10	4	0.01	240	343.10	2	0.01	266	545.10	2	0.01
215	281.10	451	1.14	241	345.10	2	0.01	267	546.40	2	0.01
216	282.10	53	0.13	242	348.10	2	0.01	268	556.40	2	0.01
217	283.10	59	0.15	243	355.10	7	0.02	269	566.10	2	0.01
218	285.10	5	0.01	244	369.20	5	0.01	270	568.10	2	0.01
219	288.10	54	0.14	245	374.10	4	0.01	271	570.40	4	0.01
220	289.10	2	0.01	246	375.10	2	0.01	272	574.10	2	0.01
221	292.10	17	0.04	247	383.00	2	0.01	273	581.40	2	0.01
222	293.10	15	0.04	248	387.10	29	0.07	274	588.40	2	0.01
223	297.10	4	0.01	249	388.10	4	0.01	275	629.40	2	0.01
224	298.10	26	0.07	250	394.10	2	0.01	276	641.10	2	0.01
225	299.10	2	0.01	251	399.20	2	0.01	277	660.10	2	0.01
226	302.10	10	0.03	252	409.10	5	0.01	278	668.40	2	0.01
227	307.10	4	0.01	253	410.20	2	0.01	279	690.10	2	0.01

Line#:22 R.Time:33.7(Scan#:8907)
MassPeaks:367
RawMode:Averaged 33.4-33.8(8818-8946) BasePeak:137(79578)
BG Mode:None Group 1 - Event 1

#	m/z	Abs. Int.	Rel. Int.	#	m/z	Abs. Int.	Rel. Int.	#	m/z	Abs. Int.	Rel. Int.
1	50.15	727	0.91	49	98.15	1309	1.64	97	146.20	1547	1.94
2	51.10	1072	1.35	50	99.10	1073	1.35	98	147.20	7860	9.88
3	52.15	658	0.83	51	100.10	294	0.37	99	148.20	2719	3.42
4	53.10	5833	7.33	52	101.10	366	0.46	100	149.20	10802	13.57
5	54.15	2037	2.56	53	102.10	334	0.42	101	150.15	6195	7.78
6	55.10	44242	55.60	54	103.10	1141	1.43	102	151.20	6575	8.26
7	56.15	4716	5.93	55	104.10	580	0.73	103	152.20	2805	3.52
8	57.15	16460	20.68	56	105.10	14884	18.70	104	153.20	1960	2.46
9	58.15	974	1.22	57	106.15	3280	4.12	105	154.20	416	0.52
10	59.15	866	1.09	58	107.10	24428	30.70	106	155.15	656	0.82
11	60.10	534	0.67	59	108.10	8864	11.14	107	156.15	778	0.98
12	61.10	339	0.43	60	109.15	33658	42.30	108	157.15	1698	2.13
13	62.10	199	0.25	61	110.15	6346	7.97	109	158.15	627	0.79
14	63.10	515	0.65	62	111.15	9485	11.92	110	159.15	3933	4.94
15	64.10	378	0.48	63	112.15	1154	1.45	111	160.25	1091	1.37
16	65.05	2800	3.52	64	113.15	1261	1.58	112	161.20	5364	6.74
17	66.15	1296	1.63	65	114.15	316	0.40	113	162.20	1913	2.40
18	67.10	32304	40.59	66	115.10	1656	2.08	114	163.20	8198	10.30
19	68.10	6836	8.59	67	116.10	764	0.96	115	164.20	2282	2.87
20	69.10	50235	63.13	68	117.15	3287	4.13	116	165.20	2303	2.89
21	70.10	4640	5.83	69	118.15	1006	1.26	117	166.20	728	0.91
22	71.10	6259	7.87	70	119.15	13183	16.57	118	167.20	596	0.75
23	72.15	681	0.86	71	120.15	4038	5.07	119	168.20	200	0.25
24	73.10	1755	2.21	72	121.15	19963	25.09	120	169.15	543	0.68
25	74.10	415	0.52	73	122.20	5643	7.09	121	170.20	402	0.51
26	75.10	527	0.66	74	123.15	22550	28.34	122	171.15	1556	1.96
27	76.10	350	0.44	75	124.15	4281	5.38	123	172.20	559	0.70
28	77.10	6656	8.36	76	125.15	5309	6.67	124	173.20	3314	4.16
29	78.10	1565	1.97	77	126.20	1171	1.47	125	174.20	1002	1.26
30	79.10	18129	22.78	78	127.15	726	0.91	126	175.20	4338	5.45
31	80.10	3057	3.84	79	128.15	1235	1.55	127	176.20	1636	2.06
32	81.10	35530	44.65	80	129.10	2091	2.63	128	177.20	4804	6.04
33	82.10	9649	12.13	81	130.15	883	1.11	129	178.15	1358	1.71
34	83.10	16912	21.25	82	131.15	4758	5.98	130	179.20	3056	3.84
35	84.10	2494	3.13	83	132.15	1350	1.70	131	180.20	595	0.75
36	85.15	3040	3.82	84	133.15	9280	11.66	132	181.20	340	0.43
37	86.10	387	0.49	85	134.20	3667	4.61	133	182.20	143	0.18
38	87.10	466	0.59	86	135.15	12304	15.46	134	183.15	438	0.55
39	88.10	326	0.41	87	136.15	7780	9.78	135	184.20	224	0.28
40	89.10	403	0.51	88	137.15	79578	100.00	136	185.15	1326	1.67
41	90.10	108	0.14	89	138.15	12690	15.95	137	186.20	442	0.56
42	91.10	15005	18.86	90	139.15	3826	4.81	138	187.20	2624	3.30
43	92.10	2507	3.15	91	140.15	882	1.11	139	188.20	1075	1.35
44	93.10	21933	27.56	92	141.15	912	1.15	140	189.20	4402	5.53
45	94.15	5152	6.47	93	142.15	1053	1.32	141	190.20	1639	2.06
46	95.15	46558	58.51	94	143.15	1962	2.47	142	191.20	7995	10.05
47	96.15	9452	11.88	95	144.15	827	1.04	143	192.20	1991	2.50
48	97.15	9398	11.81	96	145.15	5250	6.60	144	193.20	1483	1.86

DEPTT. OF BOTANICAL & ENVIRONMENTAL SCIENCES, G.N.D.U. AMRITSAR

#	m/z	Abs. Int.	Rel. Int.
145	194.15	299	0.38
146	195.20	216	0.27
147	196.20	74	0.09
148	197.20	337	0.42
149	198.20	290	0.36
150	199.15	1168	1.47
151	200.20	380	0.48
152	201.15	1679	2.11
153	202.20	878	1.10
154	203.20	3587	4.51
155	204.20	2179	2.74
156	205.20	9381	11.79
157	206.20	3313	4.16
158	207.15	3161	3.97
159	208.20	721	0.91
160	209.20	340	0.43
161	210.10	59	0.07
162	211.20	264	0.33
163	212.20	112	0.14
164	213.10	795	1.00
165	214.20	336	0.42
166	215.15	1653	2.08
167	216.20	630	0.79
168	217.15	2427	3.05
169	218.20	2537	3.19
170	219.20	2984	3.75
171	220.20	1059	1.33
172	221.20	572	0.72
173	222.20	151	0.19
174	223.20	136	0.17
175	224.20	19	0.02
176	225.20	182	0.23
177	226.20	56	0.07
178	227.10	651	0.82
179	228.20	247	0.31
180	229.20	1323	1.66
181	230.15	435	0.55
182	231.20	1708	2.15
183	232.20	628	0.79
184	233.20	618	0.78
185	234.20	203	0.26
186	235.20	126	0.16
187	236.20	30	0.04
188	237.20	82	0.10
189	238.20	13	0.02
190	239.20	172	0.22
191	240.20	67	0.08
192	241.15	683	0.86
193	242.20	213	0.27
194	243.25	760	0.96
195	244.20	353	0.44
196	245.20	1539	1.93
197	246.20	503	0.63
198	247.15	757	0.95
199	248.20	189	0.24
200	249.20	172	0.22
201	250.20	20	0.03
202	251.20	58	0.07
203	252.20	5	0.01
204	253.20	210	0.26
205	254.20	47	0.06
206	255.20	1311	1.65
207	256.25	373	0.47
208	257.15	676	0.85
209	258.25	293	0.37
210	259.25	3166	3.98
211	260.25	834	1.05
212	261.20	283	0.36
213	262.20	115	0.14
214	263.20	21	0.03
215	264.10	3	0.00
216	265.30	97	0.12
217	266.20	18	0.02
218	267.20	199	0.25
219	268.20	37	0.05

#	m/z	Abs. Int.	Rel. Int.
220	269.20	513	0.64
221	270.20	179	0.22
222	271.25	668	0.84
223	272.20	327	0.41
224	273.25	1767	2.22
225	274.30	1249	1.57
226	275.30	652	0.82
227	276.30	227	0.29
228	277.30	23	0.03
229	278.30	1	0.00
230	279.30	7	0.01
231	280.30	6	0.01
232	281.05	551	0.69
233	282.20	159	0.20
234	283.20	269	0.34
235	284.30	122	0.15
236	285.20	411	0.52
237	286.30	225	0.28
238	287.25	706	0.89
239	288.25	299	0.38
240	289.30	207	0.26
241	290.20	28	0.04
242	293.30	1	0.00
243	295.30	142	0.18
244	296.30	10	0.01
245	297.30	196	0.25
246	298.30	52	0.07
247	299.30	276	0.35
248	300.30	155	0.19
249	301.25	281	0.35
250	302.30	158	0.20
251	303.30	54	0.07
252	304.30	1	0.00
253	307.30	1	0.00
254	308.20	1	0.00
255	309.30	70	0.09
256	311.30	194	0.24
257	312.30	45	0.06
258	313.30	964	1.21
259	314.30	276	0.35
260	315.30	144	0.18
261	316.30	30	0.04
262	319.30	1	0.00
263	321.30	1	0.00
264	323.30	19	0.02
265	324.30	13	0.02
266	325.30	331	0.42
267	326.30	207	0.26
268	327.30	376	0.47
269	328.30	108	0.14
270	329.30	298	0.37
271	330.30	75	0.09
272	331.30	6	0.01
273	336.30	1	0.00
274	337.30	199	0.25
275	338.30	66	0.08
276	339.30	98	0.12
277	340.20	11	0.01
278	341.30	203	0.26
279	342.20	107	0.13
280	343.35	1912	2.40
281	344.35	1640	2.06
282	345.35	440	0.55
283	346.30	32	0.04
284	351.30	13	0.02
285	352.30	13	0.02
286	353.30	55	0.07
287	354.30	38	0.05
288	355.30	706	0.89
289	356.30	210	0.26
290	357.30	54	0.07
291	362.30	1	0.00
292	365.30	71	0.09
293	367.30	42	0.05
294	368.30	9	0.01

#	m/z	Abs. Int.	Rel. Int.
295	369.30	467	0.59
296	370.40	352	0.44
297	371.40	88	0.11
298	374.10	3	0.00
299	379.40	1	0.00
300	381.30	3	0.00
301	382.40	6	0.01
302	383.30	295	0.37
303	384.30	89	0.11
304	385.30	3	0.00
305	386.30	1	0.00
306	389.10	1	0.00
307	391.30	100	0.13
308	392.30	18	0.02
309	393.45	236	0.30
310	394.30	61	0.08
311	395.30	70	0.09
312	396.40	1	0.00
313	397.30	58	0.07
314	398.40	31	0.04
315	402.30	1	0.00
316	404.30	1	0.00
317	406.30	12	0.02
318	408.40	74	0.09
319	409.30	164	0.21
320	410.40	55	0.07
321	411.35	17359	21.81
322	412.35	5440	6.84
323	413.40	933	1.17
324	414.40	106	0.13
325	415.40	3	0.00
326	416.40	1	0.00
327	422.40	6	0.01
328	424.35	496	0.62
329	425.45	181	0.23
330	426.40	2801	3.52
331	427.40	895	1.12
332	428.40	173	0.22
333	429.40	10	0.01
334	430.40	1	0.00
335	431.40	1	0.00
336	436.40	1	0.00
337	438.40	1	0.00
338	453.10	3	0.00
339	458.30	1	0.00
340	463.40	1	0.00
341	471.40	1	0.00
342	476.40	1	0.00
343	478.10	1	0.00
344	492.40	3	0.00
345	527.40	1	0.00
346	531.40	1	0.00
347	534.10	1	0.00
348	538.40	1	0.00
349	543.40	1	0.00
350	545.40	1	0.00
351	546.40	1	0.00
352	547.40	1	0.00
353	556.00	1	0.00
354	572.10	1	0.00
355	579.40	4	0.01
356	594.40	1	0.00
357	608.40	1	0.00
358	609.40	1	0.00
359	618.40	1	0.00
360	640.40	1	0.00
361	649.40	1	0.00
362	660.10	3	0.00
363	674.40	1	0.00
364	680.40	1	0.00
365	690.40	1	0.00
366	697.40	1	0.00
367	700.40	1	0.00

Line#:23 R.Time:35.2(Scan#:9353)
MassPeaks:343
RawMode:Averaged 35.0-35.3(9301-9379) BasePeak:55(23290)
BG Mode:None Group 1 - Event 1

DEPTT. OF BOTANICAL & ENVIRONMENTAL SCIENCES,
G.N.D.U.
AMRITSAR

#	m/z	Abs. Int.	Rel. Int.
1	50.15	709	3.04
2	51.15	1086	4.66
3	52.15	630	2.71
4	53.15	3120	13.40
5	54.15	932	4.00
6	55.10	23290	100.00
7	56.15	2703	11.61
8	57.10	20845	89.50
9	58.15	1286	5.52
10	59.10	616	2.64
11	60.10	478	2.05
12	61.10	251	1.08
13	62.20	226	0.97
14	63.20	613	2.63
15	64.10	439	1.88
16	65.05	1917	8.23
17	66.15	827	3.55
18	67.10	12725	54.64
19	68.15	2195	9.42
20	69.10	16540	71.02
21	70.15	1936	8.31
22	71.15	10102	43.37
23	72.15	764	3.28
24	73.10	2010	8.63
25	74.10	397	1.70
26	75.10	510	2.19
27	76.10	411	1.76
28	77.10	5125	22.01
29	78.10	1624	6.97
30	79.10	13216	56.75
31	80.15	2039	8.75
32	81.15	19156	82.25
33	82.15	2837	12.18
34	83.15	7384	31.70
35	84.15	1289	5.53
36	85.15	7431	31.91
37	86.10	655	2.81
38	87.20	383	1.64
39	88.20	79	0.34
40	89.20	431	1.85
41	90.10	165	0.71
42	91.10	15021	64.50
43	92.10	3142	13.49
44	93.15	13788	59.20
45	94.15	3930	16.87
46	95.15	20903	89.75
47	96.15	3125	13.42
48	97.15	6054	25.99
49	98.15	940	4.04
50	99.20	1871	8.03
51	100.20	246	1.06
52	101.20	251	1.08
53	102.10	239	1.03
54	103.10	1252	5.38
55	104.15	924	3.97
56	105.15	16953	72.79
57	106.15	3986	17.11
58	107.15	15564	66.83
59	108.10	4509	19.36
60	109.15	14401	61.83
61	110.20	2447	10.51
62	111.15	4322	18.56
63	112.20	610	2.62
64	113.15	773	3.32
65	114.10	191	0.82
66	115.10	2313	9.93
67	116.10	1000	4.29
68	117.10	5688	24.42
69	118.15	2414	10.36
70	119.15	12009	51.56
71	120.15	5824	25.01
72	121.15	9643	41.40
73	122.15	3088	13.26
74	123.20	6020	25.85
75	124.15	2297	9.86
76	125.20	2245	9.64
77	126.15	424	1.82
78	127.15	945	4.06
79	128.15	2128	9.14
80	129.15	3715	15.95

#	m/z	Abs. Int.	Rel. Int.
81	130.15	1636	7.02
82	131.15	7847	33.69
83	132.15	2403	10.32
84	133.15	10051	43.16
85	134.20	3679	15.80
86	135.15	8112	34.83
87	136.15	2753	11.82
88	137.20	2581	11.08
89	138.15	961	4.13
90	139.20	924	3.97
91	140.20	167	0.72
92	141.20	1387	5.96
93	142.10	1246	5.35
94	143.15	5551	23.83
95	144.15	2005	8.61
96	145.15	10793	46.34
97	146.20	3002	12.89
98	147.15	7775	33.38
99	148.20	2654	11.40
100	149.20	5009	21.51
101	150.20	1095	4.70
102	151.20	1774	7.62
103	152.20	645	2.77
104	153.15	692	2.97
105	154.20	370	1.59
106	155.15	1076	4.62
107	156.25	717	3.08
108	157.20	3349	14.38
109	158.15	2663	11.43
110	159.20	6898	29.62
111	160.20	3924	16.85
112	161.20	6681	28.69
113	162.25	1971	8.46
114	163.20	5819	24.98
115	164.20	1115	4.79
116	165.20	1330	5.71
117	166.20	496	2.13
118	167.20	485	2.08
119	168.20	251	1.08
120	169.15	893	3.83
121	170.20	422	1.81
122	171.20	3085	13.25
123	172.20	1118	4.80
124	173.20	3972	17.05
125	174.25	1585	6.81
126	175.20	2726	11.70
127	176.20	1053	4.52
128	177.20	2131	9.15
129	178.25	1929	8.28
130	179.20	1061	4.56
131	180.20	304	1.31
132	181.20	344	1.48
133	182.20	181	0.78
134	183.15	722	3.10
135	184.20	371	1.59
136	185.20	2597	11.15
137	186.20	1160	4.98
138	187.20	2699	11.59
139	188.25	911	3.91
140	189.25	4708	20.21
141	190.20	1584	6.80
142	191.20	3312	14.22
143	192.20	712	3.06
144	193.25	640	2.75
145	194.20	139	0.60
146	195.20	256	1.10
147	196.20	82	0.35
148	197.15	805	3.46
149	198.20	320	1.37
150	199.15	3102	13.32
151	200.20	1037	4.45
152	201.15	1704	7.32
153	202.20	718	3.08
154	203.20	2720	11.68
155	204.25	2178	9.35
156	205.25	3583	15.38
157	206.25	1416	6.08
158	207.15	3111	13.36
159	208.15	736	3.16
160	209.10	412	1.77

#	m/z	Abs. Int.	Rel. Int.
161	210.10	78	0.33
162	211.15	586	2.52
163	212.25	332	1.43
164	213.15	6023	25.86
165	214.20	1783	7.66
166	215.20	1607	6.90
167	216.15	494	2.12
168	217.20	872	3.74
169	218.20	2651	11.38
170	219.20	1239	5.32
171	220.20	301	1.29
172	221.20	599	2.57
173	222.20	158	0.68
174	223.20	128	0.55
175	224.20	34	0.15
176	225.20	370	1.59
177	226.20	179	0.77
178	227.20	1309	5.62
179	228.20	1188	5.10
180	229.20	2134	9.16
181	230.20	565	2.43
182	231.20	2795	12.00
183	232.15	842	3.62
184	233.20	536	2.30
185	234.25	521	2.24
186	235.20	251	1.08
187	236.30	73	0.31
188	237.20	78	0.33
189	238.20	23	0.10
190	239.20	645	2.77
191	240.20	178	0.76
192	241.20	874	3.75
193	242.20	296	1.27
194	243.20	359	1.54
195	244.20	226	0.97
196	245.25	471	2.02
197	246.20	479	2.06
198	247.20	488	2.10
199	248.30	217	0.93
200	249.20	159	0.68
201	251.20	58	0.25
202	252.20	11	0.05
203	253.20	402	1.73
204	254.20	256	1.10
205	255.25	3252	13.96
206	256.20	743	3.19
207	257.25	610	2.62
208	258.20	121	0.52
209	259.20	344	1.48
210	260.20	187	0.80
211	261.20	198	0.85
212	262.20	21	0.09
213	263.30	2	0.01
214	265.20	106	0.46
215	266.20	5	0.02
216	267.20	261	1.12
217	268.20	69	0.30
218	269.20	300	1.29
219	270.20	89	0.38
220	271.30	380	1.63
221	272.20	244	1.05
222	273.20	1588	6.82
223	274.30	567	2.43
224	275.25	807	3.47
225	276.30	192	0.82
226	277.30	7	0.03
227	278.30	2	0.01
228	280.30	5	0.02
229	281.10	695	2.98
230	282.10	208	0.89
231	283.25	440	1.89
232	284.30	106	0.46
233	285.30	109	0.47
234	286.30	8	0.03
235	287.30	199	0.85
236	288.30	314	1.35
237	289.30	202	0.87
238	290.30	78	0.33
239	291.30	6	0.03
240	294.20	2	0.01

DEPTT. OF BOTANICAL & ENVIRONMENTAL SCIENCES, G.N.D.U. AMRITSAR

#	m/z	Abs. Int.	Rel. Int.
241	295.30	13	0.06
242	296.30	8	0.03
243	297.30	96	0.41
244	299.30	47	0.20
245	300.30	28	0.12
246	301.30	151	0.65
247	302.35	582	2.50
248	303.35	1783	7.66
249	304.35	451	1.94
250	305.30	35	0.15
251	306.40	2	0.01
252	311.30	22	0.09
253	312.40	2	0.01
254	313.30	30	0.13
255	314.30	41	0.18
256	315.30	83	0.36
257	316.30	8	0.03
258	318.30	2	0.01
259	325.30	35	0.15
260	326.30	17	0.07
261	327.30	86	0.37
262	328.40	152	0.65
263	329.35	2441	10.48
264	330.40	650	2.79
265	331.40	111	0.48
266	335.20	5	0.02
267	337.30	2	0.01
268	339.40	81	0.35
269	340.40	14	0.06
270	341.40	190	0.82
271	342.40	187	0.80
272	343.40	49	0.21
273	344.40	11	0.05
274	352.40	2	0.01
275	353.40	68	0.29
276	354.35	385	1.65
277	355.35	342	1.47
278	356.40	112	0.48
279	357.40	116	0.50
280	358.40	20	0.09
281	366.30	3	0.01
282	367.40	85	0.36
283	368.40	60	0.26
284	369.40	9	0.04
285	370.40	33	0.14
286	371.30	63	0.27
287	372.40	14	0.06
288	378.10	2	0.01
289	379.30	11	0.05
290	380.40	2	0.01
291	381.40	1355	5.82
292	382.40	406	1.74
293	383.40	73	0.31
294	384.30	7	0.03
295	385.30	16	0.07
296	386.40	15	0.06
297	390.10	2	0.01
298	391.40	8	0.03
299	392.40	2	0.01
300	393.40	14	0.06
301	394.40	23	0.10
302	395.40	29	0.12
303	396.40	1475	6.33
304	397.40	521	2.24
305	398.40	67	0.29
306	399.40	993	4.26
307	400.40	274	1.18
308	401.40	20	0.09
309	406.20	2	0.01
310	408.40	196	0.84
311	409.40	24	0.10
312	410.40	24	0.10
313	411.40	163	0.70
314	412.40	181	0.78
315	413.40	48	0.21
316	414.40	1640	7.04
317	415.45	549	2.36
318	416.40	90	0.39
319	422.40	3	0.01
320	423.40	2	0.01
321	424.40	5	0.02
322	426.40	200	0.86
323	427.40	26	0.11
324	428.40	8	0.03
325	429.40	5	0.02
326	433.40	2	0.01
327	435.40	2	0.01
328	440.30	2	0.01
329	442.20	2	0.01
330	456.40	2	0.01
331	469.40	2	0.01
332	473.20	2	0.01
333	478.40	2	0.01
334	480.40	2	0.01
335	517.10	3	0.01
336	519.30	2	0.01
337	544.40	2	0.01
338	606.20	5	0.02
339	626.40	2	0.01
340	637.40	2	0.01
341	670.40	6	0.03
342	686.40	2	0.01
343	691.20	2	0.01

Line#:24 R.Time:35.4(Scan#:9430)
MassPeaks:354
RawMode:Averaged 35.3-35.5(9379-9458) BasePeak:218(135255)
BG Mode:None Group 1 - Event 1

#	m/z	Abs. Int.	Rel. Int.
1	50.15	823	0.61
2	51.10	1452	1.07
3	52.15	843	0.62
4	53.15	7635	5.64
5	54.15	1563	1.16
6	55.15	55104	40.74
7	56.15	5159	3.81
8	57.10	28003	20.70
9	58.15	2214	1.64
10	59.10	2026	1.50
11	60.10	569	0.42
12	61.10	327	0.24
13	62.10	260	0.19
14	63.10	678	0.50
15	64.15	479	0.35
16	65.10	3858	2.85
17	66.15	1325	0.98
18	67.10	28731	21.24
19	68.15	4611	3.41
20	69.10	55426	40.98
21	70.10	4473	3.31
22	71.10	11918	8.81
23	72.10	1344	0.99
24	73.10	2508	1.85
25	74.10	443	0.33
26	75.10	602	0.45
27	76.15	452	0.33
28	77.10	10115	7.48
29	78.10	2755	2.04
30	79.10	31626	23.38
31	80.10	8488	6.28
32	81.10	46765	34.58
33	82.10	5630	4.16
34	83.10	12861	9.51
35	84.10	1790	1.32
36	85.10	4476	3.31
37	86.10	753	0.56
38	87.15	542	0.40
39	88.10	185	0.14
40	89.10	513	0.38
41	90.15	267	0.20
42	91.10	30048	22.22
43	92.10	5628	4.16
44	93.10	38717	28.63
45	94.10	27230	20.13
46	95.15	56860	42.04
47	96.15	6942	5.13
48	97.15	8992	6.65
49	98.20	1434	1.06
50	99.10	1451	1.07
51	100.10	460	0.34
52	101.10	462	0.34
53	102.15	389	0.29
54	103.10	2336	1.73
55	104.10	1362	1.01
56	105.10	37611	27.81
57	106.15	8507	6.29
58	107.15	39195	28.98
59	108.15	15910	11.76
60	109.15	35980	26.60
61	110.15	4421	3.27
62	111.15	8507	6.29
63	112.15	1088	0.80
64	113.20	905	0.67
65	114.15	457	0.34
66	115.10	3766	2.78
67	116.10	1696	1.25
68	117.10	8568	6.33
69	118.15	2685	1.99
70	119.15	38773	28.67
71	120.15	13679	10.11
72	121.15	30668	22.67
73	122.15	16751	12.38
74	123.15	18076	13.36
75	124.15	2836	2.10
76	125.20	2542	1.88
77	126.15	565	0.42
78	127.15	1450	1.07
79	128.10	3156	2.33
80	129.10	5021	3.71
81	130.10	2220	1.64
82	131.15	11521	8.52
83	132.15	3362	2.49
84	133.15	27164	20.08
85	134.15	14610	10.80
86	135.15	29282	21.65
87	136.20	24297	17.96
88	137.20	15857	11.72
89	138.20	2380	1.76
90	139.15	2670	1.97
91	140.15	492	0.36
92	141.15	2143	1.58
93	142.10	2126	1.57
94	143.15	4622	3.42
95	144.10	2038	1.51
96	145.15	10751	7.95
97	146.15	3730	2.76
98	147.15	23486	17.36
99	148.15	12627	9.34
100	149.20	14349	10.61
101	150.15	4242	3.14
102	151.15	2323	1.72
103	152.20	815	0.60
104	153.15	1144	0.85
105	154.15	728	0.54
106	155.15	1654	1.22
107	156.15	1233	0.91
108	157.15	3298	2.44
109	158.15	1438	1.06
110	159.15	7278	5.38
111	160.15	2521	1.86
112	161.20	16149	11.94
113	162.20	9956	7.36
114	163.20	8843	6.54
115	164.20	1632	1.21
116	165.15	1618	1.20
117	166.20	671	0.50

DEPTT. OF BOTANICAL & ENVIRONMENTAL SCIENCES, G.N.D.U. AMRITSAR

#	m/z	Abs. Int.	Rel. Int.
118	167.20	687	0.51
119	168.15	460	0.34
120	169.10	1131	0.84
121	170.15	593	0.44
122	171.15	2347	1.74
123	172.20	827	0.61
124	173.20	4738	3.50
125	174.20	1592	1.18
126	175.20	19741	14.60
127	176.20	6631	4.90
128	177.20	5011	3.70
129	178.20	1276	0.94
130	179.20	1631	1.21
131	180.15	459	0.34
132	181.20	507	0.37
133	182.10	263	0.19
134	183.15	830	0.61
135	184.20	376	0.28
136	185.20	1620	1.20
137	186.20	705	0.52
138	187.20	4593	3.40
139	188.25	1864	1.38
140	189.20	40644	30.05
141	190.20	14400	10.65
142	191.20	7513	5.55
143	192.25	1487	1.10
144	193.15	1048	0.77
145	194.20	324	0.24
146	195.20	385	0.28
147	196.20	153	0.11
148	197.10	702	0.52
149	198.10	279	0.21
150	199.15	1436	1.06
151	200.15	564	0.42
152	201.15	3372	2.49
153	202.25	1899	1.40
154	203.20	105202	77.78
155	204.20	19954	14.75
156	205.20	7321	5.41
157	206.20	2841	2.10
158	207.15	12658	9.36
159	208.20	3400	2.51
160	209.15	828	0.61
161	210.20	168	0.12
162	211.20	642	0.47
163	212.20	236	0.17
164	213.15	1467	1.08
165	214.20	716	0.53
166	215.20	3507	2.59
167	216.20	1347	1.00
168	217.25	2417	1.79
169	218.20	135255	100.00
170	219.20	24342	18.00
171	220.20	2641	1.95
172	221.15	803	0.59
173	222.20	216	0.16
174	223.20	239	0.18
175	224.20	35	0.03
176	225.20	419	0.31
177	226.10	206	0.15
178	227.15	991	0.73
179	228.15	468	0.35
180	229.20	2972	2.20
181	230.20	926	0.68
182	231.20	2124	1.57
183	232.20	967	0.71
184	233.20	785	0.58
185	234.20	613	0.45
186	235.10	219	0.16
187	236.20	16	0.01
188	237.20	125	0.09
189	238.20	17	0.01
190	239.20	512	0.38
191	240.20	151	0.11
192	241.20	904	0.67
193	242.20	353	0.26
194	243.20	2290	1.69
195	244.20	819	0.61
196	245.20	823	0.61

#	m/z	Abs. Int.	Rel. Int.
197	246.20	299	0.22
198	247.20	189	0.14
199	248.20	65	0.05
200	249.20	219	0.16
201	250.20	25	0.02
202	251.20	182	0.13
203	252.20	15	0.01
204	253.20	407	0.30
205	254.20	156	0.12
206	255.20	1579	1.17
207	256.25	472	0.35
208	257.20	4604	3.40
209	258.20	1252	0.93
210	259.20	830	0.61
211	260.20	165	0.12
212	261.20	17	0.01
213	262.20	6	0.00
214	263.30	2	0.00
215	265.20	262	0.19
216	266.20	62	0.05
217	267.20	384	0.28
218	268.20	117	0.09
219	269.20	874	0.65
220	270.15	513	0.38
221	271.20	1140	0.84
222	272.25	1100	0.81
223	273.25	476	0.35
224	274.30	167	0.12
225	275.20	128	0.09
226	276.20	10	0.01
227	277.30	2	0.00
228	279.30	5	0.00
229	280.10	17	0.01
230	281.05	1102	0.81
231	282.10	391	0.29
232	283.20	492	0.36
233	284.20	235	0.17
234	285.25	298	0.22
235	286.25	283	0.21
236	287.10	136	0.10
237	288.30	119	0.09
238	289.30	15	0.01
239	290.10	5	0.00
240	291.10	7	0.01
241	292.20	2	0.00
242	293.10	18	0.01
243	295.20	115	0.09
244	296.30	56	0.04
245	297.20	222	0.16
246	298.30	55	0.04
247	299.20	160	0.12
248	300.30	42	0.03
249	301.10	21	0.02
250	302.10	10	0.01
251	303.30	53	0.04
252	304.10	2	0.00
253	305.20	2	0.00
254	307.20	9	0.01
255	309.30	39	0.03
256	311.20	150	0.11
257	312.10	52	0.04
258	313.10	22	0.02
259	314.20	98	0.07
260	315.10	61	0.05
261	316.30	10	0.01
262	321.20	5	0.00
263	323.20	44	0.03
264	324.10	13	0.01
265	325.20	70	0.05
266	326.10	8	0.01
267	327.10	41	0.03
268	328.20	10	0.01
269	329.20	84	0.06
270	330.10	2	0.00
271	336.20	6	0.00
272	337.20	152	0.11
273	338.10	18	0.01
274	339.20	96	0.07
275	340.20	8	0.01

#	m/z	Abs. Int.	Rel. Int.
276	341.30	121	0.09
277	342.10	24	0.02
278	343.20	9	0.01
279	347.20	2	0.00
280	349.20	2	0.00
281	351.20	12	0.01
282	352.20	8	0.01
283	353.20	11	0.01
284	354.30	5	0.00
285	355.20	164	0.12
286	356.20	17	0.01
287	357.20	19	0.01
288	358.20	2	0.00
289	359.20	2	0.00
290	363.10	2	0.00
291	365.30	188	0.14
292	366.20	54	0.04
293	367.30	11	0.01
294	368.10	10	0.01
295	369.30	30	0.02
296	379.30	6	0.00
297	381.20	57	0.04
298	382.30	16	0.01
299	383.20	18	0.01
300	384.10	7	0.01
301	385.10	5	0.00
302	390.30	2	0.00
303	391.30	8	0.01
304	393.35	687	0.51
305	394.40	270	0.20
306	395.40	54	0.04
307	396.30	102	0.08
308	397.40	17	0.01
309	398.40	6	0.00
310	399.20	18	0.01
311	401.30	32	0.02
312	406.30	18	0.01
313	407.40	14	0.01
314	408.40	385	0.28
315	409.30	224	0.17
316	410.40	107	0.08
317	411.40	1200	0.89
318	412.35	391	0.29
319	413.40	42	0.03
320	414.40	45	0.03
321	415.30	13	0.01
322	416.40	16	0.01
323	424.40	101	0.07
324	425.30	28	0.02
325	426.40	1719	1.27
326	427.35	572	0.42
327	428.40	96	0.07
328	429.40	5	0.00
329	435.40	2	0.00
330	441.40	2	0.00
331	445.20	2	0.00
332	446.40	2	0.00
333	449.10	2	0.00
334	465.40	2	0.00
335	469.10	2	0.00
336	491.30	7	0.01
337	497.20	2	0.00
338	511.30	2	0.00
339	512.10	2	0.00
340	514.20	2	0.00
341	538.20	8	0.01
342	540.40	2	0.00
343	548.40	2	0.00
344	550.40	2	0.00
345	557.20	2	0.00
346	569.40	2	0.00
347	583.10	2	0.00
348	590.20	2	0.00
349	598.20	2	0.00
350	611.20	2	0.00
351	626.40	2	0.00
352	630.20	2	0.00
353	645.40	2	0.00
354	665.40	2	0.00

DEPTT. OF BOTANICAL & ENVIRONMENTAL SCIENCES, G.N.D.U. AMRITSAR

Line#:25 R.Time:35.6(Scan#:9479)
MassPeaks:273
RawMode:Averaged 35.5-35.7(9458-9507) BasePeak:55(14134)
BG Mode:None Group 1 - Event 1

#	m/z	Abs. Int.	Rel. Int.
1	50.00	690	4.88
2	51.10	1038	7.34
3	52.10	606	4.29
4	53.15	2568	18.17
5	54.15	762	5.39
6	55.15	14134	100.00
7	56.15	1905	13.48
8	57.15	6357	44.98
9	58.15	717	5.07
10	59.10	763	5.40
11	60.20	514	3.64
12	61.10	310	2.19
13	62.10	239	1.69
14	63.10	576	4.08
15	64.10	418	2.96
16	65.10	1661	11.75
17	66.15	657	4.65
18	67.10	8073	57.12
19	68.10	1760	12.45
20	69.10	11230	79.45
21	70.15	1560	11.04
22	71.15	3328	23.55
23	72.10	390	2.76
24	73.10	2562	18.13
25	74.10	446	3.16
26	75.10	607	4.29
27	76.10	445	3.15
28	77.10	4070	28.80
29	78.10	1199	8.48
30	79.15	8263	58.46
31	80.10	1872	13.24
32	81.15	11444	80.97
33	82.15	2070	14.65
34	83.15	3936	27.85
35	84.15	872	6.17
36	85.10	1896	13.41
37	86.10	285	2.02
38	87.10	450	3.18
39	88.20	96	0.68
40	89.20	435	3.08
41	90.10	75	0.53
42	91.10	9345	66.12
43	92.10	1855	13.12
44	93.15	9577	67.76
45	94.15	3816	27.00
46	95.15	12509	88.50
47	96.15	2438	17.25
48	97.15	2706	19.15
49	98.20	686	4.85
50	99.10	716	5.07
51	100.10	193	1.37
52	101.10	226	1.60
53	102.10	236	1.67
54	103.10	1025	7.25
55	104.15	556	3.93
56	105.15	10362	73.31
57	106.15	2880	20.38
58	107.15	9507	67.26
59	108.15	4963	35.11
60	109.15	8501	60.15
61	110.15	1478	10.46
62	111.15	1786	12.64
63	112.20	391	2.77
64	113.20	483	3.42
65	114.10	124	0.88
66	115.10	1781	12.60
67	116.15	725	5.13
68	117.15	2964	20.97
69	118.15	991	7.01
70	119.15	9927	70.23
71	120.15	4437	31.39
72	121.20	7287	51.56
73	122.15	7297	51.63
74	123.20	5091	36.02
75	124.15	920	6.51
76	125.15	985	6.97
77	126.20	304	2.15
78	127.20	610	4.32
79	128.15	1214	8.59
80	129.10	1829	12.94
81	130.15	730	5.16
82	131.10	3314	23.45
83	132.15	1281	9.06
84	133.15	7330	51.86
85	134.15	6833	48.34
86	135.15	6933	49.05
87	136.20	4250	30.07
88	137.25	2039	14.43
89	138.20	441	3.12
90	139.20	571	4.04
91	140.20	134	0.95
92	141.15	812	5.75
93	142.15	747	5.29
94	143.15	1459	10.32
95	144.15	654	4.63
96	145.20	2968	21.00
97	146.20	1122	7.94
98	147.20	5873	41.55
99	148.20	3470	24.55
100	149.20	3378	23.90
101	150.20	920	6.51
102	151.20	752	5.32
103	152.20	396	2.80
104	153.20	487	3.45
105	154.20	202	1.43
106	155.20	596	4.22
107	156.20	429	3.04
108	157.20	1045	7.39
109	158.20	497	3.52
110	159.20	1866	13.20
111	160.15	782	5.53
112	161.20	3843	27.19
113	162.20	2189	15.49
114	163.25	1942	13.74
115	164.30	452	3.20
116	165.15	749	5.30
117	166.20	343	2.43
118	167.20	348	2.46
119	168.20	176	1.25
120	169.20	474	3.35
121	170.20	228	1.61
122	171.15	798	5.65
123	172.20	278	1.97
124	173.25	1283	9.08
125	174.25	503	3.56
126	175.25	4154	29.39
127	176.25	1505	10.65
128	177.20	1629	11.53
129	178.20	518	3.66
130	179.20	506	3.58
131	180.20	167	1.18
132	181.20	213	1.51
133	182.20	58	0.41
134	183.20	363	2.57
135	184.20	101	0.71
136	185.20	584	4.13
137	186.20	281	1.99
138	187.20	1206	8.53
139	188.20	557	3.94
140	189.20	6218	43.99
141	190.20	2072	14.66
142	191.20	1626	11.50
143	192.20	517	3.66
144	193.20	539	3.81
145	194.20	121	0.86
146	195.10	177	1.25
147	196.20	72	0.51
148	197.10	307	2.17
149	198.20	72	0.51
150	199.20	462	3.27
151	200.20	223	1.58
152	201.20	829	5.87
153	202.20	627	4.44
154	203.20	5757	40.73
155	204.25	6060	42.88
156	205.25	2184	15.45
157	206.15	565	4.00
158	207.15	3198	22.63
159	208.10	749	5.30
160	209.10	449	3.18
161	210.10	53	0.37
162	211.10	242	1.71
163	212.10	38	0.27
164	213.10	404	2.86
165	214.10	149	1.05
166	215.10	647	4.58
167	216.10	312	2.21
168	217.25	499	3.53
169	218.25	9215	65.20
170	219.20	1962	13.88
171	220.20	314	2.22
172	221.20	327	2.31
173	222.30	34	0.24
174	223.20	140	0.99
175	224.20	22	0.16
176	225.20	156	1.10
177	226.20	19	0.13
178	227.20	276	1.95
179	228.20	57	0.40
180	229.20	571	4.04
181	230.20	232	1.64
182	231.20	499	3.53
183	232.20	226	1.60
184	233.20	145	1.03
185	234.20	108	0.76
186	235.20	51	0.36
187	237.20	47	0.33
188	238.20	8	0.06
189	239.20	149	1.05
190	240.20	18	0.13
191	241.20	241	1.71
192	242.20	65	0.46
193	243.20	462	3.27
194	244.20	144	1.02
195	245.20	191	1.35
196	246.20	27	0.19
197	247.20	10	0.07
198	248.20	17	0.12
199	249.20	139	0.98
200	250.20	4	0.03
201	251.20	93	0.66
202	252.30	4	0.03
203	253.20	150	1.06
204	254.20	19	0.13
205	255.20	291	2.06
206	256.20	51	0.36
207	257.20	401	2.84
208	258.20	232	1.64
209	259.20	134	0.95
210	265.20	188	1.33
211	266.20	13	0.09
212	267.20	265	1.87
213	268.20	10	0.07
214	269.20	159	1.12
215	270.20	58	0.41
216	271.20	207	1.46
217	272.20	148	1.05
218	273.20	32	0.23
219	274.20	4	0.03
220	279.20	4	0.03
221	281.10	831	5.88
222	282.20	247	1.75
223	283.20	222	1.57
224	284.20	60	0.42
225	285.20	33	0.23
226	286.20	40	0.28
227	287.10	4	0.03
228	290.20	10	0.07

DEPTT. OF BOTANICAL & ENVIRONMENTAL SCIENCES, G.N.D.U. AMRITSAR

#	m/z	Abs. Int.	Rel. Int.
229	293.20	4	0.03
230	295.20	4	0.03
231	296.20	4	0.03
232	297.20	9	0.06
233	298.20	9	0.06
234	299.20	4	0.03
235	304.20	12	0.08
236	305.20	4	0.03
237	306.10	4	0.03
238	311.20	54	0.38
239	313.20	25	0.18
240	327.20	16	0.11
241	333.20	4	0.03
242	341.20	27	0.19
243	342.30	8	0.06

#	m/z	Abs. Int.	Rel. Int.
244	347.20	4	0.03
245	355.20	108	0.76
246	356.20	17	0.12
247	357.20	4	0.03
248	363.20	4	0.03
249	366.10	4	0.03
250	370.20	4	0.03
251	388.20	4	0.03
252	393.10	12	0.08
253	409.20	137	0.97
254	410.20	38	0.27
255	411.20	71	0.50
256	412.20	64	0.45
257	413.20	4	0.03
258	418.20	4	0.03

#	m/z	Abs. Int.	Rel. Int.
259	424.20	171	1.21
260	425.20	23	0.16
261	426.20	22	0.16
262	429.10	8	0.06
263	456.20	8	0.06
264	558.10	4	0.03
265	583.20	8	0.06
266	586.20	4	0.03
267	606.20	4	0.03
268	611.20	4	0.03
269	612.20	4	0.03
270	645.20	4	0.03
271	654.20	4	0.03
272	660.20	4	0.03
273	686.20	4	0.03

Line#:26 R.Time:36.3(Scan#:9692)
MassPeaks:423
RawMode:Averaged 35.9-36.4(9585-9726) BasePeak:218(184365)
BG Mode:None Group 1 - Event 1

#	m/z	Abs. Int.	Rel. Int.
1	50.05	999	0.54
2	51.10	1910	1.04
3	52.15	1132	0.61
4	53.10	16128	8.75
5	54.15	4705	2.55
6	55.10	117164	63.55
7	56.15	15076	8.18
8	57.15	65901	35.74
9	58.10	6746	3.66
10	59.10	8705	4.72
11	60.10	922	0.50
12	61.10	493	0.27
13	62.10	355	0.19
14	63.10	883	0.48
15	64.05	595	0.32
16	65.10	7459	4.05
17	66.15	3444	1.87
18	67.10	77327	41.94
19	68.10	30372	16.47
20	69.10	110783	60.09
21	70.15	13393	7.26
22	71.10	40187	21.80
23	72.10	3883	2.11
24	73.10	4366	2.37
25	74.10	765	0.41
26	75.05	860	0.47
27	76.15	608	0.33
28	77.10	20724	11.24
29	78.10	4718	2.56
30	79.10	63551	34.47
31	80.10	16232	8.80
32	81.10	112451	60.99
33	82.10	27397	14.86
34	83.10	44654	24.22
35	84.15	7963	4.32
36	85.15	17448	9.46
37	86.10	2234	1.21
38	87.10	1080	0.59
39	88.10	515	0.28
40	89.10	723	0.39
41	90.15	628	0.34
42	91.10	59605	32.33
43	92.10	11870	6.44
44	93.10	99154	53.78
45	94.10	38051	20.64
46	95.10	131479	71.31
47	96.10	25631	13.90
48	97.15	34720	18.83
49	98.15	5432	2.95
50	99.20	5729	3.11
51	100.15	1435	0.78
52	101.15	994	0.54
53	102.15	716	0.39
54	103.10	3886	2.11
55	104.15	2228	1.21
56	105.10	73568	39.90
57	106.10	23091	12.52
58	107.10	101965	55.31
59	108.10	50964	27.64

#	m/z	Abs. Int.	Rel. Int.
60	109.15	96472	52.33
61	110.15	18006	9.77
62	111.15	24555	13.32
63	112.20	3875	2.10
64	113.20	3610	1.96
65	114.15	1436	0.78
66	115.10	5698	3.09
67	116.05	2636	1.43
68	117.10	14134	7.67
69	118.15	4677	2.54
70	119.10	88617	48.07
71	120.15	34827	18.89
72	121.10	86436	46.88
73	122.15	81207	44.05
74	123.15	71108	38.57
75	124.15	11051	5.99
76	125.20	11534	6.26
77	126.20	2368	1.28
78	127.15	4002	2.17
79	128.10	4835	2.62
80	129.10	7552	4.10
81	130.15	3610	1.96
82	131.15	21463	11.64
83	132.15	9194	4.99
84	133.15	73885	40.08
85	134.15	47514	25.77
86	135.15	96003	52.07
87	136.15	64130	34.78
88	137.20	27970	15.17
89	138.15	5563	3.02
90	139.15	9605	5.21
91	140.15	2110	1.14
92	141.15	4071	2.21
93	142.10	3501	1.90
94	143.10	7349	3.99
95	144.15	3432	1.86
96	145.10	23356	12.67
97	146.15	9723	5.27
98	147.15	68291	37.04
99	148.20	49973	27.11
100	149.20	49691	26.95
101	150.20	13639	7.40
102	151.20	7066	3.83
103	152.15	4877	2.65
104	153.15	4492	2.44
105	154.15	1650	0.89
106	155.10	2918	1.58
107	156.15	2027	1.10
108	157.15	6020	3.27
109	158.15	2662	1.44
110	159.15	18424	9.99
111	160.20	7495	4.07
112	161.15	56173	30.47
113	162.20	28261	15.33
114	163.20	25763	13.97
115	164.20	4801	2.60
116	165.20	3773	2.05
117	166.20	2013	1.09
118	167.25	1906	1.03

#	m/z	Abs. Int.	Rel. Int.
119	168.15	924	0.50
120	169.15	2108	1.14
121	170.15	990	0.54
122	171.15	4662	2.53
123	172.15	1923	1.04
124	173.15	14347	7.78
125	174.15	4773	2.59
126	175.20	37219	20.19
127	176.20	12392	6.72
128	177.20	13175	7.15
129	178.20	3680	2.00
130	179.15	5266	2.86
131	180.15	1330	0.72
132	181.25	1247	0.68
133	182.15	598	0.32
134	183.15	1501	0.81
135	184.10	629	0.34
136	185.15	3416	1.85
137	186.15	1941	1.05
138	187.15	17519	9.50
139	188.15	6682	3.62
140	189.15	91766	49.77
141	190.15	33846	18.36
142	191.15	22503	12.21
143	192.15	5135	2.79
144	193.15	3343	1.81
145	194.20	1079	0.59
146	195.15	991	0.54
147	196.20	436	0.24
148	197.10	1189	0.64
149	198.15	493	0.27
150	199.10	2707	1.47
151	200.10	1093	0.59
152	201.15	12456	6.76
153	202.15	5267	2.86
154	203.20	70775	38.39
155	204.20	25969	14.09
156	205.15	15319	8.31
157	206.15	10703	5.81
158	207.15	50422	27.35
159	208.15	13736	7.45
160	209.20	2510	1.36
161	210.10	541	0.29
162	211.10	987	0.54
163	212.15	425	0.23
164	213.15	2774	1.50
165	214.15	1096	0.59
166	215.15	7193	3.90
167	216.15	5979	3.24
168	217.15	9081	4.93
169	218.20	184365	100.00
170	219.20	38230	20.74
171	220.15	8476	4.60
172	221.15	3286	1.78
173	222.15	1101	0.60
174	223.15	630	0.34
175	224.20	268	0.15
176	225.15	771	0.42
177	226.15	335	0.18

DEPTT. OF BOTANICAL & ENVIRONMENTAL SCIENCES, G.N.D.U. AMRITSAR

#	m/z	Abs. Int.	Rel. Int.	#	m/z	Abs. Int.	Rel. Int.	#	m/z	Abs. Int.	Rel. Int.
178	227.15	2152	1.17	258	307.20	11	0.01	338	393.30	2464	1.34
179	228.20	980	0.53	259	308.10	11	0.01	339	394.30	837	0.45
180	229.15	9321	5.06	260	309.20	301	0.16	340	395.30	183	0.10
181	230.15	3025	1.64	261	310.20	99	0.05	341	396.30	84	0.05
182	231.15	5872	3.18	262	311.20	432	0.23	342	397.30	195	0.11
183	232.20	2589	1.40	263	312.25	230	0.12	343	398.30	92	0.05
184	233.15	1689	0.92	264	313.20	1802	0.98	344	399.30	12	0.01
185	234.15	5615	3.05	265	314.25	952	0.52	345	401.30	4	0.00
186	235.15	1292	0.70	266	315.25	3683	2.00	346	402.30	1	0.00
187	236.10	320	0.17	267	316.25	2090	1.13	347	403.40	1	0.00
188	237.20	389	0.21	268	317.25	978	0.53	348	404.40	8	0.00
189	238.20	181	0.10	269	318.20	231	0.13	349	406.30	80	0.04
190	239.15	731	0.40	270	319.20	38	0.02	350	407.30	114	0.06
191	240.10	308	0.17	271	320.30	18	0.01	351	408.30	1205	0.65
192	241.15	1780	0.97	272	321.30	19	0.01	352	409.30	618	0.34
193	242.15	865	0.47	273	322.30	4	0.00	353	410.30	176	0.10
194	243.15	4849	2.63	274	323.25	413	0.22	354	411.30	5187	2.81
195	244.20	1629	0.88	275	324.20	231	0.13	355	412.30	1721	0.93
196	245.15	2638	1.43	276	325.25	614	0.33	356	413.30	293	0.16
197	246.20	1420	0.77	277	326.30	379	0.21	357	414.30	75	0.04
198	247.15	3058	1.66	278	327.25	330	0.18	358	415.30	2	0.00
199	248.15	1324	0.72	279	328.30	144	0.08	359	416.10	4	0.00
200	249.10	580	0.31	280	329.30	226	0.12	360	417.30	2	0.00
201	250.20	248	0.13	281	330.30	93	0.05	361	418.40	9	0.00
202	251.20	390	0.21	282	331.30	54	0.03	362	419.40	1	0.00
203	252.20	193	0.10	283	334.20	4	0.00	363	420.30	1	0.00
204	253.15	683	0.37	284	335.30	6	0.00	364	421.30	3	0.00
205	254.20	271	0.15	285	336.30	8	0.00	365	422.30	25	0.01
206	255.20	3068	1.66	286	337.25	507	0.27	366	423.30	14	0.01
207	256.15	1054	0.57	287	338.30	218	0.12	367	424.30	274	0.15
208	257.20	11257	6.11	288	339.25	567	0.31	368	425.40	154	0.08
209	258.20	3311	1.80	289	340.30	344	0.19	369	426.35	8706	4.72
210	259.25	5808	3.15	290	341.25	623	0.34	370	427.35	2852	1.55
211	260.20	1417	0.77	291	342.30	575	0.31	371	428.35	521	0.28
212	261.20	431	0.23	292	343.25	607	0.33	372	429.30	90	0.05
213	262.20	106	0.06	293	344.25	610	0.33	373	430.30	28	0.02
214	263.20	91	0.05	294	345.25	167	0.09	374	431.40	4	0.00
215	264.20	135	0.07	295	346.20	19	0.01	375	432.40	32	0.02
216	265.10	485	0.26	296	347.20	7	0.00	376	433.40	9	0.00
217	266.20	228	0.12	297	348.30	6	0.00	377	435.30	39	0.02
218	267.10	833	0.45	298	349.20	2	0.00	378	436.40	8	0.00
219	268.20	287	0.16	299	350.20	11	0.01	379	440.40	6	0.00
220	269.15	1724	0.94	300	351.25	253	0.14	380	441.30	3	0.00
221	270.20	1392	0.76	301	352.30	121	0.07	381	445.30	1	0.00
222	271.20	3189	1.73	302	353.30	138	0.07	382	449.30	1	0.00
223	272.20	5399	2.93	303	354.25	101	0.05	383	450.40	66	0.04
224	273.20	2570	1.39	304	355.20	544	0.30	384	451.40	21	0.01
225	274.30	2734	1.48	305	356.25	259	0.14	385	452.40	3	0.00
226	275.30	940	0.51	306	357.25	580	0.31	386	456.40	1	0.00
227	276.20	262	0.14	307	358.30	279	0.15	387	462.10	1	0.00
228	277.20	88	0.05	308	359.20	51	0.03	388	465.40	1	0.00
229	278.20	83	0.05	309	360.30	3	0.00	389	466.40	84	0.05
230	279.30	96	0.05	310	361.20	2	0.00	390	467.40	16	0.01
231	280.20	52	0.03	311	362.20	4	0.00	391	468.10	2	0.00
232	281.05	2316	1.26	312	363.20	16	0.01	392	472.20	1	0.00
233	282.05	738	0.40	313	364.30	6	0.00	393	475.40	1	0.00
234	283.20	1393	0.76	314	365.30	1449	0.79	394	480.00	1	0.00
235	284.20	792	0.43	315	366.20	473	0.26	395	484.10	1	0.00
236	285.20	1139	0.62	316	367.25	167	0.09	396	486.10	1	0.00
237	286.25	2738	1.49	317	368.30	170	0.09	397	495.40	1	0.00
238	287.20	1130	0.61	318	369.25	415	0.23	398	500.40	1	0.00
239	288.25	642	0.35	319	370.30	952	0.52	399	514.10	1	0.00
240	289.20	233	0.13	320	371.30	272	0.15	400	533.30	1	0.00
241	290.20	100	0.05	321	372.30	46	0.02	401	537.10	1	0.00
242	291.30	39	0.02	322	375.30	1	0.00	402	539.40	1	0.00
243	292.10	38	0.02	323	377.30	1	0.00	403	544.40	1	0.00
244	293.20	57	0.03	324	378.30	13	0.01	404	545.40	1	0.00
245	294.20	12	0.01	325	379.30	77	0.04	405	554.30	1	0.00
246	295.20	547	0.30	326	380.30	34	0.02	406	556.10	1	0.00
247	296.20	313	0.17	327	381.30	73	0.04	407	558.40	1	0.00
248	297.25	2753	1.49	328	382.30	28	0.02	408	569.40	1	0.00
249	298.25	1200	0.65	329	383.30	1149	0.62	409	607.30	1	0.00
250	299.25	1622	0.88	330	384.25	381	0.21	410	608.40	3	0.00
251	300.25	1414	0.77	331	385.20	73	0.04	411	612.10	1	0.00
252	301.20	850	0.46	332	386.30	1	0.00	412	616.10	1	0.00
253	302.20	430	0.23	333	387.30	3	0.00	413	622.30	1	0.00
254	303.20	146	0.08	334	389.30	13	0.01	414	637.10	3	0.00
255	304.20	11	0.01	335	390.30	50	0.03	415	641.10	1	0.00
256	305.30	4	0.00	336	391.30	134	0.07	416	647.30	3	0.00
257	306.20	15	0.01	337	392.30	52	0.03	417	648.40	1	0.00

DEPTT. OF BOTANICAL & ENVIRONMENTAL SCIENCES, G.N.D.U. AMRITSAR

#	m/z	Abs. Int.	Rel. Int.
418	652.40	1	0.00
419	654.40	4	0.00
420	675.40	1	0.00
421	676.10	1	0.00
422	695.40	1	0.00
423	700.20	1	0.00

Line#:27 R.Time:37.2(Scan#:9958)
MassPeaks:347
RawMode:Averaged 37.1-37.3(9928-9989) BasePeak:69(31129)
BG Mode:None Group 1 - Event 1

#	m/z	Abs. Int.	Rel. Int.
1	50.15	757	2.43
2	51.10	1145	3.68
3	52.15	735	2.36
4	53.15	4096	13.16
5	54.15	1430	4.59
6	55.15	26481	85.07
7	56.10	3334	10.71
8	57.15	14023	45.05
9	58.15	1048	3.37
10	59.15	1013	3.25
11	60.10	827	2.66
12	61.10	622	2.00
13	62.10	261	0.84
14	63.10	601	1.93
15	64.10	429	1.38
16	65.10	2137	6.86
17	66.10	904	2.90
18	67.10	19228	61.77
19	68.15	5177	16.63
20	69.10	31129	100.00
21	70.15	3371	10.83
22	71.10	5701	18.31
23	72.15	722	2.32
24	73.10	5940	19.08
25	74.10	862	2.77
26	75.10	1305	4.19
27	76.10	484	1.55
28	77.10	5310	17.06
29	78.10	1337	4.30
30	79.10	12276	39.44
31	80.10	2544	8.17
32	81.10	24843	79.81
33	82.10	8330	26.76
34	83.10	12121	38.94
35	84.15	1800	5.78
36	85.10	3421	10.99
37	86.10	593	1.90
38	87.10	729	2.34
39	88.10	435	1.40
40	89.10	698	2.24
41	90.10	185	0.59
42	91.10	10915	35.06
43	92.10	2037	6.54
44	93.10	14765	47.43
45	94.10	5392	17.32
46	95.10	28288	90.87
47	96.15	12724	40.88
48	97.15	7533	24.20
49	98.15	1298	4.17
50	99.10	1348	4.33
51	100.20	259	0.83
52	101.10	428	1.37
53	102.10	393	1.26
54	103.10	1366	4.39
55	104.10	631	2.03
56	105.15	10946	35.16
57	106.15	2736	8.79
58	107.15	15099	48.50
59	108.15	6457	20.74
60	109.15	21178	68.03
61	110.15	5175	16.62
62	111.15	6454	20.73
63	112.15	933	3.00
64	113.15	1068	3.43
65	114.10	249	0.80
66	115.10	1976	6.35
67	116.10	837	2.69
68	117.10	2889	9.28
69	118.15	979	3.14
70	119.15	11175	35.90
71	120.15	3891	12.50
72	121.15	13136	42.20

#	m/z	Abs. Int.	Rel. Int.
73	122.15	5078	16.31
74	123.15	15517	49.85
75	124.20	5150	16.54
76	125.20	8798	28.26
77	126.20	1271	4.08
78	127.20	1170	3.76
79	128.10	1187	3.81
80	129.15	1927	6.19
81	130.15	737	2.37
82	131.10	3530	11.34
83	132.15	1460	4.69
84	133.15	9565	30.73
85	134.15	4489	14.42
86	135.15	9782	31.42
87	136.15	5197	16.70
88	137.20	6645	21.35
89	138.20	2065	6.63
90	139.20	1223	3.93
91	140.20	294	0.94
92	141.10	911	2.93
93	142.15	693	2.23
94	143.15	1593	5.12
95	144.15	685	2.20
96	145.15	3945	12.67
97	146.20	1787	5.74
98	147.15	7422	23.84
99	148.20	3456	11.10
100	149.20	6360	20.43
101	150.20	2752	8.84
102	151.20	2466	7.92
103	152.20	1278	4.11
104	153.20	1018	3.27
105	154.20	415	1.33
106	155.15	724	2.33
107	156.10	464	1.49
108	157.15	1361	4.37
109	158.15	650	2.09
110	159.20	3199	10.28
111	160.15	1852	5.95
112	161.20	6259	20.11
113	162.20	2426	7.79
114	163.20	6383	20.50
115	164.20	2590	8.32
116	165.20	3455	11.10
117	166.25	756	2.43
118	167.20	499	1.60
119	168.20	280	0.90
120	169.20	564	1.81
121	170.20	269	0.86
122	171.15	1202	3.86
123	172.25	576	1.85
124	173.20	2529	8.12
125	174.20	1008	3.24
126	175.20	4521	14.52
127	176.20	1764	5.67
128	177.20	2737	8.79
129	178.25	1071	3.44
130	179.20	3070	9.86
131	180.25	668	2.15
132	181.20	353	1.13
133	182.20	185	0.59
134	183.20	459	1.47
135	184.20	215	0.69
136	185.15	1141	3.67
137	186.20	484	1.55
138	187.20	2347	7.54
139	188.20	881	2.83
140	189.20	6766	21.74
141	190.20	2316	7.44
142	191.20	5366	17.24
143	192.20	1325	4.26
144	193.15	1685	5.41

#	m/z	Abs. Int.	Rel. Int.
145	194.20	450	1.45
146	195.20	401	1.29
147	196.10	86	0.28
148	197.10	391	1.26
149	198.10	114	0.37
150	199.20	963	3.09
151	200.25	365	1.17
152	201.15	1728	5.55
153	202.20	902	2.90
154	203.20	9722	31.23
155	204.20	3476	11.17
156	205.20	4532	14.56
157	206.15	1620	5.20
158	207.05	7934	25.49
159	208.10	1806	5.80
160	209.05	1106	3.55
161	210.10	257	0.83
162	211.10	373	1.20
163	212.10	86	0.28
164	213.15	796	2.56
165	214.15	386	1.24
166	215.20	1866	5.99
167	216.20	761	2.44
168	217.20	2007	6.45
169	218.20	11790	37.87
170	219.20	2845	9.14
171	220.20	1205	3.87
172	221.15	973	3.13
173	222.20	321	1.03
174	223.10	324	1.04
175	224.20	87	0.28
176	225.10	289	0.93
177	226.20	73	0.23
178	227.15	554	1.78
179	228.20	246	0.79
180	229.20	2015	6.47
181	230.20	725	2.33
182	231.20	2469	7.93
183	232.20	1463	4.70
184	233.20	938	3.01
185	234.20	671	2.16
186	235.20	313	1.01
187	236.20	51	0.16
188	237.20	155	0.50
189	238.20	39	0.13
190	239.20	208	0.67
191	240.20	36	0.12
192	241.20	891	2.86
193	242.20	254	0.82
194	243.20	641	2.06
195	244.20	271	0.87
196	245.20	1014	3.26
197	246.20	1762	5.66
198	247.25	1457	4.68
199	248.20	702	2.26
200	249.10	695	2.23
201	250.20	156	0.50
202	251.10	301	0.97
203	252.30	58	0.19
204	253.10	280	0.90
205	254.30	30	0.10
206	255.25	1030	3.31
207	256.20	265	0.85
208	257.20	1128	3.62
209	258.25	366	1.18
210	259.20	1183	3.80
211	260.20	309	0.99
212	261.25	321	1.03
213	262.20	82	0.26
214	263.20	24	0.08
215	264.20	3	0.01
216	265.20	623	2.00

#	m/z	Abs. Int.	Rel. Int.
217	266.20	120	0.39
218	267.05	671	2.16
219	268.20	209	0.67
220	269.25	585	1.88
221	270.25	291	0.93
222	271.25	456	1.46
223	272.35	232	0.75
224	273.25	1609	5.17
225	274.25	954	3.06
226	275.25	365	1.17
227	276.20	87	0.28
228	277.20	7	0.02
229	278.20	10	0.03
230	279.10	16	0.05
231	280.10	3	0.01
232	281.05	2585	8.30
233	282.10	773	2.48
234	283.10	613	1.97
235	284.10	183	0.59
236	285.25	307	0.99
237	286.10	130	0.42
238	287.25	424	1.36
239	288.30	175	0.56
240	289.30	52	0.17
241	290.00	10	0.03
242	291.30	7	0.02
243	293.10	14	0.04
244	295.30	109	0.35
245	297.30	94	0.30
246	298.20	25	0.08
247	299.30	70	0.22
248	300.10	45	0.14
249	301.30	69	0.22
250	302.30	460	1.48
251	303.30	209	0.67
252	304.30	15	0.05
253	305.10	7	0.02
254	309.30	3	0.01
255	310.10	6	0.02
256	311.10	26	0.08
257	312.30	7	0.02
258	313.30	6	0.02
259	315.10	3	0.01
260	316.30	14	0.04

#	m/z	Abs. Int.	Rel. Int.
261	323.30	26	0.08
262	325.30	106	0.34
263	327.30	145	0.47
264	328.40	13	0.04
265	329.30	3	0.01
266	331.30	3	0.01
267	335.10	3	0.01
268	337.10	3	0.01
269	338.10	3	0.01
270	339.30	54	0.17
271	340.10	6	0.02
272	341.35	468	1.50
273	342.30	145	0.47
274	343.10	38	0.12
275	344.30	35	0.11
276	347.30	3	0.01
277	353.40	7	0.02
278	354.40	3	0.01
279	355.30	399	1.28
280	356.10	126	0.40
281	357.10	24	0.08
282	358.10	3	0.01
283	364.10	6	0.02
284	365.30	30	0.10
285	366.10	3	0.01
286	367.30	6	0.02
287	368.30	11	0.04
288	369.30	20	0.06
289	370.10	10	0.03
290	373.10	3	0.01
291	375.10	3	0.01
292	377.40	3	0.01
293	379.30	26	0.08
294	380.30	4	0.01
295	381.30	3	0.01
296	384.30	19	0.06
297	387.10	3	0.01
298	391.30	19	0.06
299	392.30	3	0.01
300	393.40	155	0.50
301	394.30	58	0.19
302	395.10	3	0.01
303	397.10	10	0.03
304	401.30	3	0.01

#	m/z	Abs. Int.	Rel. Int.
305	404.30	6	0.02
306	405.30	6	0.02
307	407.40	47	0.15
308	408.40	85	0.27
309	409.30	43	0.14
310	410.30	7	0.02
311	411.35	429	1.38
312	412.30	146	0.47
313	413.30	10	0.03
314	415.10	8	0.03
315	416.10	3	0.01
316	424.10	6	0.02
317	425.40	22	0.07
318	426.40	279	0.90
319	427.40	152	0.49
320	428.10	18	0.06
321	429.10	62	0.20
322	430.10	118	0.38
323	431.10	68	0.22
324	434.30	3	0.01
325	440.10	3	0.01
326	453.30	21	0.07
327	454.40	6	0.02
328	457.10	3	0.01
329	468.40	49	0.16
330	470.10	3	0.01
331	473.10	7	0.02
332	486.10	3	0.01
333	493.40	3	0.01
334	535.40	3	0.01
335	557.10	3	0.01
336	558.10	3	0.01
337	573.10	3	0.01
338	594.10	3	0.01
339	595.30	3	0.01
340	609.10	3	0.01
341	620.10	6	0.02
342	633.40	3	0.01
343	660.10	3	0.01
344	671.10	3	0.01
345	680.10	3	0.01
346	686.10	3	0.01
347	689.10	3	0.01

Line#:28 R.Time:38.0(Scan#:10191)
MassPeaks:419
RawMode:Averaged 37.7-38.0(10099-10207) BasePeak:69(202219)
BG Mode:None Group 1 - Event 1

#	m/z	Abs. Int.	Rel. Int.
1	50.05	1038	0.51
2	51.10	1877	0.93
3	52.15	1214	0.60
4	53.10	18865	9.33
5	54.15	6517	3.22
6	55.10	143097	70.76
7	56.10	14508	7.17
8	57.15	63327	31.32
9	58.15	3354	1.66
10	59.15	1988	0.98
11	60.10	1007	0.50
12	61.05	936	0.46
13	62.10	366	0.18
14	63.10	779	0.39
15	64.15	585	0.29
16	65.05	7643	3.78
17	66.15	3638	1.80
18	67.10	121445	60.06
19	68.10	32969	16.30
20	69.10	202219	100.00
21	70.10	17572	8.69
22	71.10	15185	7.51
23	72.10	2219	1.10
24	73.10	7689	3.80
25	74.10	1443	0.71
26	75.15	2297	1.14
27	76.15	657	0.32
28	77.05	17718	8.76
29	78.15	3892	1.92
30	79.10	59258	29.30

#	m/z	Abs. Int.	Rel. Int.
31	80.10	11153	5.52
32	81.10	143672	71.05
33	82.10	63718	31.51
34	83.10	78226	38.68
35	84.10	8380	4.14
36	85.10	8635	4.27
37	86.10	2284	1.13
38	87.10	1288	0.64
39	88.10	1503	0.74
40	89.10	1549	0.77
41	90.15	462	0.23
42	91.10	43449	21.49
43	92.10	7801	3.86
44	93.10	78509	38.82
45	94.15	22808	11.28
46	95.10	185260	91.61
47	96.10	109357	54.08
48	97.10	43283	21.40
49	98.15	5360	2.65
50	99.10	4141	2.05
51	100.15	1075	0.53
52	101.10	1226	0.61
53	102.15	1522	0.75
54	103.05	3226	1.60
55	104.15	1498	0.74
56	105.10	43388	21.46
57	106.10	11694	5.78
58	107.10	85473	42.27
59	108.15	39469	19.52
60	109.20	154307	76.31

#	m/z	Abs. Int.	Rel. Int.
61	110.10	41331	20.44
62	111.15	41700	20.62
63	112.15	4332	2.14
64	113.15	4174	2.06
65	114.15	1256	0.62
66	115.10	4104	2.03
67	116.05	2242	1.11
68	117.10	8140	4.03
69	118.10	2931	1.45
70	119.10	47306	23.39
71	120.10	20154	9.97
72	121.15	80363	39.74
73	122.15	32782	16.21
74	123.15	132208	65.38
75	124.15	43627	21.57
76	125.15	98639	48.78
77	126.15	10570	5.23
78	127.15	4810	2.38
79	128.10	3015	1.49
80	129.10	4368	2.16
81	130.10	2030	1.00
82	131.10	11269	5.57
83	132.15	8135	4.02
84	133.10	43861	21.69
85	134.10	22452	11.10
86	135.15	57886	28.63
87	136.15	28859	14.27
88	137.15	50461	24.95
89	138.15	16904	8.36
90	139.15	7423	3.67

DEPTT. OF BOTANICAL & ENVIRONMENTAL SCIENCES, G.N.D.U. AMRITSAR

#	m/z	Abs. Int.	Rel. Int.
91	140.15	1405	0.69
92	141.15	2230	1.10
93	142.15	2023	1.00
94	143.10	4841	2.39
95	144.15	2266	1.12
96	145.10	17543	8.68
97	146.15	9306	4.60
98	147.15	35941	17.77
99	148.15	19086	9.44
100	149.15	43758	21.64
101	150.15	23690	11.72
102	151.20	19310	9.55
103	152.20	10102	5.00
104	153.15	3426	1.69
105	154.15	1072	0.53
106	155.15	1537	0.76
107	156.15	1129	0.56
108	157.15	4500	2.23
109	158.15	2318	1.15
110	159.15	14813	7.33
111	160.15	12877	6.37
112	161.15	43932	21.72
113	162.15	14645	7.24
114	163.15	54552	26.98
115	164.15	17804	8.80
116	165.20	10455	5.17
117	166.15	2027	1.00
118	167.15	1145	0.57
119	168.15	540	0.27
120	169.15	1157	0.57
121	170.15	712	0.35
122	171.15	4181	2.07
123	172.15	2841	1.40
124	173.15	12844	6.35
125	174.15	4804	2.38
126	175.15	23402	11.57
127	176.15	10241	5.06
128	177.20	19201	9.50
129	178.20	6527	3.23
130	179.15	34393	17.01
131	180.15	5548	2.74
132	181.20	1156	0.57
133	182.15	414	0.20
134	183.10	992	0.49
135	184.15	465	0.23
136	185.10	4965	2.46
137	186.10	2187	1.08
138	187.15	12674	6.27
139	188.15	4310	2.13
140	189.15	28321	14.01
141	190.15	12252	6.06
142	191.15	39080	19.33
143	192.15	10225	5.06
144	193.10	10016	4.95
145	194.15	3169	1.57
146	195.15	1260	0.62
147	196.20	321	0.16
148	197.10	802	0.40
149	198.15	402	0.20
150	199.10	4754	2.35
151	200.15	1946	0.96
152	201.10	9360	4.63
153	202.15	6034	2.98
154	203.15	20408	10.09
155	204.15	21743	10.75
156	205.15	39320	19.44
157	206.15	14543	7.19
158	207.10	18891	9.34
159	208.10	4555	2.25
160	209.10	2178	1.08
161	210.00	491	0.24
162	211.10	662	0.33
163	212.10	293	0.14
164	213.10	3641	1.80
165	214.15	2341	1.16
166	215.15	15448	7.64
167	216.15	4984	2.46
168	217.15	21640	10.70
169	218.15	46291	22.89
170	219.10	14136	6.99

#	m/z	Abs. Int.	Rel. Int.
171	220.15	13554	6.70
172	221.15	4893	2.42
173	222.15	1385	0.68
174	223.10	607	0.30
175	224.10	153	0.08
176	225.15	492	0.24
177	226.15	206	0.10
178	227.10	2340	1.16
179	228.15	1444	0.71
180	229.10	11381	5.63
181	230.15	4691	2.32
182	231.10	30146	14.91
183	232.10	20618	10.20
184	233.15	11759	5.81
185	234.15	9110	4.51
186	235.20	2292	1.13
187	236.20	441	0.22
188	237.20	352	0.17
189	238.20	99	0.05
190	239.10	497	0.25
191	240.10	152	0.08
192	241.10	2263	1.12
193	242.15	867	0.43
194	243.15	2637	1.30
195	244.15	1839	0.91
196	245.15	11795	5.83
197	246.10	25931	12.82
198	247.15	10177	5.03
199	248.15	8275	4.09
200	249.15	2592	1.28
201	250.10	507	0.25
202	251.00	575	0.28
203	252.10	124	0.06
204	253.20	496	0.25
205	254.25	194	0.10
206	255.15	7046	3.48
207	256.15	2080	1.03
208	257.15	9929	4.91
209	258.15	2468	1.22
210	259.15	6913	3.42
211	260.15	2197	1.09
212	261.20	3163	1.56
213	262.20	1078	0.53
214	263.20	423	0.21
215	264.00	103	0.05
216	264.95	1101	0.54
217	266.00	365	0.18
218	267.00	1251	0.62
219	268.15	388	0.19
220	269.20	3071	1.52
221	270.20	1367	0.68
222	271.20	2767	1.37
223	272.25	1922	0.95
224	273.20	28695	14.19
225	274.20	14548	7.19
226	275.20	5985	2.96
227	276.20	1071	0.53
228	277.20	194	0.10
229	278.20	43	0.02
230	279.30	101	0.05
231	280.00	33	0.02
232	281.00	4846	2.40
233	282.00	1405	0.69
234	283.10	1401	0.69
235	284.20	1295	0.64
236	285.20	2171	1.07
237	286.20	1883	0.93
238	287.20	7681	3.80
239	288.20	2171	1.07
240	289.20	546	0.27
241	290.20	101	0.05
242	291.20	41	0.02
243	292.20	21	0.01
244	293.20	71	0.04
245	294.20	6	0.00
246	295.20	315	0.16
247	296.20	95	0.05
248	297.25	449	0.22
249	298.20	189	0.09
250	299.25	766	0.38

#	m/z	Abs. Int.	Rel. Int.
251	300.20	485	0.24
252	301.20	670	0.33
253	302.20	11038	5.46
254	303.25	4360	2.16
255	304.20	806	0.40
256	305.20	136	0.07
257	306.20	14	0.01
258	307.20	47	0.02
259	308.20	6	0.00
260	309.20	129	0.06
261	310.20	46	0.02
262	311.20	209	0.10
263	312.20	74	0.04
264	313.25	362	0.18
265	314.20	134	0.07
266	315.25	334	0.17
267	316.20	135	0.07
268	317.20	89	0.04
269	318.20	7	0.00
270	319.30	14	0.01
271	320.30	6	0.00
272	321.20	29	0.01
273	322.20	6	0.00
274	323.20	157	0.08
275	324.20	63	0.03
276	325.20	368	0.18
277	326.20	161	0.08
278	327.25	776	0.38
279	328.25	264	0.13
280	329.25	253	0.13
281	330.20	74	0.04
282	331.20	88	0.04
283	332.20	10	0.00
284	333.30	3	0.00
285	334.30	9	0.00
286	335.30	15	0.01
287	336.30	5	0.00
288	337.30	82	0.04
289	338.20	31	0.02
290	339.20	529	0.26
291	340.25	238	0.12
292	341.30	5310	2.63
293	342.25	1538	0.76
294	343.15	399	0.20
295	344.25	943	0.47
296	345.35	260	0.13
297	346.30	16	0.01
298	347.30	1	0.00
299	348.30	1	0.00
300	349.20	7	0.00
301	351.30	29	0.01
302	352.30	12	0.01
303	353.30	108	0.05
304	354.15	299	0.15
305	355.05	932	0.46
306	356.20	381	0.19
307	357.20	309	0.15
308	358.10	97	0.05
309	359.10	12	0.01
310	360.10	6	0.00
311	361.20	3	0.00
312	362.10	1	0.00
313	363.20	16	0.01
314	364.10	2	0.00
315	365.30	160	0.08
316	366.30	24	0.01
317	367.20	27	0.01
318	368.30	33	0.02
319	369.25	254	0.13
320	370.10	141	0.07
321	371.20	72	0.04
322	372.10	6	0.00
323	375.20	6	0.00
324	377.30	29	0.01
325	378.20	7	0.00
326	379.20	98	0.05
327	380.30	33	0.02
328	381.30	54	0.03
329	382.10	17	0.01
330	383.25	273	0.14

DEPTT. OF BOTANICAL & ENVIRONMENTAL SCIENCES, G.N.D.U. AMRITSAR

#	m/z	Abs. Int.	Rel. Int.
331	384.25	461	0.23
332	385.20	139	0.07
333	386.30	8	0.00
334	387.10	2	0.00
335	389.20	7	0.00
336	390.30	14	0.01
337	391.20	60	0.03
338	392.20	22	0.01
339	393.30	635	0.31
340	394.30	232	0.11
341	395.30	134	0.07
342	396.30	73	0.04
343	397.30	176	0.09
344	398.30	66	0.03
345	399.30	41	0.02
346	400.10	7	0.00
347	401.10	20	0.01
348	402.10	1	0.00
349	403.20	1	0.00
350	405.30	11	0.01
351	406.30	33	0.02
352	407.30	65	0.03
353	408.30	392	0.19
354	409.30	372	0.18
355	410.35	311	0.15
356	411.25	6204	3.07
357	412.30	2043	1.01
358	413.30	363	0.18
359	414.30	46	0.02
360	415.30	12	0.01

#	m/z	Abs. Int.	Rel. Int.
361	416.30	5	0.00
362	418.30	4	0.00
363	420.30	3	0.00
364	422.30	41	0.02
365	423.30	101	0.05
366	424.30	214	0.11
367	425.30	145	0.07
368	426.30	7571	3.74
369	427.30	2585	1.28
370	428.35	517	0.26
371	429.30	242	0.12
372	430.40	37	0.02
373	431.30	15	0.01
374	433.30	1	0.00
375	437.30	4	0.00
376	438.30	17	0.01
377	439.30	10	0.00
378	440.40	46	0.02
379	441.30	21	0.01
380	442.30	13	0.01
381	444.10	4	0.00
382	446.30	12	0.01
383	449.10	1	0.00
384	450.10	2	0.00
385	453.40	59	0.03
386	454.40	13	0.01
387	455.30	3	0.00
388	458.30	1	0.00
389	460.30	4	0.00
390	463.40	1	0.00

#	m/z	Abs. Int.	Rel. Int.
391	468.40	105	0.05
392	469.20	35	0.02
393	470.40	8	0.00
394	472.10	3	0.00
395	475.40	1	0.00
396	484.10	1	0.00
397	489.40	1	0.00
398	505.30	4	0.00
399	515.40	1	0.00
400	523.40	1	0.00
401	531.40	2	0.00
402	539.30	3	0.00
403	561.10	1	0.00
404	563.20	1	0.00
405	570.10	1	0.00
406	576.30	1	0.00
407	577.10	1	0.00
408	580.30	1	0.00
409	582.30	4	0.00
410	593.30	1	0.00
411	594.10	1	0.00
412	609.40	1	0.00
413	621.40	1	0.00
414	628.10	1	0.00
415	637.40	1	0.00
416	653.40	2	0.00
417	655.10	1	0.00
418	659.30	1	0.00
419	667.40	3	0.00

Line#:29 R.Time:39.9(Scan#:10769)
MassPeaks:399
RawMode:Averaged 39.8-40.0(10735-10805) BasePeak:203(53655)
BG Mode:None Group 1 - Event 1

#	m/z	Abs. Int.	Rel. Int.
1	50.10	822	1.53
2	51.15	1083	2.02
3	52.10	730	1.36
4	53.10	3144	5.86
5	54.15	1156	2.15
6	55.15	20736	38.65
7	56.15	2720	5.07
8	57.10	13093	24.40
9	58.10	1388	2.59
10	59.10	2458	4.58
11	60.10	1093	2.04
12	61.10	1185	2.21
13	62.10	315	0.59
14	63.10	674	1.26
15	64.15	504	0.94
16	65.10	2017	3.76
17	66.10	917	1.71
18	67.10	12700	23.67
19	68.10	2752	5.13
20	69.10	18715	34.88
21	70.15	2588	4.82
22	71.10	8121	15.14
23	72.15	1188	2.21
24	73.10	17927	33.41
25	74.10	2024	3.77
26	75.10	3062	5.71
27	76.10	680	1.27
28	77.10	5620	10.47
29	78.10	1570	2.93
30	79.10	11993	22.35
31	80.10	2650	4.94
32	81.10	19581	36.49
33	82.10	4738	8.83
34	83.10	8764	16.33
35	84.15	1863	3.47
36	85.10	5019	9.35
37	86.20	705	1.31
38	87.10	1631	3.04
39	88.05	706	1.32
40	89.00	1532	2.86
41	90.10	413	0.77
42	91.10	13385	24.95
43	92.10	2461	4.59
44	93.10	14903	27.78

#	m/z	Abs. Int.	Rel. Int.
45	94.10	4314	8.04
46	95.15	21245	39.60
47	96.10	10271	19.14
48	97.15	7256	13.52
49	98.15	1810	3.37
50	99.15	1903	3.55
51	100.10	891	1.66
52	101.15	751	1.40
53	102.10	566	1.05
54	103.10	2840	5.29
55	104.10	1204	2.24
56	105.10	15966	29.76
57	106.15	4088	7.62
58	107.15	15360	28.63
59	108.10	4299	8.01
60	109.15	13192	24.59
61	110.15	2801	5.22
62	111.15	4043	7.54
63	112.15	1000	1.86
64	113.15	1240	2.31
65	114.10	379	0.71
66	115.10	3417	6.37
67	116.10	1370	2.55
68	117.15	7027	13.10
69	118.10	2951	5.50
70	119.15	18404	34.30
71	120.15	6056	11.29
72	121.15	12562	23.41
73	122.15	4387	8.18
74	123.15	8138	15.17
75	124.15	1728	3.22
76	125.15	3308	6.17
77	126.10	1050	1.96
78	127.15	1604	2.99
79	128.10	1867	3.48
80	129.10	3468	6.46
81	130.15	1405	2.62
82	131.15	9030	16.83
83	132.15	4274	7.97
84	133.15	46047	85.82
85	134.15	8370	15.60
86	135.15	11024	20.55
87	136.15	3724	6.94
88	137.20	3668	6.84

#	m/z	Abs. Int.	Rel. Int.
89	138.15	1034	1.93
90	139.15	1983	3.70
91	140.15	626	1.17
92	141.10	1602	2.99
93	142.15	1372	2.56
94	143.15	3123	5.82
95	144.15	1265	2.36
96	145.20	7242	13.50
97	146.15	3297	6.14
98	147.15	12091	22.53
99	148.20	3782	7.05
100	149.15	5754	10.72
101	150.15	1572	2.93
102	151.15	2051	3.82
103	152.20	1257	2.34
104	153.15	1167	2.18
105	154.15	781	1.46
106	155.15	1369	2.55
107	156.15	885	1.65
108	157.15	2493	4.65
109	158.15	1022	1.90
110	159.15	5303	9.88
111	160.20	1912	3.56
112	161.20	5991	11.17
113	162.20	1992	3.71
114	163.20	5185	9.66
115	164.15	1297	2.42
116	165.10	2182	4.07
117	166.15	838	1.56
118	167.20	856	1.60
119	168.20	441	0.82
120	169.15	968	1.80
121	170.20	458	0.85
122	171.15	1898	3.54
123	172.20	773	1.44
124	173.20	3522	6.56
125	174.20	1540	2.87
126	175.20	6150	11.46
127	176.15	2400	4.47
128	177.15	4800	8.95
129	178.20	1331	2.48
130	179.10	1971	3.67
131	180.10	592	1.10
132	181.10	670	1.25

DEPTT. OF BOTANICAL & ENVIRONMENTAL SCIENCES, G.N.D.U. AMRITSAR

#	m/z	Abs. Int.	Rel. Int.
133	182.20	364	0.68
134	183.10	898	1.67
135	184.10	340	0.63
136	185.15	1611	3.00
137	186.20	736	1.37
138	187.20	4995	9.31
139	188.20	1762	3.28
140	189.20	9204	17.15
141	190.15	3963	7.39
142	191.15	9287	17.31
143	192.05	2027	3.78
144	193.05	3565	6.64
145	194.00	866	1.61
146	195.05	700	1.30
147	196.10	284	0.53
148	197.10	715	1.33
149	198.10	314	0.59
150	199.15	1267	2.36
151	200.20	542	1.01
152	201.15	3290	6.13
153	202.25	2291	4.27
154	203.20	53655	100.00
155	204.20	9691	18.06
156	205.15	3426	6.39
157	206.15	1557	2.90
158	207.10	41814	77.93
159	208.10	8760	16.33
160	209.05	4747	8.85
161	210.00	837	1.56
162	211.10	768	1.43
163	212.10	250	0.47
164	213.15	1020	1.90
165	214.15	518	0.97
166	215.20	1830	3.41
167	216.20	2578	4.80
168	217.15	2220	4.14
169	218.15	1288	2.40
170	219.15	1662	3.10
171	220.15	1279	2.38
172	221.15	3038	5.66
173	222.15	970	1.81
174	223.20	860	1.60
175	224.20	236	0.44
176	225.10	505	0.94
177	226.20	208	0.39
178	227.20	842	1.57
179	228.10	313	0.58
180	229.15	1157	2.16
181	230.20	417	0.78
182	231.15	699	1.30
183	232.20	1255	2.34
184	233.15	829	1.55
185	234.20	4708	8.77
186	235.20	1456	2.71
187	236.20	346	0.64
188	237.20	415	0.77
189	238.20	105	0.20
190	239.20	486	0.91
191	240.20	139	0.26
192	241.15	711	1.33
193	242.20	278	0.52
194	243.20	569	1.06
195	244.20	163	0.30
196	245.10	423	0.79
197	246.20	214	0.40
198	247.25	584	1.09
199	248.00	414	0.77
200	249.00	2274	4.24
201	250.00	641	1.19
202	251.00	1227	2.29
203	252.00	363	0.68
204	253.10	662	1.23
205	254.10	201	0.37
206	255.25	865	1.61
207	256.30	307	0.57
208	257.20	711	1.33
209	258.20	235	0.44
210	259.20	371	0.69
211	260.20	145	0.27
212	261.20	127	0.24

#	m/z	Abs. Int.	Rel. Int.
213	262.10	45	0.08
214	263.10	158	0.29
215	264.00	24	0.04
216	265.05	2912	5.43
217	266.05	890	1.66
218	267.00	2950	5.50
219	268.05	855	1.59
220	269.10	889	1.66
221	270.10	335	0.62
222	271.15	498	0.93
223	272.20	254	0.47
224	273.20	845	1.57
225	274.20	265	0.49
226	275.20	320	0.60
227	276.20	51	0.10
228	277.20	75	0.14
229	278.20	19	0.04
230	279.20	128	0.24
231	280.10	48	0.09
232	281.05	12238	22.81
233	282.05	3443	6.42
234	283.05	2375	4.43
235	284.10	577	1.08
236	285.10	386	0.72
237	286.20	421	0.78
238	287.20	278	0.52
239	288.20	149	0.28
240	289.00	116	0.22
241	290.10	27	0.05
242	291.10	84	0.16
243	292.10	10	0.02
244	293.10	167	0.31
245	294.00	39	0.07
246	295.15	532	0.99
247	296.10	124	0.23
248	297.20	310	0.58
249	298.20	62	0.12
250	299.20	94	0.18
251	300.10	57	0.11
252	301.10	86	0.16
253	302.20	58	0.11
254	303.20	44	0.08
255	305.10	3	0.01
256	306.10	13	0.02
257	307.10	22	0.04
258	308.20	2	0.00
259	309.10	137	0.26
260	310.10	34	0.06
261	311.20	120	0.22
262	312.20	15	0.03
263	313.20	107	0.20
264	314.20	30	0.06
265	315.00	83	0.15
266	316.10	18	0.03
267	317.10	27	0.05
268	319.10	11	0.02
269	320.10	3	0.01
270	321.20	12	0.02
271	323.20	129	0.24
272	324.20	33	0.06
273	325.00	442	0.82
274	326.10	130	0.24
275	327.10	481	0.90
276	328.10	182	0.34
277	329.10	86	0.16
278	331.20	35	0.07
279	332.10	9	0.02
280	333.10	15	0.03
281	334.10	3	0.01
282	335.10	5	0.01
283	337.20	10	0.02
284	339.10	204	0.38
285	340.10	37	0.07
286	341.00	1161	2.16
287	342.10	423	0.79
288	343.10	282	0.53
289	344.10	25	0.05
290	347.10	6	0.01
291	350.10	3	0.01
292	351.00	17	0.03

#	m/z	Abs. Int.	Rel. Int.
293	352.00	10	0.02
294	353.00	38	0.07
295	354.00	12	0.02
296	355.10	1929	3.60
297	356.05	737	1.37
298	357.10	512	0.95
299	358.10	112	0.21
300	360.10	2	0.00
301	363.10	3	0.01
302	365.10	2	0.00
303	366.10	6	0.01
304	367.10	53	0.10
305	368.10	6	0.01
306	369.00	153	0.29
307	370.10	31	0.06
308	371.10	49	0.09
309	378.10	3	0.01
310	379.10	12	0.02
311	380.10	3	0.01
312	381.10	95	0.18
313	382.10	25	0.05
314	383.10	36	0.07
315	384.10	97	0.18
316	385.10	80	0.15
317	387.10	19	0.04
318	391.10	65	0.12
319	392.10	5	0.01
320	393.10	124	0.23
321	394.10	30	0.06
322	395.10	8	0.01
323	396.10	36	0.07
324	397.10	22	0.04
325	399.10	42	0.08
326	401.10	192	0.36
327	402.10	9	0.02
328	403.10	9	0.02
329	405.10	9	0.02
330	406.10	9	0.02
331	407.10	55	0.10
332	408.10	16	0.03
333	409.10	178	0.33
334	410.10	85	0.16
335	411.35	421	0.78
336	412.40	180	0.34
337	413.40	32	0.06
338	414.10	6	0.01
339	415.10	212	0.40
340	416.10	9	0.02
341	417.10	11	0.02
342	418.10	5	0.01
343	421.10	9	0.02
344	422.30	11	0.02
345	423.00	24	0.04
346	424.30	205	0.38
347	425.30	147	0.27
348	426.30	70	0.13
349	427.30	216	0.40
350	428.40	47	0.09
351	429.05	563	1.05
352	430.10	298	0.56
353	431.00	94	0.18
354	432.10	13	0.02
355	435.10	2	0.00
356	437.10	2	0.00
357	438.30	2	0.00
358	440.40	12	0.02
359	441.00	5	0.01
360	442.30	106	0.20
361	443.30	24	0.04
362	444.30	3	0.01
363	445.40	6	0.01
364	446.00	3	0.01
365	447.10	3	0.01
366	457.10	2	0.00
367	458.10	2	0.00
368	461.00	2	0.00
369	465.00	7	0.01
370	469.10	2	0.00
371	475.10	2	0.00
372	478.30	2	0.00

DEPTT. OF BOTANICAL & ENVIRONMENTAL SCIENCES, G.N.D.U. AMRITSAR

#	m/z	Abs. Int.	Rel. Int.
373	483.10	15	0.03
374	484.40	3	0.01
375	489.30	3	0.01
376	490.10	3	0.01
377	499.10	2	0.00
378	501.40	3	0.01
379	502.10	2	0.00
380	503.00	82	0.15
381	504.10	37	0.07
382	505.30	13	0.02
383	506.10	6	0.01
384	509.10	2	0.00
385	513.10	2	0.00
386	538.10	3	0.01
387	553.30	2	0.00
388	554.10	3	0.01
389	559.10	3	0.01
390	561.10	3	0.01
391	589.00	3	0.01
392	623.10	2	0.00
393	647.40	12	0.02
394	648.10	3	0.01
395	653.10	2	0.00
396	657.10	6	0.01
397	660.10	2	0.00
398	662.30	6	0.01
399	697.10	2	0.00

Peak Report TIC

Peak#	R.Time	Area	Area%	Height	Name
1	8.049	224315	0.08	109242	1-Dodecene (CAS) Adacene 12 $$ n-Dodec-1-ene $$ .alpha.-Dodecene $$ dodecene $$ n-undecane. 1-dodecene $$ Dodec-1-e
2	12.029	615449	0.22	417015	1-Tetradecene (CAS) n-Tetradec-1-ene $$ .alpha.-Tetradecene $$ 1-Butadecene $$ Dialene 14 $$
3	14.815	1014273	0.37	737001	Docosanoic acid (CAS) Behenic acid $$ Glycon B-70 $$ Hydrofol 2022-55 $$ Hydrofol Acid 560 $$ n-Docosanoic acid $$ 1-D
4	17.144	1298151	0.47	928856	1-Nonadecene (CAS)
5	17.640	2561456	0.93	1448882	NEOPHYTADIENE $$ 2,6,10-TRIMETHYL,14-ETHYLENE-14-PENTADECNE $$
6	18.070	660295	0.24	424683	3,7,11,15-Tetramethyl-2-hexadecen-1-ol $$ (2E)-3,7,11,15-Tetramethyl-2-hexadecen-1-ol # $$
7	18.845	3969267	1.44	1416885	Pentadecanoic acid $$ Pentadecylic acid $$ n-Pentadecanoic acid $$ n-Pentadecylic acid $$
8	19.211	1307357	0.47	925671	3-Eicosene, (E)- $$ (3E)-3-Icosene # $$
9	20.252	1519034	0.55	923686	2-Hexadecen-1-ol, 3,7,11,15-tetramethyl-, [R-[R*,R*-(E)]]- (CAS) Phytol $$ trans-Phytol $$ (E)-(7R,11R)-3,7,11,15-tetrameth
10	20.459	623076	0.23	457134	Linoleic acid P1365
11	20.708	1117833	0.41	574338	9-Octadecenoic acid (Z)- (CAS) Oleic acid $$ Red oil $$ Oelsauere $$ Oleine 7503 $$ Pamolyn 100 $$ Emersol 211 $$ Vopco
12	21.088	977101	0.35	751969	9-Eicosene, (E)- $$ (9E)-9-Icosene # $$
13	22.809	821064	0.30	562997	Heptadecyl trifluoroacetate
14	23.682	733605	0.27	573374	Hexatriacontane (CAS) n-Hexatriacontane $$ NOR-HEXATRIACONTANE $$
15	24.417	585621	0.21	386989	1-Decanol, 2-hexyl- $$ 2-Hexyl-1-decanol $$
16	25.404	7294763	2.64	3718172	Tetratetracontane (CAS) n-Tetratetracontane $$
17	26.372	387444	0.14	185108	Docosyl pentafluoropropionate
18	26.574	1996751	0.72	897059	FARNESOL ISOMER A $$
19	27.518	1012911	0.37	308751	Acetic acid, chloro-, octadecyl ester $$ Chloroacetic acid, octadecyl ester $$ Octadecyl chloroacetate # $$
20	27.765	3688977	1.34	1372980	Pentatriacontane (CAS) n-Pentatriacontane $$
21	31.128	6232646	2.26	1125708	Stearyl alcohol P1298
22	33.685	22888125	8.30	3017221	D:B-Friedo-18,19-secolup-19-ene, 3,10-epoxy-, (3.beta.,10.beta.)- $$ Baccharis oxide $$
23	35.174	6128986	2.22	1084538	Cholest-5-ene, 3-bromo-, (3.beta.)- $$ Cholest-5-ene, 3.beta.-bromo- $$ Cholesteryl bromide $$ 3.beta.-Bromocholest-5-ene $$
24	35.429	20914958	7.58	3766443	METHYL COMMATE C $$
25	35.593	1376491	0.50	390653	METHYL COMMATE B $$
26	36.302	98245935	35.62	9408446	METHYL COMMATE D $$
27	37.189	5583263	2.02	1532686	03027205002 FLAVONE 4'-OH,5-OH,7-DI-O-GLUCOSIDE $$
28	37.966	76711444	27.81	13759650	GLOBULOL $$ (-)-Globulol $$ 1H-Cycloprop[e]azulen-4-ol, decahydro-1,1,4,7-tetramethyl-, [1aR-(1a.alpha.,4.alpha.,4a.alpha
29	39.895	5334968	1.93	1277709	Olean-12-en-28-al (CAS)
		275825559	100.00	52483846	

Supplementary file S-4
GCMS Report Hexane Leaves

DEPTT. OF BOTANICAL & ENVIRONMENTAL SCIENCES, G.N.D.U. AMRITSAR

Sample Information

Analyzed by	: Admin
Analyzed	: 8/22/2015 1:16:32 PM
Sample Type	: Unknown
Sample Name	: ACIDS
Sample ID	: ANKET
Injection Volume	: 2
Data File	: E:\GCMS\GCMS DATA\Vandana\3.qgd
Method File	: E:\GCMS\GCMS DATA\anket\ANKET. B. JUNCEA PROFILING.qgm
Tuning File	: C:\GCMSsolution\System\Tune1\05-06-2014.qgt

Chromatogram ACIDS E:\GCMS\GCMS DATA\Vandana\3.qgd

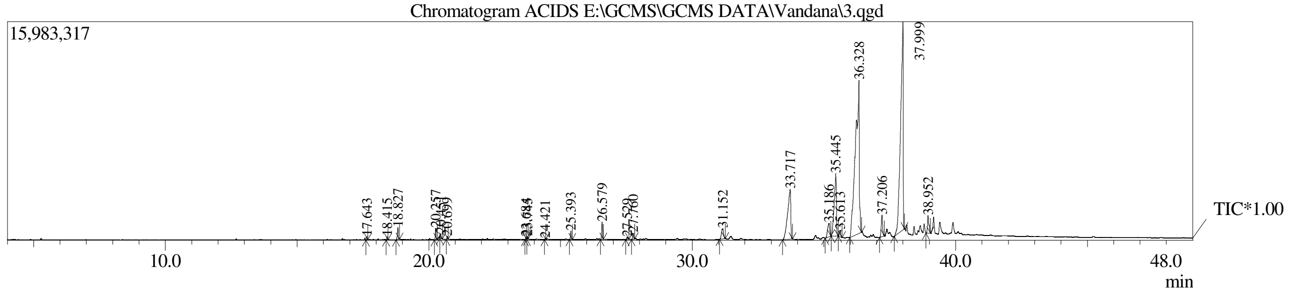

Spectrum

Line#:1 R.Time:17.6(Scan#:4094)
MassPeaks:123
RawMode:Averaged 17.6-17.7(4080-4106) BasePeak:57(10189)
BG Mode:None Group 1 - Event 1

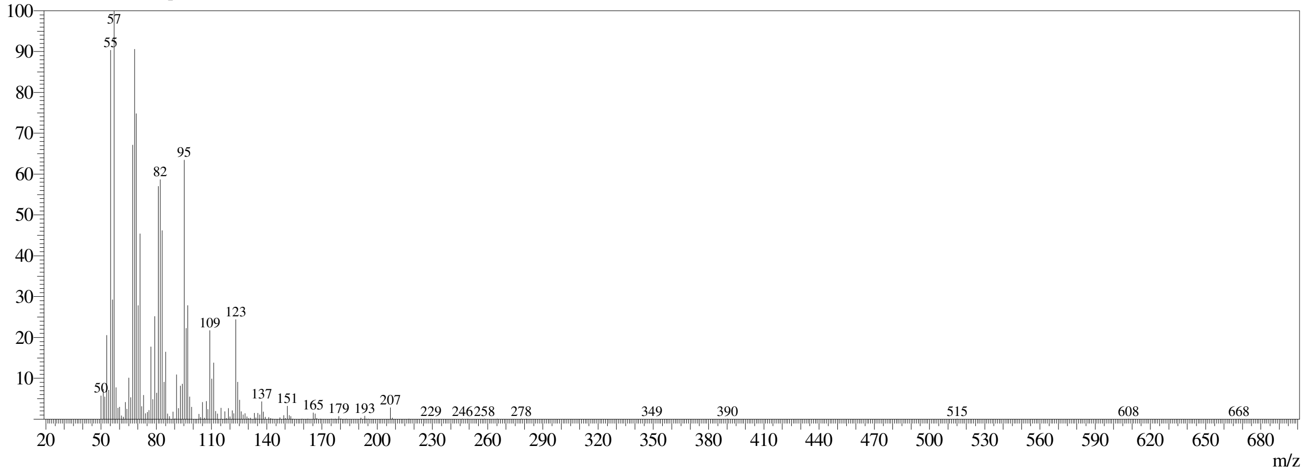

DEPTT. OF BOTANICAL & ENVIRONMENTAL SCIENCES,
G.N.D.U.
AMRITSAR

Line#:2 R.Time:18.4(Scan#:4326)
MassPeaks:150
RawMode:Averaged 18.4-18.5(4316-4341) BasePeak:74(16218)
BG Mode:None Group 1 - Event 1

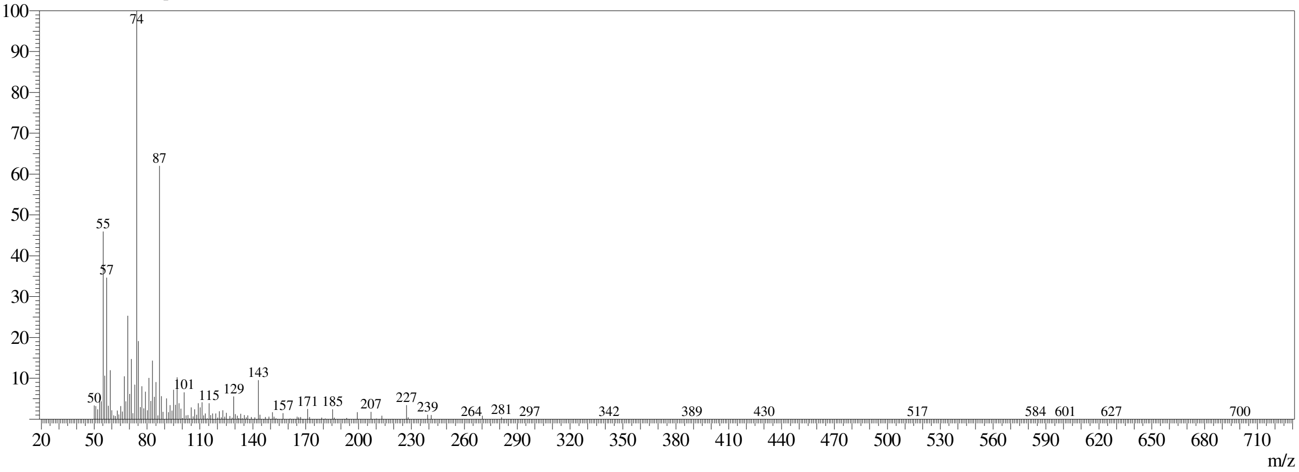

Line#:3 R.Time:18.8(Scan#:4449)
MassPeaks:184
RawMode:Averaged 18.8-18.9(4429-4468) BasePeak:73(29011)
BG Mode:None Group 1 - Event 1

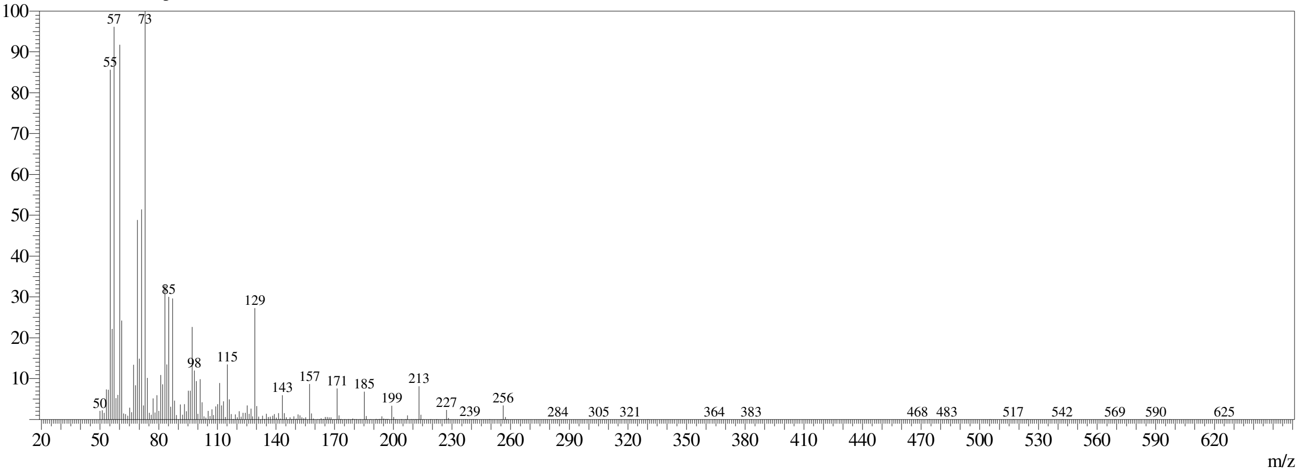

Line#:4 R.Time:20.3(Scan#:4878)
MassPeaks:168
RawMode:Averaged 20.2-20.3(4865-4894) BasePeak:71(44550)
BG Mode:None Group 1 - Event 1

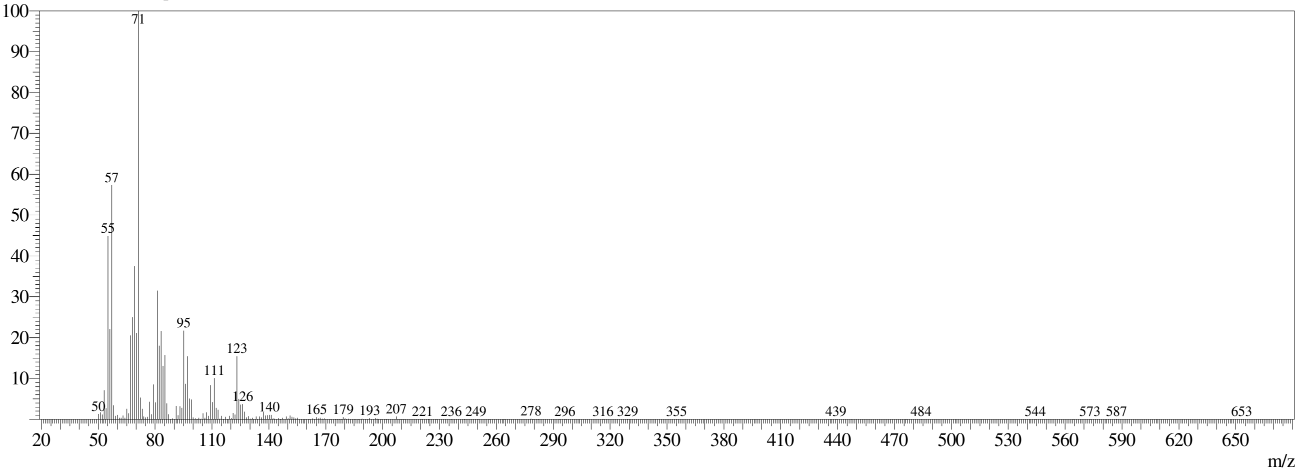

DEPTT. OF BOTANICAL & ENVIRONMENTAL SCIENCES, G.N.D.U. AMRITSAR

Line#:5 R.Time:20.4(Scan#:4936)
MassPeaks:183
RawMode:Averaged 20.4-20.5(4927-4957) BasePeak:55(20093)
BG Mode:None Group 1 - Event 1

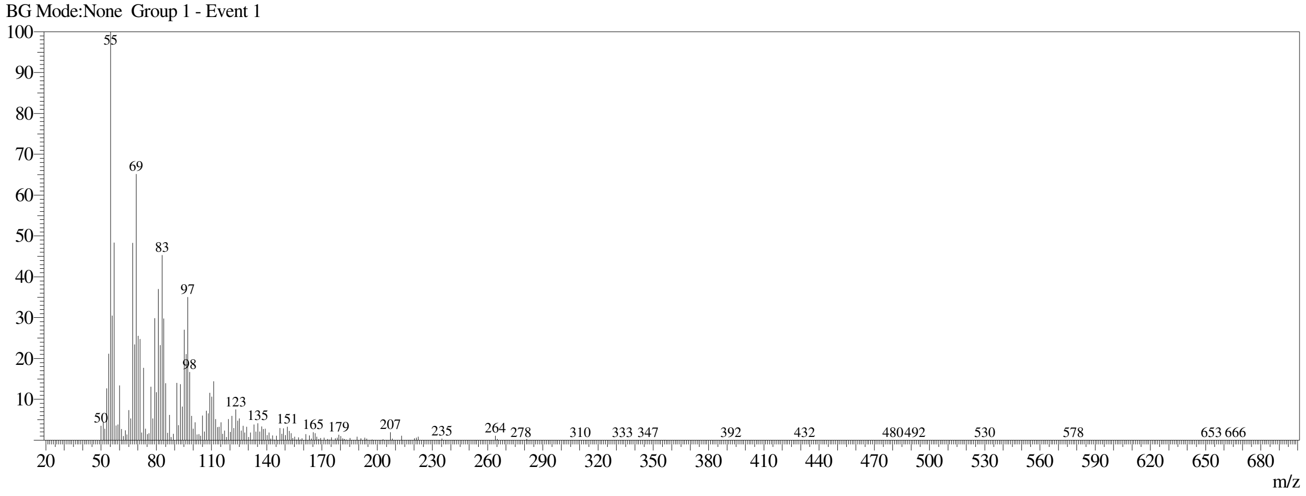

Line#:6 R.Time:20.7(Scan#:5011)
MassPeaks:181
RawMode:Averaged 20.7-20.7(4998-5025) BasePeak:57(11949)
BG Mode:None Group 1 - Event 1

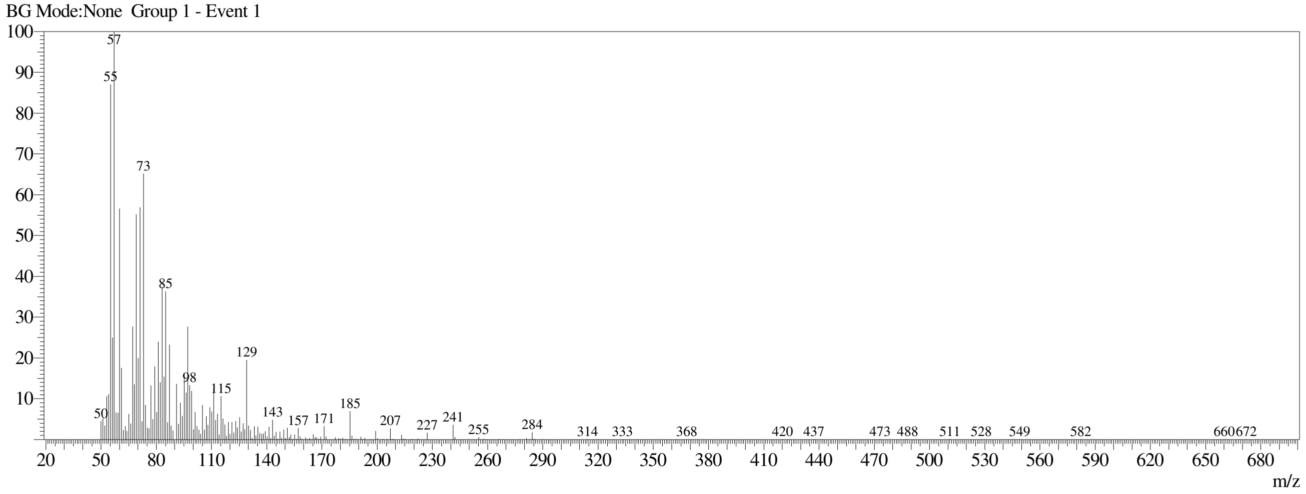

Line#:7 R.Time:23.7(Scan#:5906)
MassPeaks:167
RawMode:Averaged 23.7-23.7(5896-5916) BasePeak:57(21258)
BG Mode:None Group 1 - Event 1

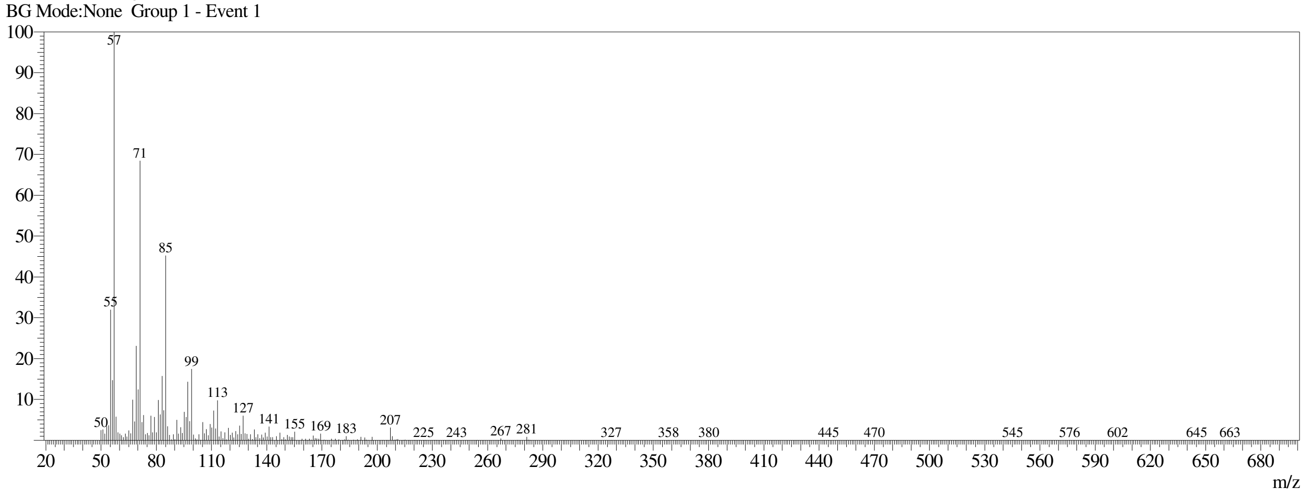

DEPTT. OF BOTANICAL & ENVIRONMENTAL SCIENCES, G.N.D.U. AMRITSAR

Line#:8   R.Time:23.7(Scan#:5924)
MassPeaks:171
RawMode:Averaged 23.7-23.8(5916-5934) BasePeak:149(18032)
BG Mode:None Group 1 - Event 1

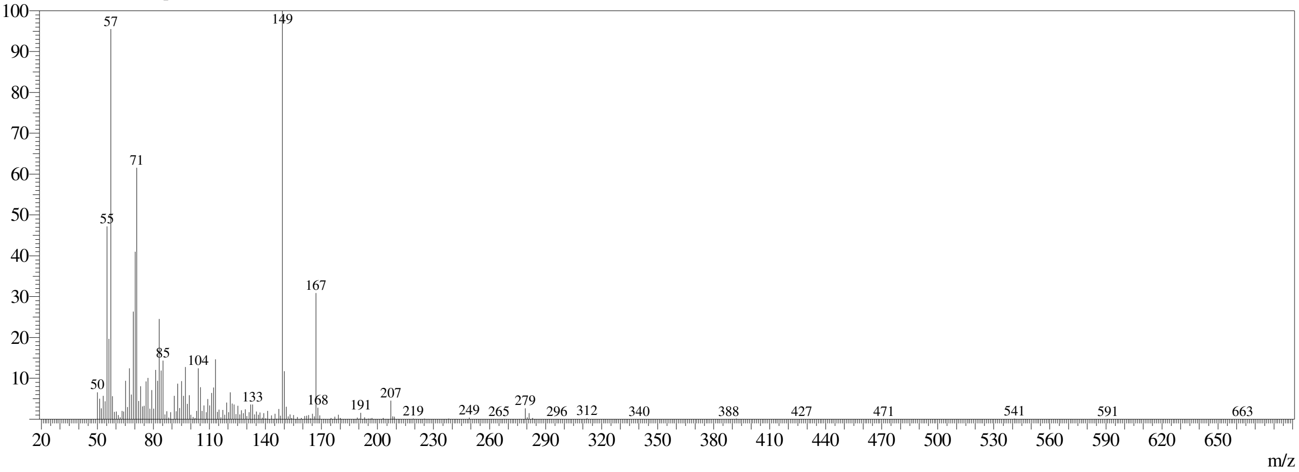

Line#:9   R.Time:24.4(Scan#:6127)
MassPeaks:167
RawMode:Averaged 24.4-24.4(6117-6134) BasePeak:57(8212)
BG Mode:None Group 1 - Event 1

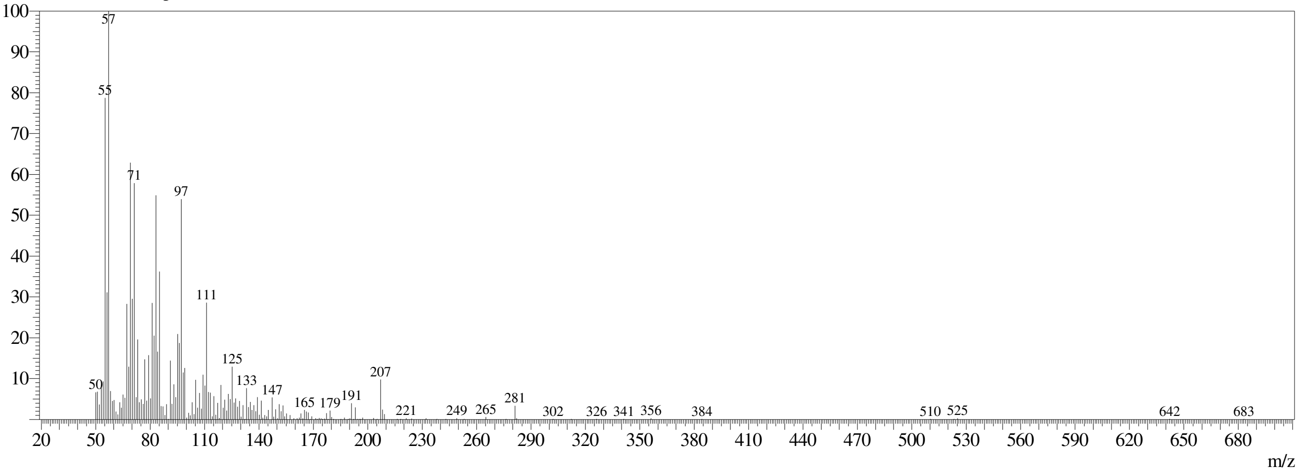

Line#:10   R.Time:25.4(Scan#:6419)
MassPeaks:207
RawMode:Averaged 25.3-25.4(6405-6433) BasePeak:57(54064)
BG Mode:None Group 1 - Event 1

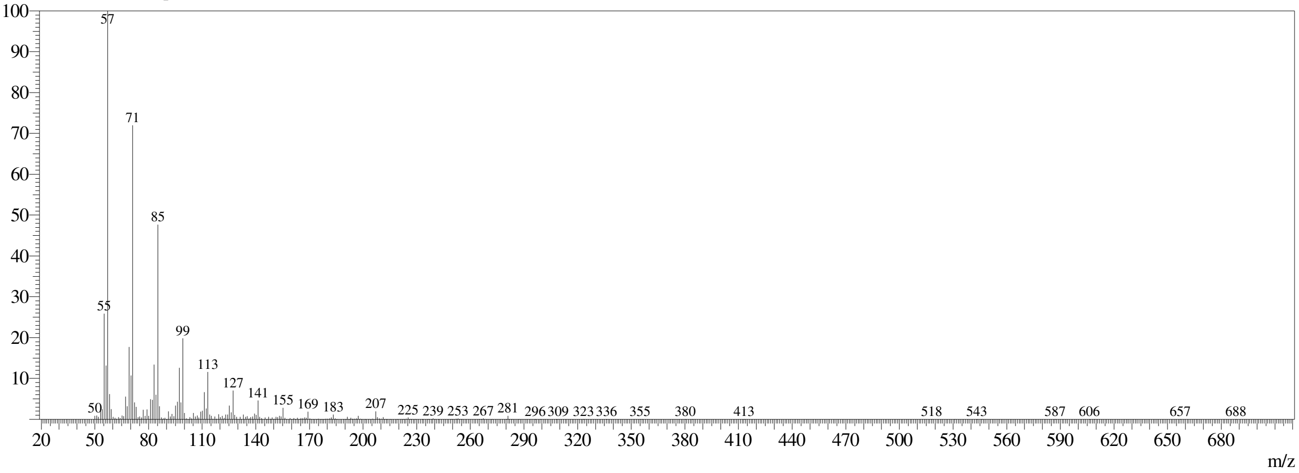

DEPTT. OF BOTANICAL & ENVIRONMENTAL SCIENCES, G.N.D.U. AMRITSAR

Line#:11 R.Time:26.6(Scan#:6775)
MassPeaks:220
RawMode:Averaged 26.5-26.6(6754-6794) BasePeak:69(104589)
BG Mode:None Group 1 - Event 1

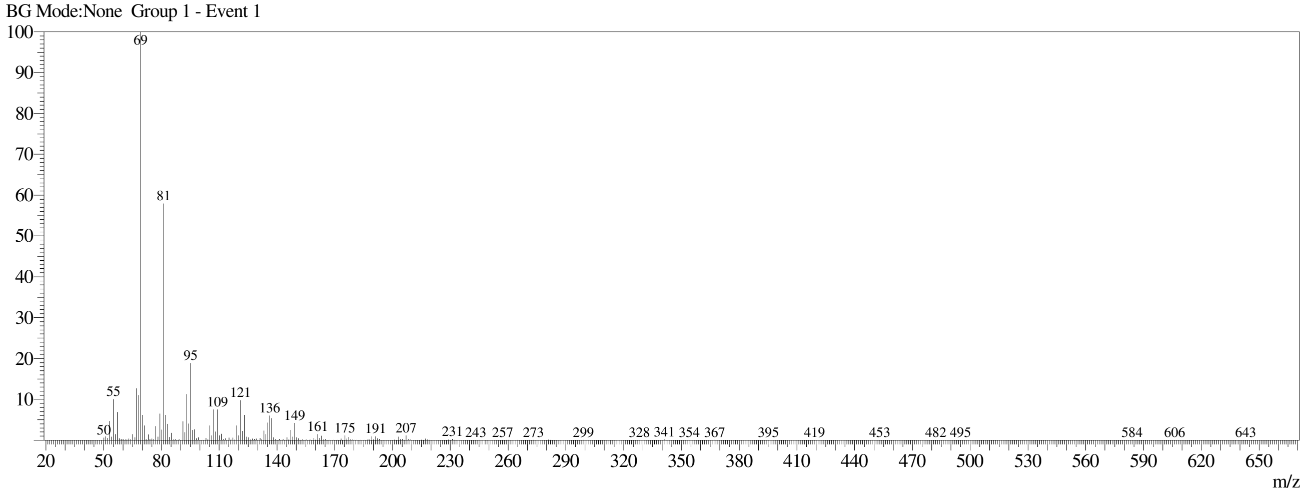

Line#:12 R.Time:27.5(Scan#:7060)
MassPeaks:204
RawMode:Averaged 27.5-27.6(7041-7088) BasePeak:57(11111)
BG Mode:None Group 1 - Event 1

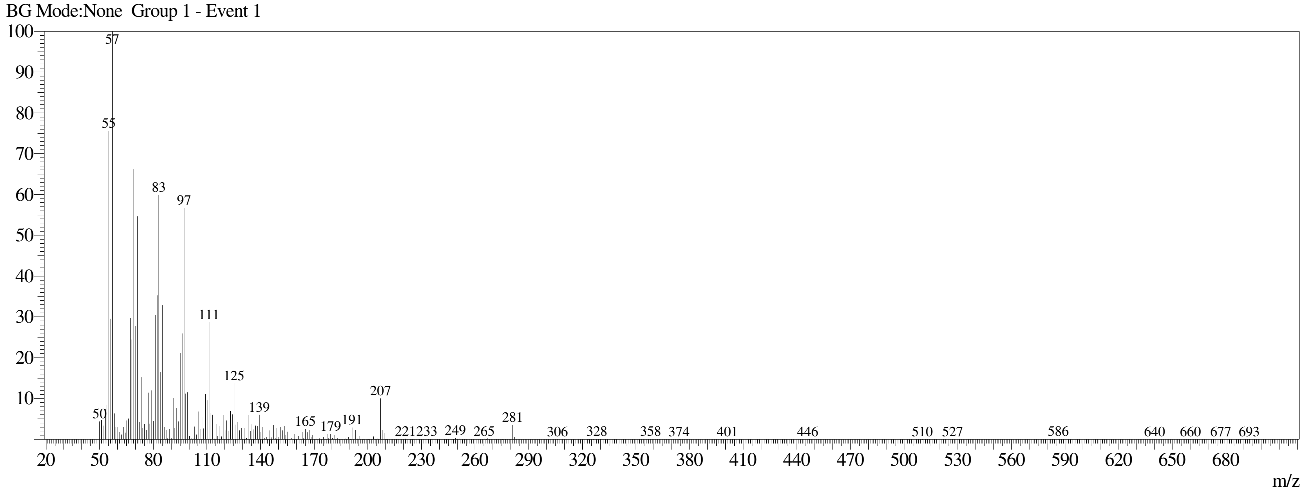

Line#:13 R.Time:27.8(Scan#:7129)
MassPeaks:210
RawMode:Averaged 27.7-27.8(7109-7144) BasePeak:57(43140)
BG Mode:None Group 1 - Event 1

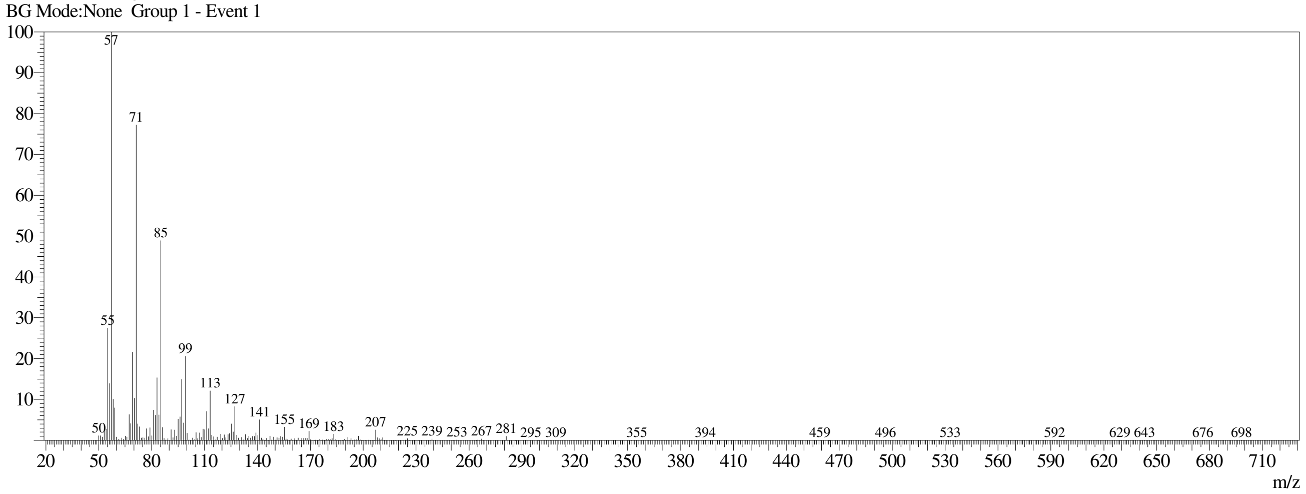

DEPTT. OF BOTANICAL & ENVIRONMENTAL SCIENCES, G.N.D.U. AMRITSAR

Line#:14 R.Time:31.2(Scan#:8147)
MassPeaks:278
RawMode:Averaged 31.0-31.3(8110-8179) BasePeak:165(40621)
BG Mode:None Group 1 - Event 1

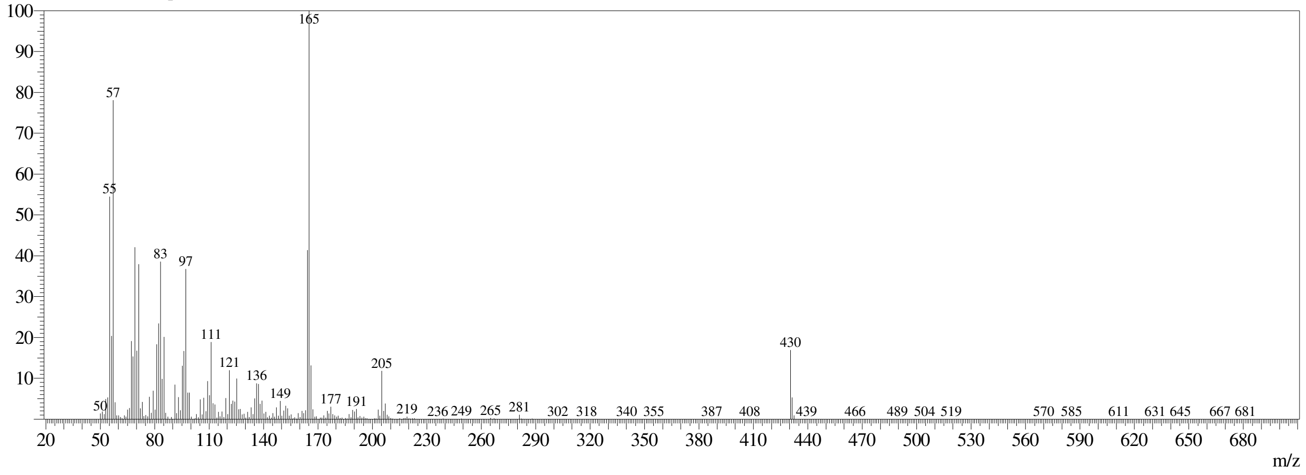

Line#:15 R.Time:33.7(Scan#:8916)
MassPeaks:367
RawMode:Averaged 33.4-33.8(8826-8939) BasePeak:137(120935)
BG Mode:None Group 1 - Event 1

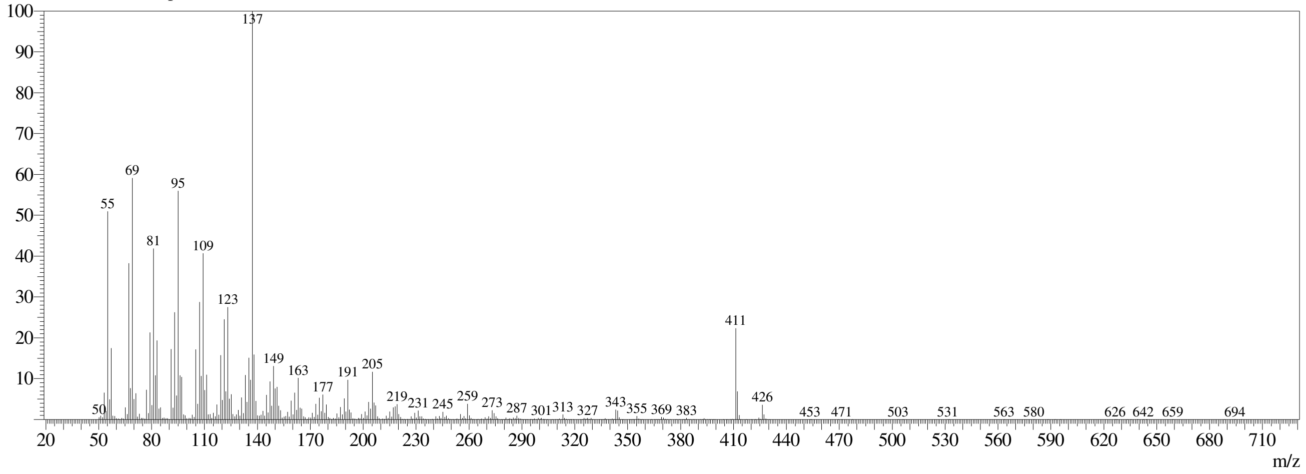

Line#:16 R.Time:35.2(Scan#:9357)
MassPeaks:327
RawMode:Averaged 35.0-35.3(9315-9384) BasePeak:55(23493)
BG Mode:None Group 1 - Event 1

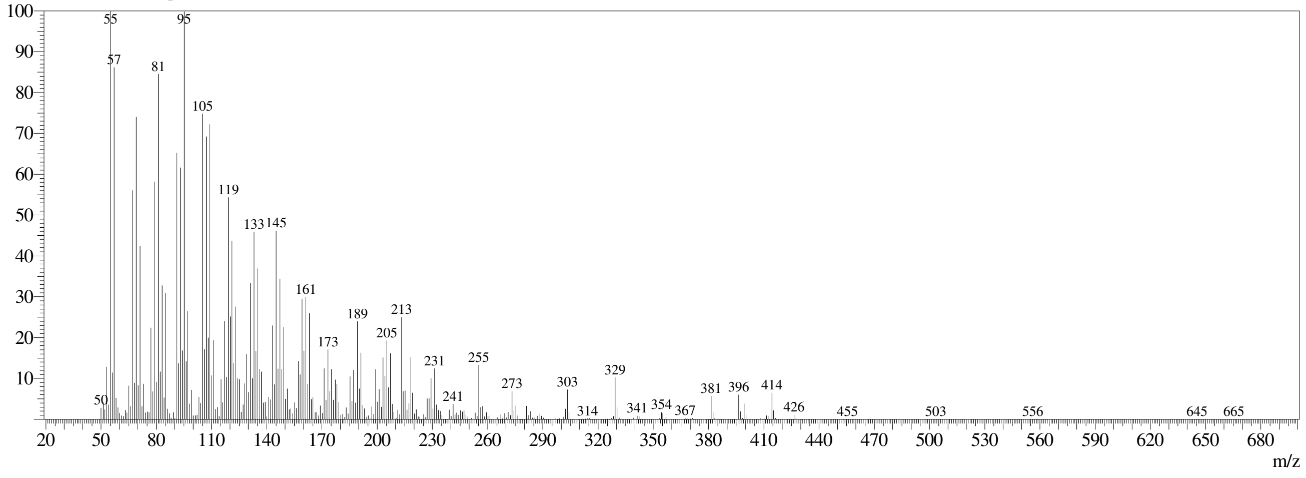

DEPTT. OF BOTANICAL & ENVIRONMENTAL SCIENCES, G.N.D.U. AMRITSAR

Line#:17   R.Time:35.4(Scan#:9434)
MassPeaks:355
RawMode:Averaged 35.3-35.5(9380-9459) BasePeak:218(172388)
BG Mode:None Group 1 - Event 1

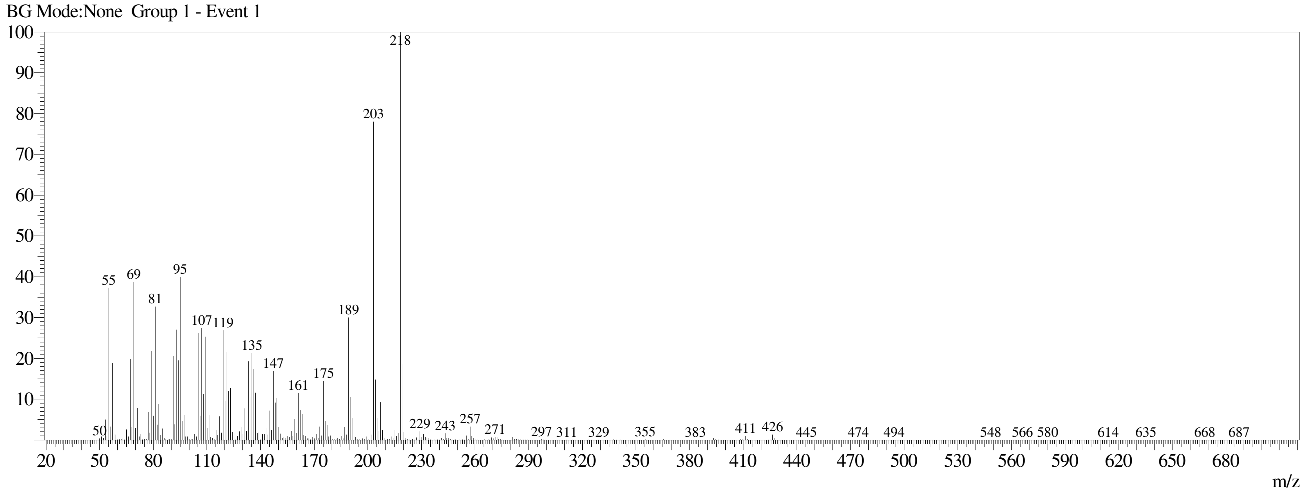

Line#:18   R.Time:35.6(Scan#:9485)
MassPeaks:283
RawMode:Averaged 35.5-35.7(9459-9505) BasePeak:55(16679)
BG Mode:None Group 1 - Event 1

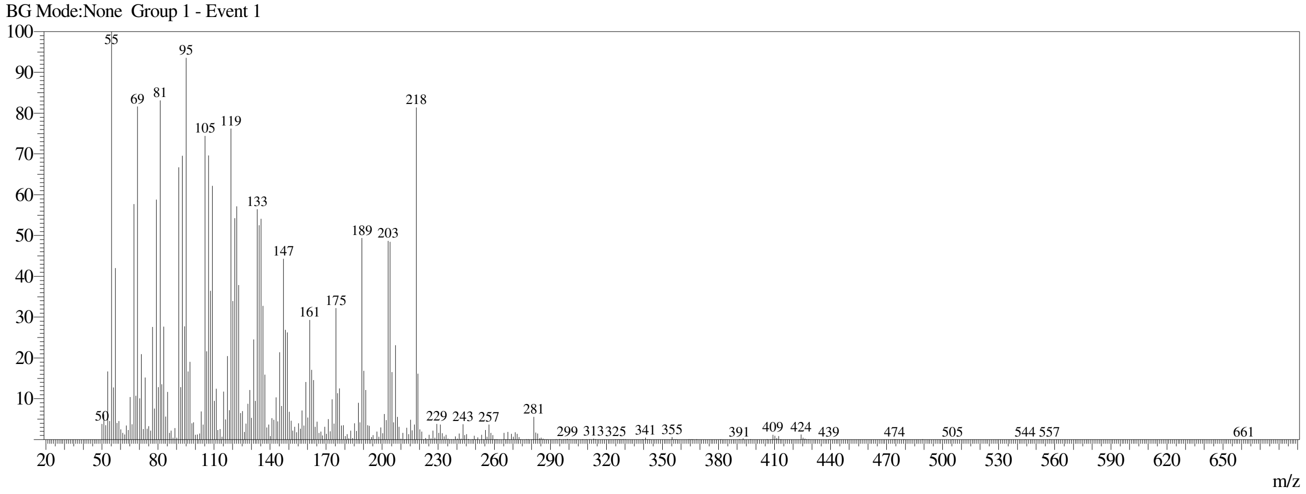

Line#:19   R.Time:36.3(Scan#:9699)
MassPeaks:418
RawMode:Averaged 36.0-36.4(9590-9730) BasePeak:218(224924)
BG Mode:None Group 1 - Event 1

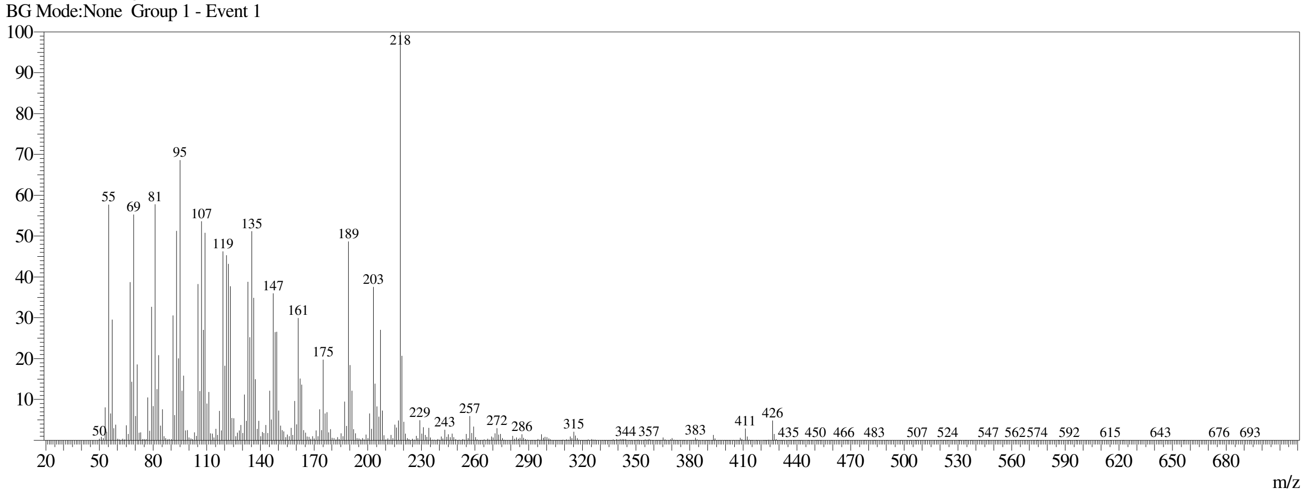

DEPTT. OF BOTANICAL & ENVIRONMENTAL SCIENCES, G.N.D.U. AMRITSAR

Line#:20 R.Time:37.2(Scan#:9963)
MassPeaks:345
RawMode:Averaged 37.1-37.3(9936-9993) BasePeak:69(34282)
BG Mode:None Group 1 - Event 1

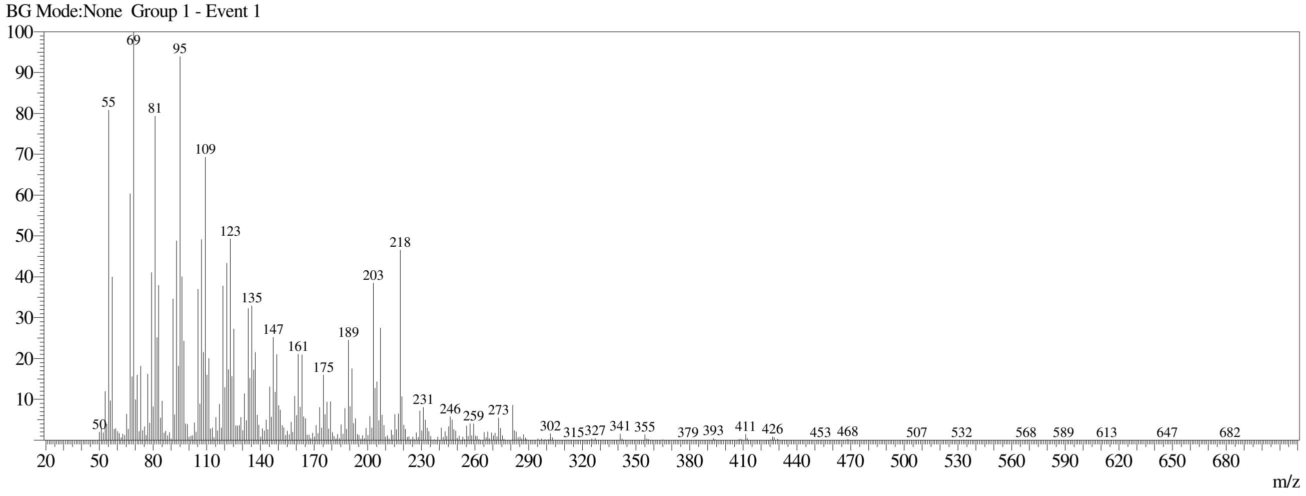

Line#:21 R.Time:38.0(Scan#:10201)
MassPeaks:439
RawMode:Averaged 37.7-38.1(10100-10217) BasePeak:69(243275)
BG Mode:None Group 1 - Event 1

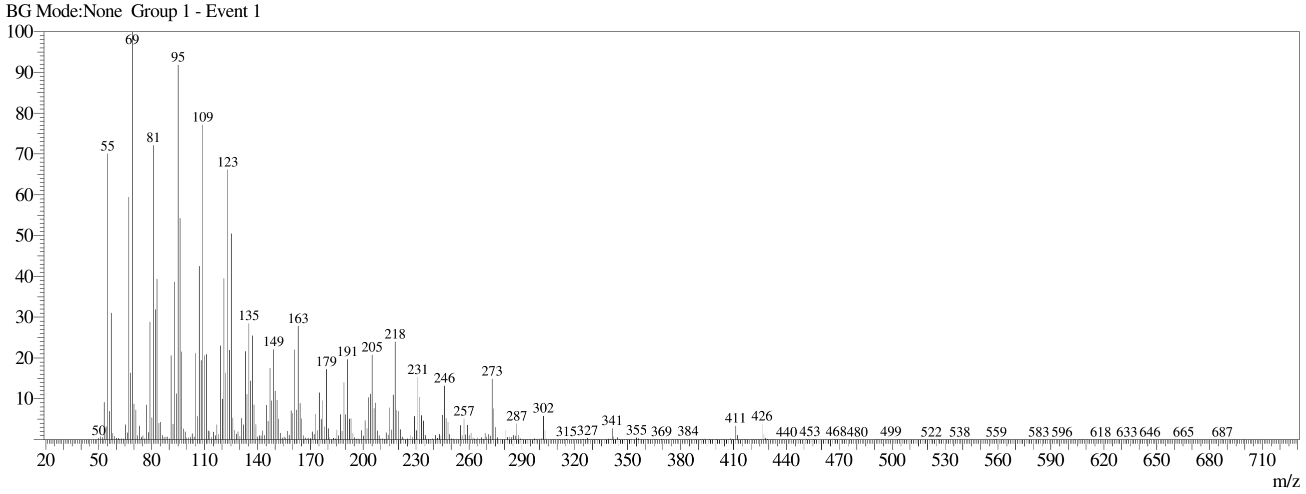

Line#:22 R.Time:39.0(Scan#:10487)
MassPeaks:386
RawMode:Averaged 38.9-39.1(10467-10519) BasePeak:203(47878)
BG Mode:None Group 1 - Event 1

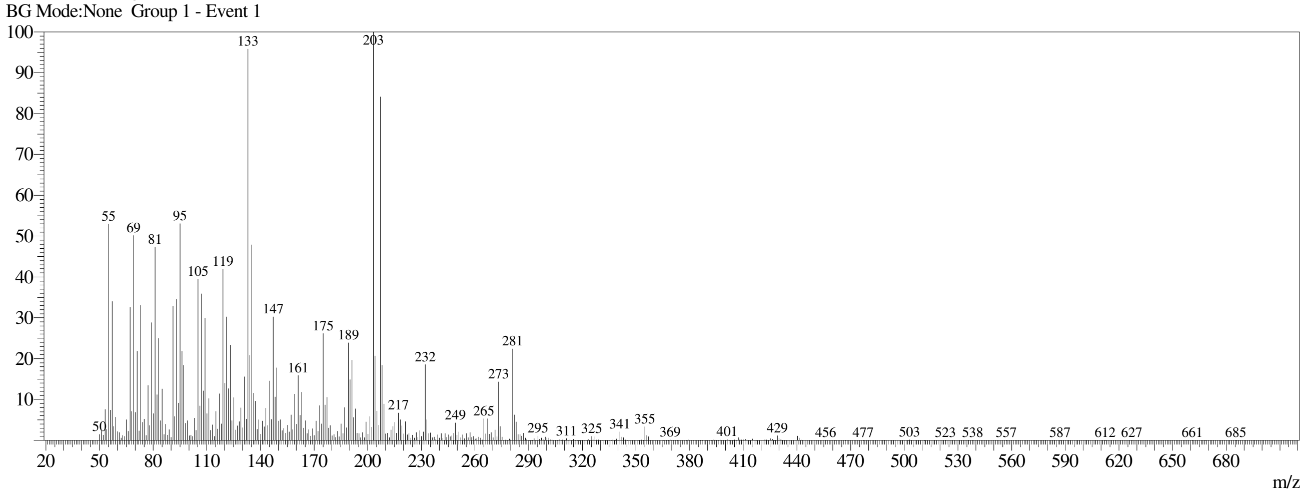

DEPTT. OF BOTANICAL & ENVIRONMENTAL SCIENCES, G.N.D.U. AMRITSAR

#	m/z	Abs. Int.	Rel. Int.
85	134.10	42	0.26
86	135.20	166	1.02
87	136.20	77	0.47
88	137.20	147	0.91
89	139.20	88	0.54
90	141.10	81	0.50
91	143.15	1548	9.54
92	144.10	183	1.13
93	145.10	18	0.11
94	147.10	90	0.55
95	149.10	107	0.66
96	150.20	8	0.05
97	151.10	279	1.72
98	152.10	102	0.63
99	153.20	44	0.27
100	154.10	7	0.04
101	157.20	241	1.49
102	158.10	18	0.11
103	161.10	24	0.15
104	163.20	8	0.05
105	165.20	107	0.66
106	166.10	79	0.49
107	167.20	90	0.55
108	171.20	403	2.48
109	172.20	86	0.53
110	179.20	65	0.40
111	181.20	25	0.15
112	185.20	395	2.44
113	186.20	68	0.42
114	191.20	19	0.12
115	192.20	7	0.04
116	193.20	49	0.30
117	194.20	7	0.04
118	199.20	280	1.73
119	200.20	16	0.10
120	207.10	289	1.78
121	209.10	7	0.04
122	210.20	25	0.15
123	212.20	16	0.10
124	213.20	136	0.84
125	214.20	8	0.05
126	219.20	8	0.05
127	227.20	555	3.42
128	228.20	71	0.44
129	229.10	8	0.05
130	239.20	178	1.10
131	240.20	17	0.10
132	241.20	159	0.98
133	264.10	8	0.05
134	267.20	16	0.10
135	270.20	146	0.90
136	271.20	8	0.05
137	281.10	76	0.47
138	285.20	9	0.06
139	297.20	8	0.05
140	342.20	7	0.04
141	388.10	8	0.05
142	389.10	17	0.10
143	430.10	8	0.05
144	517.20	7	0.04
145	584.20	7	0.04
146	601.10	8	0.05
147	609.10	8	0.05
148	622.20	7	0.04
149	627.10	8	0.05
150	700.20	7	0.04

Line#:3 R.Time:18.8(Scan#:4449)
MassPeaks:184
RawMode:Averaged 18.8-18.9(4429-4468) BasePeak:73(29011)
BG Mode:None Group 1 - Event 1

#	m/z	Abs. Int.	Rel. Int.
1	50.00	610	2.10
2	51.10	668	2.30
3	52.00	478	1.65
4	53.15	2148	7.40
5	54.15	2113	7.28
6	55.15	24841	85.63
7	56.15	6449	22.23
8	57.15	27894	96.15
9	58.15	1531	5.28
10	59.15	1744	6.01
11	60.10	26632	91.80
12	61.10	7035	24.25
13	62.10	423	1.46
14	63.10	393	1.35
15	64.10	269	0.93
16	65.10	833	2.87
17	66.10	515	1.78
18	67.10	3890	13.41
19	68.10	2435	8.39
20	69.10	14165	48.83
21	70.15	4320	14.89
22	71.15	14926	51.45
23	72.15	992	3.42
24	73.10	29011	100.00
25	74.10	2962	10.21
26	75.10	486	1.68
27	76.10	343	1.18
28	77.05	1506	5.19
29	78.10	509	1.75
30	79.10	1740	6.00
31	80.10	624	2.15
32	81.10	3161	10.90
33	82.10	2500	8.62
34	83.10	9602	33.10
35	84.10	3913	13.49
36	85.10	8715	30.04
37	86.10	918	3.16
38	87.10	8612	29.69
39	88.10	1352	4.66
40	89.10	323	1.11
41	90.10	16	0.06
42	91.10	1079	3.72
43	92.10	339	1.17
44	93.10	1085	3.74
45	94.10	583	2.01
46	95.10	2042	7.04
47	96.15	2043	7.04
48	97.15	6565	22.63
49	98.15	3490	12.03
50	99.15	2728	9.40
51	100.10	409	1.41
52	101.10	2870	9.89
53	102.10	1238	4.27
54	103.10	233	0.80
55	104.10	131	0.45
56	105.20	612	2.11
57	106.20	247	0.85
58	107.15	724	2.50
59	108.10	322	1.11
60	109.15	932	3.21
61	110.15	1097	3.78
62	111.15	2590	8.93
63	112.15	1003	3.46
64	113.15	1286	4.43
65	114.10	186	0.64
66	115.15	3914	13.49
67	116.15	1425	4.91
68	117.10	385	1.33
69	118.10	52	0.18
70	119.10	364	1.25
71	120.10	161	0.55
72	121.15	602	2.08
73	122.10	213	0.73
74	123.15	474	1.63
75	124.20	480	1.65
76	125.20	990	3.41
77	126.20	402	1.39
78	127.20	772	2.66
79	128.10	234	0.81
80	129.15	7917	27.29
81	130.15	957	3.30
82	131.10	210	0.72
83	132.10	11	0.04
84	133.10	281	0.97
85	134.10	37	0.13
86	135.10	410	1.41
87	136.10	193	0.67
88	137.10	205	0.71
89	138.25	282	0.97
90	139.15	389	1.34
91	140.20	143	0.49
92	141.20	466	1.61
93	142.20	74	0.26
94	143.20	1734	5.98
95	144.20	457	1.58
96	145.20	150	0.52
97	146.20	12	0.04
98	147.20	161	0.55
99	148.10	38	0.13
100	149.20	221	0.76
101	150.20	57	0.20
102	151.20	354	1.22
103	152.20	290	1.00
104	153.20	165	0.57
105	154.20	87	0.30
106	155.20	164	0.57
107	157.20	2534	8.73
108	158.15	436	1.50
109	159.20	70	0.24
110	161.20	32	0.11
111	162.10	5	0.02
112	163.10	85	0.29
113	164.20	35	0.12
114	165.20	193	0.67
115	166.20	180	0.62
116	167.10	168	0.58
117	168.20	148	0.51
118	169.20	5	0.02
119	170.20	5	0.02
120	171.20	2211	7.62
121	172.20	303	1.04
122	173.20	44	0.15
123	175.20	28	0.10
124	177.20	16	0.06
125	179.20	59	0.20
126	180.10	28	0.10
127	181.10	25	0.09
128	183.10	15	0.05
129	185.20	1986	6.85
130	186.20	260	0.90
131	188.20	5	0.02
132	191.20	42	0.14
133	192.20	5	0.02
134	193.20	49	0.17
135	194.20	223	0.77
136	195.20	59	0.20
137	196.20	48	0.17
138	197.20	49	0.17
139	199.20	984	3.39
140	200.20	171	0.59
141	201.20	10	0.03
142	204.20	5	0.02
143	206.20	5	0.02
144	207.20	292	1.01
145	209.20	23	0.08
146	210.10	5	0.02
147	211.20	7	0.02
148	212.20	34	0.12
149	213.20	2359	8.13
150	214.20	343	1.18
151	215.20	23	0.08
152	219.20	17	0.06
153	222.20	5	0.02
154	226.20	10	0.03
155	227.15	693	2.39
156	228.20	105	0.36

DEPTT. OF BOTANICAL & ENVIRONMENTAL SCIENCES, G.N.D.U. AMRITSAR

#	m/z	Abs. Int.	Rel. Int.	#	m/z	Abs. Int.	Rel. Int.	#	m/z	Abs. Int.	Rel. Int.
157	237.10	5	0.02	167	284.20	5	0.02	177	505.10	5	0.02
158	239.20	15	0.05	168	305.10	5	0.02	178	517.10	6	0.02
159	240.20	5	0.02	169	307.20	5	0.02	179	524.10	5	0.02
160	241.10	12	0.04	170	321.20	5	0.02	180	542.20	5	0.02
161	242.20	11	0.04	171	330.20	5	0.02	181	569.10	5	0.02
162	249.10	5	0.02	172	364.20	11	0.04	182	590.10	5	0.02
163	255.10	10	0.03	173	383.10	5	0.02	183	597.10	5	0.02
164	256.25	996	3.43	174	468.20	5	0.02	184	625.20	5	0.02
165	257.30	174	0.60	175	474.10	5	0.02				
166	258.20	5	0.02	176	483.20	5	0.02				

Line#:4 R.Time:20.3(Scan#:4878)
MassPeaks:168
RawMode:Averaged 20.2-20.3(4865-4894) BasePeak:71(44550)
BG Mode:None Group 1 - Event 1

#	m/z	Abs. Int.	Rel. Int.	#	m/z	Abs. Int.	Rel. Int.	#	m/z	Abs. Int.	Rel. Int.
1	50.00	590	1.32	57	106.20	160	0.36	113	168.20	68	0.15
2	51.05	761	1.71	58	107.15	776	1.74	114	169.20	92	0.21
3	52.15	514	1.15	59	108.15	400	0.90	115	172.20	13	0.03
4	53.15	3170	7.12	60	109.15	3735	8.38	116	175.20	13	0.03
5	54.15	1217	2.73	61	110.15	1888	4.24	117	176.20	7	0.02
6	55.15	19990	44.87	62	111.15	4516	10.14	118	177.20	17	0.04
7	56.15	9869	22.15	63	112.20	1263	2.84	119	178.20	6	0.01
8	57.15	25529	57.30	64	113.15	1039	2.33	120	179.10	252	0.57
9	58.15	1544	3.47	65	114.20	73	0.16	121	180.20	108	0.24
10	59.10	395	0.89	66	115.10	397	0.89	122	181.10	52	0.12
11	60.10	486	1.09	67	116.10	40	0.09	123	182.10	83	0.19
12	61.20	190	0.43	68	117.20	276	0.62	124	183.20	46	0.10
13	62.10	177	0.40	69	118.10	42	0.09	125	185.20	8	0.02
14	63.10	404	0.91	70	119.20	368	0.83	126	187.20	14	0.03
15	64.10	186	0.42	71	120.20	140	0.31	127	189.10	53	0.12
16	65.10	1142	2.56	72	121.10	697	1.56	128	191.10	46	0.10
17	66.10	667	1.50	73	122.15	528	1.19	129	193.20	138	0.31
18	67.10	9152	20.54	74	123.15	6869	15.42	130	196.20	157	0.35
19	68.10	11134	24.99	75	124.15	2238	5.02	131	197.10	68	0.15
20	69.10	16702	37.49	76	125.20	1617	3.63	132	198.10	21	0.05
21	70.15	9426	21.16	77	126.20	1675	3.76	133	199.20	15	0.03
22	71.10	44550	100.00	78	127.20	841	1.89	134	201.10	13	0.03
23	72.15	2388	5.36	79	128.20	205	0.46	135	202.10	6	0.01
24	73.10	1157	2.60	80	129.20	348	0.78	136	207.20	309	0.69
25	74.10	319	0.72	81	130.20	57	0.13	137	208.20	32	0.07
26	75.10	227	0.51	82	131.20	178	0.40	138	209.20	7	0.02
27	76.10	294	0.66	83	132.20	18	0.04	139	210.20	14	0.03
28	77.10	1906	4.28	84	133.20	314	0.70	140	211.20	14	0.03
29	78.15	547	1.23	85	134.20	31	0.07	141	220.20	16	0.04
30	79.10	3822	8.58	86	135.20	328	0.74	142	221.10	41	0.09
31	80.15	1855	4.16	87	136.20	206	0.46	143	224.20	6	0.01
32	81.15	14038	31.51	88	137.20	802	1.80	144	228.10	7	0.02
33	82.10	8053	18.08	89	138.15	414	0.93	145	236.20	13	0.03
34	83.15	9659	21.68	90	139.15	453	1.02	146	243.10	6	0.01
35	84.10	5833	13.09	91	140.20	495	1.11	147	249.20	33	0.07
36	85.15	7039	15.80	92	141.20	492	1.10	148	250.20	7	0.02
37	86.15	1745	3.92	93	142.20	110	0.25	149	256.10	6	0.01
38	87.10	571	1.28	94	143.10	55	0.12	150	264.20	6	0.01
39	88.10	21	0.05	95	145.10	149	0.33	151	278.20	63	0.14
40	89.10	110	0.25	96	146.10	6	0.01	152	279.20	7	0.02
41	90.10	22	0.05	97	147.10	158	0.35	153	281.20	46	0.10
42	91.10	1478	3.32	98	149.20	315	0.71	154	282.10	14	0.03
43	92.10	456	1.02	99	150.20	109	0.24	155	293.20	6	0.01
44	93.10	1446	3.25	100	151.20	433	0.97	156	296.10	13	0.03
45	94.15	1256	2.82	101	152.20	274	0.62	157	316.20	6	0.01
46	95.15	9678	21.72	102	153.20	193	0.43	158	329.20	7	0.02
47	96.15	3862	8.67	103	154.20	103	0.23	159	355.10	7	0.02
48	97.15	6880	15.44	104	155.20	166	0.37	160	439.10	7	0.02
49	98.15	2254	5.06	105	157.20	23	0.05	161	447.10	7	0.02
50	99.15	2156	4.84	106	159.20	46	0.10	162	476.20	6	0.01
51	100.10	227	0.51	107	161.20	15	0.03	163	484.10	7	0.02
52	101.20	103	0.23	108	163.20	108	0.24	164	544.20	7	0.02
53	102.10	14	0.03	109	164.20	31	0.07	165	564.20	6	0.01
54	103.10	174	0.39	110	165.20	238	0.53	166	573.10	7	0.02
55	104.20	57	0.13	111	166.10	118	0.26	167	587.20	6	0.01
56	105.20	668	1.50	112	167.10	160	0.36	168	653.20	13	0.03

Line#:5 R.Time:20.4(Scan#:4936)
MassPeaks:183
RawMode:Averaged 20.4-20.5(4927-4957) BasePeak:55(20093)
BG Mode:None Group 1 - Event 1

#	m/z	Abs. Int.	Rel. Int.	#	m/z	Abs. Int.	Rel. Int.	#	m/z	Abs. Int.	Rel. Int.
1	50.00	712	3.54	3	52.10	565	2.81	5	54.15	4260	21.20
2	51.10	904	4.50	4	53.15	2551	12.70	6	55.15	20093	100.00

DEPTT. OF BOTANICAL & ENVIRONMENTAL SCIENCES, G.N.D.U. AMRITSAR

#	m/z	Abs. Int.	Rel. Int.
7	56.10	6125	30.48
8	57.15	9730	48.42
9	58.10	721	3.59
10	59.10	777	3.87
11	60.10	2688	13.38
12	61.10	544	2.71
13	62.10	208	1.04
14	63.10	494	2.46
15	64.10	278	1.38
16	65.10	1476	7.35
17	66.10	1064	5.30
18	67.10	9705	48.30
19	68.15	4714	23.46
20	69.15	13095	65.17
21	70.15	5141	25.59
22	71.15	4981	24.79
23	72.10	379	1.89
24	73.10	3562	17.73
25	74.10	568	2.83
26	75.10	306	1.52
27	76.10	352	1.75
28	77.10	2636	13.12
29	78.10	1071	5.33
30	79.15	6004	29.88
31	80.10	2371	11.80
32	81.10	7438	37.02
33	82.10	4685	23.32
34	83.10	9102	45.30
35	84.10	5996	29.84
36	85.15	2806	13.97
37	86.10	365	1.82
38	87.10	1239	6.17
39	88.10	158	0.79
40	89.20	315	1.57
41	90.10	22	0.11
42	91.10	2814	14.00
43	92.10	747	3.72
44	93.10	2758	13.73
45	94.10	1659	8.26
46	95.15	5435	27.05
47	96.15	4246	21.13
48	97.15	7041	35.04
49	98.15	3350	16.67
50	99.15	1197	5.96
51	100.10	574	2.86
52	101.10	880	4.38
53	102.10	284	1.41
54	103.10	299	1.49
55	104.10	213	1.06
56	105.10	1208	6.01
57	106.20	428	2.13
58	107.10	1443	7.18
59	108.15	1324	6.59
60	109.15	2325	11.57
61	110.15	2140	10.65
62	111.15	2895	14.41
63	112.15	1040	5.18
64	113.20	648	3.23
65	114.15	658	3.27

#	m/z	Abs. Int.	Rel. Int.
66	115.15	880	4.38
67	116.10	310	1.54
68	117.10	470	2.34
69	118.20	150	0.75
70	119.15	1045	5.20
71	120.20	411	2.05
72	121.15	1196	5.95
73	122.20	595	2.96
74	123.15	1513	7.53
75	124.15	958	4.77
76	125.15	1069	5.32
77	126.20	467	2.32
78	127.15	706	3.51
79	128.10	384	1.91
80	129.10	659	3.28
81	130.10	164	0.82
82	131.10	383	1.91
83	132.20	34	0.17
84	133.15	780	3.88
85	134.20	428	2.13
86	135.20	829	4.13
87	136.20	420	2.09
88	137.20	676	3.36
89	138.15	556	2.77
90	139.20	569	2.83
91	140.20	260	1.29
92	141.20	379	1.89
93	142.20	79	0.39
94	143.20	234	1.16
95	144.10	37	0.18
96	145.20	213	1.06
97	146.20	6	0.03
98	147.20	599	2.98
99	148.20	317	1.58
100	149.15	584	2.91
101	150.20	254	1.26
102	151.20	668	3.32
103	152.20	455	2.26
104	153.20	343	1.71
105	154.20	108	0.54
106	155.10	173	0.86
107	156.20	28	0.14
108	157.20	145	0.72
109	158.20	35	0.17
110	159.20	101	0.50
111	161.20	299	1.49
112	162.20	21	0.10
113	163.20	240	1.19
114	164.20	82	0.41
115	165.20	389	1.94
116	166.20	347	1.73
117	167.10	171	0.85
118	168.20	57	0.28
119	169.20	113	0.56
120	171.20	121	0.60
121	172.20	13	0.06
122	173.10	66	0.33
123	174.20	38	0.19
124	175.20	146	0.73

#	m/z	Abs. Int.	Rel. Int.
125	177.20	93	0.46
126	178.20	130	0.65
127	179.20	261	1.30
128	180.20	207	1.03
129	181.20	102	0.51
130	182.10	65	0.32
131	183.20	35	0.17
132	185.20	108	0.54
133	187.10	7	0.03
134	189.10	171	0.85
135	190.20	7	0.03
136	191.20	87	0.43
137	193.20	130	0.65
138	194.20	72	0.36
139	197.20	24	0.12
140	199.20	20	0.10
141	202.10	6	0.03
142	203.20	49	0.24
143	205.10	8	0.04
144	207.10	400	1.99
145	208.20	79	0.39
146	209.20	23	0.11
147	211.20	22	0.11
148	213.20	217	1.08
149	215.10	6	0.03
150	217.20	20	0.10
151	218.20	13	0.06
152	220.20	92	0.46
153	221.20	121	0.60
154	222.20	169	0.84
155	223.20	20	0.10
156	235.20	74	0.37
157	236.20	13	0.06
158	241.20	6	0.03
159	253.20	6	0.03
160	254.20	7	0.03
161	262.10	6	0.03
162	264.20	224	1.11
163	265.20	64	0.32
164	278.10	6	0.03
165	281.20	63	0.31
166	282.10	6	0.03
167	283.20	6	0.03
168	310.20	13	0.06
169	332.20	6	0.03
170	333.20	7	0.03
171	347.20	7	0.03
172	356.10	13	0.06
173	392.20	7	0.03
174	403.10	6	0.03
175	428.10	6	0.03
176	432.20	7	0.03
177	480.10	14	0.07
178	492.20	13	0.06
179	530.10	6	0.03
180	578.20	6	0.03
181	653.20	7	0.03
182	666.20	6	0.03
183	670.20	6	0.03

Line#:6 R.Time:20.7(Scan#:5011)
MassPeaks:181
RawMode:Averaged 20.7-20.7(4998-5025) BasePeak:57(11949)
BG Mode:None Group 1 - Event 1

#	m/z	Abs. Int.	Rel. Int.
1	50.00	545	4.56
2	51.00	629	5.26
3	52.00	417	3.49
4	53.10	1272	10.65
5	54.15	1329	11.12
6	55.15	10398	87.02
7	56.15	2992	25.04
8	57.15	11949	100.00
9	58.15	798	6.68
10	59.10	788	6.59
11	60.10	6768	56.64
12	61.10	2095	17.53
13	62.10	278	2.33
14	63.20	389	3.26
15	64.10	249	2.08

#	m/z	Abs. Int.	Rel. Int.
16	65.10	750	6.28
17	66.10	472	3.95
18	67.10	3310	27.70
19	68.10	1620	13.56
20	69.10	6596	55.20
21	70.15	2390	20.00
22	71.15	6801	56.92
23	72.15	537	4.49
24	73.10	7786	65.16
25	74.10	1008	8.44
26	75.10	345	2.89
27	76.10	329	2.75
28	77.10	1585	13.26
29	78.10	599	5.01
30	79.10	2142	17.93

#	m/z	Abs. Int.	Rel. Int.
31	80.15	808	6.76
32	81.15	2866	23.99
33	82.10	1678	14.04
34	83.15	4471	37.42
35	84.15	1845	15.44
36	85.15	4341	36.33
37	86.15	504	4.22
38	87.10	2780	23.27
39	88.10	414	3.46
40	89.10	268	2.24
41	91.05	1632	13.66
42	92.10	457	3.82
43	93.15	1074	8.99
44	94.10	694	5.81
45	95.15	1945	16.28

DEPTT. OF BOTANICAL & ENVIRONMENTAL SCIENCES, G.N.D.U. AMRITSAR

#	m/z	Abs. Int.	Rel. Int.
46	96.15	1373	11.49
47	97.15	3312	27.72
48	98.15	1593	13.33
49	99.20	1427	11.94
50	100.20	299	2.50
51	101.10	803	6.72
52	102.10	391	3.27
53	103.10	295	2.47
54	104.10	173	1.45
55	105.10	1013	8.48
56	106.20	286	2.39
57	107.15	680	5.69
58	108.10	433	3.62
59	109.15	946	7.92
60	110.20	824	6.90
61	111.15	1475	12.34
62	112.15	570	4.77
63	113.20	761	6.37
64	114.10	153	1.28
65	115.15	1276	10.68
66	116.15	618	5.17
67	117.20	445	3.72
68	118.10	109	0.91
69	119.10	513	4.29
70	120.10	171	1.43
71	121.10	515	4.31
72	122.10	209	1.75
73	123.10	543	4.54
74	124.10	356	2.98
75	125.20	654	5.47
76	126.20	232	1.94
77	127.20	474	3.97
78	128.10	302	2.53
79	129.15	2333	19.52
80	130.20	415	3.47
81	131.20	281	2.35
82	132.10	49	0.41
83	133.20	398	3.33
84	134.20	122	1.02
85	135.20	376	3.15
86	136.20	199	1.67
87	137.20	165	1.38
88	138.20	175	1.46
89	139.20	241	2.02
90	140.20	94	0.79
91	141.20	377	3.16

#	m/z	Abs. Int.	Rel. Int.
92	142.10	44	0.37
93	143.15	578	4.84
94	144.10	110	0.92
95	145.10	229	1.92
96	146.10	31	0.26
97	147.10	230	1.92
98	148.10	58	0.49
99	149.20	293	2.45
100	150.10	48	0.40
101	151.10	349	2.92
102	152.20	73	0.61
103	153.10	149	1.25
104	154.20	15	0.13
105	155.20	153	1.28
106	156.20	17	0.14
107	157.10	335	2.80
108	158.10	97	0.81
109	159.10	26	0.22
110	161.10	63	0.53
111	162.10	15	0.13
112	163.10	50	0.42
113	164.10	15	0.13
114	165.20	155	1.30
115	166.20	78	0.65
116	167.10	64	0.54
117	168.20	16	0.13
118	169.20	89	0.74
119	171.20	392	3.28
120	172.20	93	0.78
121	177.20	70	0.59
122	179.10	48	0.40
123	180.10	16	0.13
124	181.20	55	0.46
125	182.10	8	0.07
126	183.10	14	0.12
127	184.10	7	0.06
128	185.20	830	6.95
129	186.20	116	0.97
130	187.10	15	0.13
131	188.20	15	0.13
132	189.10	16	0.13
133	191.10	83	0.69
134	192.10	7	0.06
135	193.20	49	0.41
136	196.20	7	0.06
137	199.20	256	2.14

#	m/z	Abs. Int.	Rel. Int.
138	200.20	34	0.28
139	203.20	15	0.13
140	205.20	15	0.13
141	207.20	327	2.74
142	208.20	15	0.13
143	209.10	8	0.07
144	213.20	142	1.19
145	214.20	31	0.26
146	215.10	15	0.13
147	219.20	16	0.13
148	221.10	14	0.12
149	222.20	30	0.25
150	227.20	207	1.73
151	241.20	430	3.60
152	242.20	71	0.59
153	255.20	72	0.60
154	258.10	15	0.13
155	266.10	17	0.14
156	268.20	7	0.06
157	277.20	7	0.06
158	281.10	40	0.33
159	283.10	8	0.07
160	284.20	214	1.79
161	285.20	27	0.23
162	290.10	8	0.07
163	314.20	8	0.07
164	320.20	7	0.06
165	333.20	14	0.12
166	368.20	7	0.06
167	374.20	7	0.06
168	420.20	8	0.07
169	424.10	7	0.06
170	437.20	7	0.06
171	442.10	7	0.06
172	473.20	7	0.06
173	487.20	7	0.06
174	488.20	8	0.07
175	511.10	16	0.13
176	528.10	7	0.06
177	549.10	7	0.06
178	582.20	7	0.06
179	660.20	8	0.07
180	668.20	7	0.06
181	672.20	7	0.06

Line#:7 R.Time:23.7(Scan#:5906)
MassPeaks:167
RawMode:Averaged 23.7-23.7(5896-5916) BasePeak:57(21258)
BG Mode:None Group 1 - Event 1

#	m/z	Abs. Int.	Rel. Int.
1	50.00	539	2.54
2	51.00	560	2.63
3	52.00	348	1.64
4	53.10	734	3.45
5	54.15	776	3.65
6	55.15	6795	31.96
7	56.15	3128	14.71
8	57.15	21258	100.00
9	58.20	1228	5.78
10	59.10	411	1.93
11	60.20	345	1.62
12	61.10	265	1.25
13	62.10	166	0.78
14	63.10	346	1.63
15	64.10	206	0.97
16	65.10	509	2.39
17	66.10	370	1.74
18	67.10	2123	9.99
19	68.15	978	4.60
20	69.15	4912	23.11
21	70.15	2645	12.44
22	71.15	14552	68.45
23	72.15	953	4.48
24	73.15	1321	6.21
25	74.10	322	1.51
26	75.10	390	1.83
27	76.10	263	1.24
28	77.10	1290	6.07

#	m/z	Abs. Int.	Rel. Int.
29	78.00	425	2.00
30	79.05	1210	5.69
31	80.10	418	1.97
32	81.10	2108	9.92
33	82.10	1351	6.36
34	83.15	3355	15.78
35	84.10	1573	7.40
36	85.15	9617	45.24
37	86.15	737	3.47
38	87.20	287	1.35
39	88.20	46	0.22
40	89.20	310	1.46
41	90.20	71	0.33
42	91.10	1064	5.01
43	92.10	347	1.63
44	93.20	676	3.18
45	94.20	380	1.79
46	95.15	1488	7.00
47	96.15	1221	5.74
48	97.15	3056	14.38
49	98.15	999	4.70
50	99.15	3723	17.51
51	100.20	296	1.39
52	101.20	92	0.43
53	102.20	51	0.24
54	103.10	314	1.48
55	104.20	30	0.14
56	105.20	950	4.47

#	m/z	Abs. Int.	Rel. Int.
57	106.20	369	1.74
58	107.20	577	2.71
59	108.20	259	1.22
60	109.20	851	4.00
61	110.20	662	3.11
62	111.20	1557	7.32
63	112.15	615	2.89
64	113.20	2089	9.83
65	114.20	205	0.96
66	115.10	468	2.20
67	116.20	117	0.55
68	117.20	416	1.96
69	118.10	45	0.21
70	119.20	646	3.04
71	120.20	288	1.35
72	121.20	401	1.89
73	122.20	158	0.74
74	123.20	477	2.24
75	124.20	322	1.51
76	125.25	762	3.58
77	126.20	340	1.60
78	127.20	1281	6.03
79	128.20	366	1.72
80	129.20	328	1.54
81	130.20	76	0.36
82	131.20	319	1.50
83	132.20	64	0.30
84	133.20	565	2.66

#	m/z	Abs. Int.	Rel. Int.
85	134.20	170	0.80
86	135.20	317	1.49
87	136.20	111	0.52
88	137.20	299	1.41
89	138.20	158	0.74
90	139.20	405	1.91
91	140.20	204	0.96
92	141.20	711	3.34
93	142.20	164	0.77
94	143.20	162	0.76
95	145.20	209	0.98
96	147.20	398	1.87
97	148.20	66	0.31
98	149.20	165	0.78
99	150.20	44	0.21
100	151.20	285	1.34
101	152.20	198	0.93
102	153.20	169	0.79
103	154.20	160	0.75
104	155.20	455	2.14
105	159.20	86	0.40
106	161.10	88	0.41
107	162.20	9	0.04
108	163.20	88	0.41
109	164.20	33	0.16
110	165.20	245	1.15
111	166.20	118	0.56
112	167.20	104	0.49
113	168.20	81	0.38
114	169.20	346	1.63
115	170.20	10	0.05
116	171.20	30	0.14
117	172.20	20	0.09
118	175.20	77	0.36
119	177.20	88	0.41
120	179.10	45	0.21
121	181.20	19	0.09
122	182.20	46	0.22
123	183.20	211	0.99
124	184.20	34	0.16
125	187.20	26	0.12
126	189.20	51	0.24
127	191.20	191	0.90
128	193.20	155	0.73
129	194.10	32	0.15
130	195.20	10	0.05
131	197.20	187	0.88
132	203.20	9	0.04
133	205.20	21	0.10
134	207.20	675	3.18
135	208.20	215	1.01
136	209.20	32	0.15
137	210.20	45	0.21
138	211.20	70	0.33
139	214.20	9	0.04
140	215.20	31	0.15
141	217.20	19	0.09
142	220.20	20	0.09
143	223.20	9	0.04
144	225.20	22	0.10
145	226.20	20	0.09
146	231.20	21	0.10
147	232.20	10	0.05
148	236.20	10	0.05
149	239.20	10	0.05
150	243.20	20	0.09
151	254.20	9	0.04
152	267.20	98	0.46
153	276.20	10	0.05
154	281.20	184	0.87
155	282.20	30	0.14
156	283.10	9	0.04
157	327.20	21	0.10
158	358.20	10	0.05
159	380.20	9	0.04
160	445.20	22	0.10
161	470.10	9	0.04
162	545.20	10	0.05
163	576.20	10	0.05
164	602.20	9	0.04
165	645.20	9	0.04
166	663.20	20	0.09
167	665.20	9	0.04

Line#:8 R.Time:23.7(Scan#:5924)
MassPeaks:171
RawMode:Averaged 23.7-23.8(5916-5934) BasePeak:149(18032)
BG Mode:None Group 1 - Event 1

#	m/z	Abs. Int.	Rel. Int.
1	50.05	1192	6.61
2	51.15	901	5.00
3	52.10	482	2.67
4	53.15	1026	5.69
5	54.15	788	4.37
6	55.15	8508	47.18
7	56.15	3548	19.68
8	57.15	17231	95.56
9	58.15	1024	5.68
10	59.10	324	1.80
11	60.20	334	1.85
12	61.20	185	1.03
13	62.20	91	0.50
14	63.20	371	2.06
15	64.10	335	1.86
16	65.10	1702	9.44
17	66.10	533	2.96
18	67.10	2249	12.47
19	68.10	1096	6.08
20	69.15	4751	26.35
21	70.15	7400	41.04
22	71.10	11108	61.60
23	72.10	813	4.51
24	73.10	1459	8.09
25	74.10	563	3.12
26	75.10	594	3.29
27	76.05	1667	9.24
28	77.10	1826	10.13
29	78.10	471	2.61
30	79.10	1280	7.10
31	80.10	466	2.58
32	81.15	2185	12.12
33	82.15	1692	9.38
34	83.15	4424	24.53
35	84.15	2147	11.91
36	85.15	2588	14.35
37	86.20	214	1.19
38	87.20	353	1.96
39	88.20	69	0.38
40	89.20	318	1.76
41	90.20	11	0.06
42	91.10	1037	5.75
43	92.10	348	1.93
44	93.10	1576	8.74
45	94.10	490	2.72
46	95.15	1676	9.29
47	96.15	1034	5.73
48	97.15	2305	12.78
49	98.20	685	3.80
50	99.15	1056	5.86
51	100.10	191	1.06
52	101.20	103	0.57
53	102.20	61	0.34
54	103.10	365	2.02
55	104.05	2246	12.46
56	105.10	1408	7.81
57	106.10	367	2.04
58	107.10	606	3.36
59	108.20	318	1.76
60	109.10	886	4.91
61	110.20	610	3.38
62	111.20	1154	6.40
63	112.20	1399	7.76
64	113.20	2641	14.65
65	114.20	320	1.77
66	115.20	421	2.33
67	116.20	86	0.48
68	117.20	409	2.27
69	118.20	204	1.13
70	119.20	734	4.07
71	120.20	305	1.69
72	121.10	1190	6.60
73	122.10	700	3.88
74	123.10	646	3.58
75	124.20	225	1.25
76	125.20	598	3.32
77	126.20	218	1.21
78	127.20	395	2.19
79	128.20	273	1.51
80	129.10	433	2.40
81	130.10	145	0.80
82	131.20	306	1.70
83	132.10	646	3.58
84	133.10	652	3.62
85	134.10	209	1.16
86	135.20	343	1.90
87	136.20	194	1.08
88	137.10	291	1.61
89	138.20	74	0.41
90	139.10	271	1.50
91	140.20	47	0.26
92	141.20	374	2.07
93	143.20	163	0.90
94	144.20	11	0.06
95	145.10	243	1.35
96	146.20	34	0.19
97	147.20	448	2.48
98	148.10	153	0.85
99	149.10	18032	100.00
100	150.10	2118	11.75
101	151.15	558	3.09
102	152.10	131	0.73
103	153.10	213	1.18
104	154.10	63	0.35
105	155.10	178	0.99
106	157.10	97	0.54
107	158.10	10	0.06
108	159.20	53	0.29
109	160.20	11	0.06
110	161.10	141	0.78
111	162.10	155	0.86
112	163.10	190	1.05
113	164.10	36	0.20
114	165.10	244	1.35
115	166.10	132	0.73
116	167.10	5571	30.90
117	168.10	510	2.83
118	169.10	165	0.92
119	171.10	13	0.07
120	175.10	57	0.32
121	176.10	21	0.12
122	177.10	118	0.65
123	179.10	202	1.12
124	180.10	61	0.34
125	184.10	10	0.06
126	185.10	12	0.07
127	189.10	69	0.38
128	190.10	25	0.14
129	191.10	288	1.60
130	193.10	81	0.45
131	195.10	26	0.14
132	196.10	22	0.12
133	197.10	57	0.32
134	203.10	54	0.30
135	207.10	825	4.58
136	208.10	128	0.71
137	209.10	109	0.60
138	211.10	11	0.06

DEPTT. OF BOTANICAL & ENVIRONMENTAL SCIENCES, G.N.D.U. AMRITSAR

#	m/z	Abs. Int.	Rel. Int.	#	m/z	Abs. Int.	Rel. Int.	#	m/z	Abs. Int.	Rel. Int.
139	217.10	10	0.06	150	268.10	10	0.06	161	312.10	36	0.20
140	219.10	24	0.13	151	269.10	11	0.06	162	340.10	11	0.06
141	220.20	11	0.06	152	276.10	21	0.12	163	388.10	10	0.06
142	221.10	11	0.06	153	279.15	482	2.67	164	392.10	23	0.13
143	225.20	24	0.13	154	280.10	80	0.44	165	427.10	12	0.07
144	227.10	11	0.06	155	281.10	266	1.48	166	431.10	11	0.06
145	238.10	23	0.13	156	282.20	11	0.06	167	471.10	11	0.06
146	242.10	10	0.06	157	283.10	50	0.28	168	541.10	23	0.13
147	249.10	68	0.38	158	292.10	11	0.06	169	542.10	11	0.06
148	253.10	10	0.06	159	293.10	11	0.06	170	591.10	11	0.06
149	265.10	11	0.06	160	296.10	10	0.06	171	663.20	11	0.06

Line#:9 R.Time:24.4(Scan#:6127)
MassPeaks:167
RawMode:Averaged 24.4-24.4(6117-6134) BasePeak:57(8212)
BG Mode:None Group 1 - Event 1

#	m/z	Abs. Int.	Rel. Int.	#	m/z	Abs. Int.	Rel. Int.	#	m/z	Abs. Int.	Rel. Int.
1	50.00	549	6.69	57	106.10	238	2.90	113	166.20	158	1.92
2	51.00	562	6.84	58	107.20	536	6.53	114	167.20	142	1.73
3	52.00	305	3.71	59	108.20	219	2.67	115	168.20	11	0.13
4	53.00	703	8.56	60	109.20	901	10.97	116	169.20	64	0.78
5	54.10	766	9.33	61	110.15	685	8.34	117	171.20	25	0.30
6	55.15	6469	78.77	62	111.15	2348	28.59	118	172.20	13	0.16
7	56.15	2559	31.16	63	112.10	553	6.73	119	173.20	30	0.37
8	57.15	8212	100.00	64	113.20	544	6.62	120	174.20	23	0.28
9	58.10	574	6.99	65	114.20	64	0.78	121	175.10	11	0.13
10	59.20	371	4.52	66	115.20	470	5.72	122	176.20	22	0.27
11	60.10	392	4.77	67	116.20	97	1.18	123	177.20	128	1.56
12	61.10	163	1.98	68	117.20	336	4.09	124	178.20	11	0.13
13	62.20	105	1.28	69	118.10	35	0.43	125	179.20	182	2.22
14	63.20	350	4.26	70	119.10	693	8.44	126	180.10	49	0.60
15	64.20	237	2.89	71	120.20	236	2.87	127	181.20	11	0.13
16	65.10	500	6.09	72	121.20	398	4.85	128	185.20	11	0.13
17	66.10	441	5.37	73	122.20	183	2.23	129	187.20	38	0.46
18	67.15	2327	28.34	74	123.10	516	6.28	130	189.20	12	0.15
19	68.15	1062	12.93	75	124.10	412	5.02	131	190.10	25	0.30
20	69.10	5167	62.92	76	125.20	1062	12.93	132	191.10	328	3.99
21	70.15	2430	29.59	77	126.20	341	4.15	133	192.20	13	0.16
22	71.15	4755	57.90	78	127.20	424	5.16	134	193.10	244	2.97
23	72.20	453	5.52	79	128.20	256	3.12	135	194.20	13	0.16
24	73.10	1607	19.57	80	129.20	374	4.55	136	195.20	14	0.17
25	74.10	351	4.27	81	130.20	60	0.73	137	196.10	12	0.15
26	75.10	405	4.93	82	131.20	287	3.49	138	197.20	37	0.45
27	76.10	316	3.85	83	132.20	12	0.15	139	203.20	30	0.37
28	77.10	1211	14.75	84	133.20	630	7.67	140	205.20	11	0.13
29	78.10	381	4.64	85	134.20	251	3.06	141	207.05	802	9.77
30	79.15	1295	15.77	86	135.20	355	4.32	142	208.20	201	2.45
31	80.20	427	5.20	87	136.20	196	2.39	143	209.20	107	1.30
32	81.15	2344	28.54	88	137.20	289	3.52	144	216.20	12	0.15
33	82.10	1685	20.52	89	138.20	167	2.03	145	218.20	11	0.13
34	83.15	4509	54.91	90	139.20	449	5.47	146	221.20	26	0.32
35	84.10	1366	16.63	91	140.20	99	1.21	147	224.20	22	0.27
36	85.15	2977	36.25	92	141.20	382	4.65	148	232.20	27	0.33
37	86.10	271	3.30	93	142.20	37	0.45	149	238.20	11	0.13
38	87.10	264	3.21	94	143.20	93	1.13	150	243.10	11	0.13
39	88.10	89	1.08	95	144.20	63	0.77	151	249.10	23	0.28
40	89.10	312	3.80	96	145.20	191	2.33	152	253.20	11	0.13
41	90.10	12	0.15	97	147.20	443	5.39	153	265.10	53	0.65
42	91.10	1188	14.47	98	148.10	59	0.72	154	276.20	12	0.15
43	92.10	318	3.87	99	149.20	206	2.51	155	281.20	277	3.37
44	93.10	708	8.62	100	150.20	23	0.28	156	282.00	24	0.29
45	94.10	448	5.46	101	151.20	306	3.73	157	302.20	11	0.13
46	95.15	1722	20.97	102	152.20	169	2.06	158	312.20	12	0.15
47	96.15	1542	18.78	103	153.20	284	3.46	159	326.20	12	0.15
48	97.15	4432	53.97	104	154.20	67	0.82	160	341.20	13	0.16
49	98.15	950	11.57	105	155.20	123	1.50	161	356.20	25	0.30
50	99.15	1040	12.66	106	157.10	89	1.08	162	384.20	11	0.13
51	100.20	37	0.45	107	159.20	25	0.30	163	510.20	13	0.16
52	101.20	135	1.64	108	161.10	25	0.30	164	523.20	11	0.13
53	102.10	81	0.99	109	162.20	37	0.45	165	525.20	25	0.30
54	103.20	348	4.24	110	163.20	123	1.50	166	642.20	13	0.16
55	104.20	108	1.32	111	164.20	25	0.30	167	683.20	11	0.13
56	105.10	796	9.69	112	165.10	194	2.36				

Line#:10 R.Time:25.4(Scan#:6419)
MassPeaks:207
RawMode:Averaged 25.3-25.4(6405-6433) BasePeak:57(54064)
BG Mode:None Group 1 - Event 1

#	m/z	Abs. Int.	Rel. Int.	#	m/z	Abs. Int.	Rel. Int.	#	m/z	Abs. Int.	Rel. Int.
1	50.00	479	0.89	2	51.00	528	0.98	3	52.00	352	0.65

DEPTT. OF BOTANICAL & ENVIRONMENTAL SCIENCES, G.N.D.U. AMRITSAR

#	m/z	Abs. Int.	Rel. Int.	#	m/z	Abs. Int.	Rel. Int.	#	m/z	Abs. Int.	Rel. Int.
4	53.15	1092	2.02	72	121.20	452	0.84	140	194.30	99	0.18
5	54.15	1369	2.53	73	122.20	230	0.43	141	195.30	66	0.12
6	55.15	14009	25.91	74	123.20	623	1.15	142	196.30	116	0.21
7	56.15	7131	13.19	75	124.20	616	1.14	143	197.20	487	0.90
8	57.15	54064	100.00	76	125.20	1822	3.37	144	198.30	72	0.13
9	58.15	3340	6.18	77	126.20	953	1.76	145	199.30	28	0.05
10	59.15	1363	2.52	78	127.20	3802	7.03	146	201.30	7	0.01
11	60.10	326	0.60	79	128.20	589	1.09	147	203.30	34	0.06
12	61.20	222	0.41	80	129.20	359	0.66	148	205.30	70	0.13
13	62.20	95	0.18	81	130.20	44	0.08	149	207.15	1067	1.97
14	63.20	276	0.51	82	131.20	334	0.62	150	208.10	306	0.57
15	64.10	165	0.31	83	132.20	30	0.06	151	209.10	142	0.26
16	65.10	523	0.97	84	133.10	622	1.15	152	210.20	94	0.17
17	66.10	438	0.81	85	134.20	327	0.60	153	211.20	304	0.56
18	67.10	3020	5.59	86	135.20	404	0.75	154	212.20	18	0.03
19	68.10	1757	3.25	87	136.20	134	0.25	155	213.20	21	0.04
20	69.15	9580	17.72	88	137.20	293	0.54	156	215.10	14	0.03
21	70.15	5822	10.77	89	138.20	363	0.67	157	219.10	9	0.02
22	71.15	38946	72.04	90	139.20	750	1.39	158	221.20	28	0.05
23	72.15	2259	4.18	91	140.20	590	1.09	159	222.30	15	0.03
24	73.10	1653	3.06	92	141.20	2489	4.60	160	223.00	25	0.05
25	74.10	301	0.56	93	142.20	334	0.62	161	224.20	69	0.13
26	75.10	402	0.74	94	143.20	152	0.28	162	225.20	247	0.46
27	76.10	242	0.45	95	145.20	242	0.45	163	226.20	8	0.01
28	77.05	1271	2.35	96	146.10	7	0.01	164	227.20	7	0.01
29	78.10	457	0.85	97	147.10	356	0.66	165	229.10	7	0.01
30	79.15	1316	2.43	98	148.20	71	0.13	166	233.20	7	0.01
31	80.10	446	0.82	99	149.20	270	0.50	167	236.30	7	0.01
32	81.15	2654	4.91	100	150.20	80	0.15	168	237.20	23	0.04
33	82.15	2592	4.79	101	151.20	347	0.64	169	238.20	27	0.05
34	83.10	7248	13.41	102	152.20	304	0.56	170	239.10	137	0.25
35	84.15	3250	6.01	103	153.20	460	0.85	171	240.10	14	0.03
36	85.15	25760	47.65	104	154.20	394	0.73	172	242.20	7	0.01
37	86.15	1751	3.24	105	155.25	1518	2.81	173	249.10	15	0.03
38	87.20	268	0.50	106	156.20	225	0.42	174	250.10	7	0.01
39	88.20	124	0.23	107	157.30	47	0.09	175	252.10	102	0.19
40	89.20	220	0.41	108	159.20	157	0.29	176	253.20	123	0.23
41	90.10	15	0.03	109	160.10	8	0.01	177	254.10	17	0.03
42	91.10	1050	1.94	110	161.30	175	0.32	178	255.10	6	0.01
43	92.20	376	0.70	111	162.20	13	0.02	179	265.00	15	0.03
44	93.10	725	1.34	112	163.20	193	0.36	180	266.20	6	0.01
45	94.20	431	0.80	113	164.30	7	0.01	181	267.20	136	0.25
46	95.15	1841	3.41	114	165.20	188	0.35	182	272.10	15	0.03
47	96.15	2325	4.30	115	166.30	186	0.34	183	280.20	8	0.01
48	97.15	6835	12.64	116	167.30	260	0.48	184	281.10	447	0.83
49	98.15	2235	4.13	117	168.20	232	0.43	185	282.00	43	0.08
50	99.20	10708	19.81	118	169.25	1024	1.89	186	283.20	7	0.01
51	100.15	869	1.61	119	170.30	118	0.22	187	287.10	7	0.01
52	101.20	131	0.24	120	171.30	39	0.07	188	296.20	7	0.01
53	102.10	39	0.07	121	172.10	6	0.01	189	299.30	7	0.01
54	103.20	297	0.55	122	173.30	23	0.04	190	308.10	7	0.01
55	104.10	147	0.27	123	174.20	6	0.01	191	309.20	23	0.04
56	105.15	861	1.59	124	175.10	69	0.13	192	323.20	14	0.03
57	106.10	362	0.67	125	176.30	14	0.03	193	336.20	7	0.01
58	107.20	495	0.92	126	177.20	51	0.09	194	355.00	7	0.01
59	108.20	246	0.46	127	178.20	7	0.01	195	356.10	7	0.01
60	109.15	1025	1.90	128	179.20	76	0.14	196	380.20	16	0.03
61	110.15	1134	2.10	129	180.30	98	0.18	197	384.10	7	0.01
62	111.15	3613	6.68	130	181.30	120	0.22	198	413.20	7	0.01
63	112.20	1429	2.64	131	182.20	262	0.48	199	518.10	7	0.01
64	113.20	6271	11.60	132	183.30	627	1.16	200	531.00	7	0.01
65	114.20	644	1.19	133	184.30	115	0.21	201	543.20	16	0.03
66	115.10	470	0.87	134	185.20	14	0.03	202	546.20	15	0.03
67	116.10	78	0.14	135	189.20	62	0.11	203	587.10	6	0.01
68	117.10	392	0.73	136	190.30	7	0.01	204	606.20	7	0.01
69	118.20	88	0.16	137	191.10	323	0.60	205	609.00	7	0.01
70	119.20	669	1.24	138	192.10	15	0.03	206	657.10	7	0.01
71	120.20	279	0.52	139	193.20	232	0.43	207	688.20	15	0.03

Line#:11 R.Time:26.6(Scan#:6775)
MassPeaks:220
RawMode:Averaged 26.5-26.6(6754-6794) BasePeak:69(104589)
BG Mode:None Group 1 - Event 1

#	m/z	Abs. Int.	Rel. Int.	#	m/z	Abs. Int.	Rel. Int.	#	m/z	Abs. Int.	Rel. Int.
1	50.10	674	0.64	7	56.15	1525	1.46	13	62.10	191	0.18
2	51.15	1003	0.96	8	57.15	7203	6.89	14	63.10	426	0.41
3	52.15	595	0.57	9	58.10	515	0.49	15	64.10	218	0.21
4	53.10	4946	4.73	10	59.10	339	0.32	16	65.10	1526	1.46
5	54.15	883	0.84	11	60.10	347	0.33	17	66.15	770	0.74
6	55.15	10499	10.04	12	61.20	193	0.18	18	67.10	13261	12.68

DEPTT. OF BOTANICAL & ENVIRONMENTAL SCIENCES, G.N.D.U. AMRITSAR

#	m/z	Abs. Int.	Rel. Int.
19	68.15	11584	11.08
20	69.15	104589	100.00
21	70.15	6455	6.17
22	71.15	3770	3.60
23	72.10	271	0.26
24	73.15	1507	1.44
25	74.10	313	0.30
26	75.10	379	0.36
27	76.10	291	0.28
28	77.10	3614	3.46
29	78.10	924	0.88
30	79.10	6810	6.51
31	80.10	2735	2.61
32	81.10	60659	58.00
33	82.10	6518	6.23
34	83.10	4168	3.99
35	84.10	783	0.75
36	85.15	1849	1.77
37	86.20	176	0.17
38	87.10	249	0.24
39	88.20	31	0.03
40	89.10	252	0.24
41	90.10	33	0.03
42	91.10	4847	4.63
43	92.10	2050	1.96
44	93.10	11814	11.30
45	94.10	4301	4.11
46	95.15	19810	18.94
47	96.15	2658	2.54
48	97.15	2812	2.69
49	98.10	533	0.51
50	99.15	736	0.70
51	100.20	30	0.03
52	101.20	51	0.05
53	102.20	109	0.10
54	103.10	545	0.52
55	104.10	249	0.24
56	105.15	3811	3.64
57	106.15	1255	1.20
58	107.15	7865	7.52
59	108.15	2204	2.11
60	109.15	7896	7.55
61	110.15	1213	1.16
62	111.15	1651	1.58
63	112.20	294	0.28
64	113.20	487	0.47
65	114.20	26	0.02
66	115.05	621	0.59
67	116.10	201	0.19
68	117.10	691	0.66
69	118.10	197	0.19
70	119.15	3750	3.59
71	120.15	1223	1.17
72	121.15	10215	9.77
73	122.15	2380	2.28
74	123.15	6442	6.16
75	124.20	923	0.88
76	125.20	718	0.69
77	126.20	133	0.13
78	127.20	396	0.38
79	128.20	335	0.32
80	129.20	439	0.42
81	130.20	99	0.09
82	131.15	599	0.57
83	132.10	229	0.22
84	133.15	2432	2.33
85	134.15	1549	1.48
86	135.15	4476	4.28

#	m/z	Abs. Int.	Rel. Int.
87	136.20	6293	6.02
88	137.20	5662	5.41
89	138.20	757	0.72
90	139.20	287	0.27
91	140.20	21	0.02
92	141.20	339	0.32
93	142.20	96	0.09
94	143.20	281	0.27
95	144.20	91	0.09
96	145.15	722	0.69
97	146.20	226	0.22
98	147.20	2603	2.49
99	148.20	922	0.88
100	149.20	4454	4.26
101	150.20	766	0.73
102	151.20	473	0.45
103	152.20	122	0.12
104	153.20	160	0.15
105	154.20	13	0.01
106	155.20	152	0.15
107	156.20	16	0.02
108	157.20	129	0.12
109	158.20	34	0.03
110	159.15	574	0.55
111	160.20	192	0.18
112	161.20	1525	1.46
113	162.20	567	0.54
114	163.20	1159	1.11
115	164.20	181	0.17
116	165.20	283	0.27
117	166.20	27	0.03
118	167.20	61	0.06
119	169.20	60	0.06
120	170.20	10	0.01
121	171.20	69	0.07
122	172.20	17	0.02
123	173.20	372	0.36
124	174.20	116	0.11
125	175.20	1241	1.19
126	176.20	441	0.42
127	177.20	855	0.82
128	178.20	215	0.21
129	179.20	175	0.17
130	180.20	51	0.05
131	181.20	66	0.06
132	183.20	73	0.07
133	185.20	60	0.06
134	187.20	299	0.29
135	188.10	135	0.13
136	189.20	983	0.94
137	190.25	336	0.32
138	191.20	1010	0.97
139	192.20	495	0.47
140	193.20	343	0.33
141	195.20	15	0.01
142	197.20	32	0.03
143	199.20	5	0.00
144	200.10	5	0.00
145	201.20	206	0.20
146	202.20	115	0.11
147	203.20	903	0.86
148	204.20	313	0.30
149	205.20	300	0.29
150	206.20	55	0.05
151	207.05	1214	1.16
152	208.10	264	0.25
153	209.10	178	0.17
154	211.00	21	0.02

#	m/z	Abs. Int.	Rel. Int.
155	212.20	10	0.01
156	213.10	58	0.06
157	215.10	204	0.20
158	216.10	109	0.10
159	217.20	390	0.37
160	218.20	171	0.16
161	219.20	91	0.09
162	220.20	5	0.00
163	221.00	37	0.04
164	223.10	16	0.02
165	224.10	10	0.01
166	227.20	23	0.02
167	228.20	5	0.00
168	229.20	175	0.17
169	230.10	71	0.07
170	231.20	341	0.33
171	232.20	54	0.05
172	233.20	28	0.03
173	235.10	10	0.01
174	241.10	16	0.02
175	243.20	76	0.07
176	244.20	22	0.02
177	245.20	74	0.07
178	246.20	10	0.01
179	249.00	4	0.00
180	251.20	22	0.02
181	253.10	11	0.01
182	255.20	21	0.02
183	257.20	112	0.11
184	258.20	36	0.03
185	259.20	79	0.08
186	260.20	48	0.05
187	265.00	60	0.06
188	267.20	58	0.06
189	271.20	75	0.07
190	272.20	41	0.04
191	273.20	91	0.09
192	281.20	359	0.34
193	282.00	59	0.06
194	283.10	10	0.01
195	285.10	23	0.02
196	287.20	5	0.00
197	297.20	16	0.02
198	299.20	71	0.07
199	325.20	4	0.00
200	326.20	4	0.00
201	327.10	10	0.01
202	328.20	35	0.03
203	341.20	172	0.16
204	342.20	23	0.02
205	354.10	10	0.01
206	367.20	76	0.07
207	368.20	10	0.01
208	395.20	5	0.00
209	401.20	5	0.00
210	419.10	4	0.00
211	427.10	10	0.01
212	453.00	5	0.00
213	463.10	4	0.00
214	482.10	4	0.00
215	495.00	5	0.00
216	584.10	4	0.00
217	589.00	5	0.00
218	606.20	5	0.00
219	613.10	4	0.00
220	643.00	5	0.00

Line#:12 R.Time:27.5(Scan#:7060)
MassPeaks:204
RawMode:Averaged 27.5-27.6(7041-7088) BasePeak:57(11111)
BG Mode:None Group 1 - Event 1

#	m/z	Abs. Int.	Rel. Int.
1	50.00	490	4.41
2	51.00	525	4.73
3	52.00	377	3.39
4	53.15	825	7.43
5	54.15	940	8.46
6	55.15	8393	75.54

#	m/z	Abs. Int.	Rel. Int.
7	56.15	3283	29.55
8	57.15	11111	100.00
9	58.20	704	6.34
10	59.10	327	2.94
11	60.10	331	2.98
12	61.20	204	1.84

#	m/z	Abs. Int.	Rel. Int.
13	62.10	130	1.17
14	63.20	336	3.02
15	64.20	184	1.66
16	65.20	518	4.66
17	66.10	565	5.09
18	67.10	3301	29.71

DEPTT. OF BOTANICAL & ENVIRONMENTAL SCIENCES, G.N.D.U. AMRITSAR

#	m/z	Abs. Int.	Rel. Int.
19	68.10	2722	24.50
20	69.15	7356	66.20
21	70.15	3082	27.74
22	71.15	6073	54.66
23	72.10	469	4.22
24	73.15	1691	15.22
25	74.10	305	2.75
26	75.10	414	3.73
27	76.10	254	2.29
28	77.10	1275	11.48
29	78.10	431	3.88
30	79.10	1330	11.97
31	80.10	493	4.44
32	81.15	3386	30.47
33	82.15	3918	35.26
34	83.15	6662	59.96
35	84.15	1838	16.54
36	85.15	3650	32.85
37	86.20	333	3.00
38	87.20	253	2.28
39	88.10	42	0.38
40	89.10	281	2.53
41	90.20	27	0.24
42	91.10	1131	10.18
43	92.10	306	2.75
44	93.10	855	7.70
45	94.10	490	4.41
46	95.15	2349	21.14
47	96.15	2884	25.96
48	97.15	6300	56.70
49	98.20	1246	11.21
50	99.20	1284	11.56
51	100.20	87	0.78
52	101.10	38	0.34
53	102.20	39	0.35
54	103.10	352	3.17
55	104.20	131	1.18
56	105.10	755	6.80
57	106.20	278	2.50
58	107.20	603	5.43
59	108.20	299	2.69
60	109.20	1239	11.15
61	110.15	1061	9.55
62	111.20	3190	28.71
63	112.20	715	6.44
64	113.15	667	6.00
65	114.20	18	0.16
66	115.10	421	3.79
67	116.10	91	0.82
68	117.20	361	3.25
69	118.10	77	0.69
70	119.10	665	5.99
71	120.10	246	2.21
72	121.10	515	4.64
73	122.20	226	2.03
74	123.25	772	6.95
75	124.20	677	6.09
76	125.20	1526	13.73
77	126.20	402	3.62
78	127.20	475	4.28
79	128.20	245	2.21
80	129.20	316	2.84

#	m/z	Abs. Int.	Rel. Int.
81	130.20	43	0.39
82	131.20	311	2.80
83	132.20	32	0.29
84	133.10	664	5.98
85	134.20	228	2.05
86	135.20	413	3.72
87	136.20	266	2.39
88	137.20	377	3.39
89	138.20	366	3.29
90	139.20	669	6.02
91	140.20	203	1.83
92	141.20	338	3.04
93	142.20	28	0.25
94	143.20	70	0.63
95	144.20	14	0.13
96	145.20	248	2.23
97	146.20	53	0.48
98	147.20	392	3.53
99	148.20	30	0.27
100	149.20	309	2.78
101	150.20	73	0.66
102	151.20	344	3.10
103	152.20	245	2.21
104	153.20	358	3.22
105	154.20	112	1.01
106	155.20	207	1.86
107	156.10	9	0.08
108	157.20	36	0.32
109	158.10	4	0.04
110	159.20	141	1.27
111	160.20	4	0.04
112	161.20	87	0.78
113	162.20	20	0.18
114	163.20	204	1.84
115	164.20	32	0.29
116	165.10	280	2.52
117	166.20	189	1.70
118	167.20	252	2.27
119	168.20	71	0.64
120	169.20	118	1.06
121	171.20	14	0.13
122	173.20	41	0.37
123	174.20	4	0.04
124	175.20	73	0.66
125	176.20	18	0.16
126	177.20	144	1.30
127	178.20	38	0.34
128	179.20	152	1.37
129	180.20	40	0.36
130	181.20	119	1.07
131	182.20	13	0.12
132	183.20	34	0.31
133	184.20	4	0.04
134	185.20	13	0.12
135	187.20	39	0.35
136	188.20	4	0.04
137	189.20	66	0.59
138	190.20	4	0.04
139	191.20	320	2.88
140	192.20	26	0.23
141	193.20	254	2.29
142	194.20	18	0.16

#	m/z	Abs. Int.	Rel. Int.
143	195.20	98	0.88
144	197.20	4	0.04
145	200.20	4	0.04
146	201.20	9	0.08
147	202.20	14	0.13
148	203.20	75	0.68
149	204.20	9	0.08
150	205.20	23	0.21
151	207.10	1113	10.02
152	208.10	259	2.33
153	209.00	166	1.49
154	210.10	4	0.04
155	211.20	8	0.07
156	213.10	5	0.05
157	214.00	4	0.04
158	221.10	14	0.13
159	224.20	4	0.04
160	225.20	4	0.04
161	227.00	8	0.07
162	228.20	9	0.08
163	231.10	9	0.08
164	233.10	14	0.13
165	234.00	8	0.07
166	235.20	4	0.04
167	238.10	9	0.08
168	245.20	4	0.04
169	248.00	4	0.04
170	249.00	36	0.32
171	250.10	20	0.18
172	251.10	18	0.16
173	254.10	4	0.04
174	265.10	18	0.16
175	266.20	4	0.04
176	267.00	38	0.34
177	268.10	4	0.04
178	275.10	4	0.04
179	277.10	13	0.12
180	281.10	394	3.55
181	282.20	61	0.55
182	288.00	8	0.07
183	306.10	4	0.04
184	315.20	4	0.04
185	323.10	4	0.04
186	326.10	4	0.04
187	328.20	9	0.08
188	330.10	4	0.04
189	335.20	4	0.04
190	338.20	4	0.04
191	358.10	9	0.08
192	374.10	4	0.04
193	401.10	4	0.04
194	446.10	4	0.04
195	447.00	4	0.04
196	510.10	4	0.04
197	512.10	4	0.04
198	527.10	4	0.04
199	586.20	9	0.08
200	640.10	4	0.04
201	644.00	4	0.04
202	660.10	4	0.04
203	677.10	4	0.04
204	693.10	4	0.04

Line#:13 R.Time:27.8(Scan#:7129)
MassPeaks:210
RawMode:Averaged 27.7-27.8(7109-7144) BasePeak:57(43140)
BG Mode:None Group 1 - Event 1

#	m/z	Abs. Int.	Rel. Int.
1	50.00	492	1.14
2	51.00	522	1.21
3	52.00	364	0.84
4	53.10	1058	2.45
5	54.15	1218	2.82
6	55.15	11883	27.55
7	56.15	6017	13.95
8	57.20	43140	100.00
9	58.15	4367	10.12
10	59.10	3436	7.96
11	60.10	367	0.85
12	61.10	99	0.23

#	m/z	Abs. Int.	Rel. Int.
13	62.10	72	0.17
14	63.10	270	0.63
15	64.10	150	0.35
16	65.10	470	1.09
17	66.10	388	0.90
18	67.20	2744	6.36
19	68.15	1792	4.15
20	69.15	9343	21.66
21	70.15	4461	10.34
22	71.20	33342	77.29
23	72.20	1772	4.11
24	73.10	1440	3.34

#	m/z	Abs. Int.	Rel. Int.
25	74.10	256	0.59
26	75.10	301	0.70
27	76.10	256	0.59
28	77.10	1247	2.89
29	78.10	420	0.97
30	79.10	1364	3.16
31	80.10	494	1.15
32	81.10	3223	7.47
33	82.20	2685	6.22
34	83.15	6640	15.39
35	84.20	2722	6.31
36	85.20	21097	48.90

DEPTT. OF BOTANICAL & ENVIRONMENTAL SCIENCES, G.N.D.U. AMRITSAR

#	m/z	Abs. Int.	Rel. Int.	#	m/z	Abs. Int.	Rel. Int.	#	m/z	Abs. Int.	Rel. Int.
37	86.15	1374	3.18	95	145.20	239	0.55	153	205.20	19	0.04
38	87.10	240	0.56	96	146.20	5	0.01	154	206.20	11	0.03
39	88.10	64	0.15	97	147.10	461	1.07	155	207.05	1123	2.60
40	89.20	199	0.46	98	148.10	61	0.14	156	208.10	303	0.70
41	91.15	1142	2.65	99	149.20	381	0.88	157	209.10	192	0.45
42	92.10	313	0.73	100	150.10	76	0.18	158	210.10	118	0.27
43	93.15	1101	2.55	101	151.20	310	0.72	159	211.10	319	0.74
44	94.10	466	1.08	102	152.20	260	0.60	160	212.10	30	0.07
45	95.15	2275	5.27	103	153.20	439	1.02	161	215.20	17	0.04
46	96.10	2520	5.84	104	154.20	380	0.88	162	216.20	6	0.01
47	97.15	6458	14.97	105	155.25	1418	3.29	163	217.10	26	0.06
48	98.15	1847	4.28	106	156.20	184	0.43	164	218.20	5	0.01
49	99.15	8899	20.63	107	157.20	118	0.27	165	221.10	7	0.02
50	100.20	784	1.82	108	158.20	11	0.03	166	222.10	40	0.09
51	101.20	83	0.19	109	159.20	168	0.39	167	223.10	33	0.08
52	102.20	13	0.03	110	160.10	19	0.04	168	224.10	56	0.13
53	103.20	265	0.61	111	161.20	196	0.45	169	225.10	219	0.51
54	104.20	113	0.26	112	162.20	18	0.04	170	226.10	5	0.01
55	105.15	832	1.93	113	163.10	271	0.63	171	227.10	19	0.04
56	106.20	192	0.45	114	164.20	6	0.01	172	229.10	6	0.01
57	107.15	825	1.91	115	165.20	220	0.51	173	231.10	12	0.03
58	108.10	345	0.80	116	166.30	226	0.52	174	233.10	12	0.03
59	109.20	1214	2.81	117	167.20	246	0.57	175	237.20	17	0.04
60	110.15	1165	2.70	118	168.20	246	0.57	176	238.10	48	0.11
61	111.20	3079	7.14	119	169.25	986	2.29	177	239.10	156	0.36
62	112.20	1255	2.91	120	170.20	132	0.31	178	241.10	12	0.03
63	113.20	5256	12.18	121	171.30	21	0.05	179	249.10	13	0.03
64	114.20	563	1.31	122	172.30	13	0.03	180	250.20	11	0.03
65	115.20	405	0.94	123	173.20	65	0.15	181	251.10	19	0.04
66	116.20	66	0.15	124	174.20	6	0.01	182	252.10	31	0.07
67	117.20	367	0.85	125	175.30	139	0.32	183	253.10	102	0.24
68	118.20	45	0.10	126	176.20	37	0.09	184	264.10	5	0.01
69	119.15	666	1.54	127	177.20	110	0.25	185	265.00	52	0.12
70	120.20	241	0.56	128	178.10	25	0.06	186	266.10	17	0.04
71	121.15	596	1.38	129	179.20	94	0.22	187	267.10	175	0.41
72	122.20	297	0.69	130	180.30	119	0.28	188	268.10	18	0.04
73	123.30	658	1.53	131	181.30	99	0.23	189	281.10	451	1.05
74	124.20	741	1.72	132	182.30	187	0.43	190	282.10	33	0.08
75	125.20	1772	4.11	133	183.30	669	1.55	191	283.20	30	0.07
76	126.20	918	2.13	134	184.30	90	0.21	192	286.20	6	0.01
77	127.20	3588	8.32	135	185.20	18	0.04	193	292.10	5	0.01
78	128.20	569	1.32	136	186.20	13	0.03	194	295.10	18	0.04
79	129.20	265	0.61	137	187.20	23	0.05	195	299.10	5	0.01
80	130.20	44	0.10	138	188.10	6	0.01	196	304.10	5	0.01
81	131.10	341	0.79	139	189.20	146	0.34	197	306.20	6	0.01
82	132.10	63	0.15	140	190.10	12	0.03	198	309.30	7	0.02
83	133.10	641	1.49	141	191.20	347	0.80	199	352.10	6	0.01
84	134.20	237	0.55	142	192.30	24	0.06	200	355.20	18	0.04
85	135.10	475	1.10	143	193.10	197	0.46	201	394.10	5	0.01
86	136.10	255	0.59	144	194.20	39	0.09	202	459.10	6	0.01
87	137.20	424	0.98	145	195.30	134	0.31	203	464.30	5	0.01
88	138.20	423	0.98	146	196.30	148	0.34	204	496.20	5	0.01
89	139.20	817	1.89	147	197.25	481	1.11	205	533.00	5	0.01
90	140.20	526	1.22	148	198.30	56	0.13	206	592.10	5	0.01
91	141.20	2201	5.10	149	199.30	5	0.01	207	629.20	5	0.01
92	142.20	261	0.61	150	201.20	5	0.01	208	643.20	6	0.01
93	143.10	152	0.35	151	203.20	81	0.19	209	676.20	5	0.01
94	144.20	11	0.03	152	204.10	5	0.01	210	698.20	5	0.01

Line#:14 R.Time:31.2(Scan#:8147)
MassPeaks:278
RawMode:Averaged 31.0-31.3(8110-8179) BasePeak:165(40621)
BG Mode:None Group 1 - Event 1

#	m/z	Abs. Int.	Rel. Int.	#	m/z	Abs. Int.	Rel. Int.	#	m/z	Abs. Int.	Rel. Int.
1	50.00	573	1.41	17	66.10	1115	2.74	33	82.15	9533	23.47
2	51.05	764	1.88	18	67.10	7774	19.14	34	83.15	15659	38.55
3	52.15	505	1.24	19	68.10	6237	15.35	35	84.15	4015	9.88
4	53.10	2006	4.94	20	69.10	17123	42.15	36	85.15	8195	20.17
5	54.15	2154	5.30	21	70.15	6829	16.81	37	86.10	643	1.58
6	55.15	22151	54.53	22	71.15	15433	37.99	38	87.20	254	0.63
7	56.15	8291	20.41	23	72.15	1084	2.67	39	88.20	43	0.11
8	57.15	31717	78.08	24	73.10	1711	4.21	40	89.20	223	0.55
9	58.15	1674	4.12	25	74.10	325	0.80	41	90.10	39	0.10
10	59.10	381	0.94	26	75.10	400	0.98	42	91.10	3426	8.43
11	60.10	380	0.94	27	76.10	301	0.74	43	92.10	598	1.47
12	61.20	230	0.57	28	77.05	2243	5.52	44	93.10	2213	5.45
13	62.10	107	0.26	29	78.10	638	1.57	45	94.15	882	2.17
14	63.20	369	0.91	30	79.10	2823	6.95	46	95.15	5306	13.06
15	64.10	262	0.64	31	80.15	946	2.33	47	96.15	6774	16.68
16	65.10	963	2.37	32	81.15	7458	18.36	48	97.15	14956	36.82

DEPTT. OF BOTANICAL & ENVIRONMENTAL SCIENCES, G.N.D.U. AMRITSAR

#	m/z	Abs. Int.	Rel. Int.
49	98.15	2642	6.50
50	99.15	2651	6.53
51	100.20	263	0.65
52	101.20	25	0.06
53	102.20	93	0.23
54	103.05	516	1.27
55	104.10	199	0.49
56	105.10	1968	4.84
57	106.10	471	1.16
58	107.10	2138	5.26
59	108.20	787	1.94
60	109.15	3806	9.37
61	110.20	2375	5.85
62	111.15	7684	18.92
63	112.20	1595	3.93
64	113.20	1470	3.62
65	114.20	161	0.40
66	115.15	748	1.84
67	116.10	280	0.69
68	117.10	754	1.86
69	118.10	223	0.55
70	119.15	2115	5.21
71	120.20	518	1.28
72	121.15	4890	12.04
73	122.15	1527	3.76
74	123.15	1848	4.55
75	124.20	1743	4.29
76	125.20	4047	9.96
77	126.20	990	2.44
78	127.15	1014	2.50
79	128.20	480	1.18
80	129.20	546	1.34
81	130.20	150	0.37
82	131.15	728	1.79
83	132.10	233	0.57
84	133.10	1176	2.90
85	134.20	496	1.22
86	135.10	2069	5.09
87	136.15	3556	8.75
88	137.15	3514	8.65
89	138.15	1536	3.78
90	139.20	1855	4.57
91	140.15	569	1.40
92	141.20	741	1.82
93	142.20	196	0.48
94	143.20	345	0.85
95	144.20	169	0.42
96	145.15	614	1.51
97	146.10	261	0.64
98	147.15	1167	2.87
99	148.10	360	0.89
100	149.15	1819	4.48
101	150.10	337	0.83
102	151.15	846	2.08
103	152.20	1349	3.32
104	153.20	1073	2.64
105	154.20	363	0.89
106	155.20	477	1.17
107	156.20	82	0.20
108	157.20	206	0.51
109	158.20	65	0.16
110	159.15	621	1.53
111	160.10	219	0.54
112	161.15	818	2.01
113	162.15	620	1.53
114	163.15	893	2.20
115	164.15	16806	41.37
116	165.15	40621	100.00
117	166.20	5357	13.19
118	167.20	992	2.44
119	168.20	242	0.60
120	169.20	284	0.70
121	170.20	22	0.05
122	171.20	157	0.39
123	172.20	152	0.37
124	173.20	374	0.92
125	174.20	163	0.40

#	m/z	Abs. Int.	Rel. Int.
126	175.15	834	2.05
127	176.10	589	1.45
128	177.15	1258	3.10
129	178.15	499	1.23
130	179.20	394	0.97
131	180.20	243	0.60
132	181.20	357	0.88
133	182.20	140	0.34
134	183.20	150	0.37
135	184.20	3	0.01
136	185.20	112	0.28
137	186.20	57	0.14
138	187.15	514	1.27
139	188.20	177	0.44
140	189.10	928	2.28
141	190.15	768	1.89
142	191.15	1030	2.54
143	192.10	198	0.49
144	193.10	305	0.75
145	194.20	171	0.42
146	195.20	269	0.66
147	196.20	107	0.26
148	197.10	104	0.26
149	198.20	12	0.03
150	199.20	15	0.04
151	200.10	6	0.01
152	201.20	103	0.25
153	202.10	53	0.13
154	203.15	946	2.33
155	204.15	362	0.89
156	205.15	4809	11.84
157	206.15	825	2.03
158	207.05	1556	3.83
159	208.10	439	1.08
160	209.10	299	0.74
161	210.10	117	0.29
162	211.10	70	0.17
163	212.10	2	0.00
164	213.10	19	0.05
165	214.10	3	0.01
166	215.10	90	0.22
167	216.10	6	0.01
168	217.10	91	0.22
169	218.10	116	0.29
170	219.10	250	0.62
171	220.10	89	0.22
172	221.10	71	0.17
173	222.10	93	0.23
174	223.10	94	0.23
175	224.10	27	0.07
176	225.10	20	0.05
177	226.10	3	0.01
178	229.10	15	0.04
179	230.10	2	0.00
180	231.10	15	0.04
181	232.10	16	0.04
182	233.10	6	0.01
183	235.10	17	0.04
184	236.00	44	0.11
185	237.10	35	0.09
186	238.10	21	0.05
187	239.10	48	0.12
188	241.10	7	0.02
189	243.10	9	0.02
190	245.10	9	0.02
191	246.10	9	0.02
192	247.10	7	0.02
193	248.10	2	0.00
194	249.10	52	0.13
195	250.10	36	0.09
196	251.10	23	0.06
197	253.10	12	0.03
198	255.10	5	0.01
199	259.10	2	0.00
200	260.10	22	0.05
201	261.10	16	0.04
202	264.10	19	0.05

#	m/z	Abs. Int.	Rel. Int.
203	265.10	117	0.29
204	266.10	23	0.06
205	267.10	106	0.26
206	268.10	3	0.01
207	269.00	3	0.01
208	274.10	44	0.11
209	275.00	3	0.01
210	278.10	6	0.01
211	279.10	6	0.01
212	280.10	9	0.02
213	281.10	459	1.13
214	282.10	78	0.19
215	283.10	18	0.04
216	288.10	68	0.17
217	297.10	3	0.01
218	298.10	2	0.00
219	302.10	35	0.09
220	303.10	6	0.01
221	305.10	2	0.00
222	308.10	2	0.00
223	309.10	2	0.00
224	316.10	6	0.01
225	318.10	12	0.03
226	319.00	3	0.01
227	322.10	2	0.00
228	323.10	3	0.01
229	326.10	3	0.01
230	330.10	2	0.00
231	340.10	3	0.01
232	341.10	3	0.01
233	353.10	2	0.00
234	355.10	21	0.05
235	356.10	2	0.00
236	358.10	6	0.01
237	359.10	2	0.00
238	361.10	2	0.00
239	362.10	2	0.00
240	365.00	3	0.01
241	372.10	3	0.01
242	375.10	3	0.01
243	381.10	2	0.00
244	383.10	6	0.01
245	386.10	2	0.00
246	387.10	35	0.09
247	389.10	2	0.00
248	395.10	5	0.01
249	408.10	3	0.01
250	428.10	6	0.01
251	429.40	13	0.03
252	430.40	6876	16.93
253	431.35	2154	5.30
254	432.35	386	0.95
255	433.30	31	0.08
256	439.20	3	0.01
257	466.10	3	0.01
258	468.40	3	0.01
259	472.40	2	0.00
260	475.40	3	0.01
261	485.20	2	0.00
262	486.10	2	0.00
263	489.40	3	0.01
264	504.40	3	0.01
265	516.30	2	0.00
266	519.10	2	0.00
267	527.40	2	0.00
268	570.10	6	0.01
269	585.40	3	0.01
270	611.40	3	0.01
271	612.40	3	0.01
272	618.10	2	0.00
273	631.40	3	0.01
274	645.40	6	0.01
275	667.30	3	0.01
276	671.10	3	0.01
277	681.20	3	0.01
278	683.40	3	0.01

Line#:15 R.Time:33.7(Scan#:8916)
MassPeaks:367

DEPTT. OF BOTANICAL & ENVIRONMENTAL SCIENCES, G.N.D.U. AMRITSAR

RawMode:Averaged 33.4-33.8(8826-8939) BasePeak:137(120935)
BG Mode:None Group 1 - Event 1

#	m/z	Abs. Int.	Rel. Int.
1	50.15	657	0.54
2	51.15	1124	0.93
3	52.15	711	0.59
4	53.10	7970	6.59
5	54.15	2570	2.13
6	55.15	61608	50.94
7	56.15	5960	4.93
8	57.10	21165	17.50
9	58.10	1122	0.93
10	59.10	1061	0.88
11	60.10	425	0.35
12	61.10	329	0.27
13	62.10	151	0.12
14	63.10	495	0.41
15	64.10	322	0.27
16	65.10	3642	3.01
17	66.15	1611	1.33
18	67.10	46292	38.28
19	68.10	9273	7.67
20	69.10	71482	59.11
21	70.10	6027	4.98
22	71.10	7772	6.43
23	72.10	834	0.69
24	73.10	1715	1.42
25	74.10	431	0.36
26	75.10	465	0.38
27	76.10	327	0.27
28	77.10	8861	7.33
29	78.15	1929	1.60
30	79.10	25780	21.32
31	80.10	4272	3.53
32	81.10	50642	41.88
33	82.10	13107	10.84
34	83.10	23385	19.34
35	84.15	3204	2.65
36	85.10	3634	3.00
37	86.10	427	0.35
38	87.10	525	0.43
39	88.10	415	0.34
40	89.10	426	0.35
41	90.10	139	0.11
42	91.10	20901	17.28
43	92.10	3494	2.89
44	93.10	31776	26.28
45	94.10	7158	5.92
46	95.10	67691	55.97
47	96.15	13131	10.86
48	97.15	12657	10.47
49	98.10	1534	1.27
50	99.10	1219	1.01
51	100.05	357	0.30
52	101.15	459	0.38
53	102.10	377	0.31
54	103.10	1415	1.17
55	104.10	723	0.60
56	105.10	20793	17.19
57	106.15	4658	3.85
58	107.15	34830	28.80
59	108.10	12867	10.64
60	109.15	49271	40.74
61	110.15	8692	7.19
62	111.15	13279	10.98
63	112.15	1536	1.27
64	113.15	1629	1.35
65	114.15	458	0.38
66	115.10	1995	1.65
67	116.10	947	0.78
68	117.10	4466	3.69
69	118.10	1392	1.15
70	119.15	19028	15.73
71	120.15	5751	4.76
72	121.15	29719	24.57
73	122.15	8363	6.92
74	123.15	33339	27.57
75	124.15	6202	5.13
76	125.20	7526	6.22
77	126.15	1610	1.33
78	127.15	945	0.78

#	m/z	Abs. Int.	Rel. Int.
79	128.10	1525	1.26
80	129.15	2811	2.32
81	130.10	1146	0.95
82	131.10	6585	5.45
83	132.15	1902	1.57
84	133.10	13173	10.89
85	134.15	5205	4.30
86	135.15	18281	15.12
87	136.15	11781	9.74
88	137.15	120935	100.00
89	138.15	19291	15.95
90	139.15	5532	4.57
91	140.15	1227	1.01
92	141.10	1133	0.94
93	142.10	1410	1.17
94	143.10	2533	2.09
95	144.15	1114	0.92
96	145.15	7314	6.05
97	146.15	2068	1.71
98	147.15	11321	9.36
99	148.15	4069	3.36
100	149.15	15800	13.06
101	150.15	9215	7.62
102	151.20	9700	8.02
103	152.15	4116	3.40
104	153.20	2781	2.30
105	154.10	587	0.49
106	155.10	859	0.71
107	156.15	1080	0.89
108	157.15	2306	1.91
109	158.10	870	0.72
110	159.15	5604	4.63
111	160.20	1532	1.27
112	161.15	8013	6.63
113	162.15	2856	2.36
114	163.20	12297	10.17
115	164.20	3480	2.88
116	165.20	3237	2.68
117	166.20	914	0.76
118	167.15	641	0.53
119	168.20	225	0.19
120	169.15	662	0.55
121	170.20	535	0.44
122	171.15	2018	1.67
123	172.15	748	0.62
124	173.15	4673	3.86
125	174.20	1425	1.18
126	175.20	6428	5.32
127	176.20	2489	2.06
128	177.20	7413	6.13
129	178.15	1953	1.61
130	179.15	4421	3.66
131	180.15	804	0.66
132	181.20	452	0.37
133	182.20	164	0.14
134	183.15	512	0.42
135	184.15	292	0.24
136	185.15	1830	1.51
137	186.15	631	0.52
138	187.15	3683	3.05
139	188.15	1547	1.28
140	189.20	6235	5.16
141	190.15	2379	1.97
142	191.20	11733	9.70
143	192.20	2914	2.41
144	193.15	2103	1.74
145	194.15	421	0.35
146	195.20	273	0.23
147	196.10	107	0.09
148	197.20	427	0.35
149	198.15	383	0.32
150	199.15	1599	1.32
151	200.15	510	0.42
152	201.15	2347	1.94
153	202.15	1260	1.04
154	203.15	5215	4.31
155	204.20	3222	2.66
156	205.20	14092	11.65

#	m/z	Abs. Int.	Rel. Int.
157	206.20	4987	4.12
158	207.15	4209	3.48
159	208.15	921	0.76
160	209.10	398	0.33
161	210.20	99	0.08
162	211.10	317	0.26
163	212.10	146	0.12
164	213.15	1110	0.92
165	214.15	504	0.42
166	215.15	2401	1.99
167	216.15	874	0.72
168	217.15	3595	2.97
169	218.20	3863	3.19
170	219.15	4524	3.74
171	220.15	1567	1.30
172	221.15	795	0.66
173	222.20	215	0.18
174	223.20	162	0.13
175	224.20	21	0.02
176	225.20	233	0.19
177	226.20	98	0.08
178	227.15	937	0.77
179	228.15	313	0.26
180	229.15	1960	1.62
181	230.20	615	0.51
182	231.15	2614	2.16
183	232.15	985	0.81
184	233.15	924	0.76
185	234.20	255	0.21
186	235.20	191	0.16
187	236.10	17	0.01
188	237.10	82	0.07
189	238.20	6	0.00
190	239.20	205	0.17
191	240.20	92	0.08
192	241.20	923	0.76
193	242.20	282	0.23
194	243.15	1073	0.89
195	244.15	516	0.43
196	245.20	2303	1.90
197	246.20	744	0.62
198	247.20	1105	0.91
199	248.20	355	0.29
200	249.20	227	0.19
201	250.20	28	0.02
202	251.20	84	0.07
203	252.20	4	0.00
204	253.20	280	0.23
205	254.20	84	0.07
206	255.15	1659	1.37
207	256.20	489	0.40
208	257.20	1062	0.88
209	258.15	421	0.35
210	259.15	4674	3.86
211	260.20	1226	1.01
212	261.20	433	0.36
213	262.20	161	0.13
214	263.20	50	0.04
215	264.20	3	0.00
216	265.20	151	0.12
217	266.20	7	0.01
218	267.20	252	0.21
219	268.20	48	0.04
220	269.20	700	0.58
221	270.20	266	0.22
222	271.20	972	0.80
223	272.25	514	0.43
224	273.20	2772	2.29
225	274.25	1919	1.59
226	275.25	989	0.82
227	276.25	354	0.29
228	277.20	55	0.05
229	278.20	5	0.00
230	279.30	3	0.00
231	280.20	2	0.00
232	281.05	609	0.50
233	282.20	144	0.12
234	283.20	369	0.31

DEPTT. OF BOTANICAL & ENVIRONMENTAL SCIENCES, G.N.D.U. AMRITSAR

#	m/z	Abs. Int.	Rel. Int.
235	284.10	180	0.15
236	285.20	602	0.50
237	286.25	344	0.28
238	287.20	1106	0.91
239	288.20	446	0.37
240	289.25	310	0.26
241	290.30	48	0.04
242	291.20	4	0.00
243	292.20	1	0.00
244	293.20	1	0.00
245	295.20	169	0.14
246	296.20	17	0.01
247	297.20	256	0.21
248	298.20	87	0.07
249	299.25	444	0.37
250	300.30	238	0.20
251	301.20	456	0.38
252	302.30	222	0.18
253	303.20	93	0.08
254	304.30	3	0.00
255	307.20	1	0.00
256	309.30	103	0.09
257	310.20	12	0.01
258	311.25	285	0.24
259	312.30	124	0.10
260	313.30	1500	1.24
261	314.25	447	0.37
262	315.30	221	0.18
263	316.30	38	0.03
264	317.30	1	0.00
265	318.00	1	0.00
266	319.10	1	0.00
267	323.30	27	0.02
268	324.30	8	0.01
269	325.25	516	0.43
270	326.30	290	0.24
271	327.25	519	0.43
272	328.20	168	0.14
273	329.25	466	0.39
274	330.30	126	0.10
275	334.10	3	0.00
276	335.20	1	0.00
277	336.30	3	0.00
278	337.20	335	0.28
279	338.20	86	0.07
280	339.20	128	0.11
281	340.20	50	0.04
282	341.20	290	0.24
283	342.30	174	0.14
284	343.30	2974	2.46
285	344.30	2691	2.23
286	345.30	630	0.52
287	346.30	86	0.07
288	347.30	2	0.00
289	351.30	22	0.02
290	352.30	19	0.02
291	353.30	103	0.09
292	354.20	96	0.08
293	355.25	1068	0.88
294	356.30	307	0.25
295	357.30	102	0.08
296	358.30	5	0.00
297	363.30	3	0.00
298	365.30	83	0.07
299	366.30	9	0.01
300	367.30	75	0.06
301	368.30	33	0.03
302	369.25	746	0.62
303	370.30	564	0.47
304	371.30	154	0.13
305	372.40	4	0.00
306	374.10	1	0.00
307	377.30	3	0.00
308	379.30	3	0.00
309	381.30	16	0.01
310	382.30	7	0.01
311	383.35	447	0.37
312	384.30	122	0.10
313	385.30	5	0.00
314	387.30	3	0.00
315	391.30	110	0.09
316	392.30	9	0.01
317	393.30	359	0.30
318	394.30	114	0.09
319	395.30	128	0.11
320	396.30	22	0.02
321	397.30	114	0.09
322	398.40	37	0.03
323	399.30	1	0.00
324	404.30	4	0.00
325	406.30	11	0.01
326	408.30	142	0.12
327	409.40	230	0.19
328	410.30	89	0.07
329	411.30	27028	22.35
330	412.30	8360	6.91
331	413.30	1343	1.11
332	414.30	142	0.12
333	417.30	1	0.00
334	420.20	1	0.00
335	422.30	3	0.00
336	424.35	560	0.46
337	425.30	195	0.16
338	426.30	4370	3.61
339	427.35	1480	1.22
340	428.40	277	0.23
341	429.30	6	0.00
342	430.30	3	0.00
343	453.40	1	0.00
344	454.10	1	0.00
345	469.40	2	0.00
346	471.40	3	0.00
347	477.40	1	0.00
348	503.30	1	0.00
349	506.10	1	0.00
350	531.40	2	0.00
351	532.40	1	0.00
352	563.40	1	0.00
353	580.30	5	0.00
354	588.30	1	0.00
355	592.30	1	0.00
356	595.30	1	0.00
357	626.40	1	0.00
358	627.30	1	0.00
359	633.30	1	0.00
360	635.40	3	0.00
361	639.10	1	0.00
362	642.40	4	0.00
363	644.30	3	0.00
364	659.20	1	0.00
365	688.30	1	0.00
366	694.40	3	0.00
367	698.00	1	0.00

Line#:16 R.Time:35.2(Scan#:9357)
MassPeaks:327
RawMode:Averaged 35.0-35.3(9315-9384) BasePeak:55(23493)
BG Mode:None Group 1 - Event 1

#	m/z	Abs. Int.	Rel. Int.
1	50.00	668	2.84
2	51.15	976	4.15
3	52.10	577	2.46
4	53.15	3031	12.90
5	54.15	845	3.60
6	55.10	23493	100.00
7	56.15	2698	11.48
8	57.15	20254	86.21
9	58.15	1241	5.28
10	59.10	674	2.87
11	60.10	370	1.57
12	61.10	220	0.94
13	62.10	177	0.75
14	63.10	528	2.25
15	64.10	387	1.65
16	65.10	1939	8.25
17	66.15	759	3.23
18	67.10	13176	56.08
19	68.10	2104	8.96
20	69.10	17388	74.01
21	70.15	1956	8.33
22	71.15	9969	42.43
23	72.15	759	3.23
24	73.10	2041	8.69
25	74.10	381	1.62
26	75.10	443	1.89
27	76.10	413	1.76
28	77.10	5273	22.44
29	78.15	1603	6.82
30	79.10	13671	58.19
31	80.15	2154	9.17
32	81.15	19854	84.51
33	82.15	2753	11.72
34	83.10	7694	32.75
35	84.15	1246	5.30
36	85.15	7287	31.02
37	86.10	607	2.58
38	87.10	354	1.51
39	88.10	83	0.35
40	89.20	403	1.72
41	90.10	78	0.33
42	91.10	15330	65.25
43	92.10	3221	13.71
44	93.10	14475	61.61
45	94.15	3968	16.89
46	95.15	23457	99.85
47	96.15	3340	14.22
48	97.15	6228	26.51
49	98.15	911	3.88
50	99.15	1694	7.21
51	100.10	217	0.92
52	101.20	226	0.96
53	102.10	267	1.14
54	103.10	1288	5.48
55	104.10	945	4.02
56	105.10	17579	74.83
57	106.15	4042	17.21
58	107.10	16266	69.24
59	108.15	4703	20.02
60	109.15	16974	72.25
61	110.15	2522	10.74
62	111.15	4549	19.36
63	112.20	583	2.48
64	113.20	697	2.97
65	114.10	189	0.80
66	115.10	2308	9.82
67	116.10	969	4.12
68	117.10	5661	24.10
69	118.10	2433	10.36
70	119.15	12771	54.36
71	120.15	5909	25.15
72	121.15	10264	43.69
73	122.15	3245	13.81
74	123.15	6495	27.65
75	124.15	2354	10.02
76	125.15	2300	9.79
77	126.20	425	1.81
78	127.10	848	3.61
79	128.10	2055	8.75
80	129.10	3751	15.97
81	130.15	1561	6.64
82	131.15	7825	33.31
83	132.15	2351	10.01
84	133.15	10782	45.89
85	134.15	3928	16.72
86	135.15	8683	36.96
87	136.15	2897	12.33

DEPTT. OF BOTANICAL & ENVIRONMENTAL SCIENCES, G.N.D.U. AMRITSAR

#	m/z	Abs. Int.	Rel. Int.
88	137.15	2743	11.68
89	138.15	957	4.07
90	139.15	991	4.22
91	140.10	173	0.74
92	141.10	1282	5.46
93	142.15	1141	4.86
94	143.10	5392	22.95
95	144.15	2007	8.54
96	145.15	10858	46.22
97	146.15	2913	12.40
98	147.15	8096	34.46
99	148.15	2893	12.31
100	149.20	5303	22.57
101	150.20	1172	4.99
102	151.20	1760	7.49
103	152.20	567	2.41
104	153.15	622	2.65
105	154.20	346	1.47
106	155.15	981	4.18
107	156.10	648	2.76
108	157.20	3345	14.24
109	158.15	2577	10.97
110	159.15	6903	29.38
111	160.15	3945	16.79
112	161.20	7036	29.95
113	162.20	2052	8.73
114	163.20	6107	25.99
115	164.20	1166	4.96
116	165.15	1269	5.40
117	166.20	404	1.72
118	167.20	433	1.84
119	168.20	230	0.98
120	169.15	793	3.38
121	170.20	371	1.58
122	171.20	2928	12.46
123	172.25	1125	4.79
124	173.20	4023	17.12
125	174.20	1630	6.94
126	175.20	2892	12.31
127	176.25	1130	4.81
128	177.20	2278	9.70
129	178.15	2033	8.65
130	179.20	1004	4.27
131	180.20	262	1.12
132	181.20	305	1.30
133	182.20	137	0.58
134	183.20	680	2.89
135	184.25	328	1.40
136	185.20	2460	10.47
137	186.20	1049	4.47
138	187.20	2832	12.05
139	188.20	950	4.04
140	189.20	5639	24.00
141	190.20	1776	7.56
142	191.20	3840	16.35
143	192.15	821	3.49
144	193.15	622	2.65
145	194.20	140	0.60
146	195.20	213	0.91
147	196.20	32	0.14
148	197.10	740	3.15
149	198.10	322	1.37
150	199.15	2872	12.22
151	200.15	1012	4.31
152	201.15	1736	7.39
153	202.20	720	3.06
154	203.20	3555	15.13
155	204.20	2479	10.55
156	205.20	4534	19.30
157	206.15	1845	7.85
158	207.10	3796	16.16
159	208.20	890	3.79
160	209.10	422	1.80
161	210.20	75	0.32
162	211.20	553	2.35
163	212.20	287	1.22
164	213.15	5876	25.01
165	214.15	1626	6.92
166	215.15	1654	7.04
167	216.20	547	2.33

#	m/z	Abs. Int.	Rel. Int.
168	217.20	914	3.89
169	218.25	3600	15.32
170	219.20	1525	6.49
171	220.20	340	1.45
172	221.20	571	2.43
173	222.20	171	0.73
174	223.20	149	0.63
175	224.20	9	0.04
176	225.20	296	1.26
177	226.20	137	0.58
178	227.15	1198	5.10
179	228.20	1209	5.15
180	229.15	2359	10.04
181	230.20	623	2.65
182	231.20	2924	12.45
183	232.15	847	3.61
184	233.20	535	2.28
185	234.25	487	2.07
186	235.20	255	1.09
187	236.20	45	0.19
188	237.10	25	0.11
189	238.10	9	0.04
190	239.20	544	2.32
191	240.20	179	0.76
192	241.15	879	3.74
193	242.20	280	1.19
194	243.20	378	1.61
195	244.10	261	1.11
196	245.20	509	2.17
197	246.20	443	1.89
198	247.20	519	2.21
199	248.20	263	1.12
200	249.20	165	0.70
201	250.10	13	0.06
202	251.10	79	0.34
203	253.20	394	1.68
204	254.20	206	0.88
205	255.20	3132	13.33
206	256.20	703	2.99
207	257.15	761	3.24
208	258.20	163	0.69
209	259.25	398	1.69
210	260.20	184	0.78
211	261.20	219	0.93
212	262.20	16	0.07
213	265.20	108	0.46
214	266.10	2	0.01
215	267.20	285	1.21
216	268.20	94	0.40
217	269.20	349	1.49
218	270.20	119	0.51
219	271.20	419	1.78
220	272.30	236	1.00
221	273.20	1624	6.91
222	274.20	537	2.29
223	275.30	795	3.38
224	276.30	214	0.91
225	277.20	9	0.04
226	278.20	3	0.01
227	281.05	771	3.28
228	282.20	238	1.01
229	283.25	469	2.00
230	284.30	96	0.41
231	285.20	108	0.46
232	286.20	46	0.20
233	287.10	208	0.89
234	288.30	324	1.38
235	289.30	203	0.86
236	290.30	98	0.42
237	291.10	5	0.02
238	295.00	10	0.04
239	296.10	11	0.05
240	297.10	65	0.28
241	298.30	3	0.01
242	299.10	72	0.31
243	300.20	6	0.03
244	301.10	151	0.64
245	302.25	603	2.57
246	303.30	1712	7.29
247	304.25	398	1.69

#	m/z	Abs. Int.	Rel. Int.
248	305.30	29	0.12
249	306.30	3	0.01
250	309.20	6	0.03
251	311.30	33	0.14
252	313.30	35	0.15
253	314.30	62	0.26
254	315.30	57	0.24
255	316.30	3	0.01
256	325.30	37	0.16
257	326.10	8	0.03
258	327.30	75	0.32
259	328.30	186	0.79
260	329.30	2411	10.26
261	330.35	677	2.88
262	331.40	98	0.42
263	339.30	88	0.37
264	341.30	194	0.83
265	342.30	158	0.67
266	343.30	51	0.22
267	344.20	9	0.04
268	345.30	6	0.03
269	351.30	6	0.03
270	352.40	12	0.05
271	353.40	51	0.22
272	354.35	400	1.70
273	355.20	342	1.46
274	356.30	119	0.51
275	357.40	154	0.66
276	358.30	12	0.05
277	367.30	82	0.35
278	368.30	61	0.26
279	369.30	24	0.10
280	370.30	40	0.17
281	371.30	67	0.29
282	372.40	3	0.01
283	379.40	3	0.01
284	380.40	5	0.02
285	381.35	1341	5.71
286	382.35	416	1.77
287	383.30	56	0.24
288	384.40	3	0.01
289	385.30	28	0.12
290	386.30	6	0.03
291	393.40	12	0.05
292	394.30	11	0.05
293	395.30	20	0.09
294	396.35	1418	6.04
295	397.35	467	1.99
296	398.40	81	0.34
297	399.35	906	3.86
298	400.40	259	1.10
299	401.40	28	0.12
300	406.20	2	0.01
301	408.30	60	0.26
302	409.30	12	0.05
303	410.30	38	0.16
304	411.40	217	0.92
305	412.40	195	0.83
306	413.30	62	0.26
307	414.35	1524	6.49
308	415.35	514	2.19
309	416.40	78	0.33
310	424.40	36	0.15
311	425.40	3	0.01
312	426.40	265	1.13
313	427.40	75	0.32
314	430.30	2	0.01
315	434.20	3	0.01
316	447.40	3	0.01
317	453.20	3	0.01
318	455.40	6	0.03
319	503.40	3	0.01
320	556.20	2	0.01
321	558.40	2	0.01
322	563.20	3	0.01
323	645.40	3	0.01
324	653.40	2	0.01
325	663.40	2	0.01
326	665.30	3	0.01
327	670.20	2	0.01

DEPTT. OF BOTANICAL & ENVIRONMENTAL SCIENCES, G.N.D.U. AMRITSAR

Line#:17   R.Time:35.4(Scan#:9434)
MassPeaks:355
RawMode:Averaged 35.3-35.5(9380-9459) BasePeak:218(172388)
BG Mode:None Group 1 - Event 1

#	m/z	Abs. Int.	Rel. Int.
1	50.05	735	0.43
2	51.10	1448	0.84
3	52.15	796	0.46
4	53.10	8609	4.99
5	54.15	1650	0.96
6	55.10	64397	37.36
7	56.15	5668	3.29
8	57.10	32469	18.83
9	58.05	2554	1.48
10	59.10	2270	1.32
11	60.10	461	0.27
12	61.10	296	0.17
13	62.10	264	0.15
14	63.10	639	0.37
15	64.10	442	0.26
16	65.05	4397	2.55
17	66.15	1487	0.86
18	67.10	34288	19.89
19	68.10	5346	3.10
20	69.10	66846	38.78
21	70.10	5095	2.96
22	71.10	13550	7.86
23	72.10	1481	0.86
24	73.10	2600	1.51
25	74.10	469	0.27
26	75.10	464	0.27
27	76.15	428	0.25
28	77.10	11766	6.83
29	78.10	3097	1.80
30	79.10	37775	21.91
31	80.10	10237	5.94
32	81.10	56354	32.69
33	82.10	6496	3.77
34	83.10	15167	8.80
35	84.10	2004	1.16
36	85.10	4873	2.83
37	86.10	873	0.51
38	87.10	559	0.32
39	88.20	210	0.12
40	89.10	503	0.29
41	90.15	300	0.17
42	91.10	35455	20.57
43	92.10	6561	3.81
44	93.10	46659	27.07
45	94.10	33704	19.55
46	95.10	68846	39.94
47	96.10	8131	4.72
48	97.15	10737	6.23
49	98.15	1535	0.89
50	99.10	1443	0.84
51	100.15	481	0.28
52	101.20	500	0.29
53	102.10	428	0.25
54	103.10	2605	1.51
55	104.15	1519	0.88
56	105.10	45175	26.21
57	106.10	10304	5.98
58	107.10	47272	27.42
59	108.10	19498	11.31
60	109.10	43704	25.35
61	110.15	5120	2.97
62	111.15	10573	6.13
63	112.10	1234	0.72
64	113.15	919	0.53
65	114.10	511	0.30
66	115.10	4191	2.43
67	116.10	1996	1.16
68	117.10	10044	5.83
69	118.15	3134	1.82
70	119.10	46334	26.88
71	120.15	16642	9.65
72	121.15	37183	21.57
73	122.15	20718	12.02
74	123.15	22036	12.78
75	124.20	3353	1.95

#	m/z	Abs. Int.	Rel. Int.
76	125.15	3093	1.79
77	126.15	589	0.34
78	127.15	1629	0.94
79	128.10	3616	2.10
80	129.10	5584	3.24
81	130.10	2559	1.48
82	131.15	13439	7.80
83	132.10	3822	2.22
84	133.15	33289	19.31
85	134.15	18297	10.61
86	135.15	36741	21.31
87	136.15	30002	17.40
88	137.15	19975	11.59
89	138.20	2930	1.70
90	139.15	3287	1.91
91	140.15	574	0.33
92	141.10	2417	1.40
93	142.10	2430	1.41
94	143.10	5165	3.00
95	144.10	2310	1.34
96	145.15	12459	7.23
97	146.15	4339	2.52
98	147.15	29258	16.97
99	148.15	15870	9.21
100	149.20	17784	10.32
101	150.15	5369	3.11
102	151.20	2640	1.53
103	152.20	885	0.51
104	153.15	1290	0.75
105	154.15	875	0.51
106	155.15	1708	0.99
107	156.15	1384	0.80
108	157.15	3780	2.19
109	158.15	1633	0.95
110	159.15	8811	5.11
111	160.15	2925	1.70
112	161.20	19813	11.49
113	162.15	12526	7.27
114	163.20	10912	6.33
115	164.20	1970	1.14
116	165.15	1812	1.05
117	166.15	667	0.39
118	167.10	743	0.43
119	168.15	471	0.27
120	169.15	1235	0.72
121	170.15	614	0.36
122	171.15	2601	1.51
123	172.15	926	0.54
124	173.15	5649	3.28
125	174.20	1877	1.09
126	175.20	24928	14.46
127	176.20	8145	4.72
128	177.20	6292	3.65
129	178.15	1470	0.85
130	179.15	1935	1.12
131	180.15	464	0.27
132	181.20	521	0.30
133	182.20	250	0.15
134	183.15	865	0.50
135	184.15	410	0.24
136	185.15	1765	1.02
137	186.15	743	0.43
138	187.15	5529	3.21
139	188.20	2257	1.31
140	189.15	51784	30.04
141	190.20	18131	10.52
142	191.20	9272	5.38
143	192.20	1746	1.01
144	193.10	1181	0.69
145	194.10	342	0.20
146	195.10	447	0.26
147	196.20	160	0.09
148	197.10	700	0.41
149	198.10	294	0.17
150	199.15	1471	0.85

#	m/z	Abs. Int.	Rel. Int.
151	200.15	584	0.34
152	201.15	4021	2.33
153	202.25	2288	1.33
154	203.15	134560	78.06
155	204.20	25532	14.81
156	205.20	9237	5.36
157	206.20	3764	2.18
158	207.15	15916	9.23
159	208.15	4377	2.54
160	209.15	933	0.54
161	210.10	219	0.13
162	211.05	582	0.34
163	212.10	227	0.13
164	213.10	1540	0.89
165	214.10	752	0.44
166	215.15	4183	2.43
167	216.15	1612	0.94
168	217.25	3032	1.76
169	218.20	172388	100.00
170	219.20	32204	18.68
171	220.15	3409	1.98
172	221.20	928	0.54
173	222.10	230	0.13
174	223.10	275	0.16
175	224.10	42	0.02
176	225.10	403	0.23
177	226.10	184	0.11
178	227.15	1074	0.62
179	228.15	499	0.29
180	229.15	3682	2.14
181	230.20	1162	0.67
182	231.15	2585	1.50
183	232.15	1161	0.67
184	233.15	902	0.52
185	234.20	796	0.46
186	235.20	235	0.14
187	236.20	19	0.01
188	237.10	140	0.08
189	238.10	40	0.02
190	239.20	468	0.27
191	240.10	142	0.08
192	241.15	948	0.55
193	242.15	362	0.21
194	243.20	2871	1.67
195	244.20	992	0.58
196	245.20	1008	0.58
197	246.10	362	0.21
198	247.20	189	0.11
199	248.20	128	0.07
200	249.10	210	0.12
201	250.10	11	0.01
202	251.10	146	0.08
203	252.10	29	0.02
204	253.10	376	0.22
205	254.10	155	0.09
206	255.15	1886	1.09
207	256.15	588	0.34
208	257.20	5694	3.30
209	258.20	1644	0.95
210	259.20	976	0.57
211	260.20	202	0.12
212	261.20	81	0.05
213	263.20	2	0.00
214	264.20	2	0.00
215	265.20	249	0.14
216	266.20	27	0.02
217	267.20	422	0.24
218	268.20	130	0.08
219	269.20	1037	0.60
220	270.20	616	0.36
221	271.20	1414	0.82
222	272.20	1353	0.78
223	273.25	603	0.35
224	274.20	227	0.13
225	275.20	139	0.08

DEPTT. OF BOTANICAL & ENVIRONMENTAL SCIENCES, G.N.D.U. AMRITSAR

#	m/z	Abs. Int.	Rel. Int.	#	m/z	Abs. Int.	Rel. Int.	#	m/z	Abs. Int.	Rel. Int.
226	276.20	5	0.00	270	326.20	28	0.02	314	399.30	6	0.00
227	277.20	5	0.00	271	327.30	36	0.02	315	401.30	22	0.01
228	278.30	2	0.00	272	328.20	2	0.00	316	404.30	5	0.00
229	279.20	28	0.02	273	329.20	111	0.06	317	405.30	2	0.00
230	280.10	5	0.00	274	330.20	3	0.00	318	406.40	8	0.00
231	281.10	1180	0.68	275	332.20	2	0.00	319	408.30	441	0.26
232	282.10	401	0.23	276	334.20	3	0.00	320	409.40	231	0.13
233	283.20	564	0.33	277	337.20	242	0.14	321	410.30	140	0.08
234	284.10	289	0.17	278	338.20	63	0.04	322	411.30	1563	0.91
235	285.20	420	0.24	279	339.30	131	0.08	323	412.30	513	0.30
236	286.20	432	0.25	280	340.30	24	0.01	324	413.30	51	0.03
237	287.20	140	0.08	281	341.20	111	0.06	325	414.30	24	0.01
238	288.20	141	0.08	282	342.10	16	0.01	326	415.20	13	0.01
239	289.20	69	0.04	283	351.30	21	0.01	327	416.20	5	0.00
240	290.20	12	0.01	284	352.20	8	0.00	328	417.30	2	0.00
241	291.10	7	0.00	285	353.10	38	0.02	329	424.30	167	0.10
242	293.10	5	0.00	286	354.20	20	0.01	330	425.30	78	0.05
243	294.10	2	0.00	287	355.20	204	0.12	331	426.30	2242	1.30
244	295.20	126	0.07	288	356.20	29	0.02	332	427.35	787	0.46
245	296.30	36	0.02	289	357.00	20	0.01	333	428.40	119	0.07
246	297.20	292	0.17	290	358.30	2	0.00	334	429.40	5	0.00
247	298.30	96	0.06	291	361.30	2	0.00	335	430.20	7	0.00
248	299.20	171	0.10	292	363.20	5	0.00	336	431.30	2	0.00
249	300.00	46	0.03	293	365.30	264	0.15	337	444.40	2	0.00
250	301.10	61	0.04	294	366.30	36	0.02	338	445.40	5	0.00
251	302.10	13	0.01	295	367.20	5	0.00	339	450.30	2	0.00
252	303.20	40	0.02	296	368.20	32	0.02	340	474.40	2	0.00
253	304.30	5	0.00	297	369.30	46	0.03	341	475.30	2	0.00
254	307.10	11	0.01	298	370.10	5	0.00	342	494.40	2	0.00
255	308.20	2	0.00	299	371.10	5	0.00	343	498.30	2	0.00
256	309.20	34	0.02	300	379.30	2	0.00	344	548.40	2	0.00
257	310.20	13	0.01	301	380.10	2	0.00	345	555.30	2	0.00
258	311.30	172	0.10	302	381.20	24	0.01	346	566.20	2	0.00
259	312.30	38	0.02	303	382.20	5	0.00	347	580.30	6	0.00
260	313.20	50	0.03	304	383.20	27	0.02	348	614.20	2	0.00
261	314.30	98	0.06	305	386.20	5	0.00	349	620.10	2	0.00
262	315.20	54	0.03	306	390.00	5	0.00	350	635.30	2	0.00
263	316.30	2	0.00	307	391.20	14	0.01	351	645.40	5	0.00
264	319.20	2	0.00	308	392.30	5	0.00	352	654.30	2	0.00
265	321.20	5	0.00	309	393.30	899	0.52	353	668.20	2	0.00
266	322.20	5	0.00	310	394.35	332	0.19	354	677.30	2	0.00
267	323.30	92	0.05	311	395.40	45	0.03	355	687.30	2	0.00
268	324.20	7	0.00	312	396.20	84	0.05				
269	325.30	109	0.06	313	397.30	39	0.02				

Line#:18 R.Time:35.6(Scan#:9485)
MassPeaks:283
RawMode:Averaged 35.5-35.7(9459-9505) BasePeak:55(16679)
BG Mode:None Group 1 - Event 1

#	m/z	Abs. Int.	Rel. Int.	#	m/z	Abs. Int.	Rel. Int.	#	m/z	Abs. Int.	Rel. Int.
1	50.00	642	3.85	31	80.10	2146	12.87	61	110.15	1587	9.51
2	51.05	1037	6.22	32	81.15	13871	83.16	62	111.15	2074	12.43
3	52.10	587	3.52	33	82.10	2262	13.56	63	112.10	391	2.34
4	53.15	2792	16.74	34	83.10	4623	27.72	64	113.20	428	2.57
5	54.15	757	4.54	35	84.15	945	5.67	65	114.20	124	0.74
6	55.15	16679	100.00	36	85.15	1952	11.70	66	115.10	1957	11.73
7	56.15	2127	12.75	37	86.10	275	1.65	67	116.10	824	4.94
8	57.15	7008	42.02	38	87.10	361	2.16	68	117.10	3413	20.46
9	58.10	681	4.08	39	88.10	80	0.48	69	118.15	1204	7.22
10	59.05	757	4.54	40	89.10	473	2.84	70	119.10	12710	76.20
11	60.10	415	2.49	41	90.10	83	0.50	71	120.15	5661	33.94
12	61.10	272	1.63	42	91.10	11127	66.71	72	121.15	9049	54.25
13	62.10	204	1.22	43	92.10	2149	12.88	73	122.15	9541	57.20
14	63.10	575	3.45	44	93.10	11608	69.60	74	123.20	6317	37.87
15	64.10	391	2.34	45	94.15	4634	27.78	75	124.20	1080	6.48
16	65.10	1745	10.46	46	95.15	15612	93.60	76	125.20	1159	6.95
17	66.15	633	3.80	47	96.15	2782	16.68	77	126.20	318	1.91
18	67.10	9633	57.76	48	97.15	3173	19.02	78	127.20	653	3.92
19	68.10	1796	10.77	49	98.10	663	3.98	79	128.10	1461	8.76
20	69.10	13619	81.65	50	99.10	701	4.20	80	129.15	2030	12.17
21	70.15	1687	10.11	51	100.10	198	1.19	81	130.10	890	5.34
22	71.10	3487	20.91	52	101.10	203	1.22	82	131.15	4094	24.55
23	72.10	431	2.58	53	102.20	250	1.50	83	132.15	1586	9.51
24	73.10	2542	15.24	54	103.10	1145	6.86	84	133.15	9419	56.47
25	74.10	447	2.68	55	104.15	612	3.67	85	134.15	8760	52.52
26	75.10	552	3.31	56	105.15	12416	74.44	86	135.15	9026	54.12
27	76.10	364	2.18	57	106.10	3608	21.63	87	136.15	5473	32.81
28	77.10	4605	27.61	58	107.15	11617	69.65	88	137.20	2650	15.89
29	78.10	1271	7.62	59	108.15	6079	36.45	89	138.20	491	2.94
30	79.10	9806	58.79	60	109.15	10371	62.18	90	139.20	615	3.69

DEPTT. OF BOTANICAL & ENVIRONMENTAL SCIENCES, G.N.D.U. AMRITSAR

#	m/z	Abs. Int.	Rel. Int.
91	140.20	147	0.88
92	141.05	875	5.25
93	142.10	815	4.89
94	143.15	1732	10.38
95	144.15	751	4.50
96	145.15	3572	21.42
97	146.20	1371	8.22
98	147.15	7394	44.33
99	148.20	4483	26.88
100	149.20	4377	26.24
101	150.20	1140	6.83
102	151.20	777	4.66
103	152.20	370	2.22
104	153.20	523	3.14
105	154.20	303	1.82
106	155.20	671	4.02
107	156.20	441	2.64
108	157.15	1196	7.17
109	158.15	575	3.45
110	159.20	2360	14.15
111	160.15	906	5.43
112	161.20	4899	29.37
113	162.20	2855	17.12
114	163.20	2434	14.59
115	164.20	528	3.17
116	165.20	727	4.36
117	166.20	275	1.65
118	167.20	321	1.92
119	168.10	172	1.03
120	169.20	524	3.14
121	170.20	238	1.43
122	171.20	836	5.01
123	172.20	339	2.03
124	173.20	1647	9.87
125	174.20	653	3.92
126	175.20	5378	32.24
127	176.20	1896	11.37
128	177.20	2089	12.52
129	178.20	578	3.47
130	179.20	586	3.51
131	180.20	138	0.83
132	181.20	216	1.30
133	182.20	54	0.32
134	183.20	368	2.21
135	184.20	69	0.41
136	185.15	671	4.02
137	186.20	358	2.15
138	187.20	1500	8.99
139	188.15	711	4.26
140	189.20	8236	49.38
141	190.20	2807	16.83
142	191.15	2032	12.18
143	192.20	583	3.50
144	193.10	560	3.36
145	194.20	99	0.59
146	195.20	187	1.12
147	196.10	22	0.13
148	197.20	326	1.95
149	198.20	124	0.74
150	199.20	500	3.00
151	200.20	261	1.56
152	201.15	1052	6.31
153	202.20	796	4.77
154	203.15	8118	48.67
155	204.20	8084	48.47

#	m/z	Abs. Int.	Rel. Int.
156	205.20	2766	16.58
157	206.15	702	4.21
158	207.10	3864	23.17
159	208.10	930	5.58
160	209.10	525	3.15
161	210.10	38	0.23
162	211.10	269	1.61
163	212.10	4	0.02
164	213.10	490	2.94
165	214.10	223	1.34
166	215.20	813	4.87
167	216.10	375	2.25
168	217.20	612	3.67
169	218.20	13577	81.40
170	219.20	2697	16.17
171	220.20	417	2.50
172	221.20	328	1.97
173	222.20	18	0.11
174	223.20	67	0.40
175	224.20	9	0.05
176	225.20	193	1.16
177	226.20	49	0.29
178	227.20	369	2.21
179	228.20	72	0.43
180	229.20	635	3.81
181	230.20	279	1.67
182	231.20	613	3.68
183	232.20	264	1.58
184	233.20	135	0.81
185	234.20	201	1.21
186	235.20	42	0.25
187	236.20	9	0.05
188	237.20	10	0.06
189	238.20	8	0.05
190	239.20	135	0.81
191	240.20	41	0.25
192	241.20	251	1.50
193	242.20	45	0.27
194	243.20	633	3.80
195	244.20	186	1.12
196	245.20	228	1.37
197	246.20	29	0.17
198	248.20	8	0.05
199	249.20	155	0.93
200	250.20	8	0.05
201	251.20	92	0.55
202	253.20	192	1.15
203	254.20	13	0.08
204	255.20	398	2.39
205	256.20	121	0.73
206	257.20	617	3.70
207	258.20	274	1.64
208	259.20	177	1.06
209	260.20	4	0.02
210	261.20	4	0.02
211	265.20	272	1.63
212	266.20	10	0.06
213	267.20	314	1.88
214	268.20	4	0.02
215	269.20	232	1.39
216	270.20	121	0.73
217	271.20	302	1.81
218	272.20	238	1.43
219	273.20	104	0.62
220	274.20	13	0.08

#	m/z	Abs. Int.	Rel. Int.
221	277.20	17	0.10
222	278.20	8	0.05
223	280.20	4	0.02
224	281.10	926	5.55
225	282.20	290	1.74
226	283.20	251	1.50
227	284.20	49	0.29
228	285.10	82	0.49
229	286.20	39	0.23
230	287.20	13	0.08
231	288.20	5	0.03
232	293.10	9	0.05
233	295.10	26	0.16
234	297.20	9	0.05
235	298.20	4	0.02
236	299.20	29	0.17
237	300.20	4	0.02
238	307.20	4	0.02
239	309.10	4	0.02
240	311.20	13	0.08
241	313.20	43	0.26
242	314.20	9	0.05
243	325.10	19	0.11
244	326.20	4	0.02
245	327.20	4	0.02
246	328.20	4	0.02
247	329.10	8	0.05
248	330.20	9	0.05
249	339.20	4	0.02
250	341.00	81	0.49
251	342.20	15	0.09
252	343.20	4	0.02
253	355.20	104	0.62
254	356.20	4	0.02
255	357.20	4	0.02
256	364.20	9	0.05
257	365.20	4	0.02
258	366.20	4	0.02
259	368.20	4	0.02
260	381.20	4	0.02
261	391.20	5	0.03
262	393.20	48	0.29
263	397.20	10	0.06
264	406.10	9	0.05
265	408.20	10	0.06
266	409.20	196	1.18
267	410.20	155	0.93
268	411.20	66	0.40
269	412.20	139	0.83
270	413.20	4	0.02
271	424.20	214	1.28
272	425.20	77	0.46
273	426.20	37	0.22
274	427.10	9	0.05
275	428.20	4	0.02
276	439.20	4	0.02
277	474.20	4	0.02
278	479.20	4	0.02
279	489.20	4	0.02
280	505.20	4	0.02
281	544.10	4	0.02
282	557.20	4	0.02
283	661.20	4	0.02

Line#:19 R.Time:36.3(Scan#:9699)
MassPeaks:418
RawMode:Averaged 36.0-36.4(9590-9730) BasePeak:218(224924)
BG Mode:None Group 1 - Event 1

#	m/z	Abs. Int.	Rel. Int.
1	50.05	935	0.42
2	51.10	1945	0.86
3	52.15	1192	0.53
4	53.10	18092	8.04
5	54.15	4765	2.12
6	55.10	129797	57.71
7	56.15	14840	6.60
8	57.15	66445	29.54
9	58.10	6650	2.96

#	m/z	Abs. Int.	Rel. Int.
10	59.10	8697	3.87
11	60.10	831	0.37
12	61.10	443	0.20
13	62.10	335	0.15
14	63.05	856	0.38
15	64.15	575	0.26
16	65.05	8263	3.67
17	66.15	3577	1.59
18	67.10	87213	38.77

#	m/z	Abs. Int.	Rel. Int.
19	68.10	32330	14.37
20	69.10	124349	55.28
21	70.15	13338	5.93
22	71.10	41782	18.58
23	72.10	4167	1.85
24	73.10	4484	1.99
25	74.10	762	0.34
26	75.10	718	0.32
27	76.15	624	0.28

DEPTT. OF BOTANICAL & ENVIRONMENTAL SCIENCES, G.N.D.U. AMRITSAR

#	m/z	Abs. Int.	Rel. Int.
28	77.05	23719	10.55
29	78.10	5335	2.37
30	79.10	73501	32.68
31	80.10	18853	8.38
32	81.10	130015	57.80
33	82.10	28306	12.58
34	83.10	47000	20.90
35	84.15	8136	3.62
36	85.15	17073	7.59
37	86.10	2304	1.02
38	87.10	1182	0.53
39	88.10	574	0.26
40	89.05	693	0.31
41	90.15	712	0.32
42	91.10	68725	30.55
43	92.10	13925	6.19
44	93.10	115409	51.31
45	94.10	45188	20.09
46	95.10	154281	68.59
47	96.10	27257	12.12
48	97.10	35712	15.88
49	98.15	5493	2.44
50	99.20	5673	2.52
51	100.10	1556	0.69
52	101.15	1035	0.46
53	102.10	755	0.34
54	103.05	4471	1.99
55	104.15	2516	1.12
56	105.10	86155	38.30
57	106.10	27218	12.10
58	107.10	120613	53.62
59	108.10	60894	27.07
60	109.10	114333	50.83
61	110.10	20352	9.05
62	111.10	26566	11.81
63	112.15	3952	1.76
64	113.20	3676	1.63
65	114.10	1555	0.69
66	115.10	6384	2.84
67	116.10	2965	1.32
68	117.10	16172	7.19
69	118.15	5491	2.44
70	119.10	104152	46.31
71	120.10	41121	18.28
72	121.10	101981	45.34
73	122.15	97168	43.20
74	123.15	84838	37.72
75	124.15	12362	5.50
76	125.15	12169	5.41
77	126.20	2366	1.05
78	127.15	4253	1.89
79	128.10	5453	2.42
80	129.10	8543	3.80
81	130.10	4112	1.83
82	131.10	25157	11.18
83	132.10	10747	4.78
84	133.10	87253	38.79
85	134.15	56746	25.23
86	135.15	115257	51.24
87	136.15	78457	34.88
88	137.15	33668	14.97
89	138.15	6285	2.79
90	139.10	10754	4.78
91	140.15	2311	1.03
92	141.15	4526	2.01
93	142.05	3975	1.77
94	143.10	8536	3.80
95	144.15	4016	1.79
96	145.10	27342	12.16
97	146.15	11447	5.09
98	147.15	80970	36.00
99	148.15	59627	26.51
100	149.15	59761	26.57
101	150.15	16367	7.28
102	151.20	8132	3.62
103	152.15	5716	2.54
104	153.15	4987	2.22
105	154.15	1761	0.78
106	155.10	3180	1.41
107	156.10	2291	1.02

#	m/z	Abs. Int.	Rel. Int.
108	157.15	6861	3.05
109	158.15	3074	1.37
110	159.15	21653	9.63
111	160.15	8773	3.90
112	161.15	67464	29.99
113	162.15	33967	15.10
114	163.15	30656	13.63
115	164.15	5617	2.50
116	165.15	4297	1.91
117	166.15	2102	0.93
118	167.25	1855	0.82
119	168.10	929	0.41
120	169.15	2227	0.99
121	170.10	1103	0.49
122	171.15	5425	2.41
123	172.15	2288	1.02
124	173.15	17060	7.58
125	174.15	5630	2.50
126	175.15	44420	19.75
127	176.20	14897	6.62
128	177.15	15507	6.89
129	178.15	4397	1.95
130	179.15	6175	2.75
131	180.15	1406	0.63
132	181.10	1266	0.56
133	182.20	614	0.27
134	183.15	1681	0.75
135	184.05	699	0.31
136	185.10	3833	1.70
137	186.15	2244	1.00
138	187.15	21275	9.46
139	188.15	8025	3.57
140	189.15	109603	48.73
141	190.15	41449	18.43
142	191.15	27327	12.15
143	192.15	6172	2.74
144	193.15	3866	1.72
145	194.10	1185	0.53
146	195.10	1010	0.45
147	196.10	443	0.20
148	197.10	1315	0.58
149	198.05	531	0.24
150	199.15	3149	1.40
151	200.15	1201	0.53
152	201.10	14759	6.56
153	202.10	6292	2.80
154	203.15	84430	37.54
155	204.15	31250	13.89
156	205.15	18697	8.31
157	206.15	13083	5.82
158	207.15	60870	27.06
159	208.15	16461	7.32
160	209.10	2905	1.29
161	210.15	536	0.24
162	211.15	1068	0.47
163	212.10	431	0.19
164	213.10	3083	1.37
165	214.15	1232	0.55
166	215.10	8566	3.81
167	216.10	7135	3.17
168	217.15	10955	4.87
169	218.15	224924	100.00
170	219.15	46490	20.67
171	220.15	10269	4.57
172	221.10	3756	1.67
173	222.15	1251	0.56
174	223.10	675	0.30
175	224.20	257	0.11
176	225.10	824	0.37
177	226.15	351	0.16
178	227.10	2517	1.12
179	228.10	1158	0.51
180	229.10	11051	4.91
181	230.15	3679	1.64
182	231.10	7192	3.20
183	232.15	3085	1.37
184	233.15	1969	0.88
185	234.15	6801	3.02
186	235.10	1514	0.67
187	236.10	330	0.15

#	m/z	Abs. Int.	Rel. Int.
188	237.10	388	0.17
189	238.20	198	0.09
190	239.15	783	0.35
191	240.10	352	0.16
192	241.10	2053	0.91
193	242.10	1041	0.46
194	243.15	5899	2.62
195	244.15	1986	0.88
196	245.15	3295	1.46
197	246.15	1701	0.76
198	247.15	3785	1.68
199	248.15	1701	0.76
200	249.10	669	0.30
201	250.20	246	0.11
202	251.10	397	0.18
203	252.20	158	0.07
204	253.10	706	0.31
205	254.10	284	0.13
206	255.15	3500	1.56
207	256.15	1231	0.55
208	257.10	13458	5.98
209	258.15	4038	1.80
210	259.20	7577	3.37
211	260.20	1741	0.77
212	261.15	507	0.23
213	262.20	115	0.05
214	263.20	67	0.03
215	264.20	110	0.05
216	265.10	524	0.23
217	266.10	240	0.11
218	267.00	897	0.40
219	268.10	304	0.14
220	269.15	2055	0.91
221	270.15	1646	0.73
222	271.15	3862	1.72
223	272.20	6625	2.95
224	273.20	3130	1.39
225	274.25	3515	1.56
226	275.25	1152	0.51
227	276.15	341	0.15
228	277.20	103	0.05
229	278.30	90	0.04
230	279.20	112	0.05
231	280.10	46	0.02
232	281.00	2429	1.08
233	282.10	807	0.36
234	283.15	1623	0.72
235	284.20	937	0.42
236	285.20	1412	0.63
237	286.20	3326	1.48
238	287.20	1406	0.63
239	288.20	774	0.34
240	289.20	305	0.14
241	290.20	123	0.05
242	291.20	39	0.02
243	292.20	22	0.01
244	293.20	61	0.03
245	294.20	9	0.00
246	295.20	626	0.28
247	296.20	383	0.17
248	297.20	3301	1.47
249	298.20	1499	0.67
250	299.20	2000	0.89
251	300.20	1745	0.78
252	301.20	1004	0.45
253	302.20	573	0.25
254	303.10	182	0.08
255	304.20	19	0.01
256	305.20	3	0.00
257	306.20	17	0.01
258	307.30	42	0.02
259	308.20	13	0.01
260	309.20	360	0.16
261	310.20	144	0.06
262	311.20	492	0.22
263	312.25	270	0.12
264	313.20	2137	0.95
265	314.20	1213	0.54
266	315.20	4688	2.08
267	316.20	2601	1.16

DEPTT. OF BOTANICAL & ENVIRONMENTAL SCIENCES, G.N.D.U. AMRITSAR

#	m/z	Abs. Int.	Rel. Int.
268	317.20	1244	0.55
269	318.15	304	0.14
270	319.20	65	0.03
271	320.20	6	0.00
272	321.30	17	0.01
273	322.20	12	0.01
274	323.20	501	0.22
275	324.25	262	0.12
276	325.20	756	0.34
277	326.25	440	0.20
278	327.25	414	0.18
279	328.30	162	0.07
280	329.20	254	0.11
281	330.20	126	0.06
282	331.20	49	0.02
283	332.30	4	0.00
284	333.20	4	0.00
285	334.30	7	0.00
286	335.20	14	0.01
287	336.10	10	0.00
288	337.25	612	0.27
289	338.20	245	0.11
290	339.20	726	0.32
291	340.20	398	0.18
292	341.25	707	0.31
293	342.20	716	0.32
294	343.20	745	0.33
295	344.25	789	0.35
296	345.20	200	0.09
297	346.20	23	0.01
298	347.20	1	0.00
299	349.30	7	0.00
300	350.30	10	0.00
301	351.20	288	0.13
302	352.30	163	0.07
303	353.20	181	0.08
304	354.20	120	0.05
305	355.15	604	0.27
306	356.15	345	0.15
307	357.25	698	0.31
308	358.20	333	0.15
309	359.20	71	0.03
310	361.20	1	0.00
311	362.30	6	0.00
312	363.30	28	0.01
313	364.30	18	0.01
314	365.20	1656	0.74
315	366.20	553	0.25
316	367.20	181	0.08
317	368.20	206	0.09
318	369.25	470	0.21

#	m/z	Abs. Int.	Rel. Int.
319	370.25	1155	0.51
320	371.25	368	0.16
321	372.30	61	0.03
322	373.10	7	0.00
323	374.30	4	0.00
324	375.00	1	0.00
325	376.30	5	0.00
326	377.20	17	0.01
327	379.20	123	0.05
328	380.20	28	0.01
329	381.20	79	0.04
330	382.20	49	0.02
331	383.20	1345	0.60
332	384.20	488	0.22
333	385.20	86	0.04
334	387.10	1	0.00
335	389.30	8	0.00
336	390.30	33	0.01
337	391.20	139	0.06
338	392.30	45	0.02
339	393.25	3012	1.34
340	394.25	997	0.44
341	395.30	212	0.09
342	396.30	82	0.04
343	397.30	237	0.11
344	398.30	113	0.05
345	399.30	35	0.02
346	400.30	9	0.00
347	401.20	4	0.00
348	402.20	1	0.00
349	404.30	6	0.00
350	406.30	94	0.04
351	407.30	144	0.06
352	408.30	1428	0.63
353	409.25	750	0.33
354	410.25	210	0.09
355	411.25	6494	2.89
356	412.25	2080	0.92
357	413.25	372	0.17
358	414.30	50	0.02
359	415.10	12	0.01
360	417.30	1	0.00
361	418.30	14	0.01
362	421.30	4	0.00
363	422.30	19	0.01
364	423.20	3	0.00
365	424.30	322	0.14
366	425.30	189	0.08
367	426.30	10930	4.86
368	427.30	3609	1.60
369	428.30	654	0.29

#	m/z	Abs. Int.	Rel. Int.
370	429.30	145	0.06
371	430.30	41	0.02
372	431.30	3	0.00
373	432.30	36	0.02
374	435.40	26	0.01
375	436.40	1	0.00
376	438.20	1	0.00
377	439.30	2	0.00
378	440.30	20	0.01
379	441.30	7	0.00
380	442.30	3	0.00
381	446.30	3	0.00
382	448.30	1	0.00
383	450.40	62	0.03
384	451.30	22	0.01
385	452.30	1	0.00
386	453.30	2	0.00
387	459.40	1	0.00
388	466.30	109	0.05
389	467.30	22	0.01
390	468.30	1	0.00
391	478.00	1	0.00
392	483.30	3	0.00
393	490.30	3	0.00
394	498.20	3	0.00
395	503.30	3	0.00
396	506.20	1	0.00
397	507.30	3	0.00
398	520.30	1	0.00
399	522.30	1	0.00
400	524.30	6	0.00
401	530.30	1	0.00
402	531.30	1	0.00
403	534.30	1	0.00
404	537.30	1	0.00
405	542.10	1	0.00
406	547.10	1	0.00
407	562.00	1	0.00
408	574.40	1	0.00
409	586.30	1	0.00
410	592.30	1	0.00
411	596.30	1	0.00
412	615.30	1	0.00
413	622.30	1	0.00
414	643.30	1	0.00
415	646.30	1	0.00
416	656.40	1	0.00
417	676.00	1	0.00
418	693.40	1	0.00

Line#:20 R.Time:37.2(Scan#:9963)
MassPeaks:345
RawMode:Averaged 37.1-37.3(9936-9993) BasePeak:69(34282)
BG Mode:None Group 1 - Event 1

#	m/z	Abs. Int.	Rel. Int.
1	50.00	694	2.02
2	51.10	1066	3.11
3	52.10	658	1.92
4	53.15	4109	11.99
5	54.15	1381	4.03
6	55.10	27732	80.89
7	56.10	3345	9.76
8	57.15	13706	39.98
9	58.15	942	2.75
10	59.05	999	2.91
11	60.10	753	2.20
12	61.10	581	1.69
13	62.10	229	0.67
14	63.10	549	1.60
15	64.10	421	1.23
16	65.10	2198	6.41
17	66.15	938	2.74
18	67.10	20702	60.39
19	68.15	5353	15.61
20	69.10	34282	100.00
21	70.15	3408	9.94
22	71.15	5490	16.01
23	72.10	741	2.16

#	m/z	Abs. Int.	Rel. Int.
24	73.10	6233	18.18
25	74.10	844	2.46
26	75.15	1150	3.35
27	76.10	430	1.25
28	77.05	5577	16.27
29	78.15	1462	4.26
30	79.10	14102	41.14
31	80.10	2823	8.23
32	81.10	27221	79.40
33	82.10	8632	25.18
34	83.15	13019	37.98
35	84.10	1872	5.46
36	85.10	3313	9.66
37	86.15	617	1.80
38	87.10	792	2.31
39	88.10	408	1.19
40	89.10	679	1.98
41	90.10	136	0.40
42	91.10	11894	34.69
43	92.10	2153	6.28
44	93.10	16740	48.83
45	94.15	6226	18.16
46	95.10	32213	93.96

#	m/z	Abs. Int.	Rel. Int.
47	96.15	13732	40.06
48	97.15	8325	24.28
49	98.15	1405	4.10
50	99.10	1336	3.90
51	100.10	291	0.85
52	101.10	385	1.12
53	102.10	404	1.18
54	103.10	1492	4.35
55	104.10	690	2.01
56	105.10	12678	36.98
57	106.10	3077	8.98
58	107.15	16858	49.17
59	108.15	7406	21.60
60	109.15	23764	69.32
61	110.15	5478	15.98
62	111.15	6873	20.05
63	112.15	962	2.81
64	113.15	1051	3.07
65	114.10	234	0.68
66	115.05	1947	5.68
67	116.05	808	2.36
68	117.10	3029	8.84
69	118.10	1062	3.10

DEPTT. OF BOTANICAL & ENVIRONMENTAL SCIENCES, G.N.D.U. AMRITSAR

#	m/z	Abs. Int.	Rel. Int.
70	119.10	12950	37.77
71	120.15	4443	12.96
72	121.15	14882	43.41
73	122.15	5938	17.32
74	123.15	16918	49.35
75	124.15	5385	15.71
76	125.20	9362	27.31
77	126.15	1232	3.59
78	127.15	1205	3.51
79	128.15	1276	3.72
80	129.10	1941	5.66
81	130.10	844	2.46
82	131.10	3939	11.49
83	132.15	1665	4.86
84	133.15	11068	32.29
85	134.15	5215	15.21
86	135.15	11282	32.91
87	136.15	5913	17.25
88	137.20	7395	21.57
89	138.20	2136	6.23
90	139.15	1296	3.78
91	140.10	298	0.87
92	141.10	990	2.89
93	142.15	806	2.35
94	143.15	1723	5.03
95	144.15	919	2.68
96	145.15	4503	13.14
97	146.15	1965	5.73
98	147.15	8667	25.28
99	148.20	4050	11.81
100	149.15	7219	21.06
101	150.20	2933	8.56
102	151.15	2555	7.45
103	152.15	1269	3.70
104	153.15	1073	3.13
105	154.20	426	1.24
106	155.10	778	2.27
107	156.15	493	1.44
108	157.15	1539	4.49
109	158.15	712	2.08
110	159.20	3722	10.86
111	160.20	2106	6.14
112	161.20	7237	21.11
113	162.20	2806	8.19
114	163.20	7168	20.91
115	164.20	1995	5.82
116	165.20	1815	5.29
117	166.20	464	1.35
118	167.20	448	1.31
119	168.10	158	0.46
120	169.20	607	1.77
121	170.20	293	0.85
122	171.20	1260	3.68
123	172.20	602	1.76
124	173.20	2782	8.12
125	174.20	1057	3.08
126	175.20	5485	16.00
127	176.20	2169	6.33
128	177.20	3218	9.39
129	178.20	960	2.80
130	179.20	3260	9.51
131	180.20	679	1.98
132	181.20	347	1.01
133	182.20	152	0.44
134	183.20	503	1.47
135	184.20	159	0.46
136	185.15	1329	3.88
137	186.15	543	1.58
138	187.20	2691	7.85
139	188.20	946	2.76
140	189.20	8428	24.58
141	190.15	2840	8.28
142	191.15	6011	17.53
143	192.15	1422	4.15
144	193.15	1821	5.31
145	194.20	507	1.48
146	195.20	424	1.24
147	196.20	131	0.38
148	197.10	404	1.18
149	198.10	157	0.46

#	m/z	Abs. Int.	Rel. Int.
150	199.15	1026	2.99
151	200.15	428	1.25
152	201.15	2008	5.86
153	202.20	1047	3.05
154	203.15	13192	38.48
155	204.15	4370	12.75
156	205.15	4919	14.35
157	206.15	1679	4.90
158	207.05	9431	27.51
159	208.05	2124	6.20
160	209.05	1267	3.70
161	210.00	286	0.83
162	211.10	406	1.18
163	212.00	138	0.40
164	213.15	866	2.53
165	214.15	444	1.30
166	215.15	2185	6.37
167	216.15	910	2.65
168	217.20	2231	6.51
169	218.20	15975	46.60
170	219.20	3678	10.73
171	220.20	1299	3.79
172	221.15	937	2.73
173	222.20	268	0.78
174	223.10	314	0.92
175	224.10	83	0.24
176	225.20	293	0.85
177	226.10	91	0.27
178	227.10	640	1.87
179	228.20	262	0.76
180	229.15	2483	7.24
181	230.20	808	2.36
182	231.15	2764	8.06
183	232.20	1715	5.00
184	233.20	1051	3.07
185	234.20	740	2.16
186	235.20	361	1.05
187	236.20	35	0.10
188	237.20	79	0.23
189	238.10	10	0.03
190	239.10	285	0.83
191	240.20	33	0.10
192	241.20	1039	3.03
193	242.20	259	0.76
194	243.20	751	2.19
195	244.15	351	1.02
196	245.20	1156	3.37
197	246.15	1997	5.83
198	247.15	1721	5.02
199	248.15	909	2.65
200	249.10	811	2.37
201	250.00	201	0.59
202	251.10	369	1.08
203	252.20	31	0.09
204	253.10	285	0.83
205	254.00	81	0.24
206	255.20	1199	3.50
207	256.20	350	1.02
208	257.20	1399	4.08
209	258.20	405	1.18
210	259.20	1411	4.12
211	260.20	375	1.09
212	261.20	337	0.98
213	262.20	77	0.22
214	263.20	23	0.07
215	264.20	7	0.02
216	265.20	674	1.97
217	266.20	236	0.69
218	267.00	733	2.14
219	268.00	157	0.46
220	269.20	646	1.88
221	270.25	400	1.17
222	271.20	608	1.77
223	272.25	328	0.96
224	273.20	1869	5.45
225	274.20	1031	3.01
226	275.25	404	1.18
227	276.20	118	0.34
228	277.30	11	0.03
229	278.20	11	0.03

#	m/z	Abs. Int.	Rel. Int.
230	279.20	7	0.02
231	280.00	11	0.03
232	281.05	2969	8.66
233	282.00	838	2.44
234	283.10	735	2.14
235	284.10	236	0.69
236	285.10	309	0.90
237	286.10	132	0.39
238	287.20	473	1.38
239	288.20	221	0.64
240	289.00	75	0.22
241	290.00	7	0.02
242	291.10	7	0.02
243	293.10	18	0.05
244	295.20	130	0.38
245	296.00	32	0.09
246	297.20	137	0.40
247	298.20	23	0.07
248	299.20	78	0.23
249	300.30	47	0.14
250	301.20	93	0.27
251	302.25	565	1.65
252	303.25	232	0.68
253	304.20	33	0.10
254	307.20	3	0.01
255	308.20	3	0.01
256	309.30	7	0.02
257	311.20	16	0.05
258	312.10	11	0.03
259	313.00	23	0.07
260	314.30	11	0.03
261	315.20	24	0.07
262	316.20	11	0.03
263	317.20	3	0.01
264	321.00	3	0.01
265	323.20	37	0.11
266	325.20	139	0.41
267	326.20	34	0.10
268	327.20	161	0.47
269	328.10	14	0.04
270	329.20	3	0.01
271	330.10	15	0.04
272	339.20	37	0.11
273	340.30	15	0.04
274	341.20	550	1.60
275	342.20	135	0.39
276	343.10	22	0.06
277	344.20	30	0.09
278	345.20	11	0.03
279	347.10	3	0.01
280	353.00	7	0.02
281	354.20	12	0.04
282	355.00	492	1.44
283	356.00	134	0.39
284	357.00	70	0.20
285	358.00	11	0.03
286	359.00	3	0.01
287	365.20	49	0.14
288	367.00	3	0.01
289	368.20	11	0.03
290	369.10	14	0.04
291	371.00	3	0.01
292	376.20	3	0.01
293	377.20	3	0.01
294	379.20	30	0.09
295	380.20	7	0.02
296	381.30	7	0.02
297	383.20	8	0.02
298	384.20	8	0.02
299	387.10	3	0.01
300	392.20	7	0.02
301	393.20	173	0.50
302	394.20	68	0.20
303	395.00	11	0.03
304	397.20	19	0.06
305	398.20	8	0.02
306	401.00	3	0.01
307	403.00	4	0.01
308	407.20	43	0.13
309	408.20	95	0.28

DEPTT. OF BOTANICAL & ENVIRONMENTAL SCIENCES, G.N.D.U. AMRITSAR

#	m/z	Abs. Int.	Rel. Int.
310	409.20	92	0.27
311	410.10	10	0.03
312	411.30	522	1.52
313	412.30	158	0.46
314	413.40	8	0.02
315	414.10	3	0.01
316	415.30	11	0.03
317	417.10	11	0.03
318	420.00	3	0.01
319	422.30	8	0.02
320	423.20	11	0.03
321	424.10	11	0.03
322	425.30	42	0.12
323	426.30	305	0.89
324	427.30	235	0.69
325	428.30	33	0.10
326	429.30	116	0.34
327	430.30	18	0.05
328	431.10	11	0.03
329	438.00	7	0.02
330	453.30	18	0.05
331	454.30	8	0.02
332	468.40	79	0.23
333	469.40	3	0.01
334	478.10	3	0.01
335	479.30	3	0.01
336	507.10	3	0.01
337	532.10	3	0.01
338	568.10	3	0.01
339	589.00	3	0.01
340	597.10	3	0.01
341	613.30	3	0.01
342	647.10	7	0.02
343	652.10	3	0.01
344	682.10	3	0.01
345	692.00	3	0.01

Line#:21 R.Time:38.0(Scan#:10201)
MassPeaks:439
RawMode:Averaged 37.7-38.1(10100-10217) BasePeak:69(243275)
BG Mode:None Group 1 - Event 1

#	m/z	Abs. Int.	Rel. Int.
1	50.05	984	0.40
2	51.05	1895	0.78
3	52.15	1279	0.53
4	53.10	22238	9.14
5	54.15	7755	3.19
6	55.10	170499	70.08
7	56.10	16956	6.97
8	57.10	75647	31.10
9	58.10	3832	1.58
10	59.10	2077	0.85
11	60.05	996	0.41
12	61.10	1002	0.41
13	62.10	381	0.16
14	63.10	745	0.31
15	64.15	563	0.23
16	65.05	8892	3.66
17	66.15	4168	1.71
18	67.05	144549	59.42
19	68.10	39820	16.37
20	69.10	243275	100.00
21	70.10	21311	8.76
22	71.10	17724	7.29
23	72.10	2541	1.04
24	73.10	8296	3.41
25	74.10	1604	0.66
26	75.05	2465	1.01
27	76.15	678	0.28
28	77.05	20842	8.57
29	78.15	4356	1.79
30	79.10	70308	28.90
31	80.10	13166	5.41
32	81.10	175577	72.17
33	82.10	77564	31.88
34	83.10	95693	39.34
35	84.10	9845	4.05
36	85.10	10422	4.28
37	86.10	2730	1.12
38	87.10	1476	0.61
39	88.10	1798	0.74
40	89.10	1706	0.70
41	90.15	508	0.21
42	91.05	50197	20.63
43	92.10	9278	3.81
44	93.10	94114	38.69
45	94.10	27400	11.26
46	95.10	223336	91.80
47	96.10	132065	54.29
48	97.10	52385	21.53
49	98.10	6461	2.66
50	99.10	4809	1.98
51	100.05	1238	0.51
52	101.10	1349	0.55
53	102.10	1791	0.74
54	103.05	3561	1.46
55	104.15	1627	0.67
56	105.10	51535	21.18
57	106.10	13961	5.74
58	107.10	103332	42.48
59	108.10	47373	19.47
60	109.10	187773	77.19
61	110.15	50244	20.65
62	111.10	50898	20.92
63	112.15	5406	2.22
64	113.10	4924	2.02
65	114.10	1589	0.65
66	115.10	4612	1.90
67	116.05	2545	1.05
68	117.10	8950	3.68
69	118.15	3310	1.36
70	119.10	56137	23.08
71	120.10	24226	9.96
72	121.10	96143	39.52
73	122.15	39894	16.40
74	123.15	160969	66.17
75	124.15	53420	21.96
76	125.15	122808	50.48
77	126.15	12917	5.31
78	127.15	5629	2.31
79	128.10	3388	1.39
80	129.10	4862	2.00
81	130.05	2177	0.89
82	131.10	12692	5.22
83	132.10	8942	3.68
84	133.10	52641	21.64
85	134.10	27119	11.15
86	135.10	69244	28.46
87	136.15	35180	14.46
88	137.15	61995	25.48
89	138.15	20874	8.58
90	139.10	9079	3.73
91	140.10	1706	0.70
92	141.10	2450	1.01
93	142.10	2256	0.93
94	143.05	5347	2.20
95	144.15	2620	1.08
96	145.10	20566	8.45
97	146.10	11154	4.58
98	147.10	42755	17.57
99	148.15	23311	9.58
100	149.15	53794	22.11
101	150.15	29095	11.96
102	151.15	23608	9.70
103	152.15	12415	5.10
104	153.15	4077	1.68
105	154.15	1229	0.51
106	155.10	1625	0.67
107	156.15	1238	0.51
108	157.10	5079	2.09
109	158.10	2674	1.10
110	159.10	17443	7.17
111	160.10	15765	6.48
112	161.15	53596	22.03
113	162.15	17696	7.27
114	163.15	67747	27.85
115	164.15	21752	8.94
116	165.15	12586	5.17
117	166.15	2421	1.00
118	167.20	1232	0.51
119	168.15	529	0.22
120	169.10	1189	0.49
121	170.10	818	0.34
122	171.10	4683	1.92
123	172.15	3224	1.33
124	173.15	15317	6.30
125	174.15	5580	2.29
126	175.15	28095	11.55
127	176.15	12159	5.00
128	177.15	23277	9.57
129	178.15	7878	3.24
130	179.15	41922	17.23
131	180.15	6725	2.76
132	181.15	1316	0.54
133	182.15	433	0.18
134	183.10	981	0.40
135	184.15	508	0.21
136	185.10	5950	2.45
137	186.10	2546	1.05
138	187.10	15137	6.22
139	188.10	5062	2.08
140	189.10	34241	14.08
141	190.10	15053	6.19
142	191.15	47837	19.66
143	192.10	12491	5.13
144	193.10	12610	5.18
145	194.10	3910	1.61
146	195.10	1431	0.59
147	196.10	332	0.14
148	197.05	836	0.34
149	198.15	414	0.17
150	199.10	5603	2.30
151	200.10	2285	0.94
152	201.10	11384	4.68
153	202.15	6643	2.73
154	203.10	25267	10.39
155	204.10	27356	11.24
156	205.10	50512	20.76
157	206.15	18723	7.70
158	207.10	21927	9.01
159	208.10	5429	2.23
160	209.00	2493	1.02
161	210.05	519	0.21
162	211.05	694	0.29
163	212.15	321	0.13
164	213.10	4274	1.76
165	214.10	2854	1.17
166	215.10	19101	7.85
167	216.10	5924	2.44
168	217.10	26801	11.02
169	218.10	58385	24.00
170	219.10	17610	7.24
171	220.10	16927	6.96
172	221.10	6098	2.51
173	222.15	1702	0.70
174	223.10	685	0.28
175	224.10	172	0.07
176	225.10	533	0.22
177	226.05	254	0.10
178	227.10	2751	1.13
179	228.10	1743	0.72
180	229.10	13856	5.70
181	230.10	5591	2.30
182	231.10	37203	15.29
183	232.10	25427	10.45
184	233.10	14445	5.94
185	234.10	11245	4.62
186	235.15	2688	1.10

DEPTT. OF BOTANICAL & ENVIRONMENTAL SCIENCES, G.N.D.U. AMRITSAR

#	m/z	Abs. Int.	Rel. Int.	#	m/z	Abs. Int.	Rel. Int.	#	m/z	Abs. Int.	Rel. Int.
187	236.15	489	0.20	267	316.20	172	0.07	347	400.20	5	0.00
188	237.10	335	0.14	268	317.20	109	0.04	348	401.20	32	0.01
189	238.20	117	0.05	269	318.20	13	0.01	349	402.20	7	0.00
190	239.15	551	0.23	270	319.20	13	0.01	350	403.20	3	0.00
191	240.10	212	0.09	271	320.30	11	0.00	351	404.20	2	0.00
192	241.10	2664	1.10	272	321.20	25	0.01	352	405.20	5	0.00
193	242.10	1034	0.43	273	322.20	10	0.00	353	406.20	9	0.00
194	243.15	3201	1.32	274	323.20	201	0.08	354	407.20	42	0.02
195	244.10	2242	0.92	275	324.20	69	0.03	355	408.20	481	0.20
196	245.10	14675	6.03	276	325.15	421	0.17	356	409.25	457	0.19
197	246.10	32130	13.21	277	326.20	211	0.09	357	410.20	325	0.13
198	247.10	12701	5.22	278	327.20	1037	0.43	358	411.25	8225	3.38
199	248.15	10411	4.28	279	328.20	336	0.14	359	412.20	2678	1.10
200	249.05	3150	1.29	280	329.20	310	0.13	360	413.20	481	0.20
201	250.05	585	0.24	281	330.30	92	0.04	361	414.20	54	0.02
202	251.00	546	0.22	282	331.20	114	0.05	362	415.20	56	0.02
203	252.10	154	0.06	283	332.20	1	0.00	363	416.20	8	0.00
204	253.10	515	0.21	284	333.30	3	0.00	364	417.20	3	0.00
205	254.15	232	0.10	285	334.20	12	0.00	365	418.20	2	0.00
206	255.15	8591	3.53	286	335.20	7	0.00	366	420.20	3	0.00
207	256.15	2500	1.03	287	337.20	104	0.04	367	421.20	4	0.00
208	257.10	12336	5.07	288	338.20	40	0.02	368	422.20	34	0.01
209	258.15	2999	1.23	289	339.15	588	0.24	369	423.20	101	0.04
210	259.10	8784	3.61	290	340.25	305	0.13	370	424.20	140	0.06
211	260.15	2686	1.10	291	341.25	6638	2.73	371	425.30	146	0.06
212	261.15	4067	1.67	292	342.20	1976	0.81	372	426.25	9560	3.93
213	262.20	1274	0.52	293	343.20	463	0.19	373	427.25	3339	1.37
214	263.15	497	0.20	294	344.20	1291	0.53	374	428.25	699	0.29
215	264.00	153	0.06	295	345.20	350	0.14	375	429.25	338	0.14
216	265.00	1168	0.48	296	346.20	33	0.01	376	430.20	133	0.05
217	266.00	392	0.16	297	347.20	3	0.00	377	431.30	36	0.01
218	266.95	1380	0.57	298	348.20	5	0.00	378	432.10	3	0.00
219	268.10	427	0.18	299	349.20	4	0.00	379	433.30	1	0.00
220	269.15	3867	1.59	300	350.20	9	0.00	380	437.40	1	0.00
221	270.15	1625	0.67	301	351.20	52	0.02	381	438.20	19	0.01
222	271.15	3233	1.33	302	352.20	19	0.01	382	439.30	7	0.00
223	272.15	2274	0.93	303	353.10	114	0.05	383	440.20	49	0.02
224	273.15	36304	14.92	304	354.15	392	0.16	384	441.30	13	0.01
225	274.15	18532	7.62	305	355.10	1213	0.50	385	442.30	14	0.01
226	275.15	7481	3.08	306	356.10	494	0.20	386	444.30	1	0.00
227	276.15	1401	0.58	307	357.05	413	0.17	387	445.30	1	0.00
228	277.05	253	0.10	308	358.00	130	0.05	388	446.30	26	0.01
229	278.10	52	0.02	309	359.10	15	0.01	389	447.30	4	0.00
230	279.20	75	0.03	310	360.20	3	0.00	390	451.30	3	0.00
231	280.00	36	0.01	311	361.10	1	0.00	391	452.30	13	0.01
232	281.00	5677	2.33	312	363.00	5	0.00	392	453.40	113	0.05
233	281.95	1618	0.67	313	365.20	196	0.08	393	454.30	26	0.01
234	283.05	1652	0.68	314	366.10	48	0.02	394	455.30	3	0.00
235	284.15	1549	0.64	315	367.10	38	0.02	395	456.40	3	0.00
236	285.15	2596	1.07	316	368.10	42	0.02	396	461.30	3	0.00
237	286.20	2272	0.93	317	369.15	360	0.15	397	464.10	1	0.00
238	287.15	9574	3.94	318	370.20	168	0.07	398	467.30	1	0.00
239	288.15	2673	1.10	319	371.20	114	0.05	399	468.40	210	0.09
240	289.15	655	0.27	320	372.10	12	0.00	400	469.10	76	0.03
241	290.20	147	0.06	321	373.10	1	0.00	401	470.30	3	0.00
242	291.10	74	0.03	322	375.20	1	0.00	402	471.10	7	0.00
243	292.20	29	0.01	323	376.10	1	0.00	403	472.10	2	0.00
244	293.10	78	0.03	324	377.20	19	0.01	404	473.30	1	0.00
245	294.10	19	0.01	325	378.10	12	0.00	405	476.20	1	0.00
246	295.10	370	0.15	326	379.20	140	0.06	406	480.30	5	0.00
247	296.20	116	0.05	327	380.10	55	0.02	407	490.30	4	0.00
248	297.20	523	0.21	328	381.20	68	0.03	408	491.30	1	0.00
249	298.20	225	0.09	329	382.20	12	0.00	409	492.30	1	0.00
250	299.20	911	0.37	330	383.20	348	0.14	410	494.30	1	0.00
251	300.20	555	0.23	331	384.20	598	0.25	411	497.30	3	0.00
252	301.25	866	0.36	332	385.20	188	0.08	412	499.30	125	0.05
253	302.20	14163	5.82	333	386.20	13	0.01	413	500.30	43	0.02
254	303.15	5812	2.39	334	387.00	1	0.00	414	503.30	7	0.00
255	304.20	1018	0.42	335	388.20	3	0.00	415	504.30	5	0.00
256	305.20	143	0.06	336	389.20	8	0.00	416	505.30	1	0.00
257	306.20	35	0.01	337	390.20	10	0.00	417	522.30	3	0.00
258	307.20	45	0.02	338	391.20	82	0.03	418	526.30	1	0.00
259	308.20	5	0.00	339	392.20	38	0.02	419	538.30	1	0.00
260	309.20	172	0.07	340	393.25	820	0.34	420	545.30	1	0.00
261	310.20	32	0.01	341	394.20	284	0.12	421	557.30	1	0.00
262	311.20	243	0.10	342	395.30	168	0.07	422	558.30	1	0.00
263	312.20	102	0.04	343	396.30	75	0.03	423	559.10	3	0.00
264	313.20	357	0.15	344	397.20	220	0.09	424	562.30	1	0.00
265	314.20	176	0.07	345	398.20	119	0.05	425	583.20	1	0.00
266	315.20	404	0.17	346	399.30	56	0.02	426	596.30	1	0.00

DEPTT. OF BOTANICAL & ENVIRONMENTAL SCIENCES, G.N.D.U. AMRITSAR

#	m/z	Abs. Int.	Rel. Int.
427	603.30	1	0.00
428	606.10	1	0.00
429	607.30	1	0.00
430	608.30	1	0.00
431	610.30	1	0.00
432	618.30	1	0.00
433	626.30	1	0.00
434	633.10	3	0.00
435	646.30	1	0.00
436	665.30	1	0.00
437	687.30	3	0.00
438	694.10	1	0.00
439	699.30	1	0.00

Line#:22 R.Time:39.0(Scan#:10487)
MassPeaks:386
RawMode:Averaged 38.9-39.1(10467-10519) BasePeak:203(47878)
BG Mode:None Group 1 - Event 1

#	m/z	Abs. Int.	Rel. Int.
1	50.00	711	1.49
2	51.10	1092	2.28
3	52.10	645	1.35
4	53.10	3641	7.60
5	54.15	1326	2.77
6	55.15	25401	53.05
7	56.10	3566	7.45
8	57.10	16311	34.07
9	58.10	1637	3.42
10	59.10	2732	5.71
11	60.10	1059	2.21
12	61.10	954	1.99
13	62.10	301	0.63
14	63.10	599	1.25
15	64.10	481	1.00
16	65.05	2436	5.09
17	66.10	1078	2.25
18	67.10	15630	32.65
19	68.10	3412	7.13
20	69.10	24038	50.21
21	70.10	3311	6.92
22	71.10	10462	21.85
23	72.10	1139	2.38
24	73.10	15855	33.12
25	74.10	2122	4.43
26	75.05	2504	5.23
27	76.10	616	1.29
28	77.10	6472	13.52
29	78.10	1766	3.69
30	79.10	13813	28.85
31	80.15	3158	6.60
32	81.10	22682	47.37
33	82.10	5388	11.25
34	83.10	11986	25.03
35	84.10	2335	4.88
36	85.10	6052	12.64
37	86.10	724	1.51
38	87.05	1909	3.99
39	88.10	636	1.33
40	89.05	1269	2.65
41	90.10	367	0.77
42	91.10	15775	32.95
43	92.10	2799	5.85
44	93.10	16578	34.63
45	94.10	4390	9.17
46	95.10	25429	53.11
47	96.10	10481	21.89
48	97.10	8836	18.46
49	98.15	2039	4.26
50	99.10	2339	4.89
51	100.20	576	1.20
52	101.10	647	1.35
53	102.10	488	1.02
54	103.05	2640	5.51
55	104.10	1183	2.47
56	105.10	18925	39.53
57	106.10	4067	8.49
58	107.10	17209	35.94
59	108.15	5812	12.14
60	109.10	14361	29.99
61	110.15	3170	6.62
62	111.15	4935	10.31
63	112.15	1204	2.51
64	113.15	1835	3.83
65	114.15	503	1.05
66	115.05	3425	7.15
67	116.10	1367	2.86
68	117.10	5490	11.47
69	118.10	1966	4.11
70	119.10	20100	41.98
71	120.10	6735	14.07
72	121.10	14491	30.27
73	122.15	6067	12.67
74	123.15	11190	23.37
75	124.15	2346	4.90
76	125.20	5017	10.48
77	126.15	1235	2.58
78	127.10	1741	3.64
79	128.10	2208	4.61
80	129.10	3827	7.99
81	130.10	1506	3.15
82	131.10	7463	15.59
83	132.10	2511	5.24
84	133.10	45887	95.84
85	134.15	9994	20.87
86	135.10	22941	47.92
87	136.15	5556	11.60
88	137.15	4618	9.65
89	138.15	1298	2.71
90	139.15	2444	5.10
91	140.10	746	1.56
92	141.10	2285	4.77
93	142.10	1597	3.34
94	143.10	3804	7.95
95	144.10	1749	3.65
96	145.15	6999	14.62
97	146.15	2484	5.19
98	147.10	14479	30.24
99	148.15	5117	10.69
100	149.15	8540	17.84
101	150.15	2285	4.77
102	151.15	2438	5.09
103	152.15	1159	2.42
104	153.15	1417	2.96
105	154.15	854	1.78
106	155.15	1819	3.80
107	156.15	1013	2.12
108	157.10	3020	6.31
109	158.15	1289	2.69
110	159.15	5455	11.39
111	160.15	1933	4.04
112	161.15	7606	15.89
113	162.15	2961	6.18
114	163.15	5662	11.83
115	164.15	1449	3.03
116	165.15	2345	4.90
117	166.15	840	1.75
118	167.10	1263	2.64
119	168.15	544	1.14
120	169.10	1386	2.89
121	170.15	615	1.28
122	171.15	2274	4.75
123	172.15	1094	2.28
124	173.15	4087	8.54
125	174.20	1962	4.10
126	175.15	12526	26.16
127	176.20	4155	8.68
128	177.15	5078	10.61
129	178.10	1464	3.06
130	179.10	1780	3.72
131	180.10	562	1.17
132	181.10	723	1.51
133	182.10	394	0.82
134	183.15	1106	2.31
135	184.10	445	0.93
136	185.15	1961	4.10
137	186.15	842	1.76
138	187.15	3873	8.09
139	188.15	1496	3.12
140	189.15	11463	23.94
141	190.20	7150	14.93
142	191.10	9429	19.69
143	192.15	2711	5.66
144	193.10	3713	7.76
145	194.10	872	1.82
146	195.10	828	1.73
147	196.00	349	0.73
148	197.10	941	1.97
149	198.10	351	0.73
150	199.10	2177	4.55
151	200.15	745	1.56
152	201.15	2827	5.90
153	202.25	1576	3.29
154	203.15	47878	100.00
155	204.15	9908	20.69
156	205.15	3456	7.22
157	206.15	1788	3.73
158	207.10	40281	84.13
159	208.10	8811	18.40
160	209.05	4299	8.98
161	210.00	788	1.65
162	211.05	861	1.80
163	212.10	327	0.68
164	213.10	1248	2.61
165	214.15	1668	3.48
166	215.15	2133	4.46
167	216.15	857	1.79
168	217.15	3217	6.72
169	218.15	2448	5.11
170	219.15	1742	3.64
171	220.10	777	1.62
172	221.10	2214	4.62
173	222.10	659	1.38
174	223.10	838	1.75
175	224.10	306	0.64
176	225.10	630	1.32
177	226.10	315	0.66
178	227.10	947	1.98
179	228.10	380	0.79
180	229.20	1156	2.41
181	230.10	506	1.06
182	231.10	1027	2.15
183	232.15	8889	18.57
184	233.15	2454	5.13
185	234.15	836	1.75
186	235.10	920	1.92
187	236.10	326	0.68
188	237.10	430	0.90
189	238.10	167	0.35
190	239.10	665	1.39
191	240.10	288	0.60
192	241.15	832	1.74
193	242.10	271	0.57
194	243.20	842	1.76
195	244.10	358	0.75
196	245.20	660	1.38
197	246.20	451	0.94
198	247.10	550	1.15
199	248.05	860	1.80
200	248.95	2069	4.32
201	249.90	621	1.30
202	250.90	1031	2.15
203	252.00	351	0.73
204	253.10	680	1.42
205	254.00	244	0.51
206	255.20	829	1.73
207	256.10	320	0.67

DEPTT. OF BOTANICAL & ENVIRONMENTAL SCIENCES, G.N.D.U. AMRITSAR

#	m/z	Abs. Int.	Rel. Int.
208	257.15	945	1.97
209	258.10	394	0.82
210	259.10	498	1.04
211	260.10	202	0.42
212	261.00	235	0.49
213	262.15	421	0.88
214	263.20	311	0.65
215	264.00	83	0.17
216	265.00	2568	5.36
217	266.00	790	1.65
218	267.00	2554	5.33
219	268.05	743	1.55
220	269.10	942	1.97
221	270.10	331	0.69
222	271.20	1256	2.62
223	272.20	450	0.94
224	273.15	6884	14.38
225	274.15	1643	3.43
226	275.20	401	0.84
227	276.20	30	0.06
228	277.20	141	0.29
229	278.20	17	0.04
230	279.20	175	0.37
231	280.00	67	0.14
232	281.00	10755	22.46
233	282.05	2993	6.25
234	283.05	2168	4.53
235	284.15	717	1.50
236	285.10	719	1.50
237	286.10	526	1.10
238	287.15	862	1.80
239	288.20	300	0.63
240	289.00	121	0.25
241	290.20	8	0.02
242	291.20	17	0.04
243	292.00	12	0.03
244	293.00	213	0.44
245	294.20	21	0.04
246	295.20	510	1.07
247	296.10	144	0.30
248	297.20	319	0.67
249	298.10	130	0.27
250	299.25	410	0.86
251	300.20	297	0.62
252	301.20	296	0.62
253	302.20	73	0.15
254	303.10	60	0.13
255	305.00	29	0.06
256	306.00	8	0.02
257	307.20	59	0.12
258	308.00	16	0.03
259	309.00	92	0.19
260	310.20	8	0.02
261	311.20	222	0.46
262	312.10	26	0.05
263	313.10	168	0.35
264	314.20	16	0.03
265	315.10	140	0.29
266	316.20	4	0.01
267	317.20	4	0.01

#	m/z	Abs. Int.	Rel. Int.
268	318.20	8	0.02
269	320.10	4	0.01
270	321.10	35	0.07
271	322.20	34	0.07
272	323.20	141	0.29
273	324.20	38	0.08
274	325.20	471	0.98
275	326.00	136	0.28
276	327.00	441	0.92
277	328.20	103	0.22
278	329.20	112	0.23
279	330.20	8	0.02
280	331.00	9	0.02
281	333.10	16	0.03
282	334.20	4	0.01
283	335.00	8	0.02
284	336.20	4	0.01
285	337.10	59	0.12
286	338.30	15	0.03
287	339.10	174	0.36
288	340.10	51	0.11
289	341.00	1015	2.12
290	342.00	407	0.85
291	343.00	332	0.69
292	344.00	75	0.16
293	345.10	10	0.02
294	346.00	4	0.01
295	351.30	21	0.04
296	352.00	8	0.02
297	353.20	82	0.17
298	354.10	9	0.02
299	355.05	1632	3.41
300	356.10	600	1.25
301	357.00	437	0.91
302	358.00	53	0.11
303	360.00	4	0.01
304	361.00	13	0.03
305	365.10	8	0.02
306	367.00	7	0.01
307	368.10	8	0.02
308	369.10	82	0.17
309	370.00	24	0.05
310	371.10	24	0.05
311	372.10	8	0.02
312	373.00	4	0.01
313	378.10	3	0.01
314	379.10	111	0.23
315	380.00	8	0.02
316	381.00	37	0.08
317	382.10	4	0.01
318	383.10	30	0.06
319	385.00	4	0.01
320	386.00	3	0.01
321	387.00	3	0.01
322	389.10	17	0.04
323	390.00	4	0.01
324	391.10	42	0.09
325	392.10	12	0.03
326	393.10	151	0.32
327	394.10	69	0.14

#	m/z	Abs. Int.	Rel. Int.
328	395.00	54	0.11
329	396.00	7	0.01
330	397.10	78	0.16
331	398.00	7	0.01
332	399.10	49	0.10
333	401.00	164	0.34
334	402.00	13	0.03
335	403.00	3	0.01
336	404.10	8	0.02
337	405.00	81	0.17
338	406.10	8	0.02
339	407.35	338	0.71
340	408.10	153	0.32
341	409.10	81	0.17
342	410.10	77	0.16
343	411.10	144	0.30
344	412.10	60	0.13
345	413.10	8	0.02
346	415.10	178	0.37
347	416.00	42	0.09
348	417.00	16	0.03
349	422.10	118	0.25
350	423.00	66	0.14
351	424.10	35	0.07
352	425.10	227	0.47
353	426.30	139	0.29
354	427.30	8	0.02
355	428.00	12	0.03
356	429.10	572	1.19
357	430.00	256	0.53
358	431.00	145	0.30
359	432.00	7	0.01
360	433.10	3	0.01
361	440.30	525	1.10
362	441.30	315	0.66
363	442.30	82	0.17
364	443.00	20	0.04
365	451.00	4	0.01
366	456.10	11	0.02
367	459.00	4	0.01
368	461.30	4	0.01
369	467.00	3	0.01
370	477.00	3	0.01
371	489.00	3	0.01
372	502.10	3	0.01
373	503.00	68	0.14
374	504.00	12	0.03
375	511.30	4	0.01
376	523.00	3	0.01
377	536.00	3	0.01
378	538.30	4	0.01
379	557.00	8	0.02
380	587.10	4	0.01
381	612.30	3	0.01
382	627.20	4	0.01
383	638.00	3	0.01
384	661.00	4	0.01
385	668.20	4	0.01
386	685.30	4	0.01

DEPTT. OF BOTANICAL & ENVIRONMENTAL SCIENCES, G.N.D.U. AMRITSAR

Peak Report TIC

Peak#	R.Time	Area	Area%	Height	Name
1	17.643	418612	0.14	237919	3,7,11,15-Tetramethyl-2-hexadecen-1-ol $$ (2E)-3,7,11,15-Tetramethyl-2-hexadecen-1-ol # $$
2	18.415	306527	0.10	225334	Pentadecanoic acid, 14-methyl-, methyl ester (CAS) METHYL 14-METHYL-PENTADECANOATE $$ 14-METHYL-PENTA
3	18.827	1890608	0.62	891040	9-Octadecenoic acid (Z)- (CAS) Oleic acid $$ Red oil $$ Oelsauere $$ Oleine 7503 $$ Pamolyn 100 $$ Emersol 211 $$ Vopco
4	20.257	1413931	0.46	855274	2-Hexadecen-1-ol, 3,7,11,15-tetramethyl-, [R-[R*,R*-(E)]]- (CAS) Phytol $$ trans-Phytol $$ (E)-(7R,11R)-3,7,11,15-tetrameth
5	20.451	530997	0.17	311422	Linoleic acid P1365
6	20.699	320054	0.10	206703	Docosanoic acid (CAS) Behenic acid $$ Glycon B-70 $$ Hydrofol 2022-55 $$ Hydrofol Acid 560 $$ n-Docosanoic acid $$ 1-I
7	23.684	245925	0.08	182199	Octadecane P1197
8	23.745	268783	0.09	192511	1,2-Benzenedicarboxylic acid, ditridecyl ester $$ Phthalic acid, ditridecyl ester $$ Bis(tridecyl) phthalate $$ Ditridecyl pht
9	24.421	100518	0.03	66234	1-Decanol, 2-hexyl- $$ 2-Hexyl-1-decanol $$
10	25.393	1183712	0.39	628112	Tetratetracontane (CAS) n-Tetratetracontane $$
11	26.579	2894248	0.95	1275416	2,6,10,14,18,22-Tetracosahexaene, 2,6,10,15,19,23-hexamethyl-, (all-E)- $$ All-trans-Squalene $$ trans-Squalene $$ Spinacen
12	27.529	529345	0.17	164561	Acetic acid, chloro-, octadecyl ester $$ Chloroacetic acid, octadecyl ester $$ Octadecyl chloroacetate # $$
13	27.760	1148990	0.38	442773	Octadecane P1197
14	31.152	4098717	1.34	759936	Vitamin E $$ 2H-1-Benzopyran-6-ol, 3,4-dihydro-2,5,7,8-tetramethyl-2-(4,8,12-trimethyltridecyl)-, [2R-[2R*(4R*,8R*)]]- $$ .a
15	33.717	29999633	9.82	3642244	D:B-Friedo-18,19-secolup-19-ene, 3,10-epoxy-, (3.beta.,10.beta.)- $$ Baccharis oxide $$
16	35.186	5524365	1.81	986163	Cholest-5-ene, 3-bromo-, (3.beta.)- $$ Cholest-5-ene, 3.beta.-bromo- $$ Cholesteryl bromide $$ 3.beta.-Bromocholest-5-ene $$
17	35.445	26416901	8.65	4576507	METHYL COMMATE C $$
18	35.613	1830161	0.60	491237	METHYL COMMATE B $$
19	36.328	114449592	37.46	11215033	METHYL COMMATE D $$

DEPTT. OF BOTANICAL & ENVIRONMENTAL SCIENCES,
G.N.D.U.
AMRITSAR

Peak#	R.Time	Area	Area%	Height	Name
20	37.206	5695005	1.86	1576170	03027205002 FLAVONE 4'-OH,5-OH,7-DI-O-GLUCOSIDE $$
21	37.999	101436600	33.20	15225281	GLOBULOL $$ (-)-Globulol $$ 1H-Cycloprop[e]azulen-4-ol, decahydro-1,1,4,7-tetramethyl-, [1aR-(1a.alpha.,4.alpha.,4a.alpha
22	38.952	4845505	1.59	1278227	Olean-12-en-28-al (CAS)
		305548729	100.00	45430296	

Supplementary file S-5

GCMS Report Ethyl Acetate Leaves

DEPTT. OF BOTANICAL & ENVIRONMENTAL SCIENCES, G.N.D.U. AMRITSAR

Sample Information

Analyzed by	: Admin
Analyzed	: 8/22/2015 5:29:02 PM
Sample Type	: Unknown
Sample Name	: ACIDS
Sample ID	: ANKET
Injection Volume	: 2
Data File	: E:\GCMS\GCMS DATA\Vandana\4.qgd
Method File	: E:\GCMS\GCMS DATA\anket\ANKET. B. JUNCEA PROFILING.qgm
Tuning File	: C:\GCMSsolution\System\Tune1\05-06-2014.qgt

Chromatogram ACIDS E:\GCMS\GCMS DATA\Vandana\4.qgd

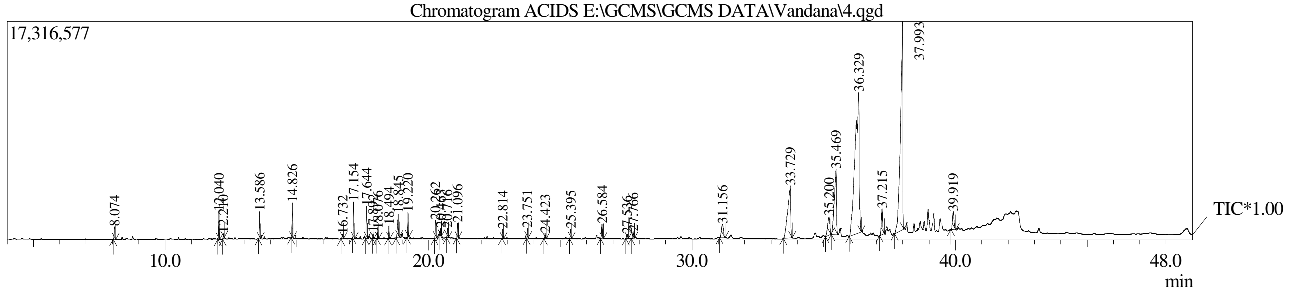

Spectrum

Line#:1 R.Time:8.1(Scan#:1223)
MassPeaks:168
RawMode:Averaged 8.0-8.2(1204-1259) BasePeak:55(29027)
BG Mode:None Group 1 - Event 1

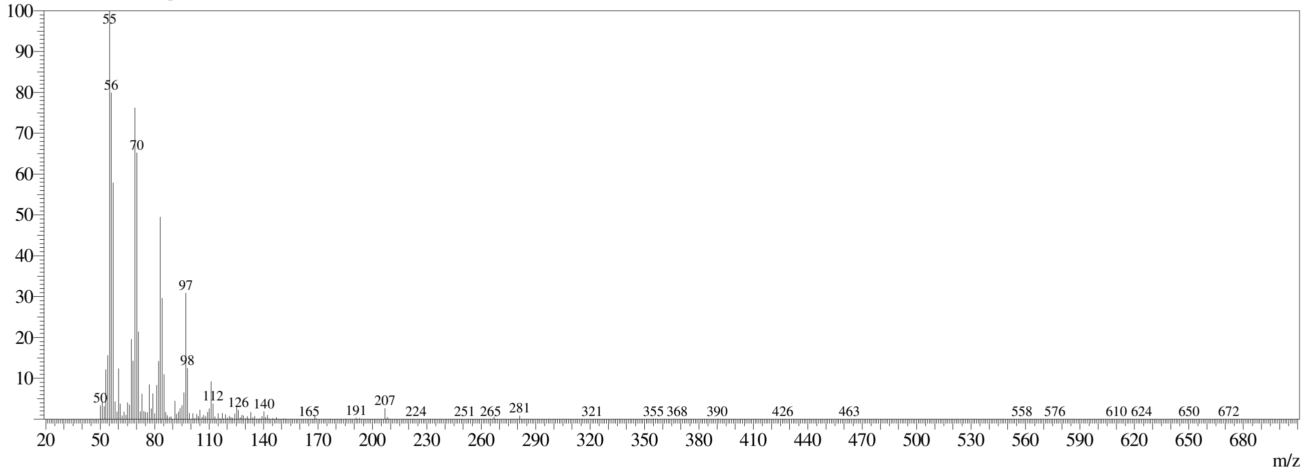

DEPTT. OF BOTANICAL & ENVIRONMENTAL SCIENCES, G.N.D.U. AMRITSAR

Line#:2 R.Time:12.0(Scan#:2413)
MassPeaks:161
RawMode:Averaged 12.0-12.1(2401-2427) BasePeak:55(83947)
BG Mode:None Group 1 - Event 1

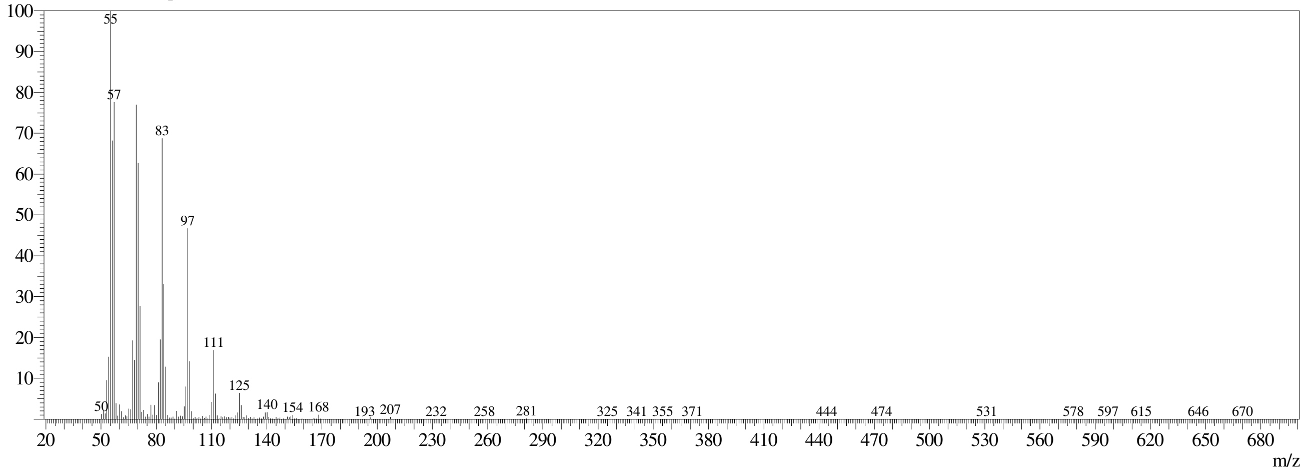

Line#:3 R.Time:12.2(Scan#:2464)
MassPeaks:155
RawMode:Averaged 12.2-12.2(2452-2472) BasePeak:57(52899)
BG Mode:None Group 1 - Event 1

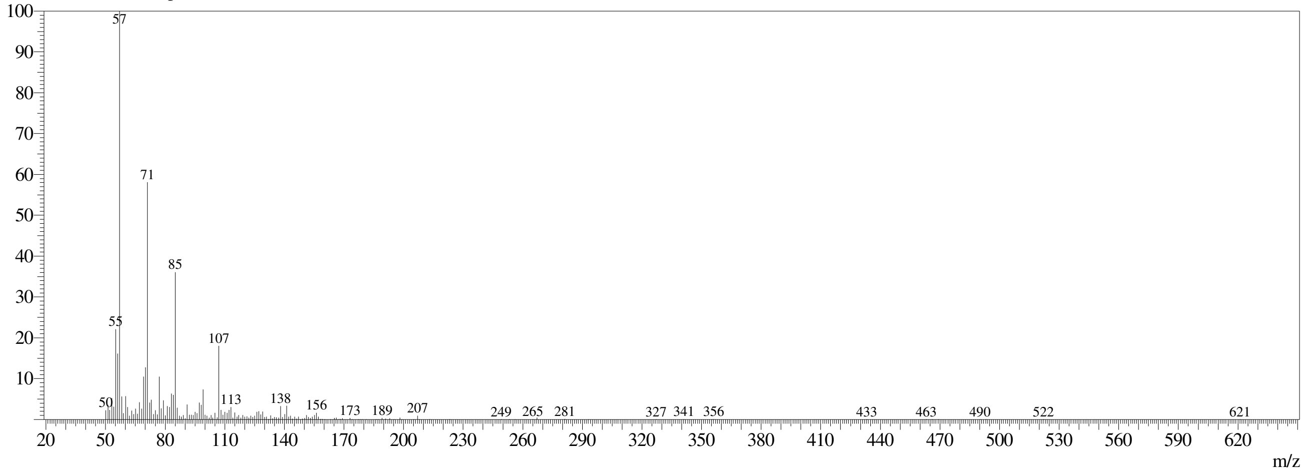

Line#:4 R.Time:13.6(Scan#:2877)
MassPeaks:159
RawMode:Averaged 13.6-13.6(2866-2889) BasePeak:191(169271)
BG Mode:None Group 1 - Event 1

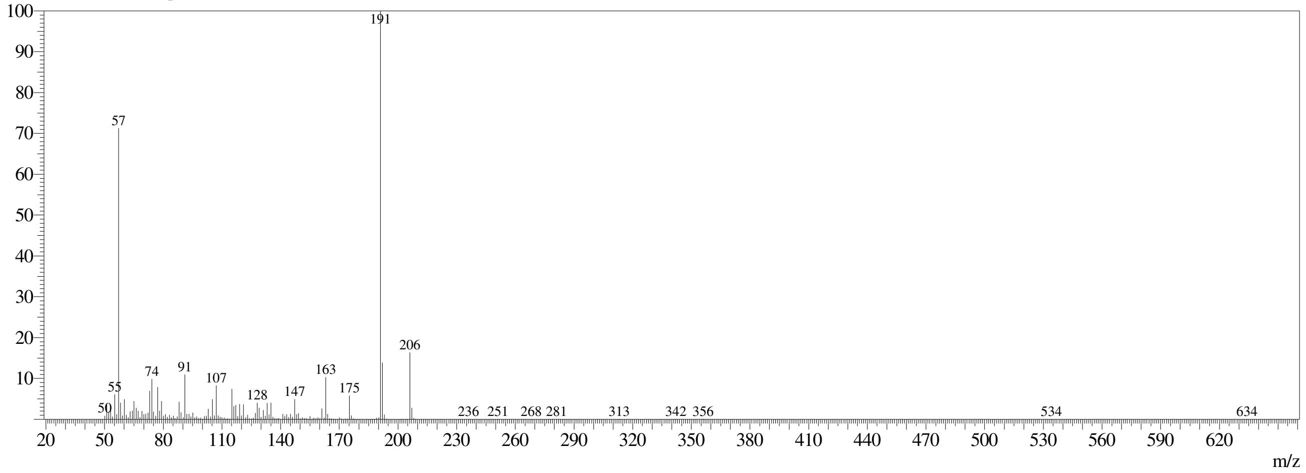

DEPTT. OF BOTANICAL & ENVIRONMENTAL SCIENCES, G.N.D.U. AMRITSAR

Line#:5 R.Time:14.8(Scan#:3249)
MassPeaks:183
RawMode:Averaged 14.8-14.9(3237-3261) BasePeak:55(102093)
BG Mode:None Group 1 - Event 1

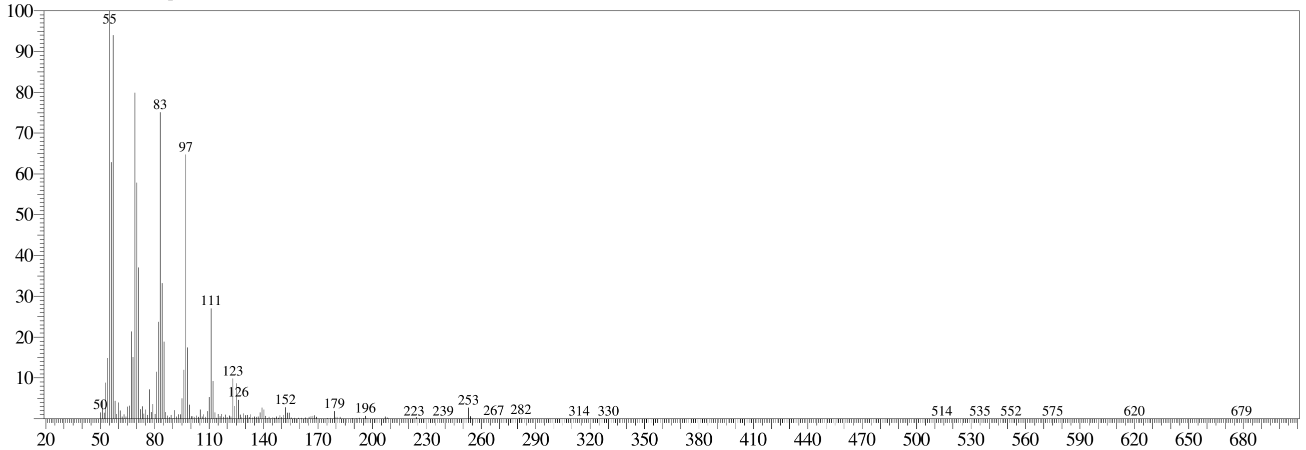

Line#:6 R.Time:16.7(Scan#:3821)
MassPeaks:189
RawMode:Averaged 16.7-16.8(3808-3836) BasePeak:57(17126)
BG Mode:None Group 1 - Event 1

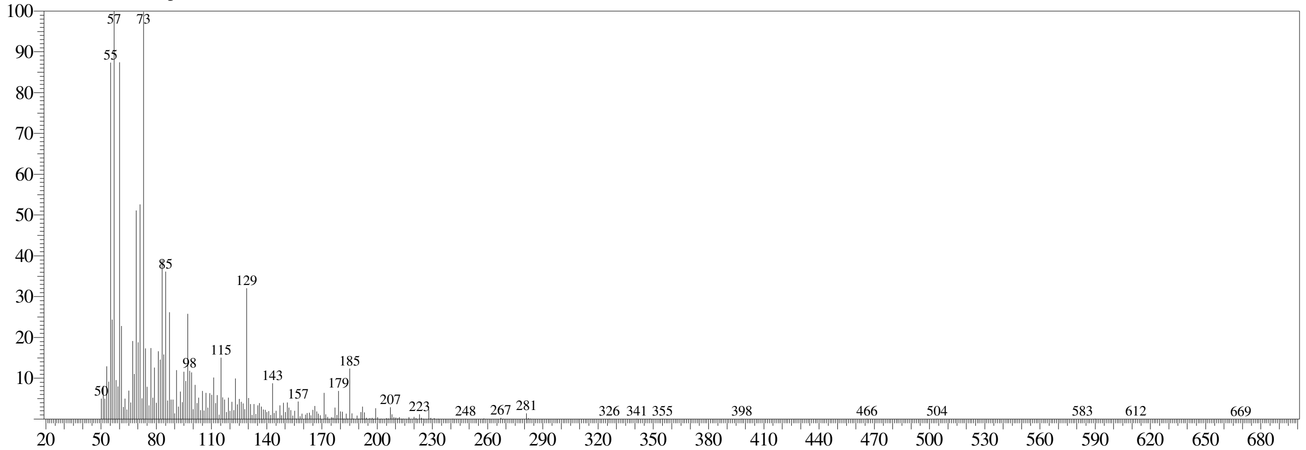

Line#:7 R.Time:17.2(Scan#:3947)
MassPeaks:191
RawMode:Averaged 17.1-17.2(3934-3958) BasePeak:55(95553)
BG Mode:None Group 1 - Event 1

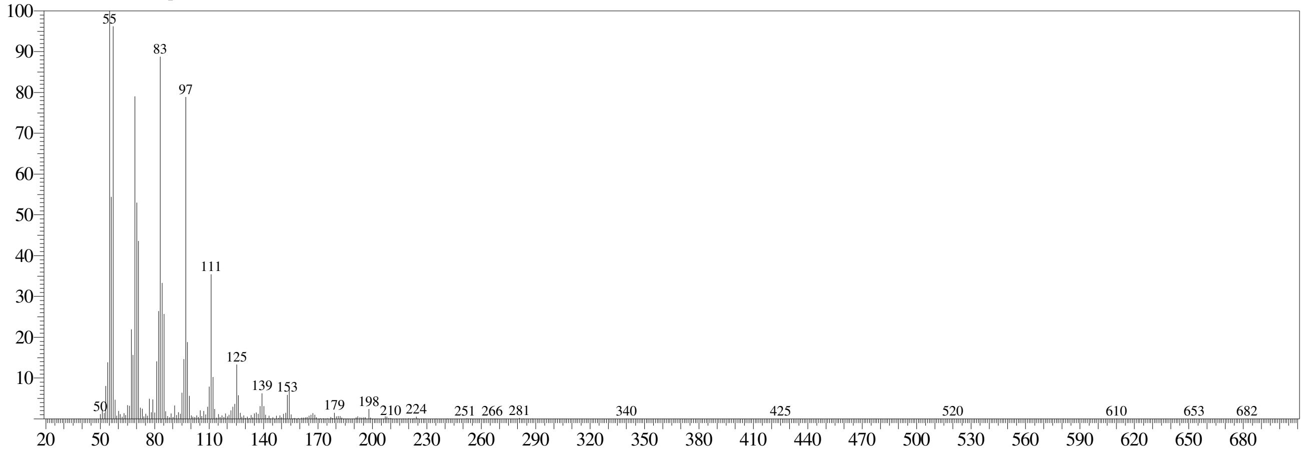

DEPTT. OF BOTANICAL & ENVIRONMENTAL SCIENCES, G.N.D.U. AMRITSAR

Line#:8 R.Time:17.6(Scan#:4094)
MassPeaks:186
RawMode:Averaged 17.6-17.7(4085-4107) BasePeak:68(90232)
BG Mode:None Group 1 - Event 1

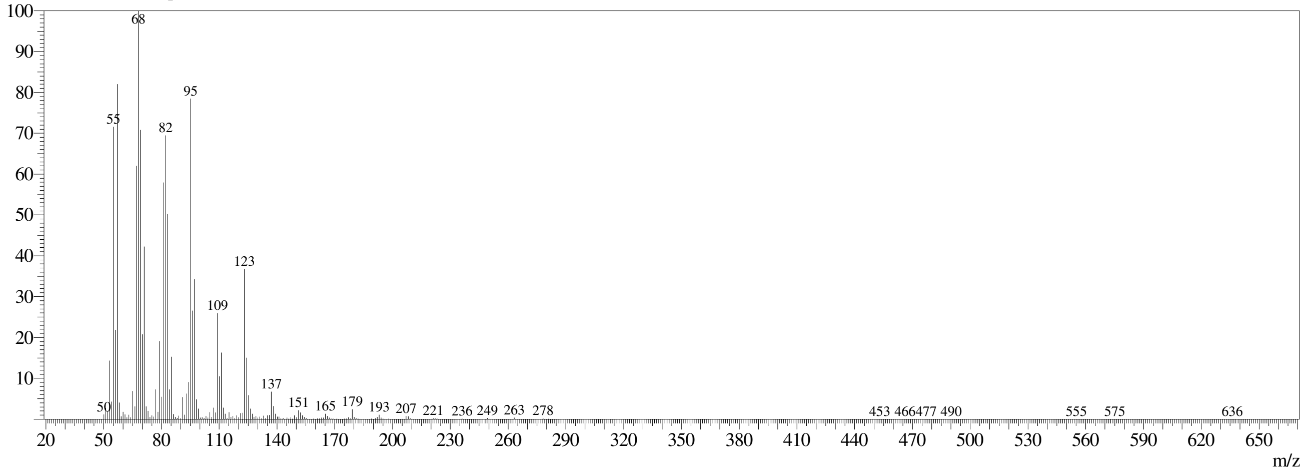

Line#:9 R.Time:17.9(Scan#:4168)
MassPeaks:210
RawMode:Averaged 17.8-17.9(4154-4181) BasePeak:57(15404)
BG Mode:None Group 1 - Event 1

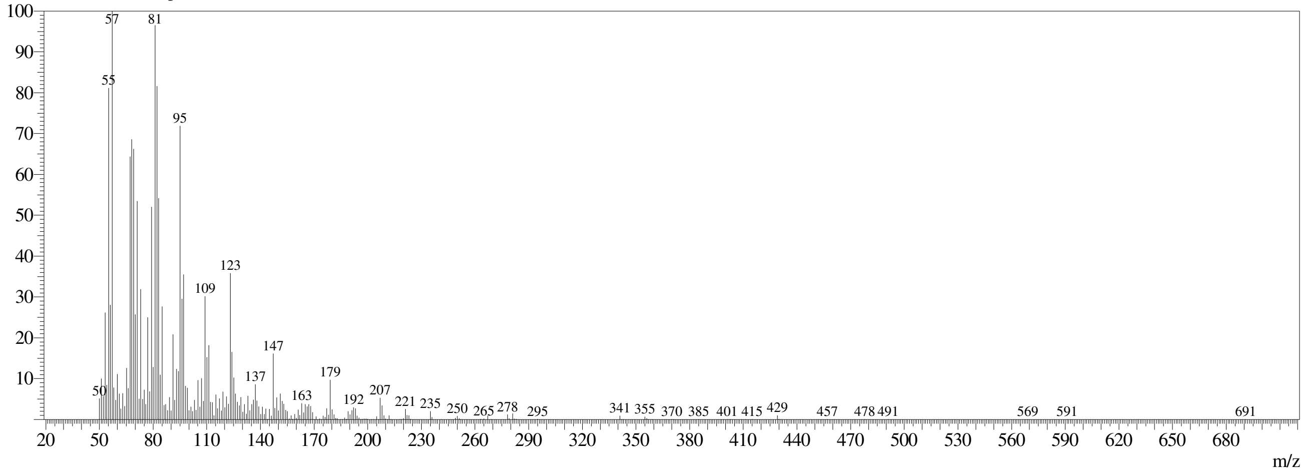

Line#:10 R.Time:18.1(Scan#:4224)
MassPeaks:190
RawMode:Averaged 18.0-18.1(4212-4239) BasePeak:81(22702)
BG Mode:None Group 1 - Event 1

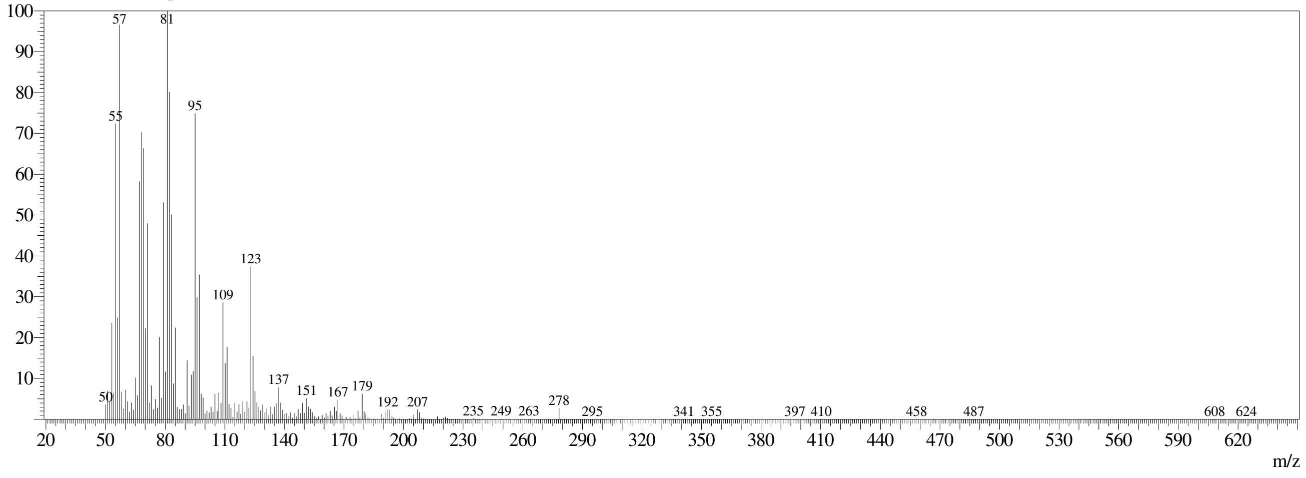

DEPTT. OF BOTANICAL & ENVIRONMENTAL SCIENCES, G.N.D.U. AMRITSAR

Line#:11 R.Time:18.5(Scan#:4349)
MassPeaks:207
RawMode:Averaged 18.5-18.5(4339-4360) BasePeak:149(185218)
BG Mode:None Group 1 - Event 1

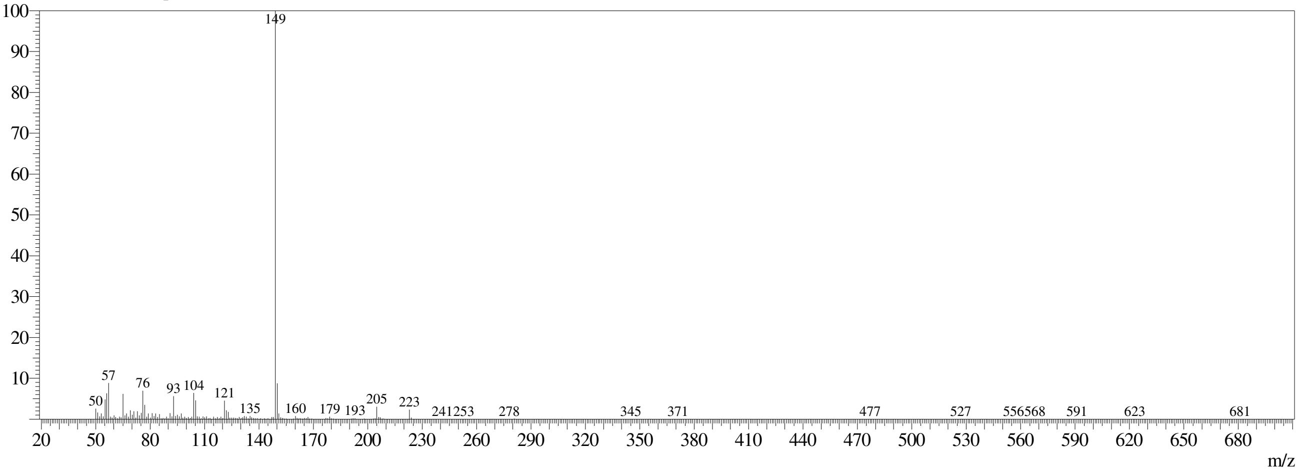

Line#:12 R.Time:18.8(Scan#:4455)
MassPeaks:219
RawMode:Averaged 18.8-18.9(4429-4468) BasePeak:73(69762)
BG Mode:None Group 1 - Event 1

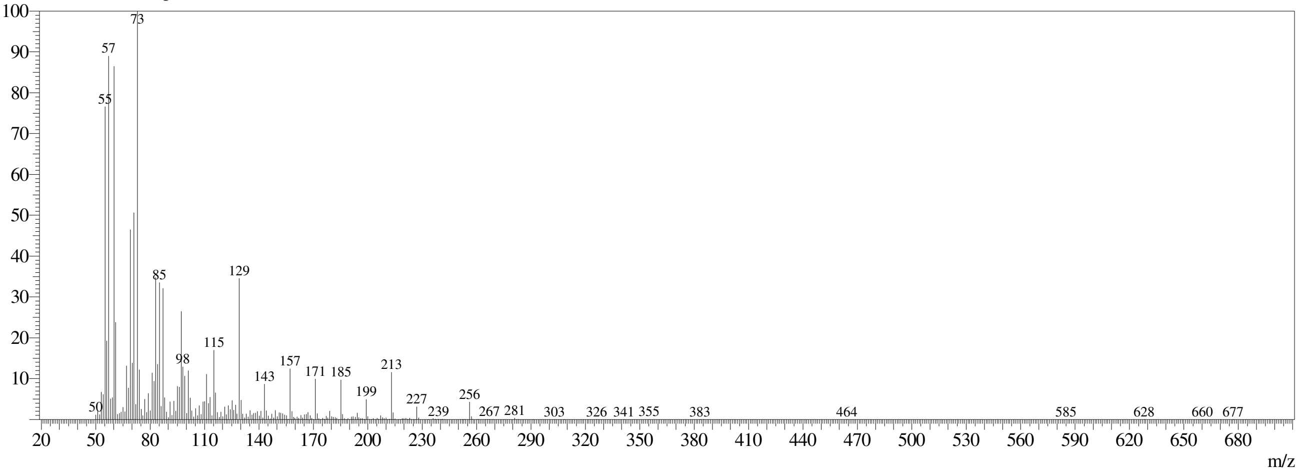

Line#:13 R.Time:19.2(Scan#:4567)
MassPeaks:202
RawMode:Averaged 19.2-19.3(4555-4576) BasePeak:57(81148)
BG Mode:None Group 1 - Event 1

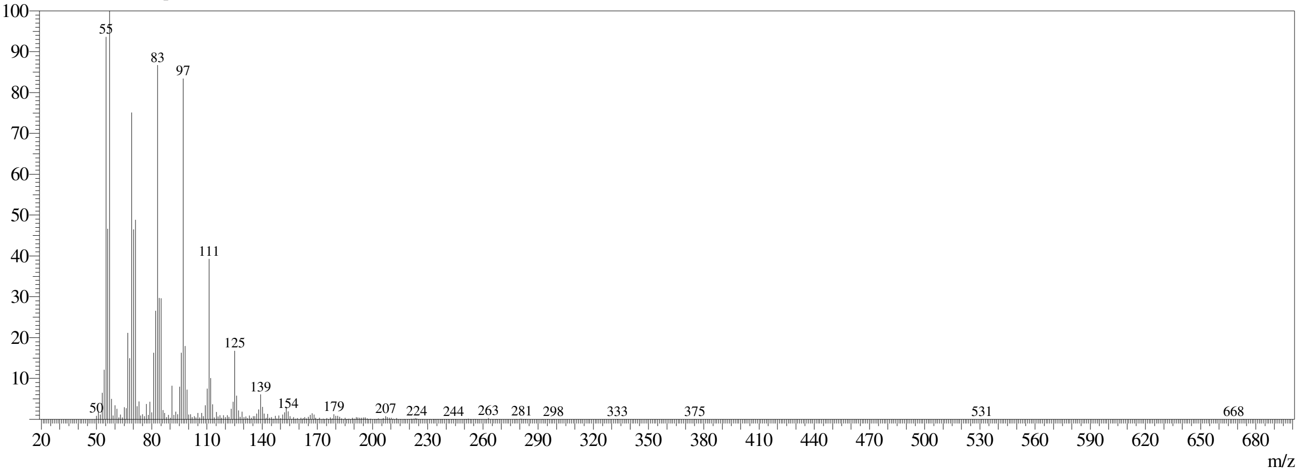

DEPTT. OF BOTANICAL & ENVIRONMENTAL SCIENCES, G.N.D.U. AMRITSAR

Line#:14 R.Time:20.3(Scan#:4880)
MassPeaks:207
RawMode:Averaged 20.2-20.3(4869-4897) BasePeak:71(90082)
BG Mode:None Group 1 - Event 1

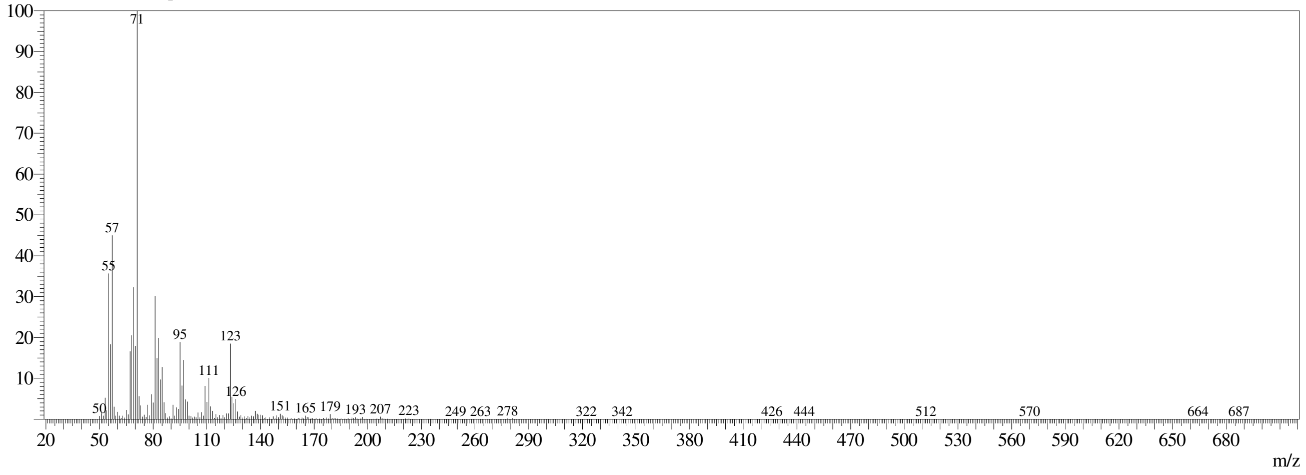

Line#:15 R.Time:20.5(Scan#:4940)
MassPeaks:220
RawMode:Averaged 20.4-20.5(4930-4952) BasePeak:55(41940)
BG Mode:None Group 1 - Event 1

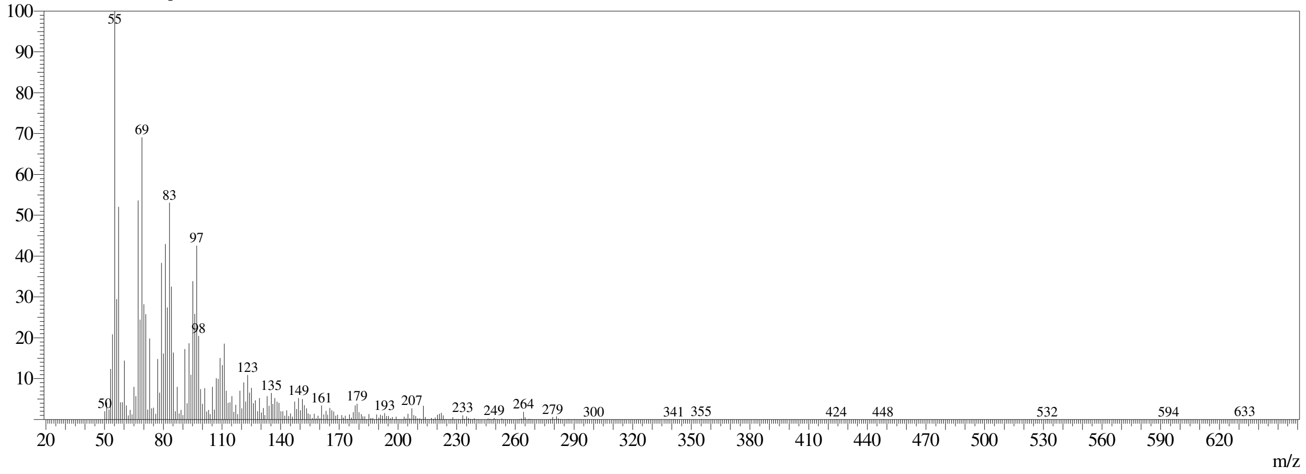

Line#:16 R.Time:20.7(Scan#:5016)
MassPeaks:219
RawMode:Averaged 20.7-20.8(5000-5032) BasePeak:57(27289)
BG Mode:None Group 1 - Event 1

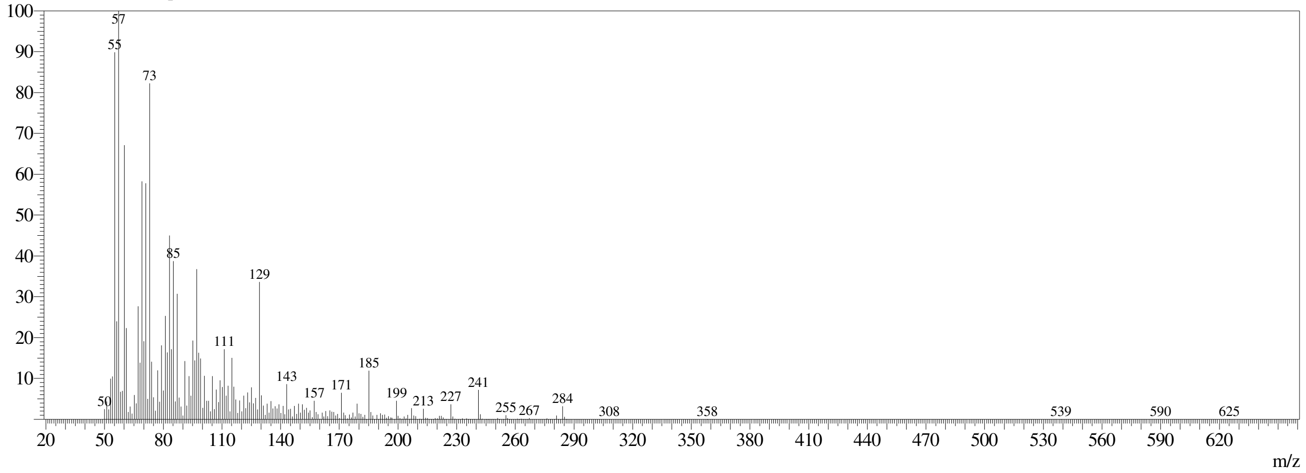

DEPTT. OF BOTANICAL & ENVIRONMENTAL SCIENCES, G.N.D.U. AMRITSAR

Line#:17 R.Time:21.1(Scan#:5130)
MassPeaks:204
RawMode:Averaged 21.1-21.1(5119-5144) BasePeak:57(46039)
BG Mode:None Group 1 - Event 1

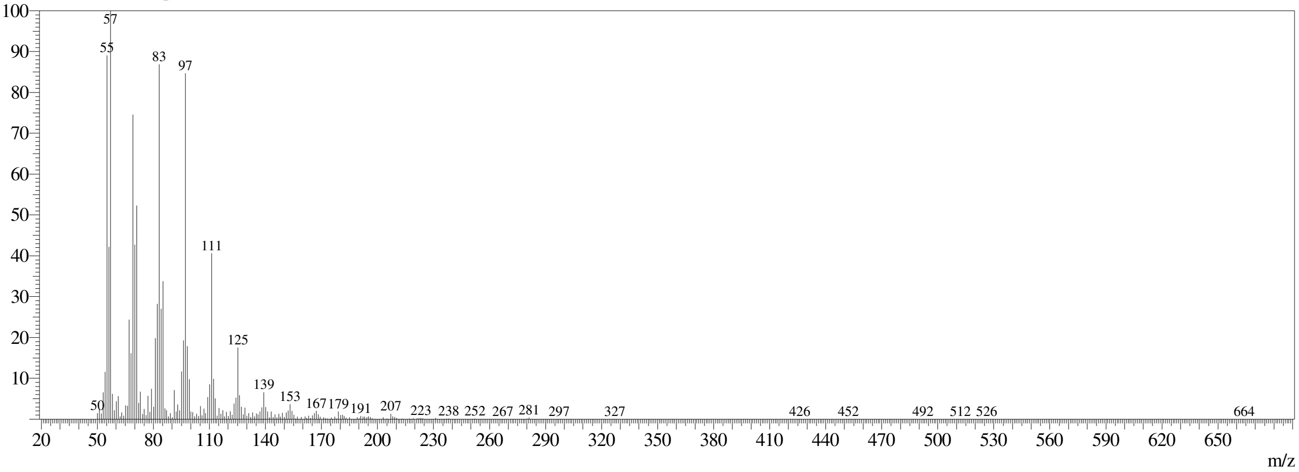

Line#:18 R.Time:22.8(Scan#:5645)
MassPeaks:209
RawMode:Averaged 22.8-22.9(5635-5656) BasePeak:57(31251)
BG Mode:None Group 1 - Event 1

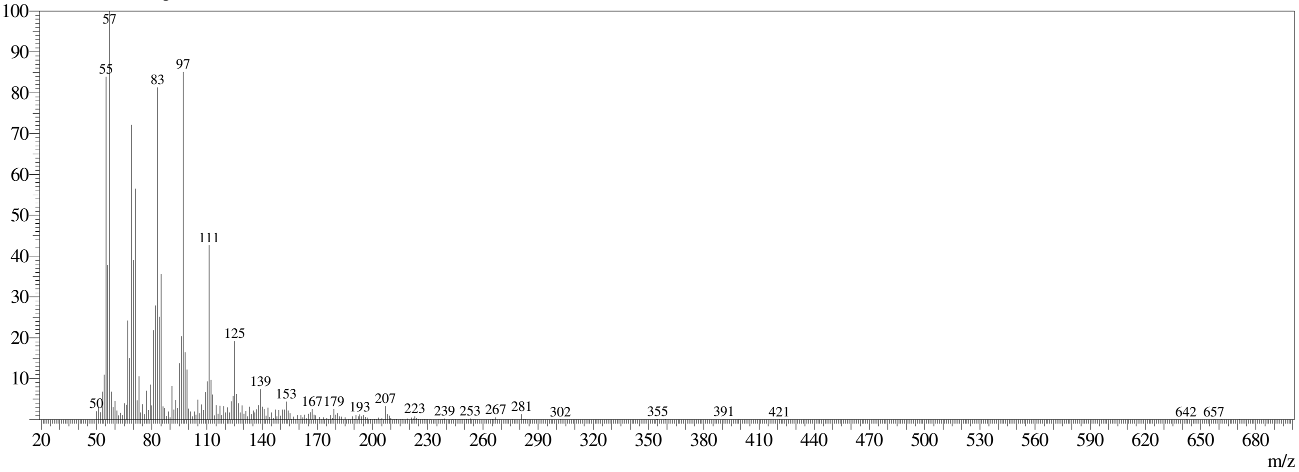

Line#:19 R.Time:23.8(Scan#:5926)
MassPeaks:209
RawMode:Averaged 23.7-23.8(5914-5936) BasePeak:149(71252)
BG Mode:None Group 1 - Event 1

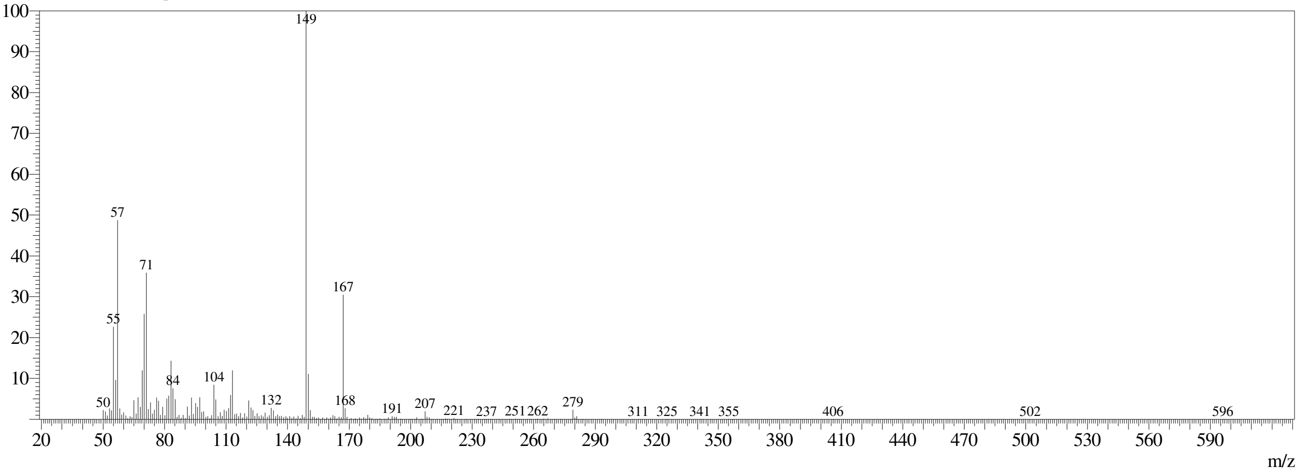

DEPTT. OF BOTANICAL & ENVIRONMENTAL SCIENCES, G.N.D.U. AMRITSAR

Line#:20 R.Time:24.4(Scan#:6128)
MassPeaks:212
RawMode:Averaged 24.4-24.5(6117-6139) BasePeak:57(20722)
BG Mode:None Group 1 - Event 1

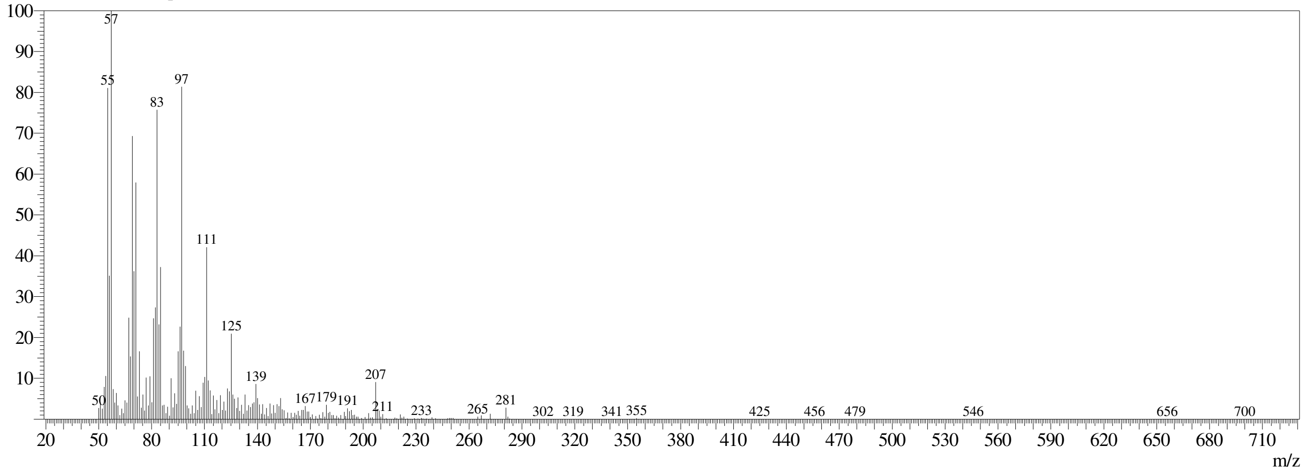

Line#:21 R.Time:25.4(Scan#:6419)
MassPeaks:231
RawMode:Averaged 25.3-25.4(6405-6434) BasePeak:57(57437)
BG Mode:None Group 1 - Event 1

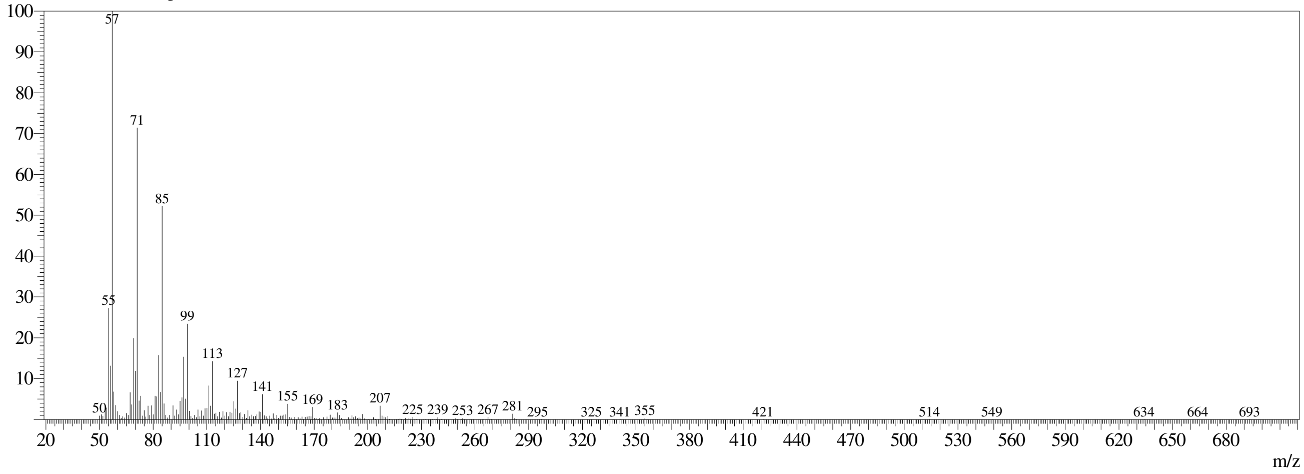

Line#:22 R.Time:26.6(Scan#:6776)
MassPeaks:240
RawMode:Averaged 26.5-26.6(6759-6791) BasePeak:69(116492)
BG Mode:None Group 1 - Event 1

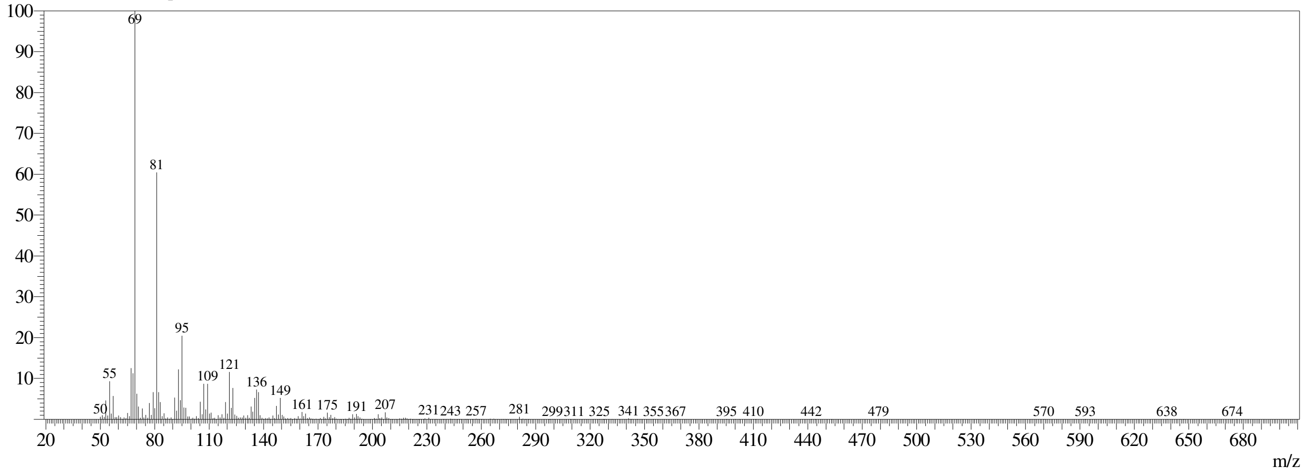

DEPTT. OF BOTANICAL & ENVIRONMENTAL SCIENCES, G.N.D.U. AMRITSAR

Line#:23 R.Time:27.5(Scan#:7062)
MassPeaks:211
RawMode:Averaged 27.5-27.6(7045-7080) BasePeak:57(20267)
BG Mode:None Group 1 - Event 1

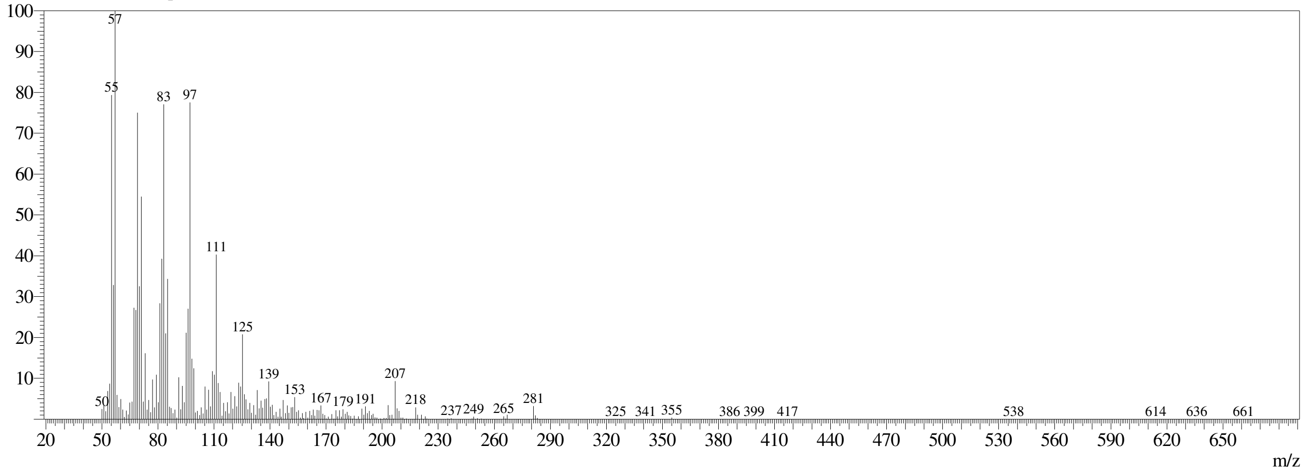

Line#:24 R.Time:27.8(Scan#:7131)
MassPeaks:226
RawMode:Averaged 27.7-27.8(7116-7146) BasePeak:57(52141)
BG Mode:None Group 1 - Event 1

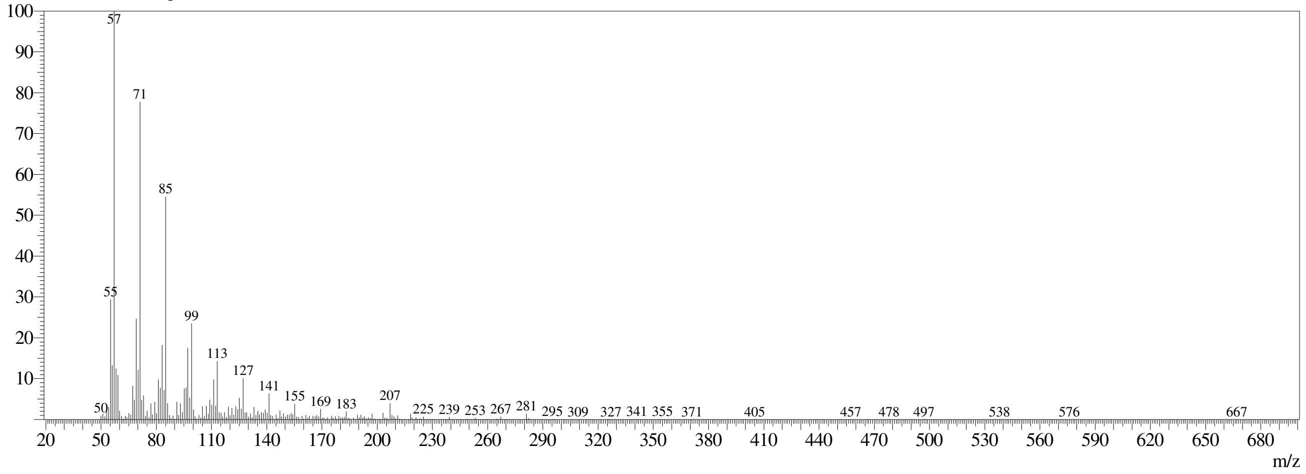

Line#:25 R.Time:31.2(Scan#:8148)
MassPeaks:290
RawMode:Averaged 31.0-31.3(8111-8187) BasePeak:165(56546)
BG Mode:None Group 1 - Event 1

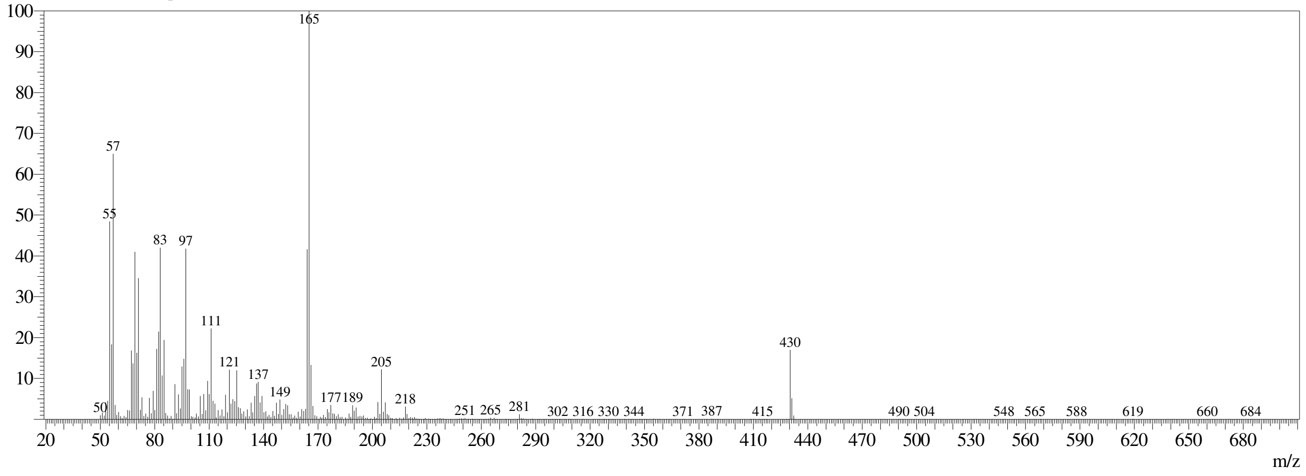

DEPTT. OF BOTANICAL & ENVIRONMENTAL SCIENCES,
G.N.D.U.
AMRITSAR

Line#:26 R.Time:33.7(Scan#:8920)
MassPeaks:361
RawMode:Averaged 33.5-33.8(8836-8940) BasePeak:137(156045)
BG Mode:None Group 1 - Event 1

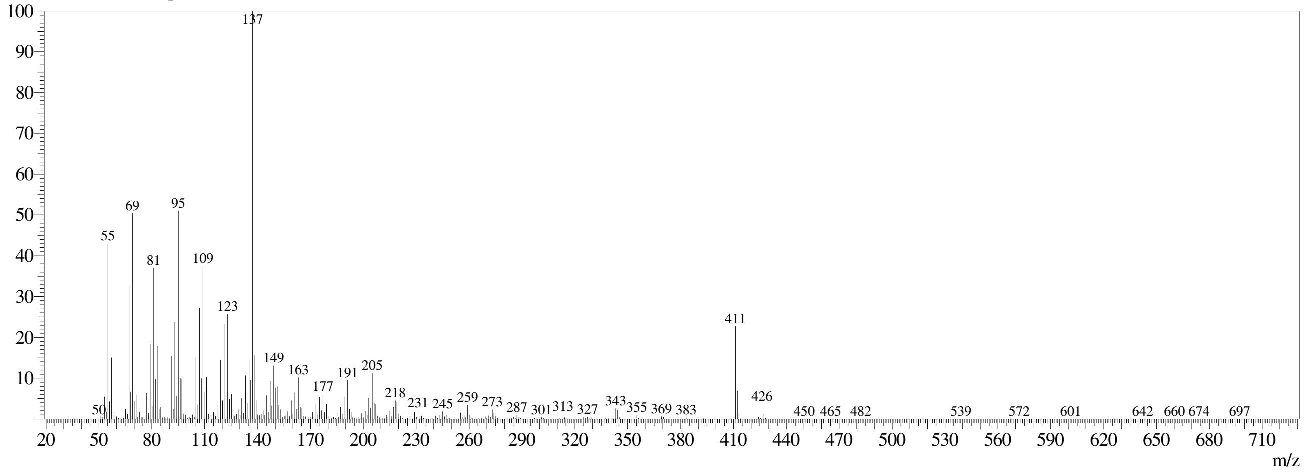

Line#:27 R.Time:35.2(Scan#:9361)
MassPeaks:348
RawMode:Averaged 35.1-35.3(9323-9389) BasePeak:95(34893)
BG Mode:None Group 1 - Event 1

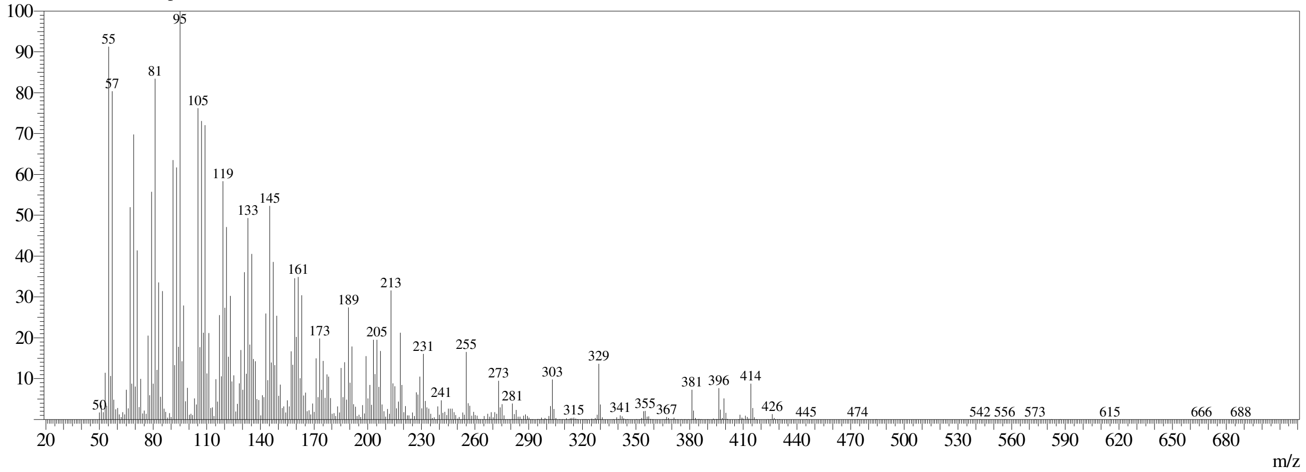

Line#:28 R.Time:35.5(Scan#:9442)
MassPeaks:347
RawMode:Averaged 35.3-35.5(9389-9465) BasePeak:218(210860)
BG Mode:None Group 1 - Event 1

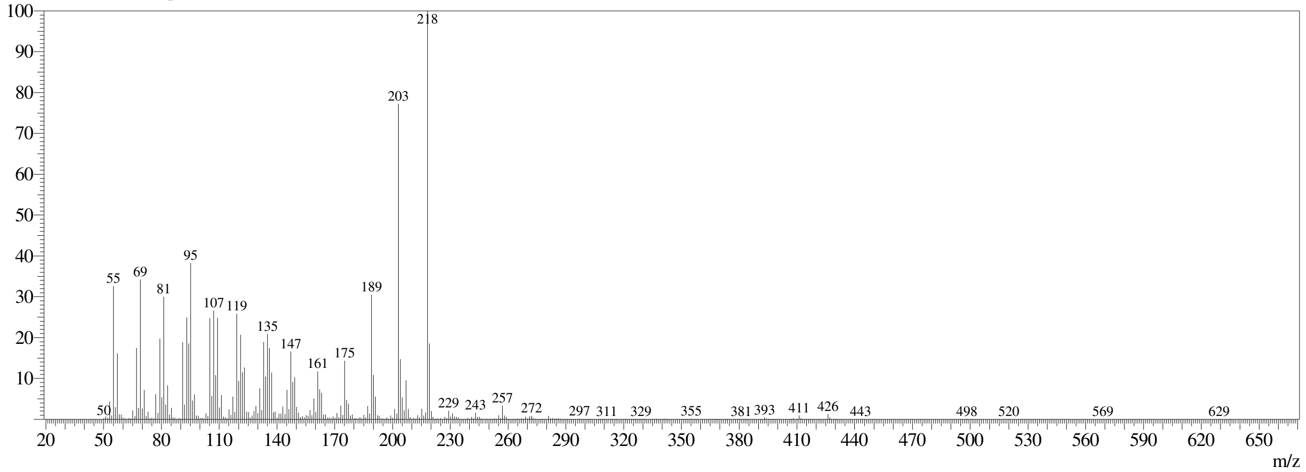

DEPTT. OF BOTANICAL & ENVIRONMENTAL SCIENCES, G.N.D.U. AMRITSAR

Line#:29 R.Time:36.3(Scan#:9700)
MassPeaks:415
RawMode:Averaged 36.0-36.4(9588-9727) BasePeak:218(244782)
BG Mode:None Group 1 - Event 1

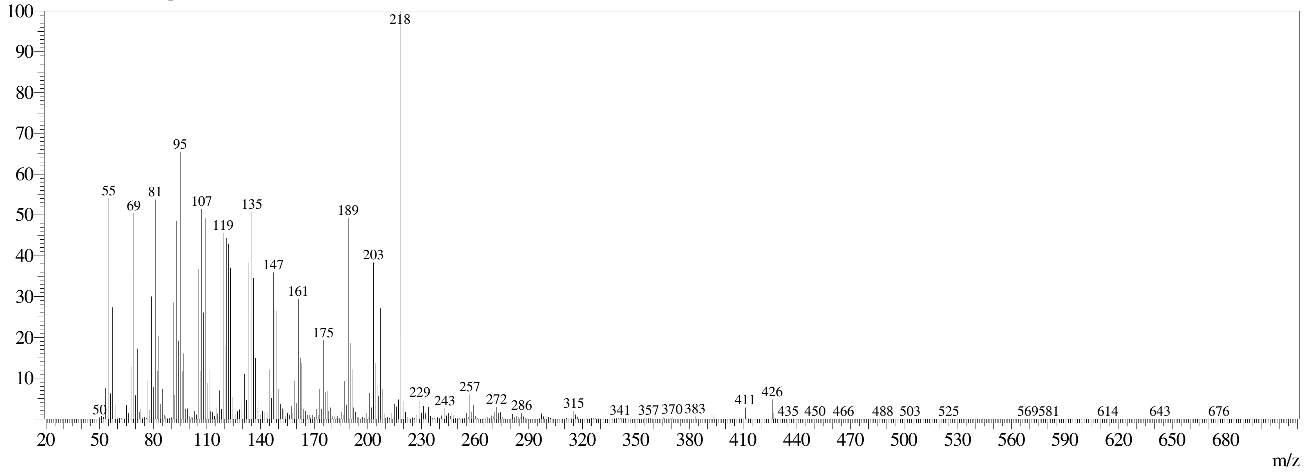

Line#:30 R.Time:37.2(Scan#:9965)
MassPeaks:343
RawMode:Averaged 37.1-37.3(9938-9994) BasePeak:69(43750)
BG Mode:None Group 1 - Event 1

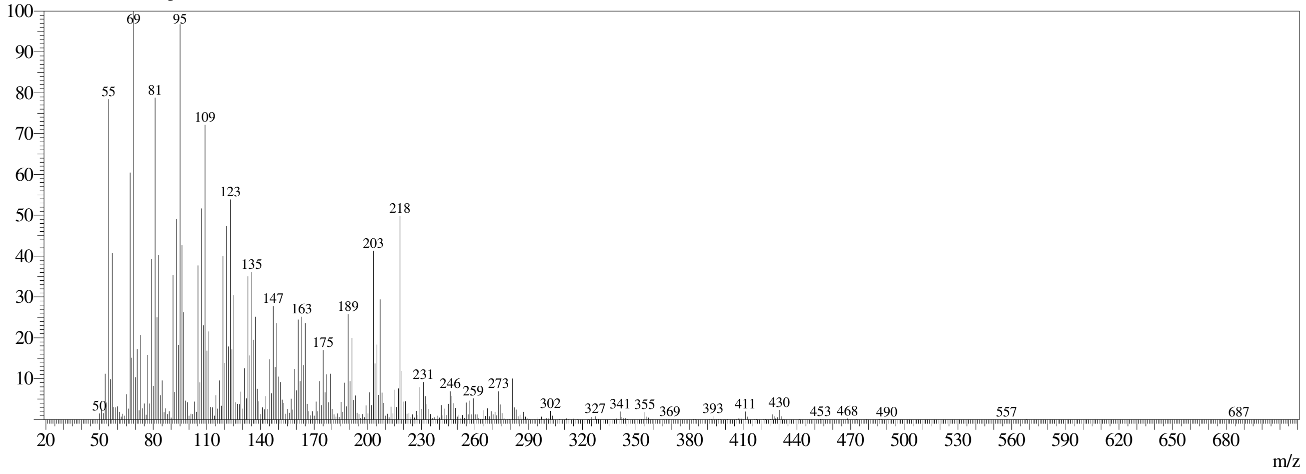

Line#:31 R.Time:38.0(Scan#:10199)
MassPeaks:413
RawMode:Averaged 37.7-38.0(10107-10211) BasePeak:69(259452)
BG Mode:None Group 1 - Event 1

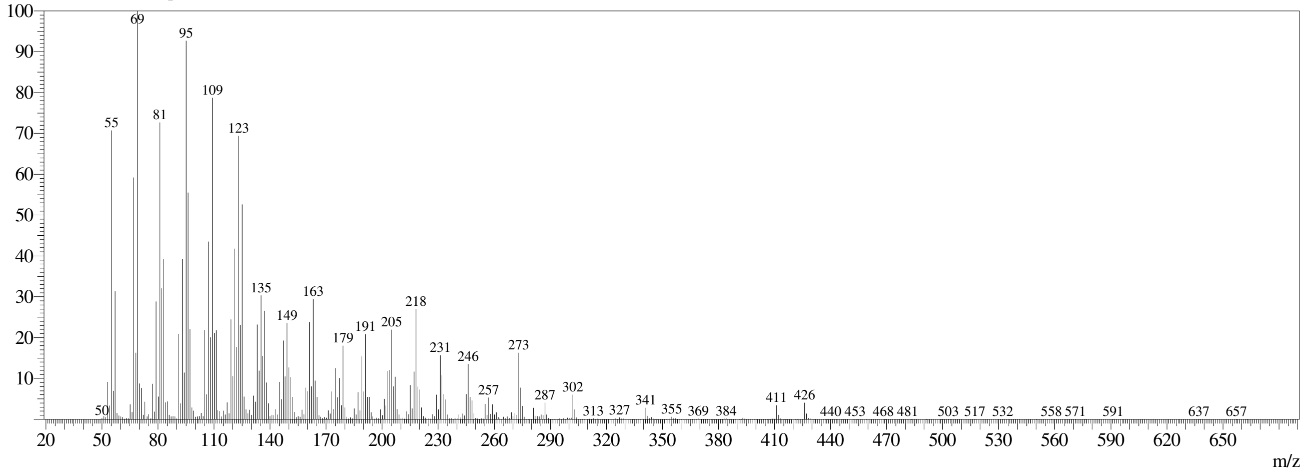

DEPTT. OF BOTANICAL & ENVIRONMENTAL SCIENCES, G.N.D.U. AMRITSAR

Line#:32 R.Time:39.9(Scan#:10777)
MassPeaks:399
RawMode:Averaged 39.8-40.0(10753-10810) BasePeak:203(79236)
BG Mode:None Group 1 - Event 1

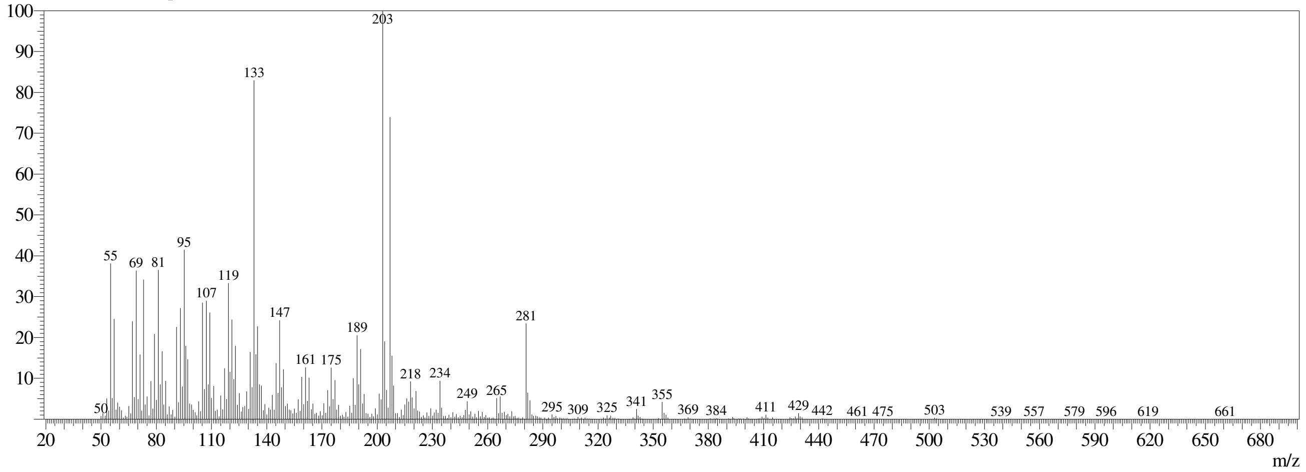

Mass Table
Line#:1 R.Time:8.1(Scan#:1223)
MassPeaks:168
RawMode:Averaged 8.0-8.2(1204-1259) BasePeak:55(29027)
BG Mode:None Group 1 - Event 1

#	m/z	Abs. Int.	Rel. Int.
1	50.10	950	3.27
2	51.10	1227	4.23
3	52.15	921	3.17
4	53.10	3523	12.14
5	54.10	4531	15.61
6	55.10	29027	100.00
7	56.10	23190	79.89
8	57.10	16796	57.86
9	58.10	1258	4.33
10	59.15	535	1.84
11	60.10	3595	12.39
12	61.05	1099	3.79
13	62.10	256	0.88
14	63.10	530	1.83
15	64.10	290	1.00
16	65.05	1195	4.12
17	66.10	1020	3.51
18	67.10	5697	19.63
19	68.10	4144	14.28
20	69.10	22125	76.22
21	70.10	18932	65.22
22	71.10	6220	21.43
23	72.10	554	1.91
24	73.05	1804	6.21
25	74.10	578	1.99
26	75.00	500	1.72
27	76.00	484	1.67
28	77.05	2449	8.44
29	78.05	753	2.59
30	79.05	1822	6.28
31	80.05	412	1.42
32	81.10	2403	8.28
33	82.10	4128	14.22
34	83.05	14356	49.46
35	84.05	8614	29.68
36	85.10	3194	11.00
37	86.10	509	1.75
38	87.00	266	0.92
39	88.10	156	0.54
40	89.10	189	0.65
41	90.10	5	0.02
42	91.10	1297	4.47
43	92.10	364	1.25
44	93.10	527	1.82
45	94.05	766	2.64
46	95.10	970	3.34
47	96.10	1881	6.48

#	m/z	Abs. Int.	Rel. Int.
48	97.10	8959	30.86
49	98.10	3618	12.46
50	99.10	443	1.53
51	100.10	20	0.07
52	101.10	415	1.43
53	102.00	67	0.23
54	103.10	365	1.26
55	104.10	215	0.74
56	105.00	665	2.29
57	106.10	152	0.52
58	107.10	295	1.02
59	108.10	201	0.69
60	109.10	491	1.69
61	110.10	749	2.58
62	111.10	2685	9.25
63	112.15	1090	3.76
64	113.20	178	0.61
65	114.10	3	0.01
66	115.00	399	1.37
67	116.00	67	0.23
68	117.20	423	1.46
69	118.00	52	0.18
70	119.00	351	1.21
71	120.10	97	0.33
72	121.10	217	0.75
73	122.00	141	0.49
74	123.00	101	0.35
75	124.10	385	1.33
76	125.15	952	3.28
77	126.15	637	2.19
78	127.10	108	0.37
79	128.00	296	1.02
80	129.00	259	0.89
81	130.10	78	0.27
82	131.10	211	0.73
83	132.00	53	0.18
84	133.05	500	1.72
85	134.10	112	0.39
86	135.10	239	0.82
87	136.10	19	0.07
88	137.00	24	0.08
89	138.10	50	0.17
90	139.15	214	0.74
91	140.15	548	1.89
92	141.10	138	0.48
93	142.10	302	1.04
94	143.10	52	0.18

#	m/z	Abs. Int.	Rel. Int.
95	144.20	3	0.01
96	145.20	75	0.26
97	146.10	3	0.01
98	147.10	128	0.44
99	148.20	25	0.09
100	149.10	7	0.02
101	150.00	8	0.03
102	151.10	46	0.16
103	152.10	31	0.11
104	153.10	7	0.02
105	155.10	7	0.02
106	157.00	7	0.02
107	159.10	3	0.01
108	163.10	7	0.02
109	165.10	23	0.08
110	166.20	7	0.02
111	168.20	194	0.67
112	169.10	36	0.12
113	174.20	3	0.01
114	177.00	30	0.10
115	179.10	7	0.02
116	182.20	3	0.01
117	183.00	7	0.02
118	185.20	12	0.04
119	191.10	101	0.35
120	192.10	8	0.03
121	193.00	63	0.22
122	194.20	3	0.01
123	201.00	3	0.01
124	203.10	7	0.02
125	205.10	7	0.02
126	206.10	3	0.01
127	206.95	764	2.63
128	208.20	144	0.50
129	209.10	56	0.19
130	217.00	3	0.01
131	219.10	3	0.01
132	222.00	3	0.01
133	224.00	7	0.02
134	229.00	3	0.01
135	231.10	3	0.01
136	238.00	3	0.01
137	240.10	3	0.01
138	249.00	3	0.01
139	250.90	4	0.01
140	253.10	3	0.01
141	265.10	24	0.08

#	m/z	Abs. Int.	Rel. Int.
142	267.10	166	0.57
143	268.10	7	0.02
144	281.20	251	0.86
145	282.10	11	0.04
146	285.10	3	0.01
147	321.00	3	0.01
148	325.20	3	0.01
149	334.10	3	0.01
150	351.10	3	0.01

#	m/z	Abs. Int.	Rel. Int.
151	355.00	16	0.06
152	356.10	12	0.04
153	368.00	3	0.01
154	390.10	4	0.01
155	398.10	3	0.01
156	426.20	3	0.01
157	463.00	3	0.01
158	558.10	4	0.01
159	566.00	3	0.01

#	m/z	Abs. Int.	Rel. Int.
160	576.20	3	0.01
161	586.00	3	0.01
162	610.00	7	0.02
163	624.10	3	0.01
164	650.20	3	0.01
165	651.00	3	0.01
166	653.00	3	0.01
167	672.00	3	0.01
168	675.10	3	0.01

Line#:2 R.Time:12.0(Scan#:2413)
MassPeaks:161
RawMode:Averaged 12.0-12.1(2401-2427) BasePeak:55(83947)
BG Mode:None Group 1 - Event 1

#	m/z	Abs. Int.	Rel. Int.
1	50.10	1071	1.28
2	51.10	1704	2.03
3	52.15	1155	1.38
4	53.10	8001	9.53
5	54.10	12821	15.27
6	55.10	83947	100.00
7	56.10	57307	68.27
8	57.10	65163	77.62
9	58.10	3286	3.91
10	59.05	725	0.86
11	60.10	3030	3.61
12	61.05	1619	1.93
13	62.10	389	0.46
14	63.10	821	0.98
15	64.10	586	0.70
16	65.10	2199	2.62
17	66.10	2065	2.46
18	67.10	16222	19.32
19	68.10	12191	14.52
20	69.10	64669	77.04
21	70.10	52693	62.77
22	71.10	23321	27.78
23	72.10	1505	1.79
24	73.05	1921	2.29
25	74.10	555	0.66
26	75.10	1051	1.25
27	76.10	496	0.59
28	77.05	2954	3.52
29	78.05	895	1.07
30	79.05	2893	3.45
31	80.05	824	0.98
32	81.10	7565	9.01
33	82.10	16423	19.56
34	83.10	57731	68.77
35	84.10	27806	33.12
36	85.10	10768	12.83
37	86.10	837	1.00
38	87.10	338	0.40
39	88.10	315	0.38
40	89.10	496	0.59
41	90.10	138	0.16
42	91.05	1711	2.04
43	92.00	548	0.65
44	93.05	723	0.86
45	94.05	609	0.73
46	95.10	2656	3.16
47	96.10	6683	7.96
48	97.10	39209	46.71
49	98.10	11916	14.19
50	99.10	1650	1.97
51	100.10	200	0.24
52	101.10	437	0.52
53	102.10	180	0.21
54	103.10	487	0.58

#	m/z	Abs. Int.	Rel. Int.
55	104.10	113	0.13
56	105.10	669	0.80
57	106.10	271	0.32
58	107.00	552	0.66
59	108.10	203	0.24
60	109.10	885	1.05
61	110.10	3560	4.24
62	111.10	14194	16.91
63	112.10	5244	6.25
64	113.10	798	0.95
65	114.10	126	0.15
66	115.10	608	0.72
67	116.10	390	0.46
68	117.10	564	0.67
69	118.10	408	0.49
70	119.10	459	0.55
71	120.10	318	0.38
72	121.10	445	0.53
73	122.10	280	0.33
74	123.10	770	0.92
75	124.15	1365	1.63
76	125.15	5369	6.40
77	126.15	2901	3.46
78	127.10	443	0.53
79	128.00	371	0.44
80	129.10	809	0.96
81	130.10	247	0.29
82	131.10	430	0.51
83	132.10	118	0.14
84	133.10	422	0.50
85	134.10	59	0.07
86	135.10	211	0.25
87	136.10	332	0.40
88	137.10	137	0.16
89	138.10	528	0.63
90	139.15	1374	1.64
91	140.15	1419	1.69
92	141.10	450	0.54
93	142.10	231	0.28
94	143.10	160	0.19
95	144.10	16	0.02
96	145.10	441	0.53
97	146.10	252	0.30
98	147.10	318	0.38
99	148.00	23	0.03
100	149.10	118	0.14
101	150.10	23	0.03
102	151.10	519	0.62
103	152.15	405	0.48
104	153.15	599	0.71
105	154.15	881	1.05
106	155.20	204	0.24
107	156.10	166	0.20
108	157.10	45	0.05

#	m/z	Abs. Int.	Rel. Int.
109	158.10	36	0.04
110	159.10	103	0.12
111	161.10	44	0.05
112	164.10	18	0.02
113	165.20	115	0.14
114	166.10	251	0.30
115	167.20	195	0.23
116	168.20	915	1.09
117	169.20	116	0.14
118	170.20	35	0.04
119	173.20	48	0.06
120	174.10	15	0.02
121	178.20	8	0.01
122	179.20	26	0.03
123	181.20	15	0.02
124	189.10	7	0.01
125	191.10	35	0.04
126	193.20	60	0.07
127	194.10	7	0.01
128	196.15	363	0.43
129	197.20	48	0.06
130	199.10	15	0.02
131	207.20	465	0.55
132	208.00	83	0.10
133	209.10	17	0.02
134	210.10	7	0.01
135	229.10	16	0.02
136	232.00	18	0.02
137	237.20	7	0.01
138	253.20	7	0.01
139	258.20	16	0.02
140	281.10	155	0.18
141	288.10	7	0.01
142	325.10	7	0.01
143	329.10	7	0.01
144	341.10	50	0.06
145	342.00	7	0.01
146	355.20	7	0.01
147	360.00	7	0.01
148	371.20	7	0.01
149	444.10	7	0.01
150	474.10	24	0.03
151	529.10	7	0.01
152	531.10	14	0.02
153	540.20	17	0.02
154	578.20	7	0.01
155	597.10	9	0.01
156	600.20	7	0.01
157	615.10	7	0.01
158	623.20	7	0.01
159	646.10	7	0.01
160	652.10	7	0.01
161	670.10	7	0.01

Line#:3 R.Time:12.2(Scan#:2464)
MassPeaks:155
RawMode:Averaged 12.2-12.2(2452-2472) BasePeak:57(52899)
BG Mode:None Group 1 - Event 1

#	m/z	Abs. Int.	Rel. Int.
1	50.15	1219	2.30
2	51.15	1889	3.57
3	52.05	1224	2.31
4	53.10	2792	5.28
5	54.15	1639	3.10

#	m/z	Abs. Int.	Rel. Int.
6	55.10	11682	22.08
7	56.10	8550	16.16
8	57.10	52899	100.00
9	58.15	2978	5.63
10	59.00	823	1.56

#	m/z	Abs. Int.	Rel. Int.
11	60.10	3023	5.71
12	61.10	1612	3.05
13	62.10	487	0.92
14	63.10	1147	2.17
15	64.05	714	1.35

DEPTT. OF BOTANICAL & ENVIRONMENTAL SCIENCES, G.N.D.U. AMRITSAR

#	m/z	Abs. Int.	Rel. Int.
16	65.05	1406	2.66
17	66.10	740	1.40
18	67.05	2251	4.26
19	68.10	1399	2.64
20	69.10	5578	10.54
21	70.10	6779	12.81
22	71.10	30751	58.13
23	72.10	2181	4.12
24	73.05	2571	4.86
25	74.10	697	1.32
26	75.05	1221	2.31
27	76.05	669	1.26
28	77.05	5553	10.50
29	78.05	1447	2.74
30	79.10	2503	4.73
31	80.10	557	1.05
32	81.10	1745	3.30
33	82.10	1611	3.05
34	83.10	3362	6.36
35	84.10	3201	6.05
36	85.10	19071	36.05
37	86.10	1545	2.92
38	87.10	488	0.92
39	88.10	359	0.68
40	89.10	575	1.09
41	90.10	147	0.28
42	91.10	1964	3.71
43	92.10	616	1.16
44	93.10	642	1.21
45	94.10	567	1.07
46	95.10	1016	1.92
47	96.10	835	1.58
48	97.10	2195	4.15
49	98.15	1925	3.64
50	99.15	3893	7.36
51	100.10	639	1.21
52	101.10	516	0.98
53	102.10	268	0.51
54	103.10	569	1.08
55	104.00	230	0.43
56	105.10	856	1.62
57	106.10	306	0.58
58	107.05	9554	18.06
59	108.10	1265	2.39
60	109.10	614	1.16
61	110.00	1015	1.92
62	111.10	852	1.61
63	112.15	1248	2.36
64	113.15	1621	3.06
65	114.10	257	0.49
66	115.05	916	1.73
67	116.10	354	0.67
68	117.00	594	1.12
69	118.10	235	0.44
70	119.00	578	1.09
71	120.10	369	0.70
72	121.10	414	0.78
73	122.10	260	0.49
74	123.10	508	0.96
75	124.10	341	0.64
76	125.10	501	0.95
77	126.15	1001	1.89
78	127.15	1093	2.07
79	128.10	663	1.25
80	129.05	1027	1.94
81	130.00	326	0.62
82	131.00	388	0.73
83	132.10	65	0.12
84	133.10	521	0.98
85	134.10	181	0.34
86	135.10	317	0.60
87	136.00	311	0.59
88	137.10	231	0.44
89	138.05	1726	3.26
90	139.10	317	0.60
91	140.15	724	1.37
92	141.10	1796	3.40
93	142.10	363	0.69
94	143.10	483	0.91
95	144.10	117	0.22
96	145.10	360	0.68
97	146.10	121	0.23
98	147.10	359	0.68
99	148.10	42	0.08
100	149.10	109	0.21
101	150.10	193	0.36
102	151.10	587	1.11
103	152.10	321	0.61
104	153.10	211	0.40
105	154.10	370	0.70
106	155.20	574	1.09
107	156.10	857	1.62
108	157.15	392	0.74
109	158.10	84	0.16
110	159.10	87	0.16
111	160.20	9	0.02
112	163.10	10	0.02
113	165.10	221	0.42
114	166.10	244	0.46
115	167.20	43	0.08
116	168.10	86	0.16
117	169.20	154	0.29
118	173.10	209	0.40
119	174.20	53	0.10
120	177.10	20	0.04
121	178.10	20	0.04
122	179.10	59	0.11
123	181.10	11	0.02
124	186.10	9	0.02
125	187.10	11	0.02
126	189.20	203	0.38
127	191.00	125	0.24
128	193.10	157	0.30
129	195.10	9	0.02
130	198.20	250	0.47
131	199.10	11	0.02
132	205.10	9	0.02
133	207.10	497	0.94
134	208.10	22	0.04
135	213.20	30	0.06
136	223.10	10	0.02
137	249.10	9	0.02
138	253.10	9	0.02
139	256.00	10	0.02
140	265.10	21	0.04
141	267.10	233	0.44
142	281.10	87	0.16
143	293.10	9	0.02
144	327.10	19	0.04
145	341.10	21	0.04
146	355.10	10	0.02
147	356.10	40	0.08
148	433.10	10	0.02
149	463.10	10	0.02
150	466.00	10	0.02
151	469.10	10	0.02
152	490.10	9	0.02
153	493.10	9	0.02
154	522.10	10	0.02
155	621.10	9	0.02

Line#:4 R.Time:13.6(Scan#:2877)
MassPeaks:159
RawMode:Averaged 13.6-13.6(2866-2889) BasePeak:191(169271)
BG Mode:None Group 1 - Event 1

#	m/z	Abs. Int.	Rel. Int.
1	50.10	1446	0.85
2	51.10	6467	3.82
3	52.10	3232	1.91
4	53.10	5397	3.19
5	54.10	1165	0.69
6	55.10	10398	6.14
7	56.15	2185	1.29
8	57.10	120737	71.33
9	58.10	6874	4.06
10	59.05	1630	0.96
11	60.10	8300	4.90
12	61.05	1793	1.06
13	62.05	752	0.44
14	63.05	3336	1.97
15	64.10	3549	2.10
16	65.05	7548	4.46
17	66.10	4839	2.86
18	67.05	3561	2.10
19	68.05	907	0.54
20	69.10	3489	2.06
21	70.05	2118	1.25
22	71.10	2242	1.32
23	72.15	2775	1.64
24	73.10	11855	7.00
25	74.10	16675	9.85
26	75.05	3187	1.88
27	76.10	1419	0.84
28	77.05	13351	7.89
29	78.05	3596	2.12
30	79.10	7581	4.48
31	80.05	1394	0.82
32	81.05	2172	1.28
33	82.05	1027	0.61
34	83.10	1887	1.11
35	84.10	778	0.46
36	85.05	1483	0.88
37	86.00	526	0.31
38	87.15	1214	0.72
39	88.05	7317	4.32
40	89.00	2983	1.76
41	90.15	810	0.48
42	91.05	18614	11.00
43	92.05	2291	1.35
44	93.10	2287	1.35
45	94.10	1257	0.74
46	95.05	2852	1.68
47	96.10	734	0.43
48	97.10	1251	0.74
49	98.10	533	0.31
50	99.10	649	0.38
51	100.10	310	0.18
52	101.05	1348	0.80
53	102.05	1438	0.85
54	103.05	4391	2.59
55	104.10	1212	0.72
56	105.05	8419	4.97
57	106.05	1537	0.91
58	107.05	14092	8.33
59	108.05	1609	0.95
60	109.00	1109	0.66
61	110.05	825	0.49
62	111.10	678	0.40
63	112.10	277	0.16
64	113.00	406	0.24
65	114.05	294	0.17
66	115.05	12602	7.44
67	116.05	5408	3.19
68	117.05	6012	3.55
69	118.05	1278	0.76
70	119.05	6402	3.78
71	120.05	1650	0.97
72	121.05	6236	3.68
73	122.05	900	0.53
74	123.05	1860	1.10
75	124.10	408	0.24
76	125.10	397	0.23
77	126.05	599	0.35
78	127.10	2638	1.56
79	128.05	6956	4.11
80	129.05	4842	2.86
81	130.05	1103	0.65

DEPTT. OF BOTANICAL & ENVIRONMENTAL SCIENCES, G.N.D.U. AMRITSAR

#	m/z	Abs. Int.	Rel. Int.
82	131.05	3804	2.25
83	132.10	1463	0.86
84	133.05	6776	4.00
85	134.05	1994	1.18
86	135.10	6887	4.07
87	136.10	1002	0.59
88	137.10	533	0.31
89	138.10	338	0.20
90	139.05	564	0.33
91	140.10	172	0.10
92	141.05	2301	1.36
93	142.05	1473	0.87
94	143.05	2173	1.28
95	144.05	864	0.51
96	145.10	2228	1.32
97	146.10	1044	0.62
98	147.10	8364	4.94
99	148.10	2118	1.25
100	149.10	2555	1.51
101	150.10	390	0.23
102	151.10	773	0.46
103	152.10	583	0.34
104	153.10	581	0.34
105	154.10	258	0.15
106	155.10	1268	0.75
107	156.10	240	0.14

#	m/z	Abs. Int.	Rel. Int.
108	157.05	677	0.40
109	158.10	518	0.31
110	159.05	824	0.49
111	160.10	533	0.31
112	161.10	4577	2.70
113	162.15	714	0.42
114	163.10	17519	10.35
115	164.10	2214	1.31
116	165.05	420	0.25
117	166.00	444	0.26
118	167.10	295	0.17
119	168.10	166	0.10
120	169.10	198	0.12
121	170.15	810	0.48
122	171.10	233	0.14
123	173.10	227	0.13
124	174.10	280	0.17
125	175.10	9780	5.78
126	176.10	1541	0.91
127	177.10	352	0.21
128	178.10	38	0.02
129	179.10	34	0.02
130	180.10	44	0.03
131	182.10	97	0.06
132	184.00	8	0.00
133	185.10	44	0.03

#	m/z	Abs. Int.	Rel. Int.
134	186.10	8	0.00
135	187.10	37	0.02
136	188.10	17	0.01
137	189.05	637	0.38
138	190.15	767	0.45
139	191.10	169271	100.00
140	192.10	23483	13.87
141	193.05	1940	1.15
142	194.10	223	0.13
143	201.10	9	0.01
144	202.10	8	0.00
145	204.10	18	0.01
146	205.10	100	0.06
147	206.10	27694	16.36
148	207.10	4741	2.80
149	208.10	499	0.29
150	209.10	57	0.03
151	236.10	8	0.00
152	251.10	9	0.01
153	268.10	9	0.01
154	281.10	179	0.11
155	313.10	8	0.00
156	342.10	9	0.01
157	356.10	8	0.00
158	534.10	8	0.00
159	634.10	8	0.00

Line#:5 R.Time:14.8(Scan#:3249)
MassPeaks:183
RawMode:Averaged 14.8-14.9(3237-3261) BasePeak:55(102093)
BG Mode:None Group 1 - Event 1

#	m/z	Abs. Int.	Rel. Int.
1	50.10	1584	1.55
2	51.10	4483	4.39
3	52.15	1489	1.46
4	53.10	9036	8.85
5	54.10	15203	14.89
6	55.15	102093	100.00
7	56.10	64194	62.88
8	57.10	96002	94.03
9	58.10	4461	4.37
10	59.10	1203	1.18
11	60.10	4082	4.00
12	61.05	2063	2.02
13	62.10	596	0.58
14	63.05	1111	1.09
15	64.10	595	0.58
16	65.05	3057	2.99
17	66.10	3268	3.20
18	67.10	21838	21.39
19	68.10	15460	15.14
20	69.10	81595	79.92
21	70.10	59062	57.85
22	71.10	37884	37.11
23	72.10	2435	2.39
24	73.10	3146	3.08
25	74.05	1257	1.23
26	75.05	2299	2.25
27	76.05	954	0.93
28	77.05	7379	7.23
29	78.05	1690	1.66
30	79.05	3671	3.60
31	80.15	1190	1.17
32	81.10	11747	11.51
33	82.10	24285	23.79
34	83.10	76689	75.12
35	84.10	33959	33.26
36	85.10	19333	18.94
37	86.10	1668	1.63
38	87.10	777	0.76
39	88.10	412	0.40
40	89.05	927	0.91
41	90.10	167	0.16
42	91.05	2149	2.10
43	92.10	586	0.57
44	93.10	1127	1.10
45	94.10	1106	1.08
46	95.10	5132	5.03
47	96.10	12217	11.97
48	97.10	66103	64.75

#	m/z	Abs. Int.	Rel. Int.
49	98.10	17888	17.52
50	99.10	3515	3.44
51	100.10	631	0.62
52	101.10	606	0.59
53	102.10	481	0.47
54	103.10	821	0.80
55	104.00	458	0.45
56	105.05	2314	2.27
57	106.10	546	0.53
58	107.00	1106	1.08
59	108.10	462	0.45
60	109.10	1899	1.86
61	110.10	5466	5.35
62	111.10	27651	27.08
63	112.10	9467	9.27
64	113.15	1634	1.60
65	114.10	227	0.22
66	115.05	1179	1.15
67	116.00	656	0.64
68	117.00	1220	1.19
69	118.00	441	0.43
70	119.05	1056	1.03
71	120.00	396	0.39
72	121.10	793	0.78
73	122.10	560	0.55
74	123.05	10100	9.89
75	124.15	3192	3.13
76	125.15	8896	8.71
77	126.15	4733	4.64
78	127.10	1015	0.99
79	128.10	417	0.41
80	129.10	1338	1.31
81	130.00	894	0.88
82	131.10	962	0.94
83	132.10	366	0.36
84	133.00	1118	1.10
85	134.00	343	0.34
86	135.00	598	0.59
87	136.10	495	0.48
88	137.10	578	0.57
89	138.15	1622	1.59
90	139.15	2790	2.73
91	140.15	2326	2.28
92	141.15	739	0.72
93	142.10	183	0.18
94	143.10	470	0.46
95	144.10	160	0.16
96	145.10	408	0.40

#	m/z	Abs. Int.	Rel. Int.
97	146.10	228	0.22
98	147.10	563	0.55
99	148.10	253	0.25
100	149.00	913	0.89
101	150.00	363	0.36
102	151.10	983	0.96
103	152.10	2848	2.79
104	153.15	1537	1.51
105	154.15	1518	1.49
106	155.20	350	0.34
107	156.20	65	0.06
108	157.20	226	0.22
109	158.00	56	0.05
110	159.20	210	0.21
111	160.20	55	0.05
112	161.10	210	0.21
113	162.20	85	0.08
114	163.10	281	0.28
115	164.20	93	0.09
116	165.10	470	0.46
117	166.15	674	0.66
118	167.15	708	0.69
119	168.15	879	0.86
120	169.10	471	0.46
121	170.10	58	0.06
122	171.20	54	0.05
123	172.10	16	0.02
124	173.20	103	0.10
125	174.10	29	0.03
126	175.10	146	0.14
127	177.10	217	0.21
128	178.00	85	0.08
129	179.05	1947	1.91
130	180.00	486	0.48
131	181.05	506	0.50
132	182.15	469	0.46
133	183.10	28	0.03
134	184.00	98	0.10
135	185.00	44	0.04
136	188.00	58	0.06
137	189.00	127	0.12
138	190.10	8	0.01
139	191.00	143	0.14
140	192.00	89	0.09
141	193.00	245	0.24
142	194.00	36	0.04
143	195.00	145	0.14
144	196.15	749	0.73

#	m/z	Abs. Int.	Rel. Int.	#	m/z	Abs. Int.	Rel. Int.	#	m/z	Abs. Int.	Rel. Int.
145	197.10	150	0.15	158	224.15	417	0.41	171	282.90	64	0.06
146	199.10	73	0.07	159	225.20	60	0.06	172	291.00	8	0.01
147	202.10	20	0.02	160	236.00	34	0.03	173	314.00	8	0.01
148	207.00	527	0.52	161	237.00	47	0.05	174	330.00	8	0.01
149	208.10	296	0.29	162	239.00	88	0.09	175	335.00	8	0.01
150	209.10	135	0.13	163	252.95	2841	2.78	176	340.90	34	0.03
151	211.00	36	0.04	164	253.95	627	0.61	177	342.00	8	0.01
152	217.00	8	0.01	165	254.90	272	0.27	178	514.00	9	0.01
153	218.10	17	0.02	166	265.00	8	0.01	179	535.00	8	0.01
154	219.10	8	0.01	167	266.90	172	0.17	180	552.00	8	0.01
155	220.10	16	0.02	168	269.00	20	0.02	181	575.00	8	0.01
156	222.00	8	0.01	169	281.00	174	0.17	182	620.00	17	0.02
157	223.00	85	0.08	170	281.90	405	0.40	183	679.00	9	0.01

Line#:6 R.Time:16.7(Scan#:3821)
MassPeaks:189
RawMode:Averaged 16.7-16.8(3808-3836) BasePeak:57(17126)
BG Mode:None Group 1 - Event 1

#	m/z	Abs. Int.	Rel. Int.	#	m/z	Abs. Int.	Rel. Int.	#	m/z	Abs. Int.	Rel. Int.
1	50.05	853	4.98	62	111.10	1752	10.23	123	173.10	99	0.58
2	51.10	1391	8.12	63	112.15	677	3.95	124	174.10	8	0.05
3	52.10	865	5.05	64	113.10	1009	5.89	125	175.10	84	0.49
4	53.10	2214	12.93	65	114.10	190	1.11	126	176.10	72	0.42
5	54.15	1573	9.18	66	115.10	2583	15.08	127	177.10	487	2.84
6	55.10	14958	87.34	67	116.10	914	5.34	128	178.10	177	1.03
7	56.10	4175	24.38	68	117.10	819	4.78	129	179.05	1184	6.91
8	57.10	17126	100.00	69	118.10	302	1.76	130	180.00	320	1.87
9	58.10	1639	9.57	70	119.10	899	5.25	131	181.10	307	1.79
10	59.15	1375	8.03	71	120.10	351	2.05	132	182.00	24	0.14
11	60.05	14972	87.42	72	121.10	726	4.24	133	183.10	228	1.33
12	61.05	3912	22.84	73	122.10	380	2.22	134	184.00	22	0.13
13	62.10	515	3.01	74	123.05	1709	9.98	135	185.15	2122	12.39
14	63.05	854	4.99	75	124.10	615	3.59	136	186.20	241	1.41
15	64.10	407	2.38	76	125.10	849	4.96	137	187.20	29	0.17
16	65.05	1197	6.99	77	126.15	720	4.20	138	189.00	146	0.85
17	66.05	702	4.10	78	127.10	653	3.81	139	190.10	21	0.12
18	67.10	3281	19.16	79	128.10	416	2.43	140	191.10	290	1.69
19	68.10	1894	11.06	80	129.10	5492	32.07	141	192.10	525	3.07
20	69.10	8754	51.12	81	130.15	880	5.14	142	193.10	279	1.63
21	70.10	3224	18.83	82	131.10	635	3.71	143	194.10	57	0.33
22	71.10	9018	52.66	83	132.00	172	1.00	144	195.10	7	0.04
23	72.15	874	5.10	84	133.10	627	3.66	145	196.10	37	0.22
24	73.05	17096	99.82	85	134.10	221	1.29	146	197.20	59	0.34
25	74.05	2971	17.35	86	135.10	573	3.35	147	198.10	6	0.04
26	75.05	1361	7.95	87	136.10	665	3.88	148	199.10	452	2.64
27	76.10	583	3.40	88	137.10	518	3.02	149	200.20	83	0.48
28	77.05	2984	17.42	89	138.10	398	2.32	150	202.10	7	0.04
29	78.10	899	5.25	90	139.10	391	2.28	151	205.10	36	0.21
30	79.05	2161	12.62	91	140.10	298	1.74	152	206.10	30	0.18
31	80.10	683	3.99	92	141.10	335	1.96	153	207.10	498	2.91
32	81.10	2852	16.65	93	142.10	181	1.06	154	208.00	195	1.14
33	82.10	2494	14.56	94	143.10	1507	8.80	155	209.10	80	0.47
34	83.10	6694	39.09	95	144.10	258	1.51	156	210.10	70	0.41
35	84.10	2714	15.85	96	145.10	350	2.04	157	211.10	38	0.22
36	85.10	6187	36.13	97	146.10	46	0.27	158	212.00	83	0.48
37	86.15	781	4.56	98	147.10	581	3.39	159	213.10	16	0.09
38	87.10	4492	26.23	99	148.10	161	0.94	160	214.10	7	0.04
39	88.05	815	4.76	100	149.10	679	3.96	161	217.20	82	0.48
40	89.00	814	4.75	101	150.10	292	1.71	162	220.10	99	0.58
41	90.00	242	1.41	102	151.10	692	4.04	163	221.00	40	0.23
42	91.05	2059	12.02	103	152.00	484	2.83	164	223.00	198	1.16
43	92.10	526	3.07	104	153.10	377	2.20	165	224.00	58	0.34
44	93.05	1156	6.75	105	154.10	153	0.89	166	225.10	7	0.04
45	94.05	706	4.12	106	155.10	353	2.06	167	228.10	404	2.36
46	95.05	1988	11.61	107	156.10	30	0.18	168	229.10	60	0.35
47	96.10	1592	9.30	108	157.15	744	4.34	169	231.00	35	0.20
48	97.10	4425	25.84	109	158.10	122	0.71	170	233.10	6	0.04
49	98.10	2034	11.88	110	159.10	225	1.31	171	241.10	7	0.04
50	99.10	1960	11.44	111	160.10	14	0.08	172	248.00	8	0.05
51	100.10	416	2.43	112	161.10	182	1.06	173	258.00	23	0.13
52	101.05	1442	8.42	113	162.00	255	1.49	174	267.10	55	0.32
53	102.10	671	3.92	114	163.10	268	1.56	175	268.10	6	0.04
54	103.05	899	5.25	115	164.10	161	0.94	176	281.10	246	1.44
55	104.10	380	2.22	116	165.10	384	2.24	177	282.10	38	0.22
56	105.10	1180	6.89	117	166.10	552	3.22	178	283.10	15	0.09
57	106.00	363	2.12	118	167.10	324	1.89	179	326.10	14	0.08
58	107.05	1107	6.46	119	168.10	230	1.34	180	341.10	8	0.05
59	108.10	487	2.84	120	169.10	156	0.91	181	348.10	8	0.05
60	109.10	1083	6.32	121	171.15	1096	6.40	182	355.00	7	0.04
61	110.10	1024	5.98	122	172.10	202	1.18	183	398.10	14	0.08

#	m/z	Abs. Int.	Rel. Int.
184	466.10	7	0.04
185	504.10	7	0.04
186	583.10	7	0.04
187	590.10	7	0.04
188	612.10	7	0.04
189	669.10	6	0.04

Line#:7 R.Time:17.2(Scan#:3947)
MassPeaks:191
RawMode:Averaged 17.1-17.2(3934-3958) BasePeak:55(95553)
BG Mode:None Group 1 - Event 1

#	m/z	Abs. Int.	Rel. Int.
1	50.05	1136	1.19
2	51.10	2349	2.46
3	52.15	1393	1.46
4	53.10	7740	8.10
5	54.10	13234	13.85
6	55.10	95553	100.00
7	56.10	52044	54.47
8	57.10	91929	96.21
9	58.10	4529	4.74
10	59.05	739	0.77
11	60.10	1877	1.96
12	61.05	1116	1.17
13	62.00	509	0.53
14	63.05	1340	1.40
15	64.05	871	0.91
16	65.05	3215	3.36
17	66.05	3041	3.18
18	67.10	20978	21.95
19	68.10	14973	15.67
20	69.10	75542	79.06
21	70.10	50633	52.99
22	71.10	41690	43.63
23	72.10	2611	2.73
24	73.10	2387	2.50
25	74.00	610	0.64
26	75.05	1189	1.24
27	76.05	718	0.75
28	77.05	4688	4.91
29	78.10	1579	1.65
30	79.05	4535	4.75
31	80.10	1534	1.61
32	81.10	13475	14.10
33	82.10	25272	26.45
34	83.10	84863	88.81
35	84.10	31861	33.34
36	85.10	24615	25.76
37	86.10	1781	1.86
38	87.10	662	0.69
39	88.10	347	0.36
40	89.00	1272	1.33
41	90.10	468	0.49
42	91.00	3114	3.26
43	92.05	875	0.92
44	93.10	1595	1.67
45	94.10	1209	1.27
46	95.10	6177	6.46
47	96.10	14022	14.67
48	97.10	75401	78.91
49	98.10	18022	18.86
50	99.10	5359	5.61
51	100.10	825	0.86
52	101.10	562	0.59
53	102.10	421	0.44
54	103.10	819	0.86
55	104.10	419	0.44
56	105.10	2053	2.15
57	106.05	687	0.72
58	107.05	1847	1.93
59	108.10	1072	1.12
60	109.10	2858	2.99
61	110.10	7533	7.88
62	111.10	33885	35.46
63	112.15	9789	10.24
64	113.10	2339	2.45
65	114.10	309	0.32
66	115.10	1125	1.18
67	116.10	421	0.44
68	117.10	811	0.85
69	118.10	433	0.45
70	119.00	1238	1.30
71	120.10	602	0.63
72	121.05	943	0.99
73	122.05	1999	2.09
74	123.10	2827	2.96
75	124.15	3551	3.72
76	125.15	12733	13.33
77	126.15	5579	5.84
78	127.15	1427	1.49
79	128.10	513	0.54
80	129.10	855	0.89
81	130.00	199	0.21
82	131.10	509	0.53
83	132.10	233	0.24
84	133.10	891	0.93
85	134.10	385	0.40
86	135.00	1311	1.37
87	136.00	1477	1.55
88	137.10	1164	1.22
89	138.10	2989	3.13
90	139.15	5988	6.27
91	140.15	3020	3.16
92	141.15	987	1.03
93	142.10	259	0.27
94	143.10	730	0.76
95	144.00	155	0.16
96	145.10	382	0.40
97	146.10	77	0.08
98	147.05	771	0.81
99	148.10	236	0.25
100	149.05	841	0.88
101	150.00	399	0.42
102	151.05	1174	1.23
103	152.15	1389	1.45
104	153.10	5642	5.90
105	154.10	6918	7.24
106	155.15	1039	1.09
107	156.20	175	0.18
108	157.10	262	0.27
109	158.10	61	0.06
110	159.10	252	0.26
111	160.20	81	0.08
112	161.10	276	0.29
113	162.10	312	0.33
114	163.10	394	0.41
115	164.10	471	0.49
116	165.10	733	0.77
117	166.15	990	1.04
118	167.20	1378	1.44
119	168.20	957	1.00
120	169.20	343	0.36
121	170.20	17	0.02
122	171.20	181	0.19
123	173.10	122	0.13
124	174.10	9	0.01
125	175.20	186	0.19
126	176.10	84	0.09
127	177.10	427	0.45
128	178.00	229	0.24
129	179.05	1449	1.52
130	180.15	591	0.62
131	181.15	641	0.67
132	182.15	687	0.72
133	183.10	317	0.33
134	184.20	19	0.02
135	185.00	103	0.11
136	186.10	18	0.02
137	187.00	26	0.03
138	189.20	40	0.04
139	190.10	8	0.01
140	191.10	345	0.36
141	192.00	586	0.61
142	193.10	363	0.38
143	194.20	286	0.30
144	195.20	373	0.39
145	196.10	349	0.37
146	197.00	61	0.06
147	198.05	2342	2.45
148	199.00	323	0.34
149	200.00	16	0.02
150	202.00	55	0.06
151	203.00	47	0.05
152	205.00	89	0.09
153	206.10	23	0.02
154	207.00	629	0.66
155	208.00	492	0.51
156	209.00	239	0.25
157	210.00	244	0.26
158	211.00	8	0.01
159	213.00	29	0.03
160	215.00	8	0.01
161	217.00	8	0.01
162	218.00	8	0.01
163	220.00	168	0.18
164	221.00	103	0.11
165	222.00	17	0.02
166	223.00	150	0.16
167	224.15	510	0.53
168	225.10	120	0.13
169	227.00	90	0.09
170	228.00	8	0.01
171	229.00	17	0.02
172	235.00	18	0.02
173	241.10	8	0.01
174	251.00	57	0.06
175	252.10	161	0.17
176	253.20	81	0.08
177	254.00	18	0.02
178	265.10	17	0.02
179	266.00	91	0.10
180	267.00	17	0.02
181	268.00	8	0.01
182	269.10	8	0.01
183	281.10	226	0.24
184	283.10	8	0.01
185	288.00	41	0.04
186	340.00	20	0.02
187	425.00	9	0.01
188	520.00	8	0.01
189	610.00	16	0.02
190	653.00	16	0.02
191	682.10	8	0.01

Line#:8 R.Time:17.6(Scan#:4094)
MassPeaks:186
RawMode:Averaged 17.6-17.7(4085-4107) BasePeak:68(90232)
BG Mode:None Group 1 - Event 1

#	m/z	Abs. Int.	Rel. Int.
1	50.10	1063	1.18
2	51.10	2484	2.75
3	52.10	1847	2.05
4	53.10	12931	14.33
5	54.15	3914	4.34
6	55.10	64624	71.62

DEPTT. OF BOTANICAL & ENVIRONMENTAL SCIENCES, G.N.D.U. AMRITSAR

#	m/z	Abs. Int.	Rel. Int.
7	56.10	19722	21.86
8	57.10	74012	82.02
9	58.10	3663	4.06
10	59.10	671	0.74
11	60.05	1602	1.78
12	61.05	1055	1.17
13	62.10	405	0.45
14	63.05	996	1.10
15	64.00	440	0.49
16	65.05	6248	6.92
17	66.15	2799	3.10
18	67.05	55986	62.05
19	68.10	90232	100.00
20	69.10	63876	70.79
21	70.10	18728	20.76
22	71.10	38163	42.29
23	72.10	2807	3.11
24	73.10	1869	2.07
25	74.10	505	0.56
26	75.00	863	0.96
27	76.00	598	0.66
28	77.05	6605	7.32
29	78.10	1653	1.83
30	79.05	17254	19.12
31	80.10	4954	5.49
32	81.10	52330	57.99
33	82.10	62669	69.45
34	83.10	45359	50.27
35	84.10	6564	7.27
36	85.10	13808	15.30
37	86.10	1129	1.25
38	87.10	461	0.51
39	88.10	278	0.31
40	89.00	792	0.88
41	90.00	244	0.27
42	91.05	4849	5.37
43	92.05	971	1.08
44	93.10	5676	6.29
45	94.10	8189	9.08
46	95.10	70827	78.49
47	96.10	23987	26.58
48	97.10	30902	34.25
49	98.10	4380	4.85
50	99.15	2353	2.61
51	100.10	326	0.36
52	101.10	421	0.47
53	102.10	286	0.32
54	103.10	737	0.82
55	104.10	340	0.38
56	105.10	1542	1.71
57	106.15	479	0.53
58	107.10	2526	2.80
59	108.10	1517	1.68
60	109.10	23450	25.99
61	110.10	9466	10.49
62	111.10	14740	16.34
63	112.10	2525	2.80
64	113.15	1236	1.37
65	114.10	217	0.24
66	115.05	1575	1.75

#	m/z	Abs. Int.	Rel. Int.
67	116.10	499	0.55
68	117.10	719	0.80
69	118.10	267	0.30
70	119.10	872	0.97
71	120.10	434	0.48
72	121.15	1325	1.47
73	122.25	1388	1.54
74	123.15	33199	36.79
75	124.15	13585	15.06
76	125.15	5308	5.88
77	126.15	2324	2.58
78	127.15	1201	1.33
79	128.10	502	0.56
80	129.10	699	0.77
81	130.10	354	0.39
82	131.10	573	0.64
83	132.10	232	0.26
84	133.10	775	0.86
85	134.10	245	0.27
86	135.10	843	0.93
87	136.05	911	1.01
88	137.10	6055	6.71
89	138.15	2891	3.20
90	139.15	1201	1.33
91	140.15	552	0.61
92	141.10	538	0.60
93	142.10	223	0.25
94	143.20	306	0.34
95	144.10	69	0.08
96	145.10	366	0.41
97	146.10	154	0.17
98	147.10	445	0.49
99	148.10	153	0.17
100	149.05	831	0.92
101	150.10	401	0.44
102	151.15	2016	2.23
103	152.15	1511	1.67
104	153.10	854	0.95
105	154.10	488	0.54
106	155.10	290	0.32
107	156.10	61	0.07
108	157.10	84	0.09
109	158.10	20	0.02
110	159.10	210	0.23
111	160.10	49	0.05
112	161.10	312	0.35
113	162.10	238	0.26
114	163.10	373	0.41
115	164.10	333	0.37
116	165.15	1179	1.31
117	166.10	708	0.78
118	167.10	384	0.43
119	168.10	136	0.15
120	169.10	161	0.18
121	170.10	9	0.01
122	171.10	126	0.14
123	173.10	97	0.11
124	174.20	18	0.02
125	175.10	115	0.13
126	176.10	130	0.14

#	m/z	Abs. Int.	Rel. Int.
127	177.10	436	0.48
128	178.10	129	0.14
129	179.10	2208	2.45
130	180.10	507	0.56
131	181.10	264	0.29
132	182.10	138	0.15
133	183.10	50	0.06
134	184.10	9	0.01
135	185.10	59	0.07
136	186.10	27	0.03
137	187.10	29	0.03
138	189.10	173	0.19
139	190.10	19	0.02
140	191.10	246	0.27
141	192.10	463	0.51
142	193.10	981	1.09
143	194.10	381	0.42
144	195.10	119	0.13
145	196.10	90	0.10
146	197.10	29	0.03
147	198.00	117	0.13
148	199.10	20	0.02
149	201.10	20	0.02
150	203.10	17	0.02
151	204.20	8	0.01
152	205.10	47	0.05
153	206.10	20	0.02
154	207.00	702	0.78
155	208.10	655	0.73
156	209.20	183	0.20
157	210.10	101	0.11
158	216.10	8	0.01
159	220.10	53	0.06
160	221.10	219	0.24
161	222.10	179	0.20
162	223.10	129	0.14
163	224.10	20	0.02
164	235.10	38	0.04
165	236.10	171	0.19
166	240.10	9	0.01
167	241.10	17	0.02
168	245.10	8	0.01
169	249.25	318	0.35
170	250.20	81	0.09
171	260.10	8	0.01
172	263.20	330	0.37
173	264.10	51	0.06
174	270.10	9	0.01
175	278.20	201	0.22
176	279.20	50	0.06
177	280.10	18	0.02
178	281.10	125	0.14
179	453.10	8	0.01
180	466.20	9	0.01
181	477.10	18	0.02
182	490.10	8	0.01
183	555.20	8	0.01
184	575.10	8	0.01
185	584.20	8	0.01
186	636.10	9	0.01

Line#:9 R.Time:17.9(Scan#:4168)
MassPeaks:210
RawMode:Averaged 17.8-17.9(4154-4181) BasePeak:57(15404)
BG Mode:None Group 1 - Event 1

#	m/z	Abs. Int.	Rel. Int.
1	50.00	798	5.18
2	51.05	1546	10.04
3	52.10	883	5.73
4	53.10	4039	26.22
5	54.10	1303	8.46
6	55.10	12499	81.14
7	56.10	4328	28.10
8	57.10	15404	100.00
9	58.10	1212	7.87
10	59.15	737	4.78
11	60.05	1718	11.15
12	61.10	976	6.34
13	62.00	409	2.66
14	63.05	992	6.44

#	m/z	Abs. Int.	Rel. Int.
15	64.10	510	3.31
16	65.10	1941	12.60
17	66.05	1185	7.69
18	67.10	9913	64.35
19	68.10	10576	68.66
20	69.10	10213	66.30
21	70.10	3958	25.69
22	71.10	8235	53.46
23	72.15	785	5.10
24	73.05	4922	31.95
25	74.10	774	5.02
26	75.10	1124	7.30
27	76.00	584	3.79
28	77.05	3849	24.99

#	m/z	Abs. Int.	Rel. Int.
29	78.10	1060	6.88
30	79.10	8022	52.08
31	80.10	1979	12.85
32	81.10	14869	96.53
33	82.10	12579	81.66
34	83.10	8350	54.21
35	84.05	1689	10.96
36	85.10	4268	27.71
37	86.10	548	3.56
38	87.10	585	3.80
39	88.10	356	2.31
40	89.10	851	5.52
41	90.10	341	2.21
42	91.05	3209	20.83

DEPTT. OF BOTANICAL & ENVIRONMENTAL SCIENCES, G.N.D.U. AMRITSAR

#	m/z	Abs. Int.	Rel. Int.
43	92.10	732	4.75
44	93.05	1910	12.40
45	94.10	1827	11.86
46	95.10	11073	71.88
47	96.10	4554	29.56
48	97.10	5472	35.52
49	98.05	1265	8.21
50	99.10	1195	7.76
51	100.10	345	2.24
52	101.10	481	3.12
53	102.10	309	2.01
54	103.10	732	4.75
55	104.10	366	2.38
56	105.10	1492	9.69
57	106.10	484	3.14
58	107.10	1559	10.12
59	108.10	705	4.58
60	109.10	4656	30.23
61	110.10	2357	15.30
62	111.10	2803	18.20
63	112.10	669	4.34
64	113.10	657	4.27
65	114.10	158	1.03
66	115.05	946	6.14
67	116.10	422	2.74
68	117.10	803	5.21
69	118.10	334	2.17
70	119.10	1056	6.86
71	120.10	455	2.95
72	121.05	871	5.65
73	122.10	590	3.83
74	123.10	5519	35.83
75	124.15	2548	16.54
76	125.10	1588	10.31
77	126.10	975	6.33
78	127.10	669	4.34
79	128.10	535	3.47
80	129.05	842	5.47
81	130.10	292	1.90
82	131.10	583	3.78
83	132.10	218	1.42
84	133.05	894	5.80
85	134.10	351	2.28
86	135.10	574	3.73
87	136.10	752	4.88
88	137.15	1331	8.64
89	138.10	714	4.64
90	139.10	495	3.21
91	140.10	200	1.30
92	141.10	480	3.12
93	142.10	202	1.31
94	143.10	416	2.70
95	144.10	20	0.13
96	145.00	401	2.60
97	146.10	144	0.93
98	147.10	2486	16.14

#	m/z	Abs. Int.	Rel. Int.
99	148.10	429	2.78
100	149.10	828	5.38
101	150.10	337	2.19
102	151.10	980	6.36
103	152.10	702	4.56
104	153.10	590	3.83
105	154.10	358	2.32
106	155.10	320	2.08
107	156.10	38	0.25
108	157.10	160	1.04
109	158.10	23	0.15
110	159.10	203	1.32
111	160.10	64	0.42
112	161.15	380	2.47
113	162.10	166	1.08
114	163.10	616	4.00
115	164.10	261	1.69
116	165.10	578	3.75
117	166.10	501	3.25
118	167.10	584	3.79
119	168.10	507	3.29
120	169.10	272	1.77
121	170.10	7	0.05
122	171.10	117	0.76
123	173.10	38	0.25
124	174.10	16	0.10
125	175.10	150	0.97
126	176.10	96	0.62
127	177.10	428	2.78
128	178.10	187	1.21
129	179.10	1495	9.71
130	180.10	387	2.51
131	181.10	189	1.23
132	182.10	65	0.42
133	183.00	48	0.31
134	185.00	7	0.05
135	186.00	15	0.10
136	187.00	77	0.50
137	188.10	28	0.18
138	189.00	309	2.01
139	190.10	188	1.22
140	191.10	356	2.31
141	192.10	456	2.96
142	193.10	422	2.74
143	194.10	143	0.93
144	195.10	78	0.51
145	196.10	15	0.10
146	197.00	23	0.15
147	198.00	33	0.21
148	199.10	35	0.23
149	200.00	24	0.16
150	202.10	7	0.05
151	203.10	16	0.10
152	205.00	116	0.75
153	206.00	17	0.11
154	206.95	822	5.34

#	m/z	Abs. Int.	Rel. Int.
155	208.00	537	3.49
156	209.00	152	0.99
157	210.00	40	0.26
158	211.00	17	0.11
159	212.00	153	0.99
160	213.00	9	0.06
161	218.00	7	0.05
162	219.00	8	0.05
163	220.00	52	0.34
164	221.00	393	2.55
165	222.00	164	1.06
166	223.10	154	1.00
167	224.00	23	0.15
168	235.05	315	2.04
169	236.00	93	0.60
170	241.10	7	0.05
171	249.00	60	0.39
172	250.10	135	0.88
173	251.10	45	0.29
174	252.00	7	0.05
175	263.10	23	0.15
176	265.00	41	0.27
177	267.00	140	0.91
178	269.00	15	0.10
179	278.10	190	1.23
180	279.10	54	0.35
181	281.00	233	1.51
182	282.00	31	0.20
183	283.00	30	0.19
184	295.00	23	0.15
185	341.00	140	0.91
186	342.00	17	0.11
187	343.00	7	0.05
188	355.00	104	0.68
189	356.00	51	0.33
190	357.10	15	0.10
191	359.00	8	0.05
192	370.00	7	0.05
193	371.00	7	0.05
194	385.00	7	0.05
195	399.10	15	0.10
196	401.00	16	0.10
197	402.10	7	0.05
198	403.00	7	0.05
199	409.10	7	0.05
200	411.10	7	0.05
201	415.00	15	0.10
202	429.00	160	1.04
203	430.00	26	0.17
204	431.00	15	0.10
205	457.00	7	0.05
206	478.00	16	0.10
207	491.00	7	0.05
208	569.00	9	0.06
209	591.00	7	0.05
210	691.00	8	0.05

Line#:10 R.Time:18.1(Scan#:4224)
MassPeaks:190
RawMode:Averaged 18.0-18.1(4212-4239) BasePeak:81(22702)
BG Mode:None Group 1 - Event 1

#	m/z	Abs. Int.	Rel. Int.
1	50.10	827	3.64
2	51.05	1581	6.96
3	52.10	991	4.37
4	53.10	5352	23.58
5	54.15	1457	6.42
6	55.10	16434	72.39
7	56.10	5669	24.97
8	57.10	21934	96.62
9	58.10	1526	6.72
10	59.10	588	2.59
11	60.05	1643	7.24
12	61.05	1002	4.41
13	62.00	420	1.85
14	63.05	932	4.11
15	64.00	543	2.39
16	65.05	2321	10.22
17	66.10	1340	5.90
18	67.05	13236	58.30

#	m/z	Abs. Int.	Rel. Int.
19	68.10	15960	70.30
20	69.10	15065	66.36
21	70.10	5052	22.25
22	71.10	10897	48.00
23	72.10	927	4.08
24	73.05	1891	8.33
25	74.10	573	2.52
26	75.05	1117	4.92
27	76.05	642	2.83
28	77.05	4575	20.15
29	78.10	1190	5.24
30	79.10	12060	53.12
31	80.05	2648	11.66
32	81.10	22702	100.00
33	82.10	18193	80.14
34	83.10	11383	50.14
35	84.10	1994	8.78
36	85.10	5094	22.44

#	m/z	Abs. Int.	Rel. Int.
37	86.10	679	2.99
38	87.10	567	2.50
39	88.10	566	2.49
40	89.10	814	3.59
41	90.10	335	1.48
42	91.05	3272	14.41
43	92.05	746	3.29
44	93.05	2473	10.89
45	94.10	2666	11.74
46	95.10	17008	74.92
47	96.10	6778	29.86
48	97.10	8044	35.43
49	98.10	1416	6.24
50	99.10	1216	5.36
51	100.10	312	1.37
52	101.00	487	2.15
53	102.10	371	1.63
54	103.10	699	3.08

DEPTT. OF BOTANICAL & ENVIRONMENTAL SCIENCES, G.N.D.U. AMRITSAR

#	m/z	Abs. Int.	Rel. Int.
55	104.10	406	1.79
56	105.10	1412	6.22
57	106.10	458	2.02
58	107.05	1477	6.51
59	108.10	916	4.03
60	109.10	6507	28.66
61	110.15	3112	13.71
62	111.10	4027	17.74
63	112.15	848	3.74
64	113.15	641	2.82
65	114.10	146	0.64
66	115.00	912	4.02
67	116.10	422	1.86
68	117.10	831	3.66
69	118.00	296	1.30
70	119.10	995	4.38
71	120.10	429	1.89
72	121.10	992	4.37
73	122.15	649	2.86
74	123.15	8489	37.39
75	124.15	3519	15.50
76	125.15	1567	6.90
77	126.15	946	4.17
78	127.10	710	3.13
79	128.00	493	2.17
80	129.10	814	3.59
81	130.20	383	1.69
82	131.10	601	2.65
83	132.10	233	1.03
84	133.10	700	3.08
85	134.00	283	1.25
86	135.05	704	3.10
87	136.10	891	3.92
88	137.15	1774	7.81
89	138.15	924	4.07
90	139.10	534	2.35
91	140.20	300	1.32
92	141.10	358	1.58
93	142.20	186	0.82
94	143.00	388	1.71
95	144.10	97	0.43
96	145.10	353	1.55
97	146.10	162	0.71
98	147.00	562	2.48
99	148.10	345	1.52
100	149.05	906	3.99

#	m/z	Abs. Int.	Rel. Int.
101	150.00	356	1.57
102	151.10	1168	5.14
103	152.10	715	3.15
104	153.10	580	2.55
105	154.10	398	1.75
106	155.10	176	0.78
107	156.10	47	0.21
108	157.10	153	0.67
109	158.10	23	0.10
110	159.00	226	1.00
111	160.10	97	0.43
112	161.00	340	1.50
113	162.00	197	0.87
114	163.00	467	2.06
115	164.00	219	0.96
116	165.15	695	3.06
117	166.05	460	2.03
118	167.00	1081	4.76
119	168.00	342	1.51
120	169.00	212	0.93
121	171.10	129	0.57
122	172.10	30	0.13
123	173.10	122	0.54
124	175.00	226	1.00
125	176.00	68	0.30
126	177.00	479	2.11
127	178.10	121	0.53
128	179.10	1425	6.28
129	180.10	445	1.96
130	181.00	314	1.38
131	182.10	114	0.50
132	183.00	84	0.37
133	184.10	16	0.07
134	185.10	36	0.16
135	187.00	36	0.16
136	189.00	292	1.29
137	190.00	75	0.33
138	191.10	408	1.80
139	192.05	552	2.43
140	193.10	526	2.32
141	194.10	202	0.89
142	195.00	83	0.37
143	196.10	30	0.13
144	198.10	52	0.23
145	200.10	7	0.03
146	202.00	7	0.03

#	m/z	Abs. Int.	Rel. Int.
147	203.00	52	0.23
148	204.10	50	0.22
149	205.10	271	1.19
150	206.10	7	0.03
151	207.10	560	2.47
152	208.10	395	1.74
153	209.10	99	0.44
154	210.10	60	0.26
155	211.10	41	0.18
156	213.10	16	0.07
157	217.00	156	0.69
158	218.10	16	0.07
159	219.10	11	0.05
160	220.10	96	0.42
161	221.10	135	0.59
162	222.10	69	0.30
163	223.10	24	0.11
164	226.10	8	0.04
165	232.10	25	0.11
166	235.10	47	0.21
167	236.10	26	0.11
168	238.00	7	0.03
169	240.10	7	0.03
170	241.10	18	0.08
171	244.00	7	0.03
172	246.10	7	0.03
173	249.10	40	0.18
174	261.00	23	0.10
175	263.10	39	0.17
176	266.10	15	0.07
177	267.10	16	0.07
178	278.20	623	2.74
179	279.30	89	0.39
180	281.00	42	0.19
181	282.10	7	0.03
182	295.00	15	0.07
183	341.20	16	0.07
184	355.10	7	0.03
185	397.10	7	0.03
186	410.30	8	0.04
187	458.20	7	0.03
188	487.10	7	0.03
189	608.30	7	0.03
190	624.20	15	0.07

Line#:11 R.Time:18.5(Scan#:4349)
MassPeaks:207
RawMode:Averaged 18.5-18.5(4339-4360) BasePeak:149(185218)
BG Mode:None Group 1 - Event 1

#	m/z	Abs. Int.	Rel. Int.
1	50.10	4831	2.61
2	51.10	3086	1.67
3	52.10	1281	0.69
4	53.10	2633	1.42
5	54.10	1338	0.72
6	55.10	9035	4.88
7	56.10	11833	6.39
8	57.10	16349	8.83
9	58.10	1177	0.64
10	59.10	713	0.38
11	60.10	1684	0.91
12	61.00	972	0.52
13	62.00	447	0.24
14	63.10	1230	0.66
15	64.05	811	0.44
16	65.05	11483	6.20
17	66.05	1697	0.92
18	67.05	2650	1.43
19	68.10	1158	0.63
20	69.10	4112	2.22
21	70.10	2018	1.09
22	71.05	3602	1.94
23	72.05	671	0.36
24	73.05	3573	1.93
25	74.05	1678	0.91
26	75.05	2927	1.58
27	76.05	12978	7.01
28	77.05	6598	3.56

#	m/z	Abs. Int.	Rel. Int.
29	78.05	1191	0.64
30	79.05	2619	1.41
31	80.15	715	0.39
32	81.05	2830	1.53
33	82.10	1522	0.82
34	83.10	2615	1.41
35	84.05	973	0.53
36	85.10	2382	1.29
37	86.10	402	0.22
38	87.10	623	0.34
39	88.10	420	0.23
40	89.05	1136	0.61
41	90.00	407	0.22
42	91.05	2789	1.51
43	92.00	1275	0.69
44	93.00	10519	5.68
45	94.05	1463	0.79
46	95.05	2104	1.14
47	96.10	1302	0.70
48	97.10	2622	1.42
49	98.10	792	0.43
50	99.05	1147	0.62
51	100.05	556	0.30
52	101.00	1014	0.55
53	102.10	512	0.28
54	103.05	1114	0.60
55	104.05	11883	6.42
56	105.05	8512	4.60

#	m/z	Abs. Int.	Rel. Int.
57	106.05	1273	0.69
58	107.05	1157	0.62
59	108.10	486	0.26
60	109.10	1308	0.71
61	110.05	903	0.49
62	111.10	1348	0.73
63	112.10	419	0.23
64	113.10	644	0.35
65	114.10	64	0.03
66	115.05	1110	0.60
67	116.10	482	0.26
68	117.05	983	0.53
69	118.10	409	0.22
70	119.05	1092	0.59
71	120.05	542	0.29
72	121.00	8390	4.53
73	122.05	3998	2.16
74	123.05	3338	1.80
75	124.05	656	0.35
76	125.10	760	0.41
77	126.10	671	0.36
78	127.10	557	0.30
79	128.10	437	0.24
80	129.10	1078	0.58
81	130.10	548	0.30
82	131.05	804	0.43
83	132.00	1417	0.77
84	133.05	1155	0.62

#	m/z	Abs. Int.	Rel. Int.
85	134.10	444	0.24
86	135.10	1504	0.81
87	136.05	871	0.47
88	137.00	577	0.31
89	138.10	387	0.21
90	139.10	376	0.20
91	140.10	158	0.09
92	141.00	470	0.25
93	142.10	146	0.08
94	143.10	489	0.26
95	144.10	115	0.06
96	145.00	421	0.23
97	146.10	151	0.08
98	147.05	982	0.53
99	148.05	1051	0.57
100	149.05	185218	100.00
101	150.05	16244	8.77
102	151.05	2650	1.43
103	152.05	939	0.51
104	153.10	587	0.32
105	154.00	348	0.19
106	155.00	277	0.15
107	156.00	29	0.02
108	157.00	203	0.11
109	158.10	10	0.01
110	159.10	259	0.14
111	160.10	1402	0.76
112	161.05	523	0.28
113	162.10	368	0.20
114	163.00	488	0.26
115	164.00	301	0.16
116	165.10	580	0.31
117	166.10	426	0.23
118	167.00	1072	0.58
119	168.00	318	0.17
120	169.20	228	0.12
121	170.00	9	0.00
122	171.20	144	0.08
123	172.00	10	0.01
124	173.00	75	0.04
125	174.00	20	0.01

#	m/z	Abs. Int.	Rel. Int.
126	175.00	170	0.09
127	176.10	190	0.10
128	177.00	577	0.31
129	178.00	364	0.20
130	179.00	1173	0.63
131	180.00	390	0.21
132	181.00	278	0.15
133	182.00	53	0.03
134	183.00	194	0.10
135	184.00	19	0.01
136	185.10	40	0.02
137	186.00	28	0.02
138	187.10	40	0.02
139	189.10	97	0.05
140	190.00	19	0.01
141	191.00	263	0.14
142	192.00	427	0.23
143	193.00	451	0.24
144	194.00	122	0.07
145	195.00	187	0.10
146	197.00	49	0.03
147	198.00	248	0.13
148	199.00	33	0.02
149	200.00	31	0.02
150	201.00	9	0.00
151	202.00	150	0.08
152	203.20	301	0.16
153	204.00	459	0.25
154	205.05	5602	3.02
155	206.05	1013	0.55
156	206.95	844	0.46
157	207.90	321	0.17
158	208.90	274	0.15
159	210.00	63	0.03
160	210.90	58	0.03
161	211.90	20	0.01
162	212.90	9	0.00
163	215.00	9	0.00
164	217.00	28	0.02
165	219.00	31	0.02
166	220.10	20	0.01

#	m/z	Abs. Int.	Rel. Int.
167	221.00	111	0.06
168	222.00	88	0.05
169	223.05	4330	2.34
170	224.05	725	0.39
171	225.00	76	0.04
172	227.10	49	0.03
173	228.00	9	0.00
174	233.10	182	0.10
175	234.00	46	0.02
176	235.00	50	0.03
177	236.00	76	0.04
178	240.00	11	0.01
179	241.10	63	0.03
180	249.00	19	0.01
181	250.00	32	0.02
182	250.90	11	0.01
183	253.00	89	0.05
184	254.00	30	0.02
185	254.90	19	0.01
186	267.00	42	0.02
187	268.10	9	0.00
188	269.00	63	0.03
189	275.00	10	0.01
190	278.00	139	0.08
191	279.00	51	0.03
192	280.90	297	0.16
193	283.00	65	0.04
194	284.00	174	0.09
195	285.00	41	0.02
196	291.10	9	0.00
197	341.00	9	0.00
198	345.00	11	0.01
199	354.90	38	0.02
200	371.00	9	0.00
201	477.10	9	0.00
202	527.00	9	0.00
203	556.00	9	0.00
204	567.90	9	0.00
205	591.00	11	0.01
206	623.00	9	0.00
207	681.00	9	0.00

Line#:12 R.Time:18.8(Scan#:4455)
MassPeaks:219
RawMode:Averaged 18.8-18.9(4429-4468) BasePeak:73(69762)
BG Mode:None Group 1 - Event 1

#	m/z	Abs. Int.	Rel. Int.
1	50.05	798	1.14
2	51.10	1524	2.18
3	52.10	892	1.28
4	53.10	4706	6.75
5	54.10	4348	6.23
6	55.10	53474	76.65
7	56.10	13438	19.26
8	57.10	62105	89.02
9	58.10	3566	5.11
10	59.15	3749	5.37
11	60.05	60366	86.53
12	61.05	16657	23.88
13	62.05	952	1.36
14	63.10	1116	1.60
15	64.00	1306	1.87
16	65.05	2119	3.04
17	66.05	1347	1.93
18	67.05	9186	13.17
19	68.10	5405	7.75
20	69.10	32429	46.49
21	70.10	9692	13.89
22	71.10	35347	50.67
23	72.05	2617	3.75
24	73.05	69762	100.00
25	74.05	8549	12.25
26	75.10	1799	2.58
27	76.00	738	1.06
28	77.05	3499	5.02
29	78.05	1245	1.78
30	79.05	4482	6.42
31	80.05	1511	2.17
32	81.05	8008	11.48
33	82.10	6563	9.41

#	m/z	Abs. Int.	Rel. Int.
34	83.10	24268	34.79
35	84.05	9492	13.61
36	85.10	23434	33.59
37	86.10	2288	3.28
38	87.05	22422	32.14
39	88.05	3774	5.41
40	89.10	1327	1.90
41	90.10	410	0.59
42	91.05	3080	4.42
43	92.05	793	1.14
44	93.05	3188	4.57
45	94.10	1483	2.13
46	95.10	5673	8.13
47	96.10	5603	8.03
48	97.10	18492	26.51
49	98.10	9048	12.97
50	99.10	7457	10.69
51	100.10	1085	1.56
52	101.10	8363	11.99
53	102.10	3734	5.35
54	103.05	1518	2.18
55	104.05	516	0.74
56	105.10	1889	2.71
57	106.10	717	1.03
58	107.10	2408	3.45
59	108.10	938	1.34
60	109.10	3061	4.39
61	110.10	3097	4.44
62	111.10	7777	11.15
63	112.15	2775	3.98
64	113.10	3854	5.52
65	114.15	695	1.00
66	115.10	11895	17.05

#	m/z	Abs. Int.	Rel. Int.
67	116.10	4590	6.58
68	117.15	1244	1.78
69	118.10	439	0.63
70	119.10	1297	1.86
71	120.10	524	0.75
72	121.10	2189	3.14
73	122.10	865	1.24
74	123.10	2429	3.48
75	124.15	1728	2.48
76	125.15	3290	4.72
77	126.10	1616	2.32
78	127.15	2498	3.58
79	127.90	976	1.40
80	129.10	24121	34.58
81	130.10	3332	4.78
82	131.05	981	1.41
83	132.10	339	0.49
84	133.05	971	1.39
85	134.10	468	0.67
86	135.15	1594	2.28
87	136.10	832	1.19
88	137.10	1091	1.56
89	138.10	1156	1.66
90	139.15	1446	2.07
91	140.15	656	0.94
92	141.15	1452	2.08
93	142.15	344	0.49
94	143.10	6075	8.71
95	144.10	1533	2.20
96	145.10	676	0.97
97	146.20	179	0.26
98	147.10	995	1.43
99	148.10	303	0.43

DEPTT. OF BOTANICAL & ENVIRONMENTAL SCIENCES, G.N.D.U. AMRITSAR

#	m/z	Abs. Int.	Rel. Int.	#	m/z	Abs. Int.	Rel. Int.	#	m/z	Abs. Int.	Rel. Int.
100	149.10	1580	2.26	140	189.10	229	0.33	180	236.10	212	0.30
101	150.10	557	0.80	141	190.10	57	0.08	181	237.20	114	0.16
102	151.10	1230	1.76	142	191.10	475	0.68	182	238.10	17	0.02
103	152.10	1129	1.62	143	191.85	572	0.82	183	239.10	130	0.19
104	153.10	1056	1.51	144	193.10	457	0.66	184	240.20	16	0.02
105	154.15	830	1.19	145	194.15	1154	1.65	185	241.10	103	0.15
106	155.20	705	1.01	146	195.10	377	0.54	186	242.00	11	0.02
107	156.10	128	0.18	147	196.10	243	0.35	187	251.10	10	0.01
108	157.10	8685	12.45	148	197.10	218	0.31	188	252.20	5	0.01
109	158.15	1442	2.07	149	198.10	86	0.12	189	253.10	10	0.01
110	159.10	437	0.63	150	199.10	3447	4.94	190	254.10	79	0.11
111	159.85	262	0.38	151	200.05	557	0.80	191	255.10	16	0.02
112	161.10	479	0.69	152	201.10	121	0.17	192	256.15	2983	4.28
113	161.90	281	0.40	153	202.10	30	0.04	193	257.15	548	0.79
114	163.10	765	1.10	154	203.10	209	0.30	194	257.90	75	0.11
115	164.10	354	0.51	155	204.10	53	0.08	195	260.20	10	0.01
116	165.10	855	1.23	156	205.10	271	0.39	196	261.10	28	0.04
117	166.15	949	1.36	157	206.10	94	0.13	197	265.00	5	0.01
118	167.05	1255	1.80	158	207.10	704	1.01	198	266.10	19	0.03
119	168.20	688	0.99	159	208.10	372	0.53	199	267.10	58	0.08
120	169.10	275	0.39	160	209.10	221	0.32	200	269.10	5	0.01
121	170.10	46	0.07	161	210.10	334	0.48	201	275.10	5	0.01
122	171.15	6962	9.98	162	211.10	60	0.09	202	281.10	268	0.38
123	172.15	1026	1.47	163	212.10	89	0.13	203	282.10	10	0.01
124	173.10	241	0.35	164	213.10	8087	11.59	204	283.20	10	0.01
125	174.10	61	0.09	165	214.05	1200	1.72	205	291.10	24	0.03
126	175.10	292	0.42	166	215.10	174	0.25	206	302.90	5	0.01
127	176.10	100	0.14	167	217.10	44	0.06	207	308.20	5	0.01
128	177.15	625	0.90	168	218.10	5	0.01	208	326.10	5	0.01
129	178.00	308	0.44	169	219.00	232	0.33	209	341.20	5	0.01
130	179.00	1469	2.11	170	220.00	243	0.35	210	355.20	34	0.05
131	180.10	534	0.77	171	221.10	273	0.39	211	383.10	5	0.01
132	181.00	423	0.61	172	222.10	126	0.18	212	385.10	5	0.01
133	182.10	359	0.51	173	223.10	247	0.35	213	464.10	6	0.01
134	183.00	260	0.37	174	224.10	65	0.09	214	585.10	5	0.01
135	184.10	33	0.05	175	225.10	5	0.01	215	628.00	5	0.01
136	185.15	6766	9.70	176	226.10	20	0.03	216	660.10	10	0.01
137	186.10	922	1.32	177	227.10	2174	3.12	217	670.10	10	0.01
138	187.10	228	0.33	178	228.15	330	0.47	218	677.10	5	0.01
139	188.10	22	0.03	179	233.20	10	0.01	219	684.10	5	0.01

Line#:13 R.Time:19.2(Scan#:4567)
MassPeaks:202
RawMode:Averaged 19.2-19.3(4555-4576) BasePeak:57(81148)
BG Mode:None Group 1 - Event 1

#	m/z	Abs. Int.	Rel. Int.	#	m/z	Abs. Int.	Rel. Int.	#	m/z	Abs. Int.	Rel. Int.
1	50.00	723	0.89	35	84.10	24098	29.70	69	118.00	347	0.43
2	51.05	1484	1.83	36	85.10	24068	29.66	70	119.05	855	1.05
3	52.10	931	1.15	37	86.10	1834	2.26	71	120.00	418	0.52
4	53.10	5288	6.52	38	87.00	1249	1.54	72	121.00	801	0.99
5	54.10	9844	12.13	39	88.10	418	0.52	73	122.00	529	0.65
6	55.10	76017	93.68	40	89.00	860	1.06	74	123.10	2126	2.62
7	56.10	37886	46.69	41	90.10	335	0.41	75	124.15	3485	4.29
8	57.10	81148	100.00	42	91.05	6691	8.25	76	125.15	13646	16.82
9	58.10	4075	5.02	43	92.05	914	1.13	77	126.15	4740	5.84
10	59.05	764	0.94	44	93.05	1553	1.91	78	127.15	1811	2.23
11	60.05	2774	3.42	45	94.05	1002	1.23	79	128.10	537	0.66
12	61.05	2117	2.61	46	95.10	6474	7.98	80	129.10	1549	1.91
13	62.10	382	0.47	47	96.10	13265	16.35	81	130.10	420	0.52
14	63.05	948	1.17	48	97.10	67701	83.43	82	131.10	604	0.74
15	64.00	380	0.47	49	98.10	14604	18.00	83	132.10	227	0.28
16	65.05	2395	2.95	50	99.10	5897	7.27	84	133.00	754	0.93
17	66.05	2208	2.72	51	100.10	968	1.19	85	134.10	289	0.36
18	67.05	17163	21.15	52	101.05	1046	1.29	86	135.10	668	0.82
19	68.10	12126	14.94	53	102.00	426	0.52	87	136.10	657	0.81
20	69.10	60977	75.14	54	103.10	629	0.78	88	137.15	1127	1.39
21	70.10	37736	46.50	55	104.10	369	0.45	89	138.15	1976	2.44
22	71.10	39656	48.87	56	105.10	1273	1.57	90	139.15	4933	6.08
23	72.10	2579	3.18	57	106.10	413	0.51	91	140.15	2498	3.08
24	73.05	3589	4.42	58	107.10	1247	1.54	92	141.15	1145	1.41
25	74.00	741	0.91	59	108.10	655	0.81	93	142.10	312	0.38
26	75.05	1044	1.29	60	109.10	2802	3.45	94	143.05	1057	1.30
27	76.00	612	0.75	61	110.15	6094	7.51	95	144.10	287	0.35
28	77.05	3057	3.77	62	111.10	31867	39.27	96	145.10	461	0.57
29	78.05	873	1.08	63	112.10	8232	10.14	97	146.10	150	0.18
30	79.05	3498	4.31	64	113.15	2968	3.66	98	147.10	681	0.84
31	80.10	1381	1.70	65	114.00	355	0.44	99	148.10	190	0.23
32	81.10	13236	16.31	66	115.10	1435	1.77	100	149.10	745	0.92
33	82.10	21567	26.58	67	116.10	542	0.67	101	150.10	279	0.34
34	83.10	70385	86.74	68	117.05	839	1.03	102	151.10	938	1.16

DEPTT. OF BOTANICAL & ENVIRONMENTAL SCIENCES, G.N.D.U. AMRITSAR

#	m/z	Abs. Int.	Rel. Int.
103	152.15	1384	1.71
104	153.15	2396	2.95
105	154.15	1582	1.95
106	155.20	656	0.81
107	156.20	104	0.13
108	157.10	460	0.57
109	158.10	102	0.13
110	159.10	228	0.28
111	161.10	339	0.42
112	162.20	258	0.32
113	163.10	418	0.52
114	164.10	176	0.22
115	165.10	508	0.63
116	166.10	864	1.06
117	167.15	1218	1.50
118	168.15	977	1.20
119	169.10	295	0.36
120	170.20	30	0.04
121	171.10	333	0.41
122	173.20	115	0.14
123	175.10	224	0.28
124	176.20	77	0.09
125	177.10	352	0.43
126	178.10	176	0.22
127	179.05	1039	1.28
128	180.10	678	0.84
129	181.15	675	0.83
130	182.10	517	0.64
131	183.10	202	0.25
132	185.10	321	0.40
133	186.10	9	0.01
134	187.10	149	0.18
135	189.00	325	0.40
136	190.10	111	0.14
137	191.10	432	0.53
138	192.10	367	0.45
139	193.10	333	0.41
140	194.05	339	0.42
141	195.10	382	0.47
142	196.10	357	0.44
143	197.10	184	0.23
144	198.00	31	0.04
145	199.00	109	0.13
146	201.10	27	0.03
147	202.20	31	0.04
148	203.10	173	0.21
149	204.10	58	0.07
150	205.20	153	0.19
151	206.00	251	0.31
152	207.10	653	0.80
153	208.10	443	0.55
154	209.10	348	0.43
155	210.10	311	0.38
156	211.10	106	0.13
157	213.00	246	0.30
158	215.00	9	0.01
159	217.10	29	0.04
160	219.10	35	0.04
161	220.10	68	0.08
162	221.10	100	0.12
163	222.10	115	0.14
164	223.10	240	0.30
165	224.10	209	0.26
166	225.10	59	0.07
167	227.20	34	0.04
168	231.10	22	0.03
169	233.10	20	0.02
170	234.00	9	0.01
171	235.00	19	0.02
172	236.00	45	0.06
173	237.10	21	0.03
174	238.10	70	0.09
175	239.10	32	0.04
176	240.10	9	0.01
177	241.10	9	0.01
178	243.10	9	0.01
179	244.10	93	0.11
180	245.10	9	0.01
181	250.10	9	0.01
182	251.10	42	0.05
183	252.10	158	0.19
184	253.20	40	0.05
185	255.10	47	0.06
186	256.00	41	0.05
187	263.00	228	0.28
188	267.10	21	0.03
189	269.10	45	0.06
190	278.10	10	0.01
191	280.10	112	0.14
192	281.10	121	0.15
193	282.10	20	0.02
194	283.20	9	0.01
195	292.10	10	0.01
196	295.10	9	0.01
197	298.20	19	0.02
198	333.10	9	0.01
199	375.10	18	0.02
200	531.10	9	0.01
201	668.00	10	0.01
202	673.10	9	0.01

Line#:14 R.Time:20.3(Scan#:4880)
MassPeaks:207
RawMode:Averaged 20.2-20.3(4869-4897) BasePeak:71(90082)
BG Mode:None Group 1 - Event 1

#	m/z	Abs. Int.	Rel. Int.
1	50.05	695	0.77
2	51.10	1351	1.50
3	52.10	774	0.86
4	53.10	4703	5.22
5	54.15	2005	2.23
6	55.10	32119	35.66
7	56.10	16558	18.38
8	57.10	40522	44.98
9	58.15	2731	3.03
10	59.10	796	0.88
11	60.10	1660	1.84
12	61.10	781	0.87
13	62.10	302	0.34
14	63.10	792	0.88
15	64.10	380	0.42
16	65.10	2040	2.26
17	66.10	1031	1.14
18	67.10	14961	16.61
19	68.10	18522	20.56
20	69.10	29111	32.32
21	70.10	16197	17.98
22	71.10	90082	100.00
23	72.10	5104	5.67
24	73.05	3046	3.38
25	74.00	594	0.66
26	75.00	1021	1.13
27	76.10	508	0.56
28	77.05	3178	3.53
29	78.10	847	0.94
30	79.10	5522	6.13
31	80.10	3657	4.06
32	81.10	27168	30.16
33	82.10	13468	14.95
34	83.10	17980	19.96
35	84.10	8790	9.76
36	85.10	11549	12.82
37	86.10	3715	4.12
38	87.10	1368	1.52
39	88.10	333	0.37
40	89.10	659	0.73
41	90.10	130	0.14
42	91.10	3164	3.51
43	92.10	757	0.84
44	93.10	2618	2.91
45	94.10	2244	2.49
46	95.10	17033	18.91
47	96.10	7423	8.24
48	97.10	13105	14.55
49	98.15	4391	4.87
50	99.10	3906	4.34
51	100.15	784	0.87
52	101.10	701	0.78
53	102.10	333	0.37
54	103.10	592	0.66
55	104.10	413	0.46
56	105.10	1507	1.67
57	106.10	435	0.48
58	107.10	1558	1.73
59	108.15	778	0.86
60	109.10	7339	8.15
61	110.15	3793	4.21
62	111.15	9107	10.11
63	112.15	2822	3.13
64	113.15	1815	2.01
65	114.20	253	0.28
66	115.10	1107	1.23
67	116.10	406	0.45
68	117.05	896	0.99
69	118.00	226	0.25
70	119.05	892	0.99
71	120.00	408	0.45
72	121.10	1269	1.41
73	122.15	1302	1.45
74	123.15	16687	18.52
75	124.15	4946	5.49
76	125.15	3507	3.89
77	126.15	4479	4.97
78	127.15	1725	1.91
79	128.20	539	0.60
80	129.05	938	1.04
81	130.10	385	0.43
82	131.20	629	0.70
83	132.10	182	0.20
84	133.10	682	0.76
85	134.10	402	0.45
86	135.05	807	0.90
87	136.10	628	0.70
88	137.15	1851	2.05
89	138.15	1177	1.31
90	139.15	973	1.08
91	140.15	975	1.08
92	141.15	869	0.96
93	142.20	189	0.21
94	143.10	418	0.46
95	144.10	82	0.09
96	145.10	444	0.49
97	146.10	104	0.12
98	147.10	628	0.70
99	148.20	160	0.18
100	149.15	869	0.96
101	150.10	430	0.48
102	151.15	1175	1.30
103	152.20	817	0.91
104	153.15	610	0.68
105	154.10	348	0.39
106	155.20	368	0.41
107	156.10	69	0.08
108	157.10	226	0.25
109	158.10	41	0.05
110	159.20	187	0.21
111	160.10	23	0.03
112	161.10	286	0.32
113	162.10	183	0.20
114	163.20	380	0.42
115	164.10	224	0.25
116	165.15	756	0.84
117	166.20	491	0.55
118	167.10	424	0.47
119	168.20	235	0.26
120	169.20	290	0.32

DEPTT. OF BOTANICAL & ENVIRONMENTAL SCIENCES, G.N.D.U. AMRITSAR

#	m/z	Abs. Int.	Rel. Int.
121	170.20	39	0.04
122	171.10	244	0.27
123	173.10	119	0.13
124	174.10	29	0.03
125	175.20	249	0.28
126	176.10	64	0.07
127	177.10	334	0.37
128	178.10	129	0.14
129	179.10	1148	1.27
130	180.10	292	0.32
131	181.10	307	0.34
132	182.10	220	0.24
133	183.10	175	0.19
134	185.10	159	0.18
135	186.10	14	0.02
136	187.20	107	0.12
137	189.10	221	0.25
138	190.10	36	0.04
139	191.10	370	0.41
140	192.10	286	0.32
141	193.10	459	0.51
142	194.10	120	0.13
143	195.10	89	0.10
144	196.20	306	0.34
145	197.10	512	0.57
146	198.00	67	0.07
147	199.20	75	0.08
148	201.10	24	0.03
149	202.10	23	0.03

#	m/z	Abs. Int.	Rel. Int.
150	203.20	86	0.10
151	204.20	14	0.02
152	205.10	186	0.21
153	206.20	22	0.02
154	207.10	562	0.62
155	208.10	296	0.33
156	209.10	133	0.15
157	210.00	84	0.09
158	211.20	122	0.14
159	213.10	38	0.04
160	215.10	8	0.01
161	216.10	16	0.02
162	217.10	55	0.06
163	218.10	31	0.03
164	219.10	47	0.05
165	220.10	59	0.07
166	221.10	143	0.16
167	222.20	87	0.10
168	223.10	208	0.23
169	224.10	14	0.02
170	225.10	7	0.01
171	226.20	7	0.01
172	227.20	7	0.01
173	230.10	22	0.02
174	231.10	14	0.02
175	233.10	22	0.02
176	234.10	54	0.06
177	235.10	39	0.04
178	236.10	23	0.03

#	m/z	Abs. Int.	Rel. Int.
179	237.10	6	0.01
180	238.10	14	0.02
181	239.10	21	0.02
182	241.10	8	0.01
183	243.20	22	0.02
184	247.10	7	0.01
185	249.10	103	0.11
186	250.10	14	0.02
187	251.10	21	0.02
188	252.10	7	0.01
189	256.10	8	0.01
190	263.10	56	0.06
191	264.20	8	0.01
192	267.10	40	0.04
193	274.10	7	0.01
194	278.20	157	0.17
195	279.20	7	0.01
196	280.10	15	0.02
197	281.00	258	0.29
198	283.20	23	0.03
199	322.20	7	0.01
200	342.20	7	0.01
201	426.10	7	0.01
202	444.10	7	0.01
203	512.20	6	0.01
204	570.20	6	0.01
205	664.20	7	0.01
206	669.00	7	0.01
207	687.10	7	0.01

Line#:15 R.Time:20.5(Scan#:4940)
MassPeaks:220
RawMode:Averaged 20.4-20.5(4930-4952) BasePeak:55(41940)
BG Mode:None Group 1 - Event 1

#	m/z	Abs. Int.	Rel. Int.
1	50.05	840	2.00
2	51.10	1727	4.12
3	52.15	1031	2.46
4	53.10	5208	12.42
5	54.10	8762	20.89
6	55.10	41940	100.00
7	56.10	12364	29.48
8	57.10	21857	52.11
9	58.15	1792	4.27
10	59.10	1776	4.23
11	60.05	6049	14.42
12	61.10	1462	3.49
13	62.00	424	1.01
14	63.05	997	2.38
15	64.00	519	1.24
16	65.05	3357	8.00
17	66.05	2413	5.75
18	67.05	22490	53.62
19	68.10	10251	24.44
20	69.10	28987	69.12
21	70.10	11845	28.24
22	71.10	10835	25.83
23	72.05	1012	2.41
24	73.05	8312	19.82
25	74.05	1151	2.74
26	75.05	1228	2.93
27	76.10	604	1.44
28	77.05	6208	14.80
29	78.05	2769	6.60
30	79.05	16076	38.33
31	80.10	6760	16.12
32	81.10	18022	42.97
33	82.10	11507	27.44
34	83.10	22278	53.12
35	84.10	13648	32.54
36	85.10	6866	16.37
37	86.15	857	2.04
38	87.05	3351	7.99
39	88.10	611	1.46
40	89.05	998	2.38
41	90.05	469	1.12
42	91.05	7231	17.24
43	92.10	1677	4.00
44	93.10	7839	18.69
45	94.10	4594	10.95

#	m/z	Abs. Int.	Rel. Int.
46	95.10	14206	33.87
47	96.10	10851	25.87
48	97.10	17847	42.55
49	98.10	8583	20.46
50	99.10	3112	7.42
51	100.05	1606	3.83
52	101.10	3233	7.71
53	102.10	830	1.98
54	103.10	988	2.36
55	104.00	567	1.35
56	105.10	3363	8.02
57	106.05	1016	2.42
58	107.10	4249	10.13
59	108.05	4169	9.94
60	109.10	6309	15.04
61	110.10	5608	13.37
62	111.10	7799	18.60
63	112.10	2974	7.09
64	113.10	1708	4.07
65	114.05	1785	4.26
66	115.10	2410	5.75
67	116.10	788	1.88
68	117.00	1502	3.58
69	118.10	568	1.35
70	119.10	2953	7.04
71	120.10	1142	2.72
72	121.10	3817	9.10
73	122.10	1842	4.39
74	123.10	4581	10.92
75	124.10	2775	6.62
76	125.10	3246	7.74
77	126.10	1681	4.01
78	127.10	1975	4.71
79	128.15	845	2.01
80	129.10	2192	5.23
81	130.10	722	1.72
82	131.10	1175	2.80
83	132.00	450	1.07
84	133.10	2388	5.69
85	134.10	1403	3.35
86	135.20	2732	6.51
87	136.10	1616	3.85
88	137.10	2206	5.26
89	138.15	1837	4.38
90	139.10	1714	4.09

#	m/z	Abs. Int.	Rel. Int.
91	140.15	840	2.00
92	141.05	847	2.02
93	142.10	399	0.95
94	143.10	967	2.31
95	144.10	355	0.85
96	145.10	619	1.48
97	146.10	302	0.72
98	147.10	1855	4.42
99	148.10	1077	2.57
100	149.10	2209	5.27
101	150.10	957	2.28
102	151.15	2105	5.02
103	152.15	1492	3.56
104	153.15	1160	2.77
105	154.10	617	1.47
106	155.10	525	1.25
107	156.10	177	0.42
108	157.10	599	1.43
109	158.10	136	0.32
110	159.10	382	0.91
111	160.10	139	0.33
112	161.00	1437	3.43
113	162.10	511	1.22
114	163.15	877	2.09
115	164.10	496	1.18
116	165.10	1197	2.85
117	166.10	949	2.26
118	167.10	838	2.00
119	168.10	381	0.91
120	169.10	488	1.16
121	170.10	77	0.18
122	171.20	453	1.08
123	172.10	182	0.43
124	173.10	385	0.92
125	174.20	52	0.12
126	175.20	541	1.29
127	176.10	211	0.50
128	177.10	745	1.78
129	178.05	1438	3.43
130	179.10	1614	3.85
131	180.10	766	1.83
132	181.20	547	1.30
133	182.10	313	0.75
134	183.10	344	0.82
135	184.10	28	0.07

DEPTT. OF BOTANICAL & ENVIRONMENTAL SCIENCES, G.N.D.U. AMRITSAR

#	m/z	Abs. Int.	Rel. Int.
136	185.10	543	1.29
137	186.10	162	0.39
138	187.10	165	0.39
139	189.05	526	1.25
140	190.20	201	0.48
141	191.10	502	1.20
142	192.10	386	0.92
143	193.10	642	1.53
144	194.10	370	0.88
145	195.10	355	0.85
146	196.10	127	0.30
147	197.10	249	0.59
148	198.10	48	0.11
149	199.10	291	0.69
150	200.10	48	0.11
151	201.00	19	0.05
152	202.00	9	0.02
153	203.10	302	0.72
154	204.10	148	0.35
155	205.05	596	1.42
156	206.10	139	0.33
157	207.05	1155	2.75
158	208.00	472	1.13
159	209.10	359	0.86
160	210.10	147	0.35
161	211.10	144	0.34
162	212.00	9	0.02
163	213.00	1412	3.37
164	214.00	274	0.65

#	m/z	Abs. Int.	Rel. Int.
165	215.00	42	0.10
166	216.00	18	0.04
167	217.00	151	0.36
168	218.00	66	0.16
169	219.00	199	0.47
170	220.10	497	1.19
171	221.15	590	1.41
172	222.15	676	1.61
173	223.10	423	1.01
174	224.10	48	0.11
175	225.10	31	0.07
176	226.00	12	0.03
177	227.00	38	0.09
178	228.10	215	0.51
179	229.00	29	0.07
180	230.10	20	0.05
181	231.10	43	0.10
182	233.05	424	1.01
183	234.00	80	0.19
184	235.00	336	0.80
185	236.00	166	0.40
186	237.00	79	0.19
187	238.10	43	0.10
188	239.00	122	0.29
189	241.00	30	0.07
190	246.10	100	0.24
191	247.10	60	0.14
192	248.00	10	0.02
193	249.10	177	0.42

#	m/z	Abs. Int.	Rel. Int.
194	250.00	21	0.05
195	251.10	43	0.10
196	253.00	97	0.23
197	255.00	30	0.07
198	257.00	8	0.02
199	262.00	9	0.02
200	263.00	102	0.24
201	264.15	796	1.90
202	265.10	244	0.58
203	267.00	52	0.12
204	268.00	22	0.05
205	278.10	41	0.10
206	279.10	250	0.60
207	280.20	20	0.05
208	281.10	321	0.77
209	282.10	113	0.27
210	283.10	8	0.02
211	287.00	8	0.02
212	290.10	19	0.05
213	300.00	9	0.02
214	341.00	9	0.02
215	355.10	19	0.05
216	424.10	9	0.02
217	448.00	8	0.02
218	532.00	9	0.02
219	594.10	9	0.02
220	633.00	19	0.05

Line#:16 R.Time:20.7(Scan#:5016)
MassPeaks:219
RawMode:Averaged 20.7-20.8(5000-5032) BasePeak:57(27289)
BG Mode:None Group 1 - Event 1

#	m/z	Abs. Int.	Rel. Int.
1	50.00	689	2.52
2	51.05	1201	4.40
3	52.10	657	2.41
4	53.10	2728	10.00
5	54.10	2864	10.50
6	55.10	24519	89.85
7	56.10	6546	23.99
8	57.10	27289	100.00
9	58.10	1845	6.76
10	59.05	1907	6.99
11	60.05	18327	67.16
12	61.05	6102	22.36
13	62.10	501	1.84
14	63.10	856	3.14
15	64.00	379	1.39
16	65.10	1632	5.98
17	66.10	1064	3.90
18	67.10	7564	27.72
19	68.10	3792	13.90
20	69.10	15893	58.24
21	70.10	5230	19.17
22	71.10	15774	57.80
23	72.05	1371	5.02
24	73.05	22453	82.28
25	74.05	3849	14.10
26	75.05	1485	5.44
27	76.00	579	2.12
28	77.05	3269	11.98
29	78.10	1174	4.30
30	79.05	4942	18.11
31	80.10	1931	7.08
32	81.10	6914	25.34
33	82.10	4472	16.39
34	83.10	12295	45.05
35	84.10	4686	17.17
36	85.10	10564	38.71
37	86.05	1195	4.38
38	87.05	8382	30.72
39	88.05	1459	5.35
40	89.10	861	3.16
41	90.10	250	0.92
42	91.05	3891	14.26
43	92.05	922	3.38
44	93.10	2884	10.57
45	94.10	1590	5.83

#	m/z	Abs. Int.	Rel. Int.
46	95.10	5262	19.28
47	96.10	3931	14.41
48	97.10	10033	36.77
49	98.10	4457	16.33
50	99.10	4066	14.90
51	100.15	764	2.80
52	101.05	2915	10.68
53	102.10	1232	4.51
54	103.10	1238	4.54
55	104.10	533	1.95
56	105.10	2890	10.59
57	106.10	682	2.50
58	107.10	1997	7.32
59	108.15	1148	4.21
60	109.10	2621	9.60
61	110.10	2158	7.91
62	111.10	4684	17.16
63	112.10	1575	5.77
64	113.10	2248	8.24
65	114.10	525	1.92
66	115.10	4108	15.05
67	116.10	2191	8.03
68	117.10	1328	4.87
69	118.10	425	1.56
70	119.05	1256	4.60
71	120.10	535	1.96
72	121.10	1592	5.83
73	122.15	748	2.74
74	123.10	1800	6.60
75	124.10	1127	4.13
76	125.10	2143	7.85
77	126.10	1070	3.92
78	127.15	1436	5.26
79	128.15	661	2.42
80	129.10	9173	33.61
81	130.10	1602	5.87
82	131.15	927	3.40
83	132.10	294	1.08
84	133.10	1044	3.83
85	134.10	449	1.65
86	135.10	1219	4.47
87	136.10	721	2.64
88	137.15	886	3.25
89	138.10	723	2.65
90	139.10	1000	3.66

#	m/z	Abs. Int.	Rel. Int.
91	140.20	432	1.58
92	141.20	903	3.31
93	142.10	335	1.23
94	143.10	2362	8.66
95	144.10	670	2.46
96	145.10	705	2.58
97	146.10	184	0.67
98	147.05	906	3.32
99	148.10	369	1.35
100	149.10	1039	3.81
101	150.10	442	1.62
102	151.10	1015	3.72
103	152.15	639	2.34
104	153.15	775	2.84
105	154.20	444	1.63
106	155.10	595	2.18
107	156.10	153	0.56
108	157.15	1252	4.59
109	158.20	488	1.79
110	159.10	353	1.29
111	160.10	47	0.17
112	161.20	456	1.67
113	162.10	197	0.72
114	163.10	554	2.03
115	164.10	217	0.80
116	165.10	589	2.16
117	166.10	520	1.91
118	167.10	502	1.84
119	168.10	260	0.95
120	169.10	366	1.34
121	170.10	77	0.28
122	171.15	1774	6.50
123	172.15	449	1.65
124	173.10	286	1.05
125	174.20	64	0.23
126	175.10	330	1.21
127	176.10	119	0.44
128	177.00	444	1.63
129	178.10	201	0.74
130	179.10	1057	3.87
131	180.10	412	1.51
132	181.10	360	1.32
133	182.10	161	0.59
134	183.10	289	1.06
135	184.00	20	0.07

DEPTT. OF BOTANICAL & ENVIRONMENTAL SCIENCES, G.N.D.U. AMRITSAR

#	m/z	Abs. Int.	Rel. Int.
136	185.15	3259	11.94
137	186.15	500	1.83
138	187.10	259	0.95
139	188.10	21	0.08
140	189.20	299	1.10
141	190.10	29	0.11
142	191.10	400	1.47
143	192.10	305	1.12
144	193.10	320	1.17
145	194.20	117	0.43
146	195.10	213	0.78
147	196.20	120	0.44
148	197.00	103	0.38
149	198.10	13	0.05
150	199.15	1232	4.51
151	200.10	230	0.84
152	201.10	90	0.33
153	202.10	33	0.12
154	203.10	200	0.73
155	204.10	60	0.22
156	205.00	303	1.11
157	206.10	35	0.13
158	207.00	750	2.75
159	208.10	247	0.91
160	209.10	206	0.75
161	210.10	31	0.11
162	211.10	45	0.16
163	212.10	13	0.05

#	m/z	Abs. Int.	Rel. Int.
164	213.05	713	2.61
165	214.10	116	0.43
166	215.10	87	0.32
167	216.20	12	0.04
168	217.10	26	0.10
169	218.10	12	0.04
170	219.10	84	0.31
171	220.20	65	0.24
172	221.10	229	0.84
173	222.20	238	0.87
174	223.10	123	0.45
175	224.10	27	0.10
176	225.00	12	0.04
177	227.10	1007	3.69
178	228.10	199	0.73
179	229.10	34	0.12
180	231.10	14	0.05
181	233.00	70	0.26
182	235.10	71	0.26
183	236.10	30	0.11
184	237.10	12	0.04
185	239.00	12	0.04
186	241.10	1974	7.23
187	242.10	343	1.26
188	243.10	45	0.16
189	245.10	7	0.03
190	246.10	6	0.02
191	247.10	14	0.05

#	m/z	Abs. Int.	Rel. Int.
192	248.10	14	0.05
193	249.10	26	0.10
194	251.00	84	0.31
195	252.00	6	0.02
196	254.10	7	0.03
197	255.15	279	1.02
198	256.10	80	0.29
199	257.00	12	0.04
200	261.10	42	0.15
201	262.10	6	0.02
202	263.10	38	0.14
203	264.10	29	0.11
204	265.10	27	0.10
205	266.10	14	0.05
206	267.10	78	0.29
207	276.10	70	0.26
208	278.00	6	0.02
209	280.10	6	0.02
210	281.10	258	0.95
211	282.10	17	0.06
212	284.20	886	3.25
213	285.20	173	0.63
214	286.20	6	0.02
215	308.00	14	0.05
216	358.10	6	0.02
217	539.00	6	0.02
218	590.00	6	0.02
219	625.10	6	0.02

Line#:17 R.Time:21.1(Scan#:5130)
MassPeaks:204
RawMode:Averaged 21.1-21.1(5119-5144) BasePeak:57(46039)
BG Mode:None Group 1 - Event 1

#	m/z	Abs. Int.	Rel. Int.
1	50.00	690	1.50
2	51.05	1121	2.43
3	52.10	664	1.44
4	53.10	3048	6.62
5	54.10	5349	11.62
6	55.10	41002	89.06
7	56.10	19433	42.21
8	57.10	46039	100.00
9	58.10	2864	6.22
10	59.10	1007	2.19
11	60.05	2026	4.40
12	61.10	2617	5.68
13	62.10	335	0.73
14	63.10	748	1.62
15	64.10	411	0.89
16	65.05	1561	3.39
17	66.10	1533	3.33
18	67.05	11214	24.36
19	68.05	7444	16.17
20	69.10	34330	74.57
21	70.10	19682	42.75
22	71.10	24079	52.30
23	72.10	1856	4.03
24	73.05	3123	6.78
25	74.10	561	1.22
26	75.05	1149	2.50
27	76.10	476	1.03
28	77.05	2632	5.72
29	78.10	828	1.80
30	79.05	3448	7.49
31	80.10	1395	3.03
32	81.10	9144	19.86
33	82.10	13011	28.26
34	83.10	40014	86.91
35	84.10	12440	27.02
36	85.10	15564	33.81
37	86.10	1240	2.69
38	87.05	1015	2.20
39	88.10	327	0.71
40	89.00	697	1.51
41	90.10	209	0.45
42	91.05	3284	7.13
43	92.10	855	1.86
44	93.10	1653	3.59
45	94.05	1002	2.18
46	95.10	5391	11.71

#	m/z	Abs. Int.	Rel. Int.
47	96.10	8868	19.26
48	97.10	38993	84.70
49	98.15	8245	17.91
50	99.15	4501	9.78
51	100.10	865	1.88
52	101.05	811	1.76
53	102.10	340	0.74
54	103.10	614	1.33
55	104.10	346	0.75
56	105.10	1463	3.18
57	106.10	449	0.98
58	107.05	1187	2.58
59	108.00	677	1.47
60	109.10	2495	5.42
61	110.10	3952	8.58
62	111.10	18693	40.60
63	112.15	4555	9.89
64	113.15	2353	5.11
65	114.10	397	0.86
66	115.10	1267	2.75
67	116.10	631	1.37
68	117.10	1003	2.18
69	118.00	338	0.73
70	119.10	845	1.84
71	120.10	371	0.81
72	121.05	918	1.99
73	122.15	523	1.14
74	123.15	1787	3.88
75	124.15	2404	5.22
76	125.15	8096	17.59
77	126.15	2726	5.92
78	127.15	1417	3.08
79	128.10	537	1.17
80	129.05	1308	2.84
81	130.10	393	0.85
82	131.10	678	1.47
83	132.10	242	0.53
84	133.10	769	1.67
85	134.10	321	0.70
86	135.10	659	1.43
87	136.00	543	1.18
88	137.10	862	1.87
89	138.15	1330	2.89
90	139.15	3027	6.57
91	140.15	1362	2.96
92	141.15	862	1.87

#	m/z	Abs. Int.	Rel. Int.
93	142.20	233	0.51
94	143.05	867	1.88
95	144.10	246	0.53
96	145.10	542	1.18
97	146.10	206	0.45
98	147.10	604	1.31
99	148.10	288	0.63
100	149.10	733	1.59
101	150.10	253	0.55
102	151.10	745	1.62
103	152.15	976	2.12
104	153.20	1710	3.71
105	154.20	928	2.02
106	155.20	509	1.11
107	156.10	85	0.18
108	157.10	298	0.65
109	158.10	16	0.03
110	159.10	265	0.58
111	160.10	20	0.04
112	161.20	323	0.70
113	162.10	159	0.35
114	163.10	384	0.83
115	164.20	135	0.29
116	165.20	419	0.91
117	166.10	688	1.49
118	167.20	938	2.04
119	168.25	559	1.21
120	169.20	304	0.66
121	170.20	46	0.10
122	171.10	214	0.46
123	172.20	97	0.21
124	173.20	79	0.17
125	174.20	8	0.02
126	175.20	179	0.39
127	176.20	24	0.05
128	177.20	292	0.63
129	178.20	76	0.17
130	179.10	858	1.86
131	180.15	476	1.03
132	181.20	499	1.08
133	182.20	362	0.79
134	183.20	189	0.41
135	184.00	24	0.05
136	185.10	224	0.49
137	186.20	18	0.04
138	187.20	54	0.12

#	m/z	Abs. Int.	Rel. Int.	#	m/z	Abs. Int.	Rel. Int.	#	m/z	Abs. Int.	Rel. Int.
139	189.10	200	0.43	161	213.10	74	0.16	183	251.20	26	0.06
140	190.20	91	0.20	162	214.10	16	0.03	184	252.20	56	0.12
141	191.10	365	0.79	163	215.10	18	0.04	185	257.20	7	0.02
142	192.20	279	0.61	164	217.10	86	0.19	186	264.20	39	0.08
143	193.10	301	0.65	165	218.20	41	0.09	187	265.00	8	0.02
144	194.20	220	0.48	166	219.20	94	0.20	188	267.10	17	0.04
145	195.20	307	0.67	167	221.10	124	0.27	189	278.10	8	0.02
146	196.20	246	0.53	168	222.20	142	0.31	190	280.20	83	0.18
147	197.20	111	0.24	169	223.20	143	0.31	191	281.20	190	0.41
148	198.20	19	0.04	170	224.20	106	0.23	192	282.20	7	0.02
149	199.10	49	0.11	171	225.20	26	0.06	193	297.20	8	0.02
150	200.20	18	0.04	172	227.20	17	0.04	194	308.20	7	0.02
151	201.20	32	0.07	173	231.10	157	0.34	195	327.10	8	0.02
152	203.00	200	0.43	174	233.10	11	0.02	196	426.10	8	0.02
153	204.20	8	0.02	175	235.10	25	0.05	197	432.10	7	0.02
154	205.10	87	0.19	176	236.10	29	0.06	198	452.10	8	0.02
155	206.10	19	0.04	177	237.10	67	0.15	199	456.10	7	0.02
156	207.10	597	1.30	178	238.20	61	0.13	200	492.10	8	0.02
157	208.20	308	0.67	179	239.20	16	0.03	201	511.00	7	0.02
158	209.20	265	0.58	180	241.10	17	0.04	202	512.10	8	0.02
159	210.20	130	0.28	181	247.10	8	0.02	203	526.20	8	0.02
160	211.00	8	0.02	182	250.20	16	0.03	204	664.10	8	0.02

Line#:18 R.Time:22.8(Scan#:5645)
MassPeaks:209
RawMode:Averaged 22.8-22.9(5635-5656) BasePeak:57(31251)
BG Mode:None Group 1 - Event 1

#	m/z	Abs. Int.	Rel. Int.	#	m/z	Abs. Int.	Rel. Int.	#	m/z	Abs. Int.	Rel. Int.
1	50.00	628	2.01	53	102.10	255	0.82	105	154.15	690	2.21
2	51.10	933	2.99	54	103.20	609	1.95	106	155.10	502	1.61
3	52.10	559	1.79	55	104.10	357	1.14	107	156.10	40	0.13
4	53.10	2126	6.80	56	105.10	1520	4.86	108	157.10	231	0.74
5	54.10	3426	10.96	57	106.10	487	1.56	109	158.20	30	0.10
6	55.10	26231	83.94	58	107.10	1141	3.65	110	159.10	334	1.07
7	56.10	11814	37.80	59	108.00	736	2.36	111	160.10	19	0.06
8	57.10	31251	100.00	60	109.10	2113	6.76	112	161.10	346	1.11
9	58.10	2126	6.80	61	110.10	2926	9.36	113	162.10	177	0.57
10	59.05	957	3.06	62	111.10	13342	42.69	114	163.10	360	1.15
11	60.05	1413	4.52	63	112.15	3034	9.71	115	164.10	104	0.33
12	61.10	688	2.20	64	113.15	1902	6.09	116	165.10	449	1.44
13	62.00	308	0.99	65	114.20	320	1.02	117	166.10	554	1.77
14	63.00	526	1.68	66	115.05	1103	3.53	118	167.10	817	2.61
15	64.10	344	1.10	67	116.10	447	1.43	119	168.20	363	1.16
16	65.05	1243	3.98	68	117.10	1047	3.35	120	169.10	315	1.01
17	66.10	1134	3.63	69	118.00	338	1.08	121	170.10	20	0.06
18	67.05	7582	24.26	70	119.10	1028	3.29	122	171.20	206	0.66
19	68.10	4706	15.06	71	120.10	534	1.71	123	172.20	21	0.07
20	69.10	22561	72.19	72	121.10	929	2.97	124	173.20	160	0.51
21	70.10	12215	39.09	73	122.10	532	1.70	125	174.10	29	0.09
22	71.10	17681	56.58	74	123.10	1399	4.48	126	175.10	129	0.41
23	72.10	1471	4.71	75	124.15	1808	5.79	127	176.10	29	0.09
24	73.05	3307	10.58	76	125.15	6017	19.25	128	177.20	336	1.08
25	74.00	551	1.76	77	126.15	1952	6.25	129	178.10	134	0.43
26	75.00	1179	3.77	78	127.15	1252	4.01	130	179.00	805	2.58
27	76.10	425	1.36	79	128.10	534	1.71	131	180.10	375	1.20
28	77.10	2209	7.07	80	129.10	1079	3.45	132	181.15	486	1.56
29	78.10	747	2.39	81	130.10	442	1.41	133	182.10	234	0.75
30	79.10	2682	8.58	82	131.10	656	2.10	134	183.10	232	0.74
31	80.05	1088	3.48	83	132.10	234	0.75	135	185.10	174	0.56
32	81.10	6837	21.88	84	133.15	978	3.13	136	187.10	61	0.20
33	82.10	8715	27.89	85	134.10	411	1.32	137	188.20	20	0.06
34	83.10	25415	81.33	86	135.10	682	2.18	138	189.10	255	0.82
35	84.10	7870	25.18	87	136.10	548	1.75	139	190.10	60	0.19
36	85.10	11149	35.68	88	137.15	790	2.53	140	191.00	336	1.08
37	86.10	994	3.18	89	138.15	1102	3.53	141	192.20	250	0.80
38	87.00	875	2.80	90	139.15	2333	7.47	142	193.10	401	1.28
39	88.10	258	0.83	91	140.15	978	3.13	143	194.10	248	0.79
40	89.10	609	1.95	92	141.15	815	2.61	144	195.20	336	1.08
41	90.00	177	0.57	93	142.20	300	0.96	145	196.10	188	0.60
42	91.05	2575	8.24	94	143.10	903	2.89	146	197.10	135	0.43
43	92.05	768	2.46	95	144.10	206	0.66	147	199.00	42	0.13
44	93.10	1506	4.82	96	145.10	551	1.76	148	200.10	9	0.03
45	94.05	894	2.86	97	146.10	111	0.36	149	201.10	40	0.13
46	95.10	4303	13.77	98	147.10	758	2.43	150	202.10	9	0.03
47	96.10	6378	20.41	99	148.10	242	0.77	151	203.10	141	0.45
48	97.10	26583	85.06	100	149.10	738	2.36	152	204.10	9	0.03
49	98.10	5150	16.48	101	150.00	282	0.90	153	205.00	107	0.34
50	99.15	3814	12.20	102	151.10	759	2.43	154	206.10	12	0.04
51	100.10	836	2.68	103	152.10	766	2.45	155	207.00	1028	3.29
52	101.10	620	1.98	104	153.15	1363	4.36	156	208.10	427	1.37

DEPTT. OF BOTANICAL & ENVIRONMENTAL SCIENCES, G.N.D.U. AMRITSAR

#	m/z	Abs. Int.	Rel. Int.
157	209.10	289	0.92
158	210.00	119	0.38
159	211.10	29	0.09
160	212.10	19	0.06
161	213.10	56	0.18
162	215.10	30	0.10
163	216.10	11	0.04
164	219.10	69	0.22
165	221.10	155	0.50
166	222.10	80	0.26
167	223.00	239	0.76
168	224.00	120	0.38
169	225.00	41	0.13
170	226.10	11	0.04
171	227.20	29	0.09
172	228.10	45	0.14
173	229.00	21	0.07
174	230.00	22	0.07

#	m/z	Abs. Int.	Rel. Int.
175	231.20	9	0.03
176	236.00	32	0.10
177	237.20	34	0.11
178	238.10	9	0.03
179	239.10	68	0.22
180	243.10	9	0.03
181	245.00	10	0.03
182	247.00	20	0.06
183	248.00	30	0.10
184	249.10	19	0.06
185	250.10	21	0.07
186	251.10	40	0.13
187	252.20	25	0.08
188	253.10	62	0.20
189	255.10	22	0.07
190	264.10	9	0.03
191	265.00	38	0.12
192	266.00	19	0.06

#	m/z	Abs. Int.	Rel. Int.
193	267.00	161	0.52
194	269.10	9	0.03
195	276.10	19	0.06
196	281.10	428	1.37
197	282.20	61	0.20
198	283.10	24	0.08
199	302.10	9	0.03
200	354.10	9	0.03
201	355.00	41	0.13
202	359.10	10	0.03
203	391.00	21	0.07
204	399.00	9	0.03
205	421.10	9	0.03
206	642.10	9	0.03
207	657.20	10	0.03
208	661.20	10	0.03
209	671.00	10	0.03

Line#:19 R.Time:23.8(Scan#:5926)
MassPeaks:209
RawMode:Averaged 23.7-23.8(5914-5936) BasePeak:149(71252)
BG Mode:None Group 1 - Event 1

#	m/z	Abs. Int.	Rel. Int.
1	50.05	1644	2.31
2	51.10	1384	1.94
3	52.10	692	0.97
4	53.10	1895	2.66
5	54.10	1566	2.20
6	55.10	16127	22.63
7	56.10	6899	9.68
8	57.10	34785	48.82
9	58.10	1878	2.64
10	59.15	825	1.16
11	60.10	1242	1.74
12	61.10	698	0.98
13	62.10	239	0.34
14	63.10	578	0.81
15	64.10	398	0.56
16	65.05	3359	4.71
17	66.05	998	1.40
18	67.10	3850	5.40
19	68.10	2193	3.08
20	69.05	8541	11.99
21	70.10	18362	25.77
22	71.10	25619	35.96
23	72.10	1805	2.53
24	73.10	2945	4.13
25	74.05	1010	1.42
26	75.00	1676	2.35
27	76.05	3820	5.36
28	77.05	3251	4.56
29	78.05	779	1.09
30	79.10	2203	3.09
31	80.10	716	1.00
32	81.10	3654	5.13
33	82.10	4153	5.83
34	83.10	10238	14.37
35	84.10	5398	7.58
36	85.15	3495	4.91
37	86.10	462	0.65
38	87.10	770	1.08
39	88.10	260	0.36
40	89.10	765	1.07
41	90.10	203	0.28
42	91.05	2216	3.11
43	92.05	649	0.91
44	93.05	3788	5.32
45	94.05	927	1.30
46	95.10	2875	4.03
47	96.10	2158	3.03
48	97.10	3868	5.43
49	98.10	1313	1.84
50	99.10	1371	1.92
51	100.10	447	0.63
52	101.10	547	0.77
53	102.10	240	0.34
54	103.00	736	1.03
55	104.05	6017	8.44
56	105.05	3489	4.90

#	m/z	Abs. Int.	Rel. Int.
57	106.10	572	0.80
58	107.10	1211	1.70
59	108.10	622	0.87
60	109.10	1672	2.35
61	110.10	1473	2.07
62	111.15	1962	2.75
63	112.15	4243	5.95
64	113.15	8526	11.97
65	114.15	919	1.29
66	115.05	1029	1.44
67	116.10	535	0.75
68	117.05	1135	1.59
69	118.10	346	0.49
70	119.10	1065	1.49
71	120.10	480	0.67
72	121.05	3279	4.60
73	122.10	2066	2.90
74	123.10	1596	2.24
75	124.05	620	0.87
76	125.15	1043	1.46
77	126.10	576	0.81
78	127.10	733	1.03
79	128.10	489	0.69
80	129.05	1163	1.63
81	130.10	449	0.63
82	131.10	711	1.00
83	132.00	2007	2.82
84	133.05	1551	2.18
85	134.10	538	0.76
86	135.05	818	1.15
87	136.00	553	0.78
88	137.00	617	0.87
89	138.10	405	0.57
90	139.10	567	0.80
91	140.00	293	0.41
92	141.10	581	0.82
93	142.10	287	0.40
94	143.10	506	0.71
95	144.10	188	0.26
96	145.10	596	0.84
97	146.10	201	0.28
98	147.10	799	1.12
99	148.05	449	0.63
100	149.05	71252	100.00
101	150.05	7943	11.15
102	151.00	1616	2.27
103	152.10	421	0.59
104	153.10	420	0.59
105	154.10	213	0.30
106	155.10	360	0.51
107	156.10	60	0.08
108	157.10	345	0.48
109	158.00	133	0.19
110	159.10	353	0.50
111	160.00	139	0.20
112	161.00	336	0.47

#	m/z	Abs. Int.	Rel. Int.
113	162.05	714	1.00
114	163.00	574	0.81
115	164.10	169	0.24
116	165.10	462	0.65
117	166.15	398	0.56
118	167.05	21726	30.49
119	168.05	1982	2.78
120	169.00	461	0.65
121	170.00	8	0.01
122	171.00	235	0.33
123	172.10	11	0.02
124	173.00	155	0.22
125	174.00	9	0.01
126	175.00	336	0.47
127	176.00	118	0.17
128	177.00	384	0.54
129	178.10	187	0.26
130	179.05	809	1.14
131	180.00	313	0.44
132	181.10	230	0.32
133	182.10	19	0.03
134	183.10	120	0.17
135	184.10	9	0.01
136	185.10	177	0.25
137	187.00	100	0.14
138	188.10	32	0.04
139	189.10	313	0.44
140	190.10	80	0.11
141	191.00	539	0.76
142	192.00	423	0.59
143	193.00	437	0.61
144	194.10	71	0.10
145	195.10	132	0.19
146	196.00	58	0.08
147	197.00	17	0.02
148	198.00	19	0.03
149	199.00	9	0.01
150	200.10	9	0.01
151	201.00	60	0.08
152	202.10	27	0.04
153	203.00	326	0.46
154	204.00	37	0.05
155	205.10	118	0.17
156	206.00	21	0.03
157	207.00	1409	1.98
158	208.00	463	0.65
159	209.00	330	0.46
160	210.00	19	0.03
161	211.00	31	0.04
162	213.00	75	0.11
163	215.00	27	0.04
164	216.10	21	0.03
165	217.00	142	0.20
166	218.10	88	0.12
167	219.00	60	0.08
168	220.00	57	0.08

#	m/z	Abs. Int.	Rel. Int.	#	m/z	Abs. Int.	Rel. Int.	#	m/z	Abs. Int.	Rel. Int.
169	221.00	200	0.28	183	249.00	66	0.09	197	280.05	352	0.49
170	222.00	82	0.12	184	251.00	117	0.16	198	281.00	541	0.76
171	223.00	39	0.05	185	252.10	10	0.01	199	282.00	40	0.06
172	227.00	9	0.01	186	253.00	17	0.02	200	284.10	10	0.01
173	229.00	38	0.05	187	257.00	20	0.03	201	293.00	18	0.03
174	231.00	9	0.01	188	258.00	20	0.03	202	311.00	9	0.01
175	233.00	26	0.04	189	261.00	70	0.10	203	325.00	19	0.03
176	236.00	9	0.01	190	262.00	100	0.14	204	341.10	31	0.04
177	237.00	47	0.07	191	265.00	85	0.12	205	355.10	68	0.10
178	238.00	27	0.04	192	267.00	172	0.24	206	357.10	8	0.01
179	239.00	19	0.03	193	270.00	9	0.01	207	406.00	8	0.01
180	243.00	8	0.01	194	273.00	9	0.01	208	502.10	19	0.03
181	245.00	50	0.07	195	278.00	9	0.01	209	596.00	9	0.01
182	248.10	43	0.06	196	279.10	1691	2.37				

Line#:20 R.Time:24.4(Scan#:6128)
MassPeaks:212
RawMode:Averaged 24.4-24.5(6117-6139) BasePeak:57(20722)
BG Mode:None Group 1 - Event 1

#	m/z	Abs. Int.	Rel. Int.	#	m/z	Abs. Int.	Rel. Int.	#	m/z	Abs. Int.	Rel. Int.
1	50.00	568	2.74	61	110.10	2151	10.38	121	170.10	114	0.55
2	51.00	865	4.17	62	111.15	8725	42.11	122	171.10	246	1.19
3	52.00	534	2.58	63	112.15	1974	9.53	123	172.10	18	0.09
4	53.10	1648	7.95	64	113.15	1461	7.05	124	173.20	156	0.75
5	54.10	2199	10.61	65	114.10	255	1.23	125	174.10	31	0.15
6	55.10	16801	81.08	66	115.05	1204	5.81	126	175.20	226	1.09
7	56.10	7275	35.11	67	116.00	501	2.42	127	176.10	70	0.34
8	57.15	20722	100.00	68	117.10	976	4.71	128	177.10	351	1.69
9	58.15	1523	7.35	69	118.10	313	1.51	129	178.20	128	0.62
10	59.15	838	4.04	70	119.10	1220	5.89	130	179.10	734	3.54
11	60.10	1338	6.46	71	120.10	478	2.31	131	180.20	328	1.58
12	61.00	692	3.34	72	121.10	899	4.34	132	181.20	367	1.77
13	62.10	216	1.04	73	122.00	441	2.13	133	182.20	205	0.99
14	63.10	540	2.61	74	123.10	1558	7.52	134	183.20	208	1.00
15	64.10	307	1.48	75	124.15	1407	6.79	135	184.10	41	0.20
16	65.00	960	4.63	76	125.15	4347	20.98	136	185.00	183	0.88
17	66.10	852	4.11	77	126.15	1254	6.05	137	186.10	74	0.36
18	67.10	5160	24.90	78	127.10	1039	5.01	138	187.20	207	1.00
19	68.10	3182	15.36	79	128.20	571	2.76	139	188.20	9	0.04
20	69.10	14360	69.30	80	129.05	1104	5.33	140	189.20	369	1.78
21	70.10	7504	36.21	81	130.00	424	2.05	141	190.10	161	0.78
22	71.10	12005	57.93	82	131.10	738	3.56	142	191.10	547	2.64
23	72.10	1159	5.59	83	132.10	280	1.35	143	192.20	414	2.00
24	73.10	3441	16.61	84	133.05	1258	6.07	144	193.20	478	2.31
25	74.10	584	2.82	85	134.10	440	2.12	145	194.20	214	1.03
26	75.10	1248	6.02	86	135.00	722	3.48	146	195.10	222	1.07
27	76.00	443	2.14	87	136.10	618	2.98	147	196.10	125	0.60
28	77.00	2109	10.18	88	137.15	804	3.88	148	197.10	125	0.60
29	78.10	700	3.38	89	138.15	854	4.12	149	198.10	17	0.08
30	79.05	2183	10.53	90	139.15	1793	8.65	150	199.10	57	0.28
31	80.10	859	4.15	91	140.15	1075	5.19	151	201.10	114	0.55
32	81.10	5114	24.68	92	141.20	743	3.59	152	202.10	9	0.04
33	82.10	5676	27.39	93	142.20	284	1.37	153	203.10	304	1.47
34	83.10	15700	75.76	94	143.05	769	3.71	154	204.10	83	0.40
35	84.10	4807	23.20	95	144.10	240	1.16	155	205.20	93	0.45
36	85.10	7722	37.26	96	145.20	563	2.72	156	206.00	8	0.04
37	86.10	706	3.41	97	146.10	168	0.81	157	207.05	1881	9.08
38	87.10	733	3.54	98	147.10	795	3.84	158	208.00	525	2.53
39	88.10	302	1.46	99	148.10	285	1.38	159	209.00	443	2.14
40	89.10	633	3.05	100	149.10	715	3.45	160	210.00	131	0.63
41	90.10	175	0.84	101	150.10	325	1.57	161	211.00	259	1.25
42	91.05	2080	10.04	102	151.10	769	3.71	162	212.00	9	0.04
43	92.10	602	2.91	103	152.20	665	3.21	163	213.00	45	0.22
44	93.05	1323	6.38	104	153.20	1075	5.19	164	215.00	17	0.08
45	94.10	778	3.75	105	154.15	502	2.42	165	217.00	13	0.06
46	95.10	3448	16.64	106	155.20	451	2.18	166	218.00	88	0.42
47	96.10	4697	22.67	107	156.20	61	0.29	167	219.00	47	0.23
48	97.15	16866	81.39	108	157.10	341	1.65	168	220.00	9	0.04
49	98.10	3478	16.78	109	158.10	56	0.27	169	221.00	247	1.19
50	99.15	2692	12.99	110	159.20	318	1.53	170	222.00	89	0.43
51	100.10	705	3.40	111	160.20	114	0.55	171	223.00	131	0.63
52	101.10	560	2.70	112	161.20	322	1.55	172	225.00	37	0.18
53	102.10	284	1.37	113	162.10	235	1.13	173	227.00	18	0.09
54	103.10	706	3.41	114	163.10	416	2.01	174	229.00	50	0.24
55	104.10	312	1.51	115	164.10	177	0.85	175	231.00	29	0.14
56	105.10	1444	6.97	116	165.10	464	2.24	176	233.00	57	0.28
57	106.10	476	2.30	117	166.10	474	2.29	177	234.00	28	0.14
58	107.10	1178	5.68	118	167.20	662	3.19	178	235.00	20	0.10
59	108.10	616	2.97	119	168.20	386	1.86	179	236.00	26	0.13
60	109.15	1849	8.92	120	169.20	392	1.89	180	238.00	9	0.04

DEPTT. OF BOTANICAL & ENVIRONMENTAL SCIENCES, G.N.D.U. AMRITSAR

#	m/z	Abs. Int.	Rel. Int.
181	239.00	103	0.50
182	240.00	21	0.10
183	241.00	51	0.25
184	243.00	9	0.04
185	248.00	49	0.24
186	249.00	69	0.33
187	250.00	59	0.28
188	251.00	71	0.34
189	265.00	126	0.61
190	266.00	20	0.10
191	267.00	203	0.98
192	268.00	9	0.04
193	269.00	18	0.09
194	272.00	283	1.37
195	278.00	17	0.08
196	281.00	581	2.80
197	282.00	125	0.60
198	283.00	40	0.19
199	285.00	9	0.04
200	287.00	20	0.10
201	302.00	8	0.04
202	319.00	8	0.04
203	325.00	17	0.08
204	341.00	11	0.05
205	355.00	48	0.23
206	425.00	9	0.04
207	456.00	8	0.04
208	479.00	8	0.04
209	490.00	8	0.04
210	546.00	9	0.04
211	656.00	9	0.04
212	700.00	9	0.04

Line#:21 R.Time:25.4(Scan#:6419)
MassPeaks:231
RawMode:Averaged 25.3-25.4(6405-6434) BasePeak:57(57437)
BG Mode:None Group 1 - Event 1

#	m/z	Abs. Int.	Rel. Int.
1	50.00	544	0.95
2	51.00	721	1.26
3	52.00	493	0.86
4	53.10	1457	2.54
5	54.10	1660	2.89
6	55.10	15679	27.30
7	56.15	7548	13.14
8	57.10	57437	100.00
9	58.10	3910	6.81
10	59.10	2030	3.53
11	60.10	1158	2.02
12	61.10	623	1.08
13	62.10	221	0.38
14	63.10	468	0.81
15	64.10	281	0.49
16	65.05	891	1.55
17	66.05	652	1.14
18	67.10	3816	6.64
19	68.10	2134	3.72
20	69.10	11442	19.92
21	70.10	6857	11.94
22	71.10	41040	71.45
23	72.15	2668	4.65
24	73.05	3342	5.82
25	74.10	554	0.96
26	75.10	1304	2.27
27	76.00	410	0.71
28	77.10	1946	3.39
29	78.10	620	1.08
30	79.10	1988	3.46
31	80.10	702	1.22
32	81.10	3349	5.83
33	82.00	3225	5.61
34	83.10	9039	15.74
35	84.10	3855	6.71
36	85.10	29982	52.20
37	86.15	2246	3.91
38	87.10	653	1.14
39	88.10	285	0.50
40	89.10	626	1.09
41	90.10	64	0.11
42	91.10	2000	3.48
43	92.10	552	0.96
44	93.10	1382	2.41
45	94.10	703	1.22
46	95.10	2602	4.53
47	96.10	3124	5.44
48	97.10	8822	15.36
49	98.15	2931	5.10
50	99.15	13462	23.44
51	100.20	1231	2.14
52	101.20	516	0.90
53	102.10	207	0.36
54	103.10	640	1.11
55	104.20	293	0.51
56	105.10	1400	2.44
57	106.10	435	0.76
58	107.10	1268	2.21
59	108.10	531	0.92
60	109.10	1555	2.71
61	110.15	1625	2.83
62	111.15	4764	8.29
63	112.15	1920	3.34
64	113.15	8202	14.28
65	114.15	819	1.43
66	115.05	940	1.64
67	116.10	433	0.75
68	117.10	1063	1.85
69	118.10	289	0.50
70	119.10	1162	2.02
71	120.10	548	0.95
72	121.10	1036	1.80
73	122.10	458	0.80
74	123.05	1061	1.85
75	124.15	952	1.66
76	125.15	2572	4.48
77	126.15	1513	2.63
78	127.15	5453	9.49
79	128.10	905	1.58
80	129.05	1029	1.79
81	130.10	381	0.66
82	131.10	749	1.30
83	132.10	220	0.38
84	133.05	1308	2.28
85	134.00	472	0.82
86	135.10	684	1.19
87	136.10	451	0.79
88	137.10	512	0.89
89	138.10	728	1.27
90	139.20	1130	1.97
91	140.15	1082	1.88
92	141.15	3555	6.19
93	142.15	599	1.04
94	143.20	460	0.80
95	144.20	163	0.28
96	145.10	538	0.94
97	146.20	134	0.23
98	147.10	835	1.45
99	148.10	213	0.37
100	149.10	641	1.12
101	150.10	243	0.42
102	151.10	538	0.94
103	152.20	531	0.92
104	153.15	671	1.17
105	154.15	729	1.27
106	155.20	2188	3.81
107	156.20	349	0.61
108	157.20	287	0.50
109	158.20	60	0.10
110	159.10	340	0.59
111	160.20	30	0.05
112	161.10	325	0.57
113	162.20	142	0.25
114	163.20	403	0.70
115	164.20	101	0.18
116	165.10	365	0.64
117	166.20	423	0.74
118	167.20	495	0.86
119	168.20	460	0.80
120	169.20	1770	3.08
121	170.20	220	0.38
122	171.20	164	0.29
123	172.20	21	0.04
124	173.10	247	0.43
125	174.20	6	0.01
126	175.20	294	0.51
127	176.10	67	0.12
128	177.20	394	0.69
129	178.20	77	0.13
130	179.10	684	1.19
131	180.20	287	0.50
132	181.20	292	0.51
133	182.20	259	0.45
134	183.20	978	1.70
135	184.15	621	1.08
136	185.20	188	0.33
137	186.10	36	0.06
138	187.20	61	0.11
139	189.20	321	0.56
140	190.20	120	0.21
141	191.20	565	0.98
142	192.10	337	0.59
143	193.10	462	0.80
144	194.20	178	0.31
145	195.20	250	0.44
146	196.20	210	0.37
147	197.15	773	1.35
148	198.20	113	0.20
149	199.10	58	0.10
150	200.10	13	0.02
151	201.20	35	0.06
152	202.00	39	0.07
153	203.20	313	0.54
154	204.20	83	0.14
155	205.10	96	0.17
156	206.10	24	0.04
157	207.00	1930	3.36
158	208.00	542	0.94
159	209.00	399	0.69
160	210.00	300	0.52
161	211.20	516	0.90
162	212.20	97	0.17
163	213.10	53	0.09
164	215.00	6	0.01
165	216.00	23	0.04
166	217.00	7	0.01
167	218.10	135	0.24
168	219.00	89	0.15
169	220.20	22	0.04
170	221.00	169	0.29
171	222.00	37	0.06
172	223.10	237	0.41
173	224.20	113	0.20
174	225.20	376	0.65
175	226.20	37	0.06
176	227.20	16	0.03
177	229.00	7	0.01
178	231.20	24	0.04
179	232.00	6	0.01
180	234.00	6	0.01
181	235.00	29	0.05
182	236.20	14	0.02
183	237.20	59	0.10
184	238.20	96	0.17
185	239.20	298	0.52
186	240.20	64	0.11
187	241.20	15	0.03
188	242.00	25	0.04
189	243.00	6	0.01

DEPTT. OF BOTANICAL & ENVIRONMENTAL SCIENCES, G.N.D.U. AMRITSAR

#	m/z	Abs. Int.	Rel. Int.
190	245.20	7	0.01
191	248.00	9	0.02
192	249.10	186	0.32
193	250.00	15	0.03
194	251.00	40	0.07
195	252.20	100	0.17
196	253.20	213	0.37
197	254.10	13	0.02
198	257.00	21	0.04
199	258.00	7	0.01
200	259.00	7	0.01
201	262.10	6	0.01
202	263.10	7	0.01
203	264.20	7	0.01

#	m/z	Abs. Int.	Rel. Int.
204	265.00	107	0.19
205	266.20	58	0.10
206	267.20	368	0.64
207	268.20	17	0.03
208	269.00	20	0.03
209	273.20	7	0.01
210	280.00	15	0.03
211	281.00	815	1.42
212	282.00	194	0.34
213	283.00	102	0.18
214	285.00	6	0.01
215	289.00	14	0.02
216	295.00	15	0.03
217	296.00	6	0.01

#	m/z	Abs. Int.	Rel. Int.
218	297.00	14	0.02
219	305.00	6	0.01
220	323.00	7	0.01
221	325.00	14	0.02
222	337.00	14	0.02
223	341.00	15	0.03
224	355.00	166	0.29
225	356.00	6	0.01
226	421.00	6	0.01
227	514.00	8	0.01
228	549.00	13	0.02
229	634.00	7	0.01
230	664.00	7	0.01
231	693.10	6	0.01

Line#:22 R.Time:26.6(Scan#:6776)
MassPeaks:240
RawMode:Averaged 26.5-26.6(6759-6791) BasePeak:69(116492)
BG Mode:None Group 1 - Event 1

#	m/z	Abs. Int.	Rel. Int.
1	50.10	702	0.60
2	51.10	1229	1.06
3	52.10	723	0.62
4	53.10	5398	4.63
5	54.15	1084	0.93
6	55.15	10894	9.35
7	56.10	1600	1.37
8	57.10	6630	5.69
9	58.10	671	0.58
10	59.10	675	0.58
11	60.10	1088	0.93
12	61.10	653	0.56
13	62.10	228	0.20
14	63.10	565	0.49
15	64.10	333	0.29
16	65.10	1842	1.58
17	66.10	919	0.79
18	67.05	14627	12.56
19	68.10	13135	11.28
20	69.10	116492	100.00
21	70.10	7306	6.27
22	71.10	3663	3.14
23	72.10	442	0.38
24	73.10	3086	2.65
25	74.10	564	0.48
26	75.10	1264	1.09
27	76.10	457	0.39
28	77.05	4618	3.96
29	78.10	1241	1.07
30	79.10	7744	6.65
31	80.10	3226	2.77
32	81.10	70487	60.51
33	82.10	7729	6.63
34	83.10	4914	4.22
35	84.10	928	0.80
36	85.15	1769	1.52
37	86.10	246	0.21
38	87.10	558	0.48
39	88.10	219	0.19
40	89.10	576	0.49
41	90.10	112	0.10
42	91.05	6202	5.32
43	92.10	2466	2.12
44	93.10	14230	12.22
45	94.10	5444	4.67
46	95.10	23844	20.47
47	96.10	3402	2.92
48	97.10	3302	2.83
49	98.20	811	0.70
50	99.10	830	0.71
51	100.10	140	0.12
52	101.10	463	0.40
53	102.10	181	0.16
54	103.05	876	0.75
55	104.10	402	0.35
56	105.10	5069	4.35
57	106.10	1453	1.25
58	107.10	10144	8.71
59	108.10	2815	2.42
60	109.15	10158	8.72

#	m/z	Abs. Int.	Rel. Int.
61	110.15	1624	1.39
62	111.15	1955	1.68
63	112.10	400	0.34
64	113.10	466	0.40
65	114.10	49	0.04
66	115.05	1175	1.01
67	116.10	527	0.45
68	117.05	1431	1.23
69	118.10	420	0.36
70	119.10	4944	4.24
71	120.15	1628	1.40
72	121.10	13489	11.58
73	122.15	3250	2.79
74	123.15	8989	7.72
75	124.15	1399	1.20
76	125.15	1013	0.87
77	126.10	470	0.40
78	127.10	535	0.46
79	128.20	504	0.43
80	129.10	1069	0.92
81	130.10	453	0.39
82	131.15	1169	1.00
83	132.15	433	0.37
84	133.10	3686	3.16
85	134.10	2163	1.86
86	135.10	6123	5.26
87	136.15	8502	7.30
88	137.15	7766	6.67
89	138.15	1201	1.03
90	139.10	432	0.37
91	140.10	145	0.12
92	141.10	411	0.35
93	142.10	229	0.20
94	143.10	550	0.47
95	144.10	147	0.13
96	145.10	1126	0.97
97	146.10	371	0.32
98	147.10	3809	3.27
99	148.15	1377	1.18
100	149.15	6101	5.24
101	150.20	1256	1.08
102	151.10	847	0.73
103	152.10	309	0.27
104	153.20	325	0.28
105	154.20	157	0.13
106	155.10	329	0.28
107	156.10	129	0.11
108	157.10	407	0.35
109	158.20	150	0.13
110	159.10	874	0.75
111	160.10	245	0.21
112	161.15	2113	1.81
113	162.15	889	0.76
114	163.15	1627	1.40
115	164.20	343	0.29
116	165.20	511	0.44
117	166.20	271	0.23
118	167.20	227	0.19
119	168.20	62	0.05
120	169.00	181	0.16

#	m/z	Abs. Int.	Rel. Int.
121	170.20	73	0.06
122	171.20	329	0.28
123	172.20	79	0.07
124	173.20	698	0.60
125	174.10	240	0.21
126	175.15	1902	1.63
127	176.20	618	0.53
128	177.15	1292	1.11
129	178.20	379	0.33
130	179.20	655	0.56
131	180.10	181	0.16
132	181.10	129	0.11
133	182.20	21	0.02
134	183.10	75	0.06
135	184.10	19	0.02
136	185.20	224	0.19
137	186.20	7	0.01
138	187.20	442	0.38
139	188.10	167	0.14
140	189.15	1466	1.26
141	190.15	538	0.46
142	191.15	1553	1.33
143	192.10	908	0.78
144	193.10	590	0.51
145	194.20	225	0.19
146	195.10	88	0.08
147	199.20	118	0.10
148	201.10	321	0.28
149	202.10	191	0.16
150	203.15	1490	1.28
151	204.10	401	0.34
152	205.15	518	0.44
153	206.00	107	0.09
154	207.00	1989	1.71
155	208.00	541	0.46
156	209.00	374	0.32
157	210.00	77	0.07
158	211.00	13	0.01
159	213.00	91	0.08
160	215.00	277	0.24
161	216.00	193	0.17
162	217.10	502	0.43
163	218.20	493	0.42
164	219.10	235	0.20
165	220.20	22	0.02
166	221.10	200	0.17
167	222.00	6	0.01
168	223.00	135	0.12
169	224.00	6	0.01
170	225.00	7	0.01
171	226.00	6	0.01
172	227.00	84	0.07
173	228.00	34	0.03
174	229.00	297	0.25
175	230.20	78	0.07
176	231.15	457	0.39
177	232.10	115	0.10
178	233.00	62	0.05
179	235.00	20	0.02
180	239.20	12	0.01

DEPTT. OF BOTANICAL & ENVIRONMENTAL SCIENCES, G.N.D.U. AMRITSAR

#	m/z	Abs. Int.	Rel. Int.	#	m/z	Abs. Int.	Rel. Int.	#	m/z	Abs. Int.	Rel. Int.
181	241.20	55	0.05	201	274.10	37	0.03	221	346.00	7	0.01
182	243.10	151	0.13	202	281.00	705	0.61	222	355.00	46	0.04
183	244.10	63	0.05	203	282.00	111	0.10	223	367.20	129	0.11
184	245.20	59	0.05	204	283.00	138	0.12	224	368.20	30	0.03
185	247.00	6	0.01	205	284.20	19	0.02	225	395.10	23	0.02
186	248.00	45	0.04	206	285.00	63	0.05	226	400.00	6	0.01
187	249.00	132	0.11	207	286.20	21	0.02	227	402.00	6	0.01
188	251.00	26	0.02	208	297.20	42	0.04	228	410.00	19	0.02
189	252.00	6	0.01	209	298.00	6	0.01	229	411.20	14	0.01
190	255.00	33	0.03	210	299.20	113	0.10	230	442.00	6	0.01
191	257.20	210	0.18	211	300.10	14	0.01	231	444.00	6	0.01
192	258.20	82	0.07	212	309.00	6	0.01	232	453.00	6	0.01
193	259.20	115	0.10	213	311.20	12	0.01	233	479.00	7	0.01
194	260.20	21	0.02	214	325.20	77	0.07	234	570.00	6	0.01
195	265.00	170	0.15	215	327.20	13	0.01	235	593.00	6	0.01
196	266.90	225	0.19	216	328.10	40	0.03	236	638.00	6	0.01
197	269.20	55	0.05	217	332.00	6	0.01	237	645.00	6	0.01
198	271.20	81	0.07	218	341.20	276	0.24	238	647.10	6	0.01
199	272.10	36	0.03	219	342.00	67	0.06	239	674.00	6	0.01
200	273.20	129	0.11	220	343.00	6	0.01	240	676.00	6	0.01

Line#:23 R.Time:27.5(Scan#:7062)
MassPeaks:211
RawMode:Averaged 27.5-27.6(7045-7080) BasePeak:57(20267)
BG Mode:None Group 1 - Event 1

#	m/z	Abs. Int.	Rel. Int.	#	m/z	Abs. Int.	Rel. Int.	#	m/z	Abs. Int.	Rel. Int.
1	50.00	503	2.48	55	104.10	284	1.40	109	158.00	60	0.30
2	51.00	683	3.37	56	105.10	1622	8.00	110	159.10	364	1.80
3	52.00	393	1.94	57	106.10	478	2.36	111	160.10	72	0.36
4	53.15	1396	6.89	58	107.10	1457	7.19	112	161.20	421	2.08
5	54.15	1768	8.72	59	108.10	629	3.10	113	162.20	211	1.04
6	55.10	16091	79.40	60	109.15	2389	11.79	114	163.10	472	2.33
7	56.10	6658	32.85	61	110.15	2210	10.90	115	164.00	168	0.83
8	57.10	20267	100.00	62	111.15	8169	40.31	116	165.10	461	2.27
9	58.10	1207	5.96	63	112.15	1803	8.90	117	166.20	439	2.17
10	59.10	608	3.00	64	113.20	1349	6.66	118	167.20	676	3.34
11	60.10	1005	4.96	65	114.20	177	0.87	119	168.20	266	1.31
12	61.10	477	2.35	66	115.10	809	3.99	120	169.20	213	1.05
13	62.00	114	0.56	67	116.10	398	1.96	121	170.20	30	0.15
14	63.10	432	2.13	68	117.10	846	4.17	122	171.10	133	0.66
15	64.10	231	1.14	69	118.10	280	1.38	123	172.20	12	0.06
16	65.00	829	4.09	70	119.10	1348	6.65	124	173.10	260	1.28
17	66.10	872	4.30	71	120.10	520	2.57	125	174.20	30	0.15
18	67.10	5529	27.28	72	121.15	1149	5.67	126	175.20	451	2.23
19	68.10	5418	26.73	73	122.10	631	3.11	127	176.10	137	0.68
20	69.10	15207	75.03	74	123.10	1816	8.96	128	177.20	450	2.22
21	70.10	6593	32.53	75	124.20	1615	7.97	129	178.10	123	0.61
22	71.10	11041	54.48	76	125.15	4216	20.80	130	179.10	497	2.45
23	72.10	878	4.33	77	126.20	1236	6.10	131	180.20	271	1.34
24	73.10	3277	16.17	78	127.15	989	4.88	132	181.20	368	1.82
25	74.10	483	2.38	79	128.20	498	2.46	133	182.20	194	0.96
26	75.05	959	4.73	80	129.10	808	3.99	134	183.20	152	0.75
27	76.00	350	1.73	81	130.10	317	1.56	135	184.10	25	0.12
28	77.05	1965	9.70	82	131.15	701	3.46	136	185.00	172	0.85
29	78.10	592	2.92	83	132.20	224	1.11	137	186.20	5	0.02
30	79.10	2217	10.94	84	133.05	1454	7.17	138	187.20	139	0.69
31	80.10	839	4.14	85	134.10	534	2.63	139	189.20	527	2.60
32	81.10	5751	28.38	86	135.10	926	4.57	140	190.20	222	1.10
33	82.10	7966	39.31	87	136.10	569	2.81	141	191.10	631	3.11
34	83.10	15623	77.09	88	137.15	995	4.91	142	192.10	295	1.46
35	84.10	4264	21.04	89	138.15	1036	5.11	143	193.10	414	2.04
36	85.15	6965	34.37	90	139.20	1870	9.23	144	194.20	206	1.02
37	86.10	615	3.03	91	140.20	615	3.03	145	195.20	277	1.37
38	87.10	550	2.71	92	141.15	712	3.51	146	196.20	96	0.47
39	88.10	301	1.49	93	142.10	209	1.03	147	197.20	78	0.38
40	89.10	478	2.36	94	143.20	370	1.83	148	199.10	29	0.14
41	90.10	87	0.43	95	144.20	128	0.63	149	201.00	59	0.29
42	91.10	2085	10.29	96	145.20	532	2.62	150	202.10	56	0.28
43	92.10	497	2.45	97	146.00	128	0.63	151	203.20	696	3.43
44	93.10	1655	8.17	98	147.05	952	4.70	152	204.10	210	1.04
45	94.10	848	4.18	99	148.20	302	1.49	153	205.20	216	1.07
46	95.10	4292	21.18	100	149.20	682	3.37	154	206.10	30	0.15
47	96.10	5489	27.08	101	150.10	315	1.55	155	206.95	1885	9.30
48	97.15	15718	77.55	102	151.20	586	2.89	156	208.00	535	2.64
49	98.15	3004	14.82	103	152.20	598	2.95	157	209.00	410	2.02
50	99.15	2529	12.48	104	153.20	1092	5.39	158	210.00	78	0.38
51	100.10	337	1.66	105	154.20	359	1.77	159	211.00	74	0.37
52	101.10	411	2.03	106	155.20	447	2.21	160	213.00	37	0.18
53	102.20	204	1.01	107	156.20	82	0.40	161	215.00	11	0.05
54	103.10	588	2.90	108	157.20	301	1.49	162	216.00	11	0.05

DEPTT. OF BOTANICAL & ENVIRONMENTAL SCIENCES, G.N.D.U. AMRITSAR

#	m/z	Abs. Int.	Rel. Int.
163	217.00	18	0.09
164	218.00	585	2.89
165	219.00	227	1.12
166	220.00	12	0.06
167	221.00	229	1.13
168	222.00	23	0.11
169	223.00	136	0.67
170	224.00	30	0.15
171	225.00	20	0.10
172	227.00	17	0.08
173	229.00	12	0.06
174	231.00	13	0.06
175	232.00	5	0.02
176	233.00	5	0.02
177	235.00	25	0.12
178	236.00	12	0.06
179	237.00	49	0.24
180	238.00	21	0.10
181	242.00	6	0.03
182	248.00	32	0.16
183	249.00	122	0.60
184	250.00	12	0.06
185	251.00	55	0.27
186	252.00	11	0.05
187	253.00	31	0.15
188	255.00	6	0.03
189	256.00	18	0.09
190	265.00	150	0.74
191	266.00	24	0.12
192	267.00	215	1.06
193	268.00	12	0.06
194	273.00	6	0.03
195	281.00	652	3.22
196	282.00	195	0.96
197	283.00	67	0.33
198	325.00	12	0.06
199	341.00	17	0.08
200	342.00	6	0.03
201	355.00	85	0.42
202	386.00	5	0.02
203	399.00	5	0.02
204	406.00	5	0.02
205	417.00	6	0.03
206	538.00	5	0.02
207	614.00	6	0.03
208	615.00	5	0.02
209	636.00	5	0.02
210	661.00	6	0.03
211	664.00	5	0.02

Line#:24 R.Time:27.8(Scan#:7131)
MassPeaks:226
RawMode:Averaged 27.7-27.8(7116-7146) BasePeak:57(52141)
BG Mode:None Group 1 - Event 1

#	m/z	Abs. Int.	Rel. Int.
1	50.00	500	0.96
2	51.00	723	1.39
3	52.00	417	0.80
4	53.15	1590	3.05
5	54.15	1583	3.04
6	55.10	15321	29.38
7	56.10	6897	13.23
8	57.10	52141	100.00
9	58.15	6517	12.50
10	59.10	5664	10.86
11	60.10	1123	2.15
12	61.10	452	0.87
13	62.10	136	0.26
14	63.10	443	0.85
15	64.10	309	0.59
16	65.05	815	1.56
17	66.05	631	1.21
18	67.10	4295	8.24
19	68.10	2501	4.80
20	69.10	12882	24.71
21	70.10	6343	12.17
22	71.10	40555	77.78
23	72.10	2508	4.81
24	73.10	3060	5.87
25	74.10	461	0.88
26	75.05	1084	2.08
27	76.10	317	0.61
28	77.05	2064	3.96
29	78.00	654	1.25
30	79.10	2252	4.32
31	80.10	805	1.54
32	81.10	5110	9.80
33	82.10	4061	7.79
34	83.10	9548	18.31
35	84.15	3743	7.18
36	85.10	28475	54.61
37	86.15	2093	4.01
38	87.10	559	1.07
39	88.10	148	0.28
40	89.10	479	0.92
41	90.10	81	0.16
42	91.10	2253	4.32
43	92.10	586	1.12
44	93.10	2057	3.95
45	94.10	953	1.83
46	95.10	3955	7.59
47	96.15	4086	7.84
48	97.15	9178	17.60
49	98.15	2782	5.34
50	99.15	12254	23.50
51	100.15	1267	2.43
52	101.10	425	0.82
53	102.10	108	0.21
54	103.10	582	1.12
55	104.20	251	0.48
56	105.10	1687	3.24
57	106.10	464	0.89
58	107.10	1759	3.37
59	108.15	750	1.44
60	109.10	2534	4.86
61	110.10	1885	3.62
62	111.15	5103	9.79
63	112.15	1742	3.34
64	113.15	7446	14.28
65	114.20	921	1.77
66	115.10	842	1.61
67	116.10	360	0.69
68	117.10	933	1.79
69	118.20	311	0.60
70	119.10	1625	3.12
71	120.10	548	1.05
72	121.10	1490	2.86
73	122.10	611	1.17
74	123.15	1678	3.22
75	124.15	1319	2.53
76	125.15	2773	5.32
77	126.15	1388	2.66
78	127.15	5290	10.15
79	128.15	902	1.73
80	129.10	903	1.73
81	130.10	371	0.71
82	131.10	779	1.49
83	132.10	290	0.56
84	133.05	1615	3.10
85	134.10	577	1.11
86	135.10	1104	2.12
87	136.10	643	1.23
88	137.10	945	1.81
89	138.15	852	1.63
90	139.15	1255	2.41
91	140.15	855	1.64
92	141.20	3321	6.37
93	142.20	576	1.10
94	143.10	460	0.88
95	144.20	82	0.16
96	145.10	598	1.15
97	146.20	204	0.39
98	147.10	1142	2.19
99	148.10	381	0.73
100	149.10	758	1.45
101	150.10	352	0.68
102	151.10	608	1.17
103	152.20	620	1.19
104	153.20	838	1.61
105	154.15	647	1.24
106	155.20	2021	3.88
107	156.20	369	0.71
108	157.20	344	0.66
109	158.20	28	0.05
110	159.10	467	0.90
111	160.10	134	0.26
112	161.20	560	1.07
113	162.20	233	0.45
114	163.10	461	0.88
115	164.20	74	0.14
116	165.10	450	0.86
117	166.20	401	0.77
118	167.20	557	1.07
119	168.20	418	0.80
120	169.25	1333	2.56
121	170.20	232	0.44
122	171.20	216	0.41
123	172.20	6	0.01
124	173.10	300	0.58
125	174.20	21	0.04
126	175.20	485	0.93
127	176.10	239	0.46
128	177.20	421	0.81
129	178.10	90	0.17
130	179.20	461	0.88
131	180.20	258	0.49
132	181.20	288	0.55
133	182.20	334	0.64
134	183.20	1042	2.00
135	184.20	196	0.38
136	185.10	157	0.30
137	187.10	243	0.47
138	188.20	31	0.06
139	189.20	614	1.18
140	190.20	269	0.52
141	191.10	617	1.18
142	192.20	280	0.54
143	193.10	419	0.80
144	194.10	180	0.35
145	195.20	282	0.54
146	196.20	214	0.41
147	197.20	729	1.40
148	198.20	115	0.22
149	199.20	68	0.13
150	200.20	14	0.03
151	201.10	99	0.19
152	202.10	36	0.07
153	203.10	854	1.64
154	204.20	240	0.46
155	205.20	220	0.42
156	206.00	6	0.01
157	207.00	2071	3.97
158	208.00	559	1.07
159	209.00	427	0.82
160	210.00	162	0.31
161	211.15	512	0.98
162	212.20	89	0.17
163	213.00	59	0.11
164	215.10	28	0.05
165	217.00	30	0.06
166	218.15	716	1.37
167	219.00	263	0.50
168	220.20	7	0.01
169	221.10	284	0.54
170	222.00	89	0.17
171	223.20	148	0.28

DEPTT. OF BOTANICAL & ENVIRONMENTAL SCIENCES, G.N.D.U. AMRITSAR

#	m/z	Abs. Int.	Rel. Int.
172	224.00	102	0.20
173	225.10	372	0.71
174	226.00	45	0.09
175	227.00	10	0.02
176	229.00	48	0.09
177	230.00	7	0.01
178	231.00	16	0.03
179	235.00	38	0.07
180	236.10	28	0.05
181	237.00	21	0.04
182	238.20	51	0.10
183	239.10	318	0.61
184	240.20	24	0.05
185	241.00	29	0.06
186	243.00	6	0.01
187	245.00	7	0.01
188	248.20	6	0.01
189	249.00	79	0.15
190	251.00	56	0.11
191	252.20	32	0.06
192	253.20	200	0.38
193	254.00	6	0.01
194	255.10	21	0.04
195	259.10	21	0.04
196	260.00	6	0.01
197	265.00	141	0.27
198	266.10	20	0.04
199	267.20	374	0.72
200	268.10	34	0.07
201	269.10	14	0.03
202	270.20	13	0.02
203	280.00	7	0.01
204	281.00	741	1.42
205	282.10	175	0.34
206	283.00	76	0.15
207	285.00	13	0.02
208	295.20	24	0.05
209	296.00	7	0.01
210	309.10	6	0.01
211	325.00	13	0.02
212	326.00	13	0.02
213	327.00	20	0.04
214	331.00	6	0.01
215	341.00	23	0.04
216	343.00	6	0.01
217	355.00	51	0.10
218	371.00	7	0.01
219	405.00	6	0.01
220	457.20	7	0.01
221	466.10	7	0.01
222	478.10	6	0.01
223	497.00	6	0.01
224	538.00	7	0.01
225	576.00	7	0.01
226	667.00	7	0.01

Line#:25 R.Time:31.2(Scan#:8148)
MassPeaks:290
RawMode:Averaged 31.0-31.3(8111-8187) BasePeak:165(56546)
BG Mode:None Group 1 - Event 1

#	m/z	Abs. Int.	Rel. Int.
1	50.00	555	0.98
2	51.10	921	1.63
3	52.10	551	0.97
4	53.10	2341	4.14
5	54.15	2586	4.57
6	55.15	27417	48.49
7	56.15	10378	18.35
8	57.15	36773	65.03
9	58.15	1991	3.52
10	59.10	607	1.07
11	60.10	1006	1.78
12	61.10	443	0.78
13	62.10	183	0.32
14	63.10	492	0.87
15	64.10	290	0.51
16	65.10	1276	2.26
17	66.10	1236	2.19
18	67.10	9537	16.87
19	68.10	7744	13.70
20	69.10	23188	41.01
21	70.10	9245	16.35
22	71.10	19574	34.62
23	72.15	1332	2.36
24	73.05	3076	5.44
25	74.10	507	0.90
26	75.10	795	1.41
27	76.10	365	0.65
28	77.05	2982	5.27
29	78.10	852	1.51
30	79.10	3943	6.97
31	80.05	1275	2.25
32	81.10	9765	17.27
33	82.10	12160	21.50
34	83.10	23756	42.01
35	84.10	6057	10.71
36	85.10	10998	19.45
37	86.10	905	1.60
38	87.10	536	0.95
39	88.10	154	0.27
40	89.10	472	0.83
41	90.10	109	0.19
42	91.10	4897	8.66
43	92.10	844	1.49
44	93.10	3453	6.11
45	94.10	1491	2.64
46	95.10	7328	12.96
47	96.10	8367	14.80
48	97.10	23646	41.82
49	98.15	4162	7.36
50	99.10	4130	7.30
51	100.15	424	0.75
52	101.10	358	0.63
53	102.20	271	0.48
54	103.05	802	1.42
55	104.10	409	0.72
56	105.05	3222	5.70
57	106.15	763	1.35
58	107.10	3519	6.22
59	108.10	1256	2.22
60	109.10	5341	9.45
61	110.10	3492	6.18
62	111.15	12586	22.26
63	112.15	2566	4.54
64	113.15	2173	3.84
65	114.00	278	0.49
66	115.05	1274	2.25
67	116.10	526	0.93
68	117.05	1396	2.47
69	118.10	449	0.79
70	119.10	3413	6.04
71	120.10	989	1.75
72	121.10	6885	12.18
73	122.10	2160	3.82
74	123.10	2786	4.93
75	124.15	2542	4.50
76	125.15	6767	11.97
77	126.15	1681	2.97
78	127.15	1558	2.76
79	128.10	795	1.41
80	129.10	1119	1.98
81	130.10	406	0.72
82	131.10	1354	2.39
83	132.10	444	0.79
84	133.10	2289	4.05
85	134.10	997	1.76
86	135.10	3227	5.71
87	136.10	4989	8.82
88	137.10	5200	9.20
89	138.15	2336	4.13
90	139.15	3247	5.74
91	140.20	993	1.76
92	141.20	1121	1.98
93	142.10	408	0.72
94	143.10	621	1.10
95	144.10	345	0.61
96	145.10	1164	2.06
97	146.10	491	0.87
98	147.15	2314	4.09
99	148.10	757	1.34
100	149.10	2733	4.83
101	150.10	622	1.10
102	151.10	1401	2.48
103	152.15	2119	3.75
104	153.20	1961	3.47
105	154.15	726	1.28
106	155.20	730	1.29
107	156.20	245	0.43
108	157.20	519	0.92
109	158.10	214	0.38
110	159.10	1064	1.88
111	160.10	402	0.71
112	161.10	1424	2.52
113	162.15	1144	2.02
114	163.10	1455	2.57
115	164.10	23530	41.61
116	165.10	56546	100.00
117	166.10	7561	13.37
118	167.15	1844	3.26
119	168.20	574	1.02
120	169.25	458	0.81
121	170.20	74	0.13
122	171.20	368	0.65
123	172.10	209	0.37
124	173.10	620	1.10
125	174.10	329	0.58
126	175.10	1426	2.52
127	176.10	982	1.74
128	177.10	1980	3.50
129	178.10	822	1.45
130	179.10	752	1.33
131	180.15	459	0.81
132	181.15	689	1.22
133	182.20	328	0.58
134	183.20	346	0.61
135	184.20	55	0.10
136	185.20	255	0.45
137	186.20	106	0.19
138	187.10	777	1.37
139	188.10	347	0.61
140	189.10	1948	3.44
141	190.05	1211	2.14
142	191.05	1645	2.91
143	192.10	419	0.74
144	193.10	480	0.85
145	194.15	387	0.68
146	195.10	527	0.93
147	196.20	191	0.34
148	197.20	230	0.41
149	198.20	26	0.05
150	199.10	134	0.24
151	200.10	19	0.03
152	201.10	365	0.65
153	202.10	190	0.34
154	203.10	2406	4.25
155	204.15	774	1.37
156	205.05	6921	12.24
157	206.10	1074	1.90
158	207.00	2372	4.19
159	208.10	740	1.31
160	209.10	561	0.99
161	210.10	204	0.36
162	211.00	181	0.32
163	212.00	23	0.04
164	213.00	157	0.28
165	214.00	34	0.06

DEPTT. OF BOTANICAL & ENVIRONMENTAL SCIENCES, G.N.D.U. AMRITSAR

#	m/z	Abs. Int.	Rel. Int.
166	215.00	243	0.43
167	216.00	88	0.16
168	217.10	244	0.43
169	218.15	1796	3.18
170	219.10	750	1.33
171	220.10	157	0.28
172	221.10	322	0.57
173	222.10	240	0.42
174	223.10	319	0.56
175	224.10	143	0.25
176	225.10	97	0.17
177	226.10	2	0.00
178	227.10	53	0.09
179	228.10	2	0.00
180	229.10	179	0.32
181	230.10	26	0.05
182	231.10	103	0.18
183	232.10	32	0.06
184	233.20	50	0.09
185	234.10	12	0.02
186	235.00	20	0.04
187	236.10	137	0.24
188	237.20	159	0.28
189	238.10	83	0.15
190	239.10	133	0.24
191	240.10	33	0.06
192	241.10	66	0.12
193	243.00	23	0.04
194	245.10	39	0.07
195	246.10	36	0.06
196	247.20	11	0.02
197	248.00	89	0.16
198	249.10	135	0.24
199	250.10	92	0.16
200	251.10	156	0.28
201	252.10	58	0.10
202	253.00	86	0.15
203	254.10	8	0.01
204	255.10	90	0.16
205	256.10	5	0.01
206	257.10	53	0.09
207	258.10	8	0.01

#	m/z	Abs. Int.	Rel. Int.
208	259.10	17	0.03
209	260.10	60	0.11
210	261.10	21	0.04
211	262.10	2	0.00
212	263.10	2	0.00
213	264.10	73	0.13
214	265.10	208	0.37
215	266.00	60	0.11
216	267.10	238	0.42
217	268.20	5	0.01
218	269.10	26	0.05
219	271.10	23	0.04
220	273.20	5	0.01
221	274.20	64	0.11
222	275.10	5	0.01
223	278.10	41	0.07
224	279.10	37	0.07
225	280.10	16	0.03
226	281.10	689	1.22
227	282.10	183	0.32
228	283.10	120	0.21
229	284.10	15	0.03
230	285.00	6	0.01
231	287.10	2	0.00
232	288.10	108	0.19
233	289.10	25	0.04
234	291.10	9	0.02
235	292.10	11	0.02
236	294.10	5	0.01
237	295.10	5	0.01
238	298.20	46	0.08
239	299.20	2	0.00
240	301.20	3	0.01
241	302.10	58	0.10
242	303.20	6	0.01
243	306.10	9	0.02
244	308.10	2	0.00
245	309.10	6	0.01
246	316.10	17	0.03
247	322.20	2	0.00
248	325.10	3	0.01
249	327.20	8	0.01

#	m/z	Abs. Int.	Rel. Int.
250	328.10	2	0.00
251	330.10	14	0.02
252	332.00	8	0.01
253	339.10	2	0.00
254	341.10	25	0.04
255	342.10	32	0.06
256	343.10	2	0.00
257	344.10	46	0.08
258	347.10	2	0.00
259	350.20	2	0.00
260	355.10	72	0.13
261	358.10	8	0.01
262	362.00	8	0.01
263	364.00	2	0.00
264	367.20	2	0.00
265	371.10	3	0.01
266	384.00	6	0.01
267	387.10	97	0.17
268	388.10	25	0.04
269	390.10	3	0.01
270	408.20	2	0.00
271	410.20	2	0.00
272	413.10	2	0.00
273	415.10	8	0.01
274	416.00	5	0.01
275	417.10	5	0.01
276	428.10	12	0.02
277	429.20	32	0.06
278	430.20	9605	16.99
279	431.25	2917	5.16
280	432.20	517	0.91
281	433.20	34	0.06
282	490.20	2	0.00
283	504.20	2	0.00
284	548.20	2	0.00
285	565.20	2	0.00
286	568.20	2	0.00
287	588.20	8	0.01
288	619.30	5	0.01
289	660.20	2	0.00
290	684.20	2	0.00

Line#:26 R.Time:33.7(Scan#:8920)
MassPeaks:361
RawMode:Averaged 33.5-33.8(8836-8940) BasePeak:137(156045)
BG Mode:None Group 1 - Event 1

#	m/z	Abs. Int.	Rel. Int.
1	50.05	638	0.41
2	51.10	1237	0.79
3	52.15	747	0.48
4	53.10	8623	5.53
5	54.15	2872	1.84
6	55.10	67042	42.96
7	56.10	6701	4.29
8	57.10	23472	15.04
9	58.10	1345	0.86
10	59.10	1218	0.78
11	60.10	952	0.61
12	61.10	474	0.30
13	62.10	241	0.15
14	63.10	561	0.36
15	64.10	385	0.25
16	65.05	3966	2.54
17	66.15	1887	1.21
18	67.10	50966	32.66
19	68.10	10437	6.69
20	69.10	78696	50.43
21	70.10	6812	4.37
22	71.10	9246	5.93
23	72.05	889	0.57
24	73.10	2742	1.76
25	74.00	553	0.35
26	75.00	712	0.46
27	76.10	403	0.26
28	77.05	10086	6.46
29	78.10	2181	1.40
30	79.10	28803	18.46
31	80.10	4944	3.17
32	81.10	57819	37.05

#	m/z	Abs. Int.	Rel. Int.
33	82.10	15248	9.77
34	83.10	28041	17.97
35	84.10	3861	2.47
36	85.10	4582	2.94
37	86.10	566	0.36
38	87.10	763	0.49
39	88.05	509	0.33
40	89.10	564	0.36
41	90.10	196	0.13
42	91.05	24048	15.41
43	92.10	3930	2.52
44	93.10	37070	23.76
45	94.10	8789	5.63
46	95.10	79687	51.07
47	96.10	15660	10.04
48	97.10	15422	9.88
49	98.10	2063	1.32
50	99.10	1542	0.99
51	100.10	471	0.30
52	101.15	715	0.46
53	102.05	542	0.35
54	103.05	1749	1.12
55	104.10	883	0.57
56	105.10	23886	15.31
57	106.10	5523	3.54
58	107.10	42313	27.12
59	108.10	15595	9.99
60	109.10	58543	37.52
61	110.10	10537	6.75
62	111.10	16026	10.27
63	112.10	1934	1.24
64	113.10	2121	1.36

#	m/z	Abs. Int.	Rel. Int.
65	114.15	611	0.39
66	115.05	2540	1.63
67	116.05	1228	0.79
68	117.10	5206	3.34
69	118.15	1625	1.04
70	119.10	22551	14.45
71	120.10	7136	4.57
72	121.10	36181	23.19
73	122.10	10131	6.49
74	123.10	40161	25.74
75	124.15	7593	4.87
76	125.15	9508	6.09
77	126.15	2090	1.34
78	127.10	1188	0.76
79	128.10	1879	1.20
80	129.05	3706	2.37
81	130.05	1480	0.95
82	131.10	7923	5.08
83	132.10	2289	1.47
84	133.10	16662	10.68
85	134.10	6145	3.94
86	135.10	22815	14.62
87	136.10	14967	9.59
88	137.10	156045	100.00
89	138.10	24415	15.65
90	139.15	7131	4.57
91	140.10	1657	1.06
92	141.10	1437	0.92
93	142.10	1851	1.19
94	143.10	3299	2.11
95	144.10	1422	0.91
96	145.10	9035	5.79

DEPTT. OF BOTANICAL & ENVIRONMENTAL SCIENCES, G.N.D.U. AMRITSAR

#	m/z	Abs. Int.	Rel. Int.
97	146.10	2702	1.73
98	147.10	14526	9.31
99	148.15	5175	3.32
100	149.10	20384	13.06
101	150.10	11812	7.57
102	151.15	12479	8.00
103	152.15	5276	3.38
104	153.15	3619	2.32
105	154.15	784	0.50
106	155.10	1102	0.71
107	156.15	1400	0.90
108	157.10	2972	1.90
109	158.10	1120	0.72
110	159.10	7130	4.57
111	160.10	2009	1.29
112	161.10	10215	6.55
113	162.15	3811	2.44
114	163.15	15888	10.18
115	164.15	4561	2.92
116	165.15	4292	2.75
117	166.20	1256	0.80
118	167.15	965	0.62
119	168.10	362	0.23
120	169.10	839	0.54
121	170.15	685	0.44
122	171.10	2599	1.67
123	172.10	914	0.59
124	173.15	5933	3.80
125	174.15	1765	1.13
126	175.15	8404	5.39
127	176.15	3298	2.11
128	177.15	9611	6.16
129	178.10	2585	1.66
130	179.10	5735	3.68
131	180.10	1085	0.70
132	181.10	617	0.40
133	182.10	260	0.17
134	183.10	685	0.44
135	184.15	353	0.23
136	185.05	2322	1.49
137	186.10	776	0.50
138	187.10	4613	2.96
139	188.10	1827	1.17
140	189.10	8507	5.45
141	190.15	3229	2.07
142	191.10	14811	9.49
143	192.10	3803	2.44
144	193.10	2687	1.72
145	194.05	619	0.40
146	195.05	420	0.27
147	196.10	134	0.09
148	197.10	596	0.38
149	198.10	459	0.29
150	199.10	2172	1.39
151	200.05	645	0.41
152	201.10	3081	1.97
153	202.15	1574	1.01
154	203.10	8103	5.19
155	204.15	4313	2.76
156	205.10	17590	11.27
157	206.15	6229	3.99
158	207.10	5666	3.63
159	208.10	1245	0.80
160	209.05	611	0.39
161	210.10	132	0.08
162	211.10	472	0.30
163	212.10	221	0.14
164	213.05	1541	0.99
165	214.05	683	0.44
166	215.10	3175	2.03
167	216.10	1196	0.77
168	217.10	4599	2.95
169	218.10	7148	4.58
170	219.10	6454	4.14
171	220.10	2029	1.30
172	221.10	1155	0.74
173	222.10	329	0.21
174	223.10	300	0.19
175	224.10	58	0.04
176	225.10	308	0.20

#	m/z	Abs. Int.	Rel. Int.
177	226.10	156	0.10
178	227.05	1170	0.75
179	228.05	424	0.27
180	229.10	2516	1.61
181	230.10	792	0.51
182	231.10	3251	2.08
183	232.10	1211	0.78
184	233.10	1169	0.75
185	234.05	393	0.25
186	235.10	231	0.15
187	236.10	65	0.04
188	237.10	142	0.09
189	238.00	30	0.02
190	239.10	381	0.24
191	240.10	130	0.08
192	241.10	1284	0.82
193	242.10	380	0.24
194	243.10	1471	0.94
195	244.10	633	0.41
196	245.10	2927	1.88
197	246.10	1005	0.64
198	247.10	1466	0.94
199	248.10	531	0.34
200	249.10	391	0.25
201	250.10	50	0.03
202	251.10	136	0.09
203	252.10	18	0.01
204	253.10	384	0.25
205	254.10	125	0.08
206	255.10	2488	1.59
207	256.10	715	0.46
208	257.10	1363	0.87
209	258.10	585	0.37
210	259.10	5364	3.44
211	260.10	1483	0.95
212	261.10	560	0.36
213	262.10	213	0.14
214	263.10	71	0.05
215	264.10	6	0.00
216	265.10	200	0.13
217	266.20	25	0.02
218	267.10	398	0.26
219	268.10	107	0.07
220	269.10	948	0.61
221	270.10	434	0.28
222	271.10	1294	0.83
223	272.15	688	0.44
224	273.15	3650	2.34
225	274.10	2180	1.40
226	275.15	1240	0.79
227	276.15	442	0.28
228	277.20	53	0.03
229	278.20	2	0.00
230	279.10	8	0.01
231	280.10	2	0.00
232	281.00	1030	0.66
233	282.00	310	0.20
234	283.05	538	0.34
235	284.10	268	0.17
236	285.15	781	0.50
237	286.15	434	0.28
238	287.15	1436	0.92
239	288.15	580	0.37
240	289.15	410	0.26
241	290.20	75	0.05
242	293.20	10	0.01
243	294.20	2	0.00
244	295.20	258	0.17
245	296.10	54	0.03
246	297.10	367	0.24
247	298.20	138	0.09
248	299.15	570	0.37
249	300.15	288	0.18
250	301.10	571	0.37
251	302.15	292	0.19
252	303.20	110	0.07
253	304.20	3	0.00
254	307.10	5	0.00
255	309.20	126	0.08
256	310.10	15	0.01

#	m/z	Abs. Int.	Rel. Int.
257	311.15	391	0.25
258	312.20	154	0.10
259	313.20	2000	1.28
260	314.15	588	0.38
261	315.15	287	0.18
262	316.20	91	0.06
263	317.10	12	0.01
264	321.20	1	0.00
265	323.10	70	0.04
266	324.20	60	0.04
267	325.15	675	0.43
268	326.20	424	0.27
269	327.20	754	0.48
270	328.20	235	0.15
271	329.20	590	0.38
272	330.20	175	0.11
273	331.20	10	0.01
274	335.20	8	0.01
275	337.20	430	0.28
276	338.20	122	0.08
277	339.20	191	0.12
278	340.10	86	0.06
279	341.10	393	0.25
280	342.20	211	0.14
281	343.20	4009	2.57
282	344.20	3405	2.18
283	345.20	843	0.54
284	346.20	99	0.06
285	349.20	3	0.00
286	351.20	48	0.03
287	352.20	39	0.02
288	353.20	96	0.06
289	354.20	95	0.06
290	355.15	1440	0.92
291	356.10	424	0.27
292	357.10	108	0.07
293	358.10	15	0.01
294	364.20	2	0.00
295	365.20	105	0.07
296	366.20	6	0.00
297	367.10	117	0.07
298	368.20	27	0.02
299	369.25	977	0.63
300	370.20	707	0.45
301	371.20	181	0.12
302	372.20	12	0.01
303	375.20	4	0.00
304	377.20	2	0.00
305	378.20	1	0.00
306	381.20	6	0.00
307	382.20	14	0.01
308	383.20	608	0.39
309	384.20	179	0.11
310	385.20	6	0.00
311	390.20	4	0.00
312	391.20	133	0.09
313	392.20	30	0.02
314	393.20	442	0.28
315	394.20	130	0.08
316	395.20	153	0.10
317	396.20	33	0.02
318	397.20	134	0.09
319	398.20	87	0.06
320	399.20	2	0.00
321	400.20	1	0.00
322	402.20	2	0.00
323	404.10	1	0.00
324	406.20	2	0.00
325	407.20	8	0.01
326	408.20	136	0.09
327	409.20	322	0.21
328	410.20	121	0.08
329	411.20	35537	22.77
330	412.20	10851	6.95
331	413.15	1822	1.17
332	414.20	203	0.13
333	415.10	6	0.00
334	420.20	1	0.00
335	422.30	1	0.00
336	424.20	902	0.58

DEPTT. OF BOTANICAL & ENVIRONMENTAL SCIENCES, G.N.D.U. AMRITSAR

#	m/z	Abs. Int.	Rel. Int.
337	425.25	326	0.21
338	426.25	5791	3.71
339	427.20	1865	1.20
340	428.20	311	0.20
341	429.20	22	0.01
342	430.20	8	0.01
343	432.20	4	0.00
344	450.20	2	0.00
345	465.20	2	0.00
346	482.20	1	0.00
347	492.20	2	0.00
348	539.20	2	0.00
349	566.20	1	0.00
350	572.20	2	0.00
351	588.20	1	0.00
352	596.20	1	0.00
353	601.30	2	0.00
354	601.90	2	0.00
355	610.30	4	0.00
356	634.30	1	0.00
357	642.20	5	0.00
358	651.20	2	0.00
359	660.20	2	0.00
360	674.20	1	0.00
361	697.20	1	0.00

Line#:27 R.Time:35.2(Scan#:9361)
MassPeaks:348
RawMode:Averaged 35.1-35.3(9323-9389) BasePeak:95(34893)
BG Mode:None Group 1 - Event 1

#	m/z	Abs. Int.	Rel. Int.
1	50.00	606	1.74
2	51.10	1193	3.42
3	52.10	630	1.81
4	53.10	4007	11.48
5	54.15	1188	3.40
6	55.10	31855	91.29
7	56.15	3712	10.64
8	57.15	28062	80.42
9	58.10	1710	4.90
10	59.15	889	2.55
11	60.10	986	2.83
12	61.10	440	1.26
13	62.10	231	0.66
14	63.10	633	1.81
15	64.10	434	1.24
16	65.05	2534	7.26
17	66.15	953	2.73
18	67.10	18143	52.00
19	68.10	3070	8.80
20	69.10	24352	69.79
21	70.10	2814	8.06
22	71.10	14460	41.44
23	72.15	1076	3.08
24	73.10	3462	9.92
25	74.10	521	1.49
26	75.10	757	2.17
27	76.10	482	1.38
28	77.10	7182	20.58
29	78.05	2077	5.95
30	79.10	19452	55.75
31	80.10	3063	8.78
32	81.10	29117	83.45
33	82.10	4234	12.13
34	83.10	11704	33.54
35	84.10	1956	5.61
36	85.15	10975	31.45
37	86.10	918	2.63
38	87.10	647	1.85
39	88.10	163	0.47
40	89.10	551	1.58
41	90.10	232	0.66
42	91.05	22167	63.53
43	92.10	4660	13.36
44	93.10	21536	61.72
45	94.10	6219	17.82
46	95.10	34893	100.00
47	96.10	4975	14.26
48	97.10	9750	27.94
49	98.10	1554	4.45
50	99.15	2708	7.76
51	100.20	399	1.14
52	101.10	506	1.45
53	102.10	384	1.10
54	103.05	1814	5.20
55	104.15	1299	3.72
56	105.10	26611	76.26
57	106.10	6177	17.70
58	107.10	25518	73.13
59	108.10	7409	21.23
60	109.10	25146	72.07
61	110.10	3928	11.26
62	111.10	7379	21.15
63	112.20	984	2.82
64	113.10	1035	2.97
65	114.10	290	0.83
66	115.05	3447	9.88
67	116.10	1527	4.38
68	117.10	8926	25.58
69	118.10	3689	10.57
70	119.10	20368	58.37
71	120.10	9543	27.35
72	121.10	16461	47.18
73	122.15	5360	15.36
74	123.10	10571	30.30
75	124.10	3266	9.36
76	125.15	3789	10.86
77	126.15	672	1.93
78	127.10	1335	3.83
79	128.10	3100	8.88
80	129.10	5932	17.00
81	130.10	2549	7.31
82	131.10	12588	36.08
83	132.10	3915	11.22
84	133.10	17209	49.32
85	134.10	6398	18.34
86	135.10	14155	40.57
87	136.10	5170	14.82
88	137.15	4972	14.25
89	138.10	1748	5.01
90	139.15	1662	4.76
91	140.10	348	1.00
92	141.05	2080	5.96
93	142.05	1917	5.49
94	143.10	9065	25.98
95	144.10	3363	9.64
96	145.10	18251	52.31
97	146.10	4881	13.99
98	147.15	13478	38.63
99	148.10	4654	13.34
100	149.15	8862	25.40
101	150.15	2026	5.81
102	151.15	2991	8.57
103	152.10	990	2.84
104	153.10	1133	3.25
105	154.10	595	1.71
106	155.10	1639	4.70
107	156.15	1127	3.23
108	157.10	5819	16.68
109	158.10	4685	13.43
110	159.10	12081	34.62
111	160.10	7070	20.26
112	161.15	12166	34.87
113	162.15	3538	10.14
114	163.15	10625	30.45
115	164.15	2056	5.89
116	165.15	2286	6.55
117	166.20	706	2.02
118	167.15	781	2.24
119	168.10	447	1.28
120	169.10	1368	3.92
121	170.10	669	1.92
122	171.15	5224	14.97
123	172.15	1906	5.46
124	173.15	6926	19.85
125	174.15	2550	7.31
126	175.15	4996	14.32
127	176.15	1870	5.36
128	177.15	3860	11.06
129	178.10	3671	10.52
130	179.15	1821	5.22
131	180.10	482	1.38
132	181.10	534	1.53
133	182.10	304	0.87
134	183.10	1129	3.24
135	184.15	607	1.74
136	185.10	4413	12.65
137	186.10	1929	5.53
138	187.10	4887	14.01
139	188.15	1704	4.88
140	189.15	9576	27.44
141	190.15	3152	9.03
142	191.15	6241	17.89
143	192.15	1321	3.79
144	193.10	1087	3.12
145	194.10	307	0.88
146	195.10	416	1.19
147	196.10	210	0.60
148	197.10	1222	3.50
149	198.10	529	1.52
150	199.10	5425	15.55
151	200.10	1800	5.16
152	201.15	2949	8.45
153	202.10	1269	3.64
154	203.10	6815	19.53
155	204.15	3892	11.15
156	205.15	6825	19.56
157	206.15	2796	8.01
158	207.10	5868	16.82
159	208.10	1291	3.70
160	209.10	700	2.01
161	210.00	239	0.68
162	211.10	894	2.56
163	212.15	506	1.45
164	213.10	11016	31.57
165	214.10	3105	8.90
166	215.10	2852	8.17
167	216.10	946	2.71
168	217.10	1543	4.42
169	218.15	7409	21.23
170	219.15	2968	8.51
171	220.15	640	1.83
172	221.10	1141	3.27
173	222.10	346	0.99
174	223.10	358	1.03
175	224.10	78	0.22
176	225.05	597	1.71
177	226.10	311	0.89
178	227.10	2323	6.66
179	228.10	2132	6.11
180	229.10	3680	10.55
181	230.15	963	2.76
182	231.10	5604	16.06
183	232.10	1583	4.54
184	233.10	1041	2.98
185	234.20	923	2.65
186	235.15	500	1.43
187	236.20	134	0.38
188	237.10	178	0.51
189	238.10	26	0.07
190	239.10	1113	3.19
191	240.10	403	1.15
192	241.10	1646	4.72
193	242.10	587	1.68
194	243.10	654	1.87
195	244.10	396	1.13

DEPTT. OF BOTANICAL & ENVIRONMENTAL SCIENCES, G.N.D.U. AMRITSAR

#	m/z	Abs. Int.	Rel. Int.
196	245.15	928	2.66
197	246.10	918	2.63
198	247.15	918	2.63
199	248.20	642	1.84
200	249.10	379	1.09
201	250.20	74	0.21
202	251.10	206	0.59
203	252.20	35	0.10
204	253.10	600	1.72
205	254.10	407	1.17
206	255.15	5765	16.52
207	256.15	1402	4.02
208	257.10	1172	3.36
209	258.10	316	0.91
210	259.15	650	1.86
211	260.10	378	1.08
212	261.10	342	0.98
213	262.10	45	0.13
214	263.20	27	0.08
215	264.10	3	0.01
216	265.10	300	0.86
217	266.10	36	0.10
218	267.10	481	1.38
219	268.20	237	0.68
220	269.10	616	1.77
221	270.10	197	0.56
222	271.10	619	1.77
223	272.15	493	1.41
224	273.15	3310	9.49
225	274.15	1048	3.00
226	275.20	1313	3.76
227	276.15	367	1.05
228	277.20	34	0.10
229	278.20	3	0.01
230	279.10	9	0.03
231	280.00	9	0.03
232	280.95	1369	3.92
233	282.00	453	1.30
234	283.10	812	2.33
235	284.10	244	0.70
236	285.10	242	0.69
237	286.10	67	0.19
238	287.10	339	0.97
239	288.20	441	1.26
240	289.20	298	0.85
241	290.20	167	0.48
242	291.20	25	0.07
243	292.10	3	0.01
244	295.10	50	0.14
245	296.20	28	0.08
246	297.10	157	0.45

#	m/z	Abs. Int.	Rel. Int.
247	298.10	39	0.11
248	299.10	125	0.36
249	300.10	56	0.16
250	301.20	292	0.84
251	302.25	1163	3.33
252	303.20	3418	9.80
253	304.20	902	2.59
254	305.20	127	0.36
255	307.20	3	0.01
256	309.20	15	0.04
257	311.20	99	0.28
258	312.30	10	0.03
259	313.20	117	0.34
260	314.20	129	0.37
261	315.30	167	0.48
262	316.30	89	0.26
263	317.20	10	0.03
264	320.20	6	0.02
265	321.30	6	0.02
266	322.00	3	0.01
267	323.20	6	0.02
268	325.20	71	0.20
269	326.20	3	0.01
270	327.20	127	0.36
271	328.25	406	1.16
272	329.20	4768	13.66
273	330.15	1295	3.71
274	331.20	182	0.52
275	337.30	3	0.01
276	339.20	191	0.55
277	340.20	51	0.15
278	341.20	362	1.04
279	342.20	276	0.79
280	343.20	120	0.34
281	344.20	23	0.07
282	345.20	3	0.01
283	350.20	3	0.01
284	352.20	3	0.01
285	353.20	147	0.42
286	354.20	723	2.07
287	355.20	735	2.11
288	356.20	245	0.70
289	357.20	274	0.79
290	358.20	46	0.13
291	359.20	10	0.03
292	365.20	15	0.04
293	366.20	3	0.01
294	367.20	217	0.62
295	368.20	129	0.37
296	369.20	40	0.11
297	370.20	64	0.18

#	m/z	Abs. Int.	Rel. Int.
298	371.20	166	0.48
299	372.20	27	0.08
300	373.20	3	0.01
301	375.20	3	0.01
302	377.20	3	0.01
303	379.20	6	0.02
304	381.25	2552	7.31
305	382.20	772	2.21
306	383.20	136	0.39
307	384.20	3	0.01
308	385.30	62	0.18
309	386.20	3	0.01
310	390.00	3	0.01
311	393.20	69	0.20
312	394.20	35	0.10
313	395.30	60	0.17
314	396.25	2670	7.65
315	397.25	851	2.44
316	398.20	147	0.42
317	399.25	1807	5.18
318	400.20	588	1.69
319	401.20	62	0.18
320	407.30	3	0.01
321	408.20	401	1.15
322	409.20	166	0.48
323	410.00	77	0.22
324	411.20	323	0.93
325	412.20	212	0.61
326	413.20	79	0.23
327	414.25	3067	8.79
328	415.20	983	2.82
329	416.20	181	0.52
330	417.20	3	0.01
331	423.90	35	0.10
332	425.20	3	0.01
333	426.25	453	1.30
334	427.20	141	0.40
335	428.00	3	0.01
336	429.10	6	0.02
337	430.20	6	0.02
338	445.20	3	0.01
339	474.00	3	0.01
340	484.20	3	0.01
341	542.30	3	0.01
342	556.10	3	0.01
343	573.10	3	0.01
344	582.20	6	0.02
345	587.20	3	0.01
346	615.10	6	0.02
347	666.20	3	0.01
348	688.20	3	0.01

Line#:28 R.Time:35.5(Scan#:9442)
MassPeaks:347
RawMode:Averaged 35.3-35.5(9389-9465) BasePeak:218(210860)
BG Mode:None Group 1 - Event 1

#	m/z	Abs. Int.	Rel. Int.
1	50.10	698	0.33
2	51.10	1530	0.73
3	52.10	849	0.40
4	53.10	9246	4.38
5	54.15	1989	0.94
6	55.10	68858	32.66
7	56.10	6271	2.97
8	57.10	34094	16.17
9	58.10	2696	1.28
10	59.10	2548	1.21
11	60.05	1022	0.48
12	61.10	472	0.22
13	62.10	281	0.13
14	63.05	695	0.33
15	64.10	463	0.22
16	65.05	4694	2.23
17	66.15	1601	0.76
18	67.10	36831	17.47
19	68.10	5921	2.81
20	69.10	72227	34.25
21	70.10	5681	2.69
22	71.05	15249	7.23
23	72.10	1748	0.83

#	m/z	Abs. Int.	Rel. Int.
24	73.10	3969	1.88
25	74.10	640	0.30
26	75.10	801	0.38
27	76.15	517	0.25
28	77.05	13044	6.19
29	78.10	3387	1.61
30	79.10	41740	19.80
31	80.10	11370	5.39
32	81.10	63420	30.08
33	82.10	7582	3.60
34	83.10	17594	8.34
35	84.10	2466	1.17
36	85.10	5948	2.82
37	86.05	974	0.46
38	87.05	816	0.39
39	88.10	309	0.15
40	89.10	614	0.29
41	90.15	396	0.19
42	91.05	39873	18.91
43	92.05	7603	3.61
44	93.10	52666	24.98
45	94.10	39230	18.60
46	95.10	80641	38.24

#	m/z	Abs. Int.	Rel. Int.
47	96.10	9819	4.66
48	97.10	12835	6.09
49	98.10	2034	0.96
50	99.05	1900	0.90
51	100.15	607	0.29
52	101.10	754	0.36
53	102.05	521	0.25
54	103.05	3080	1.46
55	104.15	1801	0.85
56	105.10	52257	24.78
57	106.10	12087	5.73
58	107.10	56132	26.62
59	108.10	22816	10.82
60	109.10	52424	24.86
61	110.10	6128	2.91
62	111.10	12592	5.97
63	112.10	1532	0.73
64	113.15	1165	0.55
65	114.05	636	0.30
66	115.05	5100	2.42
67	116.05	2276	1.08
68	117.05	11661	5.53
69	118.15	3800	1.80

DEPTT. OF BOTANICAL & ENVIRONMENTAL SCIENCES, G.N.D.U. AMRITSAR

#	m/z	Abs. Int.	Rel. Int.
70	119.10	54517	25.85
71	120.10	19776	9.38
72	121.10	43741	20.74
73	122.10	24389	11.57
74	123.10	26835	12.73
75	124.15	4034	1.91
76	125.15	3787	1.80
77	126.10	811	0.38
78	127.10	1938	0.92
79	128.10	4282	2.03
80	129.05	6867	3.26
81	130.10	3078	1.46
82	131.10	16068	7.62
83	132.10	4873	2.31
84	133.10	39968	18.95
85	134.10	22146	10.50
86	135.10	44213	20.97
87	136.10	36912	17.51
88	137.15	24068	11.41
89	138.15	3602	1.71
90	139.10	3997	1.90
91	140.10	789	0.37
92	141.10	2939	1.39
93	142.05	2941	1.39
94	143.05	6545	3.10
95	144.10	2791	1.32
96	145.10	15271	7.24
97	146.10	5298	2.51
98	147.10	35134	16.66
99	148.15	19393	9.20
100	149.15	21845	10.36
101	150.15	6459	3.06
102	151.15	3485	1.65
103	152.10	1145	0.54
104	153.10	1635	0.78
105	154.15	1062	0.50
106	155.10	2207	1.05
107	156.10	1746	0.83
108	157.10	4797	2.27
109	158.10	2078	0.99
110	159.10	10800	5.12
111	160.10	3732	1.77
112	161.15	24774	11.75
113	162.10	15607	7.40
114	163.15	13667	6.48
115	164.10	2554	1.21
116	165.10	2498	1.18
117	166.10	901	0.43
118	167.05	938	0.44
119	168.10	567	0.27
120	169.10	1553	0.74
121	170.10	808	0.38
122	171.10	3335	1.58
123	172.15	1198	0.57
124	173.10	7100	3.37
125	174.15	2393	1.13
126	175.10	30340	14.39
127	176.15	10164	4.82
128	177.15	8114	3.85
129	178.10	1954	0.93
130	179.10	2519	1.19
131	180.10	624	0.30
132	181.10	639	0.30
133	182.00	415	0.20
134	183.10	1112	0.53
135	184.05	453	0.21
136	185.05	2364	1.12
137	186.10	991	0.47
138	187.10	6944	3.29
139	188.15	2999	1.42
140	189.10	64370	30.53
141	190.10	23060	10.94
142	191.10	11672	5.54
143	192.15	2220	1.05
144	193.10	1594	0.76
145	194.10	516	0.24
146	195.00	554	0.26
147	196.10	255	0.12
148	197.05	915	0.43
149	198.10	398	0.19

#	m/z	Abs. Int.	Rel. Int.
150	199.05	2024	0.96
151	200.10	768	0.36
152	201.10	5294	2.51
153	202.15	2972	1.41
154	203.10	162968	77.29
155	204.10	31052	14.73
156	205.10	11408	5.41
157	206.10	4570	2.17
158	207.10	20113	9.54
159	208.10	5301	2.51
160	209.10	1225	0.58
161	210.00	307	0.15
162	211.00	860	0.41
163	212.10	382	0.18
164	213.05	2176	1.03
165	214.05	1030	0.49
166	215.10	5391	2.56
167	216.10	2040	0.97
168	217.15	3719	1.76
169	218.10	210860	100.00
170	219.10	39135	18.56
171	220.10	4279	2.03
172	221.05	1295	0.61
173	222.10	381	0.18
174	223.10	425	0.20
175	224.10	161	0.08
176	225.05	585	0.28
177	226.00	247	0.12
178	227.10	1372	0.65
179	228.10	676	0.32
180	229.10	4420	2.10
181	230.10	1468	0.70
182	231.10	3148	1.49
183	232.10	1422	0.67
184	233.10	1271	0.60
185	234.10	993	0.47
186	235.10	317	0.15
187	236.10	59	0.03
188	237.10	209	0.10
189	238.10	45	0.02
190	239.10	672	0.32
191	240.10	301	0.14
192	241.10	1307	0.62
193	242.10	500	0.24
194	243.10	3500	1.66
195	244.10	1250	0.59
196	245.10	1247	0.59
197	246.10	493	0.23
198	247.10	317	0.15
199	248.10	380	0.18
200	249.10	360	0.17
201	250.10	52	0.02
202	251.10	269	0.13
203	252.10	72	0.03
204	253.10	518	0.25
205	254.10	266	0.13
206	255.10	2117	1.00
207	256.10	719	0.34
208	257.10	7114	3.37
209	258.10	1960	0.93
210	259.10	1273	0.60
211	260.10	283	0.13
212	261.10	116	0.06
213	262.10	14	0.01
214	263.10	40	0.02
215	264.20	2	0.00
216	265.10	362	0.17
217	266.10	98	0.05
218	266.95	569	0.27
219	268.10	233	0.11
220	269.05	1308	0.62
221	270.15	744	0.35
222	271.15	1708	0.81
223	272.15	1789	0.85
224	273.10	850	0.40
225	274.10	279	0.13
226	275.10	182	0.09
227	276.10	3	0.00
228	277.20	8	0.00
229	278.10	6	0.00

#	m/z	Abs. Int.	Rel. Int.
230	279.10	45	0.02
231	280.95	1873	0.89
232	282.00	578	0.27
233	283.10	731	0.35
234	284.15	371	0.18
235	285.10	539	0.26
236	286.15	497	0.24
237	287.10	255	0.12
238	288.20	150	0.07
239	289.20	63	0.03
240	290.20	12	0.01
241	292.20	5	0.00
242	293.00	2	0.00
243	294.10	5	0.00
244	295.10	171	0.08
245	296.10	111	0.05
246	297.10	356	0.17
247	298.20	114	0.05
248	299.20	244	0.12
249	300.10	113	0.05
250	301.20	75	0.04
251	302.10	32	0.02
252	303.20	115	0.05
253	307.10	8	0.00
254	308.00	6	0.00
255	309.20	58	0.03
256	310.20	15	0.01
257	311.20	247	0.12
258	312.10	46	0.02
259	313.20	117	0.06
260	314.20	236	0.11
261	315.20	106	0.05
262	316.20	16	0.01
263	319.00	3	0.00
264	321.00	6	0.00
265	323.20	97	0.05
266	324.20	41	0.02
267	325.20	181	0.09
268	326.20	46	0.02
269	327.00	135	0.06
270	328.20	27	0.01
271	329.00	217	0.10
272	330.20	16	0.01
273	331.20	8	0.00
274	335.20	8	0.00
275	337.10	280	0.13
276	338.20	60	0.03
277	339.20	142	0.07
278	340.20	56	0.03
279	341.20	254	0.12
280	342.20	47	0.02
281	343.00	11	0.01
282	349.00	2	0.00
283	350.20	2	0.00
284	351.20	59	0.03
285	352.10	12	0.01
286	353.20	66	0.03
287	354.20	28	0.01
288	355.10	398	0.19
289	356.10	121	0.06
290	357.20	26	0.01
291	358.20	2	0.00
292	364.10	5	0.00
293	365.20	170	0.08
294	366.10	20	0.01
295	367.20	21	0.01
296	368.20	44	0.02
297	369.10	68	0.03
298	370.10	5	0.00
299	373.00	2	0.00
300	374.10	2	0.00
301	377.20	3	0.00
302	379.20	8	0.00
303	380.10	15	0.01
304	381.10	97	0.05
305	382.20	11	0.01
306	383.20	61	0.03
307	384.00	3	0.00
308	391.20	12	0.01
309	393.20	1012	0.48

#	m/z	Abs. Int.	Rel. Int.
310	394.20	328	0.16
311	395.20	73	0.03
312	396.20	151	0.07
313	397.20	67	0.03
314	398.20	10	0.00
315	399.20	23	0.01
316	400.00	9	0.00
317	401.20	65	0.03
318	404.20	8	0.00
319	406.20	2	0.00
320	407.20	25	0.01
321	408.20	450	0.21
322	409.20	242	0.11

#	m/z	Abs. Int.	Rel. Int.
323	410.20	301	0.14
324	411.20	1923	0.91
325	412.20	602	0.29
326	413.20	84	0.04
327	414.10	69	0.03
328	415.20	9	0.00
329	416.20	45	0.02
330	417.00	2	0.00
331	418.00	5	0.00
332	422.00	10	0.00
333	424.20	106	0.05
334	425.20	73	0.03
335	426.20	2794	1.33

#	m/z	Abs. Int.	Rel. Int.
336	427.20	946	0.45
337	428.20	151	0.07
338	429.20	23	0.01
339	430.20	18	0.01
340	443.00	2	0.00
341	498.20	2	0.00
342	501.00	2	0.00
343	520.00	2	0.00
344	569.00	2	0.00
345	572.00	2	0.00
346	629.20	2	0.00
347	635.20	2	0.00

Line#:29 R.Time:36.3(Scan#:9700)
MassPeaks:415
RawMode:Averaged 36.0-36.4(9588-9727) BasePeak:218(244782)
BG Mode:None Group 1 - Event 1

#	m/z	Abs. Int.	Rel. Int.
1	50.05	882	0.36
2	51.10	1991	0.81
3	52.15	1154	0.47
4	53.10	18500	7.56
5	54.15	5081	2.08
6	55.10	132523	54.14
7	56.10	15387	6.29
8	57.15	67093	27.41
9	58.10	6582	2.69
10	59.10	8859	3.62
11	60.10	1383	0.56
12	61.10	695	0.28
13	62.10	339	0.14
14	63.05	863	0.35
15	64.15	557	0.23
16	65.05	8234	3.36
17	66.15	3594	1.47
18	67.05	86117	35.18
19	68.10	31392	12.82
20	69.10	123472	50.44
21	70.10	14173	5.79
22	71.05	42148	17.22
23	72.10	4194	1.71
24	73.05	6141	2.51
25	74.10	948	0.39
26	75.05	1089	0.44
27	76.15	680	0.28
28	77.05	23534	9.61
29	78.10	5406	2.21
30	79.05	73615	30.07
31	80.10	19111	7.81
32	81.10	131706	53.81
33	82.10	28899	11.81
34	83.10	49983	20.42
35	84.10	8904	3.64
36	85.10	18218	7.44
37	86.05	2400	0.98
38	87.10	1457	0.60
39	88.05	622	0.25
40	89.00	822	0.34
41	90.15	881	0.36
42	91.05	69940	28.57
43	92.10	14383	5.88
44	93.05	118781	48.53
45	94.10	46985	19.19
46	95.10	160374	65.52
47	96.10	28511	11.65
48	97.10	39389	16.09
49	98.10	6237	2.55
50	99.15	6249	2.55
51	100.10	1694	0.69
52	101.10	1267	0.52
53	102.05	875	0.36
54	103.05	4737	1.94
55	104.15	2758	1.13
56	105.10	89795	36.68
57	106.10	28833	11.78
58	107.10	126039	51.49
59	108.10	63985	26.14
60	109.10	120401	49.19
61	110.10	21539	8.80

#	m/z	Abs. Int.	Rel. Int.
62	111.10	29767	12.16
63	112.15	4557	1.86
64	113.15	4032	1.65
65	114.15	1674	0.68
66	115.05	6959	2.84
67	116.05	3260	1.33
68	117.05	17049	6.96
69	118.10	5942	2.43
70	119.05	111463	45.54
71	120.10	43919	17.94
72	121.10	108469	44.31
73	122.10	105161	42.96
74	123.10	90902	37.14
75	124.10	13211	5.40
76	125.10	13820	5.65
77	126.15	2823	1.15
78	127.10	4672	1.91
79	128.10	5824	2.38
80	129.10	9433	3.85
81	130.10	4527	1.85
82	131.10	26893	10.99
83	132.10	11455	4.68
84	133.10	93936	38.38
85	134.10	61587	25.16
86	135.10	124132	50.71
87	136.10	84704	34.60
88	137.10	36578	14.94
89	138.10	6894	2.82
90	139.10	11967	4.89
91	140.10	2628	1.07
92	141.10	4909	2.01
93	142.05	4382	1.79
94	143.10	9223	3.77
95	144.10	4302	1.76
96	145.10	29707	12.14
97	146.10	12426	5.08
98	147.10	88014	35.96
99	148.10	65617	26.81
100	149.15	64710	26.44
101	150.15	18137	7.41
102	151.10	8985	3.67
103	152.10	6344	2.59
104	153.10	5719	2.34
105	154.10	1974	0.81
106	155.05	3538	1.45
107	156.05	2521	1.03
108	157.10	7595	3.10
109	158.10	3376	1.38
110	159.10	23025	9.41
111	160.15	9412	3.85
112	161.10	71971	29.40
113	162.15	36667	14.98
114	163.15	33509	13.69
115	164.15	6161	2.52
116	165.10	4908	2.01
117	166.10	2328	0.95
118	167.20	2249	0.92
119	168.10	1046	0.43
120	169.10	2431	0.99
121	170.10	1155	0.47
122	171.10	5975	2.44

#	m/z	Abs. Int.	Rel. Int.
123	172.15	2451	1.00
124	173.10	17902	7.31
125	174.10	5902	2.41
126	175.10	47272	19.31
127	176.15	16172	6.61
128	177.15	16825	6.87
129	178.10	4785	1.95
130	179.10	6847	2.80
131	180.10	1563	0.64
132	181.20	1482	0.61
133	182.20	695	0.28
134	183.10	1812	0.74
135	184.10	761	0.31
136	185.10	4270	1.74
137	186.15	2449	1.00
138	187.10	22699	9.27
139	188.10	8587	3.51
140	189.10	120472	49.22
141	190.10	45639	18.64
142	191.10	29668	12.12
143	192.10	6873	2.81
144	193.10	4434	1.81
145	194.10	1307	0.53
146	195.00	1222	0.50
147	196.15	516	0.21
148	197.05	1403	0.57
149	198.05	594	0.24
150	199.05	3494	1.43
151	200.10	1419	0.58
152	201.05	15825	6.46
153	202.10	6865	2.80
154	203.10	93929	38.37
155	204.10	33670	13.76
156	205.10	20552	8.40
157	206.10	14082	5.75
158	207.05	66492	27.16
159	208.10	18027	7.36
160	209.05	3331	1.36
161	210.00	675	0.28
162	211.05	1215	0.50
163	212.05	493	0.20
164	213.05	3496	1.43
165	214.15	1381	0.56
166	215.05	9041	3.69
167	216.05	7523	3.07
168	217.10	11720	4.79
169	218.10	244782	100.00
170	219.10	50543	20.65
171	220.10	10931	4.47
172	221.10	4277	1.75
173	222.05	1433	0.59
174	223.05	870	0.36
175	224.10	322	0.13
176	225.05	917	0.37
177	226.05	400	0.16
178	227.05	2667	1.09
179	228.10	1259	0.51
180	229.05	11463	4.68
181	230.10	3855	1.57
182	231.10	7670	3.13
183	232.10	3375	1.38

DEPTT. OF BOTANICAL & ENVIRONMENTAL SCIENCES, G.N.D.U. AMRITSAR

#	m/z	Abs. Int.	Rel. Int.
184	233.05	2187	0.89
185	234.05	7139	2.92
186	235.05	1638	0.67
187	236.05	422	0.17
188	237.10	464	0.19
189	238.10	183	0.07
190	239.05	925	0.38
191	240.05	421	0.17
192	241.05	2196	0.90
193	242.05	1084	0.44
194	243.10	6338	2.59
195	244.05	2155	0.88
196	245.10	3529	1.44
197	246.10	1870	0.76
198	247.05	4184	1.71
199	248.05	2156	0.88
200	249.05	832	0.34
201	250.20	302	0.12
202	251.10	457	0.19
203	252.10	189	0.08
204	253.10	804	0.33
205	254.10	354	0.14
206	255.10	3637	1.49
207	256.10	1368	0.56
208	257.10	14759	6.03
209	258.05	4322	1.77
210	259.15	8603	3.51
211	260.15	2007	0.82
212	261.10	570	0.23
213	262.10	121	0.05
214	263.00	87	0.04
215	264.10	155	0.06
216	265.00	650	0.27
217	266.00	306	0.13
218	267.00	1106	0.45
219	268.00	367	0.15
220	269.10	2166	0.88
221	270.10	1627	0.66
222	271.10	4030	1.65
223	272.10	7175	2.93
224	273.10	3490	1.43
225	274.20	4086	1.67
226	275.20	1284	0.52
227	276.15	395	0.16
228	277.10	104	0.04
229	278.20	92	0.04
230	279.20	147	0.06
231	280.00	69	0.03
232	280.95	3086	1.26
233	281.95	986	0.40
234	283.05	1751	0.72
235	284.10	1069	0.44
236	285.15	1560	0.64
237	286.10	3734	1.53
238	287.15	1566	0.64
239	288.10	856	0.35
240	289.10	313	0.13
241	290.00	135	0.06
242	291.10	45	0.02
243	292.00	31	0.01
244	293.10	96	0.04
245	294.10	23	0.01
246	295.10	631	0.26
247	296.10	371	0.15
248	297.15	3298	1.35
249	298.15	1444	0.59
250	299.15	2101	0.86
251	300.15	1803	0.74
252	301.10	1112	0.45
253	302.10	622	0.25
254	303.10	228	0.09
255	304.10	28	0.01
256	305.10	12	0.00
257	306.00	27	0.01
258	307.10	41	0.02
259	308.10	19	0.01
260	309.10	359	0.15
261	310.10	119	0.05

#	m/z	Abs. Int.	Rel. Int.
262	311.20	523	0.21
263	312.15	276	0.11
264	313.15	2228	0.91
265	314.10	1254	0.51
266	315.15	4881	1.99
267	316.15	2762	1.13
268	317.15	1274	0.52
269	318.15	333	0.14
270	319.10	55	0.02
271	320.20	9	0.00
272	321.20	11	0.00
273	322.20	7	0.00
274	323.15	513	0.21
275	324.15	239	0.10
276	325.15	806	0.33
277	326.10	453	0.19
278	327.15	464	0.19
279	328.10	195	0.08
280	329.15	314	0.13
281	330.10	145	0.06
282	331.10	58	0.02
283	332.10	7	0.00
284	332.90	3	0.00
285	334.10	20	0.01
286	335.20	1	0.00
287	336.10	4	0.00
288	337.15	621	0.25
289	338.10	294	0.12
290	339.15	650	0.27
291	340.10	387	0.16
292	341.10	816	0.33
293	342.15	758	0.31
294	343.15	816	0.33
295	344.15	808	0.33
296	345.15	228	0.09
297	346.10	34	0.01
298	347.30	1	0.00
299	348.20	9	0.00
300	349.10	13	0.01
301	350.20	7	0.00
302	351.15	298	0.12
303	352.20	132	0.05
304	353.10	239	0.10
305	354.10	143	0.06
306	355.05	770	0.31
307	356.10	425	0.17
308	357.10	774	0.32
309	358.10	345	0.14
310	359.10	66	0.03
311	360.10	6	0.00
312	362.10	3	0.00
313	363.10	32	0.01
314	364.00	18	0.01
315	365.20	1166	0.48
316	366.10	405	0.17
317	367.20	157	0.06
318	368.20	214	0.09
319	369.15	530	0.22
320	370.15	1239	0.51
321	371.20	407	0.17
322	372.20	66	0.03
323	373.10	3	0.00
324	375.10	1	0.00
325	377.10	16	0.01
326	378.20	3	0.00
327	379.10	96	0.04
328	380.10	30	0.01
329	381.20	102	0.04
330	382.20	36	0.01
331	383.15	1489	0.61
332	384.10	483	0.20
333	385.10	79	0.03
334	386.10	4	0.00
335	387.10	4	0.00
336	389.10	6	0.00
337	390.20	31	0.01
338	391.20	113	0.05
339	392.20	31	0.01

#	m/z	Abs. Int.	Rel. Int.
340	393.15	3086	1.26
341	394.15	973	0.40
342	395.20	247	0.10
343	396.20	188	0.08
344	397.10	266	0.11
345	398.10	106	0.04
346	399.10	45	0.02
347	400.20	5	0.00
348	401.00	7	0.00
349	402.10	4	0.00
350	403.10	3	0.00
351	404.10	1	0.00
352	406.10	51	0.02
353	407.20	116	0.05
354	408.20	1219	0.50
355	409.20	644	0.26
356	410.25	233	0.10
357	411.15	6931	2.83
358	412.15	2203	0.90
359	413.10	408	0.17
360	414.10	113	0.05
361	415.20	31	0.01
362	416.20	12	0.00
363	417.20	1	0.00
364	418.20	1	0.00
365	422.10	33	0.01
366	423.20	9	0.00
367	424.25	282	0.12
368	425.20	201	0.08
369	426.15	11796	4.82
370	427.15	3811	1.56
371	428.20	691	0.28
372	429.10	186	0.08
373	430.10	96	0.04
374	431.20	23	0.01
375	432.20	39	0.02
376	433.20	7	0.00
377	435.20	33	0.01
378	436.10	3	0.00
379	437.20	1	0.00
380	440.10	27	0.01
381	441.20	8	0.00
382	450.20	64	0.03
383	451.10	22	0.01
384	452.10	3	0.00
385	456.30	2	0.00
386	464.00	1	0.00
387	466.20	129	0.05
388	467.20	33	0.01
389	468.20	1	0.00
390	488.20	3	0.00
391	494.20	1	0.00
392	503.30	4	0.00
393	504.20	1	0.00
394	505.20	3	0.00
395	507.20	1	0.00
396	525.00	1	0.00
397	531.20	1	0.00
398	533.00	1	0.00
399	534.20	1	0.00
400	539.20	1	0.00
401	545.30	1	0.00
402	569.20	2	0.00
403	572.20	1	0.00
404	581.00	1	0.00
405	591.20	1	0.00
406	597.20	1	0.00
407	614.00	1	0.00
408	622.00	1	0.00
409	630.30	1	0.00
410	631.00	1	0.00
411	639.00	1	0.00
412	643.20	3	0.00
413	676.00	1	0.00
414	683.10	1	0.00
415	687.20	1	0.00

Line#:30 R.Time:37.2(Scan#:9965)

DEPTT. OF BOTANICAL & ENVIRONMENTAL SCIENCES, G.N.D.U. AMRITSAR

MassPeaks:343
RawMode:Averaged 37.1-37.3(9938-9994) BasePeak:69(43750)
BG Mode:None Group 1 - Event 1

#	m/z	Abs. Int.	Rel. Int.
1	50.00	662	1.51
2	51.10	1177	2.69
3	52.10	700	1.60
4	53.10	4918	11.24
5	54.15	1827	4.18
6	55.10	34318	78.44
7	56.10	4309	9.85
8	57.10	17835	40.77
9	58.05	1326	3.03
10	59.10	1319	3.01
11	60.05	1418	3.24
12	61.05	829	1.89
13	62.10	292	0.67
14	63.10	601	1.37
15	64.10	414	0.95
16	65.10	2716	6.21
17	66.10	1152	2.63
18	67.10	26448	60.45
19	68.10	6608	15.10
20	69.10	43750	100.00
21	70.10	4518	10.33
22	71.10	7565	17.29
23	72.10	988	2.26
24	73.10	9052	20.69
25	74.05	1217	2.78
26	75.05	1726	3.95
27	76.10	530	1.21
28	77.05	6935	15.85
29	78.10	1705	3.90
30	79.10	17176	39.26
31	80.10	3598	8.22
32	81.10	34493	78.84
33	82.10	10929	24.98
34	83.10	17616	40.27
35	84.10	2607	5.96
36	85.10	4201	9.60
37	86.10	799	1.83
38	87.05	1186	2.71
39	88.00	586	1.34
40	89.05	907	2.07
41	90.10	212	0.48
42	91.05	15489	35.40
43	92.10	2937	6.71
44	93.10	21483	49.10
45	94.10	7986	18.25
46	95.10	42347	96.79
47	96.15	18671	42.68
48	97.10	11491	26.27
49	98.10	2025	4.63
50	99.10	1861	4.25
51	100.10	426	0.97
52	101.10	633	1.45
53	102.05	593	1.36
54	103.05	1916	4.38
55	104.15	834	1.91
56	105.10	16519	37.76
57	106.10	3970	9.07
58	107.10	22614	51.69
59	108.10	10091	23.07
60	109.10	31576	72.17
61	110.10	7394	16.90
62	111.10	9444	21.59
63	112.10	1331	3.04
64	113.10	1319	3.01
65	114.15	403	0.92
66	115.05	2612	5.97
67	116.05	1163	2.66
68	117.10	4179	9.55
69	118.10	1465	3.35
70	119.10	17495	39.99
71	120.10	6078	13.89
72	121.10	20768	47.47
73	122.10	7835	17.91
74	123.10	23562	53.86
75	124.15	7505	17.15
76	125.15	13326	30.46
77	126.15	1854	4.24

#	m/z	Abs. Int.	Rel. Int.
78	127.15	1688	3.86
79	128.10	1664	3.80
80	129.10	2979	6.81
81	130.10	1165	2.66
82	131.10	5505	12.58
83	132.10	2251	5.15
84	133.10	15323	35.02
85	134.10	6865	15.69
86	135.15	15782	36.07
87	136.15	8559	19.56
88	137.15	11019	25.19
89	138.15	3296	7.53
90	139.15	1961	4.48
91	140.15	573	1.31
92	141.05	1320	3.02
93	142.10	1098	2.51
94	143.10	2507	5.73
95	144.10	1128	2.58
96	145.10	6441	14.72
97	146.10	2828	6.46
98	147.10	12163	27.80
99	148.15	5611	12.83
100	149.15	10321	23.59
101	150.15	4600	10.51
102	151.15	4003	9.15
103	152.15	2112	4.83
104	153.10	1769	4.04
105	154.15	617	1.41
106	155.15	1117	2.55
107	156.10	728	1.66
108	157.10	2225	5.09
109	158.10	1067	2.44
110	159.10	5410	12.37
111	160.10	3139	7.17
112	161.10	10717	24.50
113	162.15	4120	9.42
114	163.15	11008	25.16
115	164.10	5842	13.35
116	165.10	10333	23.62
117	166.10	1674	3.83
118	167.10	876	2.00
119	168.10	440	1.01
120	169.05	879	2.01
121	170.10	411	0.94
122	171.15	1909	4.36
123	172.10	892	2.04
124	173.15	4116	9.41
125	174.15	1551	3.55
126	175.15	7429	16.98
127	176.10	2921	6.68
128	177.10	4824	11.03
129	178.10	1846	4.22
130	179.15	4921	11.25
131	180.10	1128	2.58
132	181.10	553	1.26
133	182.20	297	0.68
134	183.10	661	1.51
135	184.10	279	0.64
136	185.05	1878	4.29
137	186.05	837	1.91
138	187.10	3953	9.04
139	188.15	1396	3.19
140	189.10	11304	25.84
141	190.15	4113	9.40
142	191.10	8737	19.97
143	192.10	2087	4.77
144	193.10	2588	5.92
145	194.10	724	1.65
146	195.10	586	1.34
147	196.00	181	0.41
148	197.10	567	1.30
149	198.10	245	0.56
150	199.10	1494	3.41
151	200.15	648	1.48
152	201.10	2905	6.64
153	202.15	1558	3.56
154	203.10	18082	41.33

#	m/z	Abs. Int.	Rel. Int.
155	204.10	6021	13.76
156	205.10	8018	18.33
157	206.05	2650	6.06
158	207.00	12852	29.38
159	208.05	2893	6.61
160	208.90	1785	4.08
161	210.00	409	0.93
162	211.00	609	1.39
163	212.00	226	0.52
164	213.05	1361	3.11
165	214.10	662	1.51
166	215.10	3202	7.32
167	216.10	1332	3.04
168	217.10	3325	7.60
169	218.10	21835	49.91
170	219.10	5222	11.94
171	220.10	1937	4.43
172	221.05	1961	4.48
173	222.00	602	1.38
174	223.05	679	1.55
175	224.10	234	0.53
176	225.10	542	1.24
177	226.10	190	0.43
178	227.10	941	2.15
179	228.10	491	1.12
180	229.10	3480	7.95
181	230.15	1141	2.61
182	231.10	4003	9.15
183	232.10	2520	5.76
184	233.10	1641	3.75
185	234.15	1139	2.60
186	235.10	589	1.35
187	236.10	139	0.32
188	237.10	249	0.57
189	238.10	29	0.07
190	239.10	425	0.97
191	240.10	192	0.44
192	241.10	1539	3.52
193	242.05	447	1.02
194	243.10	1174	2.68
195	244.10	497	1.14
196	245.10	1682	3.84
197	246.10	3007	6.87
198	247.10	2547	5.82
199	248.10	1766	4.04
200	249.00	1248	2.85
201	250.00	311	0.71
202	251.00	498	1.14
203	252.00	135	0.31
204	253.00	489	1.12
205	254.00	164	0.37
206	255.10	1806	4.13
207	256.10	533	1.22
208	257.10	2009	4.59
209	258.15	542	1.24
210	259.10	2281	5.21
211	260.05	564	1.29
212	261.10	561	1.28
213	262.10	186	0.43
214	263.10	79	0.18
215	264.10	57	0.13
216	264.95	1002	2.29
217	265.90	365	0.83
218	266.90	1212	2.77
219	267.90	358	0.82
220	269.10	908	2.08
221	270.15	547	1.25
222	271.15	832	1.90
223	272.15	462	1.06
224	273.15	3034	6.93
225	274.15	1616	3.69
226	275.15	688	1.57
227	276.20	174	0.40
228	278.10	4	0.01
229	279.10	78	0.18
230	280.95	4397	10.05
231	281.90	1289	2.95

#	m/z	Abs. Int.	Rel. Int.
232	283.05	1052	2.40
233	284.00	338	0.77
234	285.20	522	1.19
235	286.15	254	0.58
236	287.15	813	1.86
237	288.15	319	0.73
238	289.10	157	0.36
239	290.10	15	0.03
240	293.00	42	0.10
241	295.10	246	0.56
242	296.00	54	0.12
243	297.10	316	0.72
244	298.10	75	0.17
245	299.20	154	0.35
246	300.10	141	0.32
247	301.20	167	0.38
248	302.20	928	2.12
249	303.15	425	0.97
250	304.20	87	0.20
251	307.00	7	0.02
252	307.90	3	0.01
253	309.20	49	0.11
254	310.20	3	0.01
255	311.20	92	0.21
256	312.20	29	0.07
257	313.10	114	0.26
258	314.20	24	0.05
259	315.20	87	0.20
260	317.00	7	0.02
261	323.20	83	0.19
262	324.20	15	0.03
263	325.20	271	0.62
264	326.20	48	0.11
265	327.20	329	0.75
266	328.00	66	0.15
267	329.10	68	0.16
268	330.20	4	0.01
269	335.20	4	0.01

#	m/z	Abs. Int.	Rel. Int.
270	337.20	22	0.05
271	339.20	109	0.25
272	340.10	35	0.08
273	341.10	865	1.98
274	342.10	272	0.62
275	343.10	186	0.43
276	344.10	130	0.30
277	345.10	4	0.01
278	353.20	69	0.16
279	354.10	42	0.10
280	355.00	799	1.83
281	356.00	315	0.72
282	357.00	199	0.45
283	358.20	11	0.03
284	359.20	15	0.03
285	365.10	52	0.12
286	366.90	18	0.04
287	367.90	15	0.03
288	369.00	53	0.12
289	370.10	27	0.06
290	371.00	4	0.01
291	379.10	51	0.12
292	380.10	3	0.01
293	382.00	3	0.01
294	383.20	46	0.11
295	383.90	13	0.03
296	391.20	15	0.03
297	392.10	3	0.01
298	393.15	341	0.78
299	394.20	109	0.25
300	395.10	17	0.04
301	395.90	15	0.03
302	397.20	26	0.06
303	399.10	3	0.01
304	401.00	3	0.01
305	403.00	3	0.01
306	406.00	3	0.01
307	407.20	96	0.22

#	m/z	Abs. Int.	Rel. Int.
308	408.20	128	0.29
309	409.20	103	0.24
310	410.20	19	0.04
311	411.15	861	1.97
312	412.20	321	0.73
313	413.20	47	0.11
314	415.20	11	0.03
315	416.00	11	0.03
316	417.20	7	0.02
317	421.00	3	0.01
318	422.20	7	0.02
319	423.20	3	0.01
320	424.20	3	0.01
321	425.20	59	0.13
322	426.20	557	1.27
323	427.20	351	0.80
324	428.00	98	0.22
325	429.00	240	0.55
326	430.20	1022	2.34
327	431.25	340	0.78
328	432.30	52	0.12
329	441.20	3	0.01
330	453.20	65	0.15
331	454.20	18	0.04
332	468.20	119	0.27
333	469.20	32	0.07
334	473.20	3	0.01
335	478.90	3	0.01
336	485.20	3	0.01
337	485.90	3	0.01
338	487.30	3	0.01
339	490.30	3	0.01
340	557.20	3	0.01
341	561.30	3	0.01
342	568.30	3	0.01
343	687.20	7	0.02

Line#:31 R.Time:38.0(Scan#:10199)
MassPeaks:413
RawMode:Averaged 37.7-38.0(10107-10211) BasePeak:69(259452)
BG Mode:None Group 1 - Event 1

#	m/z	Abs. Int.	Rel. Int.
1	50.05	910	0.35
2	51.10	2038	0.79
3	52.15	1326	0.51
4	53.10	23821	9.18
5	54.15	8695	3.35
6	55.10	183571	70.75
7	56.10	18078	6.97
8	57.10	81481	31.41
9	58.10	4139	1.60
10	59.10	2492	0.96
11	60.05	1745	0.67
12	61.05	1336	0.51
13	62.10	381	0.15
14	63.00	805	0.31
15	64.15	552	0.21
16	65.05	9485	3.66
17	66.15	4683	1.80
18	67.05	153720	59.25
19	68.10	42240	16.28
20	69.05	259452	100.00
21	70.10	22779	8.78
22	71.10	19841	7.65
23	72.10	2795	1.08
24	73.05	11190	4.31
25	74.10	1932	0.74
26	75.05	3155	1.22
27	76.15	772	0.30
28	77.05	22563	8.70
29	78.15	4806	1.85
30	79.05	74798	28.83
31	80.10	14546	5.61
32	81.10	188679	72.72
33	82.10	83280	32.10
34	83.10	101674	39.19
35	84.10	10808	4.17
36	85.10	11300	4.36

#	m/z	Abs. Int.	Rel. Int.
37	86.05	2929	1.13
38	87.10	1881	0.72
39	88.05	2002	0.77
40	89.05	1897	0.73
41	90.15	557	0.21
42	91.05	54297	20.93
43	92.10	10109	3.90
44	93.10	101868	39.26
45	94.10	29723	11.46
46	95.10	240551	92.72
47	96.10	143971	55.49
48	97.10	57397	22.12
49	98.10	7459	2.87
50	99.05	5525	2.13
51	100.10	1600	0.62
52	101.05	1774	0.68
53	102.05	1976	0.76
54	103.05	4019	1.55
55	104.15	1942	0.75
56	105.05	56756	21.88
57	106.10	15827	6.10
58	107.05	112933	43.53
59	108.10	52152	20.10
60	109.10	204270	78.73
61	110.10	54884	21.15
62	111.10	56470	21.77
63	112.10	5957	2.30
64	113.10	5336	2.06
65	114.10	1795	0.69
66	115.05	5506	2.12
67	116.00	3019	1.16
68	117.05	10740	4.14
69	118.10	3944	1.52
70	119.05	63520	24.48
71	120.10	27463	10.59
72	121.10	108384	41.77

#	m/z	Abs. Int.	Rel. Int.
73	122.10	46017	17.74
74	123.10	180144	69.43
75	124.10	60092	23.16
76	125.10	136634	52.66
77	126.15	14451	5.57
78	127.10	6303	2.43
79	128.10	3886	1.50
80	129.05	6144	2.37
81	130.05	2764	1.07
82	131.05	14967	5.77
83	132.10	11238	4.33
84	133.10	60315	23.25
85	134.10	30975	11.94
86	135.10	78690	30.33
87	136.10	40261	15.52
88	137.10	68886	26.55
89	138.10	23385	9.01
90	139.10	10156	3.91
91	140.05	1959	0.76
92	141.05	2899	1.12
93	142.05	2651	1.02
94	143.00	6604	2.55
95	144.10	3020	1.16
96	145.10	23859	9.20
97	146.10	12872	4.96
98	147.10	50110	19.31
99	148.10	27274	10.51
100	149.10	61287	23.62
101	150.10	32966	12.71
102	151.15	26772	10.32
103	152.10	14178	5.46
104	153.10	4704	1.81
105	154.15	1456	0.56
106	155.05	1948	0.75
107	156.15	1552	0.60
108	157.05	6194	2.39

DEPTT. OF BOTANICAL & ENVIRONMENTAL SCIENCES, G.N.D.U. AMRITSAR

#	m/z	Abs. Int.	Rel. Int.
109	158.10	3216	1.24
110	159.10	20176	7.78
111	160.10	17981	6.93
112	161.10	61788	23.81
113	162.10	20981	8.09
114	163.10	76208	29.37
115	164.10	24692	9.52
116	165.10	14261	5.50
117	166.10	2707	1.04
118	167.10	1553	0.60
119	168.10	745	0.29
120	169.10	1456	0.56
121	170.15	983	0.38
122	171.10	5633	2.17
123	172.10	3758	1.45
124	173.10	17795	6.86
125	174.10	6502	2.51
126	175.10	32528	12.54
127	176.10	14102	5.44
128	177.10	26183	10.09
129	178.10	8864	3.42
130	179.10	46788	18.03
131	180.10	7495	2.89
132	181.10	1551	0.60
133	182.05	553	0.21
134	183.05	1280	0.49
135	184.15	644	0.25
136	185.05	6873	2.65
137	186.10	2955	1.14
138	187.10	17203	6.63
139	188.10	5783	2.23
140	189.10	40042	15.43
141	190.10	17615	6.79
142	191.05	54173	20.88
143	192.10	14295	5.51
144	193.05	14209	5.48
145	194.05	4450	1.72
146	195.05	1740	0.67
147	196.05	436	0.17
148	196.95	1049	0.40
149	198.05	522	0.20
150	199.00	6406	2.47
151	200.10	2664	1.03
152	201.05	13092	5.05
153	202.05	8808	3.39
154	203.05	30677	11.82
155	204.05	31402	12.10
156	205.10	56979	21.96
157	206.10	20877	8.05
158	207.00	27014	10.41
159	208.00	6542	2.52
160	209.00	3132	1.21
161	210.00	661	0.25
162	211.05	955	0.37
163	212.00	424	0.16
164	213.05	5167	1.99
165	214.10	3304	1.27
166	215.05	21673	8.35
167	216.05	6966	2.68
168	217.05	30226	11.65
169	218.05	70207	27.06
170	219.05	20704	7.98
171	220.10	18914	7.29
172	221.05	7492	2.89
173	222.05	2047	0.79
174	223.00	992	0.38
175	224.00	296	0.11
176	225.00	704	0.27
177	226.05	335	0.13
178	227.05	3188	1.23
179	228.05	2032	0.78
180	229.05	15570	6.00
181	230.05	6362	2.45
182	231.05	40716	15.69
183	232.05	28160	10.85
184	233.05	16080	6.20
185	234.05	12529	4.83
186	235.10	3094	1.19
187	236.10	668	0.26
188	237.05	513	0.20

#	m/z	Abs. Int.	Rel. Int.
189	238.10	193	0.07
190	239.05	902	0.35
191	240.15	380	0.15
192	241.05	3125	1.20
193	242.05	1228	0.47
194	243.05	3669	1.41
195	244.05	2512	0.97
196	245.05	16160	6.23
197	246.05	35262	13.59
198	247.05	14200	5.47
199	248.05	12046	4.64
200	249.05	3852	1.48
201	250.00	714	0.28
202	250.90	785	0.30
203	252.00	284	0.11
204	253.00	708	0.27
205	254.15	373	0.14
206	255.05	9778	3.77
207	256.10	2849	1.10
208	257.05	13821	5.33
209	258.05	3450	1.33
210	259.05	9589	3.70
211	260.05	2987	1.15
212	261.10	4504	1.74
213	262.10	1448	0.56
214	263.05	650	0.25
215	264.00	208	0.08
216	264.90	1579	0.61
217	265.90	512	0.20
218	266.90	1892	0.73
219	268.10	574	0.22
220	269.10	4381	1.69
221	270.10	1984	0.76
222	271.10	3944	1.52
223	272.15	2825	1.09
224	273.10	42355	16.32
225	274.05	20222	7.79
226	275.10	8606	3.32
227	276.05	1546	0.60
228	277.10	298	0.11
229	278.10	65	0.03
230	279.10	182	0.07
231	279.90	73	0.03
232	280.90	7238	2.79
233	281.85	2137	0.82
234	283.00	2061	0.79
235	284.05	1855	0.71
236	285.10	2958	1.14
237	286.10	2577	0.99
238	287.10	10624	4.09
239	288.05	2970	1.14
240	289.10	834	0.32
241	290.10	165	0.06
242	291.10	69	0.03
243	292.10	50	0.02
244	293.10	165	0.06
245	294.10	13	0.01
246	294.95	516	0.20
247	296.10	195	0.08
248	297.10	691	0.27
249	298.10	263	0.10
250	299.10	1041	0.40
251	300.10	664	0.26
252	301.15	995	0.38
253	302.10	15644	6.03
254	303.05	6248	2.41
255	304.10	1183	0.46
256	305.10	171	0.07
257	306.10	21	0.01
258	307.10	51	0.02
259	308.10	11	0.00
260	309.10	232	0.09
261	310.10	55	0.02
262	311.10	388	0.15
263	312.10	147	0.06
264	313.10	547	0.21
265	314.05	232	0.09
266	315.10	466	0.18
267	316.20	214	0.08
268	317.10	104	0.04

#	m/z	Abs. Int.	Rel. Int.
269	318.10	15	0.01
270	319.10	31	0.01
271	320.10	6	0.00
272	321.10	38	0.01
273	322.20	12	0.00
274	323.10	275	0.11
275	324.10	105	0.04
276	325.05	552	0.21
277	326.15	279	0.11
278	327.10	1189	0.46
279	328.10	441	0.17
280	329.10	406	0.16
281	330.10	168	0.06
282	331.20	141	0.05
283	332.10	20	0.01
284	333.10	15	0.01
285	334.20	8	0.00
286	335.20	23	0.01
287	336.20	9	0.00
288	337.20	139	0.05
289	338.10	58	0.02
290	339.15	742	0.29
291	340.15	396	0.15
292	341.15	7318	2.82
293	342.10	2218	0.85
294	343.05	608	0.23
295	344.15	1381	0.53
296	345.10	409	0.16
297	346.10	58	0.02
298	347.20	6	0.00
299	351.10	71	0.03
300	352.10	32	0.01
301	353.10	214	0.08
302	353.95	430	0.17
303	354.95	1698	0.65
304	356.00	689	0.27
305	357.00	581	0.22
306	358.00	172	0.07
307	359.10	47	0.02
308	360.10	14	0.01
309	361.00	9	0.00
310	362.00	1	0.00
311	363.00	24	0.01
312	364.10	6	0.00
313	365.00	197	0.08
314	366.00	60	0.02
315	367.00	117	0.05
316	368.00	70	0.03
317	369.15	475	0.18
318	370.05	213	0.08
319	371.00	136	0.05
320	372.00	6	0.00
321	372.90	1	0.00
322	374.00	2	0.00
323	376.00	2	0.00
324	377.10	36	0.01
325	378.20	21	0.01
326	379.20	179	0.07
327	380.20	52	0.02
328	381.10	126	0.05
329	382.00	25	0.01
330	383.15	467	0.18
331	384.10	630	0.24
332	385.10	232	0.09
333	386.10	34	0.01
334	389.10	36	0.01
335	391.10	83	0.03
336	392.10	52	0.02
337	393.10	995	0.38
338	394.10	360	0.14
339	395.10	222	0.09
340	396.10	128	0.05
341	397.10	266	0.10
342	398.20	144	0.06
343	399.20	87	0.03
344	400.10	13	0.01
345	400.90	96	0.04
346	402.10	19	0.01
347	403.10	6	0.00
348	404.00	6	0.00

DEPTT. OF BOTANICAL & ENVIRONMENTAL SCIENCES, G.N.D.U. AMRITSAR

#	m/z	Abs. Int.	Rel. Int.
349	405.10	12	0.00
350	406.10	29	0.01
351	407.10	95	0.04
352	408.10	563	0.22
353	409.15	534	0.21
354	410.15	449	0.17
355	411.10	9001	3.47
356	412.15	2877	1.11
357	413.10	549	0.21
358	414.10	107	0.04
359	415.10	85	0.03
360	416.10	25	0.01
361	417.10	8	0.00
362	418.20	15	0.01
363	421.20	4	0.00
364	422.10	24	0.01
365	423.10	80	0.03
366	424.10	179	0.07
367	425.25	182	0.07
368	426.15	10558	4.07
369	427.15	3750	1.45
370	428.15	743	0.29
371	429.15	479	0.18
372	430.20	195	0.08
373	431.00	80	0.03
374	432.00	2	0.00
375	436.30	2	0.00
376	437.10	3	0.00
377	438.20	28	0.01
378	439.10	25	0.01
379	440.20	140	0.05
380	441.20	21	0.01
381	442.20	26	0.01
382	443.20	6	0.00
383	446.20	24	0.01
384	447.20	8	0.00
385	449.00	2	0.00
386	452.30	1	0.00
387	453.20	145	0.06
388	454.20	57	0.02
389	456.20	1	0.00
390	459.20	1	0.00
391	465.30	1	0.00
392	468.25	289	0.11
393	469.20	82	0.03
394	477.00	1	0.00
395	481.20	2	0.00
396	483.20	1	0.00
397	491.20	1	0.00
398	499.20	8	0.00
399	500.20	4	0.00
400	503.10	13	0.01
401	504.20	2	0.00
402	517.30	4	0.00
403	532.20	2	0.00
404	550.20	2	0.00
405	558.20	4	0.00
406	569.20	1	0.00
407	571.00	4	0.00
408	576.20	1	0.00
409	591.20	1	0.00
410	637.20	2	0.00
411	657.20	2	0.00
412	662.20	2	0.00
413	663.00	2	0.00

Line#:32 R.Time:39.9(Scan#:10777)
MassPeaks:399
RawMode:Averaged 39.8-40.0(10753-10810) BasePeak:203(79236)
BG Mode:None Group 1 - Event 1

#	m/z	Abs. Int.	Rel. Int.
1	50.00	648	0.82
2	51.05	1173	1.48
3	52.15	697	0.88
4	53.10	4043	5.10
5	54.15	1702	2.15
6	55.10	30197	38.11
7	56.10	4076	5.14
8	57.10	19444	24.54
9	58.10	1880	2.37
10	59.05	3202	4.04
11	60.05	2441	3.08
12	61.10	1768	2.23
13	62.10	319	0.40
14	63.10	670	0.85
15	64.10	506	0.64
16	65.10	2530	3.19
17	66.05	1148	1.45
18	67.05	18999	23.98
19	68.10	4287	5.41
20	69.10	28812	36.36
21	70.10	3930	4.96
22	71.10	12560	15.85
23	72.10	1673	2.11
24	73.05	27078	34.17
25	74.05	2835	3.58
26	75.05	4404	5.56
27	76.10	766	0.97
28	77.05	7379	9.31
29	78.10	2034	2.57
30	79.05	16551	20.89
31	80.10	3750	4.73
32	81.10	28940	36.52
33	82.10	6775	8.55
34	83.10	13196	16.65
35	84.10	2835	3.58
36	85.10	7464	9.42
37	86.05	937	1.18
38	87.05	2472	3.12
39	88.00	918	1.16
40	89.00	1786	2.25
41	90.15	467	0.59
42	91.05	17890	22.58
43	92.10	3268	4.12
44	93.10	21557	27.21
45	94.10	6361	8.03
46	95.10	32902	41.52
47	96.10	14262	18.00
48	97.10	11606	14.65
49	98.10	3050	3.85
50	99.10	2868	3.62
51	100.05	1793	2.26
52	101.10	1364	1.72
53	102.00	747	0.94
54	103.05	3494	4.41
55	104.10	1580	1.99
56	105.10	22644	28.58
57	106.10	5839	7.37
58	107.10	22970	28.99
59	108.15	6789	8.57
60	109.10	20715	26.14
61	110.05	4111	5.19
62	111.10	6451	8.14
63	112.10	1592	2.01
64	113.10	1887	2.38
65	114.15	549	0.69
66	115.05	4618	5.83
67	116.05	1945	2.45
68	117.05	9896	12.49
69	118.10	3946	4.98
70	119.10	26420	33.34
71	120.10	9186	11.59
72	121.10	19298	24.36
73	122.10	7757	9.79
74	123.10	14223	17.95
75	124.10	2793	3.52
76	125.10	5009	6.32
77	126.10	1461	1.84
78	127.10	2361	2.98
79	128.05	2558	3.23
80	129.10	5412	6.83
81	130.10	2007	2.53
82	131.10	13037	16.45
83	132.10	6227	7.86
84	133.10	65761	82.99
85	134.10	12615	15.92
86	135.10	18011	22.73
87	136.10	6760	8.53
88	137.10	6507	8.21
89	138.10	1745	2.20
90	139.10	2916	3.68
91	140.10	932	1.18
92	141.10	2263	2.86
93	142.05	1890	2.39
94	143.05	4703	5.94
95	144.10	1851	2.34
96	145.10	10893	13.75
97	146.10	5124	6.47
98	147.10	19178	24.20
99	148.10	6235	7.87
100	149.15	9684	12.22
101	150.15	2558	3.23
102	151.15	3050	3.85
103	152.15	1847	2.33
104	153.10	1770	2.23
105	154.10	1147	1.45
106	155.10	2053	2.59
107	156.10	1269	1.60
108	157.10	3874	4.89
109	158.10	1596	2.01
110	159.10	8200	10.35
111	160.10	2912	3.68
112	161.10	10038	12.67
113	162.10	3517	4.44
114	163.10	8099	10.22
115	164.15	1947	2.46
116	165.10	3035	3.83
117	166.10	1058	1.34
118	167.10	1236	1.56
119	168.15	685	0.86
120	169.10	1536	1.94
121	170.10	733	0.93
122	171.10	3090	3.90
123	172.10	1218	1.54
124	173.10	5656	7.14
125	174.15	2455	3.10
126	175.10	9985	12.60
127	176.10	3885	4.90
128	177.10	7557	9.54
129	178.05	1882	2.38
130	179.05	2775	3.50
131	180.05	695	0.88
132	181.10	900	1.14
133	182.00	470	0.59
134	183.05	1358	1.71
135	184.05	521	0.66
136	185.05	2630	3.32
137	186.10	1242	1.57
138	187.10	7954	10.04
139	188.10	2768	3.49
140	189.10	16297	20.57
141	190.05	6774	8.55
142	191.00	13613	17.18
143	192.05	3064	3.87
144	193.00	4899	6.18
145	194.00	1151	1.45
146	195.05	1083	1.37
147	196.00	440	0.56
148	197.05	1080	1.36
149	198.05	559	0.71
150	199.05	2128	2.69
151	200.10	926	1.17
152	201.05	4988	6.30
153	202.15	3848	4.86
154	203.10	79236	100.00
155	204.10	15090	19.04
156	205.10	5675	7.16

DEPTT. OF BOTANICAL & ENVIRONMENTAL SCIENCES, G.N.D.U. AMRITSAR

#	m/z	Abs. Int.	Rel. Int.
157	206.05	2262	2.85
158	207.00	58577	73.93
159	208.00	12307	15.53
160	208.95	6497	8.20
161	210.00	1211	1.53
162	211.05	1174	1.48
163	212.10	440	0.56
164	213.10	1837	2.32
165	214.10	826	1.04
166	215.10	2835	3.58
167	216.10	4017	5.07
168	217.05	3418	4.31
169	218.15	7327	9.25
170	219.10	4242	5.35
171	220.05	2113	2.67
172	221.05	5440	6.87
173	222.05	1649	2.08
174	223.00	1506	1.90
175	224.00	445	0.56
176	225.10	748	0.94
177	226.00	325	0.41
178	227.05	1307	1.65
179	228.10	599	0.76
180	229.10	2102	2.65
181	230.10	692	0.87
182	231.10	1278	1.61
183	232.05	1859	2.35
184	233.05	1265	1.60
185	234.10	7488	9.45
186	235.05	2245	2.83
187	236.00	600	0.76
188	237.10	617	0.78
189	238.10	248	0.31
190	239.05	870	1.10
191	240.10	377	0.48
192	241.05	1346	1.70
193	242.10	512	0.65
194	243.05	972	1.23
195	244.10	372	0.47
196	245.10	686	0.87
197	246.10	369	0.47
198	247.10	826	1.04
199	247.95	1831	2.31
200	248.90	3455	4.36
201	249.90	929	1.17
202	250.90	1560	1.97
203	251.90	474	0.60
204	253.05	1042	1.32
205	254.00	376	0.47
206	255.10	1644	2.07
207	256.05	584	0.74
208	257.10	1406	1.77
209	258.10	458	0.58
210	259.10	815	1.03
211	260.10	323	0.41
212	261.00	351	0.44
213	262.10	164	0.21
214	263.10	333	0.42
215	263.90	128	0.16
216	264.90	4098	5.17
217	265.95	1196	1.51
218	266.90	4397	5.55
219	267.90	1220	1.54
220	269.05	1455	1.84
221	270.10	821	1.04
222	271.10	1029	1.30
223	272.10	536	0.68
224	273.15	1554	1.96
225	274.10	553	0.70
226	275.10	635	0.80
227	276.10	220	0.28
228	277.00	290	0.37
229	278.00	99	0.12
230	279.10	363	0.46
231	279.90	123	0.16
232	280.95	18576	23.44
233	281.90	5156	6.51
234	282.95	3640	4.59
235	284.00	904	1.14
236	285.00	627	0.79

#	m/z	Abs. Int.	Rel. Int.
237	286.10	688	0.87
238	287.10	545	0.69
239	288.10	362	0.46
240	288.90	284	0.36
241	290.00	83	0.10
242	291.10	291	0.37
243	292.10	54	0.07
244	293.10	319	0.40
245	293.90	22	0.03
246	295.00	949	1.20
247	296.00	405	0.51
248	297.05	610	0.77
249	298.00	215	0.27
250	299.20	319	0.40
251	300.10	195	0.25
252	301.10	205	0.26
253	302.20	154	0.19
254	303.20	166	0.21
255	304.90	40	0.05
256	306.10	24	0.03
257	307.20	127	0.16
258	308.00	3	0.00
259	309.20	421	0.53
260	310.10	136	0.17
261	311.10	311	0.39
262	312.20	62	0.08
263	313.00	309	0.39
264	314.10	127	0.16
265	315.20	152	0.19
266	316.00	29	0.04
267	317.10	56	0.07
268	318.10	7	0.01
269	318.90	11	0.01
270	320.00	3	0.00
271	321.10	62	0.08
272	322.20	11	0.01
273	323.10	306	0.39
274	324.10	140	0.18
275	324.95	723	0.91
276	326.10	285	0.36
277	326.85	707	0.89
278	328.00	248	0.31
279	329.20	259	0.33
280	330.00	26	0.03
281	331.10	133	0.17
282	333.90	12	0.02
283	336.20	3	0.00
284	337.20	47	0.06
285	338.10	24	0.03
286	339.10	362	0.46
287	339.90	146	0.18
288	340.90	1964	2.48
289	341.90	663	0.84
290	342.90	460	0.58
291	343.90	106	0.13
292	344.90	30	0.04
293	350.90	12	0.02
294	351.90	14	0.02
295	352.90	208	0.26
296	354.00	82	0.10
297	354.90	3334	4.21
298	355.90	1272	1.61
299	356.90	880	1.11
300	358.00	305	0.38
301	359.00	71	0.09
302	359.90	20	0.03
303	363.00	16	0.02
304	364.90	40	0.05
305	365.90	29	0.04
306	367.00	135	0.17
307	367.90	33	0.04
308	368.90	378	0.48
309	370.00	110	0.14
310	370.90	85	0.11
311	372.00	15	0.02
312	372.90	3	0.00
313	376.90	3	0.00
314	377.90	7	0.01
315	378.90	14	0.02
316	381.00	174	0.22

#	m/z	Abs. Int.	Rel. Int.
317	382.00	54	0.07
318	383.00	105	0.13
319	384.25	188	0.24
320	385.00	132	0.17
321	385.90	22	0.03
322	387.00	18	0.02
323	390.00	3	0.00
324	390.90	118	0.15
325	392.00	34	0.04
326	393.20	439	0.55
327	393.90	160	0.20
328	395.20	55	0.07
329	396.20	120	0.15
330	397.20	111	0.14
331	398.00	7	0.01
332	399.00	108	0.14
333	400.20	25	0.03
334	400.90	384	0.48
335	401.90	98	0.12
336	403.00	86	0.11
337	403.90	3	0.00
338	404.90	3	0.00
339	406.00	6	0.01
340	407.00	97	0.12
341	408.00	170	0.21
342	409.20	525	0.66
343	410.20	235	0.30
344	411.20	864	1.09
345	412.20	289	0.36
346	413.20	64	0.08
347	414.20	25	0.03
348	414.90	355	0.45
349	416.00	75	0.09
350	416.90	47	0.06
351	417.90	23	0.03
352	419.20	3	0.00
353	422.20	30	0.04
354	423.20	38	0.05
355	424.20	350	0.44
356	425.20	199	0.25
357	426.20	163	0.21
358	427.20	545	0.69
359	428.10	190	0.24
360	428.90	1166	1.47
361	429.90	558	0.70
362	430.90	378	0.48
363	432.00	77	0.10
364	433.20	11	0.01
365	439.90	11	0.01
366	440.90	11	0.01
367	441.90	172	0.22
368	443.10	53	0.07
369	443.90	7	0.01
370	454.90	3	0.00
371	458.90	7	0.01
372	459.90	4	0.01
373	460.90	24	0.03
374	461.90	15	0.02
375	464.90	7	0.01
376	467.00	3	0.00
377	474.90	20	0.03
378	475.90	7	0.01
379	482.90	3	0.00
380	484.90	7	0.01
381	485.90	3	0.00
382	486.90	3	0.00
383	489.90	3	0.00
384	493.90	3	0.00
385	497.90	79	0.10
386	498.90	15	0.02
387	502.90	298	0.38
388	503.90	101	0.13
389	505.00	65	0.08
390	507.90	3	0.00
391	511.00	3	0.00
392	513.90	4	0.01
393	539.00	3	0.00
394	556.90	3	0.00
395	578.90	3	0.00
396	596.10	3	0.00

DEPTT. OF BOTANICAL & ENVIRONMENTAL SCIENCES, G.N.D.U. AMRITSAR

#	m/z	Abs. Int.	Rel. Int.	#	m/z	Abs. Int.	Rel. Int.	#	m/z	Abs. Int.	Rel. Int.
397	618.90	3	0.00	398	660.90	3	0.00	399	666.90	3	0.00

Peak Report TIC

Peak#	R.Time	Area	Area%	Height	Name
1	8.074	1913911	0.54	997218	1-Dodecene (CAS) Adacene 12 $$ n-Dodec-1-ene $$ .alpha.-Dodecene $$ dodecene $$ n-undecane. 1-dodecene $$ Dodec-1-e
2	12.040	3232215	0.91	2164180	1-Tetradecene (CAS) n-Tetradec-1-ene $$ .alpha.-Tetradecene $$ 1-Butadecene $$ Dialene 14 $$
3	12.210	492867	0.14	367370	Tetradecane (CAS) n-Tetradecane $$ Isotetradecane $$
4	13.586	3049277	0.86	2177412	Phenol, 2,4-bis(1,1-dimethylethyl)- (CAS) 2,4-Di-tert-butylphenol $$ 2,4-BIS(TERT-BUTYL)-PHENOL $$ 2,4-Di-t-butylphen
5	14.826	3960029	1.12	2788280	9-Eicosene, (E)- $$ (9E)-9-Icosene # $$
6	16.732	585213	0.17	333000	9-Octadecenoic acid (Z)- (CAS) Oleic acid $$ Red oil $$ Oelsauere $$ Oleine 7503 $$ Pamolyn 100 $$ Emersol 211 $$ Vopco
7	17.154	4088803	1.16	2880182	3-Eicosene, (E)- (CAS)
8	17.644	3987191	1.13	2550499	NEOPHYTADIENE $$ 2,6,10-TRIMETHYL,14-ETHYLENE-14-PENTADECNE $$
9	17.892	641645	0.18	435833	3,7,11,15-Tetramethyl-2-hexadecen-1-ol $$ (2E)-3,7,11,15-Tetramethyl-2-hexadecen-1-ol # $$
10	18.076	1070590	0.30	722972	7-Octadecyne, 2-methyl- $$ 2-Methyl-7-octadecyne $$
11	18.494	1453335	0.41	1031709	Butyl-2-methylpropylphthalate @ P1351
12	18.845	4546141	1.29	1845016	Pentadecanoic acid (CAS) Pentadecylic acid $$ n-Pentadecanoic acid $$ n-Pentadecylic acid $$ Pentadecyclic acid $$ PENTA
13	19.220	2708377	0.77	1990314	Pentadecyl trifluoroacetate
14	20.262	2293976	0.65	1414962	2-Hexadecen-1-ol, 3,7,11,15-tetramethyl-, [R-[R*,R*-(E)]]- (CAS) Phytol $$ trans-Phytol $$ (E)-(7R,11R)-3,7,11,15-tetrameth
15	20.463	680267	0.19	557014	Linoleic acid P1365
16	20.716	1200822	0.34	722193	Eicosanoic acid $$ Arachic acid $$ Arachidic acid $$ Icosanoic acid $$ n-Eicosanoic acid $$ Arachidic acid (synthetic) $$
17	21.096	1720937	0.49	1190611	9-Tricosene, (Z)- (CAS) Muscalure $$ cis-9-Tricosene $$ (Z)-9-Tricosene $$ (9Z)-Tricosene $$
18	22.814	994738	0.28	703217	1-Docosanol (CAS) Behenic alcohol $$ Behenyl alcohol $$ Docosyl alcohol $$ Docosanol-(1) $$
19	23.751	1048413	0.30	762022	1,2-Benzenedicarboxylic acid, dioctyl ester (CAS) Dioctyl phthalate $$ Dinopol NOP $$ Polycizer 162 $$ n-Octyl phthalate $$
20	24.423	662325	0.19	419840	Nonadecyl pentafluoropropionate
21	25.395	1349088	0.38	745185	Octadecane P1197
22	26.584	2642591	0.75	1165448	2,6,10,14,18,22-Tetracosahexaene, 2,6,10,15,19,23-hexamethyl-, (all-E)- $$ All-trans-Squalene $$ trans-Squalene $$ Spinacen
23	27.536	929162	0.26	321125	17-Pentatriacontene $$ (17E)-17-Pentatriacontene # $$
24	27.766	1331431	0.38	513895	Nonadecane (CAS) n-Nonadecane $$
25	31.156	5760571	1.63	1060899	Vitamin E $$ 2H-1-Benzopyran-6-ol, 3,4-dihydro-2,5,7,8-tetramethyl-2-(4,8,12-trimethyltridecyl)-, [2R-[2R*(4R*,8R*)]]- (CA
26	33.729	32083174	9.07	4119282	D:B-Friedo-18,19-secolup-19-ene, 3,10-epoxy-, (3.beta.,10.beta.)- $$ Baccharis oxide $$
27	35.200	8433097	2.38	1471158	Stigmast-5-en-3-ol, (3.beta.)- (CAS) 24.BETA.-ETHYL-5.DELTA.-CHOLESTEN-3.BETA.-OL $$ SKF 14463 $$ Rhamnol $
28	35.469	29375003	8.30	5193377	METHYL COMMATE C $$
29	36.329	119303169	33.72	11166984	METHYL COMMATE D $$
30	37.215	7362453	2.08	2039394	03027205002 FLAVONE 4'-OH,5-OH,7-DI-O-GLUCOSIDE $$
31	37.993	99118149	28.02	16485800	GLOBULOL $$ (-)-Globulol $$ 1H-Cycloprop[e]azulen-4-ol, decahydro-1,1,4,7-tetramethyl-, [1aR-(1a.alpha.,4.alpha.,4a.alpha
32	39.919	5740082	1.62	1413838	Urs-12-en-28-ol
		353759042	100.00	71750229	
